# The Exploration of Novel Regulatory Relationships Drives Haloarchaeal Operon-Like Structural Dynamics over Short Evolutionary Distances

**DOI:** 10.3390/microorganisms8121900

**Published:** 2020-11-30

**Authors:** Phillip Seitzer, Andrew I. Yao, Ariana Cisneros, Marc T. Facciotti

**Affiliations:** 1 UC Davis Genome Center, UC Davis, Davis, CA 95616, USA; phillipseitzer@gmail.com (P.S.); aiyao@ucdavis.edu (A.I.Y.); accisneros@ucdavis.edu (A.C.); 2 Department of Biomedical Engineering, UC Davis, Davis, CA 95616, USA; 3 Calico Life Sciences, South San Francisco, CA 94080, USA

**Keywords:** haloarchaea, operon, archaea, genomics, evolution

## Abstract

Operons are a dominant feature of bacterial and archaeal genome organization. Numerous investigations have related aspects of operon structure to operon function, making operons exemplars for studies aimed at deciphering Nature’s design principles for genomic organization at a local scale. We consider this understanding to be both fundamentally important and ultimately useful in the de novo design of increasingly complex synthetic circuits. Here we analyze the evolution of the genomic context of operon-like structures in a set of 76 sequenced and annotated species of halophilic archaea. The phylogenetic depth and breadth of this dataset allows insight into changes in operon-like structures over shorter evolutionary time scales than have been studied in previous cross-species analysis of operon evolution. Our analysis, implemented in the updated software package JContextExplorer finds that operon-like context as measured by changes in structure frequently differs from a sequence divergence model of whole-species phylogeny and that changes seem to be dominated by the exploration of novel regulatory relationships.

## 1. Introduction

An operon is a collection of genes that are co-transcribed to form a single mRNA molecule in at least some set of biologically relevant conditions. It is useful to think of an operon as a special case of a more general “genomic context”—a pattern of tight gene organization existing at a local genomic scale—with the additional constraint that operonic genes are at least sometimes co-transcribed. Operons are highly prevalent in bacterial and archaeal genomes (on the order of 50% of genes exist in operons in these organisms [1,2]), and are also present in a number of eukaryotic species [3,4]. Operons often code for genes in the same functional pathway [5,6], are often evolutionarily conserved [7,8], but may also be acquired by horizontal gene transfer [9]. The specific selective pressures and mechanisms driving operon formation have been the subject of some debate. For instance, it has been proposed that the organization of genes into operons may reduce the probability of obtaining unfavorable combinations of genes by recombination [10,11], genes might be organized into operons because of some benefit derived through co-regulation [9,12], or operons might develop “selfishly” through progressive rounds of horizontal gene transfer [13]. Several investigations suggest that operons evolve, either in response to selective pressures imposed by the presence or absence of other genomic elements [14], changing regulatory networks [9], or other forces [15].

The number of publicly available, completely sequenced microbial genomes has increased dramatically, and this has stimulated several large-scale cross-species informatic analyses of bacterial and archaeal operons [5,16,17,18,19]. While these investigations have focused primarily on differences in the gene content between homologous operons, other functionally relevant structural factors may also be examined from biosequences data: these include the order of genes within an operon, the size of intergenic gaps (or degree of overlap) occurring between individual genes within an operon, and the presence of internal regulatory sequences. Intra-operonic gene order is in some cases associated with gene expression levels. All other things being equal, genes in operons that are transcribed earlier tend to be expressed at higher levels than those transcribed later [20]. In addition, genes encoding physically interacting protein products tend to demonstrate conserved gene order [21], and gene order can also be associated with temporal expression profiles [22]. Within metabolic operons, one study discovered that genes tend to appear in operons in the order that their protein products function in metabolism [23]. Moreover, the intergenic distance between genes and the presence of internal regulatory motifs can be excellent indicators of an operons’ regulatory plasticity (the ability for some but not all genes in an operon to be expressed). For instance, gene expression studies in *Escherichia coli* and *Bacillus subtilis* revealed a strong correlation between intergenic spacing and degree of co-expression [24], while in the archaeal domain, tiling microarray experiments in the haloarchaeon *Halobacterium* sp. NRC-1 revealed that internal promoters are often present in operons whose genes are conditionally expressed [25].

**Figure 1:**
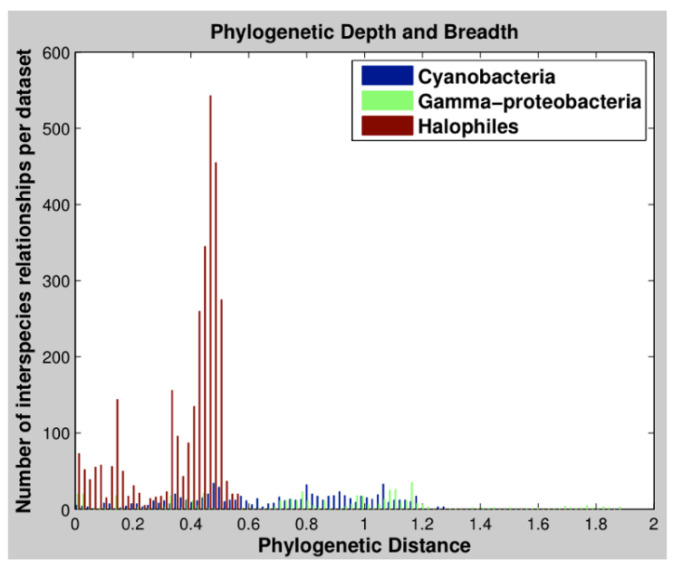
Comparative phylogenetic depth and breadth of datasets used in OLS comparison analyses. Phylogenetic distances were determined between all organisms used in two previous cross-species OLS comparisons analyses as well as the organisms we used in this study (see Methods). The organisms used in our investigation (“halophiles”, red) offer less breadth than those used in previous studies (“cyanobacteria”, blue, and “gamma-proteobacteria”, green), however, provide significantly more depth. This additional depth allows us to observe many more types of OLS modification, and increases the confidence with which report our results.

In this study, we investigate the evolution of computationally predicted operon-like structures in 76 haloarchaeal species [26]. We use the term “operon-like structures” (OLS) to denote that the genomic contexts that we compare have been computationally predicted from sequence data alone and not verified experimentally as bona fide operons. The presence of deeply sequenced subclades within our dataset facilitates the systematic evaluation of changes in multiple OLS features, including gene content, gene order, promoter content, and intergenic gene spacing. We were also able to identify cases of rapid evolution of OLSs that would be more difficult to observe in previously published bacterial datasets with shallower phylogenetic sampling [15,16] (Figure 1). In our analysis, we found that OLS context as measured by changes in composition and structure frequently differs from a sequence divergence model of whole-species phylogeny and that changes seem to be dominated by the exploration of novel regulatory relationships.

## 2. Materials and Methods 

### 2.1. The Haloarchaeal Genome Collection, Homolog Clustering, and Promoter Prediction

The genomic sequences, annotations, and homology clusters for 76 haloarchaeal species were determined, as described in [26] (NCBI accession numbers are available in Table S1). Translated coding regions in all organisms were extracted, and homologous proteins were identified using an all-versus-all protein BLAST scan. Homology clusters were generated using tribe-MCL [27], as described in Becker et al. [26]. A phylogenetic tree of all organisms was constructed by aligning a concatenated set of 38 single-copy conserved PHYECO marker genes (BA00001-34, BA00036-37, BA00039-40) and creating a maximum likelihood phylogenetic tree using FastTree 2 [28] with a WAG protein substitution model. Pairwise phylogenetic distances between organisms were computed by summing the shortest branch lengths between each pair of genomes, as described in Becker et al. [26].

To predict putative promoter locations in all 76 haloarchaeal species, we used the FIMO program [29] to scan a 14 nt model of the DNA binding site for the archaeal general transcription factors TATA-binding protein (TBP) and transcription factor B (TFB), as described in [30], across the genomes of all 76 haloarchaeal species in this dataset. FIMO was run using a 3rd-order Markov background model built from the genome over which it was scanned, with a *p*-value threshold of 1 and a *q*-value threshold of 0.001.

### 2.2. Prediction of OLSs

The challenges associated with experimentally determining OLSs for numerous sequenced organisms [16] have inspired a wealth of computational approaches, which seek to infer OLSs from DNA sequence alone. These methods use structural features such as intergenic distance, conservation of gene order, and the presence of sequence-based features such as promoters and terminators, either alone or in combination, in their predictions [19,31]. Some methods incorporate differential gene expression data and use experimental data collected from one organism to infer OLSs in other organisms [32,33,34]. While operon prediction schemes have become increasingly sophisticated, a survey comparing the performance of popular computational approaches for operon prediction to that of experimentally determined OLSs showed that incorporating factors beyond intergenic distance alone may offer little to no performance benefit [31], especially given the relative lack of experimental data available for the haloarchaea whose genomes are compared herein. Based on this result, we define OLSs simply as clusters of gene encoded on the same strand of DNA with an intergenic spacing between immediate neighbors of 50 nucleotides or less.

### 2.3. Trajectories and Associated Analyses of OLSs

We were interested to use both the phylogenetic breadth and depth of the sampled genomic dataset to study the microevolution of OLS in the haloarchaea. To this aim, we identified homologous OLSs (i.e., OLSs with homologous genes) across species and quantitatively compared their structures. To compare changes in homologous OLS across species, we defined a new data structure we term an “OLS trajectory”, a group of putative homologous OLSs derived from a starting “seed” gene. OLS trajectories were created by first identifying all protein families found in at least 10 of the 76 haloarchaeal species containing no more than a total of 100 protein instances in all organisms. These criteria ensured that each protein family selected for analysis was well represented and that the protein family was not so large as to contain a large number of paralogs (which might make it difficult to determine proper orthology mappings). OLSs were predicted for all protein instances in these protein families using the method described in Prediction of OLSs (above). All protein families featuring at least one multigene (two or more genes) OLSs were retained. Finally, all OLS instances for a given protein family deriving from the same species (homologous gene copies that belong to the same cluster in the same species) were combined into a single grouping of genes. We did not attempt to split paralogs in orthology association to capture potential gene duplication events, and to avoid misassociating paralogous genes across species. Application of this method resulted in the identification of 4005 OLS trajectories.

**Figure 2:**
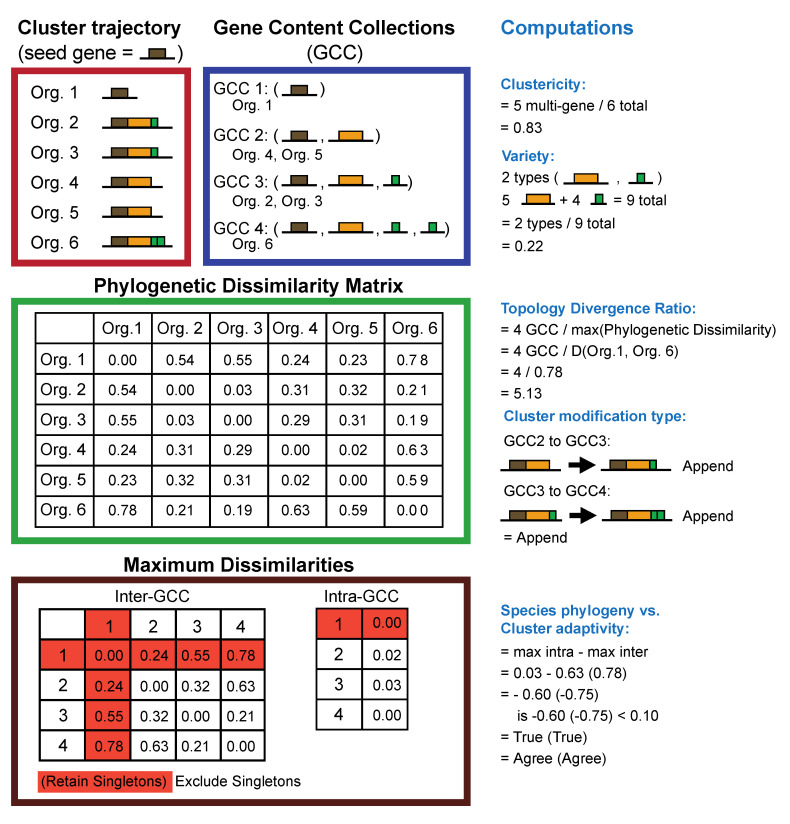
Visualization of Context Trajectory Computations. Computations applied to context trajectories are exemplified in a theoretical context trajectory: OLSs are found in six organisms described as Orgs 1–6, and contain between one and four genes (red box). This trajectory contains four gene content collections (blue box), and clustericity and variety scores of 0.83 and 0.22 (top right), respectively. Based on a theoretical matrix of species-level phylogenetic dissimilarities (green box), this trajectory has a topology divergence ratio of 5.13 (middle right). Evaluation of the gene content collections in this trajectory indicate that this context trajectory exhibits an OLS modification type of “Append” (middle right). A comparison of the dissimilarities between organisms found both within and outside of the gene content collections (brown box) enables a comparison of species phylogeny and OLS adaptivity (bottom right), which is evaluated both retaining and excluding singleton genes (red highlighter, brown box). In this example, the species-level phylogeny is consistent with differences in OLS topology between species whether or not singleton genes are excluded.

We defined several metrics to quantitatively describe differences in gene content among OLSs within a single context trajectory (a visual demonstration of these methods applied to a theoretical context trajectory, is available in Figure 2).

“Clustericity”, the frequency that the seed gene occurs in a multigene OLS relative to the number of all its occurrences (multigene OLSs and as gene singletons); “variety”, the ratio of homology group types to the total count of genes accompanying the seed gene in all OLSs; and “topology divergence ratio”, the ratio of the number of different types of gene content collections to the largest phylogenetic distance between the organisms represented in the trajectory (i.e., how do differences in OLS topology relate to a species phylogeny?). Scatter plots were generated comparing these three metrics (Table S2).

We also modeled changes in OLS that occur via the addition of a single gene either to the head, tail, or an internal site. OLSs within a context trajectory were segregated into non-overlapping clusters based strictly on gene content, and all pairs of gene content clusters that differed by a single gene and each contained at least two genes were determined. Second, for each of these OLS pairs, the position (head, tail, or internal) of the unique gene in the largest of the pair was identified. While we cannot explicitly distinguish between gene gain or loss events, for convenience, observations were always scored from the perspective of gene gain: as a “prepend” if the unique gene was found to be at the head of the OLS, an “append” if the unique gene was found the in the tail, and an “insertion” if the unique gene was found at another location.

Species evolution is most often evaluated by applying a sequence divergence model of evolution to an alignment of gene or protein sequences [35]. Since OLS can also be assumed to be subject to selection [15], we were curious to compare how patterns of OLS evolution, as measured by differences in metrics of OLS (i.e., gene content, gene order, gene spacing), compare to species divergence patterns of their host haloarchaea (as measured by a protein sequence-derived species phylogeny). Similar patterns would indicate that similar selective pressures constrain OLSs and protein evolution, while differences between OLS and species trees could indicate that OLS evolution may be decoupled from the evolution of the genes they encode. To investigate this question, individual OLSs within a context trajectory were segregated into non-overlapping clusters of identical protein family representation. For each context trajectory, this associated organisms into clusters on the basis of similar evolution of OLSs associated with each homologous seed gene. The phylogenetic distance between all organisms within each cluster was compared to the phylogenetic distance between each organism within a cluster and every organism outside of the cluster. If the maximum intra-cluster phylogenetic distance was found to exceed the maximum inter-cluster phylogenetic distance by more than 0.10, a disagreement was recorded between the species phylogeny and the context trajectory. This procedure was carried out both including and excluding gene singletons from OLS trajectories.

### 2.4. Comparing Changes in OLSs with JContextExplorer

**Figure 3:**
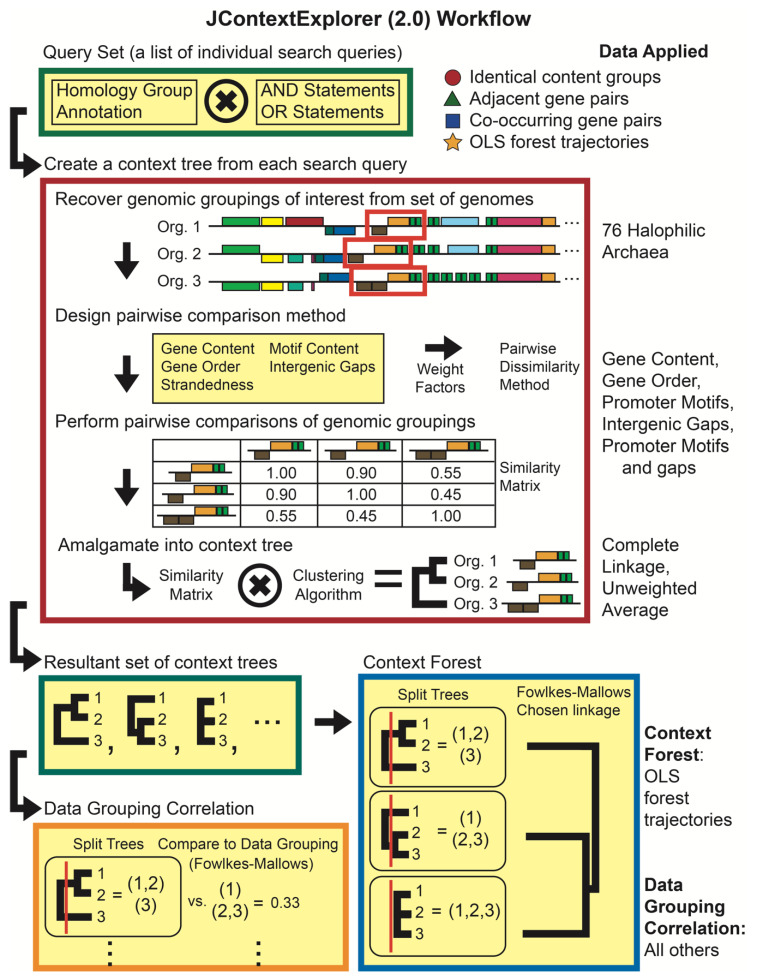
JContextExplorer 2.0 workflow and its application to 76 haloarchaeal species. The previously described JContextExplorer workflow (red box, for detailed description please see [36] and File S3) was extended to allow for the generation of a large set of context trees, which could then be correlated with external data (data grouping correlation, orange box), or to each other (context forest, blue box). In the figure, all boxes with yellow background represent additions to the original algorithm (note the addition of novel comparison metrics, yellow box within red box). The updated approach was applied to a dataset of 76 haloarchaeal species in various ways to determine the degree to which various types of OLS evolution had occurred (textual descriptions at right describe applications of the tool).

We previously developed JContextExplorer [36] to quantitatively compare the local genomic topology of gene groupings across the genomes of multiple species using a variable-group agglomerative hierarchical clustering algorithm. In this paper we report the implementation of several new features that we have used to investigate changes in OLS across the haloarchaeal genomes. These new features allow the user to create custom metrics to define biologically interesting groups of genes, compare topological features (i.e., gene content, gap sizes, etc.) of these groups to each other, associate promoter and protein-binding site information with individual genes, associate and compare context trees to imported phylogenetic trees, correlate a set of context trees to external data, and finally, draw variable-group agglomerative hierarchically clustered trees where the leaves are context trees themselves—creating what we term a “context forest”. The updated JContextExplorer workflow (Figure 3) is well suited for quantitatively investigating cross-species changes in homologous gene groupings (not limited to OLSs).

Briefly, JContextExplorer may be applied to a database of sequenced, annotated, and homology-clustered genomes in the following way: A “query set” is defined that contains a list of individual search queries. Each search query is associated with one or more protein families (Figure 3, green box). For every query in the query set, individual gene matches are retrieved from the database, and a “context set” algorithm extends gene matches to a larger collection of genes (termed a “genomic grouping”), based on criteria specified in the context set. An all-versus-all comparison of genomic groupings is undertaken, where comparisons between individual genomic groupings are specified in a “pairwise comparison method”. These comparisons are then amalgamated into a branching diagram (a “context tree”) based on the specified clustering algorithm and variable-group agglomerative hierarchical clustering method [37] (Figure 3, red box). The set of context trees may then be compared to either a species-specific grouping associated with external data using the Fowlkes–Mallows comparison method [38] (a “data grouping correlation”, Figure 3, orange box), or to each other, producing a similarity matrix that may then be amalgamated into another branching diagram using once again a specified clustering algorithm and variable-group agglomerative hierarchical clustering (a “context forest”, Figure 3, blue box). A more detailed description of the JContextExplorer algorithm (and software implementation) is available in (File S3).

JContextExplorer was applied to the haloarchaeal genomes to investigate four distinct phenomena: (1) OLS gene order, (2) OLS promoter content, (3) intergenic gap sizes of genes in the same OLS, and (4) patterns of gene content modification. To investigate these phenomena, four distinct query sets (Figure 3, upper right-hand corner, red circle, green triangle, blue square, orange star) were created, three different context sets, six different pairwise dissimilarity metrics, and two different clustering approaches were used, and ten data grouping correlations were performed (Figure 3, right side). In the following descriptions, the data grouping termed “single group” is used to indicate that no difference in data grouping correlation exists between species (agreement between a single context tree and this grouping indicates that the context tree organizes all genomic groupings into a single, unified group).

### 2.5. Evaluating Changes in Gene Order Among OLS Trajectories

A list of all pairs of haloarchaeal protein families with gene instances co-occurring in at least one predicted OLS was determined and assembled into a query set, which we refer to as the “co-occurring gene pairs” query set (Figure 3 and Table S4). Query matches were modified using the set of all predicted OLS as a context set, and the resulting genomic groupings were compared using a custom dissimilarity metric consisting only of “Linear Gene Order” dissimilarity with a linear amalgamation and unweighted average clustering (for more information, please see File S3). OLS trees could be constructed for 8136 of the 17,547 pairs in the co-occurring gene pairs query set. A data grouping correlation was carried out comparing these 8136 OLS trees to the “single group” data grouping, with a Fowlkes–Mallows segmentation threshold (*k*-value) of 0.05.

### 2.6. Evaluating Changes in Promoter Motif Construction

The presence of one or more significant sequence matches to a previously determined asymmetric 14 nt BRE-TATA sequence motif [30] was used as a proxy for experimentally determined promoter sites. Promoter motif instances were loaded together with and mapped onto the haloarchaeal genomes in JContextExplorer. Motif instances were associated as putative promoters of individual genes if the center of the motif lay no further than 200 nt before and no further than 50 nt after the predicted start site of the gene (a previous study determined that more than 75% of experimentally determined transcription start sites lie within this distance from the start codon in the archaeon *Halobacterium* sp. NRC-1 [25]). Additionally, we required that the directionality of the motif matched the directionality of the gene with which it was associated.

A query set was created containing all multigene OLSs with identical gene content in all species in which they occur, where at least two OLS instances were observed, which we will refer to from this point onwards as the “identical content groups” query set, which contained a total of 4086 queries (Figure 3 and Table S5). Query matches were modified using the aforementioned predicted OLSs as a context set. Three custom dissimilarity metrics were created, each consisting only of “Presence/Absence of Common Motifs” using a linear amalgamation: in the first case, all motifs were tabulated, in the second, only motifs that occurred at the head of a predicted OLS were tabulated, and in the third case, only motifs that occurred at an internal gene start site within a predicted OLS were tabulated. Using each of these three dissimilarity metrics and unweighted average clustering, OLS trees were constructed for all 4086 queries. A data grouping correlation was carried out comparing each set of context trees to the “single group” data grouping, with a segmentation threshold of 0.05.

### 2.7. Evaluating Changes in Intergenic Spacing

A query set was created containing all collinear pairs of genes that were found to exist in an OLS in at least one species, which we will refer to from this point onwards as the “adjacent gene pairs” query set (Figure 3 and Table S6). This set contained 11,880 queries. Query matches were modified using the “Group all genes between two queries together” context set, with the additional requirements that all gene pairs had to be on the same strand, have no genes between them, and have gene centers not separated by more than 10 kb. A custom dissimilarity metric was created using linear amalgamation with the “changes in intergenic gap size” option, with the dissimilarity being the product of 0.01 and the difference in intergenic gap size between gene pairs. Context trees were assembled from pairwise dissimilarities using variable-group agglomerative hierarchical clustering with complete linkage. Several data grouping correlations were carried out comparing the set of context trees to the “single group” data grouping, varying the segmentation threshold values corresponding to changes in intergenic gap sizes of 5, 10, 20, 50, and 100 nt.

We also investigated variation in intergenic gap size between genes contained in instances of predicted OLSs that did not change in gene content, using the “identical content groups” query set. Query matches were modified with the aforementioned predicted OLSs as a context set, and a custom dissimilarity metric was created, consisting of the “changes in intergenic gap size” option with a threshold at 30 nt (a difference in intergenic gap size of size greater than or equal to 30 nt was scored as a dissimilarity of 1, otherwise, a dissimilarity of 0 was recorded). Context trees were assembled from pairwise dissimilarities using unweighted average clustering. A data grouping correlation were carried out comparing the set of context trees to the “single group” data grouping, at a segmentation threshold of 0.05. We discovered that 466/4086 (11.4%) of these context trees displayed such a change in intergenic distance, and so a custom script was created to determine when widening in intergenic gap correlated to the appearance of one or more internal promoter motifs. We found such a correlation to occur in 108/466 (23.2%) of cases.

## 3. Results

### 3.1. The Evolutionary Dynamics of Haloarchaeal OLS Structure

#### 3.1.1. Clustericity, Variety, and Evolutionary Rate of OLS Trajectories

**Figure 4:**
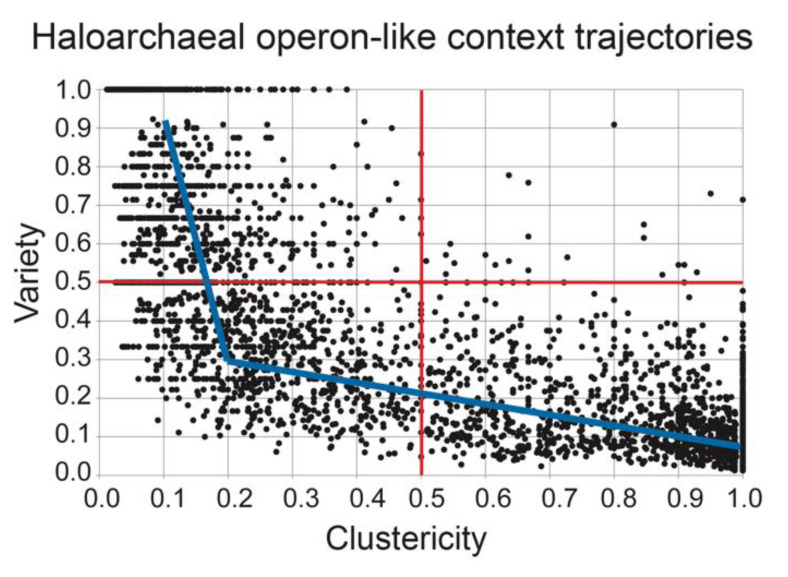
High clustericity and high variety is rare among OLS trajectories. A comparison of the haloarchaeal OLS trajectories revealed a large distribution of behavior with respect to clustericity and variety. In the above plot, each data point is a single context trajectory. Red lines segregate into 4 quadrants based on high or low clustericity paired with high or low variety. Note that the quadrant (high clustericity and high variety) is sparsely populated compared to the other three quadrants. This suggests that when many OLS gene content modifications occur (high variety), a large number of singleton gene states are also observed (decreased clustericity). A blue line is drawn to indicate a two-part piecewise fit. We propose that this model may be used to explain the path of a singleton gene evolving into a large, conserved OLS: initially, there is an exploratory phase (left blue line), where rapid formation of OLSs and dissolution of OLSs may occur until a stable OLS is formed, after which a long phase of tuning and refinement may follow (right blue line).

To evaluate the processes involved in the “birth” and “death” of OLSs in the haloarchaea, we calculated “OLS trajectories” for analogous OLSs in the haloarchaeal genomes and assessed each trajectory for the properties of clustericity, novelty, and evolutionary rate (see Methods for definitions). We found there to be abundant variability in clustericity, novelty, and evolutionary rate among individual OLS trajectories. Despite the variability among individual trajectories, we sought to discover if there were any trends present among the whole set of trajectories, as such trends could potentially indicate an underlying biological principle associated with OLS evolution in the haloarchaea. Comparing clustericity and variety for each trajectory, we discovered that only three of four possible qualitative cases (high clustericity/low variety; low clustericity/high variety; low clustericity/low variety) were represented among the trajectories, while the case of high clustericity/high variety was observed only rarely (Figure 4). 

In other words, the trajectories that displayed high variety in gene content typically also displayed a large number of singleton genes, implying that variety is typically achieved through the passage of a singleton gene state, and this singleton gene state is often more persistent than transient, evolutionarily neutral OLSs. This is consistent with the notion of rapid OLS formation and dissolution under a model of neutral selection (as suggested by Price [15]): a singleton gene may “explore” the repertoire of available genes, joining and unjoining OLSs freely, moving back to a singleton gene state before joining/forming a new OLS. If a singleton gene is to become part of a highly conserved OLS, we may imagine that following an exploratory phase of frequent joining and unjoining, the gene may finally join/form an OLS that can become progressively more stable through a period of refinement. We have illustrated this model as a two-part piecewise linear fit of our set of haloarchaeal trajectories (Figure 4).

We found that in the haloarchaea, OLSs are frequently born by the joining of two singleton genes to form a two-gene OLS (data not shown). However, a singleton gene may merge with an existing OLS, resulting in a new OLS topology, which may be thought of as either an OLS modification event (from the perspective of the existing OLS) or an OLS “birth” event (from the perspective of the singleton gene joining the OLS). Unlike OLS formation by the merging of two singleton genes, when a singleton gene is added to an existing OLS, there are three locations (relative to the existing OLS) where the singleton gene can be added: (1) prepended to the head of the existing OLS, (2) appended to the tail of the existing OLS, or (3) inserted within an existing OLS. Out of the 4005 identified haloarchaeal OLS trajectories, 2217 (55.4%) did not contain any examples of a gene singleton OLS modification events, 946 (23.6%) contained a clear example of one type of modification event, and 842 (21.0%) featured examples of multiple single-gene modification events occurring within a single context trajectory. Among the 946 clear examples of a single type of modification event, we discovered 432 prepending events (45.6%), 428 appending events (45.2%), and 86 insertion events (9.1%) (Table S7). We found that the ratio of prepending and appending events to insertion events corresponds well with an analysis of *E. coli* OLSs [15]. The observation that OLS gene content modification tends to occur equally by addition or loss of a single gene to the head or tail suggests that the mechanism(s) underlying single-gene modifications may not discriminate between prepending and appending. The low frequency of gene insertion or deletion events is not surprising given that the mechanisms we know of to mediate gene shuffling events (i.e., action of transposons, recombination) may not be sufficiently precise to disrupt existing OLS function.

#### 3.1.2. OLS Evolution is Distinct from Whole-Species Evolution in the Haloarchaea

Species phylogeny is traditionally estimated by fitting a sequence divergence-based mathematical model to an alignment made of one or more highly conserved marker genes [39]. Previous investigations have suggested that organismal phylogeny largely agrees with similarity in OLS structure [18,40,41], though a few examples of disagreement had been identified, and attributed to horizontal gene transfer [18]. However, the relatively large average phylogenetic distance between organisms in those sample sets may have biased the investigations towards evolutionarily stable OLSs and thus missed some of the underlying dynamics of OLS formation and dispersion. Using a large set of more closely related organisms, we sought to test if we could observe differences between OLS topology and species-level phylogeny for particular OLSs, as a means to explore the evolutionary trajectories of individual OLSs. We compared each context trajectory to an organismal phylogeny (Figure S8) and found that groups of OLSs clustered by identical gene content disagreed with groupings based on organismal phylogeny frequently (in 3705/4005, or 92.5% of cases). Removing all singletons from OLS trajectories and repeating the comparison, we identified 849/4005 (21.2%) disagreement between OLS structure and phylogeny. Thus, though the rapid formation and dissolution of OLSs by singleton genes accounted for the majority of cases (2856/3705, or 77.1%, of the disagreements), a significant number of disagreements were recorded excluding these cases (approximately one-fifth of all trajectories). This suggests that the disagreements between species phylogeny and OLS topology can occur frequently over shorter evolutionary time scales. Our results indicate that the evolutionary forces resulting in the shaping of OLS topology are distinct from the forces causative of sequence divergence, and that a sequence divergence model of evolution is insufficient to accurately predict changes in OLS topology within groups of organisms separated by shorter phylogenetic distances than previously analyzed.

### 3.2. Quantitative Evaluation of OLS Modification

Aside for changes in gene content, OLS topology may vary in other ways: these include changes in gene order, the gain and loss of promoter sites, and changes in intergenic gap size [29,30,31,32]. We assessed the haloarchaeal OLSs for a variety of topological changes, quantitatively evaluated the degree at which these changes were observed, and identified several interesting examples of OLSs exhibiting these changes. 

#### 3.2.1. Gene Re-Ordering Events are Rare Phenomena

**Figure 5:**
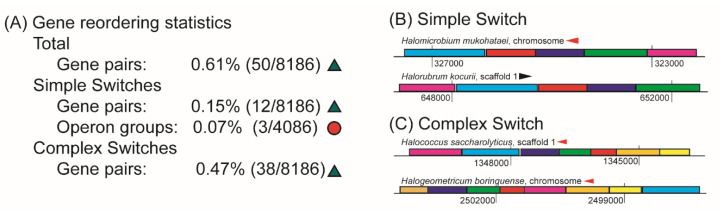
Modification by gene reordering. (**A**) Frequencies of gene reordering observed among adjacent gene pairs (green triangle) and identical content groups (red circle). These could be further segregated into simple switches (gene reordering does not accompany modification in gene content) and complex switches (gene reordering accompanies modification in gene content). Two examples are shown: a simple switch (**B**) and a complex switch (**C**). In (**B**), light blue, red, dark blue, green, and pink genes are a glycerol-3-phosphate ABC transporter, ugpA, ugpE, ugpC, and glycerophospohryl diester phosphodiesterase, respectively. In (**C**), pink, light blue, dark blue, green, red, tan, yellow, and mocha genes are dimethylbenzimidazole phosphoribosyltransferase, cobyrinic acid A,C-diamide synthase, adenosylcobinamide-phosphate synthase, cobalamin synthase, CobY, L-threonine 3-O-phosphate decarboxylase, adenosylcobinamide amidohydrolase and a “conserved cobalamin OLS protein”, respectively.

The benefits associated with gene re-ordering may be significant: order has been shown to be important for both expression level [20], and temporal coordination [22] of genes in OLSs. Examinations of changing OLS structures in bacteria have asserted that reordering occurs frequently [17]. However, from our analyses, we ascertained that variation in gene order in haloarchaeal OLSs is a rare phenomenon (Figure 5).

We found that only approximately 50/8136 adjacent gene pairs (approximately 0.6%) demonstrated a change in gene order. These cases were manually investigated, and subclassified into two categories: simple switches (the gene content between OLSs does not change, and OLSs change in gene order), and complex switches (gene reordering is combined with OLS “birth”, “death”, or gene content modification events). In total, 12 gene rearrangements were classified as simple and 38 as complex (Table S9). Investigation of the 12 adjacent gene pairs that underwent simple switches revealed that these mapped to 3 identical content groups, indicating that gene reordering is even more rare if we require that OLSs may not vary in gene content (0.07%) (Figure 5A).

Mathematical models have predicted [23] and experimental work confirmed [20] that the impact on gene order on metabolic pathway productivity is most pronounced when the position of genes located distantly within an OLS is interchanged. In the OLS pictured in Figure 5B, the pink gene (glycerophosphoryl diester phosphodiesterase, EC 3.1.4.46) is located in tail position of the OLS in *Halomicrobium mukohataei* as compared to the head position in *Halorubrum kocurri*. According to Lim et al. [20], we would expect there to be a dramatic difference in the ratio of the expression level of the pink gene to the other genes in the OLS in each of these two species (under comparable conditions). A previous finding asserted that in bacteria, genes within OLSs that encode interacting protein subunits do not undergo re-ordering [21]. This seems to hold true in the haloarchaea as well: for example, in an OLS associated with phosphate and carbon starvation (Figure 5B), the subunits ugpA, ugpE and ugpC (red, dark blue, and green genes, Figure 5B), which together form a transmembrane transporter, did not reorder, while the genes encoding functionally but not structurally associated proteins (glycerol-3-phosphate ABC transporter and glycerophosphoryl diester phosphodiesterase) exchanged positions between the head and tail of the OLS. We identified a complicated reordering event (Figure 5C) where all OLS genes encode for different enzymes in a cobalamin biosynthesis pathway (with no interacting protein subunits).

#### 3.2.2. Promoter Modification Occurs More Often at the Head of the OLS than at Internal Gene Start Sites

**Figure 6:**
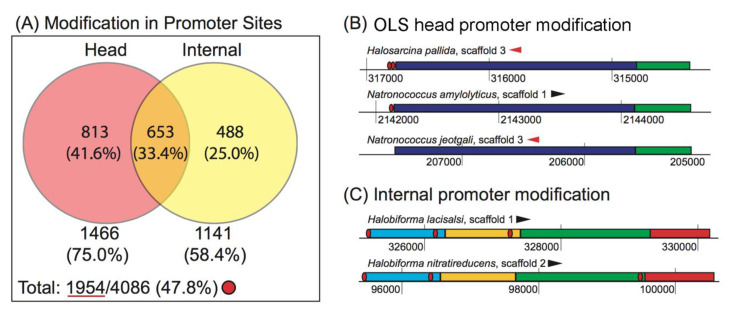
OLS modification by promoter. (**A**) Observed modifications in promoter sites. A total of 1954/4086 identical content groups (red circle) exhibit a modification in promoter content. Content groups that exhibit a modification (1954, red underline), were assessed for the location of the modification, if it occurred at the head of the OLS, an internal site, or both (Venn diagram). Two examples are shown: (**B**) a modification at the OLS head only and (**C**) modification at internal site(s) only. In (**B**), the blue gene is 3-hydroxyacyl-CoA dehydrogenase, green gene is a monoamine oxidase regulatory protein. In (**C**), blue, yellow, green, and red genes are a spemidine-binding ABC transporter, PotA, PotB, and aliphatic amidase, respectively.

Promoter modification is a highly prevalent OLS modification strategy in the haloarchaea: Nearly half (1954/4086, or 47.8%) of the OLSs in each identical gene content group demonstrated one or more changes in their promoter content. We further subclassified these OLSs according to promoter modification events occurring at the head of an OLS and promoter modifications at internal gene start sites, as these two types of events signify biologically distinct phenomena: the appearance or disappearance of a promoter at an OLS head is representative of gain or loss of a promoter, which may affect the strength of expression [42] and noisiness [43] of the entire OLS, while the appearance or disappearance of internal promoters is related to differential expression of a subset of genes in the OLS under a particular set of conditions [25]. Significant attention has been paid in the literature to programs of sub-OLS gene expression [24,25,44]. However, less has been paid to whole OLS tuning, especially how this may change across species. We found that among the haloarchaea, cross-species OLS evolution more often occurred in altering the machinery associated with tuning whole OLS units by modifications at their head (1466/1954, or 75.0%) than in condition-dependent gene subsets of the OLS by modifications at internal sites (1141/1954, or 58.4%) (Figure 6A).

Though more modifications were identified at the OLS head than to internal sites, the observation that modifications to internal sites occurred in almost 60% of cases suggests that modification of internal promoter content is also an important strategy for OLS evolution in the haloarchaea. Curation of the dataset suggests that more complicated promoter modification events also occur: for example, we found cases where the number of predicted promoter sites at the head of an OLS varied (which may be representative of the gain or loss of an alternative start site, Figure 6B), and we identified cases where multiple, distinct internal promoter modification events have occurred (Figure 6C, both the green and red genes have undergone a promoter gain/loss event). These conclusions could ultimately be empirically tested through numerous cross-species transcriptomic studies.

#### 3.2.3. OLS Genes Undergo Frequent Changes in Intergenic Gap Size

**Figure 7:**
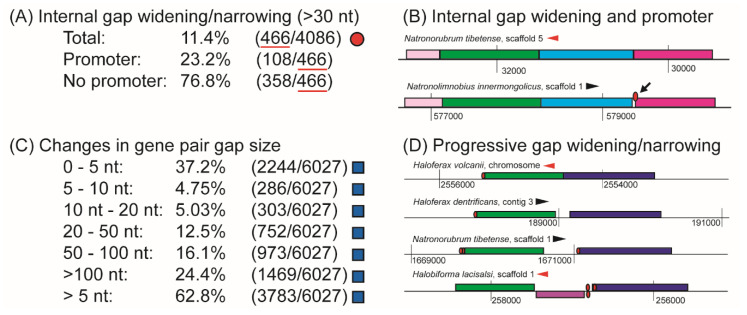
Modification by change in intergenic gap size. Intergenic gap sizes were assessed using two different query sets, based on identical content groups (**A**, red circle), and co-occurring gene pairs (**C**, blue square). Requiring that OLSs not vary in gene content, we determined that approximately 11% of these OLSs exhibited a difference in intergenic gap size of component genes of 30 nt or more (**A**). Of these (466, red underline), the appearance of an internal promoter coincided with the gap-widening event in approximately 23% (108/466) cases. (**B**) An example of such a combined internal gap widening and promoter appearance, where a pair of genes (blue and dark pink genes) transition from an intergenic gap size of 0 nt in *Natronorubrum tibetense* to an intergenic distance separation of 32 nucleotides in *Natronolimnobius innermongolicus* (black arrow indicates gap, promoter is shown elevated above intergenic gap for clarity). Light pink, green, blue and dark pink genes are acetylglutamate kinase, acetylornithine aminotransferase, acetylornithine deacetylase and ornithine carbamoyltransferase, respectively. Assessing only gene pairs that were identified to be collinear in an OLS in at least one organism, we found that changes in intergenic distance varied frequently (**C**). Certain cases appeared that may represent progressive deletion (or addition) of intervening sequence such as the example shown in (**D**), where the intergenic spacing between green (inosose isomerase) and dark blue genes (myo-inositol 2-dehydrogenase) increases progressively from *Haloferax volcanii* (3 nt), to to *Haloferax denitrificans* (174 nt), to *Natronorubrum tibetense* (442 nt), finally to *Halobiforma lacisalsi*., where a gene in the opposite orientation appears. To interrogate this example further, we identified all examples where the genes with homology clusters 866 and 1119 were collinear and nearby, extracted intergenic gaps separating these genes, and performed a series of multiple alignments using the online ClustalW web tool [45], with default parameters. The results of multiple alignments of these intergenic can be found in supplementary information (File S10).

Intergenic gap size is an important aspect of OLS structure: a study of transcriptional units in *E. coli* and *B. subtilis* found a strong correlation between intergenic gap size and whether genes were always, sometimes, or never co-expressed in an OLS over a range of physiologically relevant conditions [24]. The msagnitude of the intergenic gap size change is intimately linked to functional consequences: for example, a very large widening in gap size must be associated with OLS “death”, and a negative intergenic gap size ensures that two genes are always co-expressed (they have overlapping transcripts). How often do OLSs experience major changes in intergenic gap size, but do not vary in their gene content? We chose to use a distance threshold of 30 nt for two reasons: (1) we sought to discover cases where two genes within an OLS must be tightly packed in one species and much more loosely packed in another but still within the 50 nt criterion associated with OLS prediction (2) a minimum length of 30 nt is required to fit a canonical BRE-TATA-RNAP promoter [30] which might be required to create a novel regulatory site. We identified 466 of 4086 (11.4%) that demonstrated a significant internal gap widening without any changes in gene content, and of these, approximately one-quarter (108 of 466, or 23.2%) demonstrated the appearance of an internal promoter in the widened gap (Figure 7A). These 108 examples we have identified are especially likely to represent OLSs gaining the ability to express a subset of their genes under different conditions (one of which is shown in Figure 7B).

Intergenic gap widening may occur simultaneously as OLSs modify their gene content. We therefore expanded our investigations beyond identical content group OLSs, assessing intergenic gap widening events by analyzing a list of co-occurring gene pairs (see Methods). More than half (62.8%) of these co-occurring gene pairs demonstrate a change in intergenic gap size greater than 5 nt, making it the most prevalent mode of OLS evolution we examined. We found that approximately one-quarter of (24.4%) of cases of intergenic widening involved a large separation (more than 100 nucleotides), highly probably examples of OLS “death”. Nearly as many cases (22.2%) demonstrated changes of between 5 and 50 nt, examples of conditional modification. According to our criterion for OLS prediction, separations of between 50 and 100 nt (16.1% of cases) represent examples of OLS “death”. However, because our choice of 50 nt represents a conservative OLS prediction threshold, it remains possible that some of these cases represent examples of OLS modification as opposed to OLS “death” (Figure 7C).

We identified an example (Figure 7D) suggestive of progressive insertion or deletion of intergenic content between two genes. Based on their annotations (inosine isomerase and myo-inositol 2-dehydrogenase), these two genes could be functionally linked (both are enzymes involved inositol phosphate metabolism) which might provide a pressure to form OLSs. A series of multiple alignments comparing the intergenic sequence between these two genes across all the halophiles revealed good agreement for progressive deletion within a genus, with the interpretation being more complicated when comparing across genera (File S10). It may be the case that multiple independent evolutionary histories dictate deletion/addition, and these histories involve deletion or addition of different sequences, or that a large amount of sequence mutation has made interpreting the true history of insertion or deletion events undetectable. That an operon could form by progressive sequence deletion was proposed originally in 1996 [13]. However, to the best of our knowledge no evidence has ever been presented suggesting that this occurs in nature. This example, therefore, might represent such a progressive deletion (or conversely, “death” by progressively gene insertion) occurring in nature. The existence of promoters associated with genes with higher intergenic gap sizes could indicate a complete lack of co-transcription, while the disappearance of the promoter in myo-inositol 2-dehydrogenase in smaller intergenic gap sizes might indicate the intergenic spacing at which co-transcription under some set of conditions could occur. Note the last genomic segment pictured in Figure 7D, where a gene on the opposite strand lies between the inosine isomerase and myo-inositol 2-dehydrogenase genes. This case may exemplify the phenomenon where the path to OLS formation is obstructed by random gene insertion events. Alternatively, these two genes may not always be under strong pressure to form OLSs in all haloarchaeal organisms examined.

#### 3.2.4. Estimating Relative Rates of Evolution

In our analysis of the set of OLSs that did not vary in gene content, we discovered that 3 demonstrated a change in gene order, 466 a significant change in intergenic distance, and 1954 a change in promoter content (Figure 5, Figure 6 and Figure 7). Based on the differential frequency that these modification events occur, it stands that these three phenomena may occur on different evolutionary time scales. Using ratios of observed frequencies as a proxy for rate, we estimated the relative rates of evolution of these three modes of OLS modification (Table 1).

While our quantitative estimates may be subject to error resulting from unseen dependencies between these forces, as well as fundamental assumptions upon which we have based our analysis (specifically, computationally predicted gene calls, homology groups, OLS predictions, and promoter predictions), our qualitative assessment of the relative rates is unlikely to change.

### 3.3. Construction of an OLS Forest

A “context tree” may be constructed by comparing the gene content of every OLS to every other OLS within a context trajectory and amalgamating these comparisons using agglomerative hierarchical clustering. OLS trees therefore organize species-specific OLS topologies into hierarchical diagrams, and provide an OLS-topology based mapping of the relatedness of organisms. However, as we determined previously, haloarchaeal OLS topologies frequently deviate from a sequence divergence-based organismal phylogeny. What might it mean if two or more OLS trees are very similar to one another? To a first approximation, this might indicate that the OLSs represented in these trajectories have felt and responded to a similar set of evolutionary pressures within the set of organisms examined. This similarity implies a connection between these trajectories that need not have an obvious functional interpretation, but would still be biologically meaningful. A convenient framework to search for such cases of high similarity between OLS trees is the context forest approach we have developed (Figure 2). We have applied this approach to the subset of OLS trajectories that occur in each organism at least once, and have a clustericity of 50% or higher. The complete OLS forest is available in (Figure S11).

#### 3.3.1. Large OLSs Reveal Themselves as Context Forest “Groves”

When two genes always co-occur in the same OLS, OLS trajectories drawn from either gene will yield identical OLS trees. However, if genes only co-occur in OLSs some of the time, each OLS tree will differ for cases where the genes are not located in the same OLS. As OLSs become larger and more varied across species, it becomes more challenging to represent a unified “OLS” from a set of partially overlapping OLS trajectories. A number of approaches have been developed to define such a unified OLS (or, more generally, any collinear collection of genes) from a set of partially overlapping, related gene instances present across a set of species [5,46,47]. However, existing approaches all involve defining specific numerical thresholds, which fail to account for the different degrees at which OLS instances might overlap in their gene content. Intuitively, an OLS that remains completely conserved in gene content in all species examined is quite different from one that only remains partially conserved—the difference relating to how tightly conserved an OLS need be to accommodate for the evolutionary pressures it feels. It may be useful, therefore, to attempt to determine all such cases in a non-parametric way, and assign appropriate parameter values after all such instances have been determined.

**Figure 8:**
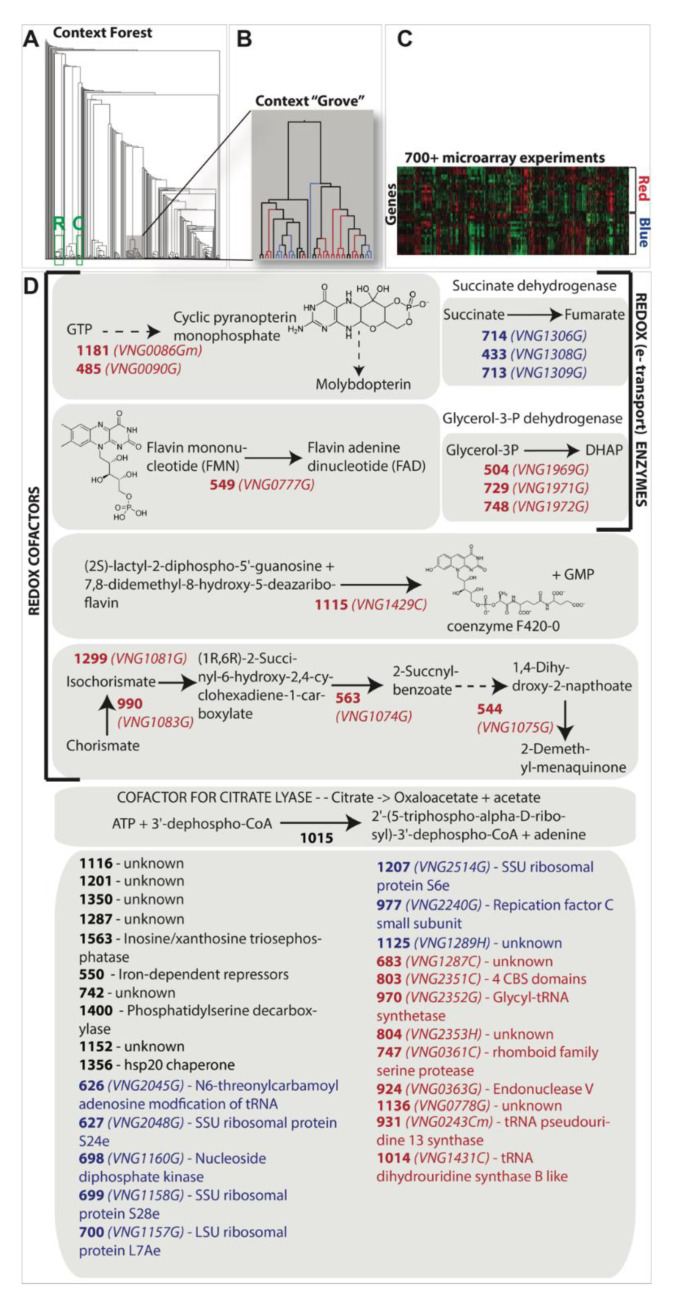
An in-depth analysis of one context grove generated from an analysis of OLS in the haloarchaea. (**A**) The tree at the top left shows the resulting context forest. A green box labeled “R” represents a ribosomal gene grove while the green box labeled “C” represents a cobalamin operon grove. (**B**) The tree immediately to the right of the context forest is an enlarged view of one context grove. (**C**) The heat map (top right) shows the patterns of transcriptional abundance for the genes found in this specific grove that are encoded in *Halobacterium salinarum* NRC-1. The expression data are derived from publicly available microarray datasets [48,49,50,51,52,53,54,55]. The data have been clustered and are found to be most parsimoniously described by two clusters of expression termed here Red and Blue. (**D**) When a gene present in the grove of panel B was also found in *H. salinarum NRC-1,* its expression cluster membership (panel C) is mapped on the branches and leaves of the context grove and in the cluster/gene names in the figure below by coloring the branch and/or gene name either Red or Blue. Gene names and annotations are labeled with cluster number in bold and *H. salinarum NRC-1* geneID in italic. Cluster numbers colored black and lacking a geneID were not found in the *H. salinarum NRC-1* genome.

A natural output of the context forest approach is the generation of “groves”—collections of OLS trees that appear visibly separate from the rest of the context forest by a qualitatively long branch separating the cluster from the rest of the tree, leading to a highly similar set of leaves (Figure 8). In many cases, the individual leaves represent the gene components of a large OLS, and the long branch length indicates that other genes rarely or never combine with the genes located in the set of OLS trees within the grove. Comparing the distances between individual leaves within a grove provides a quantitative measure for internal content dissimilarity among OLS instances, and the length of the branch connecting the grove to the rest of the forest describes the distance between this collection of OLS trees and the nearest OLS tree or collection of OLS trees. Comparing examples of large OLSs represented in these groves (Figure S11) reveals variety both in terms of the internal similarity of individual OLS trees and the branch length connecting the grove to the rest of the forest, which helps inform our understanding of the evolution of these OLSs in the haloarchaea.

#### 3.3.2. Context Groves Can Reveal Highly Conserved OLSs (Local Synteny) but also Functionally Associated yet more Spatially Distributed Collections of Genes

We can expect that conservation of function in multisubunit complexes where all components are required for function could drive grove formation in the context forest. We see this to be the case for most of the ABC family transporters and genes in known ribosomal operons (Figure 8A, Figure S11). We can also expect that operons encoding key enzymes in metabolic pathways required for coordinated functions might cluster together in groves and see evidence for this with known cobalamin gene operons, for example (Figure 8A, Figure S11). We can also hypothesize that coordinated regulatory relationships might drive genes into context groves. We see evidence for this as well. The specific example shown in Figure 8 shows suggestive evidence for the conservation of context for putative functional reasons that may be related to the regulatory coordination of distantly spaced genes. The grove described in Figure 8B illustrates a functional theme associated with respiration, particularly anaerobic metabolism, Figure 8D. The grove includes two dehydrogenates (succinate and glycerol-3-phosphate) and genes encoding enzymes in the biosynthesis of key co-factors involved with the dehydrogenases (FAD and menaquinone) and other redox cofactors molydbopterin, coenzyme F420, and a rather unique co-factor for the enzyme citrate lyase. While not perfect, genes in the grove seem to separate relatively well by expression cluster along the two main branches of the context grove (Figure 8B,C). Finally, we note that this grove contains genes from OLS that are spread across the chromosome in *H. salinarum NRC-1* and in all other organisms investigated. Membership in this grove is therefore not an artifact of local synteny.

#### 3.3.3. Hints of Genomic Functional Compartmentalization

**Figure 9:**
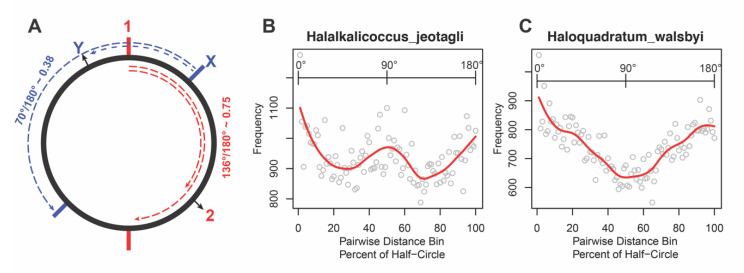
Analysis of relative chromosomal distances for genes in the haloarchaeal OLS context forest. (**A**) A schematic depicting the calculation of chromosomal distance along a relative bisect. The solid circle represents the archaeal chromosome. The relative locations for hypothetical genes 1 and 2 (colored red) are depicted by numbers. Two solid red lines represent points on the chromosome that define the bisect of the chromosome when one end is anchored on the center coordinate of gene 1. One of the dashed red lines represents the distance around half the chromosome starting at the midpoint of gene 1. A second dashed line represents the distance between gene 1 and 2. The fractional angle or distance can be computed as the ratio between the gene-pair distance and the distance around half of the chromosome. A second pair of genes X and Y are labeled blue and depict the calculation of the relative chromosomal distance between these two genes along the bisect defined by starting at the midpoint of gene X. All non-redundant pairwise distances were calculated for genes that were present in the context forest and on the main chromosome. (**B**) The binned distribution of pairwise distances are depicted for genes on the *Halalkalicoccus jeotagli* chromosome. The red line is a smoothed interpolation through the data. (**C**) The binned distribution of pairwise distances are depicted for genes on the *Haloquadratum walsbyi* chromosome. The red line is a smoothed interpolation through the data.

The recent work of Takemata et al. [56] provides evidence of distinct 3D structural domains in the chromosome in at least two *Sulfolobus* species that appear to functionally compartmentalize regions of the chromosome. Takemata et al.’s model suggests that the spatial organization of genes along the chromosome is likely subject to selective pressure and that this can organize functionally related genes (or clusters of genes) at distances along the chromosomal coordinate that are much longer than the distances characterizing OLS. If a similar organizational mechanism is used in the haloarchaea, depending on the complexity of the organization, we would expect to see some preferential spatial organization among the highly conserved genes in the chromosomes of the haloarchaeal genomes. To examine this phenomenon, we explored the context forest to determine whether we could see any evidence of long-range structural organization of the putatively-orthologous genes. For 11 organisms in our dataset whose genome sequences were completely closed when we started our study (*Halalkalicoccus jeotagli, Haloarcula marismortui, Halobacterium salinarum NRC-1, Haloferax volcanii, Halogeometricum borniquense, Halomicrobium mukohataei, Haloquadratum walsbyi, Halorhabdus utahensis, Haloterrigena turkmenica, Natrialba magadii,* and *Natronomonas pharaonis*) we examined the pairwise genomic position about a relative chromosomal bisect for pairs of genes that are present in the context forest and that also co-occur on the main chromosome of each organism (Figure 9A). While few obvious spatial patterns emerged in the analysis of 9 of the 11 chromosomes (Figure S12), we did observe bias in the pattern of pairwise distances in the genomes of *Halalkalicoccus jeotagli* and *Haloquadratum walsbyi*. Figure 9B,C show the frequency of pairwise distances relative to half the distance around the circular chromosome for *Halalkalicoccus jeotagli* and *Haloquadratum walsbyi*, respectively. In *Halalkalicoccus jeotagli* we observe clustering at short distances (likely local structure conservation like that found in OLSs), a peak of conserved distances approximately half the distance about the half circle (~90° apart), and another tendency to organize the pairwise distances of these genes across from one another in the chromosome (~180° apart). In the chromosome of *Haloquadratum walsbyi*, we observe a similar clustering of genes at small distances and a second peak at 180°. While neither of these observations indicates that these genomes are functionally compartmentalized like those of the two *Sulfolobus* species described by Takemata et al. [56], these patterns are nevertheless consistent with this behavior.

## 4. Discussion

Comparative evolutionary genomics studies typically focus either at short length scales (i.e., the nucleotide level), where changes arise largely through errors in genomic replication [57,58,59], or at the length scale where major duplications and rearrangements occur [60]. However, genome evolution occurs simultaneously at different length scales [57,58,59,60]. We expect therefore that in many sets of organisms, such as the 76 haloarchaeal species we investigate here, evolutionarily meaningful structural changes may be observed at an intermediate genomic level, such as to changes in OLS structure [15]. In this study, we have investigated the phenomena of OLS “birth”, “death”, and content modification, gene re-ordering events, promoter modification (both at the OLS head and at internal gene start sites) and changes in intergenic gap size, topological features that have all been shown to be directly related to OLS expression [15,20,23,24,25,40]. We found that though all of these phenomena occur and are important for haloarchaeal OLS evolution, they have occurred with differential frequency: our investigation has allowed us to estimate relative rates of evolution for various types of OLS modification (Table 1). Additionally, our work suggests that while sequence divergence models of phylogeny may be useful in many contexts, OLS structures frequently evolve according to a scheme not perfectly represented by a sequence divergence model of phylogeny (Results, Figure S8), highlighting the value of considering topological features to understand and classify changing OLS structures (as we have done here). A similar observation has been made regarding the diversity of expression the *Lac* operon in closely related strains of *E. coli* [61].

The observations that gene reordering and major gap changes occur less frequently than promoter appearance or disappearance and that smaller (5 nt or more) changes in intergenic gap size actually occur more frequently than promoter appearance or disappearance (Figure 6 and Figure 7) may be considered some of Nature’s “design principles” for building complex biological systems. In addition to adapting their regulatory systems to deal with selective pressures imposed by the environment, these systems must also adapt to the gain and loss of genes (regardless of mechanism) and evolve to “fine tune” the coordinated activities of different cellular functions. It is reasonable, therefore, to expect that genomic context, including their regulatory elements in OLS, would be subject to selection not only for tuning the function of the OLS itself but also to tune its behavior with that of other functionally-associated OLS structures spread throughout the genome. While preliminary, we suggest that some of the clustering of functionally associated, though spatially distributed, genes into context forest groves provides some suggestive evidence of this phenomenon. Practically, in the context of synthetic biology, it may be possible to consider the evolution of genomic context as a design target for both local optimization of function (e.g., within OLS) and even for optimizing the coordination of functional units defined across multiple OLS.

Finally, we note that OLSs are likely only one example of contextual organization within genomes (both microbial and eukaryotic) subject to selection and important to understand. The approach we have developed and presented herein is readily applicable to other datasets and easily extensible to the analysis of non-OLS context sets [62]. The tools are accessible in the open-source tool, JContextExplorer (File S13, S14).

## Figures and Tables

**Table 1:** Relative rates of evolution of these three modes of OLS modification.

	Gene Reordering	30nt Gap Widening	Promoter Modification
Gene Reordering	1	0.0064	0.0015
30nt Gap Widening	155.33	1	0.24
Promoter Modification	651.33	4.19	1

## Supplementary Materials

The following are available online at https://www.mdpi.com/2076-2607/8/12/1900/s1, Table S1: Genomes_and_NCBI_Accession_Numbers, Table S2: OperonTrajectoriesAllComparisons, File S3: UsersManual_v4, Table S4: Co-occurringGenePairsQuerySet, Table S5: IdenticalContentGroupsQuerySet, Table S6: AdjacentGenePairsQuerySet, Table S7: OperonModificationExamples, Figure S8: DisagreementWithPhylogeny, Table S9: Table_GeneOrderChanges, File S10: Clust_866_1119_Seq_and_Align, Figure S11: OperonForest, Figure S12: Cluster_distance_conservation, File S13: JCE_Pipeline_Instructions_v3, and File S14: JCE_HC_PIPELINE. JContextExplorer and the associated pipeline can be accessed at GitHub (https://github.com/PMSeitzer/JContextExplorer/releases/tag/3.0.0 or https://facciotti.bme.ucdavis.edu/resources_data/software/).

## References

[1] Bergman N.H., Passalacqua K.D., Hanna P.C., Qin Z.S. (2007). Operon prediction for sequenced bacterial genomes without experimental information. Appl. Environ. Microbiol..

[2] Price M.N., Huang K.H., Alm E.J., Arkin A.P. (2005). A novel method for accurate operon predictions in all sequenced prokaryotes. Nucleic Acids Res..

[3] Hillier L.W., Miller R.D., Baird S.E., Chinwalla A., Fulton L.A., Koboldt D.C., Waterston R.H. (2007). Comparison of C. elegans and C. briggsae genome sequences reveals extensive conservation of chromosome organization and synteny. PLoS Biol..

[4] Cutter A.D., Agrawal A.F. (2010). The evolutionary dynamics of operon distributions in Eukaryote genomes. Genetics.

[5] Rogozin I.B. (2002). Connected gene neighborhoods in prokaryotic genomes. Nucleic Acids Res..

[6] De Daruvar A., Collado-Vides J., Valencia A. (2002). Analysis of the cellular functions of Escherichia coli operons and their conservation in Bacillus subtilis. J. Mol. Evol..

[7] Wolf Y.I., Rogozin I.B., Kondrashov A.S., Koonin E.V. (2001). Genome alignment, evolution of prokaryotic genome organization, and prediction of gene function using genomic context. Genome Res..

[8] Overbeek R., Fonstein M., D’Souza M., Pusch G.D., Maltsev N. (1999). The use of gene clusters to infer functional coupling. Proc. Natl. Acad. Sci. USA.

[9] Price M.N., Huang K.H., Arkin A.P., Alm E.J. (2005). Operon formation is driven by co-regulation and not by horizontal gene transfer. Genome Res..

[10] Fisher R.A. (1930). The Genetical Theory of Natural Selection.

[11] Fang G., Rocha E.P., Danchin A. (2008). Persistence drives gene clustering in bacterial genomes. BMC Genom..

[12] Jacob F., Monod J. (1961). On the regulation of gene activity. Cold Spring Harb. Symp. Quant. Biol..

[13] Lawrence J.G. (1996). Selfish operons: Horizontal transfer may drive the evolution of gene clusters. Genetics.

[14] Yin Y., Zhang H., Olman V., Xu Y. (2010). Genomic arrangement of bacterial operons is constrained by biological pathways encoded in the genome. Proc. Natl. Acad. Sci. USA.

[15] Price M.N., Arkin A.P., Alm E.J. (2006). The life-cycle of operons. PLoS Genet..

[16] Memon D., Singh A.K., Pakrasi H.B., Wangikar P.P. (2013). A global analysis of adaptive evolution of operons in cyanobacteria. Antonie Van Leeuwenhoek.

[17] Itoh T., Takemoto K., Mori H., Gojobori T. (1999). Evolutionary instability of operon structures disclosed by sequence comparisons of complete microbial genomes. Mol. Biol. Evol..

[18] Fani R., Brilli M., Lio P. (2005). The origin and evolution of operons: The piecewise building of the proteobacterial histidine operon. J. Mol. Evol..

[19] Cao H., Ma Q., Chen X., Xu Y. (2019). DOOR: A prokaryotic operon database for genome analyses and functional inference. Brief. Bioinform..

[20] Lim H.N., Lee Y., Hussein R. (2011). Fundamental relationship between operon organization and gene expression. Proc. Natl. Acad. Sci. USA.

[21] Dandekar T., Snel B., Huynen M., Bork P. (1998). Conservation of gene order: A fingerprint of proteins that physically interact. Trends Biochem. Sci..

[22] Zaslaver A., Mayo A., Ronen M., Alon U. (2006). Optimal gene partition into operons correlates with gene functional order. Phys. Biol..

[23] Kovács K., Hurst L.D., Papp B. (2009). Stochasticity in protein levels drives colinearity of gene order in metabolic operons of Escherichia coli. PLoS Biol..

[24] Okuda S., Kawashima S., Kobayashi K., Ogasawara N., Kanehisa M., Goto S. (2007). Characterization of relationships between transcriptional units and operon structures in Bacillus subtilis and Escherichia coli. BMC Genom..

[25] Koide T., Reiss D.J., Bare J.C., Pang W.L., Facciotti M.T., Schmid A.K., Pan M., Marzolf B., Van P.T., Lo F.-Y. (2009). Prevalence of transcription promoters within archaeal operons and coding sequences. Mol. Syst. Biol..

[26] Becker E.A., Seitzer P.M., Tritt A., Larsen D., Krusor M., Yao A.I., Wu D., Madern D., Eisen J.A., Darling A.E. (2014). Phylogenetically driven sequencing of extremely halophilic archaea reveals strategies for static and dynamic osmo-response. PLoS Genet..

[27] Enright A.J., Dongen S.V., Ouzounis C.A. (2002). An efficient algorithm for large-scale detection of protein families. Nucleic Acids Res..

[28] Price M.N., Dehal P.S., Arkin A.P. (2010). FastTree 2–Approximately maximum-likelihood trees for large alignments. PLoS ONE.

[29] Grant C.E., Bailey T.L., Noble W.S. (2011). FIMO: Scanning for occurrences of a given motif. Bioinformatics.

[30] Seitzer P., Wilbanks E.G., Larsen D.J., Facciotti M.T. (2012). A Monte Carlo-based framework enhances the discovery and interpretation of regulatory sequence motifs. BMC Bioinform..

[31] Brouwer R.W.W., Kuipers O.P. (2008). The relative value of operon predictions. Brief. Bioinform..

[32] Bockhorst J., Qiu Y., Glasner J., Liu M., Blattner F., Craven M. (2003). Predicting bacterial transcription units using sequence and expression data. Bioinformatics.

[33] De Hoon M.J.L., Imoto S., Kobayashi K., Ogasawara N., Miyano S. (2003). Proceedings of the Biocomputing 2004.

[34] Edwards M.T., Rison S.C.G., Stoker N.G., Wernisch L. (2005). A universally applicable method of operon map prediction on minimally annotated genomes using conserved genomic context. Nucleic Acids Res..

[35] Baldauf S.L. (2003). Phylogeny for the faint of heart: A tutorial. Trends Genet..

[36] Seitzer P., Huynh T.A., Facciotti M.T. (2013). JContextExplorer: A tree-based approach to facilitate cross-species genomic context comparison. BMC Bioinform..

[37] Fernandez A., Gomez S. (2008). Solving non-uniqueness in agglomerative hierarchical clustering using multidendrograms. J. Classif..

[38] Fowlkes E.B., Mallows C.L. (1983). A method for comparing two hierarchical clusterings. J. Am. Stat. Assoc..

[39] Wiley E.O., Lieberman B.S. (2011). Phylogenetics: The Theory of Phylogenetic Systematics.

[40] Lathe III W.C., Snel B., Bork P. (2000). Gene context conservation of a higher order than operons. Trends Biochem. Sci..

[41] Yoon S.H., Reiss D.J., Bare J.C., Tenenbaum D., Pan M., Slagel J., Moritz R.L., Lim S., Hackett M., Menon A.L. (2011). Parallel evolution of transcriptome architecture during genome reorganization. Genome Res..

[42] Rhodius V.A., Mutalik V.K. (2010). Predicting strength and function for promoters of the Escherichia coli alternative sigma factor, σE. Proc. Natl. Acad. Sci. USA.

[43] Carey L.B., van Dijk D., Sloot P.M.A., Kaandorp J.A., Segal E. (2013). Promoter sequence determines the relationship between expression level and noise. PLoS Biol..

[44] Adhya S. (2003). Suboperonic regulatory signals. Sci. STKE.

[45] Larkin M.A., Blackshields G., Brown N.P., Chenna R., McGettigan P.A., McWilliam H., Valentin F., Wallace I.M., Wilm A., Lopez R. (2007). Clustal W and Clustal X version 2.0. Bioinformatics.

[46] Che D., Li G., Mao F., Wu H., Xu Y. (2006). Detecting uber-operons in prokaryotic genomes. Nucleic Acids Res..

[47] Novichkov P.S., Rodionov D.A., Stavrovskaya E.D., Novichkova E.S., Kazakov A.E., Gelfand M.S., Arkin A.P., Mironov A.A., Dubchak I. (2010). RegPredict: An integrated system for regulon inference in prokaryotes by comparative genomics approach. Nucleic Acids Res..

[48] Bonneau R., Reiss D.J., Shannon P., Facciotti M., Hood L., Baliga N.S., Thorsson V. (2006). The Inferelator: An algorithm for learning parsimonious regulatory networks from systems-biology data sets de novo. Genome Biol..

[49] Whitehead K., Kish A., Pan M., Kaur A., Reiss D.J., King N., Hohmann L., DiRuggiero J., Baliga N.S. (2006). An integrated systems approach for understanding cellular responses to gamma radiation. Mol. Syst. Biol..

[50] Kaur A., Pan M., Meislin M., Facciotti M.T., El-Gewely R., Baliga N.S. (2006). A systems view of haloarchaeal strategies to withstand stress from transition metals. Genome Res..

[51] Facciotti M.T., Reiss D.J., Pan M., Kaur A., Vuthoori M., Bonneau R., Shannon P., Srivastava A., Donohoe S.M., Hood L.E. (2007). General transcription factor specified global gene regulation in archaea. Proc. Natl. Acad. Sci. USA.

[52] Schmid A.K., Reiss D.J., Kaur A., Pan M., King N., Van P.T., Hohmann L., Martin D.B., Baliga N.S. (2007). The anatomy of microbial cell state transitions in response to oxygen. Genome Res..

[53] Bonneau R., Facciotti M.T., Reiss D.J., Schmid A.K., Pan M., Kaur A., Thorsson V., Shannon P., Johnson M.H., Bare J.C. (2007). A predictive model for transcriptional control of physiology in a free living cell. Cell.

[54] Whitehead K., Pan M., Masumura K., Bonneau R., Baliga N.S. (2009). Diurnally entrained anticipatory behavior in archaea. PLoS ONE.

[55] Facciotti M.T., Pang W.L., Lo F., Whitehead K., Koide T., Masumura K., Pan M., Kaur A., Larsen D.J., Reiss D.J. (2010). Large scale physiological readjustment during growth enables rapid, comprehensive and inexpensive systems analysis. BMC Syst. Biol..

[56] Takemata N., Samson R.Y., Bell S.D. (2019). Physical and functional compartmentalization of archaeal chromosomes. Cell.

[57] Jukes T.H., Cantor C.R. (1964). Mammalian Protein Metabolism.

[58] Rutschmann F. (2006). Molecular dating of phylogenetic trees: A brief review of current methods that estimate divergence times. Divers. Distrib..

[59] Levinson G., Gutman G.A. (1987). Slipped-Strand mispairing: A major mechanism for DNA sequence evolution. Mol. Biol. Evol..

[60] Taylor J.S., Raes J. (2004). Duplication and divergence: The evolution of new genes and old ideas. Annu. Rev. Genet..

[61] Phillips K.N., Widmann S., Lai H.-Y., Nguyen J., Ray J.C.J., Balázsi G., Cooper T.F. (2019). Diversity in *lac* operon regulation among diverse *Escherichia coli* isolates depends on the broader genetic background but is not explained by genetic relatedness. mBio.

[62] Seitzer P., Jeanniard A., Ma F., Van Etten J.L., Facciotti M.T., Dunigan D.D. (2018). Gene gangs of the Chloroviruses: Conserved clusters of collinear Monocistronic genes. Viruses.

