# Supplementary material for: The Exploration of Novel Regulatory Relationships Drives Haloarchaeal Operon-Like Structural Dynamics over Short Evolutionary Distances
Source: Microorganisms. 2020 Nov 30;8(12):1900. doi: 10.3390/microorganisms8121900 (PMC7760734; doi:10.3390/microorganisms8121900)
Supplement: Supplementary file 1 [file microorganisms-08-01900-s001.zip › S3_UsersManual_v4.pdf]

JContextExplorer User Manual

Phillip Seitzer

Facciotti Lab
UC Davis

March 10, 2014
Version 4.0

TABLE OF CONTENTS

CHAPTER 1: GETTING STARTED 5
WHAT IS JCONTEXTEXPLORER? 6
WHY SHOULD I USE JCONTEXTEXPLORER? 7
CHAPTER 2: LAUNCHING JCONTEXTEXPLORER 8
WHERE I CAN FIND JCONTEXTEXPLORER? 9
WHAT DO I NEED TO DO BEFORE I CAN LAUNCH JCONTEXTEXPLORER? 9
THE LAUNCH 10
CHAPTER 3: USING JCONTEXTEXPLORER 12
WINDOW LAYOUT 13
MAIN FRAME 14
Genome Set Search Area 15
OR Statement 16
AND Statment 16
IF AND ONLY IF Clause 16
Continuous range of clusters 17
Search Options Area 18
Context Tree Options 20
Internal Frame Management Area 22
Search Results Frame 23
Export Options: Search Results Frame Menu Options 24
Context Tree Frame 26
Context Tree Menu Options 27
Phylogenetic Tree Frame 28
Additional Node Selection Options 29
Search Results Analysis Area 30
Context Viewer Multiple Genome Browser 32
GENOMES MENU 38
New Genome Set 40
Import Genome Set from .gs file 41
Genome Sets 42
Manage Genome Sets 43
Current Genome Set 45
Import Genomes into Current Genome Set 47
From GenBank or .GFF Files 48
Directly from NCBI Databases 50
Import Settings 52

Feature Type Settings 53
GenBank File Options 55
NCBI Database Query Settings 56
Browse NCBI available genomes by organism name 58
Launch NCBI microbial taxonomy browser 59
Retrieve Popular Genome Set 60
Available Sets 61
Load Menu 62
Genome Sequence File(s) 63
Homology Clusters 64
Gene IDs 67
Context Set 68
Available Context Set Types 71
Context Set Filter Types 75
Dissimilarity Measure 76
Amalgamation Types 79
Dissimilarity Factors 80
Included Dissimilarity Types 91
Phylogenetic Tree 95
Sequence Motifs 98
Associating Sequence Motifs with Genomic Features 102
Export Menu 104
Genome Set as .gs file 105
Genomes as Extended GFF files 106
Genomes as GenBank files from NCBI 107
Process Menu 108
Load Query Set 109
Load Data Grouping 111
Data Grouping Correlation 112
Adjusted Fowlkes-Mallows Method 115
Problems associated with non-identical datasets and repeated elements 118
Tree Similarity Scan 120
Comparing Against a Phylogenetic Tree 122
Context Forest 124
Process Output Window 126
Scan Results Panel 127
Context Forest Panel 130
Help Menu 131
CHAPTER 4: ADDITIONAL RESOURCES 132
Video tutorials 133

AUTHOR CONTACT INFORMATION 134

CHAPTER 1: GETTING STARTED

What is JContextExplorer?

JContextExplorer is a tool to facilitate cross-species genomic context comparisons within a set of previously determined annotated genomes (and protein homology clusters). JContextExplorer uses variable-group agglomerative hierarchical clustering to create "context trees", where each leaf represents a single gene neighborhood.

JContextExplorer offers several ways to (1) define genomic groupings (i.e., create the genomic segments to be compared and clustered), (2) perform pairwise comparisons (compare each genomic segment with each other genomic segment to be clustered, and (3) assemble these comparisons into a tree (link the individual dissimilarities between genomic segments using standard clustering approaches). JContextExplorer allows for fast searching a set of annotated genomes, as well as several flexible visualization tools, and allows for direct comparisons with previously computed phylogenetic trees and additional data.

As evident in the name, JContextExplorer is designed to facilitate exploration. Each of the three major steps in context tree creation (genomic grouping definition, pairwise comparisons, tree creation) may be re-computed quickly and easily with alternative parameters. The graphical interface is designed for point-and-click investigation, and provides fast and easy export of major results (context trees, multi-genome context renderings, etc). We strongly suggest using the automated features (tree computation) in concert with the manual interrogation features (multi-genome browser) in your investigations.

WHY SHOULD I USE JCONTEXTEXPLORER?

There are many reasons to use JContextExplorer. Perhaps you would like to

(1) Resolve ambiguities in annotated features, and/or assigning putative functions to un-annotated and under-annotated genes

(2) Compare changes in gene regulatory network structure (as in the case of operons in microbial species).

(3) Discover and interpret potential horizontal gene transfer events.

(4) Within a set of duplicated homologous genes across species, determining which copies are ancestral and which represent more recent expansions.

(5) Peruse annotated features nearby to a gene or genes of interest.

(6) Compare (and count) textual annotations within a set of homology clusters.

(7) Effectively merge one or more context sets into superclusters.

These are but a short list of suggested uses. Any comparative genomic analysis that could benefit by alternative methods of organization and visualization of multiple genomes (or section of multiple genomes) stands to benefit from JContextExplorer. For a few video demonstrations of JContextExplorer in action please see **Chapter 4: Additional resources**.

CHAPTER 2: LAUNCHING JCONTEXTEXPLORER

Where I can find JContextExplorer?

JContextExplorer can be found on the software Facciotti lab website:

http://www.bme.ucdavis.edu/facciotti/resources_data/software/

On this website, JContextExplorer is available both (1) as a Java WebStart and (2) as a downloadable .JAR file. Simply click the Orange Launch button on the page. Supplementary documentation, instructions, and links to video tutorials may also be found on this page. JContextExplorer is distributed as an executable JAR. However, it is also possible to build the tool from source. All source code is available on GitHub:

https://github.com/PMSeitzer/JContextExplorer

What do I need to do before I can launch JContextExplorer?

JContextExplorer runs on the Java Virtual Machine (JVM) version 1.6 or higher. If you do not have the Java runtime environment installed, please install the latest version of Java before attempting to launch JContextExplorer.

The Java Webstart version runs with a maximum heap size of 1024 MB. Please make sure your system can accommodate for this memory allocation. If you are using the WebStart version, to launch JContextExplorer, simply click the orange WebStart launch button.

If you have downloaded the .JAR file directly, you may either (A) double-click on the icon or (B) launch JContextExplorer from the command line with the following command:

```
java -jar <path-to-file>/JContextExplorer.jar
```

You may want to launch java with a larger max heap size to avoid memory-related problems. In that case, type the following command:

```
java -Xmx1024M -jar <path-to-file>/JContextExplorer.jar
```

or

```
java -Xmx2148M -jar <path-to-file>/JContextExplorer.jar
```

THE LAUNCH

The Main Frame of JContextExplorer appears upon launch:

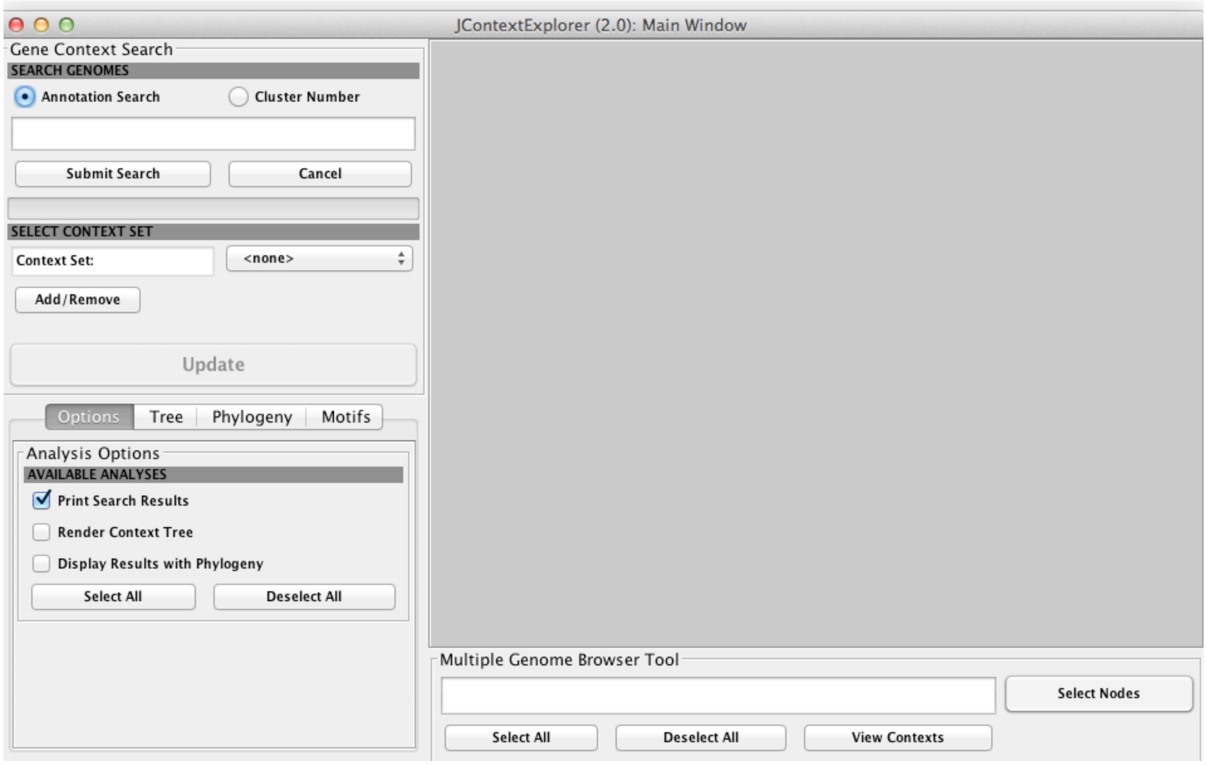

If you are working on a Windows machine, you will notice a menu bar appearing directly above the frame. If you are working on a Macintosh machine, the menu bar will appear at the top of the screen. A menu bar generated from a Macintosh machine looks like this:

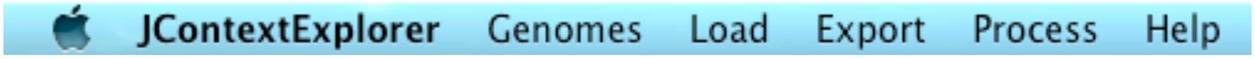

The 5 menus, **Genomes, Load, Export, Process,** and **Help** are unique to JContextExplorer, while the apple symbol and **JContextExplorer** menu are auto-generated by Macintosh. On a windows machine, the apple symbol and JContextExplorer menus will not appear.

What now?

To start, you'll need to create or load a **Genome Set**, which is simply a set of annotated genomes. Once this data has been loaded, you can search this

Genome Set for particular genes, either based on textual annotation (when the **Annotation Search** radio button is selected, in the upper right-hand corner) or homology cluster ID number (when the **Cluster Number** radio button is selected. Searches of your **Genome Set** should be carried out from the search bar in the upper right-hand corner.

Beyond searching the database for instances of a single gene, you'll also want to search for **gene groupings** – that is, instead of retrieving just one gene, you may want to retrieve a set of genes. To do this, you'll need to define a **Context Set**. After retrieving **gene groupings**, you may want to quantitatively compare these groupings to each other – in other words, you'll want to build **Context Trees**. When building such a context tree, you'll want to define an appropriate **dissimilarity measure** and **clustering algorithm** for your context tree. After you've generated context trees, you'll want to browse your contexts using the **Multi-genome browser**.

You may want to load up additional information, customize the dissimilarity metrics, or generate many context trees at once, and compare these context trees to each other. You may also want to interact with NCBI's databases and add, remove, and manage genomes or genome sets to you JContextExplorer section. In other words, there are many things you might want to do, and many things that are possible.

The best way to become familiar with JContextExplorer's features is to watch and the introductory video tutorials, which are described in more detail on page 133. These tutorials will not highlight all of JContextExplorer's features, but will provide a good starting point. **As you watch, complete the steps on you own, pausing the video as needed.** Then, once you've mastered the basics, you may return to this manual and read more about which features you'd like to learn in more detail.

CHAPTER 3: USING JCONTEXTEXPLORER

Window Layout

JContextExplorer is organized as a series of major and minor windows laid out in a semi-hierarchical manner:

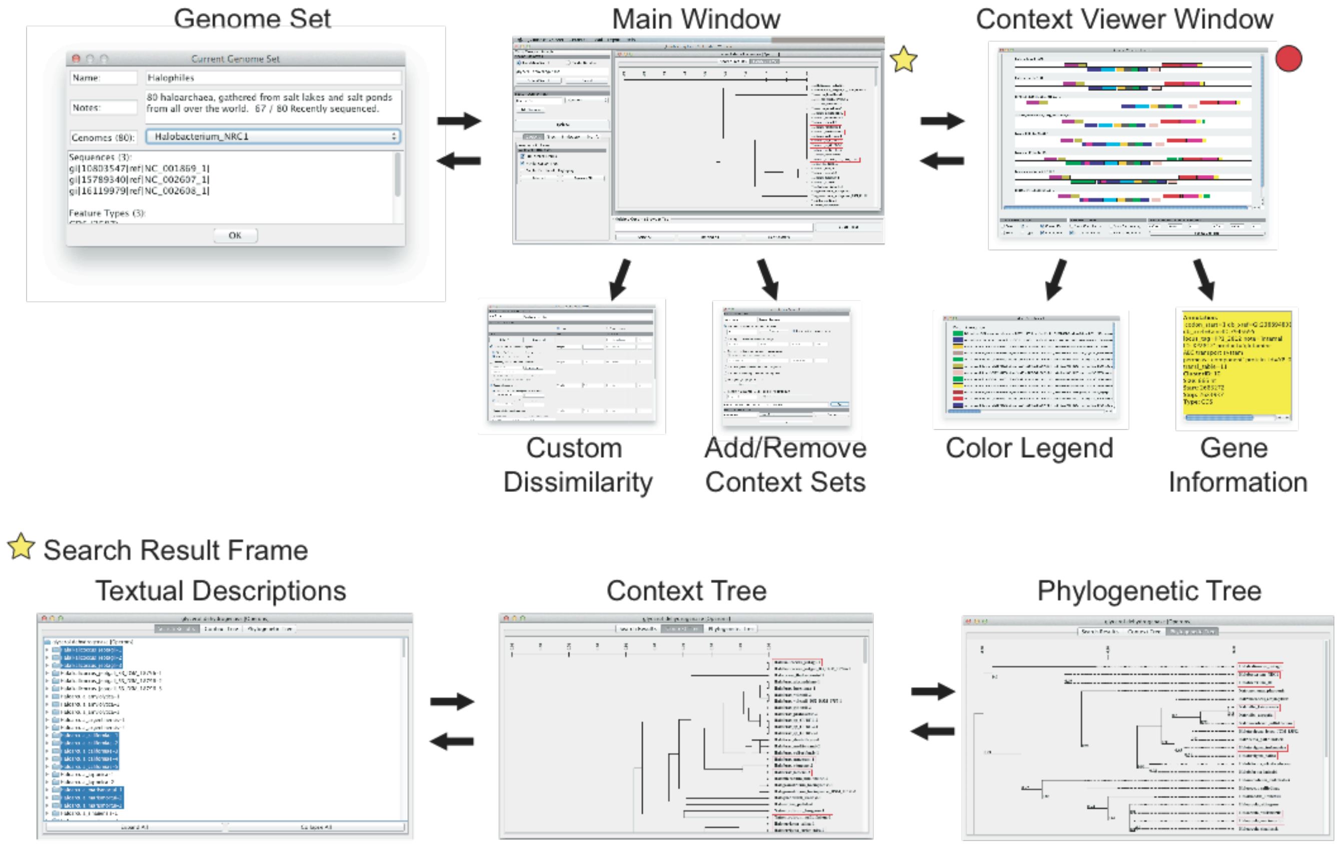

From an initial starting frame, a main window is launched. Within this window, you can do several things (conduct searches, modify search output, load phylogenetic trees, etc), which will often entail launching subordinate "child" windows. JContextExplorer is designed for frequent coordination between the main and child windows.

MAIN FRAME

Conceptually, the Main Frame may be divided into 4 regions:

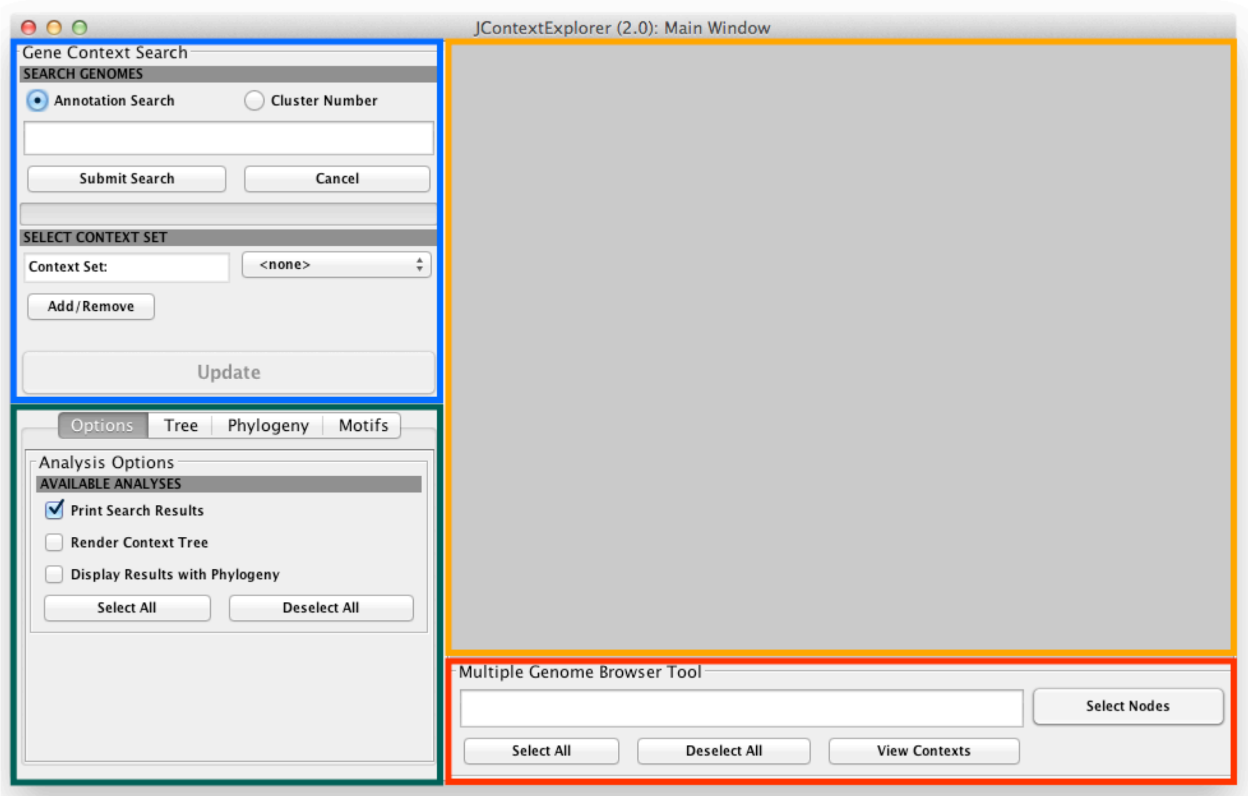

A **Genome Set Search Area** (Blue, upper left), **Search Options Area** (Green, lower left), **Internal Frame Management Area** (Orange, upper right), and a **Search Results Analysis Area** (Red, lower right).

Each of these areas is explained in more depth in the following sections.

Genome Set Search Area

The **Genome Set Search Area** is the place to conduct searches of a loaded **Genome Set** and define, switch, and manage various **Context Sets**. It is located in the upper left-hand corner of the main frame, and looks like this:

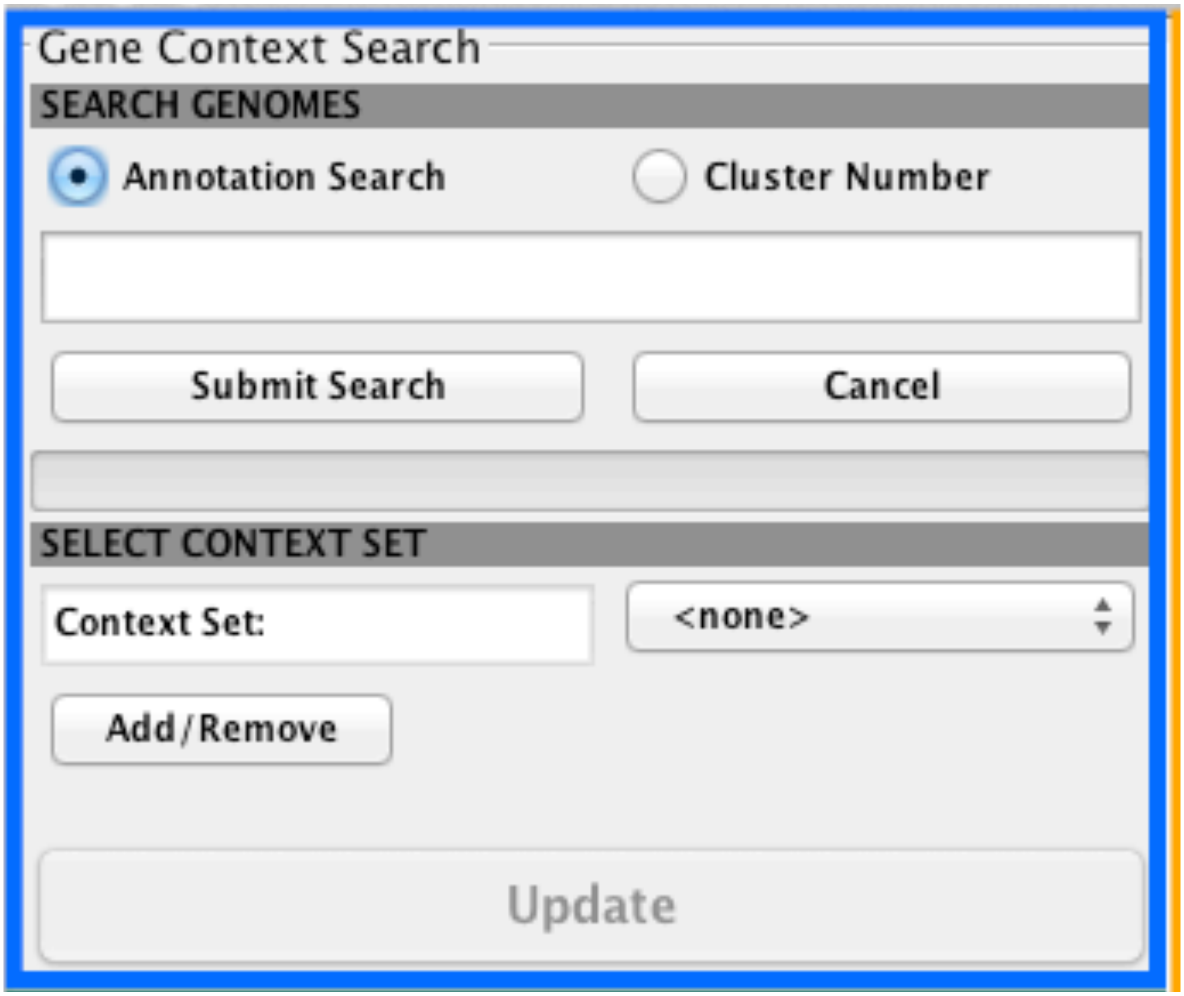

If the **Annotation Search** radio button is selected, then text strings will be searched against gene annotations. Searches are case-insensitive, and will return partial matches. For example, a search of “gluco”, for example, will return hits for genes such as “glucose”, “glucokinase”, “glucose regulator”, and “glucocorticoid”. **Annotation searches are case-insensitive**: “glucose” and “GLUCOSE” are both exact matches to “Glucose”.

If the **Cluster Number** radio button is selected, then integral values will be searched against assigned gene cluster numbers. In this case, only exact matches

will be returned. For example, a search of “43” will return all genes with cluster number 43.

<u>“OR” statement</u>: separate queries with a semicolon ; .

For example, an annotation search of

hexokinase; glucose

will return all genes with annotations that contain either the text string “hexokinase” or “glucose”. An annotation search of

hexokinase; glucose; glycerol; nitrogen

will return all genes with annotations that contain at least one of the text strings “hexokinase”, “glucose”, “glycerol”, or “nitrogen”.

This works as well for cluster IDs: a cluster ID search of

1; 65; 534

will return all genes with cluster ID 1, 65, or 534.

<u>“AND” statement</u>: separate queries with a double-dollar sign $$.

The “AND” statement operates following the application of the context set – suppose that a search for “452” with an “operon” context set yields a set of genes that have clusters 451, 452, 453, and 454 in some organisms and a set of genes that have cluster 451, 452, and 453 in others. Searching for 452 $$ 454 will return only those operons that contain both 452 and 454.

<u>“IF AND ONLY IF” clause</u>: start query with &&only

Consider the previously described scenario. If you’d like to retrieve all operons that contain 451, 452, and 453 but not 454, you can search as follows:

&&only 451 $$ 452 $$ 453

the “&&only” requires that only exat matches be returned, the “451 $$ 452 $$ 453” specifies that the genomic groupings must contain 451, 452, and 453.

to specify a <u>continuous range of clusters</u>, use a dash.

For example, a cluster ID search of

46-48

will return all genes with cluster ID 46, 47, or 48. Note that this is identical to the query

46; 47; 48

The **Cancel** button may be used to either cancel **(1)** a popular genome set being imported or **(2)** a search query / context tree rendering.

After a search has completed, a message will appear in the console listing the total number of matches. The progress bar below the search bar will display the approximate progress of the search.

Under the **Select Context Set** banner, it is possible to select the currently active context set from the drop down menu. When a search is performed, gene groupings are returned according to whichever context set is selected. The **Add/Remove** button allows you to **Add** or **Remove** a context set, as you see fit. This is explained in more detail on page 68, **Context Set.**

Finally, the large **Update** button will become enabled when one or more **Search Results Frames** are available. When a **Context Tree** is drawn, you may wish to display the resulting tree with a different font or different style. These settings can be changed in the **Context Tree sub-panel** (Explained in more detail on page 18, **Search Options Area**). Changes will take effect with a push of the **Update** button.

SEARCH OPTIONS AREA

The **Search Options Area** provides options for Search Results, Context Tree drawing, and loading one or more phylogenetic trees or Sequence Motifs. It is located in the lower left-hand corner of the main frame, and looks like this:

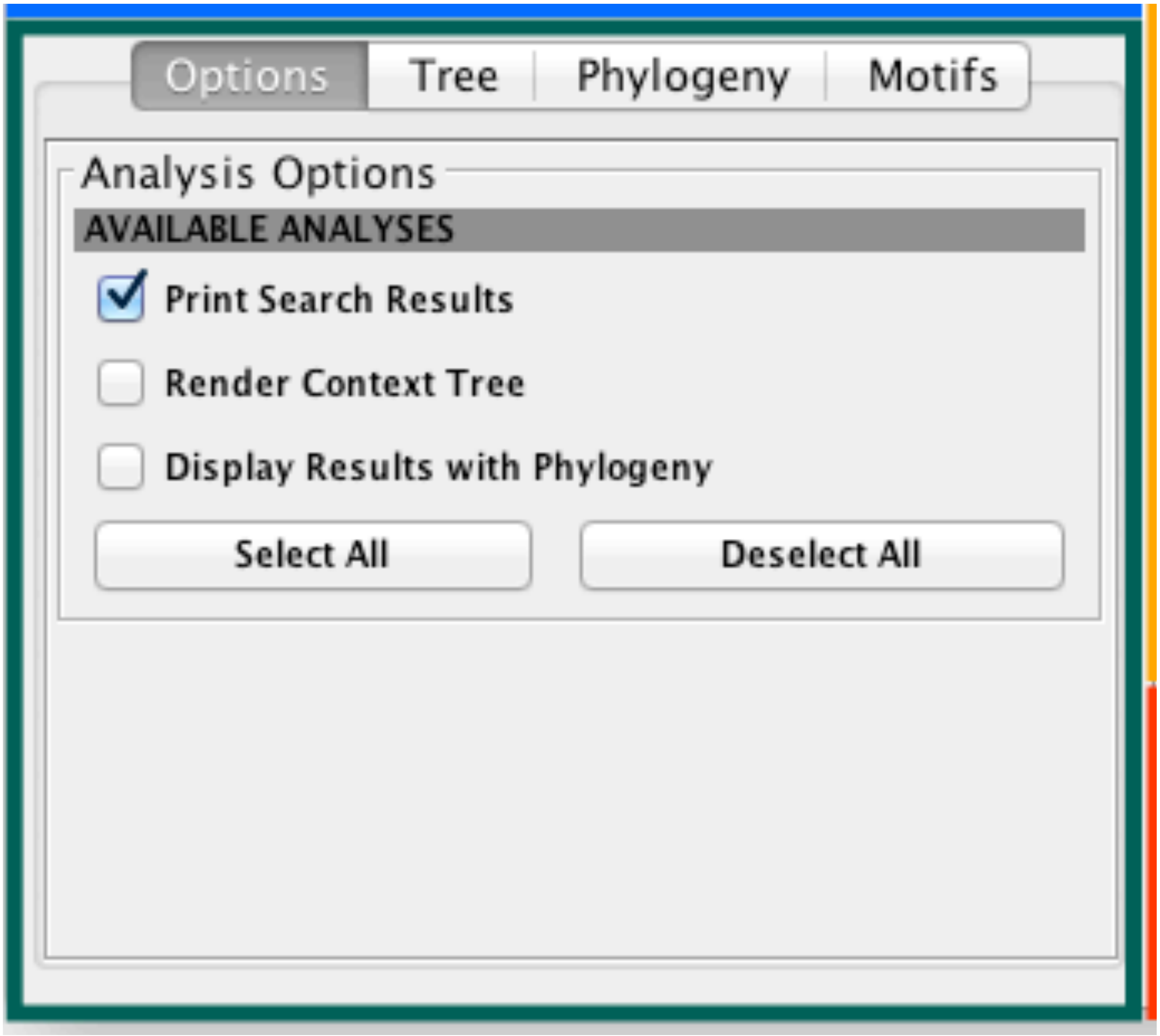

This area contains 4 tabbed panes: **Options** (shown), **Tree** (explained in next section), **Phylogeny** (explained in the **Phylogenetic Tree** section, on page 95), and **Motifs** (explained in the **Sequence Motifs** section, on page 98).

When you have entered a search in the search bar in the **Genome Set Search Area** (upper left-hand corner of main frame), a new internal frame will appear in the **Internal Frame Management Area**, showing up to 3 results panes. The

Options pane allows you to specify which results to include –Search Results can be displayed, a Context Tree can be rendered, and a Phylogenetic tree can be drawn with the frame. **Any one or all 3 options may be selected**. Pushing the **Select All** button will select all options, pushing the **Deselect All** button will deselect all options.

If no boxes are checked, the Print Search Results box will become checked, and search results will be displayed. If the phylogenetic tree box is checked, and no phylogenetic tree is loaded, then this option will become unchecked, and this pane will not be drawn.

Context Tree Options

Selecting the **Tree** tab in the **Search Options Area** displays the following panel:

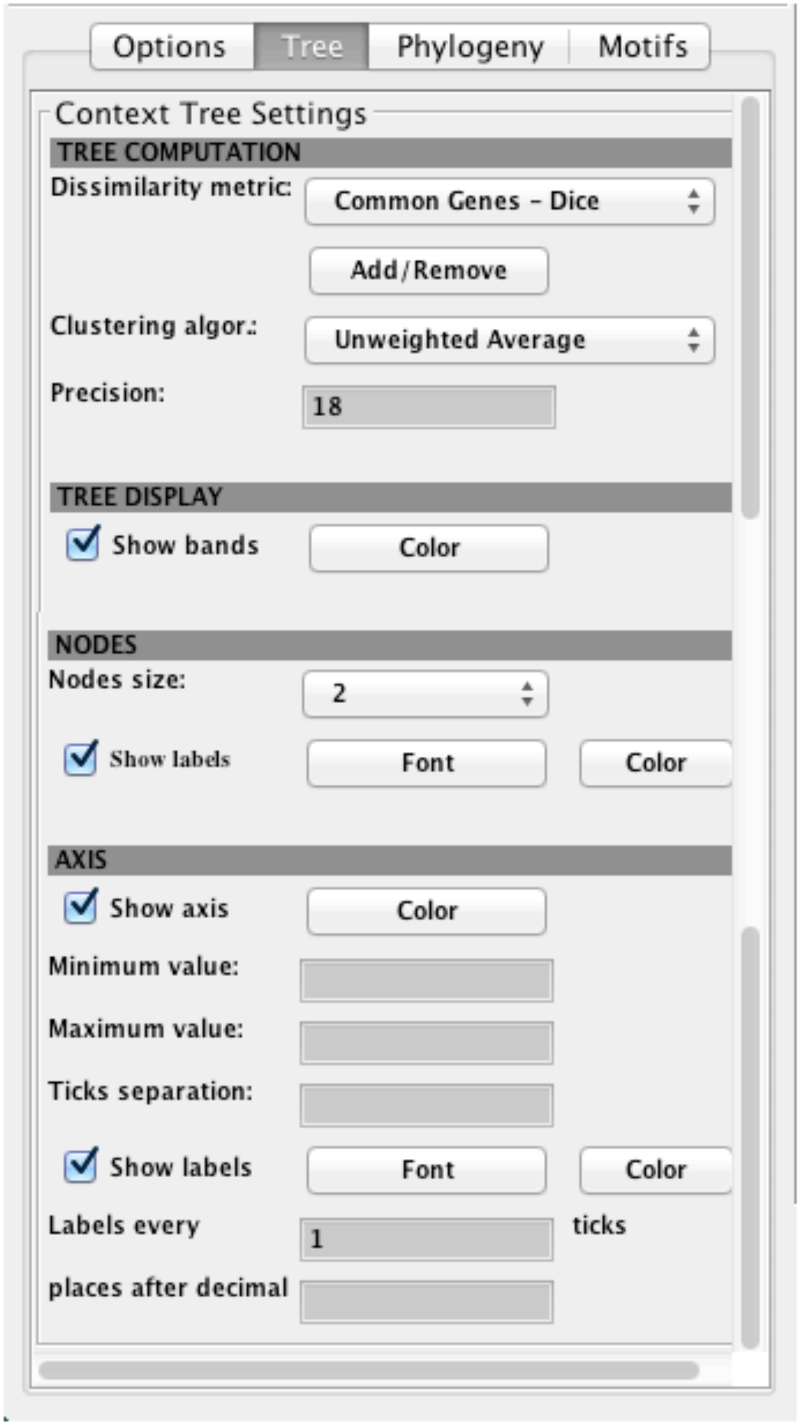

Under the **Tree Computation** banner, you may select an appropriate **Dissimilarity Metric** and **Clustering Algorithm**, from the appropriate drop-down menu. The **Add/Remove** button below the Dissimilarity Metric field allows you to create a customized Dissimilarity Metric (for more information, please see **Dissimilarity Measure,** page 76). The **Precision** Field allows you to specify the number of decimal places to use in the clustering step.

Under the **Tree Display** banner, there is an option to **Show Bands** or not, and then the option to specify a color for these bands. **Bands** demonstrate groups of nodes that have the same range of dissimilarities – for example, if the dissimilarity between nodes **A** and **B** is 0, and the dissimilarity between **B** and **C** is 0, then the dissimilarity between nodes **A** and **C must be zero**. However, depending on the algorithm, **the dissimilarity between A and C might not come out to be 0**. Suppose, for example, that the computed dissimilarity between **A** and **C** is 0.1. In that case, **A, B,** and **C** will all be grouped together, with a dissimilarity band between 0 and 0.1. If **Show Bands** is selected, you will see the range of dissimilarities, if it is not, then you will see the **smallest value** – **nodes A, B, and C, will all have a dissimilarity of 0**.

The **Banding** case is the result of **Variable group agglomerative hierarchical clustering**, where the order of comparisons does not matter. For a more complicated discussion of bands in variable group agglomerative hierarchical clustering, please see

Gomez, S., Fernandez, A., Montiel, J., & Torres, D. (n.d.). Solving Non-Uniqueness in Agglomerative Hierarchical Clustering Using Multidendrograms. *Journal of Classification*, *65*, 43-65. doi:10.1007/s00357-008-

Under the **Nodes** banner, you may specify the size of the nodes, and optionally show the labels, change the font, and change the color.

Under the **Axis** banner, you may change various properties of the axis.

When editing an existing Context Tree, make the changes in this panel, and click the Update button located above this panel at the bottom of the Search Options Area. If nothing new needs to be computed, then the tree computation should be very fast.

INTERNAL FRAME MANAGEMENT AREA

The **Internal Frame Management Area** is the portion of the **Main Frame** where **Search Results** frames, **Context Trees**, and rendered **Phylogenetic Trees** appear in their own internal windows. These windows may be dragged around, minimized, maximized, and closed.

Internal frames appear in the upper right-hand corner of the main frame.

Pictured is a sample frame containing a **Search Result** frame, a **Context Tree**, and a **Phylogenetic Tree**:

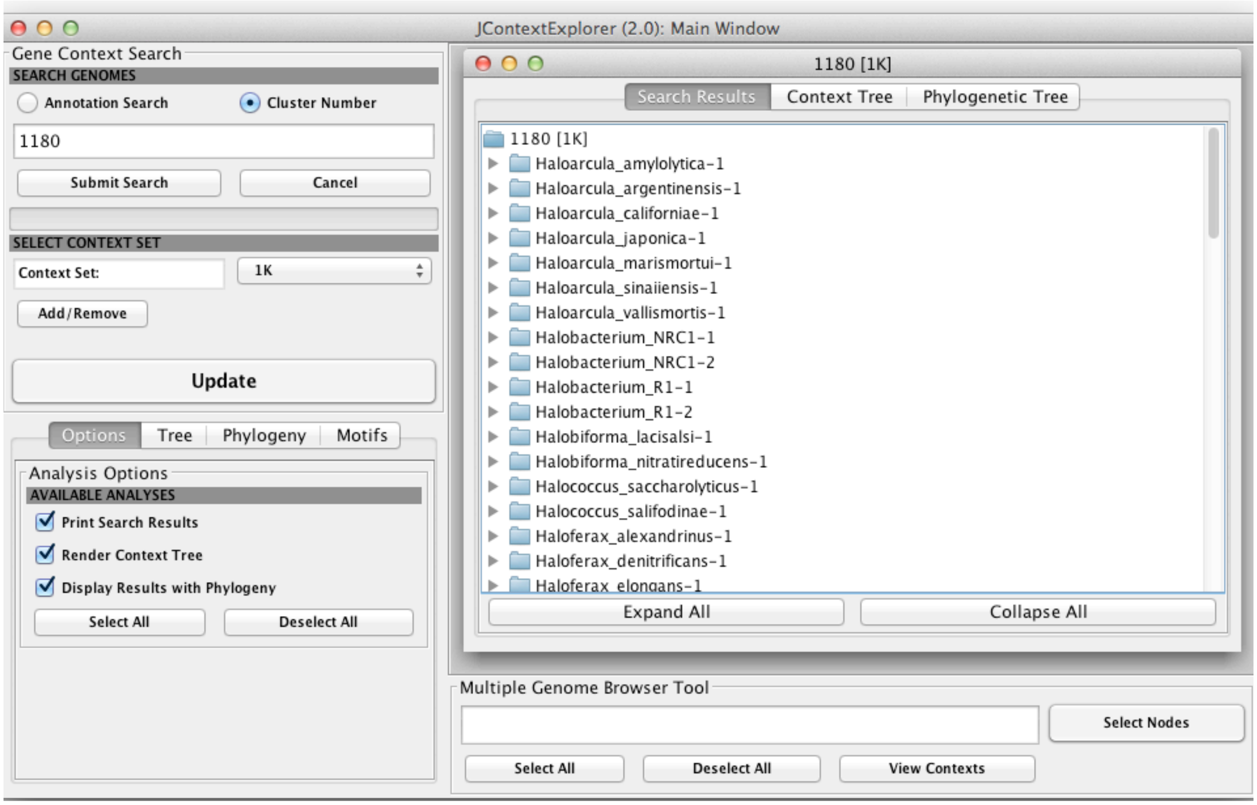

Search Results Frame

Pictured is the **Search Results** Frame from above:

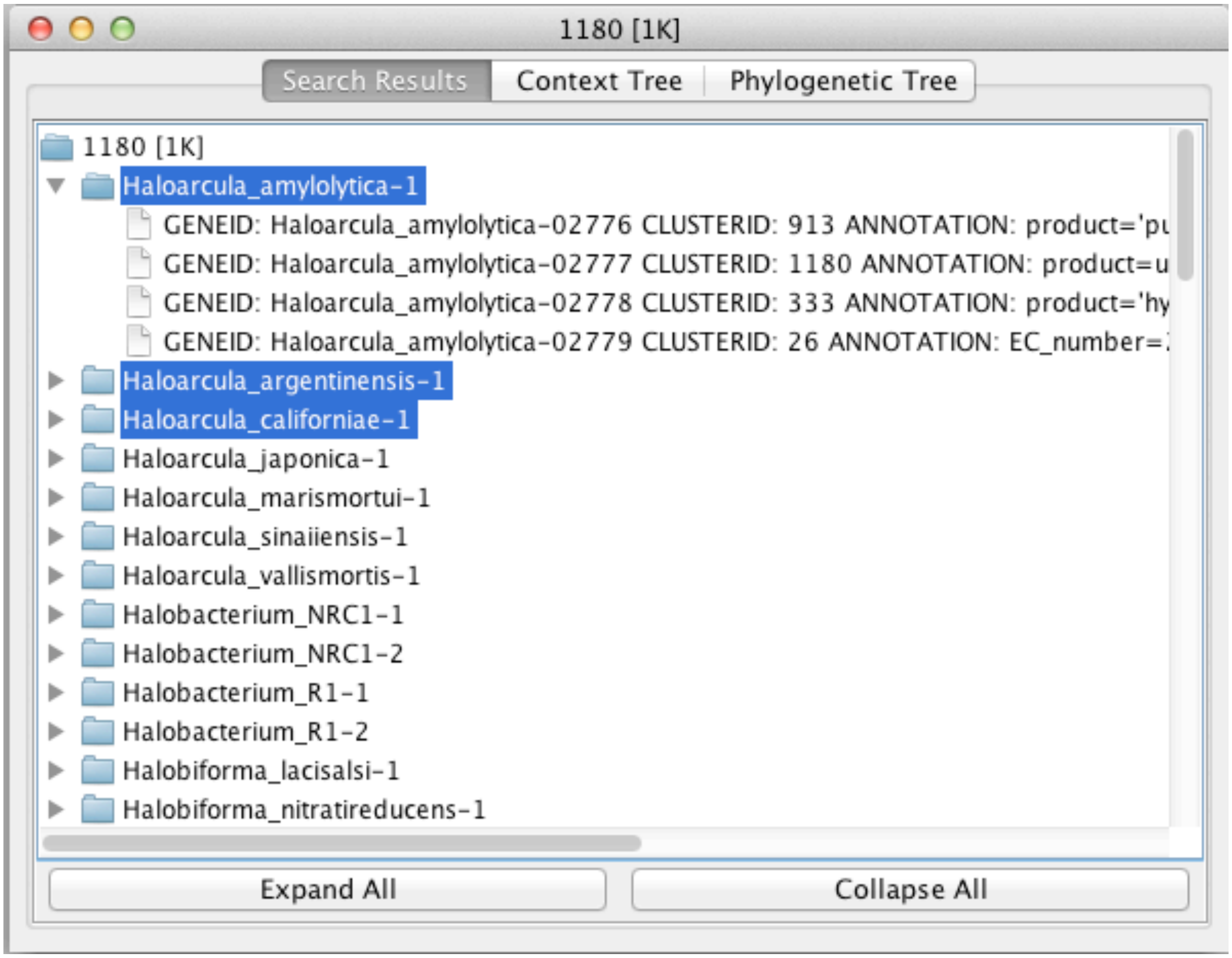

In the frame above, 3 **Genomic Groupings** are selected – Haloarcula_amylolytica-1, Haloarcula_argenintensis-1, and Haloarcula_californiae-1. Each genomic grouping is named according to the source organism, followed by a serial number, **showing the instance of a genomic grouping stemming from that organism.** All of the selected genomic groupings have a serial number of "1", indicating that each is the first genomic grouping (arbitrarily numbered) stemming from that organism.

Expanding each **Genomic Grouping** folder shows the genes included in that **Genomic Grouping**. The Gene ID, Cluster ID, and Annotation information is included for each gene in that genomic grouping.

Pushing the **Expand All** button expands all **Genomic Grouping** folders (showing all genes in all genomic grouping), while pushing the **Collapse All** button collapse all **Genomic Grouping** folders (hiding all genes in all genomic groupings).

Genomic Groupings may be selected by clicking on folders, or holding down SHIFT and selecting a range of folders, or holding down COMMAND or CTRL and selecting/de-selecting one or more folders.

<u>Export Options: Search Results Frame Menu Options</u>

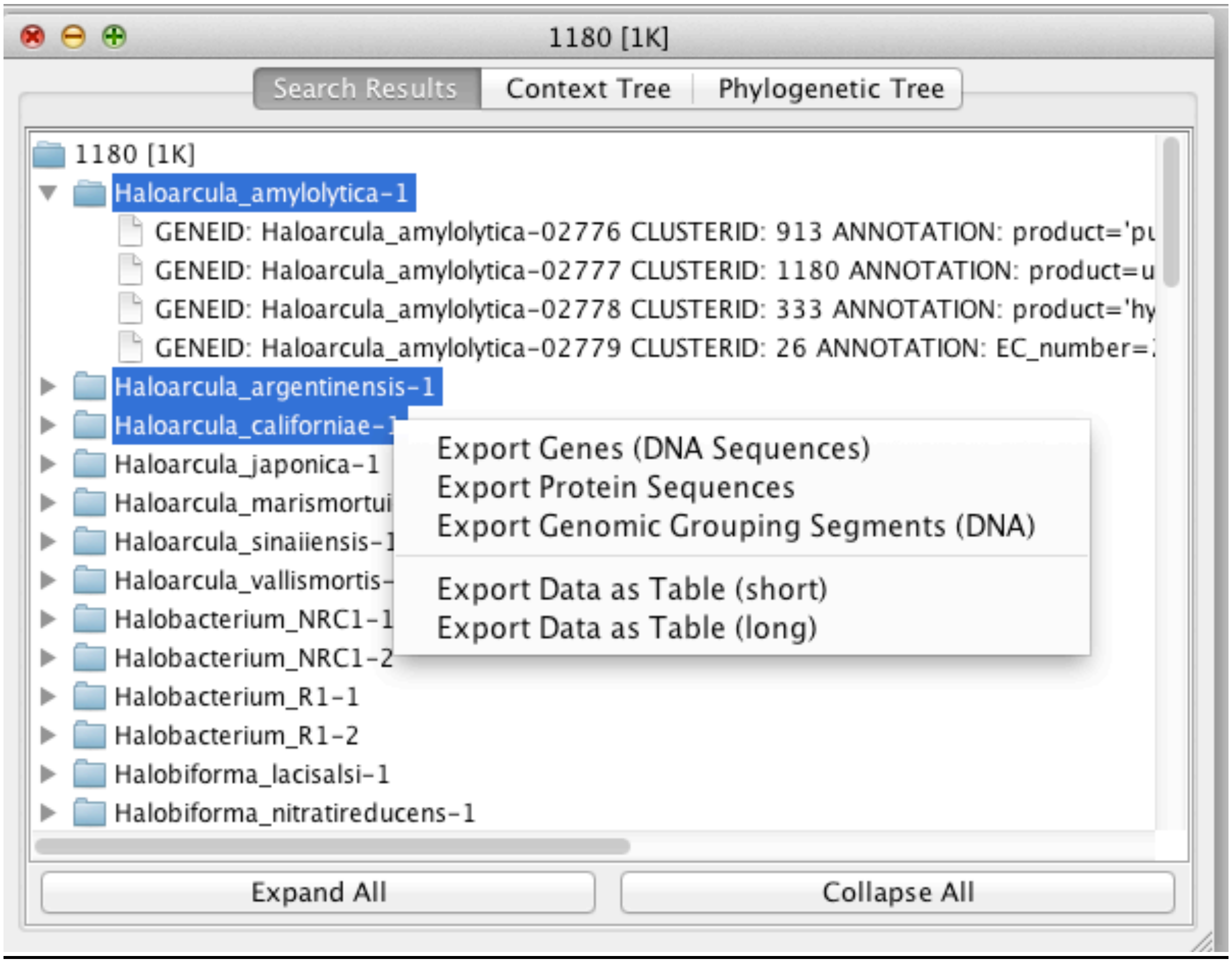

Right-clicking on the search results will cause a pop-up menu to appear where clicked (shown above). This pop-up menu offers several options to export data associated with the selected entries of the search results frame.

The firs half of the menu is associated exporting sequences associated with the sequences of individual genes, or entire genomic groupings, based on a set of pre-

loaded .fasta genome sequence files, named the same as the organism it is associated with (please see **Genome Sequence File(s),** page 63, for more information). "**Export Genes (DNA Sequences)**" will export the DNA of individual selected genes, or all component genes (if an entire genomic grouping folder is selected). "**Export Protein Sequences**" works the same way, except the DNA is translated into protien sequence. For these options, genes that are oriented in reverse complement on the genome form are output from the standpoint of the start of the gene. The last option, **"Export Genomic Grouping Segments (DNA)"**, will output the entire stretch of DNA represented in a single genomic grouping, without regard to the genes contained (from the earliest start site contained in the genomic grouping to the latest stop site in the genomic grouping). In this case, DNA is exported always according to the forward strand, intergenic DNA is included in the export, and no correction is made for genes existing on the reverse complement.

The second half of the menu is associated with exporting selected data entries in the table into a plain text file. the "Short" form (first option) exports exactly the information displayed in the table: **gene ID – cluster ID – annotation**. The "Long" form (second option) exports additional information. The information exported is as follows: **organism – contig – start – stop –strand- annotation – cluster ID – gene ID**.

Context Tree Frame

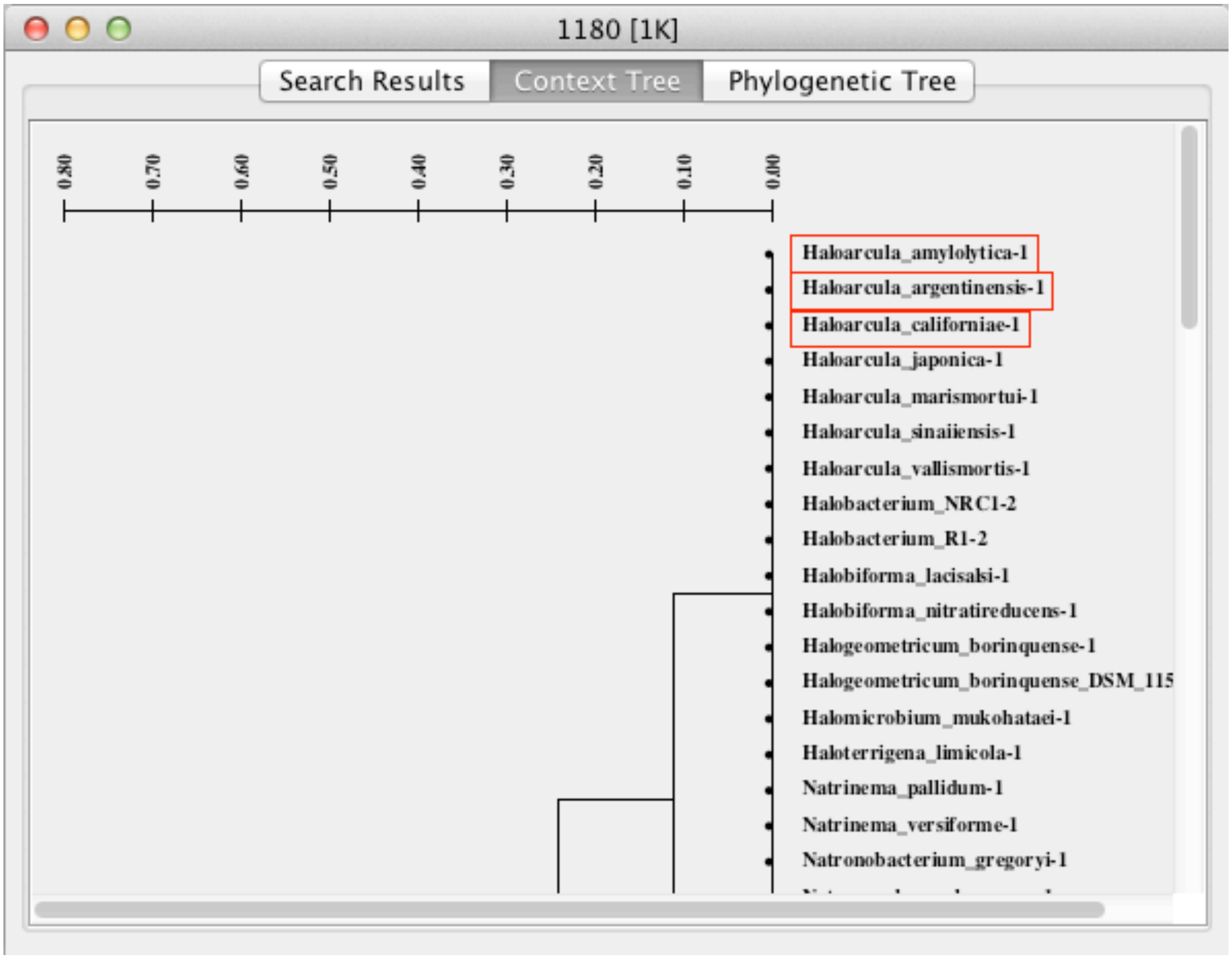

In the frame above, the same 3 **Genomic Groupings** are selected as shown in the **Search Results Frame** – Haloarcula_amylolytica-1, Haloarcula_argenintensis-1, and Haloarcula_californiae-1.

These genomic groupings were selected automatically in the Context Tree Frame, when selected in the Search Results Frame.

The same is true for the Phylogenetic Tree Frame – **Selecting or de-selecting a node in one frame will select it in all connected frames.**

In the **Context Tree** frame, selected nodes are indicated with a red box around the node name. Nodes may be selected by clicking directly on the node name, and again by using SHIFT+ clicking and COMMAND/CTRL clicking.

Relationships between the **genomic groupings** are indicated by the topology of the tree.

<u>Context Tree Menu Options</u>

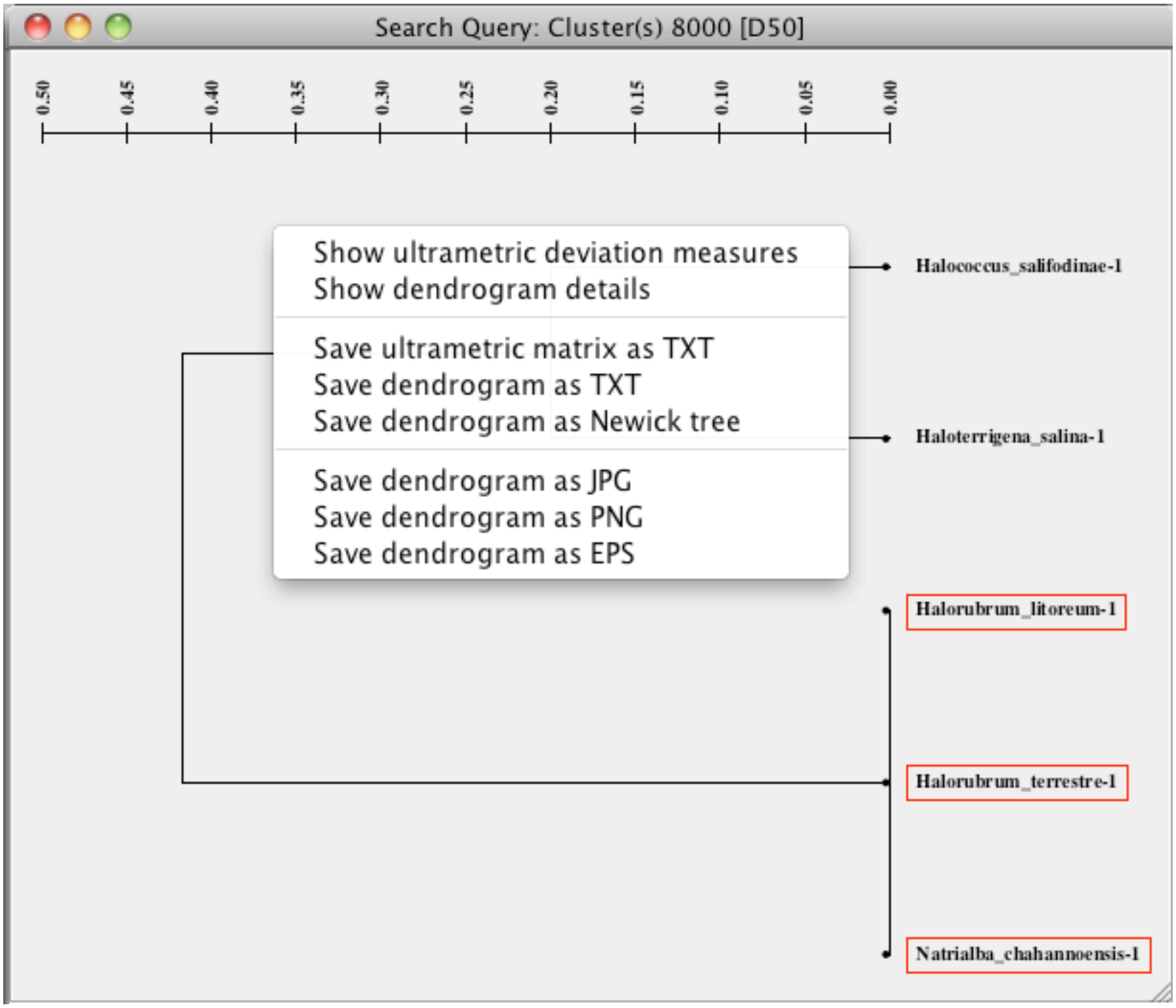

Right-clicking anyway on the frame will bring up the pop-up menu shown in the figure above.

These options are borrowed from the original MultiDendrograms software package:

Gomez, S., Fernandez, A., Montiel, J., & Torres, D. (n.d.). Solving Non-Uniqueness in Agglomerative Hierarchical Clustering Using Multidendrograms. *Journal of Classification*, *65*, 43-65. doi:10.1007/s00357-008-

A complete user manual is available at

http://arxiv.org/abs/1201.1623

Please refer to this documentation for more information.

Phylogenetic Tree Frame

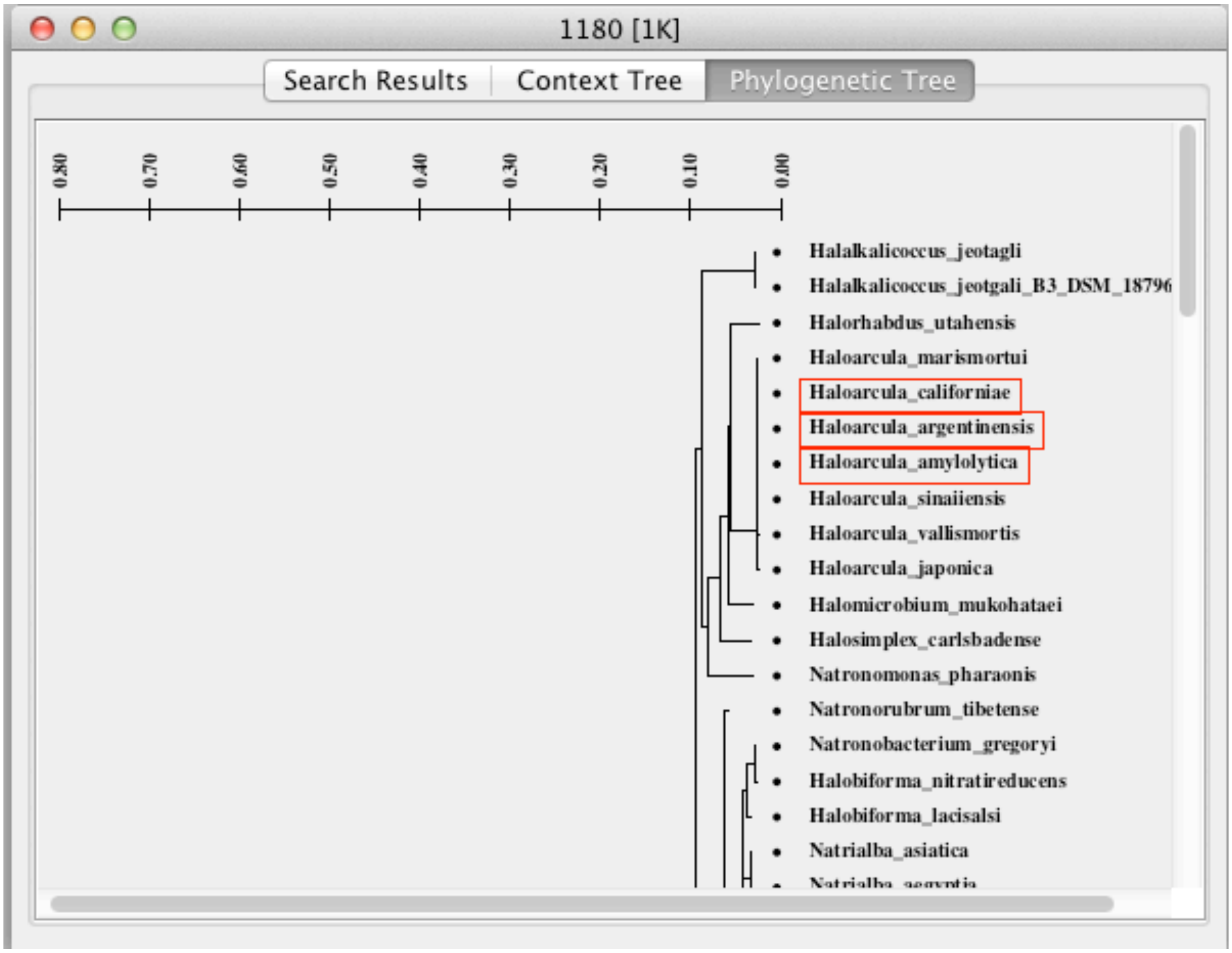

In the frame above, the same 3 **Genomic Groupings** are selected as shown in the **Search Results Frame** and **Context Tree Frames** – Haloarcula_amylolytica-1, Haloarcula_argenintensis-1, and Haloarcula_californiae-1. In this case, the nodes are named according to the Species Names, not the genomic groupings.

The Phylogenetic Tree frame is very similar to the Context Tree frame, and is governed by the same set of **Tree** options.

All genomic groupings deriving from the same organism will be selected in Context Tree / Search Results frames, when an organism is selected in the Phylogenetic Tree Frame.

For example, if 4 genomic groupings exist from organism X, selecting organism X in the phylogenetic tree will select all 4 of these nodes in the context tree and search results. Conversely, selecting any one of the genomic groupings stemming from organism X in the search results or context tree frame will select organism X on the phylogenetic tree.

Additional Node Selection Options

Additional node selection options exist – please see the next section for instructions!

Search Results Analysis Area

The **Search Results Analysis Area** provides a search bar for efficient node selection, and context visualization via the **Multiple Genome Browser**. It is located in the lower right-hand corner of the main frame, and looks like this:

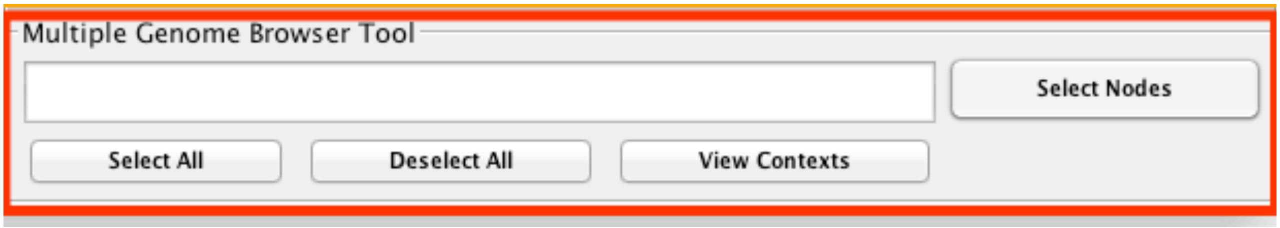

Note that this search bar is for **node selection only**.

Typing one or more key words in the bar, **separated by comma, space, or semicolon** will select all nodes containing at least one of the textual fragments. These searches are **case-insensitive**.

You may also select a subset of the nodes based on content.

To select all genomic groupings that contain a gene with a particular gene id, type **GENEID:<gene ID goes here>**.

To select all genomic groupings with a particular cluster ID, type **CLUSTERID:<cluster ID goes here>**.

To select all genomic groupings containing a gene with a particular annotation fragment, type **ANNOTATION:<annotation fragment goes here>**.

Finally, to select all genomic groupings containing a gene with an associated motif, type **MOTIF:<associated motif goes here>.**

You may combine the above tagged filtration searches with ordinary node keyword searches – for example, continuing the example shown in the **Internal Frame Management Area** section (see page 22), typing

GENEID:Haloarcula_amylolytica-02776; Haloferax

will select all genomic groupings stemming from organisms from the genus Haloferax, as well as the genomic grouping Haloarcula_amylolytica-1.

All nodes may be selected by pushing the **Select All** button, and all nodes may be deselected by pushing the **Deselect All** button.

A multiple genome browser window will appear highlighting only the selected nodes in the currently active search results frame when the **View Contexts** button is pushed.

Context Viewer Multiple Genome Browser

One of the most powerful and important features of JContextExplorer is the multiple genome browser. **We recommend using this feature with almost every analysis you perform in JContextExplorer.** By viewing the actual genomic segments, you may develop an intuition for how context trees are built, and why, exactly certain genomic groupings end up grouped together and others are grouped apart.

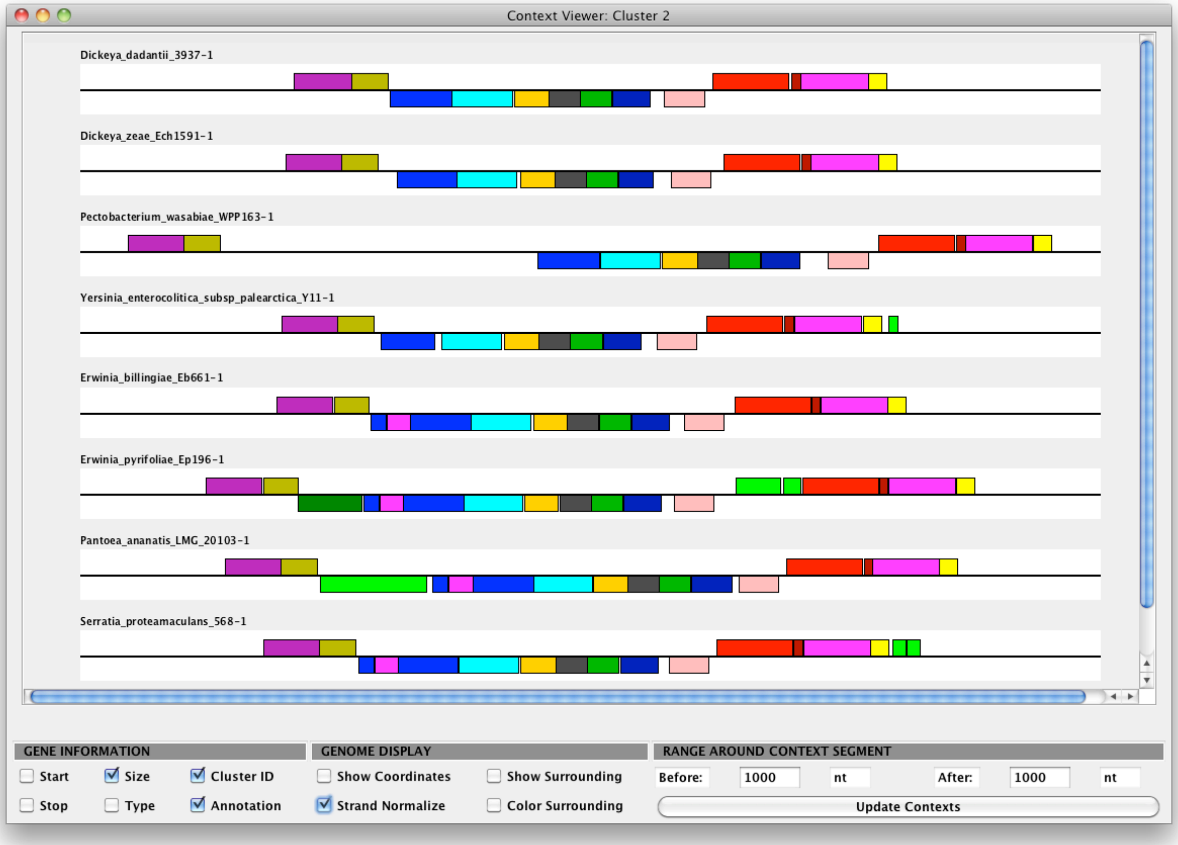

The purpose of this frame is to visualize the genomic groupings associated with the leaves on the active context tree. Annotated features are rendered as colored rectangles (**colored according to common homology cluster ID number or common annotation, depending on how the context tree was generated**) resting either above (for features on the forward strand) or below (for features on the reverse strand) a single black line, in the order they appear in the associated

annotated genome. The associated node name is printed above and to the left of each rendered genomic segment.

The Multiple Genome Browser is an active frame. Left clicking, right clicking, and center clicking on individual genes and parts of the frame do different things. Individual **Option sub-panes** in the bottom left, bottom center, and bottom right also have interactive effects.

<u>**Gene Information**</u>

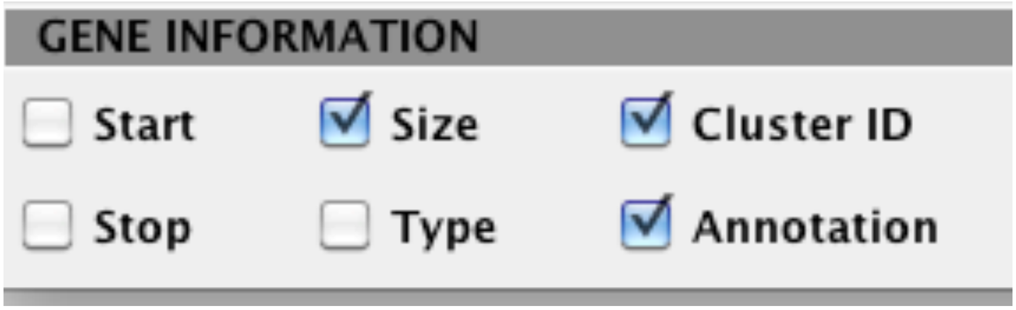

This is the **Gene Information sub-pane**. These check boxes describe which biological information should be displayed upon **left clicking** on an individual annotated feature in the Multiple Genome Browser frame.

<u>**Genome Display**</u>

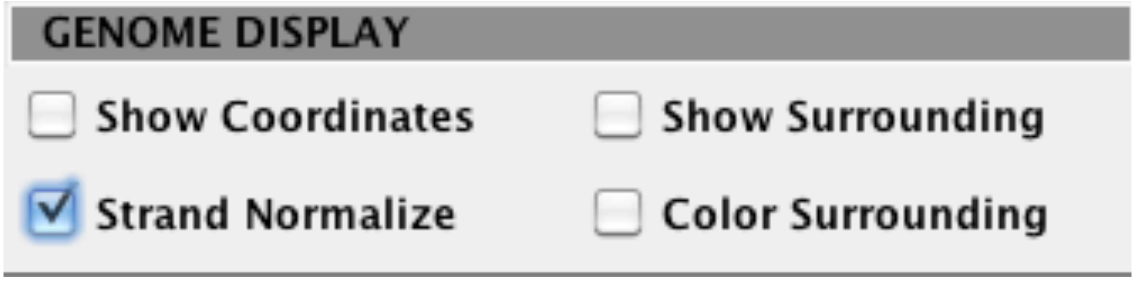

This is the **Genome Display sub-pane**. These check boxes describe how whole genomic segments should be rendered in the above **Multiple Genome Browser** frame.

if **Show Coordinates** is selected, numerical values will appear below individual rendered genomic segments displaying coordinates every 1000 nt or so. The name of the sequence will also appear in the upper-left hand corner, and a small

triangular **flag** will appear in the upper left-hand corner of each genomic segment, pointing in increasing order. If this flag is black (and pointing to the right), the sequences are increasing left to right, if the flag is red (and pointing to the left), the sequence is displayed in reverse complement, and so is increasing right to left.

If **Show Surrounding** is checked, annotated features that are not a member of the genomic grouping associated with the genomic segment displayed will also be displayed. These features may either be displayed as colored or gray, depending whether or not **Color Surrounding** is checked or unchecked.

If **Color Surrounding** is checked, annotated features will be colored according to common homology group ID or common annotation, just as the genomic groupings are colored. If **Show Surrounding** is unchecked, this option has no effect.

If **Strand Normalize** is checked, individual genomic segments may be displayed in sequence reverse complement so that query matches are on the forward strand. If the genomic segment is already oriented such that query matches are displayed on the forward strand, this option has no effect.

Range to Display

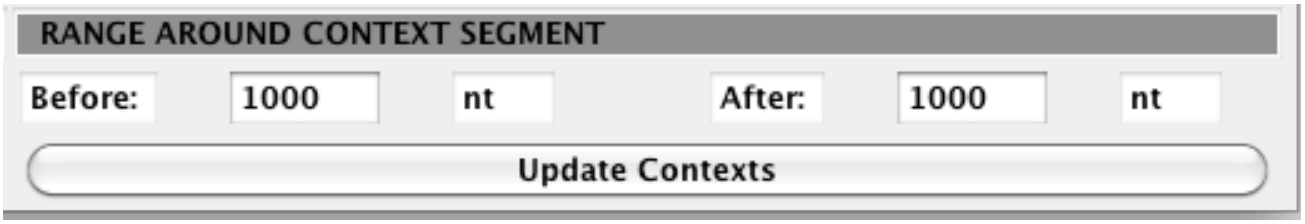

This is the **Range to Display sub-pane**. This controls how much of the surrounding genomic region should be displayed along with individual genomic groupings. Changing values in the "Before" and "After" text fields, and clicking the **Update Contexts** text field will re-render all genomic segments in the range to display sub-pane.

The **Update Contexts** button is also linked to the leaves selected on the associated **Context Tree** for the rendered contexts: **You may change the**

rendered contexts by changing the leaves selected in the context tree frame and pushing the Update Contexts button.

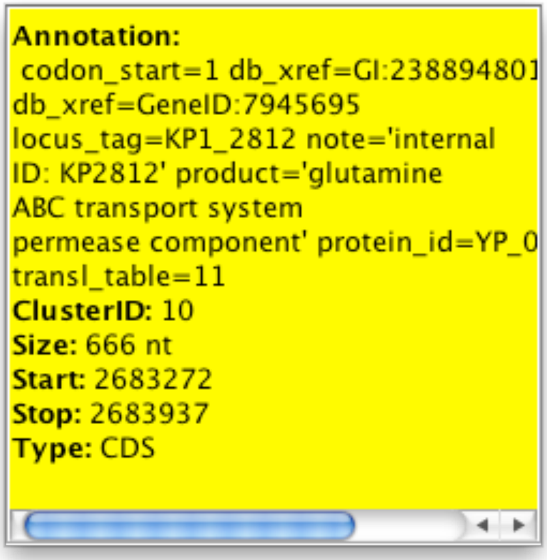

This is the **gene information sub-frame. Left clicking** on an individual annotated feature results in a small, yellow box appearing at the point of clicking, displaying biological information about the annotated feature clicked.

Left click on another part of the frame that does not contain an annotated feature to make this frame disappear; left click on a different annotated feature to display biological information for that annotated feature.

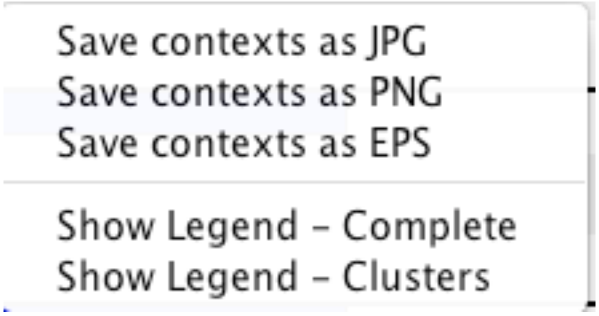

Right clicking anywhere on the frame opens the pop-up menu displayed above. left clicking away causes this popup menu to disappear.

Selecting any of the **image export** options will open a file dialog allowing for image export. In the image export, only the rendered genomic contexts will

appear, and they will always appear exactly as they do on screen. Selecting any of the **Show Legend** options will launch the **Gene Color Legend** frame (please see **Gene Color Legend Frame** below).

Middle (or center) clicking on a particular annotated feature will select all other annotated features with the same homology cluster or annotation (depending on the initial search type).

You may hold down the **CTRL** or **SHIFT** key while middle clicking, which will allow for selection of multiple annotated feature groups. If you have the **Gene Color Legend** frame open, then the entry associated with this annotated feature will also appear selected (surrounded by a thin, red rectangle).

Gene Color Legend Frame

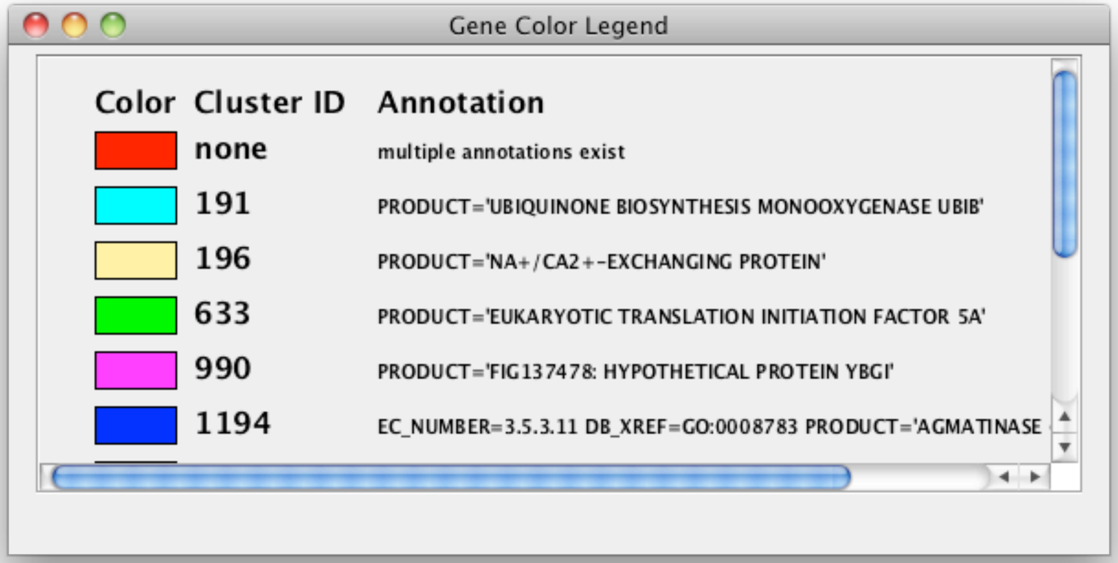

This is the Gene Color Legend frame. It contains the mapping between colors, cluster ID, and annotations associated with its parent **Multiple Genome Browser** frame.

The Gene Color Legend is an active frame. You may **Left Click / Middle Click** or **Right Click on the rows of the table in the frame (color box, cluster ID, annotation information)**. If you Left or Middle click, you will select the associated

color – clusterID – annotation relationship in the frame, as well as in the parent Multiple Genome Browser window.

Holding down the **CTRL** key while clicking on a color – clusterID – annotation mapping will select that mapping (if it unselected) or deselect that mapping (if it is selected), **without changing the selection profile of the other mappings**.

Holding down the **SHIFT** key while clicking on a leaf node will select every mapping between the currently selected mapping and the closest previously selected mapping.

Selections in this frame will appear in the parent **Multiple Genome Browser** frame.

If you right click anywhere on the frame, you will open a pop-up menu allowing for various figure export options (as **.jpg, .png**, or **.eps** files).

GENOMES MENU

JContextExplorer works within a set of defined annotated genomes, or a **Genome Set**. JContextExplorer includes functionality to create, modify, delete, and switch between **multiple genome sets simultaneously**. Because a major hurdle to bioinformatics analysis is often the retrieval and coordination of genomes from a diverse array of sources, JContextExplorer has been specifically designed to make retrieving, handling, and interacting with the source genome data easy. **If a genome is publically available somewhere, it is often possible with a click of a button to stream it into JContextExplorer**.

Genomic Information may be uploaded in JContextExplorer-unique files called **Genome Set files (.gs files)**, standard bioinformatics individual genome files (either via a series of extended **Genomic Feature Files (.gff files)** or **GenBank files (.gbk or .gb files)**, or streamed in directly from the NCBI genomes database repository or from the JContextExplorer base website. Genomes from one source may be easily combined with another, and genomes may be viewed, added, and removed, as can whole genome sets.

JContextExplorer is designed to coordinate between multiple, relatively small (about 100 genomes or fewer) genome sets. For questions to be posed to a genome set of more than 100 genomes, we suggest breaking apart this set into multiple genome sets, and amalgamating the results later. Alternatively, it is possible to launch JContextExplorer providing the Java virtual machine with a large maximum heap size.

The Genomes Menu may be selected from the main menu bar, and when expanded looks like this:

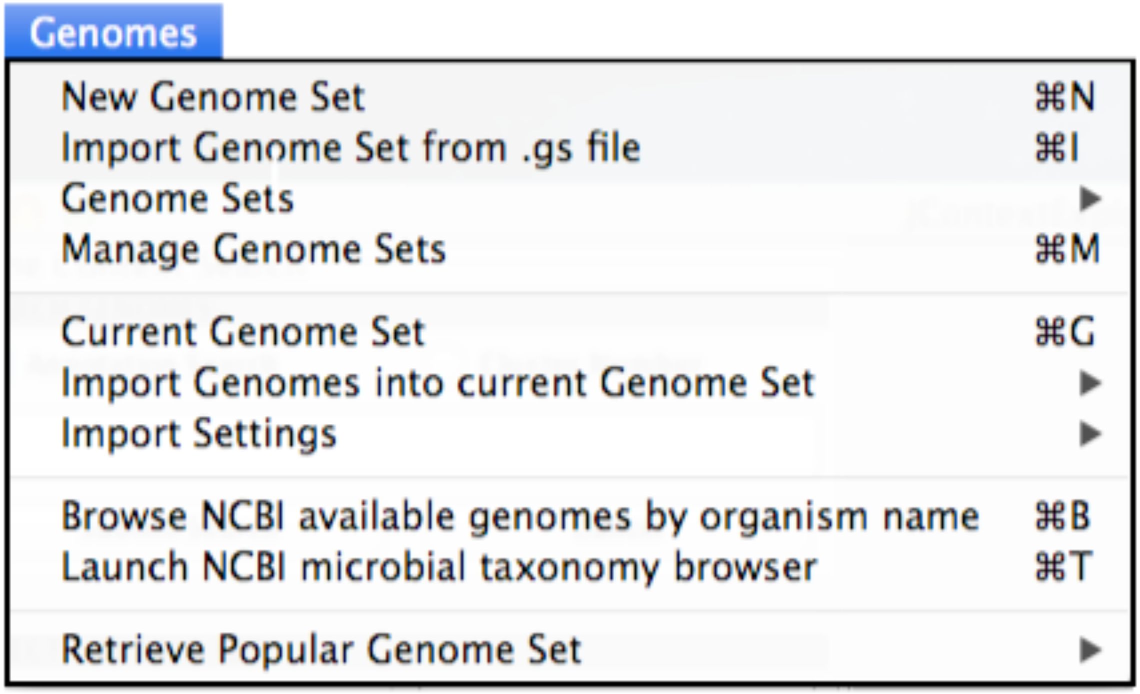

NEW GENOME SET (⌘N)

A **Genome Set** is a collection of one or more annotated genomes. To create a new, empty genome set, select **New Genome Set** from the **Genomes** drop-down menu, or ⌘N. The following window will appear:

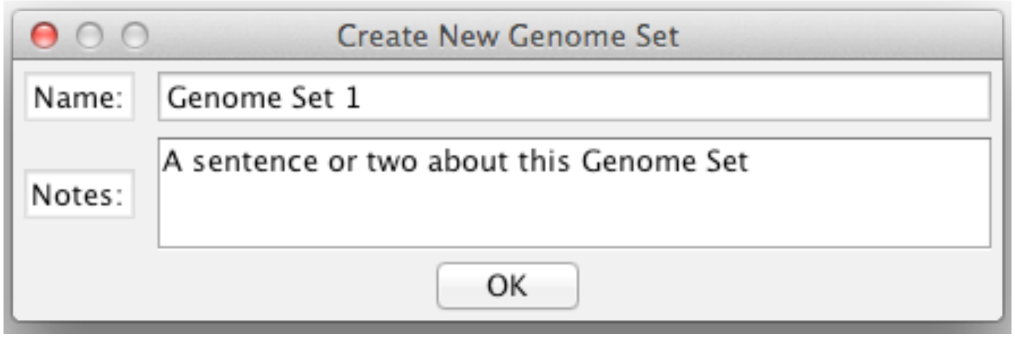

Name your Genome Set in the **Name** Field with a unique name, and provide a few notes about the genome set (if desired) in the **Notes** field. Clicking OK will initialize the genome set and close the window. If you click **OK** without providing a name, no new genome set will be created.

When you create a new genome set, you will be asked if you would like to switch to this new genome set, if another genome set exists:

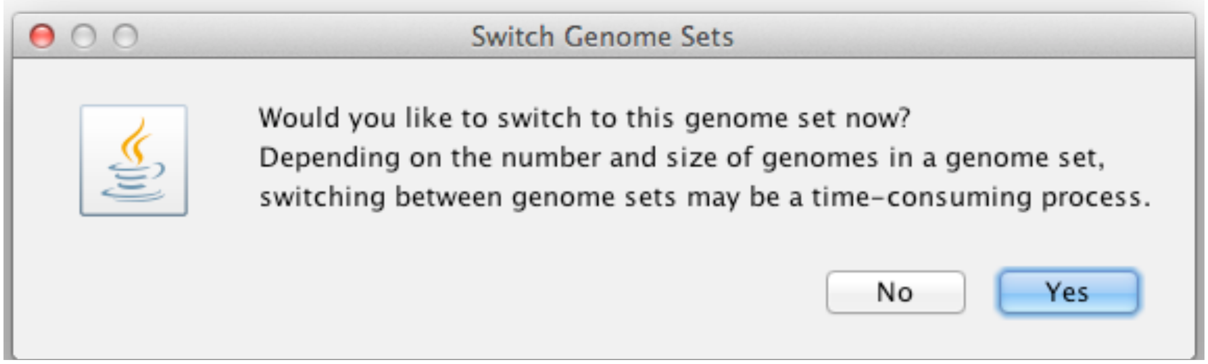

When you switch out of one genome set into another, the data in the original genome set is written to a file. When you switch back to this genome set, the data is retrieved from the file.

Any genomes you import will be associated into the current genome set.

IMPORT GENOME SET FROM .GS FILE (⌘I)

All of the information associated with a Genome Set – genomes, homology clusters, gene IDs, sequence motifs, phylogenetic trees, custom dissimilarity measures, etc – is stored in an exportable/importable JContextExplorer-unique format called a **.gs file** (“gs” for genome set). These files may be created at any time using JContextExplorer and exported.

Selecting “Import genome Set from .GS file” from the drop-down menu or typing ⌘I brings up the following file dialog box:

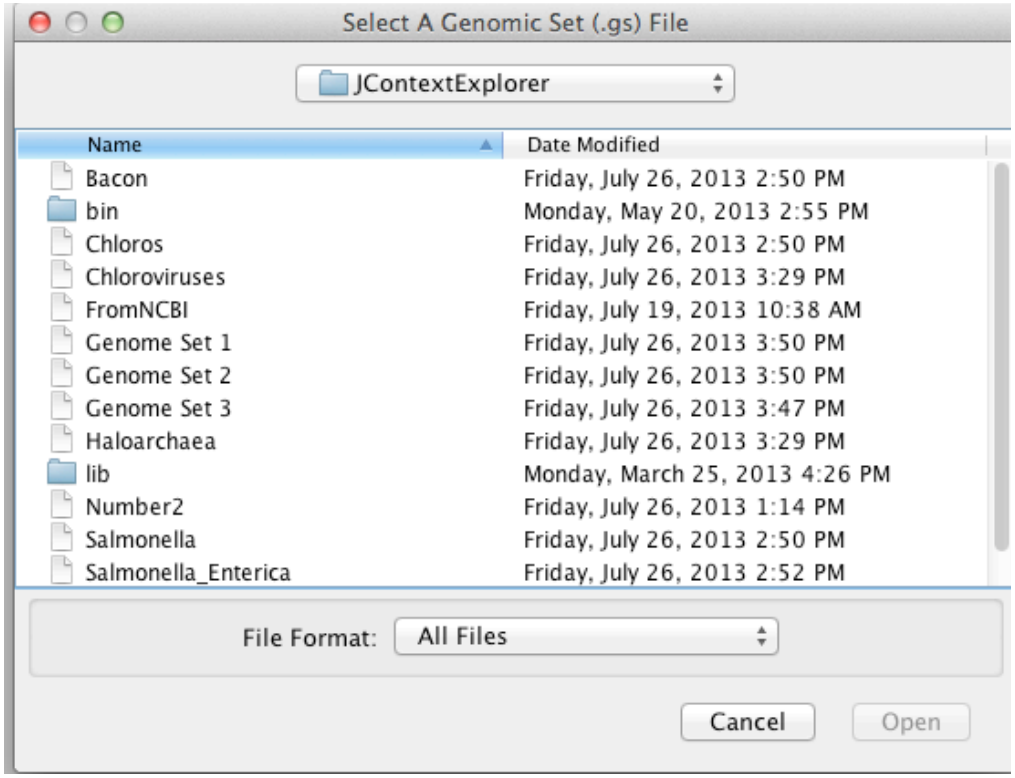

Selecting the desired **.gs** file will create the appropriate genomic set.

GENOME SETS

To switch from one genome set to another, simply identify the genome set you would like to switch into (by name) in the **Genome Sets** sub-menu, and select this set. The currently selected set will be designated with a check mark:

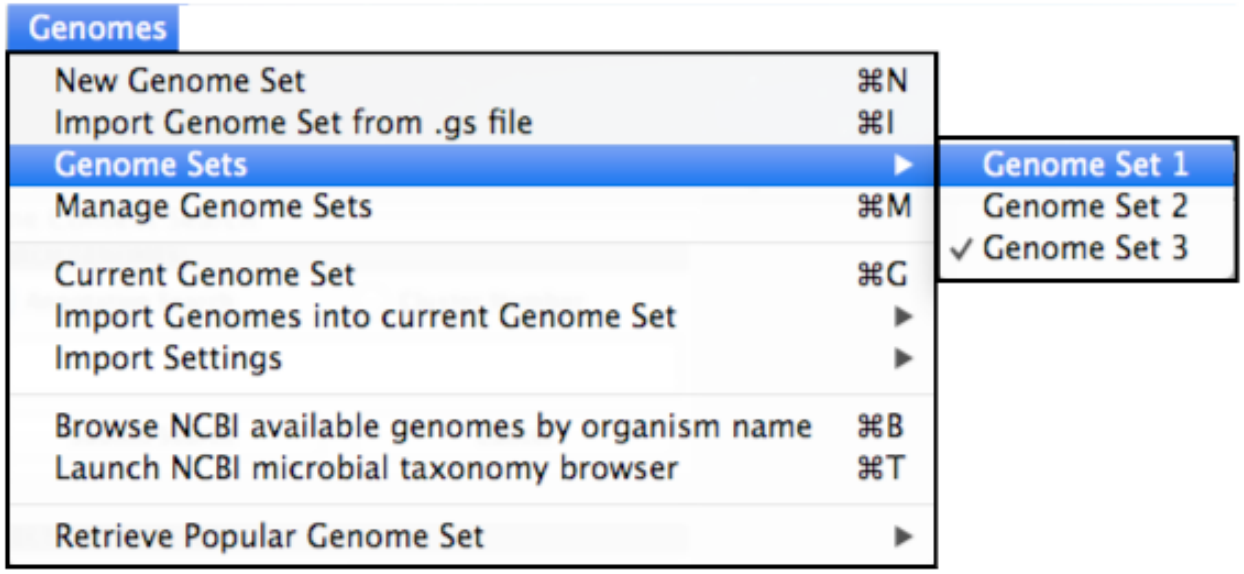

When switching from one genome set to another, all of the information associated with that genome set (genomes, dissimilarity measures, context sets, sequence motifs, phylogenetic trees, and all other supplementary information) is written to a temporary file. When switching back into that set, the information in that file is streamed back into JContextExplorer from this file.

Note that analyses carried out with one Genome Set will be retained in the main window – however, to explore these in depth (for example, browsing a set of contexts in the multi-genome context viewer browser), you will be asked if you would like to switch back to the old genome set.

MANAGE GENOME SET (⌘M)

To remove one or more genome sets from your current session of JContextExplorer, or view the contents of each genome set, select **Manage Genome Sets** from the **Genomes** drop-down menu, or type ⌘M. The following window will appear:

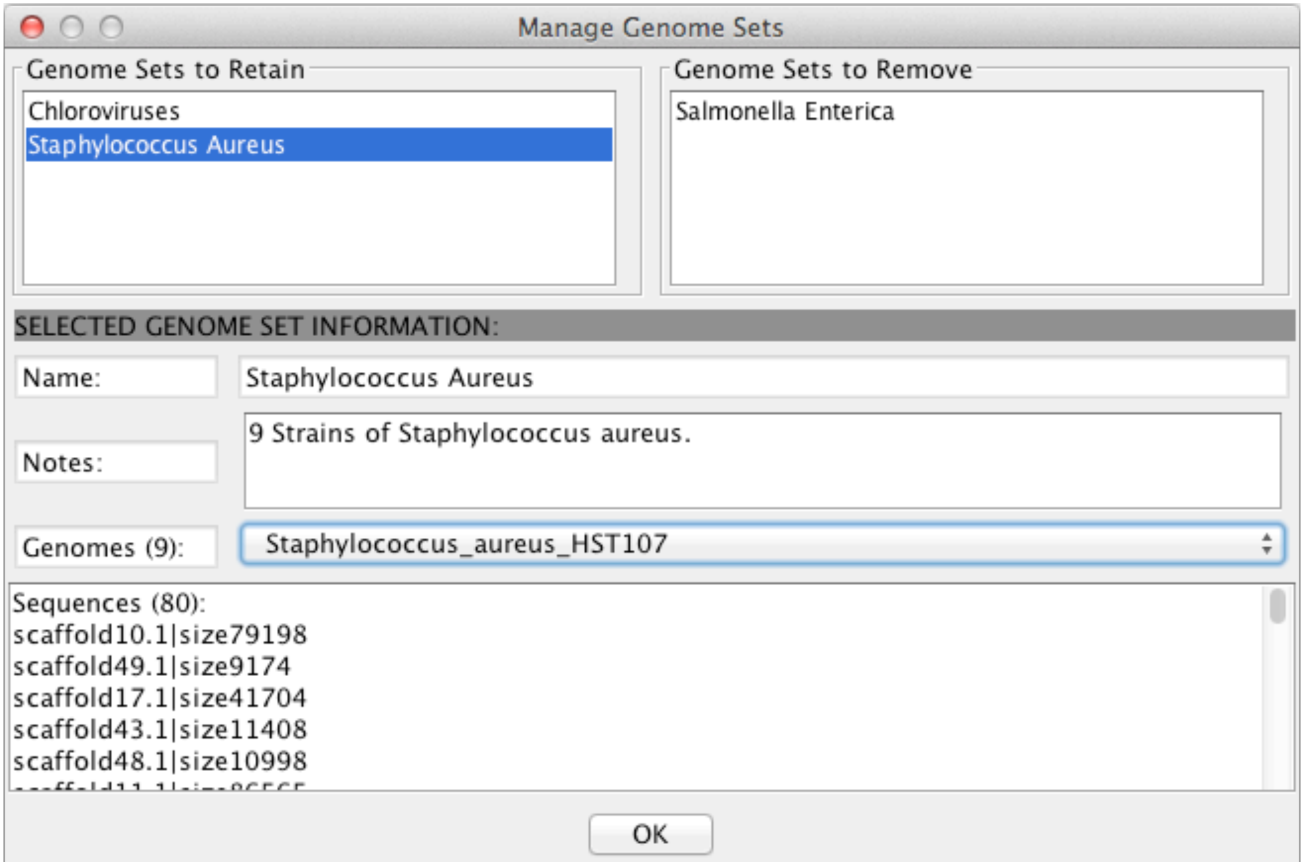

When first launching the frame, all available genome sets will appear in the **Genome Sets to Retain** list panel. Selecting a genome set from this list will cause the information associated with this genome set to appear in the **Selected Genome Set Information Panel**, including the Name, Notes, and a drop-down menu of each genome. Selecting the associated genome from the drop-down displays information about each genome in the scrollable text window below.

To schedule a genome set for removal, **click and drag the set from the Genomes Set to Retain** panel over to the **Genome Sets to Remove** panel. Once you click

the **OK** button, all genome sets in the **Genome Sets to Remove** panel will be removed.

To create new genome sets, close this panel and create a **New Genome Set** (see **New Genome Set** section, page 40).

To remove one or more genomes from an existing genome set, close this panel, switch into the genome set containing genomes you would like to remove (see the **Genome Sets** section, page 42) and view the **Current Genome Set** (see the next section, **Current Genome Set**, page 45).

Current Genome Set (⌘G)

To view information about the genomes in the currently active genome set or remove one or more genomes sets the currently active genome set, select **Current Genome Set** from the **Genomes** drop-down menu, or type ⌘G. The following window will appear:

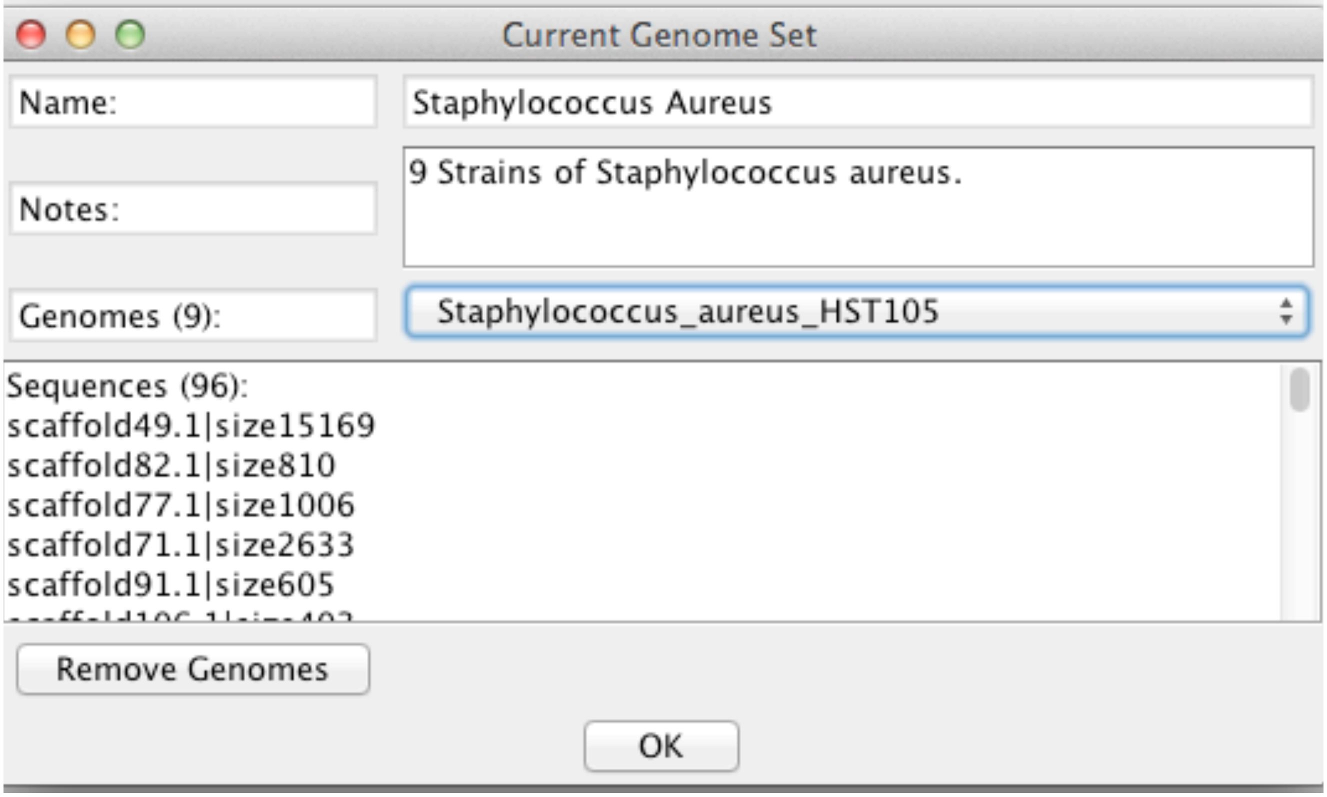

This window provides information about the genomes contained in the current (active) genomic set. Selecting the appropriate genome from the drop-down menu provides more detailed information about that particular genome.

To remove one or more genomes from this genomic working set, click the **Remove Genomes** button. The following window will appear:

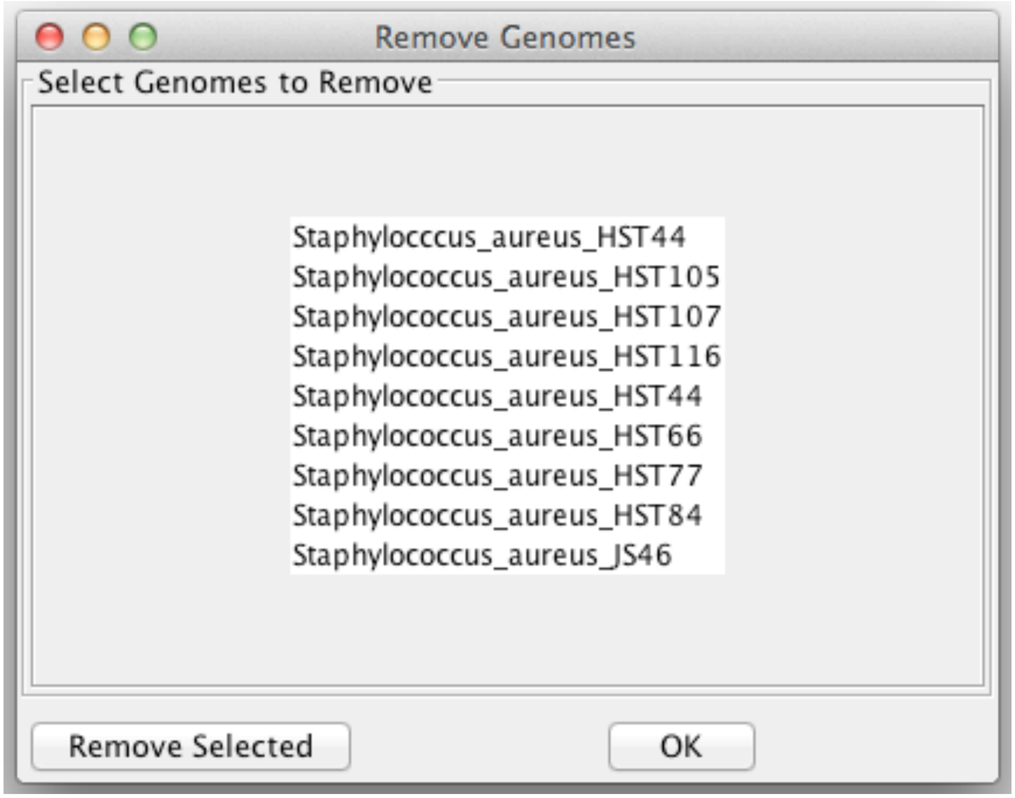

Genomes may be selected using the mouse. Holding down the shift key allows for selection of a range of genomes. Once one or more genomes have been designated for deletion, clicking the **Remove Selected** button deletes these genomes from the genomic working set, and updates the **Current Genome Set** window. Clicking the **OK** button closes the window.

Import Genomes Into Current Genome Set

There are two ways to add genomes to the current genome set: either (1) From file or (2) directly from NCBI's nucleotide database.

Both the standard **.gff (genomic feature format)** as well as some variations of this file format, and the **.gb or .gbk (GenBank file format)** are supported. **Because there are often variations among various GenBank and gff file format types, it is possible to configure file-import options into JContextExplorer** (For more information, please see the **Import Settings** Section, page 52).

When Importing from NCBI, it is possible to stream the information directly into the current genomic working set, or to export this information to file, modify as appropriate, and then re-import into JContextExplorer. **Due to frequent changes in NCBI's file formatting, we recommend importing via files as an alternative to importing directly from NCBI's online database, if possible.** Once again, it is possible to configure database – import options into JContextExplorer. (For more information, please see the **Import Settings** Section, page 52).

This sub-menu is highlighted within the **Genomes Menu**:

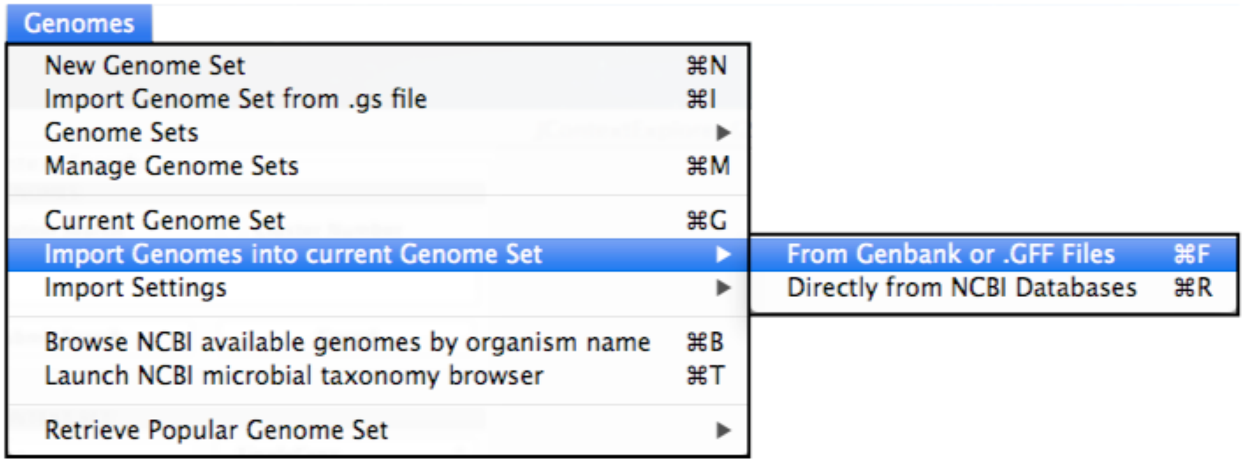

From GenBank or .GFF Files (⌘F)

Selecting this option from the list will bring up a file chooser, which will invite you to select **a directory, a single file, or multiple files**. If you select a directory, JContextExplorer will attempt to import all files in that directory that have an extension of **.gff**, **.gb**, and **.gbk**, creating a genome in each case with the file name prior to the extension. If you select one or more files, JContextExplorer will create a genome for each file (with the genome name as all text prior to the extension). **If a genome already exists of this name, then the information in the new file will be added to the existing genome.** As a good practice, we recommend naming genomes without any white spaces in the name, using underscores instead.

GenBank (designated by **.gbk** or **.gb** extension) and **General feature format** (designated by **.gff** extension) files are standard file formats used in bioinformatics. However, there are occasionally small differences between GenBank and .gff files, depending on their source and date of creation. The specifications necessary for JContextExplorer's file parsers are as follow:

GenBank Files

Contig names are designated by the **LOCUS** keyword. Features start after the **FEATURES** keyword. Each feature starts at the beginning of a line, and information about this feature is indented using various forward-slash **"/"** tags. This information is associated with the feature until the next feature is reached. When a new feature begins, the coordinates are provided between two periods **"<start>..<stop>"**, or **complement(<start>..<stop>)** for the case of features on the reverse strand. If the assembly is incomplete, it is possible that a **join()** tag will designate multiple continuous segments of a single genomic feature.

GFF Files

JContextExplorer may import ordinary GFF files (version 2.5), which contain 9 tab-delimited columns, however will also check for an optional 10th and 11th column. Each line is parsed as a single genomic feature, with all information separated by tabs. Each line is parsed as follows:

column 1: Contig or Sequence Name

column 2: the text string “GenBank” **<constant>**

column 3: Feature type (usually CDS, tRNA, or rRNA)

column 4: Feature start

column 5: Feature stop

column 6: the text string “+” **<constant>**

column 7: Strand (1 designated forward strand, -1 designates reverse strand)

column 8: the text string “.” **<constant>**

column 9: Feature Annotation

These 9 columns make the standard **.gff** file format. An optional 10th and then an optional 11th column may also be included:

column 10: Homology Cluster, if it is assigned **<must be integer>**

column 11: Gene ID, if it is assigned **<any string>**

When importing files into JCE, check carefully for inconsistencies / unusual formatting, especially when importing directly from the NCBI website or importing genomes that have not yet been completely assembled.

DIRECTLY FROM NCBI DATABASES (⌘R)

Genomes may be imported from the national repository NCBI's nucleotide database directly into the JContextExplorer's currently active **Genome Set**, or exported as **GenBank** files (which may then be re-imported to JContextExplorer). To use this feature, select **Directly from NCBI Databases** from the **Import Genomes into current Genome Set** in the **Genomes** drop-down menu, or type ⌘R. The following window will appear:

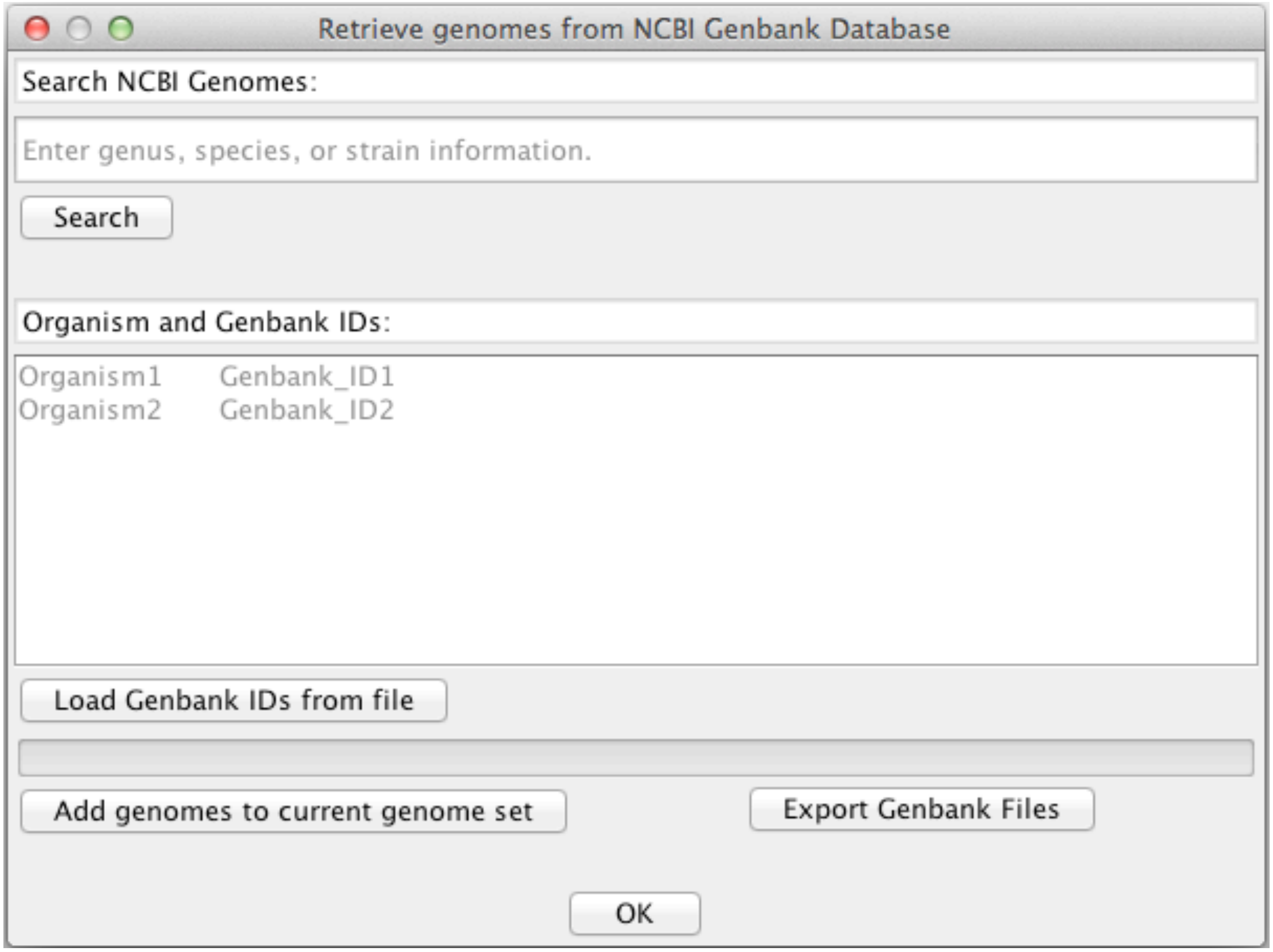

Retrieving information from NCBI's nucleotide database occurs in 2 steps: **(1) Determining available genomes / genome fragments**, and **(2) Importing information associated with one or more specific annotated genomes**.

Searches of NCBI for available genomes / genome fragments are carried out by typing one or more keywords in the **Search Bar**, with keywords separated by

white space. Search results will appear in the text area below. Individual results will be shown as a provisional organism name, followed by a tab, followed by a specific NCBI genome identification number. Text may be modified once it has appeared in this text area.

A list of pre-determined organism names / organism IDs may be imported from a plain text file. This option may be utilized by clicking the **Load GenBank IDs from file** button. Note that pushing this button will simply stream the contents of the selected file into the text area, **and will not check the IDs to see if they are valid NCBI IDs.**

Once this information has been gathered, pushing the **Add genomes to current genome set** button retrieves the whole annotated genome information for each line in the text area – parsing the first entry as organism name, and the second as the ID – formats this information appropriately, and assimilates the data into the **Genome Set**. Pushing the **Export GenBank Files** launches a file chooser that invites you to select a directory. Once a directory has been selected, all GenBank files will be written to plain text files (with the GenBank file extension **.gbk**) into the selected directory. If the process is somehow interrupted or can otherwise not be completed (say, for instances, because of an anomaly in the GenBank file format), a warning message will appear.

Occasionally, server timeout issues can occur which seem to affect directly streaming data into JContextExplorer's current Genome Set, but does not affect streaming into exported files. If streaming the data directly into JContextExplorer does not seem to be working, we recommend streaming the data into files, and then importing these files into JContextExplorer.

Import Settings

Depending on your needs, you may wish to change certain parameters associated with genome import. This may refer to either genome import as from a **GenBank** or **.gff** file, or from the NCBI's nucleotide database. When JContextExplorer is launched, certain default settings take effect, however changing values in the appropriate menu may change all these settings. Changes made in these menus will be stored for the remainder of the session, however **when JContextExplorer is closed and re-launched, the settings will revert to the defaults.**

This sub-menu is highlighted within the **Genomes Menu**:

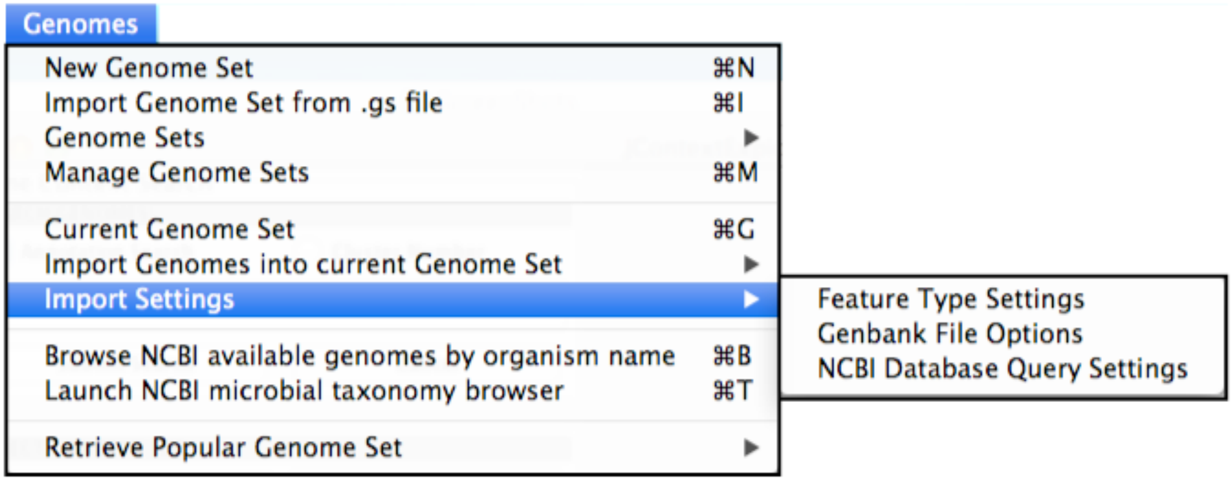

FEATURE TYPE SETTINGS

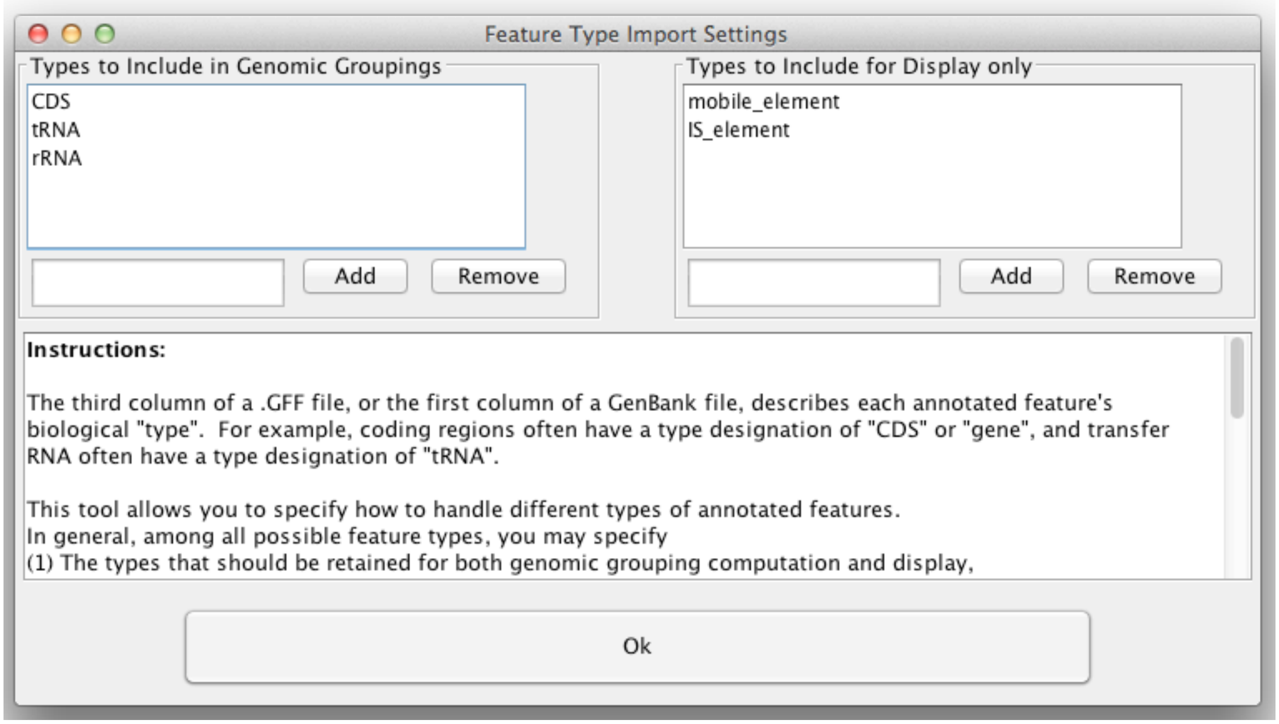

The third column of a **.gff** file, or the first column of a **GenBank** file, describes each annotated feature's biological "type". For example, coding regions often have a type designation of "CDS" or "gene", and transfer RNA often have a type designation of "tRNA".

This window allows you to specify how to handle different types of annotated features.

In general, among all possible feature types, you may specify

(1) The types that should be retained for both genomic grouping computation and display,

(2) The types that should be excluded from genomic grouping computation, but retained for display, and

(3) The types that should be excluded altogether.

Types in the list **Types to Include in Genomic Groupings** (left) will be retained for both genomic grouping computation and display. Types in the list **Types to Include for Display only** (right) will be retained for display only when viewing genomic segments. All other types will be ignored (excluded altogether).

To add types to a list, type in the type in the text field below the list and push the **Add** button.

To remove types from a list, select the type with your mouse, and push the **Remove** button.

To transfer types from one list to another, select the type with your mouse, and drag the type to the other list.

WARNING!

Features in a **.gff** or **GenBank** file may not overlap in the genomic coordinates they span. In the case that they do overlap, JContextExplorer will exhibit unpredictable behavior and likely fail.

Please ensure that no annotated features overlap prior to loading **.gff** or **GenBank** files.

GENBANK FILE OPTIONS

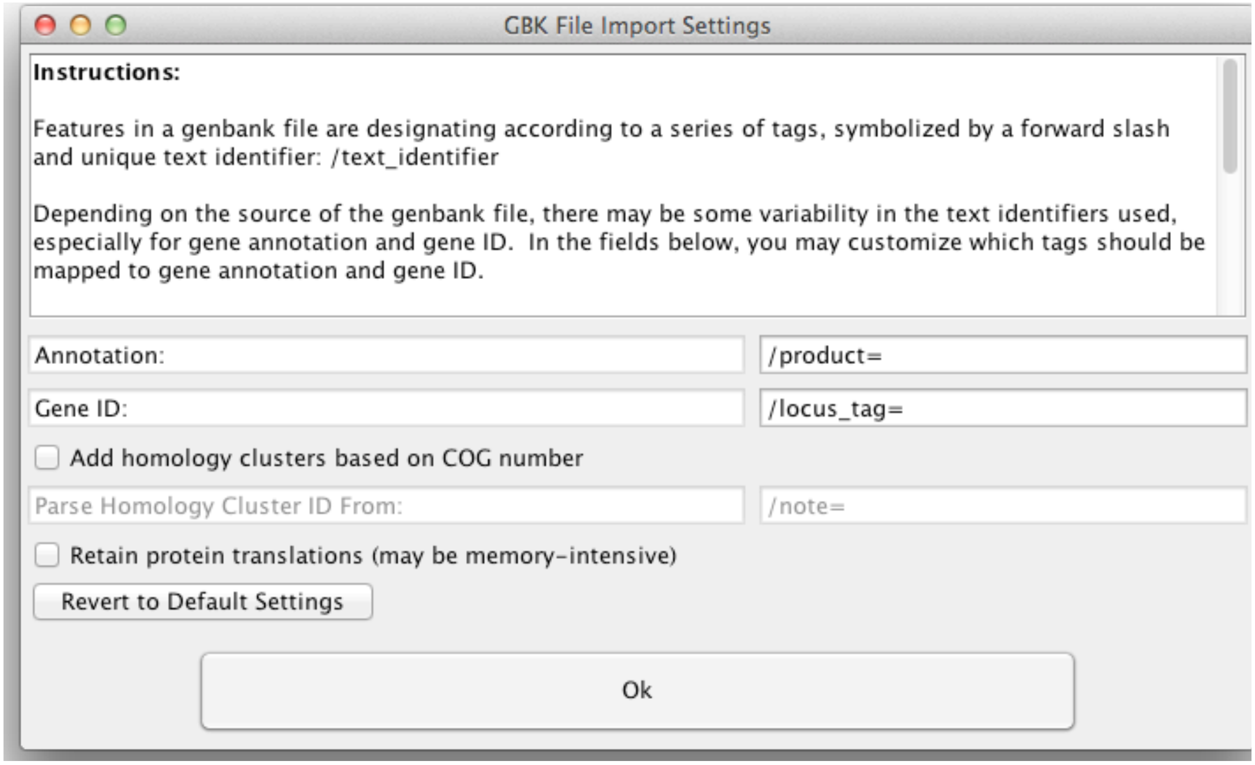

Features in a **GenBank** file are designating according to a series of tags, symbolized by a forward slash and unique text identifier: **/text_identifier**

Depending on the source of the **GenBank** file, there may be some variability in the text identifiers used, especially for gene annotation and gene ID. In the fields below, you may customize which tags should be mapped to gene annotation and gene ID.

Occasionally, **GenBank** files may have homology clusters designated, in the form of COG groupings, or an alternative standard homology cluster ID designation. It is possible to attempt to assign homology cluster IDs from a specified tag.

GenBank files contain the protein translation information for all protein-coding genes. You may retain this information if you check the appropriate box. However, be warned that this may be very memory-intensive, especially if your genomic set contains a large number of genomes.

NCBI DATABASE QUERY SETTINGS

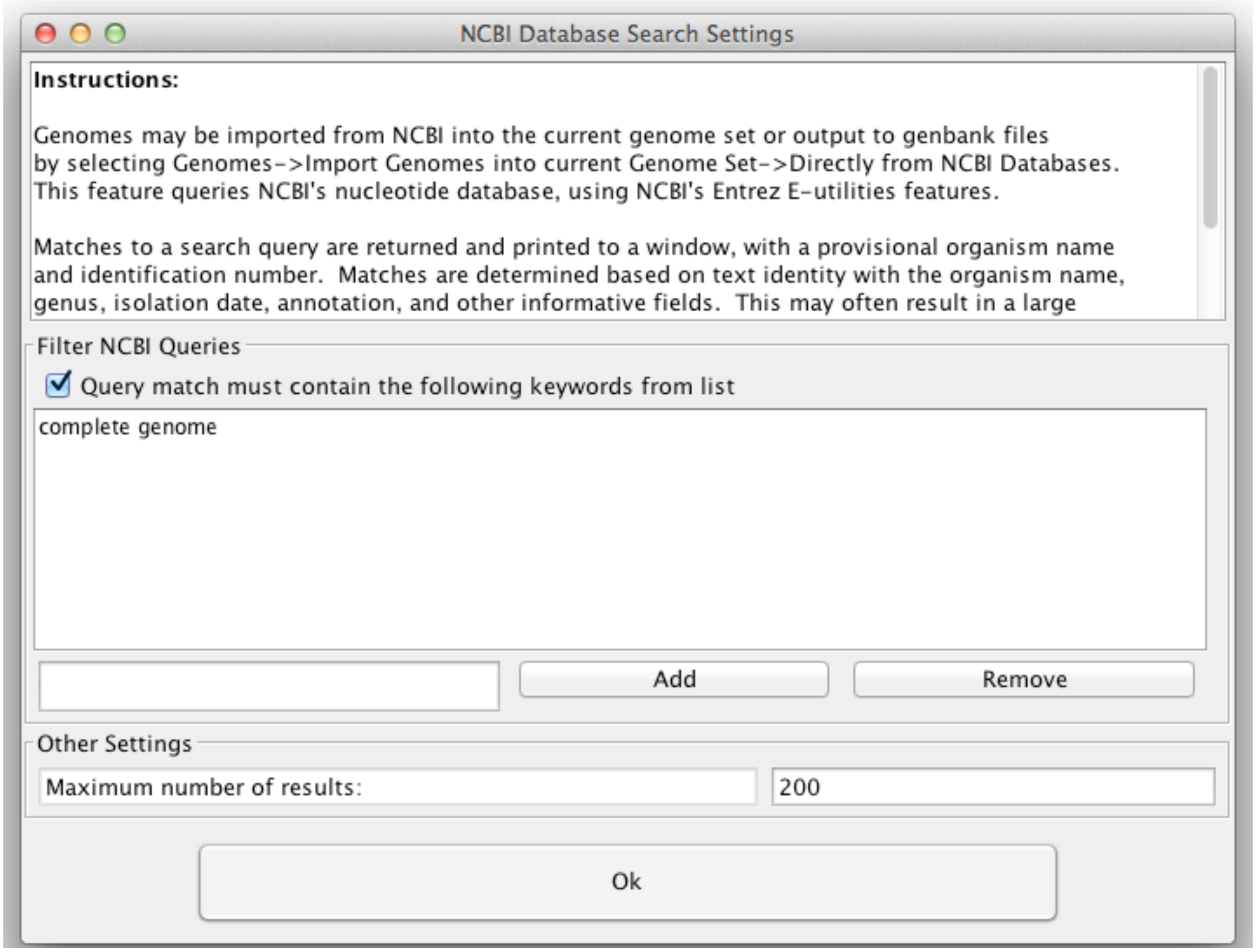

Genomes may be imported from NCBI into the current genome set or output to **GenBank** files by selecting **Genomes -> Import Genomes into current Genome Set -> Directly from NCBI Databases.** This feature queries NCBI's nucleotide database, using NCBI's Entrez E-utilities features.

Matches to a search query are returned and printed to a window, with a provisional organism name and identification number. Matches are determined based on text identity with the organism name, genus, isolation date, annotation, and other informative fields. This may often result in a large number of matches, so additional filters in the organism name may be specified below to reduce the total number of matches. It is also possible to modify the total number of search results returned. All NCBI queries and result filters are case-insensitive.

The **Maximum number of results** field may not be larger than 100,000 (values larger than 100,000 will be automatically truncated at 100,000) and refers to the number of hits **pre-filtering**. The terms in the list serve as an **AND** filter- organism names that contain **all** of the terms in the list are retained. Selecting an entry from the list (with mouse click) and pushing the **Remove** button will remove this query from the list, while typing in a new query and pushing the **Add** button adds the query to the list.

BROWSE NCBI AVAILABLE GENOMES BY ORGANISM NAME (⌘B)

JContextExplorer provides functionality to search / retrieve particular genomes internally, using the **Directly from NCBI Databases** search tool (see page 50). However, it may be easier to browse NCBI - available genomes in a standard Internet browser to determine which genomes should be included in a JContextExplorer analysis.

NCBI's master genome-browse page may be accessed directly from within JContextExplorer by selecting this option from the **Genomes** menu, or typing the ⌘B shortcut. This will open the website

http://www.ncbi.nlm.nih.gov/nuccore in your default Internet browser, which will look something like this:

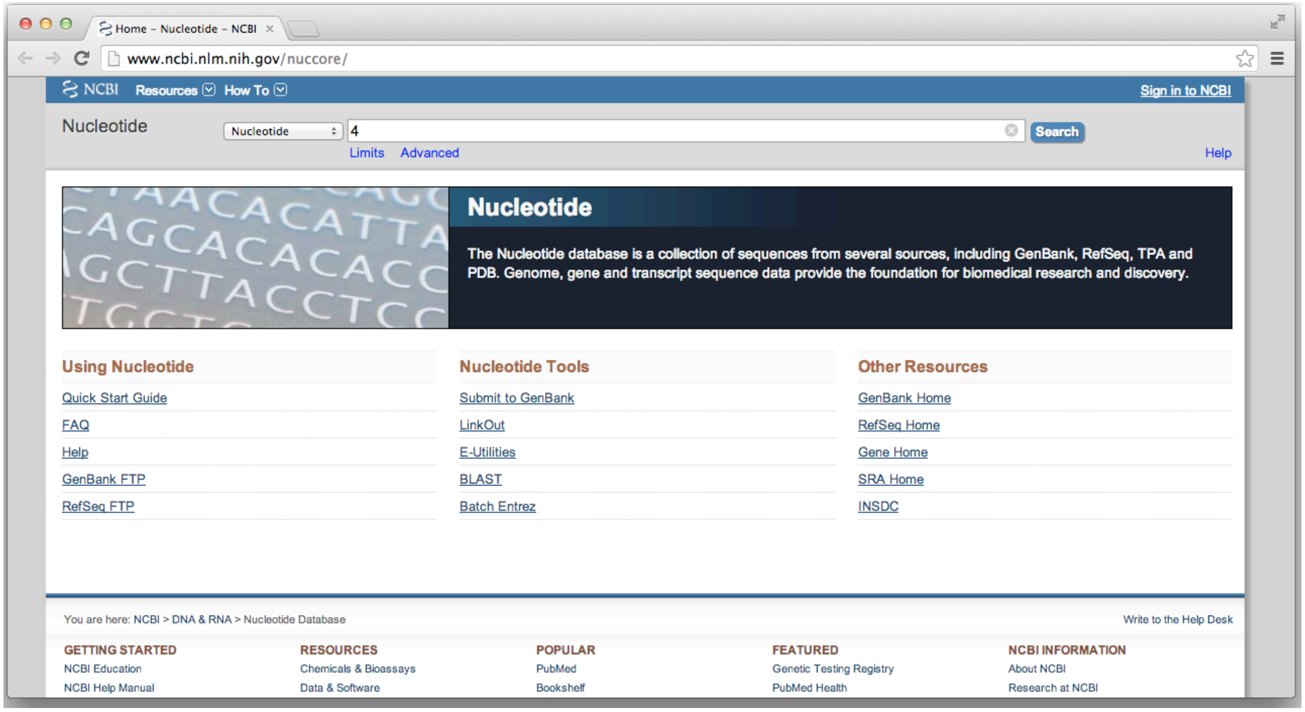

LAUNCH NCBI MICROBIAL TAXONOMY BROWSER (⌘T)

JContextExplorer provides functionality to search / retrieve particular genomes internally, using the **Directly from NCBI Databases** search tool (see page 50). However, it may be easier to browse NCBI - available genomes in a standard Internet browser to determine which genomes should be included in a JContextExplorer analysis.

NCBI implements a taxonomy tree of available microbial genomes – this way, organisms are organized based on their evolutionary relatedness. Information may be gathered about respective genomes using button clicks. This taxonomy browser will be launched in your default Internet browser by selecting this option from the **Genomes** menu, or by typing ⌘T. The website URL is

http://www.ncbi.nlm.nih.gov/genomes/MICROBES/microbial_taxtree.html

When launched, the window should look something like this:

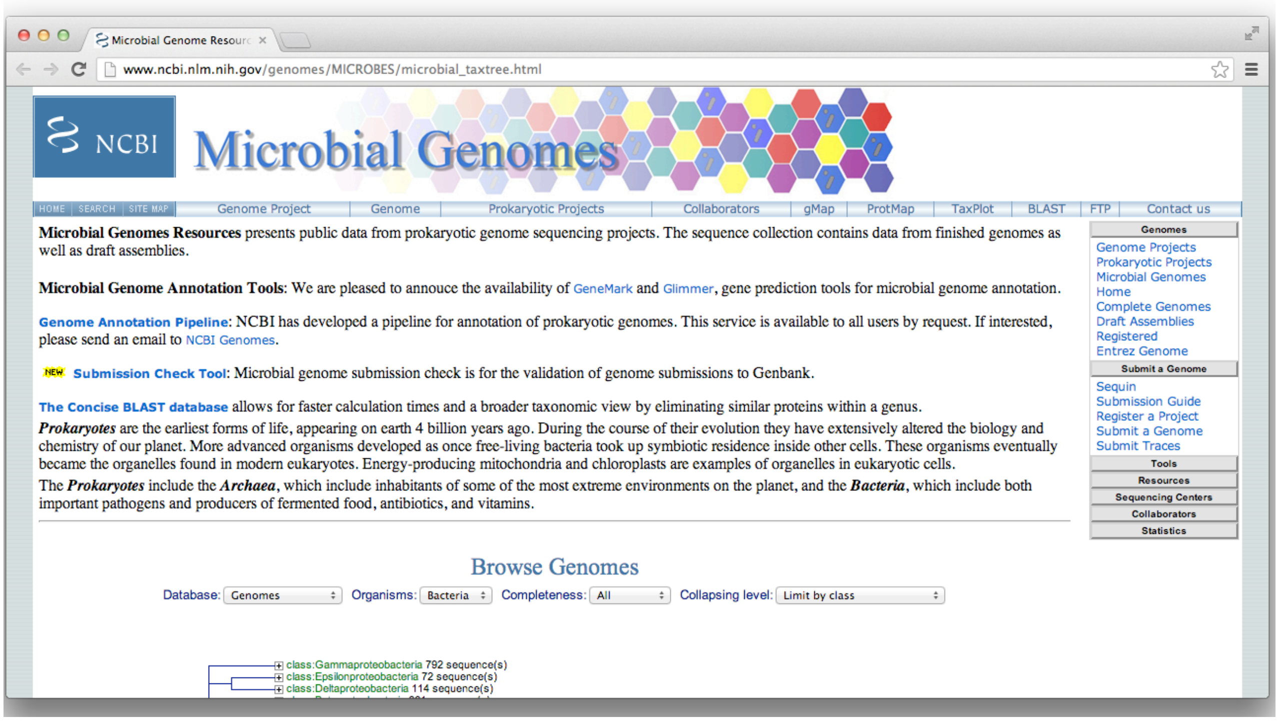

RETRIEVE POPULAR GENOME SET

Certain **Genome Sets** are used frequently by JContextExplorer creators and collaborators. These datasets may be loaded into JContextExplorer simply by selecting the appropriate genome set from the **Retrieve Popular Genome Set** menu:

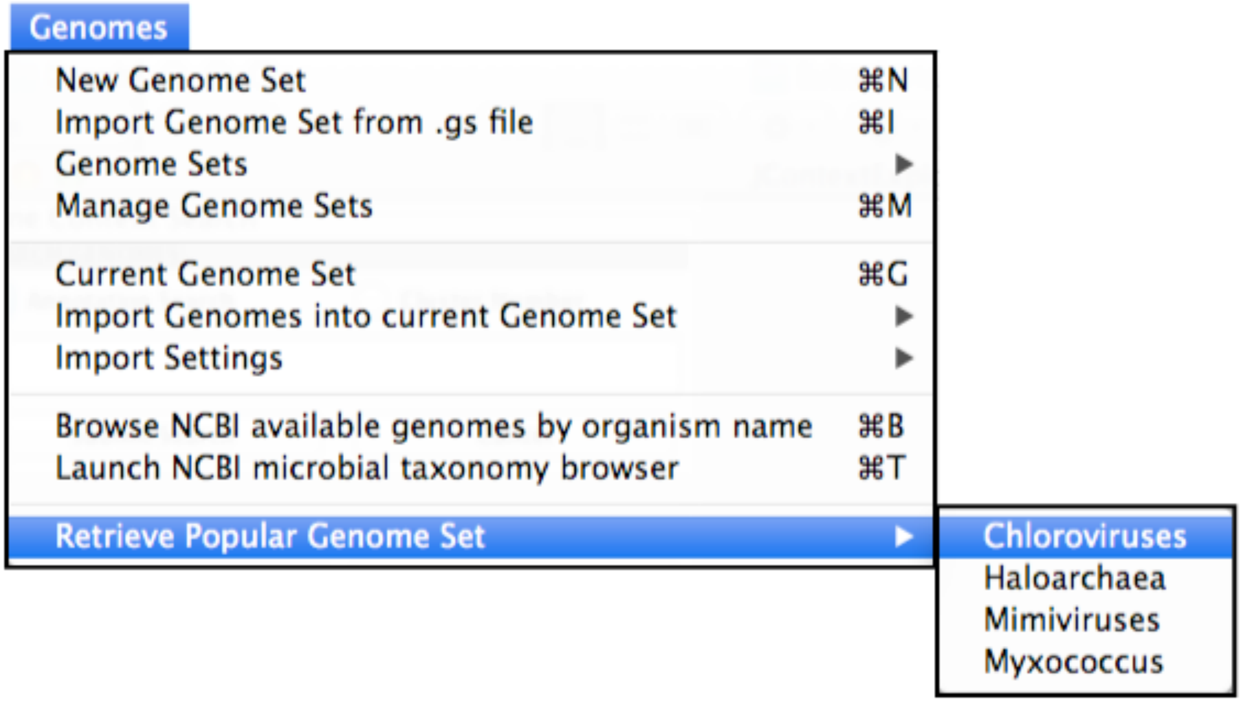

To request that a genome set be added to the **Retrieve Popular Genome Set** menu, please contact **Phillip Seitzer** at pmseitzer@ucdavis.edu

Genome Sets may be password protected! Selecting a password-protected genome set will display a dialog box asking for the password. This option is available for requested popular sets.

Available Sets

Chloroviruses

A set of 41 large, double-stranded DNA viruses known to infect various species of algae. This set includes viruses taken from 1 of 3 different hosts. This genome set has been under investigation for the past several years by the Dunigan laboratory at the Nebraska center for virology: http://www.unl.edu/virologycenter/david-d-dunigan-ph-d

Haloarchaea

The Haloarchaea genome set consists of 80 Achaeal halophiles, all closely related to each other. This genome set has been under investigation for the past several years by the Facciotti and Eisen Labs at UC Davis:

Facciotti lab: http://www.bme.ucdavis.edu/facciotti/

Eisen lab: http://phylogenomics.wordpress.com/

This set includes a phylogenetic tree, and several sample context sets.

Mimiviruses

A set of 13 recently discovered + sequenced very large viruses.

Myxococcus

A set of 5 bacterial species, 3 Myxococcus and 2 highly related. Includes the model organism *Myxococcus xanthus*.

Load Menu

Once a set of genomes has been properly imported into JContextExplorer, Additional information may be loaded in to aid analysis of the genomes. This refers to categorical-type information (such as **Homology Clusters** and **Gene IDs**), externally computed biological information (such as **Phylogenetic Trees** and pre-determined protein binding site **Sequence Motifs**) and JContextExplorer-specific analysis information (**Context Sets, Dissimilarity Measure**).

Without loading in additional information, JContextExplorer is little more than a genome feature search tool. To use JContextExplorer to its full potential as a tool to **quantitatively** interrogate genomic context, Load-menu options should be utilized. We emphasize the JContextExplorer-specific analyses (**Context Sets, Dissimilarity Measure**) as a useful starting point. These tools lay the groundwork for the operations of context tree generation. To create context trees more finely tuned to specific needs, additional biological information may be used to inform context tree construction. Finally **Gene IDs** and **Homology Clusters** are indispensable for efficient navigation of genome sets.

The Load Menu may be selected from the main menu bar, and when expanded looks like this:

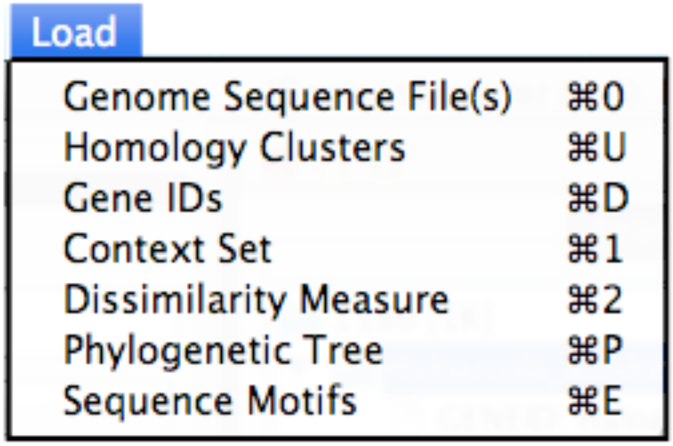

What follows is a detailed description of each menu item.

Genome Sequence File(s) (⌘0)

When JContextExplorer launches, genome annotation information is loaded into memory, and may be accessed at any time. The sequences underlying this information are not readily available, however, they may still be retrieved, if desired. To make this information retrievable, it is necessary to associate a .fasta genome file with each organism where genome information should be retrieved.

Each genome file should have the same name<.fasta> as the organism it is associated with. This will ensure that data is correctly associated. Additionally, **If the genome exists in multiple contigs, each header in the .fasta genome file should be named according to the contig to which it is associated**. No internal checks are carried out to see that the data is "correct" (in that the right genome is associated with the right organism), it is up to the user to name files apropriately.

If a single directory contains .fasta genome files for multiple genomes, **this directory may be selected, and all genomes will be associated appropriately**.

Sequence information is retrievable through **search results frames**. Please see **Export Options: Search Results Frame Menu Options** (page 24) for more information.

HOMOLOGY CLUSTERS (⌘U)

Within a single genomic working set, certain annotated features may be homologous to one another. This may occur both within a single species and across multiple species. A group of homologous features is often referred to as a **Homology Cluster**. Numerous methods exist to detect homology across and within genomes, and to cluster annotated features in a set of genomes into homology cluster groups. Often, but not necessarily, these homology cluster groups are non-overlapping. That is, each annotated feature may belong to a maximum of one homology cluster.

For all homology cluster-associated processes, JContextExplorer assumes non-overlapping homology clusters.

When JContextExplorer searches for annotated features in a genomic working set, it may do so either by (1) Matching a textual query to individual genomic feature annotations or (2) Matching a **Homology Cluster** ID number.

Textual annotations may be unreliable (especially if a genomic working set contains genomes annotated by different groups), so it may be worthwhile to compute homology clusters and load these computed homology clusters into JContextExplorer.

WARNING!

JContextExplorer cannot compute homology clusters from a set of sequenced genomes, only search a set of pre-computed, loaded homology clusters.

Selecting the **Homology Clusters** option from the Load menu, or typing ⌘U, will launch a file chooser, inviting you to supply a file containing homology clusters. Please separate your data using new line characters for each line, and tabs between individual entries. **Each line in the file will be parsed in a different way, depending on the number of tab-delimited entries in that line.**

(1) 5 tab-delimited entries in line:

If there are 5 tab-delimited entries in the line, entries take on the following values:

Column 1: Genome Name

Column 2: Sequence Name

Column 3: Feature Start Position

Column 4: Feature End Position

Column 5: Homology Cluster ID Number

If a feature starts at **Feature Start Position** and stops at **Feature Stop Position**, on the sequence named **Sequence Name**, in the genome named **Genome Name**, this feature is assigned the provided **Homology Cluster ID Number**.

(2) 4 tab-delimited entries in line:

If there are 4 tab-delimited entries in the line, entries take on the following values:

Column 1: Genome Name

Column 2: Sequence Name

Column 3: Annotation Key

Column 4: Homology Cluster ID Number

If a feature contains the string **Annotation Key** in it's annotation, and is found on the sequence named **Sequence Name** in the genome named **Genome Name**, this feature is assigned the provided **Homology Cluster ID Number**.

In the Annotation Key field, please use underscores instead of spaces.

(3) 3 tab-delimited entries in line:

If there are 3 tab-delimited entries in the line, entries take on the following values:

Column 1: Genome Name

Column 2: Annotation Key

Column 3: Homology Cluster ID Number

This format is identical to Four-column format, however does not check for agreement in the sequence name.

(4) 2 tab-delimited entries in line:

If there are 2 tab-delimited entries in the line, entries take on the following values:

Column 1: Annotation Key

Column 3: Homology Cluster ID Number

All features in all genomes in the genomic working set with an annotation that contains the **Annotation Key** are assigned the provided   Please use underscores instead of spaces.

(5) Single Column Format

If there is only a single entry in the line, this entry is taken to be the **Annotation Key**. All annotated features that contain the annotation key are given a homology cluster ID number, which is determined by the line number in the file. Please use underscores instead of spaces.

Gene IDs (⌘D)

If you expect that multiple genomic features will contain identical homology cluster IDs or annotations, then it may be helpful to have a unique textual identifier **specific to a single genomic feature in a single organism.** Here, we define this as a **Gene ID**, however it is sometimes also called a **Locus Tag**.

In the main search window, it is possible to search for genomic features based on one or more Gene IDs instead of annotation text strings. In this case, however, **an exact match is required**. Annotation-based searches require only a partial match. **Please remember to have the "Annotation Search" radio button selected to retrieve a particular Gene ID.**

To load a set of **Gene IDs**, please select the **Gene ID** option from the Load menu, or type ⌘D. This will launch a file chooser that will invite you to select a Gene ID file. **Files should be formatted as 5-column tab-delimited files with columns as follows:**

Column 1: Organism Name

Column 2: Sequence Name

Column 3: Genomic Feature Start Position

Column 4: Genomic Feature Stop Position

Column 5: Unique Gene ID

A genomic feature from the organism **Organism Name**, on the sequence **Sequence Name**, with coordinates from **Genomic Feature Start Position** to **Genomic Feature Stop Position** will be assigned the **Unique Gene ID**.

Each feature may have a maximum of **one** gene ID. Setting a gene ID to a particular genomic feature overwrites a previous gene ID.

CONTEXT SET (⌘1)

When conducting a search for a particular gene or genes in the main frame, you may be interested in not just the genes themselves, **but some additional context** – perhaps a local region around each gene, perhaps all copies of the gene within a single organism, perhaps the gene itself and the next 3 downstream genes.

A **Context Set** is a definition for such an additional description. Every search performed on a genome set returns not just genomic features that match the query, but **also all additional required genomic features that meet the requirements specified by the Context Set.**

JContextExplorer offers a wide variety of types of Context Sets. A detailed description of the different types of context sets offered can be found in the **Available Context Set Types** section (page 71).

To launch this window, either select **Context Set** from the **Load** menu, type ⌘1, or push the **Add/Remove** button in the **Genome Set Search Area** of the main window:

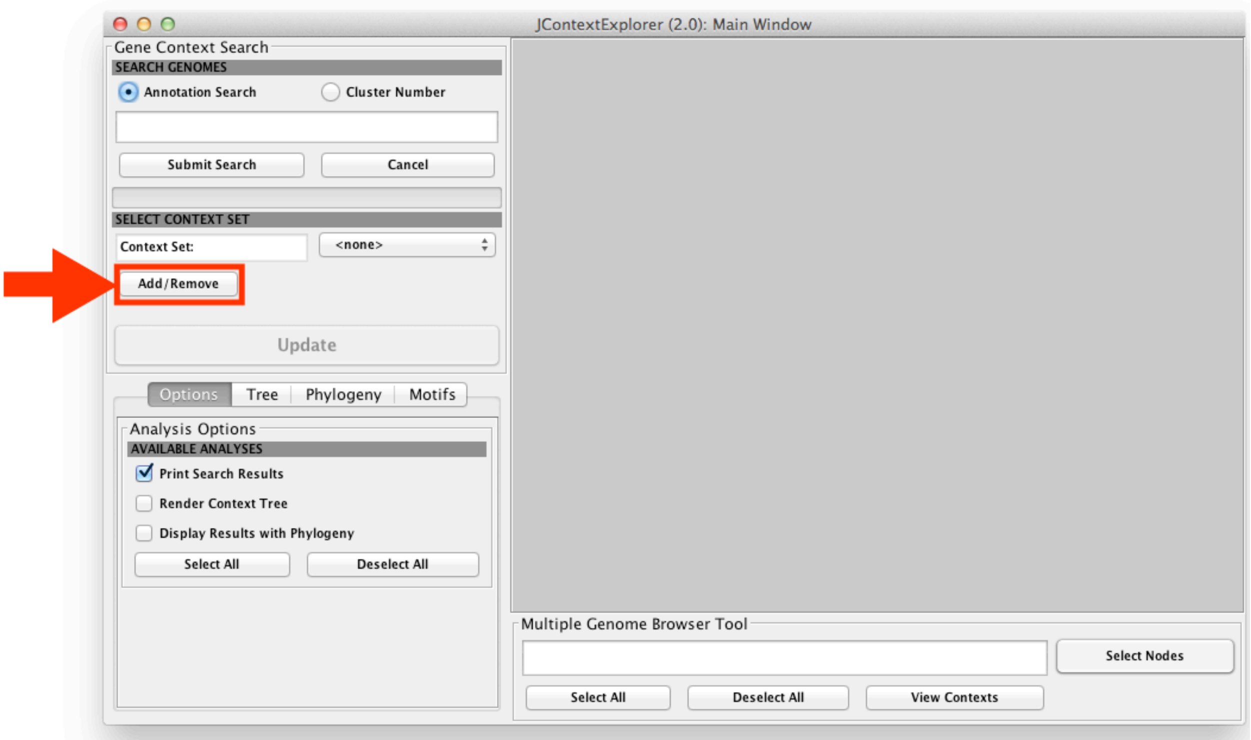

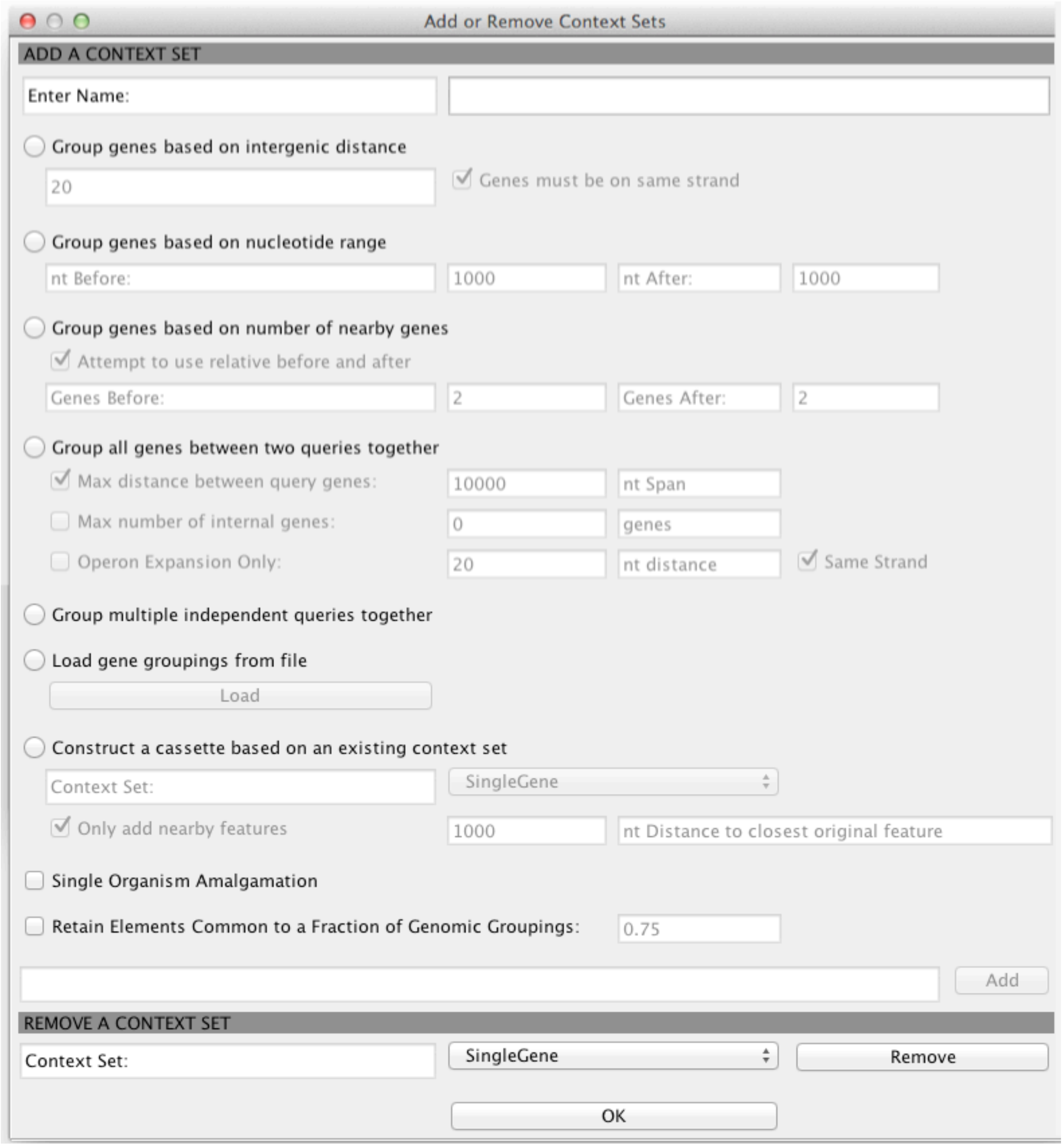

Every Context Set requires a unique name. Please enter a unique name in text field to the right of the **"Enter Name:"** label.

The first section is dedicated towards creating and adding a new context set. This section is marked with a banner with the text "**Add a Context Set.**"

Define the type of Context Set you would like to create by selecting one of the 7 radio buttons below the banner. A description of each of these methods is described in detail in the next section. Upon selecting a radio button, associated parameters with that type of Context Set will light up, with default values included.

Note the ‘Single Organism Amalgamation’ check box and ‘Retain Elements Common to a Fraction of Genomic Groupings’ check box following the 7 radio buttons. These checkboxes are explained in more detail below, in the section **Context Set Filter Types** (page 75).

Finally, once you have selected the type of context set you would like to create and adjusted all appropriate parameters, **click the “Add” button in the lower right hand corner of the screen. If you do not click this button, the set will not be added.**

To the right of the **Add button** is a white text bar, which will provide information about the individual context sets, and will inform you when you have successfully added a context set.

Below this is a banner designating a new section, for removing context sets. This section is marked by the label **‘Remove A Context Set’**.

To remove an existing context set, select the name of that context set from the drop-down menu and click the **Remove button**.

To conclude all processes, click the **OK** button to close the frame.

Please remember: **You must click the Add button to add the context set. If you specify the set you would like and click the OK button at the bottom of the screen without first clicking the ‘Add’ button, the context set will not be added.**

Available Context Set Types

There are 7 types of Context Sets available; one of which is implicit and created by default with every genomic set (**SingleGene**), the others must be made after an organism set has been defined and genomes loaded.

If additional genomes data is loaded following the definition of a context set, the old context set will still be applicable to the new genomes data.

(1) SingleGene

The 'SingleGene' context set returns only the single annotated gene match to the query.

(2) Group genes based on intergenic distance

Annotated features are organized into non-overlapping groups based on (1) intergenic distance and (possibly) (2) strandedness. The **intergenic distance threshold** field allows the user to specify a cutoff point for grouping annotated features into the same genomic grouping. **If the end (stop position) of one annotated feature is within the threshold distance from the start (start position) of the next annotated feature, these annotated features will be grouped into the same genomic grouping.** If the "Genes must be on same strand" checkbox is checked, then the genes must also be on the same strand.

This approach is analogous to a purely distance-based operon prediction algorithm.

(3) Group genes based on nucleotide range

Genomic groupings are determined by including all annotated features where the **distance between the centers** of the annotated feature and query match is less than or equal to the user-provided range values. The range of values around query matches to take may be edited in the **nt Before** and **nt After** text fields.

(4) Group genes based on number of nearby genes

Genomic Groupings are determined by taking some number of annotated features both before and/or after all query matches. The number of features to include may be edited in the **Genes Before** and **Genes After** text fields.

Checking the **Attempt to use relative before and after** attempts to correct for possible cross-species Strand changes, and selecting the genomic groupings that make sense despite possible Strand changes.
For example, if 90% of the query match is on the positive strand and 10% is on the negative strand, and all nearby genes are otherwise identical around these genes, then the 10% with the Strand change will be normalized to the other 90% (to ensure consistent output).

(5) Group all genes between two queries together

All annotated features between and including two query matches are included into genomic groupings. **This genomic grouping requires that exactly two queries be provided**. Failure to do so will result in an error message.

In the case that multiple instances of individual queries exist, then **the closest pairs of queries will match together.** This does not guarantee that the matches will all be nearby: Every instance of each individual query will be matched. **If a nearby matching option does not exist, the query will be matched with a gene that is not nearby.**

However, it is possible to specify that matches must be nearby: Checking the **Max distance between query genes** will retain only the genomic groupings where the centers of the individual query matches are within the **specified nucleotide span** (text field with default value of 10000 nucleotides).

It is also possible to specify and upper limit on the number of genes that may exist between the two query matches with the **Max number of internal genes** checkbox. Only matches that have fewer than or equal to the number of genes between the two "between" queries will be retained.

A special third checkbox option exists: **Operon Expansion Only**. Checking this box will return only those query matches that potentially represent an "operon", as predicted by intergenic distance and possibly strandedness. Specifically, all adajcent genes identified in a between context must have intergenic distances no larger than the threshold specified, and if the same strand checkbox is checked, they must all be on the same strand, for the genomic grouping to be retained.

(6) Group multiple independent queries together

All individual gene query matches within the same organism are amalgamated into the same context set. This is identical to a **SingleGene** context set with the **Single Organism Amalgamation** checkbox checked.

(7) Load gene groupings from file

It is possible to determine a context set ahead of time and load the resulting genomic groupings in via one or a series of tab-delimited files. This may be useful for cases where it may not be possible to use any of the other pre-defined context set operations to group genes appropriately.

(A) single file approach

A single file contains the mapping of individual genes into non-overlapping genomic groupings. Each line in the fille should be formatted as follows:

Column 1: Organism Name

Column 2: Sequence name

Column 3: Annotated feature start position

Column 4: annotated feature stop position

Column 5: context set ID number (any natural number is okay). genomic features with a common context set ID number will be grouped into the same genomic grouping.

(B) Set of files approach

First, a **Context Set Mapping File** should be created, formatted as a two-column tab-delimited file which should contain, in **column 1**, the name of the organism, and in **column 2**, the full path to another file (an individual **context file**).

Next, individual context files should be created for each organism. An individual **context file** should be created for each organism of interest. Each file should be a 4-column tab-delimited file, with the following information in each column:

Column 1: Sequence name

Column 2: Annotated feature start position

Column 3: annotated feature stop position

Column 4: context set ID number (any natural number is okay).

Each individual **context file** should be named the name of the organism with a **.txt** extension.

(8) Construct a cassette based on an existing context set

A **cassette** is an extension of an existing context set, and works in the following way:

(1) A search is undertaken with the original context set. All feature query matches are collected into a list.

(2) A **MultipleQuery** search is undertaken using all features in the list.

Cassette type approaches are useful in tracking the positions of genes that may be close in some organisms but have moved far away in others.

Note the checkbox below this option titled **Only add nearby features**. This provides that features that are very far away from one of the original matched genes are not retained in the final genomic grouping.

Context Set Filter Types

Several modifications are available to affect each of the above basic Context Set generation algorithms.

(1) Single Organism Amalgamation

All genomic groupings that derive from the same organism are amalgamated into a single genomic grouping. Each search, therefore, produces a maximum of one genomic grouping per organism.

Additionally, if no genomic groupings are discovered for a given organism, an empty set is produced for that organism, and used in all context tree computations, and displayed in the search results window.

(2) Retain Elements Common to a Fraction of Genomic Groupings

Following determination of genomic groupings, this modifier will remove elements that are present in fewer than a provided fraction of the total genomic groupings. Elements are defined either by common annotation or common cluster ID, depending on the search type.

This filtering step is enacted **before** cassettes are evaluted from an existing context set – so the order of processing is (1) Context Set determination, (2) Filtering based on common fraction of genomic groupings, and finally (3) computation of a cassette from the genes that remain in the post-filtered genomic set.

DISSIMILARITY MEASURE (⌘2)

Using the Context Set feature (as described in the previous section), a search query returns a set of genomic groupings. In order to assemble these genomic groupings into a **Context Tree**, the groupings must be quantitatively compared. JContextExplorer uses **variable-group agglomerative hierarchical clustering**, a generalization of hierarchical clustering. For more information on the details of variable group agglomerative hierarchical clustering as compared to regular hierarchical clustering, please see

Gomez, S., Fernandez, A., Montiel, J., & Torres, D. (n.d.). Solving Non-Uniqueness in Agglomerative Hierarchical Clustering Using Multidendrograms. *Journal of Classification*, *65*, 43-65

Hierarchical clustering works by (1) first comparing every object with every other object, and then (2) progressively grouping the objects into larger and larger groups, based in some way on the individual object to object comparisons. The **Dissimilarity Measure** is the technique used to compare individual objects.

In the context of JContextExplorer, the objects are genomic groupings, and the **Dissimilarity Measure** is the algorithm chosen to quantitatively compare these genomic groupings. JContextExplorer has several built-in dissimilarity measures, as well as functionality to define customized dissimilarity measures.

To launch this window, either select **Dissimilarity Measure** from the **Load** menu, type ⌘2, or push the **Add/Remove** button in the **Tree** sub-panel in the **Search Options Area** of the main window:

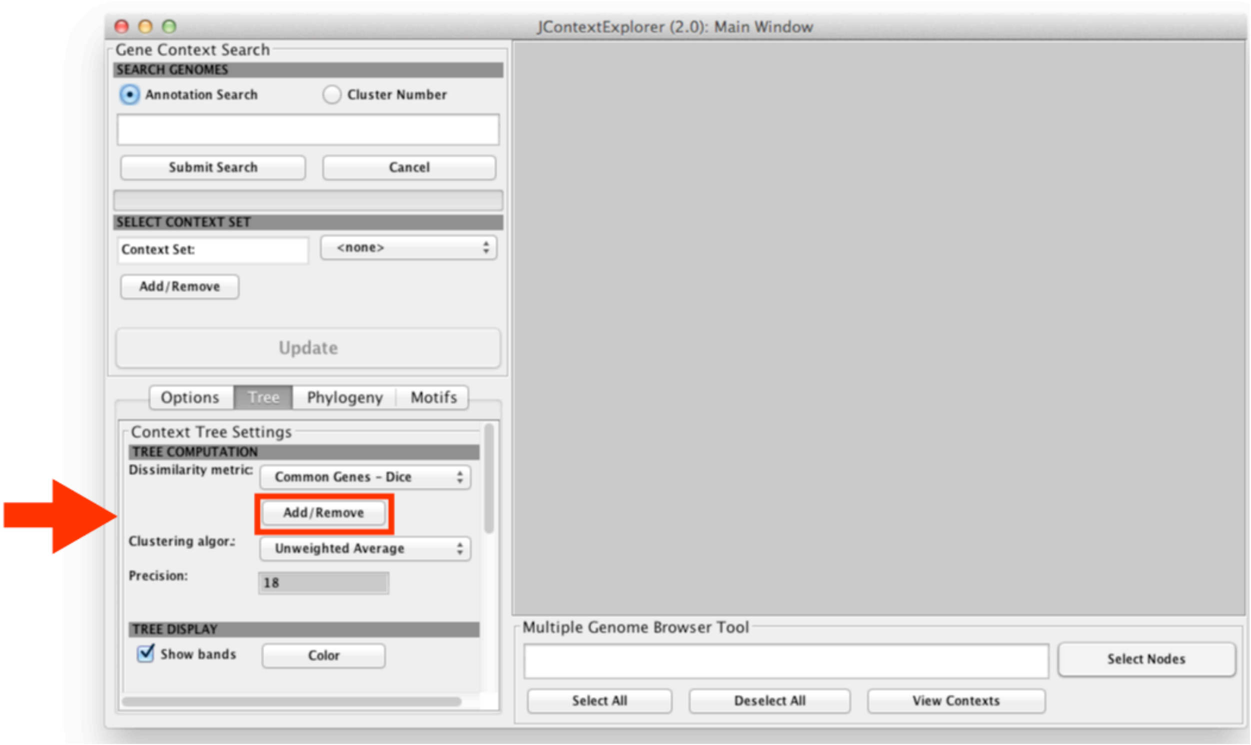

The following window will appear:

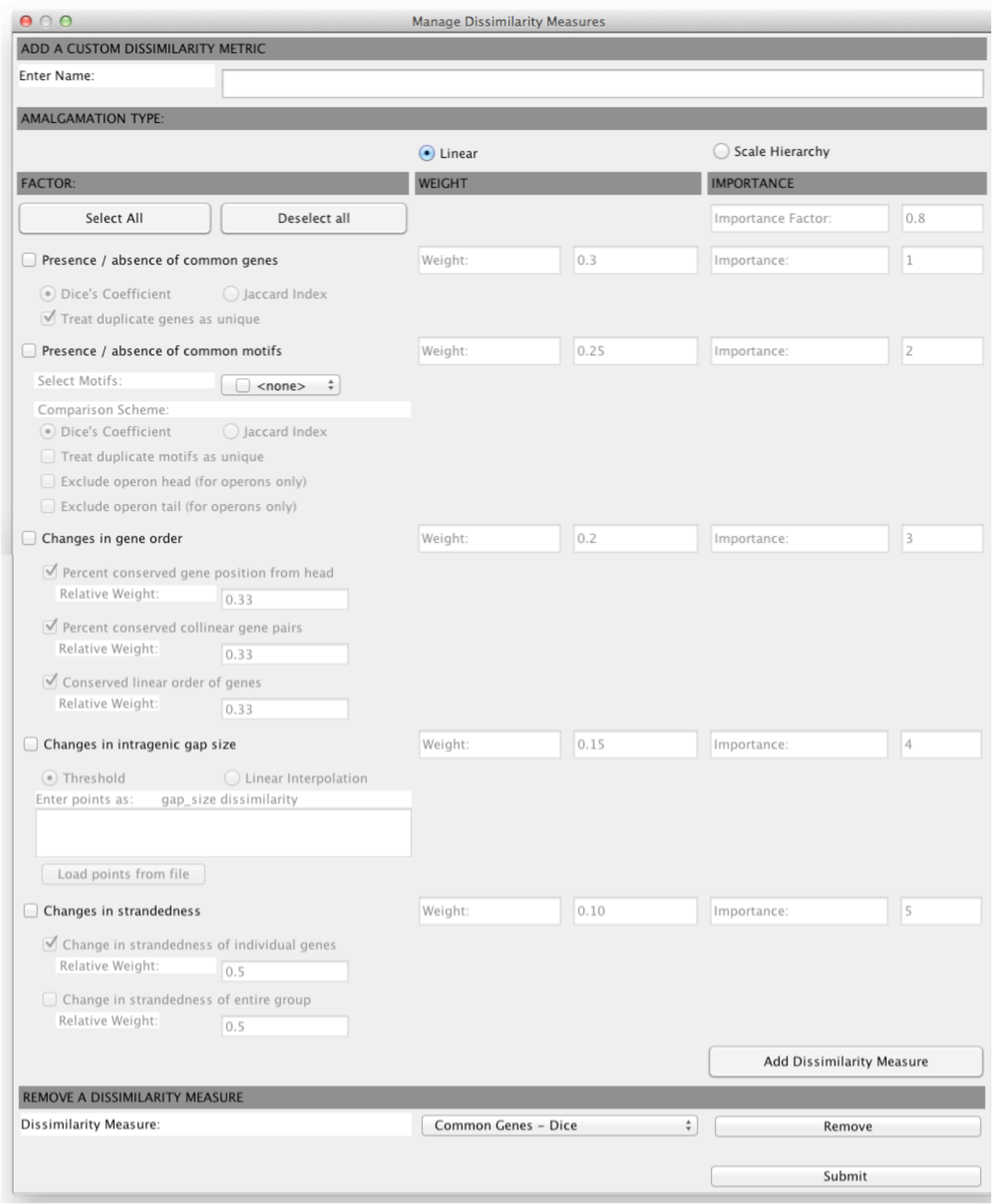

Every **Dissimilarity Measure** requires a unique name. Please enter a unique name in text field to the right of the **"Enter Name:"** label. Next, select your amalgamation type. You may select either **Linear** or **Scale Hierarchy**.

Amalgamation Types

Custom dissimilarities consist of an amalgamation of a number of biologically relevant factors relating to the genomic groupings. These amalgamation types determine the relative importance of information relating to the individual genomic groupings.

(1) Linear

The **Linear** amalgamation type sums the individual dissimilarity contribution of all factors in a weighted average, using the weights specified in the appropriate text field.

If the sum of the total weights of all selected fields is some value other than 1, then the values are scaled appropriately so that the sum of all weights equals 1.

(2) Scale Hierarchy

The **Scale Hierarchy** amalgamation type ensures that a dissimilarity contribution of lower importance never overtakes a contribution of higher importance.

Dissimilarities of lower importance are reduced to, at maximum, the dissimilarity of the next higher importance. Importance order is designated by the user in the appropriate field next to each factor, when the **Scale Hierarchy** radio button is selected. An importance factor of 1 designated maximum importance, with increasing numbers designating decreasing importance. **To designate two factors as having equal importance, assign them the same importance value.** Importance values should always start at 1 and count up, integrally, for all appropriate factors.

Once individual factor dissimilarities have been adjusted based on their importance, the factor dissimilarities are summed to produce the overall dissimilarity.

Dissimilarity Factors

To assess quantitative dissimilarity between gene groupings, many biological considerations may be important. JContextExplorer implements 5 factors which may be easily assessed from a set of annotated genomes, which may be combined using either of the above amalgamation types.

When constructing context trees, it is important to remember that individual factors will only assess a specific type of difference. **When the comparison cannot be made between sets, the dissimilarity will evaluate to 0.** For example, it is not possible to evaluate changes in gene order between two sets that do not contain any of the same genes.

A dissimilarity of zero means that **according to the factor (or factors) under investigation**, no measurable difference exists, **or the dissimilarity could not be evaluated**. Careful construction of dissimilarity and search queries should avoid this problem.

(1) Presence / absence of common genes

Genomic groupings are treated as sets of genes, and based on either gene annotation or cluster ID **(depending on the search type)**, each gene grouping is evaluated for common, shared, and unique elements.

Two algorithms are available: **Dice's Coefficient** and the **Jaccard Index**.

Given two genomic groupings **X** and **Y**, these values are defined as follows:

Dice's Coefficient: $$d=1-\frac{2|X \cap Y|}{|X|+|Y|}$$

Jaccard Index: $$d=1-\frac{|X \cap Y|}{|X \cup Y|}$$

Checking the box designated **Treat duplicate genes as unique** will retain the number of copies of identical instances for cases where multiple instances of identical elements exist. Leaving this box unchecked will condense all copies of identical elements into a single copy, across sets.

For example, if **X** contains one copy of gene *abc* and **Y** contains 2 copies of gene *abc*, with the box checked, these sets will differ by a copy of *abc*, however leaving the box unchecked indicates no difference between these sets, with regard to gene *abc*.

If a gene has no cluster ID, it will be considered unique to the set. Therefore, if both **X** and **Y** contain a large number of genes with no cluster ID, they will score a very high dissimilarity. **To avoid this problem, assign all genes a cluster ID.**

If no common genes are found between **X** and **Y**, the dissimilarity is returned as 0.

(2) Presence / absence of common motifs

It is possible to import one or more pre-computed position-specific functional features, and associate these features with one or more genes. In JContextExplorer, these features are referred to as **motifs** (after protein-binding site sequence motifs), and may refer to any type of feature often associated with one or more genes. **Motifs** need not be protein-binding site sequence motifs: they may refer to **any functional feature with a known position in the genome.** Examples include single nucleotide polymorphisms (SNPs), clustered regularly interspaced short palindromic repeats (CRISPRs), promoters, terminators, and may even refer to more abstract constructions such as "genes expressed during mid-log phase" or "gene associated with immune response". Motifs should be pre-computed and loaded into JContextExplorer in a series of files, at which point they may be associated with one or more genes. For a detailed description of how to load and associate motifs with genomic features, please see **Associating Sequence Motifs with Genomic Features**, page 102)**.**

When comparing genomic groupings, motifs are tabulated **for every gene they are associated with in gene-specific manner**, and are evaluated based on their presence or absence. This presence or absence may be selected according to both **number of different types** and **total number** of motifs.

Motif associations are compared between genomic groupings in the following way:

(A) Only motifs selected in the **drop-down check box menu** will be evaluated for. Please ensure that one or more motifs have been properly loaded and associated with the appropriate genomic features.

(B) Select either the **Dice's Coefficient** or **Jaccard Index** comparative approach. **These formulations refer to the number of motif instances associated with individual genes common to both genomic groupings.**

Dice's Coefficient: $d=1-\frac{2|X \cap Y|}{|X|+|Y|}$

Jaccard Index: $d=1-\frac{|X \cap Y|}{|X \cup Y|}$

In this case, **X** and **Y** refer to all motifs **associated with a single gene**. **X** is a gene from one genomic grouping, and **Y** is a gene from the other.

(C) If it is appropriate, check the box marked **Treat duplicate motifs as unique**. This will compress multiple instances into a single presence or absence. This should not be checked when the number of individual binding sites is important – for example, comparing genes that have 2 promoters versus 1 (a possible alternate promoter).

(D) If it is appropriate, check the box marked **Exclude operon head (for operons only)**. This will ignore motifs associated with the first gene in a same-stranded collinear gene set (often a predicted operon) in all subsequent analyses.

(E) If it is appropriate, check the box marked **Exclude operon tail (for operons only)**. This will ignore motifs that are not associated with the first gene in a same-stranded collinear gene set (often a predicted operon) in all subsequent analyses.

(F) For all common genes between **X** and **Y**, the Dice/Jaccard index is assessed. For cases where there are multiple identical genes in **X** and **Y**, a **Hungarian mapping algorithm** is employed to minimize the dissimilarity. For example, suppose **X** contains 2 copies of gene ***a*** and **Y** contains only 1 copy of gene ***a***. The first copy of gene ***a*** in **X** has motif **m** associated with it, the other has no motifs associated with it, and the copy of gene ***a*** in **Y** has no motifs associated with it. The dissimilarity for gene ***a*** may be either 1 or 0, depending on which ***a*** from **X** is compared to the ***a*** from **Y**. In JContextExplorer, the mapping that results in the minimum dissimilarity is always selected, so the dissimilarity is taken to be 0 in this case. The **Hungarian Mapping** algorithm always produces the minimum dissimilarity, for any number of copies of ***a*** in **X** and **Y**.

(G) The dissimilarity of each common gene mapping is summed, and divided by the total number of common genes.

(H) In order to make this comparison, there must be at least one common gene between **X** and **Y**. **If there are no common genes between the two sets, the dissimilarity is returned as 0**.

(3) Changes in gene order

Suppose two genomic groupings **X** and **Y** each contain a series of genes ***a*, *b*,** and ***c*.** In **X**, however, the genes are arranged in the order of ***a, b, c***, and in **Y** the genes are arranged as ***a, c, b****.* Clearly, a change in gene order has occurred – intuitively, ***b*** and ***c*** have switched positions. Perhaps **Y** contains genes arranged as ***c, a, b*** – which might be intuitively construed as ***c*** relocating from the back of ***a, b*** to the front. In both cases, these are examples of changes in gene order, however they may reflect different biological phenomena. To account for these two disparate types of changes in gene order, JContextExplorer has implemented two distinct approaches: **(1) Percent conserved gene positions from head**, and **(2) Percent conserved collinear gene pairs**.

To determine changes in gene order between genomic groupings **X** and **Y**, the following protocol is carried out:

(A) Genes that are not common to both groupings are discarded. The total number of common elements is computed (counting all duplicate identical elements as unique).

(B) The **Percent conserved gene positions from head** aspect of gene order is computed:

a. The first genomic feature is considered to be a "pivot".

b. From the head, the number of conserved positions (including the head) between gene grouping **X** and **Y** are counted. For example, if gene grouping **X** consists of **X = <*a, b, c*>,** and **Y = <*a, b, c*>**, the count is = 3. If **X = <*a, b, c*>,** and **Y = <*a, c, b*>,** the count is = 1.

c. Step (b) is repeated, however the second genomic grouping is counted backwards. This is to handle the case where every gene is on the opposing strand. For example, if gene grouping **X** consists of **X = <*a, b, c*>,** and **Y = <*c, b, a*>**, the count is = 3. If **X = <*a, b, c*>,** and **Y = <*a, c, b*>,** the count is 0.

d. The higher of the two counts are taken (moving along **Y** in the forward direction and in the reverse direction), and dissimilarity is returned as $d=1-\frac{\text{Max(Forward Count, Reverse Count)}}{\text{Total Size}}$. If the value is less than 0, a dissimilarity of 0 is returned; if the value is greater than 1 a dissimilarity of 1 is returned.

(C) The **Percent conserved collinear gene pairs** aspect of gene order is computed:

a. Lists of all adjacent pairs of genomic features in **X** are generated, Starting from the first gene in **X** and moving downstream.

b. Lists of all adjacent pairs of genomic features in **Y** are generated. A set is generated moving both downstream (the forward set) and from the most downstream gene, moving upstream (the reverse set).

c. The number of common adjacent pairs in **X** and the number of common adjacent pairs in both the forward set and reverse set of **Y** are computed.

d. The higher of the two counts in **Y** (forward or reverse) is retained.

e. The dissimilarity is returned as 1 - the ratio of the number of common adjacencies minus the maximum possible number of adjacencies:

$$d=1-\frac{\text{Max(Common Adjacencies Forward Y, Common Adjacencies Reverse Y)}}{\text{Maximum Possible Number of Common Adjacencies}}$$

f. If the one or both of the genomic groupings contains only one gene (and thus, zero adjacencies), **the dissimilarity is returned as 0** (because no comparison can be made).

g. Examples: if gene grouping **X** consists of **X = <*a, b, c*>,** and **Y = <*a, b, c*>**, the number of common adjacencies is 2 (**<*a,b*>** and **<*b, c*>**), and the dissimilarity is 0. if gene grouping **X** consists of **X = <*a, b, c, d*>,** and **Y = <*d, a, b, c*>,** the number of common adjacencies is again 2 (**<*a,b*>** and **<*b, c*>**), but the dissimilarity is 0.5 (half of all adjacencies agree and half do not. In the above two examples, **Y** could be reversed: **Y = <*c, b, a*>** and **Y = <*c, b, a, d*>** with no change to the results.

(D) The **Conserved linear order of genes** aspect of gene order is computed:

a. Lists of all adjacent pairs of genomic features in **X** are generated, Starting from the first gene in **X** and moving downstream.

b. Lists of all adjacent pairs of genomic features in **Y** are generated. A set is generated moving both downstream (the forward set) and from the most downstream gene, moving upstream (the reverse set).

c. The number of common adjacent pairs in **X** and the number of common adjacent pairs in both the forward set and reverse set of **Y** are computed.

(E) These two aspects of gene order are combined in a weighted average to describe the overall dissimilarity, using the weights supplied by the user.

(4) Changes in intergenic gap size

Suppose two genomic groupings **X** and **Y** each contain adjacent genes ***a*** and ***b***. Suppose that the distance between the start and stop positions of ***a*** and ***b*** differ in **X** and **Y** - that is, a change in **intergenic gap size** has occurred. **Note that only genes that are represented as adjacent in the genomic grouping may be evaluated for intergenic gap size.** This does not mean that the genes must be adjacent on the genome – there may be other genes between them that are not members of the genomic grouping.

JContextExplorer offers two modes to assess these differences: either the **Threshold** approach or the **Linear Interpolation** approach.

Both approaches require the user to enter a set of **gap size** to **dissimilarity** mappings in the provided text area. These mappings indicate that for a change in intergenic **gap size** of a particular size, the associated **dissimilarity** should be assessed.

Mappings should be entered one per line, formatted as:

gap_size dissimilarity_value

If a **Threshold** approach is used, gap sizes are assessed using only the provided threshold values as limits. If **Linear Interpolation** is selected,

mappings are generated that are linear interpolations between mapping points. In either case, a point-by-point mapping is determined.

This is demonstrated in the following example.

Given the mapping set

```
Enter points as:      gap_size dissimilarity
0 0.0
20 0.1
50 0.4
```

If **Threshold** is selected, the gap size – dissimilarity mapping looks like this:

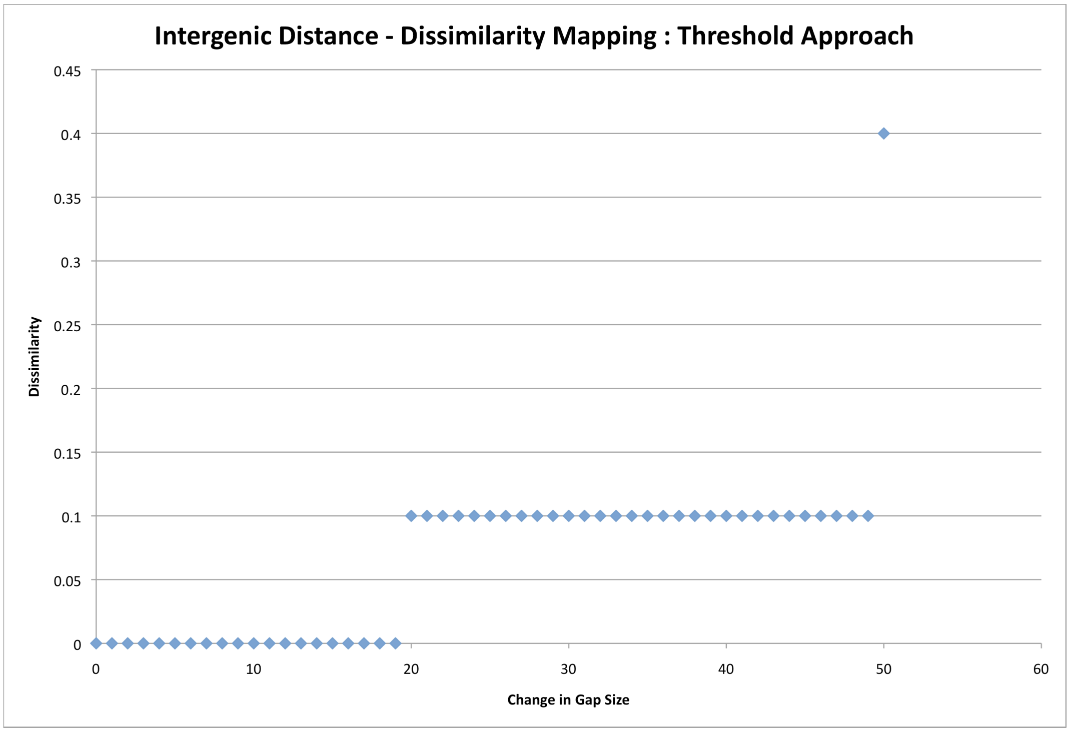

If **Linear Interpolation** is selected, the gap size – dissimilarity mapping looks like this:

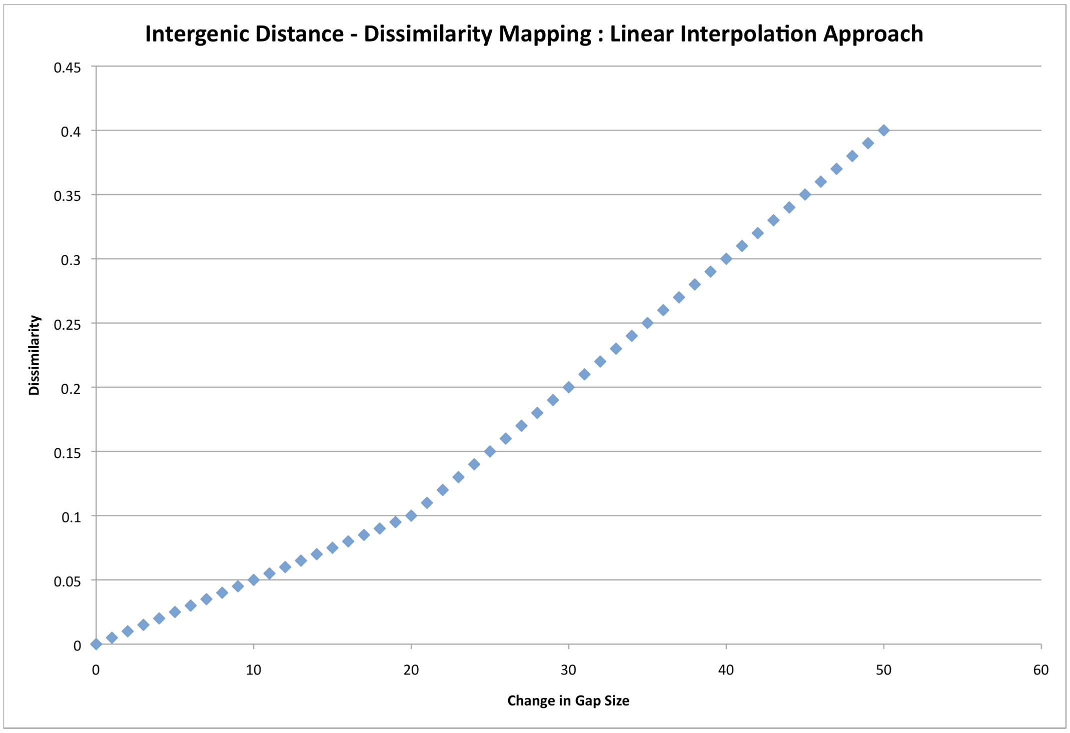

The overall dissimilarity is returned as the sum of all changes in gap size, between all determined adjacencies.

If a gap size has changed more than the maximum supplied **gap size - dissimilarity** value, the dissimilarity is returned as 1.

If no adjacent pairs are found to be in common between **X** and **Y**, the dissimilarity is returned as 0.

(5) Changes in strandedness

As genes rearrange in genomes across evolutionary time, they may relocate from the forward to the reverse strand, or vice versa. In general, this may occur in two ways: **(1)** Individual genes may switch strands or **(2)** larger groups of genes may together switch strands. JContextExplorer offers two different metrics to assess each of these types of changes in strandedness.

To determine changes in strandedness of between genomic groupings **X** and **Y**, the following protocol is carried out:

(I) Genes that are not common to both groupings are discarded.

(J) The ratio of instances where common elements have the same strand is compared to the total number of common elements.

$$r_B = \frac{|\text{Common Elements with Same Strand}|}{|\text{Common Elements}|}$$

(K) The ratio of instances where common elements have the same strand is compared to the total number of common elements, when the strandedness of every gene in one of the sets is reversed. This step is carried out because for many draft genomes, strandedness is often provided only relatively, and not with respect to a biological indicator of true strandedness. Note that mathematically, this is identical to tabulating the number of common elements with different strands from the original **X** and **Y**.

$$r_C = \frac{|\text{Common Elements with Different Strand}|}{|\text{Common Elements}|}$$

(L) One minus the higher of the two ratios computed in **(B)** and **(C)** is returned as the dissimilarity for the **change in strandedness of individual genes** (designated by a check box in the custom dissimilarity frame). If the dissimilarity computed in **(B)** is greater than or equal to the dissimilarity computed in **(C)**, the **Change in strandedness of entire group** dissimilarity (designated by a checkbox in the custom dissimilarity frame) is returned as 0, otherwise, the dissimilarity is returned as 1 (indicating that the whole segment has changed strands). Note that both ratios in **(B)** and **(C)** correspond to the Jaccard Index, where the elements are defined as common according to query match and strandedness.

Once each of these types of changes in strandedness has been evaluated, a total change of strandedness is computed via weighted average, using provided weights (indicated by the **Relative Weights**) fields in the custom dissimilarity frame. Note that these weights are used to compute only the

strandedness factor, and differ from the weights associated with linear amalgamation and scale hierarchy importance values.

For cases where multiple identical instances are found in **X** and/or **Y** changes in strandedness is evaluated in such a way as to minimize the dissimilarity. For example, suppose **X** contains 3 instances of gene ***a***, two of which exist on the forward strand and one on the reverse strand. Suppose **Y** contains 2 instances of gene ***a***, one of which exists on the forward strand and one on the reverse strand. In this case, no dissimilarity would be exacted based on Strandedness. Suppose, however, that **X** contains only 2 instances of gene ***a***, both on the forward strand, and **Y** contains 2 instances of gene ***a***, one of the forward strand and one on the reverse strand. In that case, a dissimilarity penalty would be exacted.

If no common genes are found between **X** and **Y**, the dissimilarity is returned as 0.

Included Dissimilarity Types

JContextExplorer comes pre-loaded with several dissimilarity types. These dissimilarity types are designed for disparate biological analyses. What follows is a brief description of these types, with suggested use cases. We recommend using these dissimilarity measures as starting points for your analysis, however **we strongly recommend creating customized dissimilarities.** JContextExplorer is designed for **exploration and re-analysis**, creating and tweaking parameters in customized dissimilarity measures is an effective way to do this.

(1) Common Genes – Dice

All common genes are identified between two genomic groupings. Common genes are defined either by common cluster ID number (if the search carried out is homology cluster - based) or annotation (if the search carried out is annotation – based). The pairwise dissimilarity **d** between gene groupings **X** and **Y** is computed according to the Dice Formula:

$$d=1-\frac{2|X \cap Y|}{|X|+|Y|}$$

Note that this setting treats all copies of identical instances where multiple instances of identical elements exist as unique. This is equivalent to checking the box marked “**Treat duplicate genes as unique”** In the Gene Grouping factor when designing a customized dissimilarity.

(2) Common Genes – J’accard

All common genes are identified between two genomic groupings. Common genes are defined either by common cluster ID number (if the search carried out is homology cluster - based) or annotation (if the search carried out is annotation – based). The pairwise dissimilarity between gene groupings X and Y is computed according to the J’accard Formula:

$$d=1-\frac{|X \cap Y|}{|X \cup Y|}$$

Note that this setting treats all copies of identical instances where multiple instances of identical elements exist as unique. This is equivalent to checking the box marked "**Treat duplicate genes as unique"** In the Gene Grouping factor when designing a customized dissimilarity.

(3) Moving Distances

In microbial genomes, co-transcribed features are often grouped into same-stranded positionally adjacent groupings (operons), with little intergenic spacing between them. As the spacing between individual features widens, this could indicate a change in the transcriptional processing of a genomic grouping: for example, a large widening in the center of a tightly packed gene grouping could indicate the splitting of one operon into two. Also relevant to this comparison is a rearrangement of genes within a single operon: gene order in operons may convey information about the relative importance of transcribed products. This pairwise comparison metric attempts to capture these behaviors, through a weighted sum of observed differences (penalties) between two genomic groupings X and Y.

The Moving Distances approach is designed to compare genomic groupings that contain **the same set of homologous genes**. If there is even one inclusion/exclusion, the two groupings with score a dissimilarity value of **1** (maximum dissimilarity).

Provided that for every gene in gene grouping **X** there exists a homologous gene in gene grouping **Y**, inversions / gene rearrangements between the groupings are assessed. A single rearrangement incurs a dissimilarity penalty of **0.2.** If rearrangements have occurred, the rearrangements are counted, and a dissimilarity measure is returned. Therefore, if 5 or more rearrangements are counted, genomic groupings are returned with a dissimilarity score of 1 (maximum dissimilarity).

If no rearrangements have occurred, distance-widening-based penalties are then assessed.

If no widening has occurred between analogous genes across genomic groupings, no penalty is incurred.

If a **slight widening** (between 10 and 25 nt) has occurred, this incurs a dissimilarity penalty of **0.02**

If a **medium widening** has occurred (between 25 and 200 nt) has occurred, this incurs a dissimilarity penalty of **0.05**

If a **large widening** has occurred (greater than 200 nt) has occurred, this incurs a dissimilarity penalty of **0.2**. Note that this widening often signifies a gene insertion.

Note that this dissimilarity measure bears resemblance to the **Changes in Intergenic Gap Size** factor in the customized dissimilarity metric, using a **Threshold** approach with the following mapping of gap size to dissimilarity:

gap_size dissimilarity

0 0.0
10 0.02
25 0.05
200 0.20

However, one important distinction is that in this case, **if the two genes do not contain exactly the same set of genes, the dissimilarity will score 0.** Therefore, this approach makes the most sense to use in cases among highly similar genomes, looking for effects less dramatic than gene loss or gain.

(4) Total Length

The total size of each genomic grouping **X** and **Y** is computed by taking the distance from the start of the earliest annotated feature to the stop of the latest annotated feature. The dissimilarity is taken to be the average difference:

$$d=1-\frac{2\,\text{Abs}\left(|X|-|Y|\right)}{|X|+|Y|}$$

This dissimilarity may prove useful when examining multiple gene homologs that may differ in size, or in quantifying changes in intergenic distance.

PHYLOGENETIC TREE (⌘P)

A **Phylogenetic Tree** is a branching diagram or "tree" showing the inferred evolutionary relationships among a set of species. Over the years, a tremendous number of algorithms and associated software have been developed to predict the phylogeny of organisms. To compare phylogenies with JContextExplorer's context trees, it is possible to load, view, analyze, and interactively explore phylogenies and context trees simultaneously. **JContextExplorer facilitates only loading of pre-computed phylogenetic trees, not phylogenetic tree computation.** All aspects of the tree should be determined ahead of the time and formatted appropriately. **Only Newick Tree format (.nwk) files may be imported into JContextExplorer**. Consequently, Nexus tree files should be re-formatted prior to import.

The Phylogenetic Tree has an associated panel in the main window, which is one of the tabs in the **Search Options Area**:

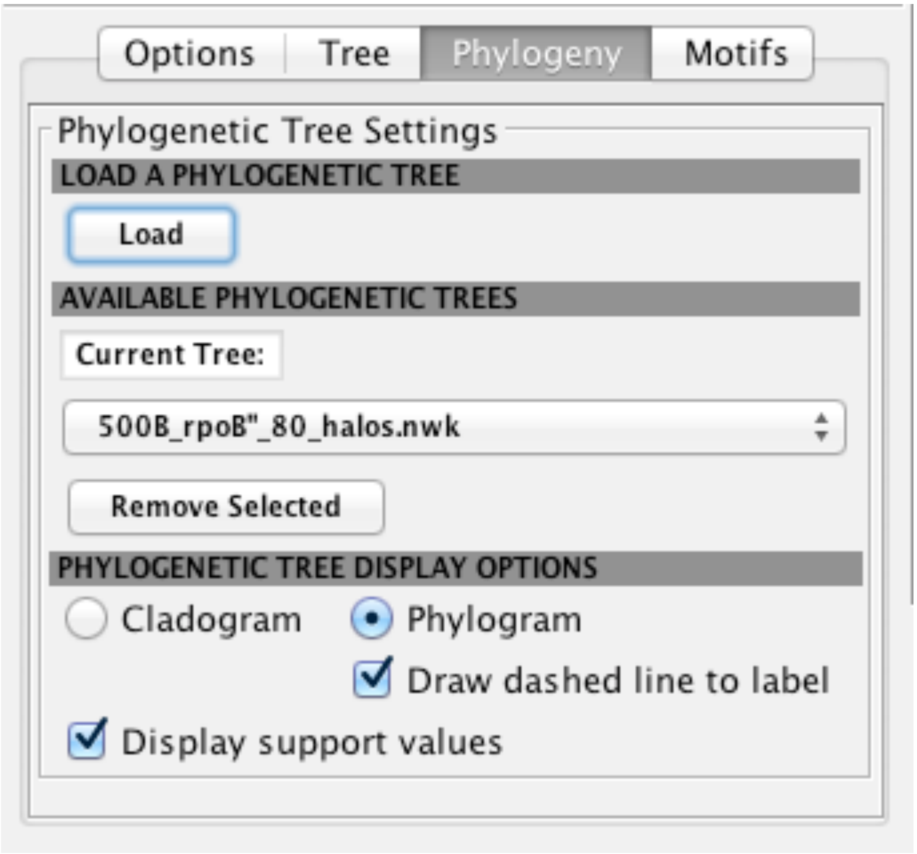

Clicking the **Load** button has the identical effect of selecting **Load Phylogenetic Tree** from the drop-down menu. This will bring up a file chooser that will invite you to select a pre-computed phylogenetic tree, in Newick format. **Please ensure that all phylogenetic tree files end with ".nwk".**

When phylogenetic trees are imported into JContextExplorer, they are always associated with the current genome set. When switching back and forth between genome sets, all phylogenetic trees associated with the new genome set are loaded as well.

Note that phylogenetic trees are drawn with the execution of a search query. **Phylogenetic trees will appear as their own panel in the internal frame generated with search results**. If a search has no results, the phylogenetic tree will not be drawn.

Phylogenetic trees may be interactively explored, like context trees, but leaf names should match organism names. Leaf names that do not match organism names are still permitted, however they will not allow for interactive exploration between component panels within a single internal frame. Unlike the interactions associated between context trees and search results frames, selecting a single leaf in a phylogenetic tree may select multiple leaves in a context tree or multiple search result entries: for example, if an organism has multiple 'hexokinase' genes, selecting that organism in the phylogenetic tree will select all 'hexokinase'-associated contexts in the other frames. For a demonstration of interaction between search windows, phylogenetic trees, and context trees, please see the **Internal Frame Management Area** section, page 22.

Multiple Phylogenetic trees may be loaded and associated with the current genome set, however only one may be displayed with contexts at a time. **The active phylogenetic tree (displayed with context trees, if appropriate) is the one selected in the drop-down menu.**

To remove a phylogenetic tree, click the **Remove selected button**. This will remove the currently active phylogenetic tree.

Sometimes, phylogenetic trees merely show branching order (cladogram), but in other cases, branch lengths can be used to quantitatively describe evolutionary distance (phylogram). Either format may be imported into JContextExplorer with the appropriate radio button selected. If a phylogram is imported into JContextExplorer, it may still be rendered as a cladogram – branch lengths will be extended to the maximum branch length.

Checking the **Display Support Values** box will display bootstrap values on the tree at appropriate branch points, if they are included in the tree file.

Before systematically comparing phylogenetic trees against context trees, please see **Comparing Against a Phylogenetic Tree** on page 122.

SEQUENCE MOTIFS (⌘E)

A **Sequence Motif** is a nucleotide pattern that is widespread and has, or is conjectured to have, biological significance. Often, this refers to the DNA-binding sites of a particular protein (or proteins), however this need not be true – for example, a single-nucleotide polymorphism (SNP) might be imported as a sequence motif, as might a transcription start site or termination site. In general, any sequence-level features of the genome that it is not appropriate to designated as genomic features may be imported as sequence motifs, and treated as such.

JContextExplorer has functionality to load the one or more sequence motifs. What is important to JContextExplorer is not the motif itself, but **the locations of the individual motif occurrences.** JContextExplorer does not have functionality to discover statistically overrepresented sequence motifs, or to scan for an existing motif – hundreds of tools already exist for this purpose. **Only after a motif has been determined, characterized, and mapped to specific nucleotide sequences may the motif be imported into JContextExplorer.**

Sequence Motifs have an associated panel in the main window, which is one of the tabs in the **Search Options Area**:

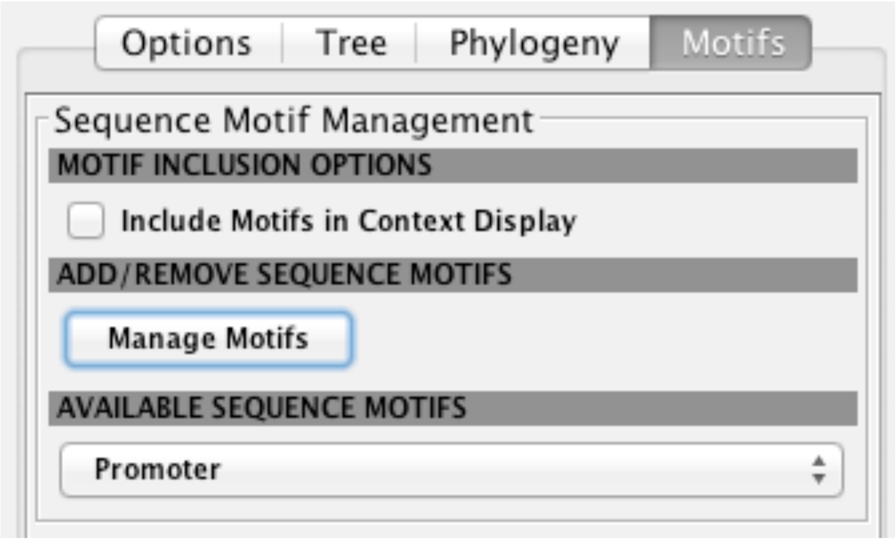

Once a motif has been loaded and (optionally) associated with one or more genomic features, checking the **Include Motifs in Context Display** box will display all motifs when a multi-genome browser viewer is launched.

At the bottom of the frame, under the banner **Available Sequence Motifs**, a drop-down list shows the currently loaded motifs.

Motifs can be added or removed by clicking the **Manage Motifs** button.

Clicking the **Manage Motifs** button will bring up the following window:

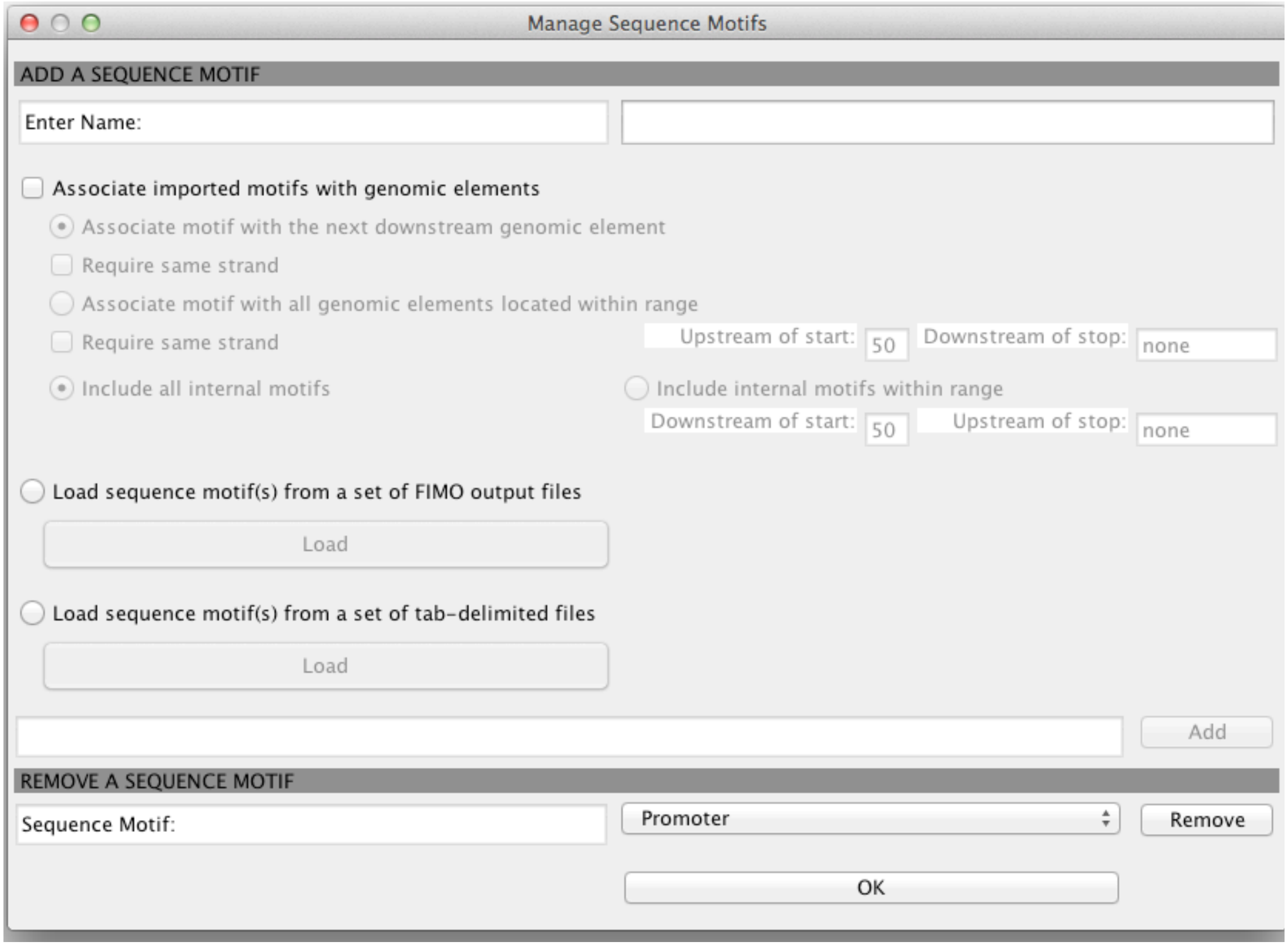

Every **Sequence Motif** requires a unique name. Please enter a unique name in text field to the right of the **"Enter Name:"** label.

Next, select whether you would like to associate the imported sequence motif with appropriate genomic elements (aka genes). Please see the next section for a detailed description for sequence motif – gene association.

Finally, select one of the radio buttons below, and click the **Load** button under the selected option to begin loading motifs.

NOTE: in both cases, if the start position occurs before the stop position, the motif is mapped to the forward strand. If the start position occurs after the stop position, the motif is mapped to the reverse strand.

Load sequence motif(s) from a set of FIMO output files.

FIMO refers to "Find Individual Motif Occurrences" and is a motif-scanning tool that is a part of the MEME Suite.

Grant, C. E., Bailey, T. L., & Noble, W. S. (2011). FIMO: scanning for occurrences of a given motif. *Bioinformatics (Oxford, England)*, *27*(7), 1017-8. doi:10.1093/bioinformatics/btr064

Among the types of output files FIMO can produce, it can produce a tab-delimited textual output file called **FIMO.txt**.

To import results from a FIMO run, after clicking the **Load** button, a file chooser will appear. Please select one of the following:

(1) A directory containing FIMO output directories named according to one or more genomes in your genome set

(2) A directory containing FIMO.txt output files re-named according to one or more genomes in your genome set (ex: Genome1.txt)

Note that JContextExplorer is designed to import either text files or directories associated with a **single genome in the genome set**. FIMO should be run serially on a number of genomes, and outputs of each FIMO run should be amalgamated into a single directory.

Load sequence motif(s) from a set of tab-delimited files.

JContextExplorer is designed to load motif information from one or more text files. **Each line in the file represents a single sequence motif instance.** Files should be formatted in one of two ways:

Single File Format

column 1: Genome

column 2: Sequence Name (Contig)

column 3: Start position

column 4: Stop position

column 5: Notes about motif (optional)

Directory Format

Note that a directory of files may also be imported. Each file in the directory should be named according to a genome in the genome set: **<GenomeName>.txt**. Files should be formatted as follows:

column 1: Sequence Name (Contig)

column 2: Start position

column 3: Stop position

column 4: Notes about motif (optional).

Associating Sequence Motifs With Genomic Features

Two methods are available in JContextExplorer to associate a motif with a genomic feature, and are designated by radio buttons:

(1) Associate motif with the next downstream genomic element

Motifs will be associated with the next appropriate downstream genomic element. If the **Require same strand** box is checked, then motif will only be associated with elements that have the same strand (differently-stranded genomic elements will be skipped over).

If the sequence motif instance is internal to a genomic element or overlaps with the start of a genomic element (partially internal), **the sequence motif instance will be associated with that element.** Once a sequence motif has been associated with a genomic element, it may be not be associated with any other genomic element.

(2) Associate motif with all genomic elements located within range

Rather than map a motif to a single genomic element, this approach allows multiple motifs to be mapped to a single genomic element, based entirely on proximity.

Checking the **Require same strand** box requires that the genomic element and the sequence motif have the same strand for the sequence motif instance to be mapped to the genomic element.

Sequence motifs may be mapped to a genomic element according to the **proximity of their center to the start position and stop position** of said genomic element. Selecting the **Include all internal motifs** radio button will associate all motifs internal to a genomic element with that genomic element, otherwise you may select ranges to associate motif instances with the start and stop of a genomic element.

For example, Sequence motif mapping settings of:

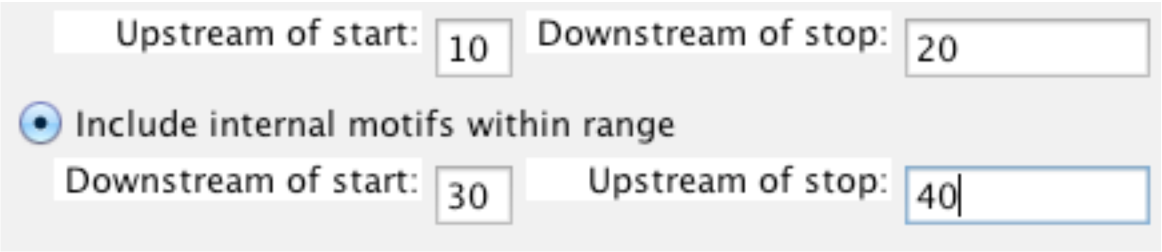

Will associate motifs to genomic elements as follows:

Elements on Forward Strand:

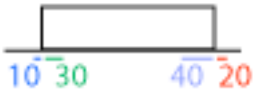

Elements on Reverse Strand:

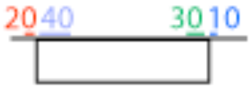

To avoid associating a motif according to any of the above distance associations, **set the value in the associated textbox to a negative integer or a non-numeric number.**

As a default, motifs are not associated downstream of the stop position, nor internally downstream of the stop (these fields are initialized to values of **none**).

EXPORT MENU

While JContextExplorer has many powerful features, there may be certain analyses that are not possible within JContextExplorer – in this case, it may be useful to export the information from a particular **Genome Set**, and perform these analyses elsewhere. Also, it may be useful to save a particular genome set, and launch this set later. The major contents of a genome set may be exported in several different forms from the **Export Menu**. Information from analyses carried out within a genome set – information generated in context trees or the contexts aligned from a series of genomes – may also be exported, however not from this menu (please see **Internal Frame Management Area**, page 22, and **Context Viewer Multiple Genome Browser**, page 32 for more information).

The Export Menu may be selected from the main menu bar, and when expanded looks like this:

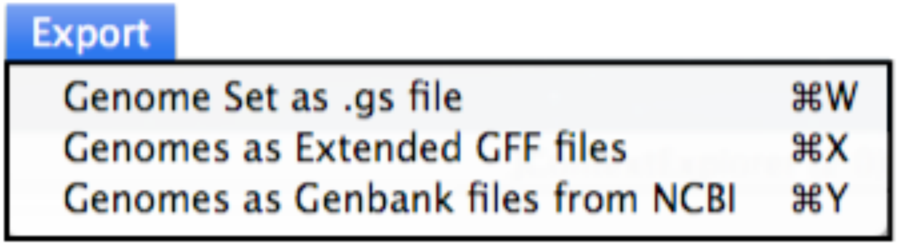

GENOME SET AS .GS FILE (⌘W)

Selecting this option from the Export Menu will cause a file dialog box to appear, allowing you to designate a name to this .gs file, and a location on your file system. The default name of the exported genome set is the name of the genome set with the extension .gs

Note that if this file is re-imported, all associated information – context sets, dissimilarity measures, sequence motifs, phylogenetic trees, homology clusters, etc – will also be retained.

GENOMES AS EXTENDED GFF FILES (⌘X)

Selecting this option from the Export Menu will cause a file dialog box to appear, allowing you to designate a directory to write the genomes into extended .GFF file format.

In the directory you select, **each genome will be written into its own file,** with the name **<genome file name>.gff**.

WARNING: If a file **<genome file name>.gff** already exists, it will be overwritten. Please choose your output directory carefully to avoid this!

Genomic features will be written, line-by-line, into each associated genome file. Each tab-delimited column in each line in the file is as follows:

column 1: Contig or Sequence Name

column 2: the text string “GenBank” **<constant>**

column 3: Feature type (usually CDS, tRNA, or rRNA)

column 4: Feature start

column 5: Feature stop

column 6: the text string “+” **<constant>**

column 7: Strand (1 designated forward strand, -1 designates reverse strand)

column 8: the text string “.” **<constant>**

column 9: Feature Annotation

column 10: Homology Cluster, if it is assigned **<unique to extended GFF format>**

column 11: Gene ID, if it is assigned **<unique to extended GFF format>**

Note that columns 2, 6, and 8 are largely information to be ignored, and columns 10 and 11 are extensions of the classic GFF file format.

GENOMES AS GENBANK FILES FROM NCBI (⌘Y)

Selecting this option from the Export Menu meets this dialog box:

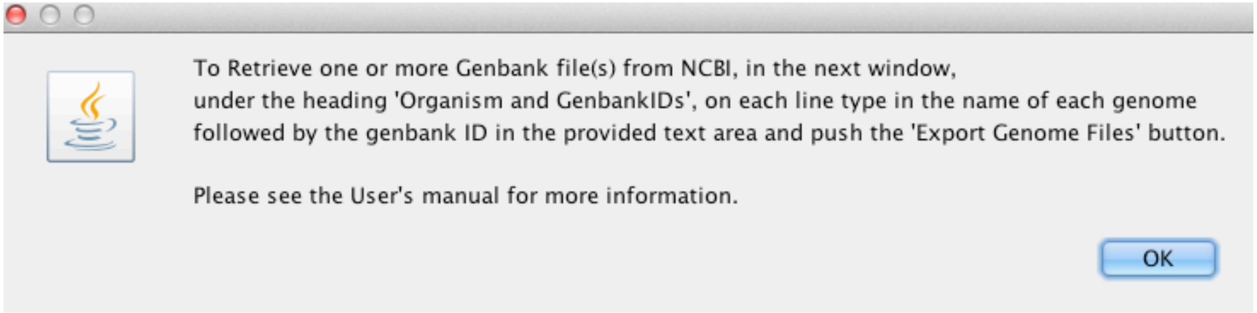

Clicking the **OK** button redirects you to the **Import Directly from NCBI Databases** window. Please see **Import Genomes into Current Genome Set -> Directly from NCBI Databases** (page 50) for more information.

PROCESS MENU

In Previous versions, JContextExplorer was almost exclusively gene-centric: A particular gene or genes could be searched for with a particular context set, and matches to the search query could be processed and assembled into a tree. The tree could then be visualized, interrogated, re-computed (as necessary), and exported.

The above use case relies on one key assumption: that the user knows which gene or genes they want to look for. When perusing recently sequenced or poorly understood genomes, this is often not the case – textual annotation searches may be little more than shots in the dark. Additionally, even if one or more genes of interest are known, it may be worthwhile to scan the whole set systematically, searching for unexpected patterns and trends.

The Process Menu facilitates **Whole Genome-Set Analysis.** Rather than interrogating single gene queries in depth, this tool is designed to systematically process a large number of queries simultaneously. Based on some criteria (external data, similarity to an existing tree, common tree topology), the processing tools may **suggest interesting genes from a dataset when they are not known**. It is even possible to use these tools to process every gene in every genome, and make statements about the contexts of every gene in every organism.

The Process Menu may be selected from the main menu bar, and when expanded looks like this:

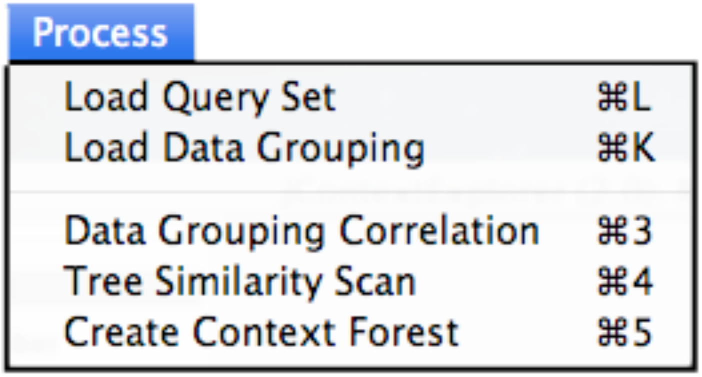

LOAD QUERY SET (⌘L)

A 'Query Set' is simply a group of search queries (and associated parameters). Rather than typing each query individually into the Main Frame window, this data object allows you to store a set of such queries, and resulting search hits / context trees. In addition to running a large number of queries with a single click, other tools in JContextExplorer allow for quantitative comparisons of the queries contained in Query Sets.

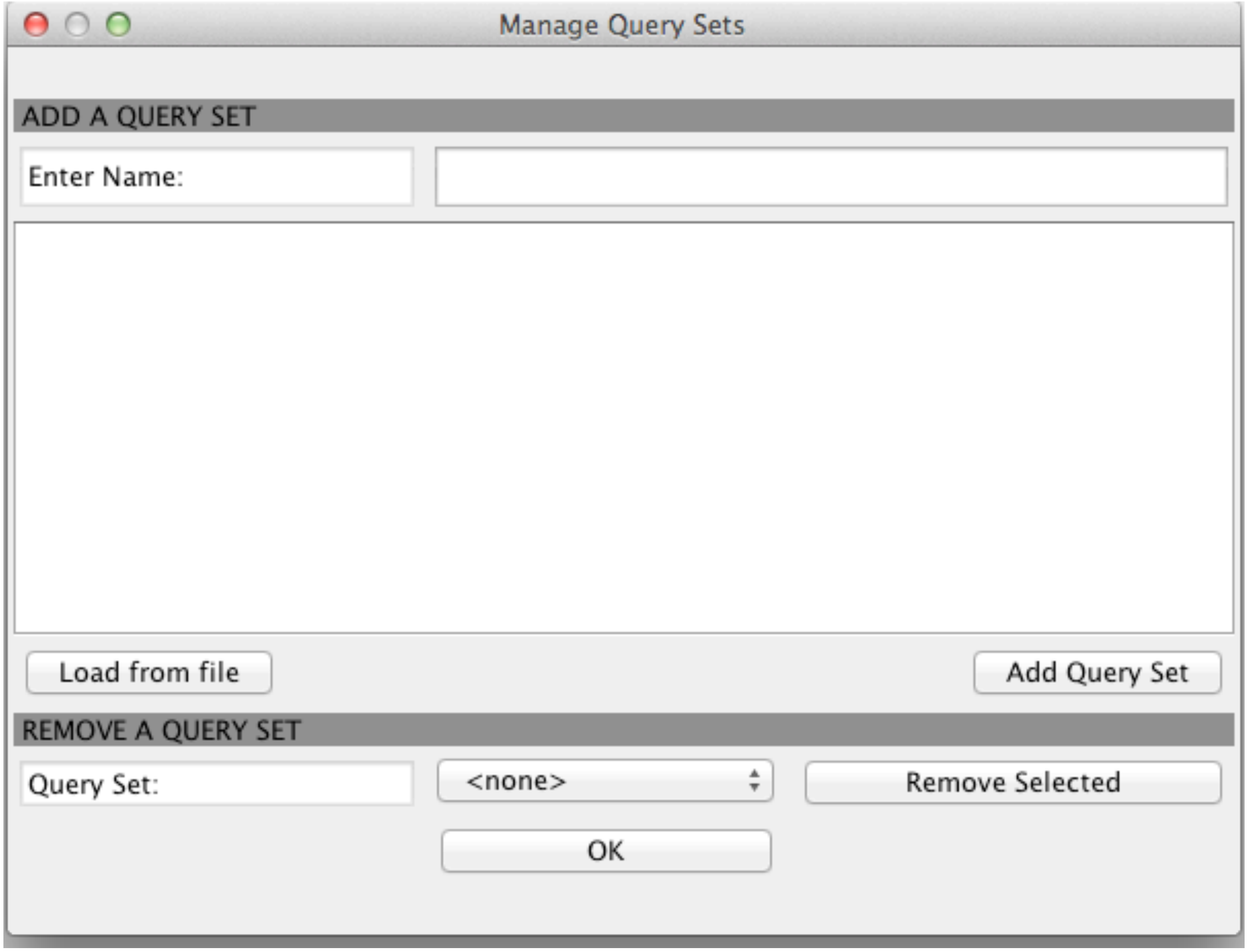

Every Query Set needs a name – please provide a unique name in the **Enter Name:** Field.

Each line in the text area is parsed as a single query. Please write your queries on this line, separated with an enter key. As a reminder, a semicolon can be used

to separate individual search elements. Each line is parsed as though it were entered into the search bar in the Main Frame window.

You may load information from a text file by clicking the **Load from file** button. This will add whatever information is in the file directly to the text area.

Once all queries have been written to the text area, click the **Add Query Set** button.

All parameters associated with the search (context set, dissimilarity measure, organism set, whether the search is annotation search / cluster number, organism set name) should be set in the JContextExplorer Main Frame window, just as they must be set in a normal, single-query search. **The values you have set at the time of submission will be stored and associated with searches in this query set, even if you change them later.** Note that this construction requires that all Query Sets must have identical parameters as far as Context Set, Annotation or Cluster Search, Dissimilarity metric, and Organism Set Name.

To remove a Query Set, click the **Remove Selected** button while the Query Set name is selected in the drop-down menu. To close the dialog box, click the OK button. If you have not added the query set (by clicking the **Add Query Set**) button, then your query set will not be retained.

Remember to click the 'Add Query Set' button before clicking the OK button at the bottom, and remember that parameters for your query set will be taken from whatever is set in the main frame at the time you click the 'Add Query Set' button, even if you change these parameters later.

Load Data Grouping (⌘K)

A “Data Grouping” is a specified grouping of individual genes or whole organisms into non-overlapping clusters. In the context of JContextExplorer, Data Groupings are computed beforehand and imported into JCE for comparisons with analogous groupings determined from Context Trees. These groupings may represent any number of things but will often summarize results of large-scale phenotype or experimental data.

Selecting ‘Load Data Grouping’ from the Process menu launches a file chooser that invites you to select a single file from your file system.

Each line in the file should consist of a tab-delimited list of Species Names, with no white space.

Each line in the file is parsed as a Data Grouping. When performing comparative analyses later, the clusters generated from Context Trees may be compared to these loaded data groupings, and assessed for similarity.

When the data has been successfully loaded, you will receive a notification. If the file could not be parsed correctly, you will also be notified.

DATA GROUPING CORRELATION (⌘3)

A data grouping correlation compares the grouping of data into non-overlapping clusters with the grouping of the same (or mostly the same) data into non-overlapping clusters another way.

In this case, the data grouped into clusters are **organisms**.

A reference grouping is created outside of JContextExplorer and loaded in, in a tab-delimited plain text file. To load in such a file, please see **Load Data Grouping** (page 111).

Groupings are created in JCE by dividing context trees into non-overlapping groups at a particular segmentation value, which is simply the height of a computed context tree. Leaves of the tree that are further apart than this segmentation value are segregated into different groups. Visually, this is often represented as “cutting” the tree at a particular value.

The grouping of the **source organisms** from the context tree id compared with the externally loaded data grouping.

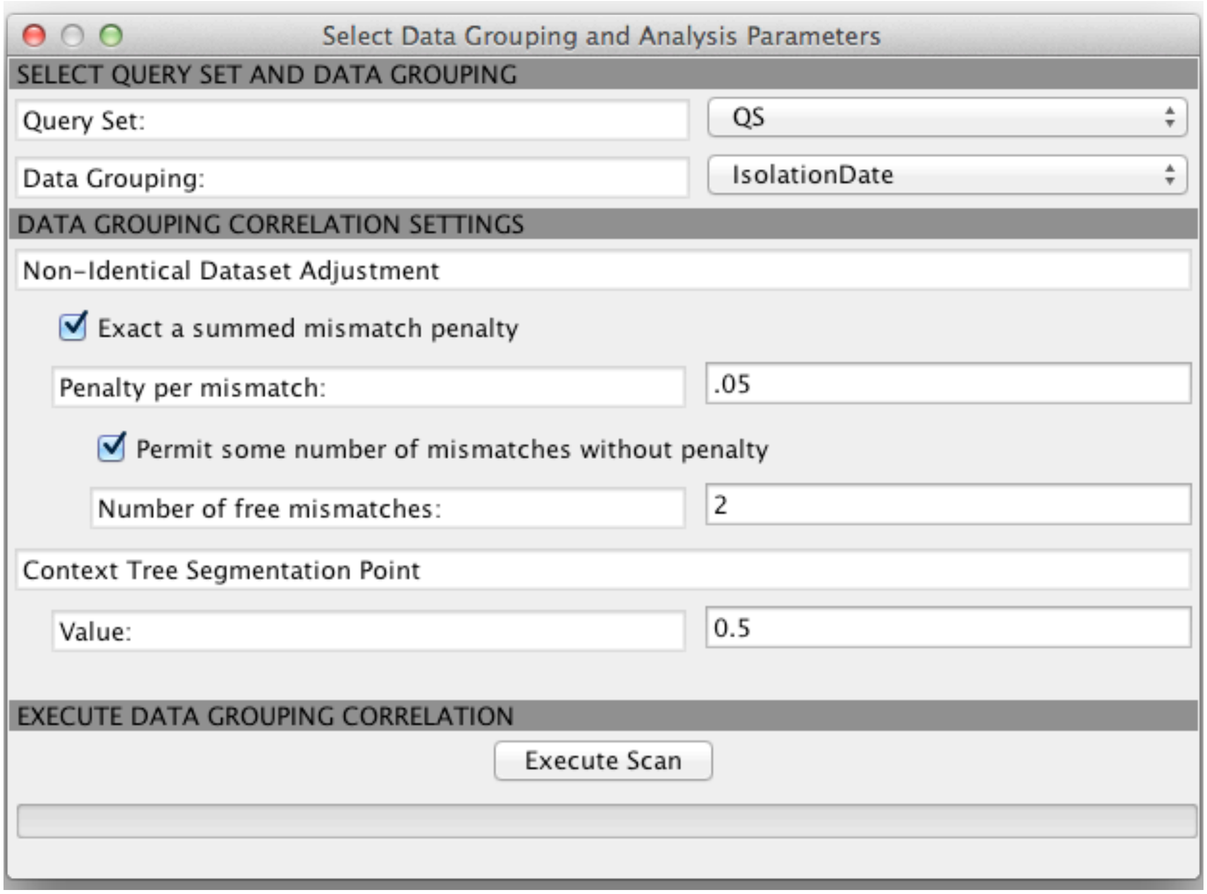

To launch the above window, one or Query Sets and one or more Data Groupings must first be loaded. A list of all available Query Sets and Data Groupings will appear under the banner, **"Select Query Set and Data Grouping".** Please select the appropriate Query Set and Data Grouping from their respective drop-down menus.

Under the banner **"Data Grouping Correlation Settings"**, the sub-menu "Non-Identical Dataset Adjustment" allows to specify a variable penalty when comparing two datasets that are not identical. This is explained in more detail in the following section, **Adjusted Fowlkes-Mallows Method**. It should be noted, however, that in many cases, dataset adjustment may not be appropriate or necessary, especially because vastly different datasets will often naturally score a dissimilarity of 0 as a product of the Fowlkes-Mallows method.

The sub-banner **"Context Tree Segmentation Point"** invites a segmentation value. This is point at which you may imagine cutting a hierarchical clustering tree into smaller, non-overlapping clusters. Please select this value with care.

When you have selected appropriate parameters and are ready to perform your correlation, click the **Execute** button. **For each query in the specified query set, the program will construct (but not display) a context tree, cut this context tree at the specified segmentation point, and use the Adjusted Fowlkes-Mallows method to describe a similarity measure (between 0 and 1) comparing the grouping of species in each context tree with the grouping of species designated in the selected Data Grouping.**

When the analysis has finished, a window will appear showing the results, sorted in order from context tree with organisms grouped most similarly to the specified external data grouping, to the context tree with organisms grouped least similarly to the specified external data grouping. For more information about the output window, please see **Process Output Window** (page 126).

The **Fowlkes-Mallows** method is the method used to compare the data grouping specified externally with the data grouping determined from each context tree. The method was first described in:

Fowlkes, E. B., & Mallows, C. B. (2013). A Method for Comparing Two Hierarchical Clusterings. *American Statistical Association*, *78*(383), 553-569.

Here, we extend this method to handle cases where the elements in each data set are not necessarily identical. This occurs when not all organisms are featured in a context tree, or a context tree contains multiple contexts from the same organism.

Adjusted Fowlkes-Mallows Method

This method works in two stages: (1) An optional adjustment step for cases where the data sets are not identical, and (2) implementation of the Fowlkes-Mallows method. In the case where the data are identical, the Adjustment step will return no adjustment (a dissimilarity of 0), and the result is identical to that of the Fowlkes-Mallows method.

(1) Adjustment Step

An individual penalty is assessed for each item that is in one data set, but not the other. This amounts to the sum of the items unique in the reference set (external grouping loaded in) and the query set (the context tree generated by JContextExplorer).

Note: multiple identical items will be scored separately. So, if the reference set contains 3 instances from Organism A and the query set contains 2 instances from the Organism A, this will be scored as 1 mismatch.

The associated penalty may be set as a numerical value between 0 and 1.

Additionally, there may be some number of mismatches allowed that incur no penalty. This number is also set in the window.

If the sum of penalties exceeds or equals 1, the similarity will be scored as 0. Otherwise, the similarity resulting from Fowlkes-Mallows method will be scaled by $1-\sum(\text{penalties})$.

(2) Fowlkes-Mallows Method

The following is a description of the Fowlkes-Mallows method, reproduced from

Fowlkes, E. B., & Mallows, C. B. (2013). A Method for Comparing Two Hierarchical Clusterings. *American Statistical Association*, *78*(383), 553-569.

Suppose we have two clusterings of the same n objects, A_1 and A_2. Suppose that A_1 contains I non-overlapping clusters and A_2 contains J non-

overlapping clusters. We may count the number of objects in common between the clusters of A_1 and A_2 and note the results in the Matrix M:

$$[M] = \begin{bmatrix} m_{11} & m_{12} & \dots & m_{1J} \\ m_{21} & m_{22} & \dots & m_{2J} \\ \vdots & \vdots & \ddots & \vdots \\ m_{I1} & m_{I2} & \cdots & m_{IJ} \end{bmatrix}$$

Where the quantity m_{ij} is the number of objects in common between the ith cluster of A_1 and the jth cluster of A_2.

The overall Similarity $s(A_1, A_2)$ is computed as

$$s(A_1, A_2) = \frac{T}{\sqrt{PQ}}$$

Where

$$T = \sum_{i=1}^{I}\sum_{j=1}^{J} m_{ij}^2 - n$$

$$m_{i\bullet} = \sum_{j=1}^{J} m_{ij}$$

$$m_{\bullet j} = \sum_{i=1}^{I} m_{ij}$$

$$m_{\bullet\bullet} = \sum_{i=1}^{I}\sum_{j=1}^{J} m_{ij} = n$$

$$P = \sum_{i=1}^{I} m_{i\bullet}^2 - n$$

$$Q = \sum_{j=1}^{J} m_{\bullet j}^2 - n$$

It is worth noting that this method is a function how a set of hierarchically clustered data is divided. Cutting a hierarchically clustered data set at a

different point will produce a vastly different set of clusters, which will result in a vastly different computed dissimilarity.

Problems associated with non-identical datasets and repeated elements

When dealing with clusters of objects that are not the same, it is possible to produce a dissimilarity that is less than 0, greater than 1, or not a valid rational number (this occurs when P or Q are 0, which would entail a division by 0). If the similarity is computed to be 0 or does not exist, the similarity returned is 0; if the dissimilarity is greater than 1, a similarity of 1 is returned.

In certain cases, there may be multiple identical elements in a dataset. This occurs often when a particular genomic context occurs multiple times in the same organism. When comparing presence or absence of multiple distinct elements, without further information, it is impossible to map distinct identical elements across sets accurately.

This is exemplified in the following case: suppose an associated context X has 2 instances in organism 1. In general, we may perform an adjusted Fowlkes-Mallows context scan on another context, context Y.

Let us suppose the special case X = Y. In general, this information would not be known when the context scan is performed. From context X, there are two contexts stemming from organism 1, and from context Y, there are again two contexts stemming from organism 1. Ideally, **each context stemming from organism 1 in the set X should map with exactly one context in the set Y** (this is guaranteed by construction, because X = Y). If this were the case, the Fowlkes-Mallow similarity value would be 1 (exact matches), which is intuitively correct.

However, in the absence of additional information, each context stemming from context 1 in X will map to **both** context 1 contexts in set Y. Algorithmically, this creates the illusion of a disagreement in the set, and will result in a Fowlkes-Mallow similarity value of less than 1.

The problem can be avoided with careful construction of the context set. In the context set window, checking the box titled **'Single Organism Amalgamation'** forces all genomic features from the same organism into the same context. The **MultipleQuery** search approach guarantees that at most one context is supplied, per organism. Similarly, the **Cassette** approach, which extends an existing

approach and forces the results into the same organism will avoid the problem. Please see the **Context Set** section for more information (page 68).

Tree Similarity Scan (⌘4)

A Tree Similarity Scan compares the grouping of data into a tree one way with the grouping of the same (or mostly the same) data into a tree another way.

The reference tree may be either (1) a JCE-computed context tree (for which you are invited to supply a query) or (2) an externally computed and imported phylogenetic tree. Please be aware of certain caveats in comparing data against a phylogenetic tree (see the "Comparing Against a Phylogenetic Tree" section below).

Input trees are first reduced to non-overlapping sets of clusters using an Adjusted Fowlkes-Mallows method, with a user-provided segmentation point and associated dataset adjustment parameters. Aside from the initial step of supplying a tree, all processes are identical to that of the Data Grouping Correlation (described in the previous section).

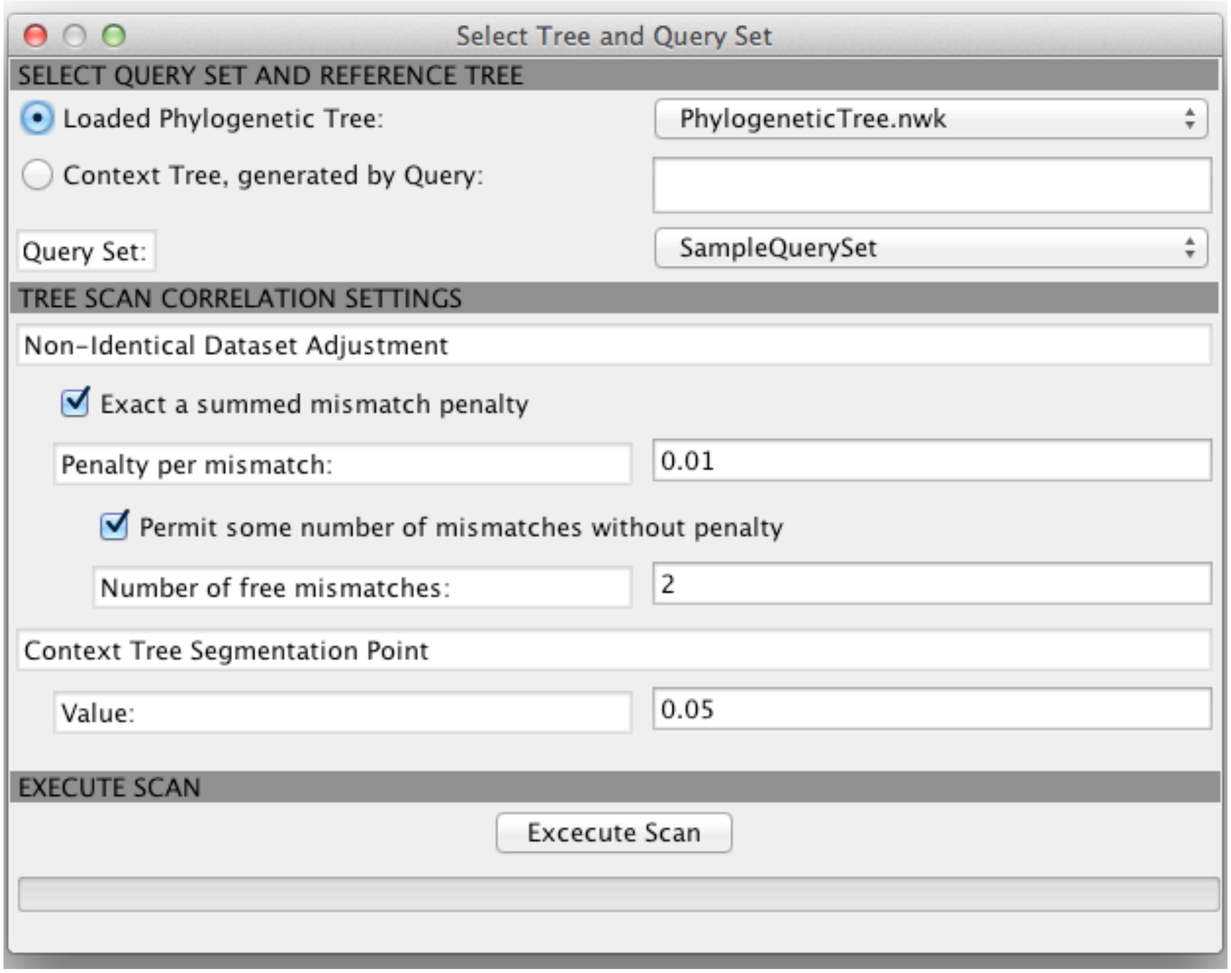

Select either the **Loaded Phylogenetic Tree** or **Context Tree, generated by Query** radio button. If there are no phylogenetic trees currently loaded, this option will be disabled. When you enter a query, a context tree will be generated **using the current context tree generation settings, as they appear in the main window.** This means the selected Context Set, Dissimilarity measure, and Clustering Algorithm. Note that these settings may differ with the settings used to generate the Query Set. Please ensure that the analysis makes sense, given the settings used in the Query Set and the settings used here.

DataSet adjustment parameters are explained in more detail in the previous section, "**Data Grouping Correlation**".

Comparing Against a Phylogenetic Tree

Depending on the format of the phylogenetic tree loaded, there may be some confusion in the parsing of the tree.

Phylogenetic trees will be divided into non-overlapping clusters by “cutting” the tree at a designated segmentation value. **Leaves that terminate at a tree height higher than the segmentation value will all be parsed as single clusters.**

For example, in the phylogenetic tree shown below, the tree is segmented into clusters at using a segmentation value of 0.06 (red line). Leaves are grouped into non-overlapping clusters as shown by blue boxes (only 2 examples shown). Branches that terminate at a tree height higher than the segmentation point are all considered to be single clusters (turquoise boxes).

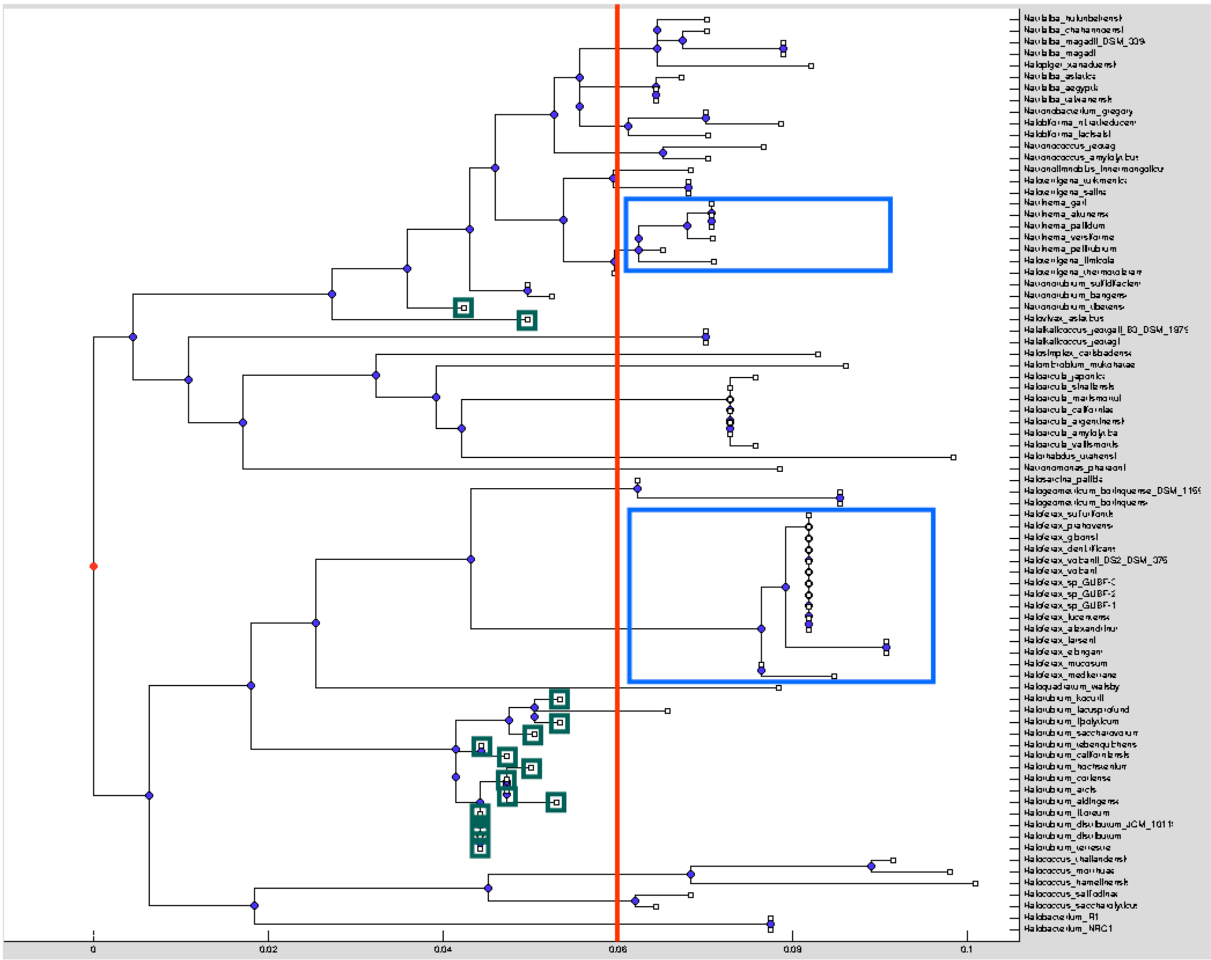

We recommend that all phylogenetic trees be constructed in such a way that cutting a tree at a particular segmentation value makes biological sense. Certain phylograms that utilize branch length as a means to convey evolutionary time may be inappropriate for this analysis.

Please format all phylogenetic trees into a form where such segmentation line divisions are appropriate.

Context Forest (⌘5)

Just as a forest is made of trees, so is a context forest made of context trees. An individual context tree is the product of variable group agglomerative hierarchical clustering applied to sets of genes across a number of closely related species – in that case, the **leaves of the tree are genomic groupings (set of genes)**. A context forest applies is the product of variable group agglomerative hierarchical clustering applied to context trees – in this case, **leaves of the tree are context trees**.

A Context Forest compares every context tree with every other context tree, and groups similar context trees together. Unlike the **Data Grouping** comparison and **Tree Similarity Scan** approaches, it is not necessary to have any idea what grouping patterns are interesting within a genome set – the Context Forest will discover trends in the topologies of a set of context trees without prior knowledge.

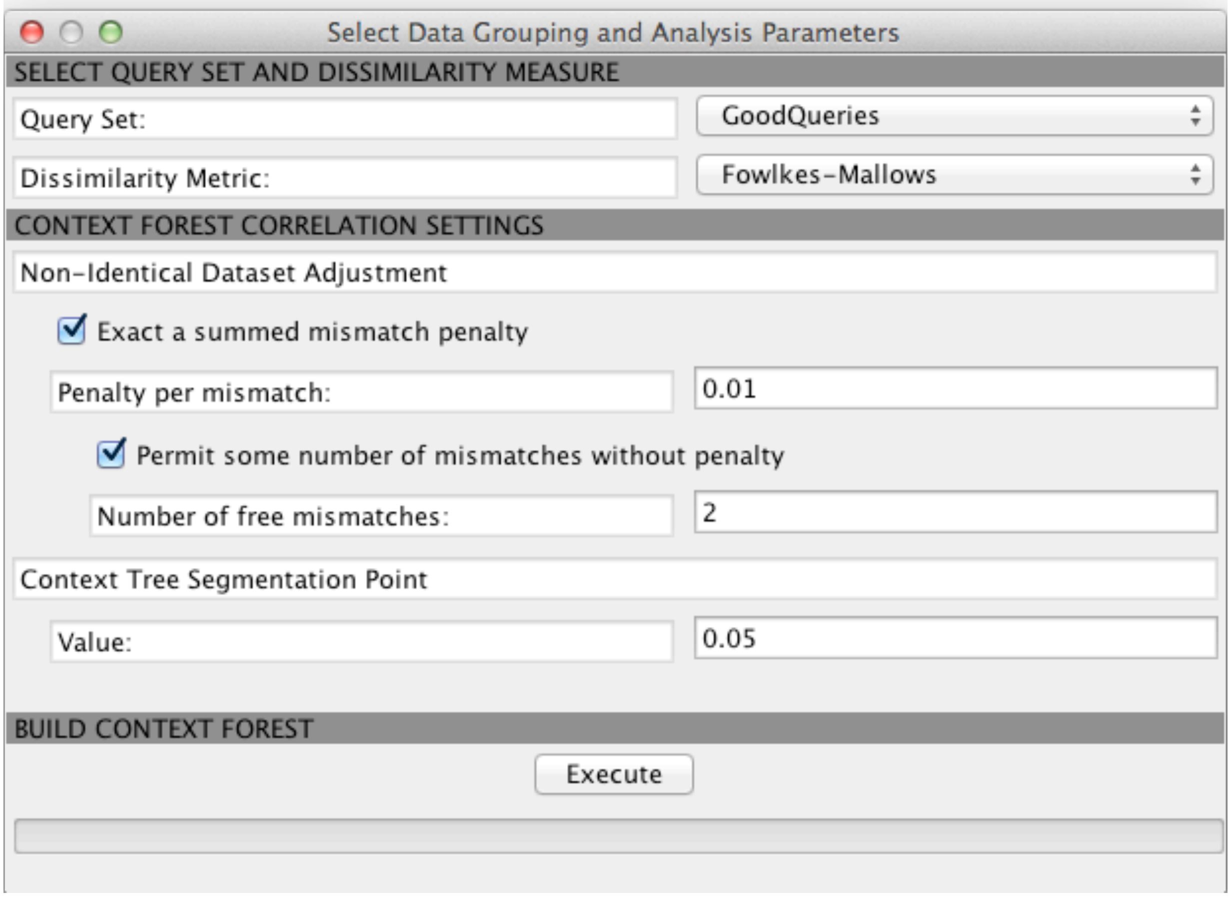

Under the banner **Select Query Set and Dissimilarity Measure,** select your query set and inter-tree dissimilarity measure (at present, only the **Adjusted Fowlkes-Mallows** method is available). Under **Context Forest Correlation Settings,** set non-identical dataset. Hitting the "**Execute**" button will build the context tree.

For a detailed description of the **Adjusted Fowlkes-Mallows** method, please see page 115.

Process Output Window

After carrying out a **Data Grouping Correlation**, **Tree Similarity Scan**, or building a **Context Forest**, a new window will appear, showing the results. This window may be closed without losing the underlying results, which will be stored with the all other information associated with the genome set. Only if a query set is removed will the results of individual process runs be lost.

If the process run is a **Data Grouping Correlation** or **Tree Similarity Scan**, the output window will be a **Scan Results Panel**. If the process run is building a context forest, the output window will be a **Context Forest Panel.**

Scan Results Panel

The **Scan Results Panel** appears after a **Query Set** has been compared to either a reference **Data Grouping** or **Reference Tree**. In either case, each Query in the Query Set is compared to the Reference grouping, and a numerical similarity value is assigned ranging between 0 and 1.

After a scan, the panel will appear:

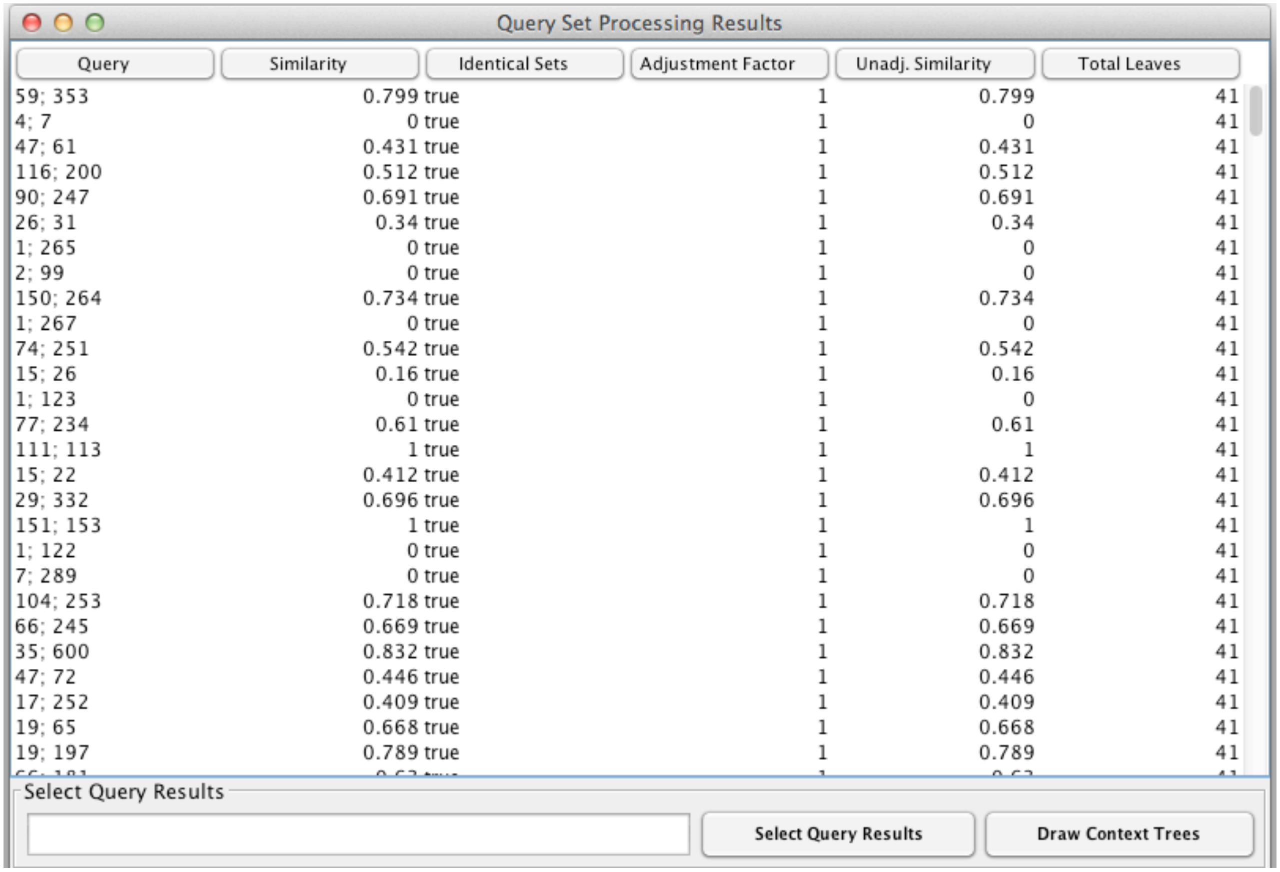

Query	Similarity	Identical Sets	Adjustment Factor	Unadj. Similarity	Total Leaves
59; 353	0.799	true	1	0.799	41
4; 7	0	true	1	0	41
47; 61	0.431	true	1	0.431	41
116; 200	0.512	true	1	0.512	41
90; 247	0.691	true	1	0.691	41
26; 31	0.34	true	1	0.34	41
1; 265	0	true	1	0	41
2; 99	0	true	1	0	41
150; 264	0.734	true	1	0.734	41
1; 267	0	true	1	0	41
74; 251	0.542	true	1	0.542	41
15; 26	0.16	true	1	0.16	41
1; 123	0	true	1	0	41
77; 234	0.61	true	1	0.61	41
111; 113	1	true	1	1	41
15; 22	0.412	true	1	0.412	41
29; 332	0.696	true	1	0.696	41
151; 153	1	true	1	1	41
1; 122	0	true	1	0	41
7; 289	0	true	1	0	41
104; 253	0.718	true	1	0.718	41
66; 245	0.669	true	1	0.669	41
35; 600	0.832	true	1	0.832	41
47; 72	0.446	true	1	0.446	41
17; 252	0.409	true	1	0.409	41
19; 65	0.668	true	1	0.668	41
19; 197	0.789	true	1	0.789	41
[illegible]	[illegible]	[illegible]	[illegible]	[illegible]	[illegible]

The top part of the frame is a table displaying the results.

In that table, the columns are as follows:

Query: The original query. Note that parameters such as the dissimilarity measure and the context set are not shown here, as these measures are constant for all members in this query set.

Similarity: Computed similarity between the context tree deriving from the query to the reference tree or data grouping.

Identical Sets: If the value is "true", then the context set generated by this query has exactly the same number and types of source genomes as the reference tree or reference data grouping. For example, if the reference set includes one genomic grouping each from genomes A, B, and C, and the query set includes one genomic grouping each from genomes A, B, and C, the sets are identical. If the query set instead includes one genomic grouping each from A, B, C, and D, the query set and the reference set are not identical sets.

Note: If there is a difference in the number of genomic groupings from a particular genome between the reference set and the query set, the two are not identical.

For example, if the query set includes two genomic groupings from A and one from B and C, and the query set includes only one genomic grouping from A (as well as one from B and one from C), the query set and the reference set are not identical sets.

Adjustment Factor: Numerical scale value describing the similarity between the reference tree / reference data grouping data set and this particular query set's data set. the Overall dissimilarity is computed by multiplying unadjusted dissimilarity by this value.

Unadj. Dissimilarity: The dissimilarity, if no adjustment penalty is exacted for the two datasets being non-identical. **Note:** in the **Fowlkes-Mallows** method, a penalty will still be exacted when there is a disparity in the number of genomic groupings deriving from the same genome.

The table may be sorted based on any column, by pushing the button above that column. Pushing the button once causes a downward facing arrow to appear on the button, which sorts the rows in ascending order according to the column selected. Pushing the button again causes an upward facing arrow to appear on the button, which sorts the rows in descending order. Pushing a different column's header button re-sorts the table according to that quality.

The **Query** and **Identical Sets** columns are sorted alphabetically or reverse-alphabetically, all other columns are treated as numerical values and sorted appropriately.

Clicking on a row selects that row, as does **Shift+Clicking** and **Ctrl+Clicking** (or **Cmd+Clicking** if working on a Mac). Rows may also be scrolled upwards and downwards using the arrow keys.

Rows may also be selected by typing in a string fragment of a query of interest in the **Search Bar.** All queries that match part of the string fragment will be selected. Both striking the enter key while the cursor is in the search bar and pushing the **Select Query Results** accomplish the same thing.

Pushing the **Draw Context Trees** button will draw the context trees for the selected rows in the table. Note that all drawing settings from the main frame will be used in rendering the trees. If the **Print Search Results** check box is selected in the main frame, this frame will be rendered along with the context trees.

Context Forest Panel

The **Context Forest Panel** appears after a context forest has been created from a **Query Set** – every context tree associated with each query in the query set has been compared to every other context tree, and the results have been amalgamated into a tree using variable-group agglomerative hierarchical clustering. Note that each leaf on the tree represents a context tree.

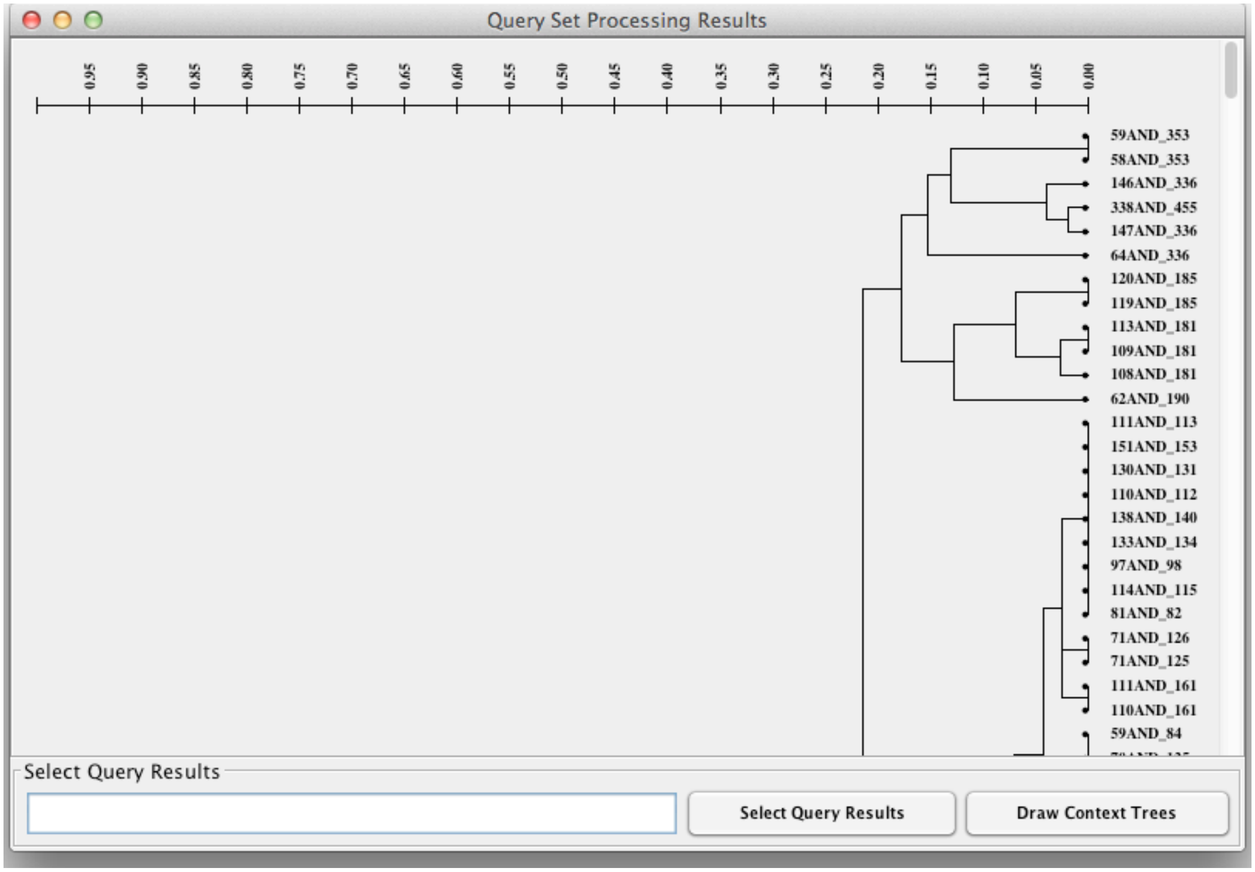

This tree looks and feels very much like the context trees associated with internal frames. Tree nodes may be selected in the same way. Please see **Internal Frame Management Area** on page 22 for more information.

Pushing the **Draw Context Trees** button will draw the context trees for the selected rows in the table. Note that all drawing settings from the main frame will be used in rendering the trees. If the **Print Search Results** check box is selected in the main frame, this frame will be rendered along with the context trees.

Help Menu

Help is available! While this **User's Manual** is the chief form of help, you may also check out the **Video Tutorials** (available at http://www.youtube.com/user/jcontextexplorer?feature=results_main) and view the associated scientific publication in a web browser by clicking **View Publication** (available at http://www.biomedcentral.com/1471-2105/14/18). If you are working on a mac, then there will be an additional **Search bar**, which will search for menu items with matching text.

A window displaying citation information for JContextExplorer will appear when selecting the **Show Citation**. The citation is

Seitzer, P., Huynh, T. A., & Facciotti, M. T. (2013). JContextExplorer: a tree-based approach to facilitate cross-species genomic context comparison. *BMC bioinformatics*, *14*(1), 18. BMC Bioinformatics. doi:10.1186/1471-2105-14-18

The Help Menu may be selected from the main menu bar, and when expanded looks like this:

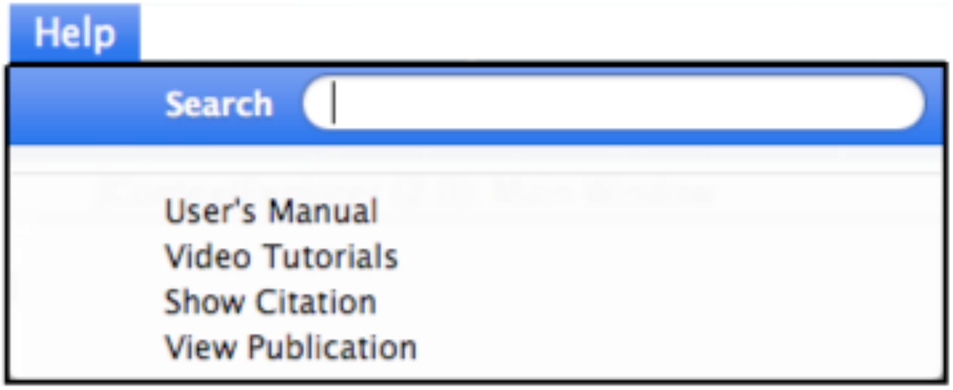

CHAPTER 4: ADDITIONAL RESOURCES

VIDEO TUTORIALS

Several video tutorials are publically available on youtube. To visit the video page, please navigate to

http://www.youtube.com/user/jcontextexplorer?feature=results_main

Author Contact Information

The chief author of this manual and software is Phillip Seitzer. He can be reached by email at pmseitzer@ucdavis.edu

Phillip Seitzer is a member of the Facciotti lab at the University of California at Davis:

http://www.bme.ucdavis.edu/facciotti/

The source code for JContextExplorer is hosted on GitHub:

https://github.com/PMSeitzer/JContextExplorer

Please do not hesitate to contact the author with questions, comments, bug reports, feature requests, and more.
