# Supplementary material for: The Exploration of Novel Regulatory Relationships Drives Haloarchaeal Operon-Like Structural Dynamics over Short Evolutionary Distances
Source: Microorganisms. 2020 Nov 30;8(12):1900. doi: 10.3390/microorganisms8121900 (PMC7760734; doi:10.3390/microorganisms8121900)
Supplement: Supplementary file 1 [file microorganisms-08-01900-s001.zip › S4_Co-occurringGenePairsQuerySet.rtf]

1 $$ 2771 $$ 3911 $$ 4001 $$ 4561 $$ 4661 $$ 5131 $$ 6031 $$ 6601 $$ 7371 $$ 7881 $$ 9391 $$ 9931 $$ 10051 $$ 12521 $$ 13371 $$ 13551 $$ 13661 $$ 13961 $$ 15551 $$ 17211 $$ 17231 $$ 17461 $$ 17521 $$ 20371 $$ 22021 $$ 22141 $$ 23181 $$ 23371 $$ 23491 $$ 23601 $$ 24621 $$ 24791 $$ 26151 $$ 27411 $$ 28381 $$ 28811 $$ 28921 $$ 29891 $$ 32581 $$ 33021 $$ 34381 $$ 43031 $$ 51531 $$ 51782 $$ 3282 $$ 3392 $$ 4152 $$ 4652 $$ 9672 $$ 10122 $$ 10532 $$ 10812 $$ 10922 $$ 11802 $$ 12832 $$ 13122 $$ 13332 $$ 13822 $$ 15172 $$ 16562 $$ 17802 $$ 18152 $$ 18292 $$ 18422 $$ 18472 $$ 19352 $$ 19932 $$ 20202 $$ 20562 $$ 21032 $$ 21192 $$ 22732 $$ 22742 $$ 23092 $$ 23522 $$ 23942 $$ 24462 $$ 24522 $$ 24602 $$ 24672 $$ 25032 $$ 25722 $$ 25862 $$ 26402 $$ 26412 $$ 28242 $$ 28952 $$ 28962 $$ 28972 $$ 29052 $$ 29382 $$ 29422 $$ 29702 $$ 30292 $$ 30302 $$ 30362 $$ 31092 $$ 31462 $$ 34172 $$ 35342 $$ 37102 $$ 37132 $$ 37592 $$ 38222 $$ 38472 $$ 42632 $$ 43312 $$ 43982 $$ 44522 $$ 44862 $$ 44872 $$ 44882 $$ 45182 $$ 45902 $$ 47182 $$ 47652 $$ 48702 $$ 49873 $$ 2793 $$ 2823 $$ 2993 $$ 3093 $$ 3313 $$ 4843 $$ 4953 $$ 5863 $$ 8223 $$ 10543 $$ 11683 $$ 12513 $$ 12833 $$ 13153 $$ 13823 $$ 13993 $$ 14403 $$ 14933 $$ 15303 $$ 16063 $$ 16183 $$ 16273 $$ 16303 $$ 20953 $$ 21713 $$ 21783 $$ 23793 $$ 24153 $$ 24653 $$ 25603 $$ 25933 $$ 29493 $$ 31153 $$ 32023 $$ 32553 $$ 32833 $$ 33603 $$ 47183 $$ 47353 $$ 49874 $$ 2804 $$ 3334 $$ 7944 $$ 8944 $$ 11204 $$ 11384 $$ 14034 $$ 15614 $$ 25554 $$ 28454 $$ 30414 $$ 32045 $$ 2985 $$ 16115 $$ 17295 $$ 31155 $$ 42966 $$ 2766 $$ 3676 $$ 3726 $$ 3816 $$ 3916 $$ 4816 $$ 5106 $$ 6546 $$ 7706 $$ 7926 $$ 10616 $$ 10746 $$ 10886 $$ 12896 $$ 14306 $$ 16336 $$ 16346 $$ 16476 $$ 16576 $$ 16606 $$ 16736 $$ 16886 $$ 18156 $$ 18326 $$ 18496 $$ 18726 $$ 20346 $$ 22286 $$ 22506 $$ 22846 $$ 24516 $$ 24526 $$ 24676 $$ 25036 $$ 25246 $$ 25326 $$ 26426 $$ 30356 $$ 37106 $$ 39776 $$ 42836 $$ 45277 $$ 2797 $$ 3047 $$ 3227 $$ 3297 $$ 3757 $$ 4187 $$ 4657 $$ 4997 $$ 5387 $$ 6747 $$ 9467 $$ 10917 $$ 11717 $$ 11837 $$ 12227 $$ 12577 $$ 12677 $$ 13237 $$ 15717 $$ 18987 $$ 21597 $$ 21997 $$ 22247 $$ 22327 $$ 22677 $$ 24177 $$ 24467 $$ 24607 $$ 25277 $$ 25867 $$ 29097 $$ 29417 $$ 30037 $$ 31367 $$ 31627 $$ 32557 $$ 33357 $$ 33437 $$ 34337 $$ 34927 $$ 36537 $$ 37297 $$ 37307 $$ 41587 $$ 50077 $$ 51658 $$ 3488 $$ 4648 $$ 4808 $$ 6178 $$ 7988 $$ 9668 $$ 12638 $$ 16098 $$ 22148 $$ 48399 $$ 5619 $$ 13119 $$ 202510 $$ 65010 $$ 73710 $$ 86310 $$ 133110 $$ 134410 $$ 146110 $$ 160210 $$ 171810 $$ 180410 $$ 182010 $$ 182810 $$ 188810 $$ 205210 $$ 228510 $$ 291210 $$ 327810 $$ 343110 $$ 358010 $$ 358110 $$ 363210 $$ 415510 $$ 516910 $$ 520411 $$ 54411 $$ 56311 $$ 69111 $$ 77611 $$ 122311 $$ 128311 $$ 132211 $$ 134211 $$ 140311 $$ 143811 $$ 152811 $$ 255511 $$ 261111 $$ 272111 $$ 273611 $$ 289511 $$ 289611 $$ 289711 $$ 294711 $$ 359111 $$ 401211 $$ 471811 $$ 498711 $$ 501212 $$ 27812 $$ 29812 $$ 30612 $$ 33912 $$ 41312 $$ 58312 $$ 58912 $$ 80312 $$ 80412 $$ 97012 $$ 133812 $$ 136512 $$ 137212 $$ 155212 $$ 157412 $$ 165912 $$ 167812 $$ 168412 $$ 182612 $$ 185312 $$ 187712 $$ 213312 $$ 215412 $$ 221112 $$ 236212 $$ 248712 $$ 253512 $$ 260212 $$ 268912 $$ 275612 $$ 275712 $$ 283712 $$ 290412 $$ 320212 $$ 320612 $$ 363112 $$ 380012 $$ 434512 $$ 488812 $$ 490012 $$ 490112 $$ 490212 $$ 491312 $$ 521612 $$ 521813 $$ 29813 $$ 30613 $$ 33913 $$ 41313 $$ 58913 $$ 80313 $$ 80413 $$ 97013 $$ 133813 $$ 155213 $$ 157413 $$ 167813 $$ 168413 $$ 182613 $$ 185313 $$ 213313 $$ 221113 $$ 236213 $$ 248713 $$ 253513 $$ 260213 $$ 268913 $$ 275613 $$ 275713 $$ 290413 $$ 320213 $$ 363113 $$ 380013 $$ 415613 $$ 434513 $$ 488813 $$ 490013 $$ 490113 $$ 490213 $$ 491313 $$ 521613 $$ 521814 $$ 35014 $$ 288314 $$ 306215 $$ 30915 $$ 36415 $$ 41315 $$ 60115 $$ 163215 $$ 177415 $$ 183215 $$ 425416 $$ 33816 $$ 36616 $$ 40516 $$ 67316 $$ 86716 $$ 87116 $$ 212916 $$ 222816 $$ 251716 $$ 346216 $$ 346316 $$ 396216 $$ 412016 $$ 423516 $$ 489917 $$ 112017 $$ 124017 $$ 199717 $$ 207917 $$ 247317 $$ 371518 $$ 33018 $$ 70118 $$ 70218 $$ 88818 $$ 90418 $$ 93018 $$ 105718 $$ 107518 $$ 118518 $$ 166218 $$ 228518 $$ 228618 $$ 245018 $$ 289518 $$ 289618 $$ 289718 $$ 298518 $$ 317118 $$ 348718 $$ 458318 $$ 470618 $$ 484719 $$ 31019 $$ 52419 $$ 68419 $$ 68519 $$ 248719 $$ 276520 $$ 37120 $$ 41420 $$ 42920 $$ 46020 $$ 116920 $$ 127620 $$ 128320 $$ 135520 $$ 148120 $$ 159520 $$ 163620 $$ 172620 $$ 178120 $$ 182920 $$ 188720 $$ 210320 $$ 219420 $$ 237920 $$ 254820 $$ 362821 $$ 41721 $$ 65421 $$ 97421 $$ 163321 $$ 163421 $$ 163921 $$ 167721 $$ 196821 $$ 214221 $$ 224321 $$ 310221 $$ 328521 $$ 391522 $$ 74522 $$ 92122 $$ 111322 $$ 327523 $$ 31223 $$ 101423 $$ 103323 $$ 106823 $$ 111523 $$ 125523 $$ 166923 $$ 178723 $$ 214623 $$ 306323 $$ 328123 $$ 355124 $$ 35724 $$ 43024 $$ 54724 $$ 81824 $$ 81924 $$ 132224 $$ 155324 $$ 159324 $$ 183724 $$ 185524 $$ 187624 $$ 215824 $$ 220024 $$ 232824 $$ 253924 $$ 282624 $$ 307524 $$ 333924 $$ 361624 $$ 481124 $$ 489625 $$ 45625 $$ 79425 $$ 86925 $$ 91725 $$ 104525 $$ 142325 $$ 172625 $$ 174625 $$ 184925 $$ 232225 $$ 232325 $$ 271326 $$ 33326 $$ 34126 $$ 35826 $$ 44326 $$ 113826 $$ 163626 $$ 460226 $$ 473526 $$ 508426 $$ 521527 $$ 94127 $$ 101827 $$ 113927 $$ 120427 $$ 152527 $$ 189727 $$ 194227 $$ 197527 $$ 197627 $$ 200327 $$ 218227 $$ 341827 $$ 421227 $$ 459628 $$ 27628 $$ 36728 $$ 53028 $$ 57228 $$ 131228 $$ 131528 $$ 138428 $$ 154528 $$ 162428 $$ 177528 $$ 182728 $$ 190128 $$ 203428 $$ 229928 $$ 230028 $$ 230128 $$ 264028 $$ 264128 $$ 399228 $$ 399328 $$ 413729 $$ 102729 $$ 127129 $$ 131329 $$ 131429 $$ 140429 $$ 147429 $$ 194029 $$ 221929 $$ 257629 $$ 257729 $$ 265629 $$ 295229 $$ 319529 $$ 324129 $$ 334429 $$ 344429 $$ 432829 $$ 458629 $$ 510330 $$ 48930 $$ 63230 $$ 79530 $$ 84830 $$ 105830 $$ 166630 $$ 182530 $$ 231130 $$ 299730 $$ 438231 $$ 27831 $$ 29831 $$ 30631 $$ 33931 $$ 41331 $$ 58331 $$ 58931 $$ 133831 $$ 136531 $$ 165931 $$ 185331 $$ 187731 $$ 215431 $$ 221131 $$ 248731 $$ 253531 $$ 260231 $$ 268931 $$ 275631 $$ 275731 $$ 283731 $$ 290431 $$ 320231 $$ 320631 $$ 363131 $$ 488831 $$ 490031 $$ 490131 $$ 490231 $$ 491331 $$ 521631 $$ 521832 $$ 29132 $$ 35032 $$ 85232 $$ 150832 $$ 154032 $$ 193932 $$ 225332 $$ 233032 $$ 501433 $$ 58233 $$ 86333 $$ 151133 $$ 191533 $$ 230433 $$ 286233 $$ 384034 $$ 41634 $$ 162534 $$ 206934 $$ 210534 $$ 443735 $$ 38935 $$ 322736 $$ 45336 $$ 147836 $$ 178837 $$ 28337 $$ 31737 $$ 92337 $$ 139537 $$ 155937 $$ 171937 $$ 195937 $$ 274937 $$ 290437 $$ 320637 $$ 505738 $$ 27338 $$ 27438 $$ 35338 $$ 38438 $$ 107338 $$ 115638 $$ 235638 $$ 269539 $$ 53639 $$ 56539 $$ 56639 $$ 79539 $$ 94639 $$ 96439 $$ 196139 $$ 202339 $$ 205739 $$ 208939 $$ 213139 $$ 282839 $$ 309039 $$ 320639 $$ 331139 $$ 428639 $$ 472939 $$ 515939 $$ 520739 $$ 523340 $$ 27840 $$ 43040 $$ 178240 $$ 203140 $$ 223240 $$ 234040 $$ 254040 $$ 427141 $$ 46941 $$ 53741 $$ 143841 $$ 331142 $$ 31542 $$ 35042 $$ 78542 $$ 78642 $$ 196142 $$ 202342 $$ 205742 $$ 208942 $$ 213142 $$ 375243 $$ 85943 $$ 101143 $$ 160843 $$ 164143 $$ 203743 $$ 215343 $$ 239043 $$ 245343 $$ 305843 $$ 360244 $$ 57144 $$ 60244 $$ 66444 $$ 111144 $$ 121344 $$ 123544 $$ 149944 $$ 166344 $$ 221344 $$ 488545 $$ 58945 $$ 80345 $$ 80445 $$ 97045 $$ 157445 $$ 167845 $$ 168445 $$ 182645 $$ 185345 $$ 213345 $$ 248745 $$ 260245 $$ 275745 $$ 363145 $$ 380045 $$ 415645 $$ 434546 $$ 142046 $$ 202747 $$ 56447 $$ 299547 $$ 299647 $$ 305547 $$ 332747 $$ 344947 $$ 359947 $$ 396947 $$ 502748 $$ 30448 $$ 31448 $$ 32348 $$ 36448 $$ 38948 $$ 103548 $$ 113448 $$ 126548 $$ 141848 $$ 160048 $$ 196548 $$ 222748 $$ 269748 $$ 305248 $$ 467948 $$ 474748 $$ 516149 $$ 27949 $$ 37549 $$ 79049 $$ 138049 $$ 144249 $$ 178149 $$ 185049 $$ 236949 $$ 342249 $$ 371950 $$ 27650 $$ 87350 $$ 199850 $$ 203750 $$ 210650 $$ 264950 $$ 379151 $$ 42051 $$ 184451 $$ 189451 $$ 216251 $$ 218651 $$ 350051 $$ 350152 $$ 43452 $$ 185052 $$ 199652 $$ 489553 $$ 31653 $$ 33153 $$ 33253 $$ 67753 $$ 111153 $$ 112253 $$ 402554 $$ 58554 $$ 117354 $$ 190654 $$ 196854 $$ 214254 $$ 224354 $$ 233154 $$ 277655 $$ 29955 $$ 56755 $$ 79655 $$ 110355 $$ 155955 $$ 282755 $$ 526256 $$ 94556 $$ 114256 $$ 115156 $$ 471757 $$ 27957 $$ 37557 $$ 41857 $$ 49957 $$ 53857 $$ 215957 $$ 222457 $$ 274957 $$ 316257 $$ 325557 $$ 334157 $$ 378058 $$ 75058 $$ 103158 $$ 114858 $$ 133058 $$ 167358 $$ 185658 $$ 191258 $$ 285359 $$ 37559 $$ 130059 $$ 259859 $$ 274959 $$ 293659 $$ 294659 $$ 303759 $$ 371459 $$ 393259 $$ 424260 $$ 28860 $$ 249660 $$ 266160 $$ 272760 $$ 285560 $$ 360661 $$ 27561 $$ 27861 $$ 30461 $$ 31461 $$ 36461 $$ 49961 $$ 116061 $$ 153461 $$ 159861 $$ 165961 $$ 229261 $$ 255561 $$ 283761 $$ 290961 $$ 313661 $$ 339061 $$ 342761 $$ 343761 $$ 353061 $$ 356961 $$ 385761 $$ 415861 $$ 473061 $$ 503662 $$ 35862 $$ 79462 $$ 111462 $$ 146262 $$ 261862 $$ 274062 $$ 303862 $$ 362962 $$ 438762 $$ 512062 $$ 517663 $$ 27363 $$ 27463 $$ 107363 $$ 115663 $$ 235663 $$ 287763 $$ 356763 $$ 418163 $$ 466964 $$ 28364 $$ 94364 $$ 109964 $$ 128264 $$ 162064 $$ 165564 $$ 171664 $$ 276264 $$ 279464 $$ 312464 $$ 317964 $$ 362664 $$ 362764 $$ 382264 $$ 433164 $$ 499465 $$ 34665 $$ 46265 $$ 144765 $$ 159865 $$ 160565 $$ 276765 $$ 344665 $$ 352866 $$ 52766 $$ 81366 $$ 223266 $$ 229866 $$ 254066 $$ 345866 $$ 400267 $$ 210067 $$ 248467 $$ 248567 $$ 290668 $$ 29968 $$ 56768 $$ 79668 $$ 240768 $$ 246668 $$ 282769 $$ 161169 $$ 228669 $$ 303769 $$ 371470 $$ 93470 $$ 93570 $$ 179170 $$ 252070 $$ 270971 $$ 103471 $$ 138771 $$ 168571 $$ 213271 $$ 276271 $$ 312472 $$ 36872 $$ 79672 $$ 177972 $$ 246172 $$ 474373 $$ 225073 $$ 314473 $$ 376974 $$ 29874 $$ 81274 $$ 101474 $$ 111574 $$ 174574 $$ 268974 $$ 275174 $$ 320274 $$ 451774 $$ 492574 $$ 516975 $$ 52675 $$ 78475 $$ 127775 $$ 170175 $$ 208475 $$ 342376 $$ 50676 $$ 94576 $$ 104976 $$ 114276 $$ 136577 $$ 112677 $$ 180177 $$ 182377 $$ 189377 $$ 219478 $$ 99378 $$ 109078 $$ 175778 $$ 276179 $$ 157679 $$ 298880 $$ 27780 $$ 29080 $$ 36780 $$ 38980 $$ 40480 $$ 115980 $$ 122880 $$ 177580 $$ 191380 $$ 230980 $$ 235280 $$ 257280 $$ 382280 $$ 383080 $$ 433180 $$ 471081 $$ 41481 $$ 50881 $$ 60681 $$ 61281 $$ 61381 $$ 116981 $$ 125981 $$ 129681 $$ 284681 $$ 385881 $$ 503982 $$ 116682 $$ 234783 $$ 27683 $$ 36783 $$ 49083 $$ 65983 $$ 131583 $$ 145483 $$ 154583 $$ 162483 $$ 177583 $$ 178783 $$ 182783 $$ 203483 $$ 228283 $$ 475984 $$ 189484 $$ 216284 $$ 218684 $$ 236885 $$ 142785 $$ 371986 $$ 28686 $$ 55786 $$ 106386 $$ 107586 $$ 112086 $$ 115086 $$ 130386 $$ 138086 $$ 144286 $$ 156086 $$ 174886 $$ 228086 $$ 236886 $$ 330886 $$ 330986 $$ 391787 $$ 28387 $$ 56887 $$ 94387 $$ 142088 $$ 36288 $$ 43588 $$ 63188 $$ 123988 $$ 156688 $$ 156788 $$ 158688 $$ 307788 $$ 332889 $$ 90589 $$ 118889 $$ 133089 $$ 167989 $$ 336990 $$ 29890 $$ 106490 $$ 167990 $$ 213490 $$ 478991 $$ 65491 $$ 106191 $$ 225091 $$ 252491 $$ 371092 $$ 47092 $$ 50293 $$ 57293 $$ 73693 $$ 80193 $$ 91093 $$ 104393 $$ 108293 $$ 138494 $$ 27894 $$ 165994 $$ 283794 $$ 292494 $$ 414395 $$ 36495 $$ 120395 $$ 136995 $$ 192195 $$ 221395 $$ 238295 $$ 244595 $$ 284496 $$ 33496 $$ 37496 $$ 45296 $$ 47696 $$ 53196 $$ 73896 $$ 73996 $$ 74096 $$ 91496 $$ 109796 $$ 122996 $$ 123096 $$ 209696 $$ 234396 $$ 503697 $$ 174297 $$ 270297 $$ 503498 $$ 76998 $$ 77698 $$ 196798 $$ 319499 $$ 86399 $$ 119999 $$ 142499 $$ 157799 $$ 208299 $$ 209499 $$ 420099 $$ 501499 $$ 5238100 $$ 845100 $$ 1191100 $$ 1636100 $$ 1726100 $$ 2019100 $$ 2194100 $$ 2944101 $$ 275101 $$ 1198101 $$ 2479102 $$ 474102 $$ 662103 $$ 278103 $$ 413103 $$ 583103 $$ 1338103 $$ 1365103 $$ 1552103 $$ 1659103 $$ 2362103 $$ 2756103 $$ 2837103 $$ 2904103 $$ 3206104 $$ 403104 $$ 1071104 $$ 2469104 $$ 2642104 $$ 3325104 $$ 3578104 $$ 3928105 $$ 406105 $$ 732105 $$ 2667105 $$ 4861106 $$ 942106 $$ 1015106 $$ 1116106 $$ 1188106 $$ 1266106 $$ 1585106 $$ 1692106 $$ 3323106 $$ 3324106 $$ 3349106 $$ 4017106 $$ 4172106 $$ 4830106 $$ 4831107 $$ 3198107 $$ 3964107 $$ 4322108 $$ 4300108 $$ 4567109 $$ 360109 $$ 977109 $$ 1763109 $$ 2974109 $$ 2975109 $$ 3058110 $$ 482110 $$ 1119110 $$ 1689110 $$ 1770110 $$ 1861110 $$ 2780110 $$ 2863110 $$ 3160111 $$ 295111 $$ 1038111 $$ 1267111 $$ 1508111 $$ 2182111 $$ 2373111 $$ 2775111 $$ 3152111 $$ 3425111 $$ 3457111 $$ 3628112 $$ 818112 $$ 844112 $$ 880112 $$ 1554112 $$ 1620112 $$ 1655112 $$ 1681112 $$ 3031113 $$ 392113 $$ 1649113 $$ 1650113 $$ 2291114 $$ 555114 $$ 4362115 $$ 830116 $$ 276116 $$ 299116 $$ 367116 $$ 1315116 $$ 1545116 $$ 1624116 $$ 1775116 $$ 1827116 $$ 2034116 $$ 5270117 $$ 415117 $$ 841118 $$ 1627118 $$ 2844118 $$ 3427118 $$ 3535120 $$ 298120 $$ 1611120 $$ 2134121 $$ 391121 $$ 1420121 $$ 2027121 $$ 3438122 $$ 1016122 $$ 1163122 $$ 1394122 $$ 2102122 $$ 2983123 $$ 655123 $$ 734123 $$ 1042123 $$ 1228123 $$ 1673123 $$ 1941124 $$ 1974125 $$ 1430125 $$ 1549125 $$ 1973125 $$ 3350126 $$ 306126 $$ 530126 $$ 609126 $$ 617126 $$ 724126 $$ 895126 $$ 1022126 $$ 1102126 $$ 1176126 $$ 1453126 $$ 1460126 $$ 1468126 $$ 1469126 $$ 2192126 $$ 2524127 $$ 796127 $$ 2590127 $$ 4958128 $$ 1135128 $$ 1528128 $$ 1870128 $$ 4584129 $$ 418129 $$ 538129 $$ 1595129 $$ 1730129 $$ 1968129 $$ 2142129 $$ 2159129 $$ 2243129 $$ 2668129 $$ 4073129 $$ 4405130 $$ 818130 $$ 1034130 $$ 1387130 $$ 1446130 $$ 1450130 $$ 1554130 $$ 1593130 $$ 1709130 $$ 1876130 $$ 1882130 $$ 2132130 $$ 2429130 $$ 3339130 $$ 4023130 $$ 4333130 $$ 4525130 $$ 4811130 $$ 5099131 $$ 451131 $$ 1951131 $$ 2147131 $$ 2836132 $$ 419132 $$ 444132 $$ 683132 $$ 878132 $$ 1029132 $$ 1030132 $$ 1125133 $$ 330133 $$ 930133 $$ 1051133 $$ 1052133 $$ 1196133 $$ 1295133 $$ 1455133 $$ 1685133 $$ 2418134 $$ 332134 $$ 677134 $$ 1269134 $$ 2901135 $$ 2244135 $$ 2301135 $$ 2326136 $$ 292136 $$ 720136 $$ 769136 $$ 838136 $$ 1300136 $$ 1524136 $$ 2512136 $$ 3035137 $$ 357137 $$ 1675137 $$ 2597137 $$ 2869137 $$ 4074140 $$ 430140 $$ 505140 $$ 1131140 $$ 2510140 $$ 2948141 $$ 452141 $$ 476141 $$ 531141 $$ 738141 $$ 739141 $$ 740141 $$ 914141 $$ 915141 $$ 1229141 $$ 1230141 $$ 1377141 $$ 2343141 $$ 2480141 $$ 3746142 $$ 1682143 $$ 1604143 $$ 1682144 $$ 788144 $$ 1140144 $$ 1815144 $$ 3612144 $$ 5121145 $$ 719145 $$ 756145 $$ 933145 $$ 1658146 $$ 471146 $$ 815146 $$ 1252146 $$ 1300146 $$ 1852146 $$ 1902146 $$ 4603147 $$ 407147 $$ 903147 $$ 1223147 $$ 1313147 $$ 1314148 $$ 319148 $$ 626148 $$ 627148 $$ 1000148 $$ 1188148 $$ 1261148 $$ 1324148 $$ 1454148 $$ 1665148 $$ 1693148 $$ 1713148 $$ 1717148 $$ 1790148 $$ 1794148 $$ 1834148 $$ 1839148 $$ 1977148 $$ 4746149 $$ 1040149 $$ 1779149 $$ 2792150 $$ 1387150 $$ 1882150 $$ 2705150 $$ 3043151 $$ 1507151 $$ 1714152 $$ 2353153 $$ 574153 $$ 1172153 $$ 1216153 $$ 1252153 $$ 1531153 $$ 1902153 $$ 2701153 $$ 4904154 $$ 1044154 $$ 1224154 $$ 1756154 $$ 2950154 $$ 3011154 $$ 5003154 $$ 5004155 $$ 375155 $$ 418155 $$ 538155 $$ 2142155 $$ 2159155 $$ 3855156 $$ 279157 $$ 1673157 $$ 1906157 $$ 2074157 $$ 2374157 $$ 2959158 $$ 684158 $$ 685158 $$ 856158 $$ 1282158 $$ 1317158 $$ 1589158 $$ 2755158 $$ 5203159 $$ 512159 $$ 4369159 $$ 4650159 $$ 4763160 $$ 295161 $$ 1012161 $$ 1104161 $$ 3508162 $$ 357162 $$ 818162 $$ 1322162 $$ 1446162 $$ 1450162 $$ 1554162 $$ 1882162 $$ 2132162 $$ 2399162 $$ 2693162 $$ 2705162 $$ 2759162 $$ 2925162 $$ 3043162 $$ 3354162 $$ 3576162 $$ 4732162 $$ 4822163 $$ 1041164 $$ 284164 $$ 749164 $$ 1401164 $$ 1859164 $$ 3230164 $$ 3824165 $$ 818165 $$ 1034165 $$ 1387165 $$ 1450165 $$ 1554165 $$ 1882165 $$ 2132165 $$ 2429165 $$ 4811166 $$ 380166 $$ 562166 $$ 1000166 $$ 1425166 $$ 2991166 $$ 3224166 $$ 3593166 $$ 3655166 $$ 4122167 $$ 341167 $$ 443167 $$ 513167 $$ 1355167 $$ 1481168 $$ 289170 $$ 1868170 $$ 1900170 $$ 1931171 $$ 396171 $$ 513171 $$ 716171 $$ 874171 $$ 890171 $$ 1187171 $$ 1345171 $$ 3214171 $$ 3353171 $$ 3772171 $$ 4047172 $$ 1936172 $$ 2095172 $$ 2164172 $$ 4619173 $$ 701173 $$ 702173 $$ 1278173 $$ 1284173 $$ 1651173 $$ 1663173 $$ 2698173 $$ 2861173 $$ 3383173 $$ 4174174 $$ 483174 $$ 1938174 $$ 1939174 $$ 2025174 $$ 2152174 $$ 2344175 $$ 977175 $$ 1170176 $$ 277176 $$ 894177 $$ 474177 $$ 774177 $$ 775177 $$ 1019177 $$ 1455177 $$ 1934178 $$ 339178 $$ 1106178 $$ 1751178 $$ 1760178 $$ 1863178 $$ 3168178 $$ 5166179 $$ 751179 $$ 752179 $$ 931179 $$ 1236179 $$ 1429179 $$ 1929180 $$ 304180 $$ 451180 $$ 607180 $$ 936180 $$ 1001180 $$ 1037180 $$ 1199180 $$ 1512180 $$ 1724180 $$ 1759180 $$ 2155180 $$ 2482180 $$ 2498180 $$ 2535180 $$ 4730181 $$ 344181 $$ 566181 $$ 1603181 $$ 1840181 $$ 2962181 $$ 4373181 $$ 5240182 $$ 287182 $$ 5097183 $$ 1312183 $$ 1935183 $$ 1993183 $$ 2905183 $$ 2970184 $$ 317184 $$ 478184 $$ 724184 $$ 1079184 $$ 1605184 $$ 1900184 $$ 1901184 $$ 2063184 $$ 3089184 $$ 3374184 $$ 3512185 $$ 317185 $$ 521185 $$ 522185 $$ 534185 $$ 810185 $$ 963185 $$ 975185 $$ 1089185 $$ 1165185 $$ 2218185 $$ 2246185 $$ 3418186 $$ 815186 $$ 1691186 $$ 4772187 $$ 327187 $$ 957187 $$ 2324187 $$ 3648187 $$ 3928187 $$ 4397187 $$ 4610188 $$ 2948189 $$ 928189 $$ 1962189 $$ 3870190 $$ 394192 $$ 433192 $$ 713192 $$ 714193 $$ 1762193 $$ 4018193 $$ 4657194 $$ 880194 $$ 881194 $$ 1606195 $$ 493195 $$ 1171195 $$ 1587195 $$ 3295195 $$ 3971196 $$ 280196 $$ 798196 $$ 815196 $$ 1342196 $$ 1930196 $$ 2697196 $$ 2895196 $$ 2896196 $$ 2897197 $$ 406197 $$ 732197 $$ 1765198 $$ 279198 $$ 1727198 $$ 1739198 $$ 3162198 $$ 3255198 $$ 3335198 $$ 3433198 $$ 3492199 $$ 403199 $$ 1802199 $$ 3325199 $$ 3928200 $$ 314200 $$ 787200 $$ 1448200 $$ 3390200 $$ 3427200 $$ 3530200 $$ 3535201 $$ 304201 $$ 451201 $$ 607201 $$ 936201 $$ 1001201 $$ 1037201 $$ 1199201 $$ 1512201 $$ 1724201 $$ 1759201 $$ 2155201 $$ 2482201 $$ 2498201 $$ 2535201 $$ 4730202 $$ 407202 $$ 1669202 $$ 3485202 $$ 4039203 $$ 672203 $$ 673203 $$ 796203 $$ 867203 $$ 1989203 $$ 2434203 $$ 3006204 $$ 609204 $$ 670204 $$ 830204 $$ 1426204 $$ 2026204 $$ 3503204 $$ 4552205 $$ 962205 $$ 1436206 $$ 327206 $$ 1193206 $$ 1769206 $$ 2498206 $$ 2952206 $$ 3195206 $$ 3263206 $$ 3648206 $$ 4076206 $$ 4397206 $$ 4586206 $$ 4599206 $$ 4610206 $$ 5044207 $$ 2046207 $$ 2048207 $$ 2161208 $$ 344208 $$ 974208 $$ 1840208 $$ 4373208 $$ 5240209 $$ 607209 $$ 747209 $$ 924209 $$ 1143209 $$ 1405209 $$ 2708210 $$ 651210 $$ 728210 $$ 1211210 $$ 1395210 $$ 1559210 $$ 2185210 $$ 2420210 $$ 4739210 $$ 5057211 $$ 753211 $$ 755211 $$ 1366211 $$ 1387211 $$ 2155211 $$ 2498211 $$ 3604211 $$ 4309212 $$ 390212 $$ 4107213 $$ 864213 $$ 1881213 $$ 3285214 $$ 651214 $$ 720214 $$ 1211214 $$ 1457214 $$ 1514214 $$ 1525214 $$ 1982214 $$ 2080214 $$ 2420214 $$ 2862214 $$ 3840215 $$ 581215 $$ 1682215 $$ 2247216 $$ 299216 $$ 567216 $$ 796218 $$ 852218 $$ 1929218 $$ 2330218 $$ 4277219 $$ 431219 $$ 840219 $$ 853219 $$ 1004219 $$ 2781220 $$ 760220 $$ 1235220 $$ 2453220 $$ 3058220 $$ 4077222 $$ 286222 $$ 545222 $$ 922222 $$ 934222 $$ 1301222 $$ 1416222 $$ 2811223 $$ 2430224 $$ 2597224 $$ 4600225 $$ 671225 $$ 968226 $$ 341226 $$ 443226 $$ 660226 $$ 1313226 $$ 1314227 $$ 347227 $$ 365227 $$ 418227 $$ 538227 $$ 2159229 $$ 331229 $$ 332229 $$ 726229 $$ 1393229 $$ 4025229 $$ 4114230 $$ 1182230 $$ 3317230 $$ 4820230 $$ 5123231 $$ 720231 $$ 2108231 $$ 2703231 $$ 3317231 $$ 4741232 $$ 2602233 $$ 276233 $$ 367233 $$ 393233 $$ 601233 $$ 1151233 $$ 1478233 $$ 1775233 $$ 1788233 $$ 2034233 $$ 2076235 $$ 309235 $$ 364236 $$ 357236 $$ 477236 $$ 547236 $$ 915236 $$ 1175236 $$ 1322236 $$ 1553236 $$ 1997236 $$ 2366236 $$ 2826236 $$ 4023237 $$ 604237 $$ 870237 $$ 1017237 $$ 1029237 $$ 3741238 $$ 619238 $$ 2299238 $$ 2300238 $$ 2301239 $$ 857239 $$ 1549239 $$ 1668239 $$ 2265239 $$ 3013240 $$ 542240 $$ 2923240 $$ 3601241 $$ 1232241 $$ 2353242 $$ 487242 $$ 1232242 $$ 1461242 $$ 3079242 $$ 3814243 $$ 337243 $$ 383243 $$ 959244 $$ 352244 $$ 449244 $$ 741244 $$ 865244 $$ 1598244 $$ 1708245 $$ 918245 $$ 1512245 $$ 1555245 $$ 1690245 $$ 1752246 $$ 328246 $$ 384246 $$ 445246 $$ 1340246 $$ 2592247 $$ 588247 $$ 607247 $$ 991247 $$ 1678247 $$ 2222248 $$ 348248 $$ 1117248 $$ 1809249 $$ 284249 $$ 749249 $$ 1401249 $$ 1859249 $$ 3824250 $$ 481250 $$ 510250 $$ 909250 $$ 4019250 $$ 4049250 $$ 4765251 $$ 1143251 $$ 4725252 $$ 355252 $$ 422252 $$ 605252 $$ 1408252 $$ 2259252 $$ 4611253 $$ 648253 $$ 852254 $$ 1589254 $$ 1864254 $$ 2335254 $$ 2380254 $$ 2439254 $$ 2478254 $$ 2552254 $$ 2814254 $$ 2843254 $$ 3053254 $$ 3222254 $$ 3321254 $$ 3442254 $$ 3443254 $$ 4140254 $$ 4451254 $$ 4528254 $$ 4749254 $$ 4880254 $$ 5032254 $$ 5033254 $$ 5194255 $$ 318255 $$ 1143255 $$ 1486255 $$ 1934255 $$ 2371255 $$ 2396255 $$ 3884257 $$ 834258 $$ 818258 $$ 1034258 $$ 1387258 $$ 1450258 $$ 1554258 $$ 1857258 $$ 1876258 $$ 1882258 $$ 2132258 $$ 2760258 $$ 4525259 $$ 3198259 $$ 5060260 $$ 912260 $$ 1276261 $$ 2795262 $$ 794262 $$ 1114262 $$ 2618263 $$ 405263 $$ 2129263 $$ 2228264 $$ 529264 $$ 776264 $$ 1216264 $$ 1320264 $$ 3126265 $$ 389265 $$ 1462265 $$ 2435266 $$ 925266 $$ 1565267 $$ 323267 $$ 932267 $$ 1863267 $$ 2053267 $$ 2164268 $$ 1068268 $$ 1968268 $$ 2053268 $$ 2274268 $$ 4398269 $$ 282269 $$ 863269 $$ 1950269 $$ 2741269 $$ 2881269 $$ 3465269 $$ 5178270 $$ 354270 $$ 2112270 $$ 2359270 $$ 4970271 $$ 481271 $$ 510271 $$ 908271 $$ 909271 $$ 1161272 $$ 313272 $$ 2580272 $$ 15285273 $$ 274273 $$ 1073273 $$ 1156273 $$ 2356273 $$ 7756273 $$ 13875273 $$ 36624273 $$ 37947273 $$ 41042274 $$ 1073274 $$ 1156274 $$ 2356274 $$ 7756274 $$ 13875274 $$ 26001274 $$ 37947275 $$ 323275 $$ 416275 $$ 1109275 $$ 1383275 $$ 3023275 $$ 8557275 $$ 26038275 $$ 43224276 $$ 367276 $$ 381276 $$ 873276 $$ 1315276 $$ 1775276 $$ 2034276 $$ 2106276 $$ 3791276 $$ 9428276 $$ 12309276 $$ 13429276 $$ 35717276 $$ 41187277 $$ 2187277 $$ 5895277 $$ 8363277 $$ 24681277 $$ 38922278 $$ 1529278 $$ 1659278 $$ 1661278 $$ 2340278 $$ 2837278 $$ 4061278 $$ 6640278 $$ 9182278 $$ 9601278 $$ 12428278 $$ 22784278 $$ 28491278 $$ 28553278 $$ 29011278 $$ 30684278 $$ 31513278 $$ 42684279 $$ 1781279 $$ 3255279 $$ 4950279 $$ 5151279 $$ 5462279 $$ 6525279 $$ 17047279 $$ 29846279 $$ 36653280 $$ 385280 $$ 815280 $$ 986280 $$ 1388280 $$ 2697280 $$ 37745280 $$ 41914281 $$ 836281 $$ 891281 $$ 1785281 $$ 3366281 $$ 10700281 $$ 38037281 $$ 38939281 $$ 43796282 $$ 1106282 $$ 1302282 $$ 3140282 $$ 28208283 $$ 923283 $$ 943283 $$ 1379283 $$ 1716283 $$ 2710283 $$ 2985283 $$ 4011283 $$ 4583283 $$ 5017283 $$ 6596283 $$ 7819283 $$ 8116283 $$ 13127283 $$ 17654283 $$ 19736283 $$ 28409283 $$ 43002283 $$ 44122284 $$ 338284 $$ 995284 $$ 996284 $$ 1401284 $$ 1562284 $$ 9149284 $$ 24796285 $$ 770285 $$ 1062285 $$ 1147285 $$ 1807285 $$ 2296285 $$ 8891285 $$ 9361285 $$ 12384285 $$ 13800285 $$ 27828285 $$ 36911286 $$ 545286 $$ 1301286 $$ 6377286 $$ 7092286 $$ 12242286 $$ 40671287 $$ 1086287 $$ 2112287 $$ 6601287 $$ 10766287 $$ 16480288 $$ 1600288 $$ 21227288 $$ 21228288 $$ 35212288 $$ 37708288 $$ 41115289 $$ 1252289 $$ 2977289 $$ 38092289 $$ 42930290 $$ 339290 $$ 621290 $$ 811290 $$ 1303290 $$ 1432290 $$ 1475290 $$ 4447290 $$ 7315290 $$ 8193290 $$ 36438290 $$ 37401290 $$ 40623290 $$ 40624290 $$ 41956291 $$ 1171291 $$ 1228291 $$ 10578291 $$ 14791292 $$ 2216292 $$ 44191293 $$ 842293 $$ 22693293 $$ 26661293 $$ 35342293 $$ 39893294 $$ 346294 $$ 633294 $$ 634294 $$ 635294 $$ 636294 $$ 637294 $$ 638294 $$ 639294 $$ 640294 $$ 641294 $$ 642294 $$ 643294 $$ 644294 $$ 645294 $$ 646294 $$ 647294 $$ 849294 $$ 850294 $$ 851294 $$ 1002294 $$ 1178294 $$ 1219294 $$ 1558294 $$ 1949294 $$ 2252294 $$ 3582294 $$ 7343294 $$ 10089294 $$ 13678294 $$ 37043295 $$ 776295 $$ 1038295 $$ 2182295 $$ 10905295 $$ 13573295 $$ 20672295 $$ 32990295 $$ 33449295 $$ 33508295 $$ 34234295 $$ 34286295 $$ 41496295 $$ 42137296 $$ 352296 $$ 416296 $$ 1185296 $$ 1582296 $$ 2593296 $$ 16806297 $$ 6855297 $$ 8075297 $$ 11200297 $$ 11307297 $$ 12806297 $$ 13761297 $$ 17512297 $$ 17513297 $$ 27008297 $$ 28268297 $$ 34847297 $$ 41091298 $$ 904298 $$ 1380298 $$ 2508298 $$ 2535298 $$ 2689298 $$ 3202298 $$ 3561298 $$ 4897298 $$ 6572298 $$ 10715298 $$ 14843298 $$ 16728298 $$ 21181298 $$ 25445298 $$ 39784299 $$ 664299 $$ 919299 $$ 1041299 $$ 1057299 $$ 1499299 $$ 1663299 $$ 2077299 $$ 2149299 $$ 6959299 $$ 26030299 $$ 30100299 $$ 36080299 $$ 36081299 $$ 39243299 $$ 40560300 $$ 754300 $$ 770300 $$ 1067300 $$ 1153300 $$ 1443300 $$ 1645300 $$ 1646300 $$ 1916300 $$ 1946300 $$ 2296300 $$ 2568300 $$ 3617300 $$ 6169300 $$ 38132300 $$ 42736300 $$ 42737301 $$ 506301 $$ 1049301 $$ 1182301 $$ 2686301 $$ 2721301 $$ 15343301 $$ 15344301 $$ 31816301 $$ 34891301 $$ 41179301 $$ 42530302 $$ 363302 $$ 515302 $$ 791302 $$ 1407302 $$ 1615302 $$ 1631302 $$ 2815302 $$ 5375302 $$ 12188302 $$ 12583302 $$ 16266302 $$ 26648302 $$ 31318302 $$ 33729302 $$ 37222303 $$ 2008303 $$ 5071303 $$ 31728304 $$ 314304 $$ 323304 $$ 499304 $$ 1035304 $$ 1134304 $$ 1265304 $$ 2909304 $$ 3136304 $$ 4158304 $$ 4730304 $$ 4747304 $$ 7103304 $$ 15749304 $$ 24131304 $$ 24478304 $$ 25851304 $$ 26309304 $$ 27381304 $$ 28166304 $$ 31687305 $$ 514305 $$ 532305 $$ 1285305 $$ 1286305 $$ 1705305 $$ 1883305 $$ 1911305 $$ 2216305 $$ 2569305 $$ 3807305 $$ 29551305 $$ 41847305 $$ 43539306 $$ 355306 $$ 782306 $$ 953306 $$ 1155306 $$ 1565306 $$ 1667306 $$ 3720306 $$ 4205306 $$ 4900306 $$ 4901306 $$ 4902306 $$ 5216306 $$ 5218306 $$ 5554306 $$ 14820306 $$ 14821306 $$ 25652307 $$ 1320307 $$ 19398308 $$ 321308 $$ 901308 $$ 3641308 $$ 34547308 $$ 35950308 $$ 41662308 $$ 43982309 $$ 364309 $$ 413309 $$ 1315309 $$ 1618309 $$ 1832309 $$ 2415309 $$ 8751309 $$ 13309309 $$ 13883309 $$ 21801309 $$ 23206309 $$ 24626309 $$ 25200309 $$ 34868309 $$ 36005309 $$ 36006309 $$ 36655310 $$ 524310 $$ 886310 $$ 1905310 $$ 6913310 $$ 17157311 $$ 719311 $$ 6522311 $$ 21090311 $$ 22163312 $$ 4820312 $$ 5408313 $$ 618313 $$ 1130313 $$ 1131313 $$ 1199313 $$ 1769313 $$ 2580313 $$ 4701313 $$ 4745313 $$ 6137313 $$ 12214313 $$ 12215314 $$ 323314 $$ 1003314 $$ 1035314 $$ 1134314 $$ 1265314 $$ 1418314 $$ 3052314 $$ 3390314 $$ 3427314 $$ 3530314 $$ 4679314 $$ 4747314 $$ 5161314 $$ 7103314 $$ 8976314 $$ 10734314 $$ 14143314 $$ 24478314 $$ 25441314 $$ 26309314 $$ 27381314 $$ 29589314 $$ 29590314 $$ 35036314 $$ 35037314 $$ 40458314 $$ 42077314 $$ 42938315 $$ 785315 $$ 786315 $$ 22404315 $$ 30157315 $$ 30429315 $$ 35807316 $$ 500316 $$ 1494316 $$ 26944317 $$ 1644317 $$ 17041317 $$ 29198317 $$ 36708317 $$ 41435317 $$ 43506318 $$ 534318 $$ 1143318 $$ 16102318 $$ 43183319 $$ 886319 $$ 1303319 $$ 1665319 $$ 1790319 $$ 1839319 $$ 1973319 $$ 1977319 $$ 6345319 $$ 10228319 $$ 24411319 $$ 28678319 $$ 28679319 $$ 29852320 $$ 463320 $$ 821320 $$ 5834320 $$ 10346320 $$ 23212320 $$ 23213320 $$ 23346320 $$ 27891321 $$ 901321 $$ 3641321 $$ 34547321 $$ 35950321 $$ 43982322 $$ 345322 $$ 1257322 $$ 2683322 $$ 6128322 $$ 14151322 $$ 14490322 $$ 25470322 $$ 36391323 $$ 1035323 $$ 1134323 $$ 1265323 $$ 1418323 $$ 3052323 $$ 4124323 $$ 4679323 $$ 4747323 $$ 7103323 $$ 8800323 $$ 8801323 $$ 8802323 $$ 8976323 $$ 10734323 $$ 24478323 $$ 25441323 $$ 27381323 $$ 28794323 $$ 35354323 $$ 36220324 $$ 387324 $$ 607324 $$ 989324 $$ 11984324 $$ 26506324 $$ 37443324 $$ 43804325 $$ 1070325 $$ 1109325 $$ 2177325 $$ 30747326 $$ 7124326 $$ 23107327 $$ 416327 $$ 1348327 $$ 2322327 $$ 2323327 $$ 2713327 $$ 11688327 $$ 11689327 $$ 11690327 $$ 22352327 $$ 24100327 $$ 25228327 $$ 25229327 $$ 30878327 $$ 34375327 $$ 42031327 $$ 42671328 $$ 445328 $$ 1283329 $$ 902329 $$ 908329 $$ 971329 $$ 1258329 $$ 1346329 $$ 1518329 $$ 1571329 $$ 1581329 $$ 1610329 $$ 1898329 $$ 2446329 $$ 2460329 $$ 2527329 $$ 2586329 $$ 3450329 $$ 5007329 $$ 5165329 $$ 5331329 $$ 5832329 $$ 8181329 $$ 8239329 $$ 14580329 $$ 15070329 $$ 16888329 $$ 25846329 $$ 28909329 $$ 31227329 $$ 36458330 $$ 448330 $$ 749330 $$ 930330 $$ 972330 $$ 1051330 $$ 1052330 $$ 1053330 $$ 1054330 $$ 1184330 $$ 1196330 $$ 1295330 $$ 1591330 $$ 21592330 $$ 23056330 $$ 36575330 $$ 39974330 $$ 43837331 $$ 332331 $$ 1393331 $$ 4025331 $$ 4114331 $$ 5681331 $$ 6756331 $$ 23366331 $$ 33525331 $$ 35079331 $$ 39344332 $$ 1393332 $$ 6756332 $$ 15016332 $$ 17720332 $$ 25261332 $$ 27830332 $$ 32355332 $$ 38051333 $$ 1138333 $$ 4602333 $$ 8083333 $$ 10528333 $$ 12894333 $$ 14673333 $$ 16115333 $$ 23559333 $$ 24000333 $$ 25794333 $$ 37544334 $$ 531334 $$ 738334 $$ 739334 $$ 740334 $$ 773334 $$ 1097334 $$ 1229334 $$ 5036334 $$ 8221334 $$ 20929335 $$ 2441335 $$ 3798335 $$ 3850335 $$ 6341335 $$ 7467335 $$ 12953335 $$ 14188335 $$ 17241335 $$ 21224335 $$ 25081335 $$ 35348335 $$ 42924336 $$ 759336 $$ 1363336 $$ 3422336 $$ 6247336 $$ 21665336 $$ 22442336 $$ 34397336 $$ 42497337 $$ 383337 $$ 679337 $$ 959337 $$ 1020337 $$ 1509337 $$ 14558337 $$ 14559337 $$ 17436337 $$ 20933337 $$ 32528337 $$ 37898337 $$ 41242337 $$ 42374338 $$ 13203339 $$ 400339 $$ 1751339 $$ 1760339 $$ 3822339 $$ 3995339 $$ 4331339 $$ 4493339 $$ 4868339 $$ 4913339 $$ 4992339 $$ 5287339 $$ 5288339 $$ 5304339 $$ 5305339 $$ 5706339 $$ 5885339 $$ 6528339 $$ 7315339 $$ 8193339 $$ 8862339 $$ 10048339 $$ 10049339 $$ 10050339 $$ 10051339 $$ 11820339 $$ 12549339 $$ 13412339 $$ 16567339 $$ 20835339 $$ 23565339 $$ 25407339 $$ 25477339 $$ 27104339 $$ 42226340 $$ 11868340 $$ 31455341 $$ 443341 $$ 1082341 $$ 1413341 $$ 1611341 $$ 1665341 $$ 1790341 $$ 1977341 $$ 2283341 $$ 2605341 $$ 3716341 $$ 3935341 $$ 5597341 $$ 5946341 $$ 6474341 $$ 7322341 $$ 33050342 $$ 1482342 $$ 34073342 $$ 36375342 $$ 41326343 $$ 617343 $$ 1023344 $$ 1603344 $$ 1840344 $$ 2962344 $$ 4373344 $$ 5240344 $$ 7950344 $$ 23184344 $$ 34640344 $$ 39821344 $$ 42534344 $$ 42535345 $$ 840345 $$ 1687345 $$ 3467345 $$ 42723346 $$ 633346 $$ 634346 $$ 635346 $$ 636346 $$ 637346 $$ 638346 $$ 639346 $$ 640346 $$ 641346 $$ 642346 $$ 643346 $$ 644346 $$ 645346 $$ 646346 $$ 647346 $$ 849346 $$ 850346 $$ 851346 $$ 1002346 $$ 1178346 $$ 1219346 $$ 1558346 $$ 1949346 $$ 2252346 $$ 3528346 $$ 3582346 $$ 4148346 $$ 4150346 $$ 7343346 $$ 8106346 $$ 10089346 $$ 12539346 $$ 13678346 $$ 16742346 $$ 28464346 $$ 29879346 $$ 29880346 $$ 30659346 $$ 36592346 $$ 37005346 $$ 37043347 $$ 365347 $$ 535347 $$ 1103347 $$ 1297347 $$ 13605347 $$ 39106347 $$ 40081347 $$ 42965347 $$ 44030348 $$ 1117348 $$ 1809348 $$ 5630348 $$ 31573349 $$ 541349 $$ 677349 $$ 1122349 $$ 1294349 $$ 1351349 $$ 2623349 $$ 3021349 $$ 4676349 $$ 6627349 $$ 7983349 $$ 12177349 $$ 14816349 $$ 15610349 $$ 25144350 $$ 1033350 $$ 1189350 $$ 16986350 $$ 36949351 $$ 428351 $$ 3695351 $$ 33873351 $$ 40464352 $$ 1582353 $$ 1254353 $$ 1358353 $$ 8682353 $$ 20041353 $$ 20062354 $$ 3288354 $$ 31984355 $$ 1155355 $$ 1565355 $$ 3160355 $$ 33257355 $$ 39505355 $$ 42287356 $$ 370356 $$ 1346356 $$ 1610356 $$ 1795356 $$ 2654356 $$ 3773357 $$ 1175357 $$ 1322357 $$ 1446357 $$ 1736357 $$ 2705357 $$ 3043357 $$ 6524357 $$ 10953357 $$ 23143357 $$ 27725357 $$ 33064358 $$ 4735358 $$ 4927358 $$ 5215358 $$ 9810358 $$ 10674358 $$ 11552358 $$ 11661358 $$ 16714358 $$ 21346359 $$ 393359 $$ 504359 $$ 517359 $$ 548359 $$ 729359 $$ 1036359 $$ 2559359 $$ 6982359 $$ 12280359 $$ 30919359 $$ 33063359 $$ 36216359 $$ 37327359 $$ 39465359 $$ 42104360 $$ 4435360 $$ 9760360 $$ 10480360 $$ 16355360 $$ 34188360 $$ 35159360 $$ 36990361 $$ 648361 $$ 1105361 $$ 7536361 $$ 28263362 $$ 888362 $$ 993362 $$ 1566362 $$ 1567362 $$ 1586362 $$ 1736362 $$ 3077362 $$ 7377362 $$ 7378362 $$ 12411362 $$ 15096362 $$ 25904363 $$ 515363 $$ 791363 $$ 1407363 $$ 1615363 $$ 1631363 $$ 2815363 $$ 2879363 $$ 5375363 $$ 12583363 $$ 16266363 $$ 26648363 $$ 28996363 $$ 33729364 $$ 465364 $$ 1023364 $$ 5255364 $$ 8440364 $$ 9097364 $$ 15414364 $$ 22832364 $$ 22928364 $$ 22929364 $$ 23206364 $$ 34868364 $$ 42009365 $$ 535365 $$ 557365 $$ 1103365 $$ 1297365 $$ 8162365 $$ 9410365 $$ 39106366 $$ 2930366 $$ 10747366 $$ 14141366 $$ 24025366 $$ 25592366 $$ 28907366 $$ 30018366 $$ 31216367 $$ 381367 $$ 1315367 $$ 1775367 $$ 2034367 $$ 9428367 $$ 12309367 $$ 13429367 $$ 13547367 $$ 19983367 $$ 19984367 $$ 29487367 $$ 41187367 $$ 42855368 $$ 4292368 $$ 31326370 $$ 392370 $$ 1316370 $$ 1479370 $$ 3586370 $$ 5131370 $$ 12244370 $$ 26075370 $$ 43518371 $$ 540371 $$ 1361371 $$ 1524371 $$ 12279371 $$ 22496371 $$ 24194371 $$ 40076372 $$ 381372 $$ 549372 $$ 1136372 $$ 1463372 $$ 1603372 $$ 1688372 $$ 2151372 $$ 2228372 $$ 3118372 $$ 3585372 $$ 6144372 $$ 27000372 $$ 30146372 $$ 33242372 $$ 37846373 $$ 1424373 $$ 4005373 $$ 26377373 $$ 33903373 $$ 39792374 $$ 764374 $$ 1422374 $$ 1455374 $$ 2103374 $$ 3665374 $$ 3847374 $$ 7841374 $$ 9731374 $$ 11502374 $$ 23767374 $$ 26559374 $$ 27040374 $$ 29497374 $$ 36107374 $$ 43455375 $$ 418375 $$ 482375 $$ 556375 $$ 767375 $$ 2159375 $$ 2946375 $$ 7920375 $$ 13538375 $$ 37688376 $$ 1129376 $$ 33011377 $$ 737377 $$ 746377 $$ 1366377 $$ 2703377 $$ 6285377 $$ 10298377 $$ 13285377 $$ 21034377 $$ 24597377 $$ 32578377 $$ 35067378 $$ 11905378 $$ 15530379 $$ 16766379 $$ 19855379 $$ 21855379 $$ 26003379 $$ 38178379 $$ 40157379 $$ 42313379 $$ 42848380 $$ 562380 $$ 3655380 $$ 4122380 $$ 4608380 $$ 8781380 $$ 11515380 $$ 14554381 $$ 1673381 $$ 1688381 $$ 2034381 $$ 2228381 $$ 2532381 $$ 12309381 $$ 30410381 $$ 40183382 $$ 2000382 $$ 5629382 $$ 30871383 $$ 679383 $$ 959383 $$ 1020383 $$ 1509383 $$ 20933383 $$ 32528383 $$ 37898383 $$ 41242384 $$ 2592384 $$ 2695384 $$ 5634384 $$ 7296384 $$ 8442384 $$ 15303384 $$ 15575384 $$ 19525384 $$ 27696384 $$ 30061384 $$ 30520384 $$ 30943384 $$ 31981384 $$ 32812384 $$ 33142384 $$ 34113384 $$ 42422385 $$ 399385 $$ 986385 $$ 2596385 $$ 3413385 $$ 5497385 $$ 25509385 $$ 31982385 $$ 39234386 $$ 1349386 $$ 1358386 $$ 2000386 $$ 2061386 $$ 2078386 $$ 2272386 $$ 2402386 $$ 2889386 $$ 3022386 $$ 9879386 $$ 14763386 $$ 14764386 $$ 26334386 $$ 28650386 $$ 30468386 $$ 41631387 $$ 607387 $$ 989387 $$ 1143387 $$ 11984387 $$ 26085387 $$ 26506387 $$ 35842387 $$ 37443388 $$ 965388 $$ 1079388 $$ 2298389 $$ 1159389 $$ 1323389 $$ 1346389 $$ 1462389 $$ 1610389 $$ 11133389 $$ 32248389 $$ 35844390 $$ 790390 $$ 1588390 $$ 24572390 $$ 26004390 $$ 31791391 $$ 770391 $$ 9530391 $$ 42010391 $$ 43220392 $$ 675392 $$ 676392 $$ 1238392 $$ 1458392 $$ 1479392 $$ 2291392 $$ 4518392 $$ 12244392 $$ 14166392 $$ 23049392 $$ 33561392 $$ 37194392 $$ 37883393 $$ 517393 $$ 1036393 $$ 1478393 $$ 3201393 $$ 4304393 $$ 25935394 $$ 987394 $$ 1481394 $$ 2626394 $$ 2978394 $$ 3332394 $$ 5156394 $$ 13949394 $$ 16835394 $$ 22366394 $$ 31627394 $$ 40722395 $$ 1254395 $$ 31260396 $$ 465396 $$ 2401396 $$ 2617396 $$ 3087396 $$ 3356396 $$ 4047396 $$ 10045396 $$ 19574396 $$ 30222396 $$ 32469396 $$ 36826397 $$ 440397 $$ 479397 $$ 723397 $$ 1386397 $$ 1481397 $$ 2216397 $$ 33617397 $$ 34477397 $$ 36335397 $$ 37968398 $$ 2948398 $$ 5064398 $$ 11912398 $$ 28140399 $$ 986399 $$ 17962399 $$ 34596399 $$ 35318400 $$ 1337400 $$ 5913400 $$ 8068400 $$ 13412400 $$ 42226401 $$ 652401 $$ 1107401 $$ 1280401 $$ 1321401 $$ 1417401 $$ 1456401 $$ 1881401 $$ 6470401 $$ 6471401 $$ 6472401 $$ 25585401 $$ 28095401 $$ 42873401 $$ 43791401 $$ 43792402 $$ 509402 $$ 597402 $$ 1047402 $$ 1195402 $$ 5966402 $$ 8397402 $$ 13569402 $$ 22382402 $$ 27704402 $$ 40496403 $$ 1741403 $$ 2519403 $$ 3165403 $$ 5838403 $$ 38462404 $$ 607404 $$ 683404 $$ 1125404 $$ 5252404 $$ 6668404 $$ 10306404 $$ 13218404 $$ 22495405 $$ 525405 $$ 599405 $$ 813405 $$ 2616405 $$ 4570405 $$ 6189405 $$ 7085405 $$ 12559405 $$ 12799405 $$ 14236405 $$ 17999405 $$ 20138406 $$ 732406 $$ 2264406 $$ 5329406 $$ 12288406 $$ 36381407 $$ 1362407 $$ 2476409 $$ 17199409 $$ 32843409 $$ 38502409 $$ 38503409 $$ 40859409 $$ 42580410 $$ 1398410 $$ 2773411 $$ 459411 $$ 462411 $$ 471411 $$ 2532411 $$ 41934411 $$ 44127412 $$ 816412 $$ 4469412 $$ 9894412 $$ 14914412 $$ 26160412 $$ 27560412 $$ 32051412 $$ 33310412 $$ 42507413 $$ 950413 $$ 951413 $$ 1193413 $$ 1632413 $$ 1832413 $$ 2756413 $$ 3346413 $$ 4089413 $$ 4254413 $$ 7993413 $$ 8751413 $$ 27880413 $$ 27881413 $$ 30880413 $$ 31266413 $$ 36208413 $$ 41877414 $$ 508414 $$ 1169414 $$ 1296414 $$ 2846414 $$ 3858414 $$ 5039414 $$ 13128414 $$ 21895414 $$ 38724415 $$ 835415 $$ 934415 $$ 993415 $$ 4051415 $$ 27823415 $$ 34843415 $$ 35837415 $$ 37448415 $$ 38211416 $$ 846416 $$ 997416 $$ 998416 $$ 1454416 $$ 1786416 $$ 1937416 $$ 1955416 $$ 2101416 $$ 2831416 $$ 3269416 $$ 3830416 $$ 3876416 $$ 4167416 $$ 4510416 $$ 5598416 $$ 7232416 $$ 15652416 $$ 40713416 $$ 42587416 $$ 42671417 $$ 654417 $$ 854417 $$ 2223417 $$ 2241417 $$ 4148417 $$ 6764417 $$ 9524417 $$ 12124417 $$ 17712417 $$ 42465418 $$ 538418 $$ 2159418 $$ 6499418 $$ 7899418 $$ 8482418 $$ 9638418 $$ 10722418 $$ 12153418 $$ 15980418 $$ 17615418 $$ 17716418 $$ 25431418 $$ 25460418 $$ 26636418 $$ 39348419 $$ 683419 $$ 878419 $$ 1029419 $$ 1030419 $$ 1125419 $$ 7437419 $$ 14382419 $$ 16444419 $$ 17006419 $$ 28362419 $$ 36331419 $$ 36995419 $$ 36996420 $$ 422420 $$ 1553420 $$ 11108420 $$ 30634420 $$ 43700420 $$ 43701421 $$ 7927421 $$ 30332422 $$ 1198422 $$ 5155422 $$ 34486423 $$ 828423 $$ 1200423 $$ 14691423 $$ 34888424 $$ 1958424 $$ 3234424 $$ 14825424 $$ 22276424 $$ 24452425 $$ 8812426 $$ 1168426 $$ 1392426 $$ 2961426 $$ 3239426 $$ 3546426 $$ 20240426 $$ 42522427 $$ 1475427 $$ 2428427 $$ 2639427 $$ 2817427 $$ 3991427 $$ 8240427 $$ 9627427 $$ 13255427 $$ 16174427 $$ 20859427 $$ 27544427 $$ 34794427 $$ 36539427 $$ 36715427 $$ 42175427 $$ 44055428 $$ 3695428 $$ 5110428 $$ 13134428 $$ 34790428 $$ 34791428 $$ 38194428 $$ 42026429 $$ 1276429 $$ 1279429 $$ 1334429 $$ 15702429 $$ 33718429 $$ 38470430 $$ 3910430 $$ 16370431 $$ 670431 $$ 853431 $$ 1004431 $$ 1514431 $$ 1953431 $$ 2010431 $$ 2333431 $$ 4365431 $$ 33057432 $$ 883432 $$ 884432 $$ 885432 $$ 985432 $$ 1024432 $$ 1025432 $$ 1225432 $$ 1530432 $$ 4010432 $$ 8703432 $$ 20471432 $$ 23120432 $$ 31275432 $$ 42112433 $$ 713433 $$ 714433 $$ 1549433 $$ 38750434 $$ 1421434 $$ 15909434 $$ 16991434 $$ 42188435 $$ 1615435 $$ 18072435 $$ 30404436 $$ 987436 $$ 1629436 $$ 1653436 $$ 1743436 $$ 1744436 $$ 1771436 $$ 2294436 $$ 4752436 $$ 12291437 $$ 592437 $$ 754437 $$ 932437 $$ 1811437 $$ 6313437 $$ 9483437 $$ 16839437 $$ 21363437 $$ 22289437 $$ 41498439 $$ 1465439 $$ 7178439 $$ 8336439 $$ 8847439 $$ 10527439 $$ 12698439 $$ 12699439 $$ 13049439 $$ 13176439 $$ 17922439 $$ 18346439 $$ 20413439 $$ 24467439 $$ 31678440 $$ 1066440 $$ 1320440 $$ 1725440 $$ 2216440 $$ 4292440 $$ 37968441 $$ 754441 $$ 3605441 $$ 22288441 $$ 25587442 $$ 1163443 $$ 1082443 $$ 1413443 $$ 2283443 $$ 3716443 $$ 3935443 $$ 5597443 $$ 20051444 $$ 529444 $$ 1614444 $$ 1700444 $$ 1873444 $$ 8704444 $$ 16271444 $$ 23289444 $$ 23290445 $$ 658445 $$ 13869445 $$ 37341445 $$ 44168446 $$ 1719446 $$ 4705446 $$ 10572446 $$ 19956446 $$ 35077447 $$ 19945447 $$ 25841447 $$ 32414448 $$ 543448 $$ 688448 $$ 749448 $$ 930448 $$ 972448 $$ 1021448 $$ 1051448 $$ 1052448 $$ 1196448 $$ 1330448 $$ 1441448 $$ 36729449 $$ 741449 $$ 43094450 $$ 1914450 $$ 2189450 $$ 3228450 $$ 3517450 $$ 4771450 $$ 29535450 $$ 43165450 $$ 43390450 $$ 44172451 $$ 1037451 $$ 1135451 $$ 1525452 $$ 476452 $$ 531452 $$ 738452 $$ 739452 $$ 740452 $$ 914452 $$ 915452 $$ 1229452 $$ 1230452 $$ 2343452 $$ 2480452 $$ 3746452 $$ 7364452 $$ 13986452 $$ 13987452 $$ 25226452 $$ 25422452 $$ 31616453 $$ 591453 $$ 981453 $$ 1167453 $$ 2381453 $$ 3954453 $$ 4250453 $$ 5636453 $$ 29654453 $$ 29655453 $$ 30863453 $$ 42956454 $$ 1240454 $$ 1747454 $$ 2636454 $$ 3046454 $$ 3733454 $$ 10002454 $$ 12364454 $$ 23381454 $$ 23382454 $$ 28763454 $$ 35630454 $$ 35631455 $$ 539455 $$ 551455 $$ 919455 $$ 1062455 $$ 1405455 $$ 1495455 $$ 2696455 $$ 3395455 $$ 6973455 $$ 15547455 $$ 15548455 $$ 19423455 $$ 21569455 $$ 24009455 $$ 32001456 $$ 1723456 $$ 1746456 $$ 4303456 $$ 6143456 $$ 7277456 $$ 11361456 $$ 11372456 $$ 16403456 $$ 16996456 $$ 17444456 $$ 20926456 $$ 20927456 $$ 42172456 $$ 43758456 $$ 43759457 $$ 796457 $$ 3477457 $$ 4642457 $$ 8861457 $$ 22225458 $$ 798458 $$ 966458 $$ 1090458 $$ 1769458 $$ 1979458 $$ 2063458 $$ 4248458 $$ 4688458 $$ 10067458 $$ 11827458 $$ 27878458 $$ 43202459 $$ 493459 $$ 2532459 $$ 30329459 $$ 44127460 $$ 505460 $$ 3226460 $$ 4404460 $$ 5878460 $$ 8699460 $$ 30440461 $$ 1269461 $$ 1602461 $$ 1796461 $$ 4136461 $$ 4384461 $$ 5027461 $$ 13229461 $$ 15094461 $$ 20750461 $$ 21545461 $$ 21778461 $$ 23443461 $$ 32164461 $$ 43193462 $$ 1447462 $$ 1605462 $$ 2599462 $$ 5267462 $$ 28617462 $$ 35791463 $$ 821463 $$ 27891464 $$ 555464 $$ 614464 $$ 615464 $$ 616464 $$ 1414464 $$ 2033464 $$ 2135464 $$ 4506464 $$ 26958465 $$ 676465 $$ 979465 $$ 1023465 $$ 1836465 $$ 2617465 $$ 4770465 $$ 5255465 $$ 9097465 $$ 9891465 $$ 29084465 $$ 31598466 $$ 837466 $$ 838466 $$ 992466 $$ 1263466 $$ 1292466 $$ 35827466 $$ 43450467 $$ 468467 $$ 497467 $$ 498467 $$ 5939467 $$ 21673467 $$ 25605467 $$ 25606467 $$ 29316468 $$ 497468 $$ 498468 $$ 25605468 $$ 25606468 $$ 28229468 $$ 28938468 $$ 39227468 $$ 39532469 $$ 684469 $$ 685469 $$ 6539469 $$ 23608469 $$ 32889470 $$ 10071470 $$ 13870470 $$ 14086470 $$ 23062470 $$ 27866470 $$ 27867470 $$ 27868471 $$ 1300471 $$ 1902471 $$ 2287471 $$ 3013471 $$ 5023471 $$ 9224471 $$ 30089472 $$ 1818472 $$ 1858472 $$ 2258472 $$ 4658472 $$ 5428472 $$ 20024472 $$ 30333473 $$ 545473 $$ 14239473 $$ 21787474 $$ 774474 $$ 775475 $$ 913475 $$ 923475 $$ 1180475 $$ 7567475 $$ 29682475 $$ 42993475 $$ 43585475 $$ 43586476 $$ 531476 $$ 738476 $$ 739476 $$ 740476 $$ 914476 $$ 915476 $$ 1229476 $$ 1230476 $$ 2343476 $$ 2480476 $$ 3746476 $$ 7364476 $$ 13986476 $$ 13987476 $$ 25226476 $$ 28018476 $$ 28019476 $$ 28020476 $$ 30890476 $$ 31616477 $$ 915477 $$ 1378477 $$ 1997477 $$ 3242477 $$ 5110477 $$ 29456477 $$ 38871477 $$ 42344478 $$ 40993479 $$ 723479 $$ 1386479 $$ 6966479 $$ 32427479 $$ 44197480 $$ 1238480 $$ 1341480 $$ 2836480 $$ 3312481 $$ 510481 $$ 909481 $$ 18991481 $$ 28647481 $$ 40879482 $$ 556482 $$ 767482 $$ 1721482 $$ 7735482 $$ 10313482 $$ 26375482 $$ 37688483 $$ 10347483 $$ 40959484 $$ 813484 $$ 1006484 $$ 1289484 $$ 7001484 $$ 11851484 $$ 40937485 $$ 1181485 $$ 1708485 $$ 2374485 $$ 10324485 $$ 12120485 $$ 13749485 $$ 14226485 $$ 18740485 $$ 28258485 $$ 32493485 $$ 40036486 $$ 653486 $$ 797486 $$ 1078486 $$ 1393486 $$ 3900486 $$ 4020486 $$ 10478486 $$ 38832487 $$ 1311487 $$ 3902487 $$ 4247487 $$ 14936487 $$ 42471488 $$ 586488 $$ 801488 $$ 43851488 $$ 43852488 $$ 43853489 $$ 632489 $$ 848489 $$ 4318489 $$ 4550489 $$ 20886489 $$ 25282489 $$ 28262490 $$ 2282490 $$ 19853491 $$ 1034491 $$ 1322491 $$ 1519491 $$ 1706491 $$ 2371491 $$ 3576491 $$ 4526491 $$ 5327491 $$ 5421491 $$ 6303491 $$ 9559492 $$ 4636492 $$ 20090492 $$ 42942493 $$ 988493 $$ 4879493 $$ 19819493 $$ 37865494 $$ 934494 $$ 935494 $$ 1499494 $$ 1791494 $$ 3459494 $$ 3748494 $$ 3775494 $$ 4452494 $$ 5377494 $$ 22399494 $$ 34352495 $$ 1824495 $$ 6138496 $$ 861496 $$ 1397496 $$ 3343496 $$ 4816496 $$ 4863496 $$ 27509496 $$ 28750497 $$ 498497 $$ 690497 $$ 5939497 $$ 21673497 $$ 25605497 $$ 25606497 $$ 28229497 $$ 28938497 $$ 29029497 $$ 30035497 $$ 31050498 $$ 28229498 $$ 28938498 $$ 39227498 $$ 39532499 $$ 2749499 $$ 2909499 $$ 3136499 $$ 3162499 $$ 3341499 $$ 3780499 $$ 4158499 $$ 6923499 $$ 9157499 $$ 24131500 $$ 2209500 $$ 26944501 $$ 2147501 $$ 5434501 $$ 5501501 $$ 8435501 $$ 8625501 $$ 11002501 $$ 15418501 $$ 15936501 $$ 28346501 $$ 31700501 $$ 39798501 $$ 41299501 $$ 41300501 $$ 42343502 $$ 10071502 $$ 42623503 $$ 1092503 $$ 3375503 $$ 7764504 $$ 548504 $$ 729504 $$ 11086504 $$ 17183504 $$ 27750504 $$ 27751504 $$ 30369504 $$ 33063504 $$ 35867504 $$ 35868504 $$ 36216504 $$ 37327504 $$ 39465504 $$ 42104505 $$ 6083505 $$ 30440506 $$ 660506 $$ 1012506 $$ 1049506 $$ 3356506 $$ 5237506 $$ 5633506 $$ 5746506 $$ 6017506 $$ 7240506 $$ 8365506 $$ 21312506 $$ 21313506 $$ 29658506 $$ 31584506 $$ 35721506 $$ 37541506 $$ 38429506 $$ 42530507 $$ 1376507 $$ 7584507 $$ 12856507 $$ 31997507 $$ 34285508 $$ 597508 $$ 1169508 $$ 1296508 $$ 2846508 $$ 3858508 $$ 5039508 $$ 6749508 $$ 13128508 $$ 19965509 $$ 597509 $$ 1195509 $$ 4867509 $$ 8049510 $$ 909510 $$ 18991510 $$ 40879511 $$ 558511 $$ 592511 $$ 1734511 $$ 16633511 $$ 26482512 $$ 1252512 $$ 1385512 $$ 8686512 $$ 17446512 $$ 26484512 $$ 34989513 $$ 1070513 $$ 1355513 $$ 6698513 $$ 16316513 $$ 38599513 $$ 42253514 $$ 1005514 $$ 1911514 $$ 2569514 $$ 2803514 $$ 3047514 $$ 3905514 $$ 10479514 $$ 13924514 $$ 13925514 $$ 37943514 $$ 39319515 $$ 791515 $$ 1208515 $$ 1407515 $$ 1615515 $$ 1631515 $$ 1952515 $$ 2815515 $$ 12583515 $$ 16266515 $$ 20103515 $$ 26647515 $$ 33729515 $$ 35699515 $$ 35700515 $$ 37222516 $$ 25095516 $$ 28835516 $$ 37749517 $$ 1036517 $$ 2232517 $$ 4304517 $$ 10968518 $$ 772518 $$ 4334518 $$ 5765518 $$ 6516518 $$ 22505518 $$ 27019519 $$ 29320520 $$ 580520 $$ 2005520 $$ 3522520 $$ 11666520 $$ 41933521 $$ 522521 $$ 810521 $$ 963521 $$ 975521 $$ 1089521 $$ 1165521 $$ 2246521 $$ 6974521 $$ 17448521 $$ 22328521 $$ 22977522 $$ 810522 $$ 963522 $$ 975522 $$ 1089522 $$ 1165522 $$ 2246522 $$ 13321522 $$ 17687522 $$ 17688522 $$ 22328522 $$ 28283522 $$ 29314522 $$ 43404524 $$ 886524 $$ 1905524 $$ 6913524 $$ 17157524 $$ 17236524 $$ 17237525 $$ 599525 $$ 600525 $$ 1811526 $$ 1433526 $$ 3981527 $$ 828527 $$ 3608527 $$ 4978527 $$ 5776527 $$ 7040527 $$ 11897527 $$ 43410528 $$ 5951528 $$ 19952528 $$ 25542528 $$ 40591529 $$ 1172529 $$ 1216529 $$ 1630529 $$ 8022529 $$ 25281529 $$ 42975530 $$ 3885530 $$ 4110530 $$ 4363530 $$ 5518530 $$ 5568530 $$ 7212530 $$ 9860530 $$ 11295530 $$ 11423530 $$ 13788530 $$ 14254530 $$ 14703530 $$ 16432530 $$ 17606530 $$ 17673530 $$ 21254530 $$ 22323530 $$ 25306530 $$ 27759530 $$ 28005530 $$ 28006530 $$ 28041530 $$ 33522530 $$ 35145530 $$ 36963531 $$ 738531 $$ 739531 $$ 740531 $$ 914531 $$ 1229531 $$ 1230531 $$ 2343531 $$ 2480531 $$ 3746531 $$ 7364531 $$ 8221531 $$ 8702531 $$ 15118531 $$ 22480531 $$ 22481531 $$ 24888531 $$ 28014531 $$ 28015531 $$ 28016531 $$ 30890531 $$ 31616531 $$ 40531532 $$ 927532 $$ 4237532 $$ 8507533 $$ 14985533 $$ 17373534 $$ 2053534 $$ 3609535 $$ 1103535 $$ 1297535 $$ 8739535 $$ 42391536 $$ 1769536 $$ 31316537 $$ 935537 $$ 1791537 $$ 12388537 $$ 25787538 $$ 2159538 $$ 7899538 $$ 8482538 $$ 10722538 $$ 15980538 $$ 25431538 $$ 25460538 $$ 26636539 $$ 1711539 $$ 6973540 $$ 904540 $$ 1577540 $$ 1778540 $$ 2508540 $$ 4779540 $$ 5757540 $$ 11216540 $$ 14607541 $$ 677541 $$ 1122541 $$ 2623541 $$ 12177541 $$ 13628542 $$ 681542 $$ 875542 $$ 1438542 $$ 1560542 $$ 40663543 $$ 688543 $$ 1021543 $$ 3332543 $$ 25901544 $$ 563544 $$ 691544 $$ 1223544 $$ 2214544 $$ 21142545 $$ 1301545 $$ 12242545 $$ 12770545 $$ 21787546 $$ 733546 $$ 1114546 $$ 10126546 $$ 17618546 $$ 20372546 $$ 27555547 $$ 1553547 $$ 14144547 $$ 21737547 $$ 25362547 $$ 32437547 $$ 33877548 $$ 729548 $$ 2022548 $$ 11086548 $$ 25801548 $$ 30369548 $$ 33063548 $$ 36216548 $$ 37327548 $$ 39465548 $$ 42104549 $$ 1136549 $$ 4288549 $$ 9951549 $$ 25530549 $$ 36509550 $$ 742550 $$ 28309550 $$ 32885550 $$ 33758550 $$ 37540550 $$ 38428550 $$ 39691550 $$ 43089551 $$ 1277551 $$ 5889551 $$ 12339551 $$ 16831551 $$ 21569551 $$ 24009551 $$ 31998552 $$ 2216552 $$ 26046553 $$ 723553 $$ 24331553 $$ 36026554 $$ 556554 $$ 5871554 $$ 40104555 $$ 614555 $$ 615555 $$ 1123555 $$ 1149555 $$ 1404555 $$ 1414555 $$ 1442555 $$ 2434555 $$ 3415555 $$ 7213555 $$ 11305555 $$ 35828556 $$ 1441556 $$ 4476556 $$ 5871556 $$ 8112556 $$ 11127556 $$ 22507556 $$ 28141556 $$ 29979556 $$ 31599556 $$ 34823556 $$ 36130556 $$ 40573557 $$ 1063557 $$ 1075557 $$ 1150557 $$ 1303557 $$ 1748557 $$ 2091557 $$ 2164557 $$ 2170557 $$ 3917557 $$ 8162557 $$ 9410557 $$ 24173557 $$ 25625557 $$ 29470557 $$ 32869557 $$ 35773558 $$ 592558 $$ 942558 $$ 1734558 $$ 16633558 $$ 26482559 $$ 2087559 $$ 5456560 $$ 3624560 $$ 3941560 $$ 4342560 $$ 4483560 $$ 5038560 $$ 8473560 $$ 10249560 $$ 23046560 $$ 23047560 $$ 24932560 $$ 33393560 $$ 38215560 $$ 41998560 $$ 42157561 $$ 3471561 $$ 6402561 $$ 12826561 $$ 19040561 $$ 29877562 $$ 990562 $$ 1299562 $$ 2037562 $$ 4828562 $$ 11515562 $$ 32364563 $$ 691563 $$ 1223563 $$ 2214563 $$ 21142564 $$ 3581564 $$ 6357564 $$ 13740564 $$ 21829564 $$ 35220565 $$ 566565 $$ 795565 $$ 964566 $$ 795566 $$ 964566 $$ 1416566 $$ 2090566 $$ 4785566 $$ 39095568 $$ 1421568 $$ 1426568 $$ 1740568 $$ 1934568 $$ 5566568 $$ 12190569 $$ 658569 $$ 1238569 $$ 1317569 $$ 1368569 $$ 1944569 $$ 4094570 $$ 2348570 $$ 40125571 $$ 602571 $$ 6959571 $$ 21869572 $$ 604572 $$ 870572 $$ 1017572 $$ 1346572 $$ 1384572 $$ 1610572 $$ 1901572 $$ 8523572 $$ 33731573 $$ 574573 $$ 575573 $$ 802573 $$ 1894573 $$ 2161573 $$ 11176573 $$ 16906573 $$ 24605573 $$ 24606573 $$ 29548573 $$ 33935574 $$ 575574 $$ 802574 $$ 1172574 $$ 1216574 $$ 1252574 $$ 1447574 $$ 1467574 $$ 1471574 $$ 1485574 $$ 1488574 $$ 1503574 $$ 1504574 $$ 1505574 $$ 1506574 $$ 1594574 $$ 1755574 $$ 1801574 $$ 1823574 $$ 1894574 $$ 2161574 $$ 11176574 $$ 15546574 $$ 16906574 $$ 24605574 $$ 24606574 $$ 29548574 $$ 33935574 $$ 34845574 $$ 34870575 $$ 802575 $$ 1894575 $$ 2161575 $$ 11176575 $$ 16906575 $$ 24605575 $$ 24606575 $$ 29548576 $$ 1239577 $$ 578577 $$ 579577 $$ 2042577 $$ 2533577 $$ 11838577 $$ 28444578 $$ 579578 $$ 2042578 $$ 2533578 $$ 11838578 $$ 28444579 $$ 1383579 $$ 2533579 $$ 9183579 $$ 28444580 $$ 1783580 $$ 13675581 $$ 1682581 $$ 2247581 $$ 6214581 $$ 43195582 $$ 10840583 $$ 1271583 $$ 1940583 $$ 2756583 $$ 3138583 $$ 8007583 $$ 26103583 $$ 30572584 $$ 595584 $$ 1664584 $$ 1875584 $$ 3106584 $$ 5192584 $$ 6505584 $$ 35838584 $$ 42023584 $$ 42962585 $$ 1173585 $$ 3185585 $$ 3529585 $$ 5207585 $$ 10369585 $$ 22932586 $$ 801587 $$ 25090588 $$ 2851588 $$ 7472588 $$ 13228588 $$ 30819589 $$ 1574589 $$ 2778589 $$ 7503589 $$ 22602589 $$ 22603589 $$ 25911590 $$ 1507591 $$ 981591 $$ 1167591 $$ 3554591 $$ 11863591 $$ 13327591 $$ 31427591 $$ 41902592 $$ 1335592 $$ 1734592 $$ 34884592 $$ 38268593 $$ 817593 $$ 824593 $$ 828593 $$ 2319593 $$ 2429593 $$ 6904593 $$ 10372593 $$ 21994593 $$ 21995593 $$ 21996593 $$ 21997593 $$ 21998593 $$ 32472594 $$ 2537594 $$ 3157594 $$ 3242594 $$ 20426594 $$ 42072594 $$ 42939594 $$ 43844595 $$ 1875595 $$ 3106595 $$ 29934595 $$ 42023595 $$ 42962596 $$ 605596 $$ 831596 $$ 1238596 $$ 2082596 $$ 2999596 $$ 3577596 $$ 5781596 $$ 28845597 $$ 1195597 $$ 4867598 $$ 825598 $$ 826598 $$ 827598 $$ 983598 $$ 1095598 $$ 1533598 $$ 1707598 $$ 9486598 $$ 13974599 $$ 600599 $$ 1811600 $$ 3671600 $$ 3850600 $$ 43865601 $$ 1019601 $$ 1318601 $$ 4117601 $$ 4980601 $$ 39742602 $$ 6959602 $$ 21869603 $$ 626603 $$ 627603 $$ 839603 $$ 1415603 $$ 1526603 $$ 1834603 $$ 2820604 $$ 869604 $$ 870604 $$ 1017604 $$ 1346604 $$ 1610604 $$ 2124604 $$ 5154604 $$ 11328604 $$ 34009605 $$ 831605 $$ 1080605 $$ 1238605 $$ 1290605 $$ 1472605 $$ 4320605 $$ 5066605 $$ 25662606 $$ 612606 $$ 613607 $$ 796607 $$ 989607 $$ 6221607 $$ 11984607 $$ 12764607 $$ 26085608 $$ 1389608 $$ 5156608 $$ 13286608 $$ 15642609 $$ 830609 $$ 2524609 $$ 12900609 $$ 32077610 $$ 30397610 $$ 41488611 $$ 1174612 $$ 613612 $$ 16719613 $$ 16719614 $$ 615614 $$ 616614 $$ 1414614 $$ 2033614 $$ 2135614 $$ 4506614 $$ 26958615 $$ 616615 $$ 1414615 $$ 2033615 $$ 2135615 $$ 4506615 $$ 26958616 $$ 1414616 $$ 2033616 $$ 2135616 $$ 2614616 $$ 4506617 $$ 1453617 $$ 6217617 $$ 13284618 $$ 766618 $$ 2231618 $$ 5643618 $$ 20937618 $$ 40047619 $$ 620619 $$ 1892619 $$ 2314619 $$ 3688619 $$ 4145619 $$ 6219619 $$ 9348619 $$ 10202619 $$ 24228619 $$ 26087619 $$ 31502619 $$ 33392620 $$ 1919620 $$ 2314620 $$ 8943620 $$ 20571620 $$ 26087620 $$ 29107621 $$ 1475621 $$ 2381621 $$ 2622621 $$ 30040621 $$ 37444622 $$ 1217622 $$ 1491622 $$ 27789622 $$ 30097622 $$ 42544623 $$ 835623 $$ 836623 $$ 993623 $$ 1218623 $$ 1303623 $$ 1833623 $$ 16481624 $$ 10780624 $$ 14112624 $$ 31044625 $$ 3315625 $$ 3868626 $$ 627626 $$ 839626 $$ 1415626 $$ 1834626 $$ 3717626 $$ 13681626 $$ 26959626 $$ 35847627 $$ 839627 $$ 1415627 $$ 1834627 $$ 3717627 $$ 13681627 $$ 20606627 $$ 35847628 $$ 6261628 $$ 7800628 $$ 7967628 $$ 19311628 $$ 25603628 $$ 27786628 $$ 33047628 $$ 33048629 $$ 841629 $$ 1228630 $$ 7227630 $$ 12739630 $$ 22986631 $$ 5496631 $$ 6118631 $$ 8744631 $$ 13746631 $$ 27841631 $$ 40584631 $$ 42427632 $$ 848632 $$ 2311632 $$ 11205632 $$ 15727632 $$ 28262633 $$ 634633 $$ 635633 $$ 636633 $$ 637633 $$ 638633 $$ 639633 $$ 640633 $$ 641633 $$ 642633 $$ 643633 $$ 644633 $$ 645633 $$ 646633 $$ 647633 $$ 849633 $$ 850633 $$ 851633 $$ 1002633 $$ 1178633 $$ 1219633 $$ 1558633 $$ 1949633 $$ 2252633 $$ 3582634 $$ 635634 $$ 636634 $$ 637634 $$ 638634 $$ 639634 $$ 640634 $$ 641634 $$ 642634 $$ 643634 $$ 644634 $$ 645634 $$ 646634 $$ 647634 $$ 849634 $$ 850634 $$ 851634 $$ 1002634 $$ 1178634 $$ 1219634 $$ 1558634 $$ 1949634 $$ 2252634 $$ 3582634 $$ 17913634 $$ 30659635 $$ 636635 $$ 637635 $$ 638635 $$ 639635 $$ 640635 $$ 641635 $$ 642635 $$ 643635 $$ 644635 $$ 645635 $$ 646635 $$ 647635 $$ 849635 $$ 850635 $$ 851635 $$ 1002635 $$ 1178635 $$ 1219635 $$ 1558635 $$ 1949635 $$ 2252635 $$ 3582635 $$ 17913635 $$ 30659636 $$ 637636 $$ 638636 $$ 639636 $$ 640636 $$ 641636 $$ 642636 $$ 643636 $$ 644636 $$ 645636 $$ 646636 $$ 647636 $$ 849636 $$ 850636 $$ 851636 $$ 1002636 $$ 1178636 $$ 1219636 $$ 1558636 $$ 1949636 $$ 2252636 $$ 3582636 $$ 17913636 $$ 30659637 $$ 638637 $$ 639637 $$ 640637 $$ 641637 $$ 642637 $$ 643637 $$ 644637 $$ 645637 $$ 646637 $$ 647637 $$ 849637 $$ 850637 $$ 851637 $$ 1002637 $$ 1178637 $$ 1219637 $$ 1558637 $$ 1949637 $$ 2252637 $$ 3582637 $$ 7343637 $$ 12539637 $$ 13678637 $$ 28464637 $$ 30659638 $$ 639638 $$ 640638 $$ 641638 $$ 642638 $$ 643638 $$ 644638 $$ 645638 $$ 646638 $$ 647638 $$ 849638 $$ 850638 $$ 851638 $$ 1002638 $$ 1178638 $$ 1219638 $$ 1558638 $$ 1949638 $$ 2252638 $$ 3582638 $$ 7343638 $$ 12539638 $$ 13678638 $$ 28464638 $$ 30659639 $$ 640639 $$ 641639 $$ 642639 $$ 643639 $$ 644639 $$ 645639 $$ 646639 $$ 647639 $$ 849639 $$ 850639 $$ 851639 $$ 1002639 $$ 1178639 $$ 1219639 $$ 1558639 $$ 1949639 $$ 2252639 $$ 3582639 $$ 7343639 $$ 12539639 $$ 13678639 $$ 28464639 $$ 30659640 $$ 641640 $$ 642640 $$ 643640 $$ 644640 $$ 645640 $$ 646640 $$ 647640 $$ 849640 $$ 850640 $$ 851640 $$ 1002640 $$ 1178640 $$ 1219640 $$ 1558640 $$ 1949640 $$ 2252640 $$ 3582640 $$ 7343640 $$ 10089640 $$ 12539640 $$ 13678640 $$ 28464640 $$ 30659641 $$ 642641 $$ 643641 $$ 644641 $$ 645641 $$ 646641 $$ 647641 $$ 849641 $$ 850641 $$ 851641 $$ 1002641 $$ 1178641 $$ 1219641 $$ 1558641 $$ 1949641 $$ 2252641 $$ 3582641 $$ 7343641 $$ 10089641 $$ 12539641 $$ 13678641 $$ 28464641 $$ 30659642 $$ 643642 $$ 644642 $$ 645642 $$ 646642 $$ 647642 $$ 849642 $$ 850642 $$ 851642 $$ 1002642 $$ 1178642 $$ 1219642 $$ 1558642 $$ 1949642 $$ 2252642 $$ 3582642 $$ 7343642 $$ 10089642 $$ 12539642 $$ 13678642 $$ 28464642 $$ 30659642 $$ 36592642 $$ 37043643 $$ 644643 $$ 645643 $$ 646643 $$ 647643 $$ 849643 $$ 850643 $$ 851643 $$ 1002643 $$ 1178643 $$ 1219643 $$ 1558643 $$ 1949643 $$ 2252643 $$ 3582643 $$ 7343643 $$ 10089643 $$ 12539643 $$ 13678643 $$ 20629643 $$ 28464643 $$ 30659643 $$ 36592643 $$ 37043644 $$ 645644 $$ 646644 $$ 647644 $$ 849644 $$ 850644 $$ 851644 $$ 1002644 $$ 1178644 $$ 1219644 $$ 1558644 $$ 1949644 $$ 2252644 $$ 3582644 $$ 7343644 $$ 10089644 $$ 13678644 $$ 20629644 $$ 36592644 $$ 37043645 $$ 646645 $$ 647645 $$ 849645 $$ 850645 $$ 851645 $$ 1002645 $$ 1178645 $$ 1219645 $$ 1558645 $$ 1949645 $$ 2252645 $$ 3582645 $$ 7343645 $$ 10089645 $$ 13678645 $$ 20629645 $$ 36592645 $$ 37043646 $$ 647646 $$ 849646 $$ 850646 $$ 851646 $$ 1002646 $$ 1178646 $$ 1219646 $$ 1558646 $$ 1949646 $$ 2252646 $$ 3582646 $$ 7343646 $$ 10089646 $$ 13678646 $$ 20629646 $$ 36592646 $$ 37043647 $$ 849647 $$ 850647 $$ 851647 $$ 1002647 $$ 1178647 $$ 1219647 $$ 1558647 $$ 1949647 $$ 2252647 $$ 3582647 $$ 7343647 $$ 10089647 $$ 13678647 $$ 20629647 $$ 36592647 $$ 37043648 $$ 1105649 $$ 1011649 $$ 26882650 $$ 6728651 $$ 1005651 $$ 1211651 $$ 1395651 $$ 2080651 $$ 2185651 $$ 2420651 $$ 8850651 $$ 12119651 $$ 24440651 $$ 25455651 $$ 32825652 $$ 720652 $$ 1107652 $$ 1110652 $$ 1321652 $$ 1417652 $$ 1456652 $$ 3704652 $$ 9191652 $$ 10571652 $$ 33966653 $$ 797653 $$ 3231653 $$ 4020653 $$ 4305653 $$ 27545654 $$ 3095654 $$ 3213654 $$ 6361654 $$ 6764654 $$ 9524654 $$ 12124654 $$ 17712655 $$ 857655 $$ 1914655 $$ 3326655 $$ 18999655 $$ 25260656 $$ 1008656 $$ 1009656 $$ 1110656 $$ 1663656 $$ 2072656 $$ 6866656 $$ 15805656 $$ 38207656 $$ 38208656 $$ 42880657 $$ 858657 $$ 1010657 $$ 1938657 $$ 5441657 $$ 8740658 $$ 861658 $$ 1062658 $$ 1653658 $$ 1765658 $$ 2546658 $$ 2622658 $$ 22969658 $$ 30363659 $$ 1652659 $$ 2074659 $$ 3182659 $$ 4344660 $$ 1012660 $$ 1049660 $$ 3356660 $$ 3655660 $$ 5237660 $$ 8365660 $$ 11492660 $$ 34738661 $$ 1119662 $$ 2091662 $$ 2170662 $$ 2510662 $$ 4393662 $$ 9100662 $$ 16344662 $$ 26385662 $$ 33896663 $$ 865663 $$ 1457663 $$ 3454663 $$ 7712663 $$ 13423663 $$ 28087663 $$ 31626663 $$ 42722664 $$ 1499664 $$ 1663664 $$ 6959664 $$ 7846664 $$ 14353664 $$ 14354665 $$ 666665 $$ 1323665 $$ 5045665 $$ 6324665 $$ 39525666 $$ 1323666 $$ 5045666 $$ 6324666 $$ 39525667 $$ 668667 $$ 1014667 $$ 1015667 $$ 1115667 $$ 1116669 $$ 4664669 $$ 44200670 $$ 1117670 $$ 1221670 $$ 2403670 $$ 2943670 $$ 4365670 $$ 4530670 $$ 10864670 $$ 12150670 $$ 12151670 $$ 25537671 $$ 3194672 $$ 673672 $$ 867672 $$ 34264672 $$ 35151672 $$ 38928672 $$ 39168672 $$ 41518673 $$ 867673 $$ 957673 $$ 1268673 $$ 3462673 $$ 3463673 $$ 5532673 $$ 9784673 $$ 33007673 $$ 35151674 $$ 1183674 $$ 1222675 $$ 676675 $$ 1458675 $$ 1479675 $$ 8966675 $$ 37194676 $$ 1479676 $$ 1836676 $$ 2999676 $$ 5289676 $$ 6395676 $$ 7101676 $$ 8966676 $$ 22625676 $$ 31259676 $$ 32171677 $$ 716677 $$ 890677 $$ 1122677 $$ 1187677 $$ 1345677 $$ 1561677 $$ 1870677 $$ 2901677 $$ 5323677 $$ 12177677 $$ 14270677 $$ 40476678 $$ 21765678 $$ 26276679 $$ 999679 $$ 1020679 $$ 13230679 $$ 20933679 $$ 22639679 $$ 22640680 $$ 1269680 $$ 23798681 $$ 1560681 $$ 3904681 $$ 25150681 $$ 41369682 $$ 808682 $$ 1292682 $$ 27723682 $$ 43958683 $$ 719683 $$ 878683 $$ 1030683 $$ 1125683 $$ 2768683 $$ 6443683 $$ 10139683 $$ 13218683 $$ 28171684 $$ 685684 $$ 908684 $$ 6539684 $$ 9996684 $$ 21105685 $$ 908685 $$ 6539685 $$ 9996685 $$ 21105686 $$ 1126686 $$ 1284686 $$ 3121686 $$ 7504686 $$ 38376687 $$ 1097687 $$ 1185687 $$ 1980687 $$ 2130688 $$ 1021688 $$ 1053688 $$ 4546688 $$ 25901688 $$ 28870688 $$ 28871689 $$ 690689 $$ 1458689 $$ 1609689 $$ 26322690 $$ 1324690 $$ 1458690 $$ 29029690 $$ 33704690 $$ 40694691 $$ 1223691 $$ 3795691 $$ 8816693 $$ 990693 $$ 1299693 $$ 1359693 $$ 42607694 $$ 1245694 $$ 2080694 $$ 2795694 $$ 4884694 $$ 7317694 $$ 7359694 $$ 9503694 $$ 22478694 $$ 24098694 $$ 26631694 $$ 29489694 $$ 37884694 $$ 42694695 $$ 696695 $$ 1537695 $$ 5862695 $$ 6005695 $$ 10467695 $$ 10515695 $$ 12199695 $$ 13736695 $$ 15048695 $$ 42695695 $$ 43091696 $$ 1537696 $$ 5862696 $$ 6005696 $$ 10467696 $$ 10515696 $$ 12199696 $$ 13736696 $$ 15048696 $$ 42695696 $$ 43091697 $$ 1244697 $$ 1579697 $$ 1673697 $$ 5474697 $$ 9194697 $$ 10516697 $$ 28269697 $$ 29202697 $$ 35917697 $$ 36560697 $$ 39929697 $$ 41113698 $$ 699698 $$ 700698 $$ 2238698 $$ 2505698 $$ 7355698 $$ 7734698 $$ 9863698 $$ 23578698 $$ 25255698 $$ 32254698 $$ 33010698 $$ 40254699 $$ 700699 $$ 2238699 $$ 2505699 $$ 7355699 $$ 7734699 $$ 9863699 $$ 23578699 $$ 25255699 $$ 32254699 $$ 33010699 $$ 40254700 $$ 2238700 $$ 2505700 $$ 7355700 $$ 7734700 $$ 9863700 $$ 23578700 $$ 25255700 $$ 32254700 $$ 33010700 $$ 40254701 $$ 702701 $$ 3754701 $$ 4174701 $$ 12206701 $$ 18083701 $$ 19760702 $$ 3754702 $$ 4174702 $$ 12206702 $$ 18083702 $$ 19760703 $$ 704703 $$ 705703 $$ 706703 $$ 707703 $$ 1226703 $$ 1247703 $$ 8210703 $$ 17671703 $$ 31267703 $$ 31268703 $$ 35338703 $$ 39169704 $$ 705704 $$ 706704 $$ 707704 $$ 1226704 $$ 1247704 $$ 8210704 $$ 31267704 $$ 31268704 $$ 35338704 $$ 41517705 $$ 706705 $$ 707705 $$ 1226705 $$ 1247705 $$ 8210705 $$ 31267705 $$ 31268705 $$ 35338705 $$ 41517706 $$ 707706 $$ 1226706 $$ 1247706 $$ 8210706 $$ 31267706 $$ 31268706 $$ 35338706 $$ 41517707 $$ 1226707 $$ 1247707 $$ 8210707 $$ 31267707 $$ 31268707 $$ 35338707 $$ 41517708 $$ 709708 $$ 710708 $$ 711709 $$ 710709 $$ 711710 $$ 711712 $$ 25253713 $$ 714713 $$ 38750714 $$ 38750715 $$ 2005715 $$ 2844715 $$ 2893715 $$ 12631715 $$ 37866716 $$ 890716 $$ 1122716 $$ 1187716 $$ 1345716 $$ 1870716 $$ 3587716 $$ 9923716 $$ 11608716 $$ 28942716 $$ 30622716 $$ 34200716 $$ 36337716 $$ 38764716 $$ 39139716 $$ 41380716 $$ 41381716 $$ 43548717 $$ 722717 $$ 1026717 $$ 1129717 $$ 1397717 $$ 1652717 $$ 2429717 $$ 4725717 $$ 10748717 $$ 23492717 $$ 24410717 $$ 33568717 $$ 34201718 $$ 2222718 $$ 13231719 $$ 6152719 $$ 7880719 $$ 37289719 $$ 37985720 $$ 1110720 $$ 3704720 $$ 5329720 $$ 37502721 $$ 1398721 $$ 3092721 $$ 4362721 $$ 9338721 $$ 10273721 $$ 12216721 $$ 13238721 $$ 13624721 $$ 20294721 $$ 21450721 $$ 35141722 $$ 1132722 $$ 1273723 $$ 1386723 $$ 1971723 $$ 5602723 $$ 12430723 $$ 29675723 $$ 30409723 $$ 34380723 $$ 36026723 $$ 41970724 $$ 814724 $$ 895724 $$ 1028724 $$ 1605724 $$ 2223724 $$ 2864724 $$ 3191724 $$ 11107724 $$ 26119724 $$ 29999724 $$ 30688725 $$ 896725 $$ 1347725 $$ 13215725 $$ 25634725 $$ 33675725 $$ 38880725 $$ 42406726 $$ 727726 $$ 9036726 $$ 9555726 $$ 12237726 $$ 12925727 $$ 1642727 $$ 29450728 $$ 1006728 $$ 1395728 $$ 1708728 $$ 9424728 $$ 31522729 $$ 11086729 $$ 17183729 $$ 33063729 $$ 35867729 $$ 35868729 $$ 36216729 $$ 37327729 $$ 39465729 $$ 42104730 $$ 1137730 $$ 1304730 $$ 17398730 $$ 20811730 $$ 21726730 $$ 30885730 $$ 32368730 $$ 43090731 $$ 1275731 $$ 1789731 $$ 2262731 $$ 2630731 $$ 3364731 $$ 5125731 $$ 5934731 $$ 8690731 $$ 10726731 $$ 13916731 $$ 14666731 $$ 25442731 $$ 43990731 $$ 43991732 $$ 5329732 $$ 12288732 $$ 22422733 $$ 1589733 $$ 1710733 $$ 12289733 $$ 13741733 $$ 22420733 $$ 29331733 $$ 29332733 $$ 30598734 $$ 1042734 $$ 1228734 $$ 1941734 $$ 28803734 $$ 29375734 $$ 29376734 $$ 32884735 $$ 736735 $$ 910735 $$ 1043735 $$ 1603735 $$ 12875735 $$ 30722736 $$ 910736 $$ 1043736 $$ 1603736 $$ 30043736 $$ 42678737 $$ 911737 $$ 3589737 $$ 21768737 $$ 21769737 $$ 24524738 $$ 739738 $$ 740738 $$ 914738 $$ 1229738 $$ 1230738 $$ 2343738 $$ 2480738 $$ 3746738 $$ 7364738 $$ 8221738 $$ 8702738 $$ 15118738 $$ 22480738 $$ 22481738 $$ 24888738 $$ 30890738 $$ 31616738 $$ 40531739 $$ 740739 $$ 914739 $$ 1229739 $$ 1230739 $$ 2343739 $$ 2480739 $$ 3746739 $$ 7364739 $$ 8221739 $$ 8702739 $$ 15118739 $$ 22480739 $$ 22481739 $$ 24888739 $$ 30890739 $$ 31616739 $$ 40531740 $$ 914740 $$ 1229740 $$ 1230740 $$ 2343740 $$ 2480740 $$ 3746740 $$ 7364740 $$ 8221740 $$ 8702740 $$ 15118740 $$ 22480740 $$ 22481740 $$ 24888740 $$ 30890740 $$ 31616740 $$ 40531741 $$ 1337741 $$ 26316741 $$ 27369742 $$ 28309742 $$ 32885742 $$ 33758742 $$ 36815742 $$ 37540742 $$ 38428742 $$ 39691742 $$ 40553742 $$ 42267743 $$ 744743 $$ 1231743 $$ 5915743 $$ 10228743 $$ 11226743 $$ 20292743 $$ 22462743 $$ 27023743 $$ 28833743 $$ 31293743 $$ 31294743 $$ 43978744 $$ 1231744 $$ 22462744 $$ 27023745 $$ 921745 $$ 10957745 $$ 31681745 $$ 39907745 $$ 39908746 $$ 922747 $$ 924747 $$ 1143747 $$ 4256747 $$ 8381747 $$ 22518747 $$ 24908748 $$ 1234748 $$ 8226748 $$ 20455748 $$ 36635748 $$ 36636749 $$ 930749 $$ 972749 $$ 1051749 $$ 1052749 $$ 1053749 $$ 1054749 $$ 1196749 $$ 1295749 $$ 1591749 $$ 1859749 $$ 3217749 $$ 21592749 $$ 23056749 $$ 26971749 $$ 31472749 $$ 44145750 $$ 751750 $$ 1148750 $$ 1236750 $$ 1429750 $$ 4531750 $$ 4628750 $$ 11338750 $$ 22295751 $$ 752751 $$ 931751 $$ 1148751 $$ 1236751 $$ 1429751 $$ 1927751 $$ 2739751 $$ 3592751 $$ 4531751 $$ 18725751 $$ 28126751 $$ 29482751 $$ 29952751 $$ 38431751 $$ 38432752 $$ 931752 $$ 1236752 $$ 1429752 $$ 6069753 $$ 755753 $$ 1366753 $$ 1603753 $$ 3604753 $$ 4309753 $$ 7395753 $$ 7737753 $$ 13110753 $$ 28127753 $$ 31263753 $$ 31264753 $$ 32663753 $$ 38641753 $$ 40055754 $$ 1443754 $$ 12989754 $$ 22288754 $$ 25219754 $$ 40204755 $$ 1198755 $$ 1309755 $$ 3654755 $$ 6170755 $$ 7738755 $$ 26339755 $$ 28127756 $$ 1309756 $$ 3922756 $$ 10998756 $$ 12352756 $$ 12353756 $$ 13119756 $$ 20349756 $$ 34753757 $$ 12356758 $$ 938758 $$ 1523758 $$ 2190758 $$ 3750759 $$ 1363759 $$ 2431759 $$ 3107759 $$ 3933759 $$ 10259759 $$ 10914759 $$ 10915759 $$ 12898759 $$ 26041759 $$ 31451759 $$ 38216759 $$ 43750760 $$ 2245760 $$ 26040760 $$ 38693760 $$ 39313761 $$ 2006761 $$ 5042762 $$ 1013762 $$ 14291762 $$ 20535762 $$ 38198762 $$ 38577762 $$ 39419762 $$ 40416763 $$ 41236764 $$ 3332764 $$ 6150764 $$ 24533765 $$ 3362765 $$ 19969766 $$ 21866766 $$ 21867767 $$ 1458768 $$ 2074768 $$ 3924768 $$ 11475768 $$ 13126768 $$ 15869768 $$ 16342768 $$ 26363768 $$ 31574768 $$ 42734769 $$ 776769 $$ 1763769 $$ 1932769 $$ 22472769 $$ 24475769 $$ 29078770 $$ 1406770 $$ 1645770 $$ 1646770 $$ 1807770 $$ 1916770 $$ 1946770 $$ 2245770 $$ 2296770 $$ 2561770 $$ 2568770 $$ 2616770 $$ 3452770 $$ 4022770 $$ 4472770 $$ 5743770 $$ 5744770 $$ 5745770 $$ 6169770 $$ 7977770 $$ 8719770 $$ 9642770 $$ 12384770 $$ 27827770 $$ 29659770 $$ 29660770 $$ 42736770 $$ 42737771 $$ 772771 $$ 1749771 $$ 15142771 $$ 37789773 $$ 1816773 $$ 3025773 $$ 3753774 $$ 775776 $$ 2214776 $$ 6704776 $$ 9924776 $$ 26327777 $$ 33832778 $$ 15171778 $$ 21518778 $$ 23731778 $$ 24327778 $$ 24587778 $$ 26479778 $$ 34836778 $$ 36940778 $$ 36979778 $$ 43174779 $$ 780779 $$ 781779 $$ 952779 $$ 1154779 $$ 1206779 $$ 4973779 $$ 12393779 $$ 15534780 $$ 781780 $$ 952780 $$ 1154780 $$ 1206780 $$ 4973780 $$ 12393780 $$ 15534780 $$ 21832780 $$ 27554781 $$ 952781 $$ 1154781 $$ 1206781 $$ 4973781 $$ 12393781 $$ 15534781 $$ 21833782 $$ 953782 $$ 1667782 $$ 3502782 $$ 12395782 $$ 14553782 $$ 16919782 $$ 21834782 $$ 25896782 $$ 25897783 $$ 809783 $$ 31459783 $$ 43170784 $$ 947784 $$ 2720784 $$ 5046784 $$ 32867784 $$ 37785785 $$ 786785 $$ 22404785 $$ 29652786 $$ 22404786 $$ 30429787 $$ 1207787 $$ 1866787 $$ 18138787 $$ 23663787 $$ 37342788 $$ 1721788 $$ 1778788 $$ 10389789 $$ 1207789 $$ 1866789 $$ 2483789 $$ 2629789 $$ 2730789 $$ 2917789 $$ 4580789 $$ 6455789 $$ 6662789 $$ 12668789 $$ 22375789 $$ 22376789 $$ 27791789 $$ 36671789 $$ 39434790 $$ 1982790 $$ 4609790 $$ 31667791 $$ 1208791 $$ 1358791 $$ 1407791 $$ 1615791 $$ 1631791 $$ 1952791 $$ 2815791 $$ 2879791 $$ 12583791 $$ 30498791 $$ 33553791 $$ 33729792 $$ 1333792 $$ 1832792 $$ 2119792 $$ 2130792 $$ 2451792 $$ 2452792 $$ 2467792 $$ 2503792 $$ 3988792 $$ 4527792 $$ 5668792 $$ 30580793 $$ 21035793 $$ 21036794 $$ 2068794 $$ 2242794 $$ 3649794 $$ 10504794 $$ 14317794 $$ 38906794 $$ 41410795 $$ 964796 $$ 1245796 $$ 3972796 $$ 5768796 $$ 16641796 $$ 37416797 $$ 1078797 $$ 1393797 $$ 4020797 $$ 10478797 $$ 11528797 $$ 38832798 $$ 966798 $$ 1212798 $$ 1979798 $$ 22395798 $$ 25832799 $$ 1831799 $$ 1951799 $$ 2064799 $$ 2325799 $$ 6371799 $$ 11828799 $$ 26877800 $$ 1637800 $$ 2054800 $$ 22394801 $$ 3089801 $$ 8190801 $$ 39071802 $$ 1894802 $$ 2065802 $$ 3575802 $$ 3705802 $$ 5864802 $$ 24605802 $$ 24606802 $$ 42464803 $$ 804803 $$ 970803 $$ 1085803 $$ 1607803 $$ 1640803 $$ 1678803 $$ 1684803 $$ 4345803 $$ 9224803 $$ 28978803 $$ 35806803 $$ 40165804 $$ 970804 $$ 1085804 $$ 1607804 $$ 1640804 $$ 1678804 $$ 1684804 $$ 4345804 $$ 11526804 $$ 14656804 $$ 28978804 $$ 41953805 $$ 4874805 $$ 22331806 $$ 2834806 $$ 11047807 $$ 1686807 $$ 2185807 $$ 4516807 $$ 9769807 $$ 23713807 $$ 24315807 $$ 24409807 $$ 28266807 $$ 31411808 $$ 1292808 $$ 27723809 $$ 7037809 $$ 41895810 $$ 963810 $$ 975810 $$ 1089810 $$ 1165810 $$ 2246810 $$ 13321810 $$ 22328810 $$ 28283810 $$ 29314810 $$ 43404811 $$ 969811 $$ 1761811 $$ 2718811 $$ 3843811 $$ 4431811 $$ 4447811 $$ 8457811 $$ 13322811 $$ 14922811 $$ 19148811 $$ 19561811 $$ 42738812 $$ 968812 $$ 42101813 $$ 2536813 $$ 10835813 $$ 11851813 $$ 29446813 $$ 29529813 $$ 34359814 $$ 895814 $$ 1028814 $$ 2864814 $$ 3193814 $$ 3404814 $$ 5483814 $$ 12219814 $$ 18603815 $$ 1852815 $$ 5535815 $$ 7055815 $$ 9219815 $$ 39757815 $$ 40135815 $$ 40136815 $$ 41306816 $$ 4469816 $$ 9894816 $$ 14914816 $$ 26160816 $$ 32051816 $$ 33310816 $$ 42507817 $$ 824817 $$ 2429817 $$ 32472818 $$ 1034818 $$ 1446818 $$ 1450818 $$ 1554818 $$ 1882818 $$ 6033818 $$ 6159818 $$ 7407818 $$ 9318818 $$ 9482818 $$ 9483818 $$ 12563818 $$ 34707819 $$ 844819 $$ 1577819 $$ 1657819 $$ 1855819 $$ 2278819 $$ 2469819 $$ 3075819 $$ 5460819 $$ 6943819 $$ 9067819 $$ 23789819 $$ 27820819 $$ 34228821 $$ 11888822 $$ 1251822 $$ 13699823 $$ 32133823 $$ 40215824 $$ 2429824 $$ 14284824 $$ 43774825 $$ 826825 $$ 827825 $$ 983825 $$ 1094825 $$ 1095825 $$ 1451825 $$ 1533825 $$ 1707825 $$ 2010825 $$ 4278825 $$ 7507825 $$ 8169825 $$ 9486825 $$ 13974825 $$ 16859825 $$ 25415825 $$ 25416825 $$ 27862825 $$ 30415825 $$ 30416825 $$ 32803825 $$ 32804825 $$ 42014826 $$ 827826 $$ 983826 $$ 1094826 $$ 1095826 $$ 1451826 $$ 1533826 $$ 1707826 $$ 2010826 $$ 4278826 $$ 7507826 $$ 8169826 $$ 9486826 $$ 13974826 $$ 16859826 $$ 25415826 $$ 25416826 $$ 27862826 $$ 30415826 $$ 30416826 $$ 32803826 $$ 32804826 $$ 42014827 $$ 983827 $$ 1094827 $$ 1095827 $$ 1451827 $$ 1533827 $$ 1707827 $$ 2010827 $$ 4278827 $$ 7507827 $$ 8169827 $$ 9486827 $$ 13974827 $$ 16859827 $$ 25415827 $$ 25416827 $$ 27862827 $$ 30415827 $$ 30416827 $$ 32803827 $$ 32804828 $$ 830828 $$ 1200828 $$ 1718828 $$ 2319828 $$ 6904828 $$ 10372828 $$ 21994828 $$ 21995828 $$ 21996828 $$ 21997828 $$ 21998829 $$ 19879829 $$ 26091830 $$ 2723830 $$ 14278830 $$ 16913830 $$ 20222830 $$ 22065830 $$ 36192831 $$ 1238831 $$ 23723831 $$ 41921832 $$ 931832 $$ 1056832 $$ 1147832 $$ 2150832 $$ 2635832 $$ 29684832 $$ 38430833 $$ 7648833 $$ 16924833 $$ 19939833 $$ 36756834 $$ 1264834 $$ 1762834 $$ 25761835 $$ 836835 $$ 993835 $$ 1218835 $$ 1833835 $$ 1932835 $$ 3439835 $$ 5860835 $$ 10356835 $$ 13747835 $$ 27957835 $$ 39307835 $$ 42002836 $$ 1218836 $$ 1833837 $$ 992837 $$ 1263837 $$ 3771837 $$ 22074837 $$ 22075837 $$ 32047837 $$ 35827838 $$ 12006838 $$ 43450839 $$ 1415839 $$ 1834839 $$ 4264839 $$ 4410839 $$ 7412839 $$ 13681839 $$ 14114839 $$ 31501840 $$ 853840 $$ 942840 $$ 1004840 $$ 1262840 $$ 1369840 $$ 1687840 $$ 1787840 $$ 2167840 $$ 3467840 $$ 3476840 $$ 4971840 $$ 5704840 $$ 28240841 $$ 1762841 $$ 3079841 $$ 12015841 $$ 28264842 $$ 22693842 $$ 26661842 $$ 39893843 $$ 1264843 $$ 1322843 $$ 1878843 $$ 1999843 $$ 2295843 $$ 4412843 $$ 6476843 $$ 13744843 $$ 13918843 $$ 14692843 $$ 16329843 $$ 23658843 $$ 23863843 $$ 24233843 $$ 25309843 $$ 25573843 $$ 26660843 $$ 42379843 $$ 42583844 $$ 1577844 $$ 2469844 $$ 3213844 $$ 3578844 $$ 5460844 $$ 6943845 $$ 997845 $$ 998845 $$ 1179845 $$ 1188845 $$ 1559845 $$ 1604845 $$ 2019845 $$ 2046845 $$ 2074845 $$ 2469845 $$ 3586845 $$ 3652845 $$ 4979845 $$ 6305845 $$ 13275845 $$ 22296845 $$ 28719846 $$ 1269846 $$ 1645846 $$ 1646846 $$ 1786846 $$ 2101846 $$ 2548846 $$ 2831846 $$ 4129846 $$ 5942846 $$ 6771846 $$ 10876846 $$ 15066846 $$ 32946846 $$ 37660846 $$ 42423847 $$ 3578847 $$ 19629847 $$ 38561848 $$ 2231848 $$ 2311848 $$ 24980848 $$ 28262849 $$ 850849 $$ 851849 $$ 1002849 $$ 1178849 $$ 1219849 $$ 1558849 $$ 1949849 $$ 2252849 $$ 3582849 $$ 12539849 $$ 30659850 $$ 851850 $$ 1002850 $$ 1178850 $$ 1219850 $$ 1558850 $$ 1949850 $$ 2252850 $$ 3582850 $$ 7343850 $$ 10089850 $$ 12539850 $$ 13678850 $$ 28464850 $$ 36592850 $$ 37043851 $$ 1002851 $$ 1178851 $$ 1219851 $$ 1558851 $$ 1949851 $$ 2252851 $$ 3582851 $$ 7343851 $$ 10089851 $$ 12539851 $$ 13678851 $$ 28464851 $$ 30659851 $$ 36592851 $$ 37043852 $$ 2330852 $$ 4277853 $$ 1004853 $$ 21504853 $$ 21848853 $$ 26173854 $$ 6362854 $$ 9524854 $$ 14989854 $$ 17794854 $$ 21852855 $$ 1967855 $$ 6253855 $$ 27661856 $$ 18998857 $$ 1787857 $$ 1906857 $$ 1914857 $$ 2058857 $$ 3310857 $$ 3326857 $$ 25033858 $$ 1010858 $$ 2860858 $$ 4221858 $$ 30750858 $$ 43648859 $$ 1011859 $$ 1608859 $$ 1641859 $$ 1824859 $$ 2116859 $$ 2390859 $$ 2429859 $$ 3139859 $$ 3267859 $$ 5281859 $$ 9248860 $$ 909860 $$ 1161860 $$ 4935860 $$ 20931860 $$ 40500861 $$ 2546861 $$ 22969861 $$ 28750862 $$ 3020862 $$ 14135862 $$ 14494862 $$ 15403862 $$ 25543862 $$ 26191862 $$ 34752863 $$ 1110863 $$ 1437863 $$ 1957863 $$ 3014863 $$ 5019863 $$ 10150863 $$ 26498863 $$ 39654863 $$ 41457864 $$ 1712864 $$ 1927864 $$ 6689864 $$ 7928864 $$ 11473864 $$ 33528865 $$ 1457865 $$ 3632865 $$ 7712865 $$ 15004865 $$ 27418865 $$ 31045865 $$ 31626865 $$ 39530866 $$ 1119866 $$ 8695866 $$ 13626867 $$ 957867 $$ 1268867 $$ 3462867 $$ 3463867 $$ 5532867 $$ 9784867 $$ 35151868 $$ 23070869 $$ 870869 $$ 1017869 $$ 2035869 $$ 2124869 $$ 2771869 $$ 24089870 $$ 1017870 $$ 1346870 $$ 1610870 $$ 2124870 $$ 5154870 $$ 11328870 $$ 34009871 $$ 10505871 $$ 25235872 $$ 2599873 $$ 1937873 $$ 1955873 $$ 2106873 $$ 3791873 $$ 4006873 $$ 19699873 $$ 24711873 $$ 33559873 $$ 38419874 $$ 5531874 $$ 20822875 $$ 1691875 $$ 20161875 $$ 23097875 $$ 36355875 $$ 40663876 $$ 25847876 $$ 25934877 $$ 954877 $$ 1792878 $$ 1029878 $$ 1030878 $$ 1125878 $$ 6443878 $$ 10139878 $$ 16444878 $$ 17006878 $$ 28362878 $$ 36331879 $$ 1022879 $$ 2768879 $$ 3048879 $$ 3316879 $$ 7353879 $$ 8208879 $$ 13196879 $$ 30379879 $$ 38735880 $$ 881880 $$ 1248880 $$ 4035880 $$ 13197880 $$ 17261880 $$ 26248880 $$ 27009880 $$ 38901881 $$ 3330881 $$ 4035881 $$ 13197881 $$ 15129881 $$ 29466881 $$ 31100881 $$ 35911882 $$ 9541882 $$ 12198882 $$ 22641882 $$ 23086883 $$ 884883 $$ 885883 $$ 985883 $$ 1024883 $$ 1025883 $$ 1225883 $$ 5986883 $$ 31275883 $$ 42110884 $$ 885884 $$ 985884 $$ 1024884 $$ 1025884 $$ 1225884 $$ 8703884 $$ 21543884 $$ 21544884 $$ 23120884 $$ 31275884 $$ 42112884 $$ 43000885 $$ 985885 $$ 1024885 $$ 1025885 $$ 1225885 $$ 2961885 $$ 35878886 $$ 1550886 $$ 6913886 $$ 16346886 $$ 16803886 $$ 17157887 $$ 12210888 $$ 2041888 $$ 5200888 $$ 6344888 $$ 18649888 $$ 21033888 $$ 32929889 $$ 1155889 $$ 1972889 $$ 4521889 $$ 7068889 $$ 13817889 $$ 32352890 $$ 1122890 $$ 1187890 $$ 1345890 $$ 1870890 $$ 3587890 $$ 9923890 $$ 11608890 $$ 28942890 $$ 30622890 $$ 34200890 $$ 36337890 $$ 38764890 $$ 39139890 $$ 41380890 $$ 41381890 $$ 43548891 $$ 1795891 $$ 3259891 $$ 3260891 $$ 8818891 $$ 14336891 $$ 16359892 $$ 1325892 $$ 1432892 $$ 1810892 $$ 13718892 $$ 31660892 $$ 33888892 $$ 39514893 $$ 898893 $$ 899893 $$ 1190893 $$ 2537893 $$ 3102893 $$ 13760893 $$ 19993893 $$ 21608893 $$ 22539893 $$ 23095893 $$ 23096894 $$ 1120894 $$ 1561894 $$ 5037894 $$ 12665894 $$ 30028894 $$ 30029894 $$ 41512895 $$ 1028895 $$ 2864895 $$ 11107895 $$ 18603895 $$ 29999895 $$ 30688896 $$ 1347896 $$ 13215896 $$ 33675896 $$ 38880896 $$ 42406897 $$ 1216897 $$ 1702897 $$ 28761898 $$ 899898 $$ 1190898 $$ 2892898 $$ 3102898 $$ 13760898 $$ 20712898 $$ 23095898 $$ 23096898 $$ 25905898 $$ 29688899 $$ 1190899 $$ 2892899 $$ 3102899 $$ 13760899 $$ 20712899 $$ 23095899 $$ 23096899 $$ 29688900 $$ 1032900 $$ 5345900 $$ 21774900 $$ 23044901 $$ 1583901 $$ 29761902 $$ 1258902 $$ 1346902 $$ 1518902 $$ 1581902 $$ 1610902 $$ 2885902 $$ 8118902 $$ 8727902 $$ 13429902 $$ 14580902 $$ 15070902 $$ 25846902 $$ 29504902 $$ 36679903 $$ 1209903 $$ 23529904 $$ 1577904 $$ 1778904 $$ 2508904 $$ 4779904 $$ 25169904 $$ 35706904 $$ 38450905 $$ 1047905 $$ 1293905 $$ 15618905 $$ 29691905 $$ 42003905 $$ 42004906 $$ 9402906 $$ 24073906 $$ 31669906 $$ 35078907 $$ 1039907 $$ 3097907 $$ 14939907 $$ 19886907 $$ 21822907 $$ 23536907 $$ 26081907 $$ 31120907 $$ 31396907 $$ 37722907 $$ 39980908 $$ 1161908 $$ 21105908 $$ 27020909 $$ 1161909 $$ 4644909 $$ 4935909 $$ 29697910 $$ 1043910 $$ 1603910 $$ 30043910 $$ 42678911 $$ 9190911 $$ 25233912 $$ 1276912 $$ 13167912 $$ 17621912 $$ 17662912 $$ 20675912 $$ 27656912 $$ 32356912 $$ 42478912 $$ 42479913 $$ 923913 $$ 1180913 $$ 7567913 $$ 8516913 $$ 29682914 $$ 915914 $$ 1229914 $$ 1230914 $$ 2343914 $$ 2480914 $$ 3746914 $$ 7364914 $$ 13986914 $$ 13987914 $$ 25226914 $$ 30890914 $$ 31616915 $$ 1230915 $$ 1378915 $$ 1448915 $$ 1997915 $$ 5352915 $$ 26244916 $$ 1326916 $$ 2948916 $$ 3738916 $$ 5452916 $$ 6404916 $$ 6562916 $$ 7084916 $$ 10320916 $$ 15514916 $$ 15945916 $$ 22581916 $$ 28213916 $$ 31262917 $$ 1045917 $$ 17440917 $$ 22582918 $$ 1242918 $$ 28868918 $$ 41447919 $$ 1405919 $$ 1883919 $$ 2077919 $$ 2250919 $$ 3692919 $$ 32593920 $$ 5418920 $$ 9812920 $$ 12327920 $$ 22570920 $$ 32268921 $$ 10957921 $$ 31681922 $$ 32263923 $$ 927923 $$ 1379923 $$ 2783923 $$ 2985923 $$ 4583923 $$ 5017923 $$ 7819923 $$ 8116923 $$ 8516923 $$ 17654923 $$ 19736923 $$ 30407923 $$ 43002924 $$ 1143924 $$ 4256924 $$ 8381924 $$ 20456924 $$ 22518924 $$ 24908925 $$ 2767925 $$ 30377926 $$ 1047926 $$ 2997926 $$ 12338926 $$ 28129926 $$ 32541927 $$ 1379927 $$ 2515927 $$ 4617928 $$ 1648928 $$ 40428928 $$ 43369930 $$ 972930 $$ 1051930 $$ 1052930 $$ 1053930 $$ 1054930 $$ 1184930 $$ 1196930 $$ 1295930 $$ 1591930 $$ 21592930 $$ 23056930 $$ 39273930 $$ 39974930 $$ 43837931 $$ 1054931 $$ 1056931 $$ 1236931 $$ 1429931 $$ 1482931 $$ 2719931 $$ 6606931 $$ 16173931 $$ 17881931 $$ 29684932 $$ 2222932 $$ 2671932 $$ 4682932 $$ 5052932 $$ 9483932 $$ 21363932 $$ 41498932 $$ 42194934 $$ 935934 $$ 1499934 $$ 1791934 $$ 2239934 $$ 2520934 $$ 3329934 $$ 4051934 $$ 5377934 $$ 34353934 $$ 38227934 $$ 38228935 $$ 1499935 $$ 1791935 $$ 2520935 $$ 3459935 $$ 3748935 $$ 4058935 $$ 5377935 $$ 38227935 $$ 38228936 $$ 1001936 $$ 1759936 $$ 1918936 $$ 2482936 $$ 9368937 $$ 1350937 $$ 4963937 $$ 5519937 $$ 13636937 $$ 24066937 $$ 30776938 $$ 1523938 $$ 1598938 $$ 2190938 $$ 3750938 $$ 25208939 $$ 1237939 $$ 29946939 $$ 40423940 $$ 1774940 $$ 3937940 $$ 6339941 $$ 1204941 $$ 4212941 $$ 33857941 $$ 37828942 $$ 1369942 $$ 1585942 $$ 1687942 $$ 5969942 $$ 27874942 $$ 35782942 $$ 43176943 $$ 1268943 $$ 1420943 $$ 1716943 $$ 4139943 $$ 6596944 $$ 1564944 $$ 4375944 $$ 5881944 $$ 8591944 $$ 22899945 $$ 1142945 $$ 1556945 $$ 6460945 $$ 7240945 $$ 20358945 $$ 21240945 $$ 44300946 $$ 947946 $$ 1278946 $$ 3499946 $$ 13117946 $$ 20968946 $$ 22927946 $$ 25874946 $$ 42503947 $$ 1278947 $$ 8114947 $$ 13117947 $$ 20968947 $$ 42503948 $$ 1267948 $$ 28105949 $$ 3424949 $$ 7456949 $$ 22470950 $$ 951950 $$ 2919950 $$ 20326950 $$ 22946951 $$ 2919951 $$ 20326951 $$ 22946952 $$ 1154952 $$ 1206952 $$ 4973952 $$ 12393952 $$ 15534953 $$ 1667953 $$ 5035953 $$ 18419953 $$ 18420953 $$ 21834953 $$ 37339954 $$ 1792954 $$ 27124954 $$ 27125954 $$ 38618954 $$ 39061954 $$ 40127955 $$ 1360955 $$ 7722955 $$ 9576955 $$ 19977956 $$ 1819956 $$ 7045956 $$ 13809956 $$ 24206956 $$ 24630957 $$ 4372957 $$ 5532957 $$ 18712957 $$ 20898957 $$ 25657958 $$ 2562958 $$ 7351958 $$ 23740958 $$ 25057958 $$ 35305958 $$ 35830958 $$ 37450960 $$ 1578960 $$ 2817960 $$ 3198960 $$ 4541960 $$ 5496960 $$ 6095960 $$ 11368960 $$ 11654960 $$ 12264960 $$ 23084960 $$ 39848961 $$ 1470961 $$ 4099961 $$ 7587962 $$ 3789962 $$ 5409962 $$ 32236962 $$ 32871962 $$ 33259962 $$ 35234962 $$ 35769962 $$ 37349962 $$ 41431963 $$ 975963 $$ 1089963 $$ 1165963 $$ 2218963 $$ 2246963 $$ 17448963 $$ 22328963 $$ 22977964 $$ 3085964 $$ 17813964 $$ 29440965 $$ 1079965 $$ 3186966 $$ 1769966 $$ 1979966 $$ 10067967 $$ 1081967 $$ 10268967 $$ 35331967 $$ 35332967 $$ 37628969 $$ 1761969 $$ 2648969 $$ 11836969 $$ 13315969 $$ 39765969 $$ 40164970 $$ 1085970 $$ 1607970 $$ 1640970 $$ 1678970 $$ 1684970 $$ 4345970 $$ 9224970 $$ 27654970 $$ 28978970 $$ 35806970 $$ 40165971 $$ 1258971 $$ 1340972 $$ 1051972 $$ 1052972 $$ 1053972 $$ 1054972 $$ 1184972 $$ 1196972 $$ 1295972 $$ 1591972 $$ 3217972 $$ 26972972 $$ 36575972 $$ 39974972 $$ 43837973 $$ 16760973 $$ 21379973 $$ 22410974 $$ 1718974 $$ 13320974 $$ 33952974 $$ 34816974 $$ 38834974 $$ 42500975 $$ 1089975 $$ 1165975 $$ 2218975 $$ 2246975 $$ 22328976 $$ 1638976 $$ 5527977 $$ 1170977 $$ 1400977 $$ 1872977 $$ 2876977 $$ 7066977 $$ 9545977 $$ 11626977 $$ 11902977 $$ 13300977 $$ 26163978 $$ 16479978 $$ 21629978 $$ 35790978 $$ 44090979 $$ 2581979 $$ 2617979 $$ 29959980 $$ 1995980 $$ 4274980 $$ 4391980 $$ 11692980 $$ 11857980 $$ 17271980 $$ 37754981 $$ 1167981 $$ 3554981 $$ 11863981 $$ 13327981 $$ 31427981 $$ 41902982 $$ 1281982 $$ 5651982 $$ 9351982 $$ 35265983 $$ 1094983 $$ 1095983 $$ 1451983 $$ 1533983 $$ 1707983 $$ 2010983 $$ 4278983 $$ 7507983 $$ 8169983 $$ 9486983 $$ 13974983 $$ 16859983 $$ 25415983 $$ 25416983 $$ 27862983 $$ 30415983 $$ 30416983 $$ 42014984 $$ 16477984 $$ 34906984 $$ 34907985 $$ 1024985 $$ 1025985 $$ 1225985 $$ 2961985 $$ 35878986 $$ 5497986 $$ 7224986 $$ 25509986 $$ 31982986 $$ 37037986 $$ 39234987 $$ 1669987 $$ 2780987 $$ 3063987 $$ 3783987 $$ 6863987 $$ 16835987 $$ 18917988 $$ 3828988 $$ 4291988 $$ 5777988 $$ 43378989 $$ 11984989 $$ 26506989 $$ 37443989 $$ 43804990 $$ 1299990 $$ 1421990 $$ 1459990 $$ 10886990 $$ 22597990 $$ 35643990 $$ 35644990 $$ 40650990 $$ 42607991 $$ 1366991 $$ 4458992 $$ 1263992 $$ 3771992 $$ 32047992 $$ 33385992 $$ 35827993 $$ 1566993 $$ 1567993 $$ 1586993 $$ 2297993 $$ 2802993 $$ 4615993 $$ 6400993 $$ 41266994 $$ 1737994 $$ 1874994 $$ 17799995 $$ 996995 $$ 1562995 $$ 9149996 $$ 1562996 $$ 9149997 $$ 998997 $$ 1005997 $$ 1179997 $$ 1454997 $$ 1604997 $$ 1731997 $$ 1786997 $$ 2101997 $$ 2469997 $$ 3652997 $$ 13067997 $$ 13275997 $$ 21656997 $$ 42587998 $$ 1179998 $$ 1454998 $$ 1786998 $$ 2030998 $$ 2101998 $$ 3874998 $$ 10077998 $$ 12019998 $$ 14180998 $$ 16459998 $$ 21656998 $$ 28645998 $$ 33863998 $$ 42587999 $$ 1020999 $$ 1267999 $$ 1343999 $$ 1476999 $$ 1639999 $$ 2851999 $$ 3426999 $$ 27505999 $$ 30475999 $$ 30476999 $$ 429681000 $$ 32111000 $$ 36371000 $$ 75061000 $$ 82091000 $$ 87911000 $$ 91331000 $$ 154371000 $$ 177981000 $$ 240461000 $$ 300141000 $$ 300151000 $$ 306931001 $$ 17591001 $$ 19181001 $$ 24821001 $$ 90621001 $$ 93681001 $$ 209831001 $$ 306361001 $$ 419831002 $$ 11781002 $$ 12191002 $$ 15581002 $$ 19491002 $$ 22521002 $$ 35821002 $$ 306591003 $$ 11341003 $$ 12651003 $$ 91401003 $$ 261711003 $$ 350361003 $$ 350371004 $$ 215041004 $$ 218481004 $$ 261731005 $$ 11231005 $$ 12111005 $$ 31601005 $$ 315201007 $$ 11091007 $$ 121221007 $$ 300701008 $$ 10091008 $$ 11101008 $$ 16631008 $$ 20721008 $$ 68661008 $$ 158051008 $$ 382071008 $$ 382081008 $$ 428801009 $$ 11101009 $$ 16631009 $$ 20721009 $$ 68661009 $$ 382071009 $$ 382081009 $$ 428801010 $$ 28601010 $$ 42211010 $$ 46341010 $$ 296081010 $$ 296091011 $$ 16081011 $$ 16411011 $$ 23901011 $$ 251061011 $$ 328651012 $$ 10491012 $$ 13971012 $$ 16561012 $$ 26051012 $$ 294001013 $$ 48361013 $$ 109661013 $$ 205351013 $$ 385771013 $$ 394191014 $$ 10151014 $$ 11151014 $$ 11161014 $$ 14931014 $$ 329751014 $$ 372981014 $$ 442041015 $$ 11151015 $$ 11161016 $$ 13941016 $$ 17301016 $$ 19441016 $$ 37281016 $$ 121521016 $$ 191611016 $$ 386361016 $$ 404371016 $$ 439271017 $$ 13461017 $$ 16101017 $$ 21241017 $$ 51541017 $$ 113281017 $$ 340091018 $$ 15251018 $$ 19981018 $$ 21821018 $$ 80051018 $$ 158071018 $$ 216411018 $$ 313411019 $$ 13181019 $$ 41171019 $$ 49801019 $$ 56311019 $$ 237531019 $$ 288481020 $$ 132301020 $$ 209331021 $$ 10531021 $$ 45461023 $$ 14361023 $$ 14831023 $$ 29771023 $$ 45331023 $$ 82091023 $$ 90971024 $$ 10251024 $$ 12251024 $$ 59861024 $$ 60501024 $$ 369241024 $$ 389241024 $$ 392441024 $$ 421101024 $$ 438741025 $$ 12251025 $$ 59861025 $$ 60501025 $$ 369241025 $$ 389241025 $$ 392441025 $$ 421101025 $$ 438741026 $$ 11291026 $$ 13971026 $$ 16521026 $$ 24291026 $$ 47251026 $$ 76711026 $$ 107481026 $$ 217181026 $$ 234921026 $$ 244101026 $$ 287231026 $$ 306611026 $$ 342011027 $$ 23771027 $$ 32161027 $$ 46181027 $$ 68601027 $$ 251201027 $$ 386841027 $$ 402061028 $$ 11891028 $$ 19821028 $$ 23591028 $$ 31931028 $$ 122191028 $$ 186031028 $$ 291571029 $$ 10301029 $$ 335721030 $$ 11251030 $$ 170061030 $$ 283621030 $$ 363311030 $$ 369951030 $$ 369961031 $$ 10411031 $$ 20291031 $$ 54131031 $$ 441481032 $$ 18701032 $$ 217741032 $$ 230441032 $$ 245201032 $$ 273021033 $$ 21141033 $$ 31301033 $$ 287591033 $$ 369551034 $$ 13871034 $$ 14501034 $$ 15541034 $$ 17091034 $$ 18821034 $$ 21321034 $$ 33671034 $$ 43331034 $$ 45251034 $$ 48111034 $$ 57151034 $$ 166091034 $$ 200581034 $$ 242151034 $$ 349661034 $$ 349671034 $$ 353731034 $$ 435761035 $$ 11341035 $$ 12651035 $$ 14181035 $$ 30521035 $$ 46791035 $$ 47471035 $$ 51611035 $$ 71031035 $$ 89761035 $$ 107341035 $$ 157491035 $$ 244781035 $$ 254411035 $$ 263091035 $$ 273811035 $$ 429381036 $$ 17641036 $$ 19631036 $$ 22311036 $$ 33681036 $$ 50441036 $$ 309191037 $$ 11351037 $$ 15251037 $$ 347171038 $$ 335211038 $$ 380521038 $$ 389201039 $$ 30971039 $$ 149391039 $$ 198861039 $$ 218221039 $$ 235361039 $$ 260811039 $$ 313961039 $$ 377221040 $$ 27741040 $$ 82251040 $$ 156091040 $$ 378351041 $$ 13411042 $$ 12281042 $$ 12781042 $$ 19411042 $$ 34731043 $$ 16031043 $$ 273311043 $$ 300431043 $$ 426781044 $$ 198101044 $$ 206841044 $$ 224911044 $$ 261181044 $$ 372331044 $$ 396811045 $$ 174401046 $$ 11941046 $$ 13061046 $$ 13071046 $$ 14801046 $$ 17721046 $$ 19231046 $$ 38271046 $$ 72221046 $$ 134831046 $$ 195981046 $$ 396631046 $$ 425361046 $$ 428891047 $$ 15621047 $$ 16041047 $$ 207021047 $$ 296911047 $$ 428921047 $$ 434751049 $$ 33561049 $$ 52371049 $$ 56331049 $$ 57461049 $$ 60171049 $$ 72401049 $$ 83651049 $$ 296581049 $$ 315841049 $$ 384291049 $$ 425301050 $$ 62241050 $$ 104901050 $$ 151461050 $$ 225161050 $$ 281391050 $$ 308941051 $$ 10521051 $$ 10531051 $$ 10541051 $$ 11841051 $$ 11961051 $$ 12951051 $$ 15911051 $$ 215921051 $$ 230561051 $$ 269721051 $$ 399741052 $$ 10531052 $$ 10541052 $$ 11841052 $$ 11961052 $$ 12951052 $$ 15911052 $$ 215921052 $$ 230561052 $$ 350691052 $$ 365751052 $$ 399741052 $$ 438371053 $$ 10541053 $$ 11961053 $$ 12951053 $$ 15911053 $$ 22741053 $$ 32171053 $$ 43941053 $$ 47591053 $$ 314731053 $$ 338721054 $$ 11961054 $$ 12951054 $$ 14471054 $$ 14661054 $$ 14671054 $$ 14711054 $$ 14821054 $$ 14841054 $$ 14851054 $$ 15271054 $$ 15911054 $$ 32171054 $$ 68541054 $$ 314811054 $$ 387441054 $$ 387451054 $$ 387461054 $$ 387471055 $$ 25121055 $$ 62061055 $$ 123471055 $$ 221841055 $$ 221851055 $$ 269681055 $$ 276771055 $$ 389311056 $$ 14431056 $$ 15401056 $$ 20551056 $$ 27191056 $$ 296841056 $$ 368181057 $$ 18851057 $$ 21491057 $$ 50521057 $$ 430851058 $$ 31841058 $$ 113171058 $$ 338181058 $$ 376261059 $$ 19891059 $$ 21451059 $$ 25581059 $$ 26431059 $$ 37601059 $$ 39261059 $$ 42041059 $$ 50401059 $$ 67501059 $$ 109061059 $$ 123501059 $$ 131231060 $$ 16121060 $$ 18221060 $$ 20671060 $$ 21671060 $$ 27201060 $$ 54881061 $$ 17671061 $$ 18021061 $$ 43771061 $$ 216641061 $$ 374201062 $$ 14451062 $$ 14951062 $$ 57481062 $$ 66771062 $$ 73941062 $$ 88911062 $$ 263821062 $$ 294671063 $$ 10751063 $$ 11501063 $$ 13031063 $$ 17481063 $$ 39171063 $$ 116151063 $$ 183711063 $$ 241741063 $$ 256251064 $$ 435091065 $$ 359041066 $$ 15121066 $$ 17251066 $$ 29481066 $$ 284341066 $$ 421211067 $$ 11531067 $$ 14431067 $$ 18651067 $$ 341581067 $$ 401781068 $$ 14271068 $$ 204031068 $$ 308571070 $$ 40471071 $$ 162811071 $$ 412461072 $$ 13771072 $$ 135731072 $$ 295001072 $$ 374361073 $$ 11561073 $$ 16991073 $$ 25731073 $$ 31281073 $$ 39311073 $$ 66291073 $$ 77561073 $$ 87561073 $$ 128131073 $$ 331871073 $$ 349141073 $$ 379471073 $$ 381161073 $$ 381171073 $$ 389701073 $$ 431551074 $$ 22841074 $$ 78131074 $$ 107651074 $$ 198661074 $$ 264501074 $$ 413041075 $$ 11501075 $$ 13031075 $$ 17481075 $$ 20601075 $$ 20911075 $$ 21641075 $$ 21701075 $$ 39171075 $$ 201751075 $$ 241731075 $$ 256241075 $$ 294701075 $$ 328691075 $$ 357731075 $$ 428821075 $$ 428831076 $$ 11601076 $$ 178121076 $$ 279001076 $$ 374171077 $$ 265111077 $$ 328161077 $$ 407041078 $$ 10851078 $$ 12561078 $$ 108411078 $$ 118231078 $$ 137001078 $$ 148821078 $$ 268741079 $$ 22981079 $$ 186981079 $$ 296311080 $$ 15781080 $$ 16811080 $$ 24931080 $$ 32731080 $$ 132951080 $$ 181171080 $$ 374081080 $$ 382501081 $$ 102681081 $$ 353311081 $$ 353321082 $$ 178221083 $$ 13201083 $$ 25331083 $$ 83701083 $$ 115921083 $$ 118371083 $$ 136951083 $$ 168951083 $$ 186881083 $$ 246091083 $$ 301191083 $$ 333491083 $$ 343691083 $$ 349601083 $$ 434211084 $$ 11861084 $$ 16821084 $$ 18131084 $$ 23671084 $$ 150471085 $$ 87311085 $$ 91631085 $$ 268741086 $$ 41871086 $$ 149251086 $$ 149261086 $$ 209051086 $$ 401491087 $$ 43411087 $$ 58651087 $$ 75681087 $$ 75691087 $$ 83511087 $$ 111081087 $$ 154321087 $$ 349991088 $$ 13671088 $$ 39771089 $$ 11651089 $$ 22181089 $$ 22461089 $$ 223281090 $$ 36411090 $$ 42481090 $$ 45021091 $$ 15711091 $$ 25271091 $$ 38971091 $$ 70721091 $$ 186631091 $$ 250871092 $$ 16761092 $$ 33751092 $$ 37131092 $$ 41851092 $$ 75081092 $$ 77641092 $$ 80021092 $$ 133261092 $$ 134711092 $$ 142771092 $$ 146211092 $$ 149131092 $$ 158811092 $$ 162401092 $$ 264431092 $$ 275581092 $$ 286401092 $$ 413141092 $$ 424631092 $$ 434071093 $$ 29621093 $$ 54651094 $$ 10951094 $$ 14511094 $$ 17071094 $$ 51141094 $$ 79971094 $$ 168591094 $$ 328031094 $$ 328041095 $$ 14511095 $$ 15331095 $$ 17071095 $$ 20101095 $$ 42781095 $$ 75071095 $$ 81691095 $$ 94861095 $$ 139741095 $$ 168591095 $$ 278621095 $$ 328031095 $$ 328041096 $$ 14331096 $$ 73021096 $$ 189931096 $$ 339561096 $$ 440931097 $$ 11851097 $$ 21301098 $$ 18101098 $$ 111751098 $$ 290681098 $$ 305161099 $$ 11011099 $$ 12011099 $$ 13501099 $$ 17161099 $$ 26121099 $$ 65111099 $$ 72331099 $$ 102041099 $$ 149521099 $$ 374461099 $$ 410731100 $$ 17641100 $$ 132491100 $$ 399901100 $$ 399911101 $$ 19221101 $$ 28401101 $$ 53021101 $$ 63071101 $$ 95431101 $$ 132801102 $$ 11761102 $$ 22411102 $$ 22641102 $$ 44021102 $$ 137451103 $$ 12971103 $$ 53141103 $$ 434661104 $$ 17381104 $$ 120241104 $$ 199501104 $$ 203091104 $$ 300671104 $$ 414561106 $$ 14171106 $$ 14561106 $$ 18631106 $$ 31681106 $$ 70971106 $$ 71291106 $$ 88691107 $$ 13211107 $$ 14171107 $$ 14471107 $$ 14561107 $$ 14671107 $$ 14711107 $$ 14851107 $$ 14881107 $$ 15031107 $$ 15041107 $$ 15061107 $$ 15941107 $$ 17551107 $$ 404621107 $$ 404631107 $$ 437911107 $$ 437921108 $$ 11201109 $$ 30231109 $$ 41161109 $$ 58891109 $$ 121221110 $$ 37041110 $$ 44061110 $$ 50161111 $$ 46141111 $$ 46841111 $$ 74681111 $$ 94211111 $$ 103411111 $$ 146951111 $$ 389841112 $$ 134231112 $$ 316241112 $$ 316251112 $$ 399461112 $$ 442671113 $$ 47931113 $$ 49891113 $$ 126961113 $$ 312251114 $$ 26131114 $$ 26181114 $$ 28471114 $$ 209461115 $$ 11161115 $$ 14931115 $$ 372981115 $$ 442041117 $$ 12211117 $$ 18091117 $$ 306581118 $$ 17301119 $$ 15951119 $$ 17211119 $$ 18541119 $$ 25071119 $$ 177831119 $$ 225061119 $$ 344811119 $$ 380421119 $$ 389231120 $$ 29151121 $$ 195991121 $$ 196261121 $$ 294861122 $$ 11871122 $$ 13451122 $$ 15611122 $$ 18701122 $$ 121771123 $$ 21101123 $$ 28471123 $$ 58451123 $$ 77241124 $$ 15341124 $$ 75491124 $$ 316751124 $$ 372301124 $$ 426301125 $$ 27681125 $$ 31311125 $$ 64431125 $$ 92931125 $$ 132181125 $$ 281711126 $$ 17081126 $$ 28671126 $$ 30051126 $$ 55411126 $$ 66311126 $$ 81771126 $$ 296931127 $$ 12681127 $$ 175191127 $$ 283001127 $$ 316221128 $$ 12701128 $$ 248801129 $$ 16521129 $$ 22571129 $$ 425981130 $$ 11311130 $$ 13241130 $$ 14191130 $$ 17691130 $$ 17871130 $$ 47451130 $$ 61371130 $$ 122141130 $$ 122151130 $$ 186231130 $$ 281091130 $$ 322611131 $$ 13241131 $$ 17691131 $$ 44631131 $$ 47451131 $$ 49511131 $$ 54781131 $$ 56201131 $$ 61371131 $$ 79721131 $$ 95441131 $$ 122141131 $$ 122151132 $$ 12731132 $$ 13731132 $$ 78041132 $$ 197251132 $$ 287511133 $$ 11401133 $$ 54401133 $$ 128301134 $$ 12651134 $$ 14181134 $$ 30521134 $$ 46791134 $$ 47471134 $$ 51611134 $$ 71031134 $$ 89761134 $$ 107341134 $$ 244781134 $$ 254411134 $$ 263091134 $$ 273811134 $$ 350361134 $$ 350371134 $$ 404581134 $$ 420771134 $$ 429381135 $$ 24411135 $$ 49931135 $$ 54691135 $$ 85461135 $$ 131381136 $$ 70331136 $$ 99511136 $$ 217271136 $$ 365091137 $$ 13041137 $$ 33131137 $$ 208111137 $$ 287281138 $$ 40261138 $$ 206861138 $$ 281171138 $$ 341311138 $$ 383671139 $$ 19421139 $$ 19751139 $$ 19761139 $$ 20031139 $$ 45961139 $$ 81451139 $$ 82231139 $$ 123171139 $$ 123181139 $$ 237591140 $$ 27161141 $$ 92991141 $$ 341591141 $$ 378071141 $$ 396641142 $$ 11511142 $$ 64601142 $$ 72401142 $$ 203581142 $$ 311681143 $$ 17961143 $$ 19911143 $$ 36451143 $$ 224591143 $$ 342541144 $$ 13081144 $$ 30401144 $$ 204571144 $$ 225171144 $$ 249091144 $$ 392451145 $$ 11461145 $$ 18841145 $$ 321691146 $$ 18841146 $$ 321691147 $$ 26351147 $$ 57421147 $$ 137261148 $$ 12361148 $$ 14291148 $$ 45311148 $$ 222951149 $$ 23731149 $$ 147811149 $$ 279631150 $$ 13031150 $$ 17481150 $$ 39171150 $$ 116151150 $$ 256251150 $$ 294701151 $$ 14261151 $$ 14781151 $$ 17881151 $$ 20761151 $$ 125131151 $$ 203571151 $$ 311681151 $$ 401751151 $$ 427441152 $$ 13561152 $$ 315761153 $$ 14431153 $$ 18651153 $$ 341581153 $$ 401781154 $$ 12061154 $$ 49731154 $$ 123931154 $$ 218321155 $$ 15651155 $$ 19721156 $$ 16991156 $$ 25731156 $$ 31281156 $$ 39311156 $$ 66291156 $$ 77561156 $$ 87561156 $$ 128131156 $$ 329091156 $$ 349141156 $$ 379471156 $$ 381161156 $$ 381171156 $$ 389701156 $$ 431551157 $$ 138541157 $$ 140161158 $$ 38201158 $$ 38901158 $$ 48591158 $$ 54671158 $$ 71131158 $$ 81081158 $$ 99571158 $$ 109441158 $$ 133481158 $$ 185081158 $$ 424241158 $$ 432561159 $$ 24051159 $$ 373231160 $$ 18011160 $$ 18231160 $$ 18931160 $$ 25551160 $$ 50361160 $$ 62021160 $$ 170171160 $$ 279001161 $$ 15751161 $$ 53421161 $$ 251671162 $$ 12851162 $$ 12861162 $$ 337911162 $$ 399101163 $$ 29831163 $$ 218701163 $$ 229951164 $$ 12661164 $$ 15251164 $$ 33181164 $$ 77261164 $$ 225041164 $$ 337161165 $$ 22181165 $$ 22461165 $$ 223281165 $$ 229771166 $$ 118551166 $$ 309011167 $$ 118631168 $$ 23051168 $$ 118901168 $$ 350411168 $$ 352281169 $$ 12591169 $$ 12961169 $$ 28461169 $$ 50391169 $$ 67491169 $$ 110481169 $$ 131281169 $$ 284081169 $$ 328731169 $$ 390371170 $$ 119021170 $$ 255991170 $$ 304961171 $$ 35711171 $$ 37181171 $$ 45001171 $$ 114841172 $$ 11791172 $$ 12161172 $$ 12521172 $$ 13881172 $$ 155461172 $$ 348451173 $$ 35291173 $$ 36221173 $$ 98441173 $$ 186931173 $$ 292081174 $$ 23021174 $$ 72361174 $$ 119911174 $$ 256021175 $$ 13221175 $$ 17091175 $$ 26331175 $$ 28261175 $$ 33201175 $$ 34301175 $$ 50301175 $$ 53251175 $$ 56531175 $$ 59241175 $$ 67141175 $$ 68471175 $$ 76801175 $$ 99121175 $$ 106571175 $$ 146171175 $$ 276091176 $$ 22411176 $$ 22641176 $$ 53171176 $$ 137451177 $$ 15981177 $$ 22261177 $$ 29761177 $$ 32941177 $$ 69301177 $$ 95291177 $$ 107321177 $$ 108541178 $$ 12191178 $$ 15581178 $$ 19491178 $$ 22521178 $$ 35821178 $$ 73431178 $$ 100891178 $$ 125391178 $$ 284641178 $$ 306591178 $$ 365921178 $$ 370431179 $$ 14991179 $$ 16631179 $$ 19451179 $$ 31991179 $$ 61321179 $$ 78241179 $$ 307141179 $$ 391291179 $$ 406511180 $$ 13331180 $$ 21191180 $$ 49461180 $$ 64061180 $$ 75671180 $$ 143021180 $$ 146471181 $$ 14411181 $$ 17081181 $$ 19141181 $$ 23741181 $$ 103241181 $$ 121201181 $$ 137491182 $$ 13861182 $$ 22161182 $$ 50961182 $$ 66551182 $$ 171891182 $$ 338901182 $$ 354351182 $$ 354361182 $$ 361701182 $$ 421891182 $$ 429361183 $$ 12221183 $$ 75031183 $$ 89721183 $$ 191651184 $$ 11961184 $$ 12881184 $$ 12951184 $$ 16791184 $$ 34011184 $$ 77391184 $$ 82551184 $$ 89021184 $$ 316721185 $$ 21301186 $$ 18131186 $$ 122041186 $$ 163471186 $$ 391701187 $$ 13451187 $$ 18701187 $$ 35871187 $$ 65231187 $$ 99231187 $$ 116081187 $$ 224751187 $$ 289411187 $$ 363371187 $$ 413801187 $$ 413811187 $$ 422361187 $$ 435481188 $$ 35861188 $$ 48891188 $$ 49791188 $$ 87571188 $$ 254051188 $$ 287191190 $$ 28921190 $$ 31021190 $$ 137601190 $$ 207121191 $$ 236151192 $$ 18911192 $$ 20511192 $$ 22281192 $$ 24041192 $$ 26731192 $$ 39191192 $$ 41301192 $$ 42341192 $$ 54121192 $$ 58421192 $$ 64561192 $$ 94211192 $$ 136031192 $$ 146081192 $$ 152321192 $$ 152331192 $$ 170381192 $$ 173171192 $$ 196061192 $$ 259971192 $$ 265011192 $$ 347341193 $$ 14971193 $$ 15211193 $$ 15361193 $$ 15411193 $$ 15421193 $$ 15571193 $$ 15841193 $$ 18141193 $$ 20041193 $$ 40891193 $$ 51211193 $$ 58771193 $$ 63491193 $$ 63501193 $$ 396601194 $$ 13061194 $$ 13071194 $$ 14801194 $$ 17721194 $$ 19231194 $$ 38271194 $$ 123251194 $$ 195981194 $$ 198031194 $$ 257511194 $$ 396631194 $$ 428891195 $$ 102051195 $$ 204911195 $$ 204921195 $$ 204931195 $$ 204941195 $$ 204951195 $$ 204961195 $$ 204971195 $$ 304061195 $$ 424761196 $$ 12951196 $$ 15911196 $$ 215921196 $$ 230561196 $$ 269721196 $$ 365751196 $$ 399741197 $$ 17491197 $$ 40651197 $$ 255221197 $$ 266671197 $$ 283141198 $$ 18381198 $$ 36541198 $$ 51551198 $$ 57501199 $$ 24821199 $$ 37891199 $$ 47011199 $$ 54091201 $$ 13501201 $$ 15231201 $$ 200481201 $$ 205521201 $$ 236311201 $$ 366761201 $$ 410731201 $$ 428651202 $$ 14041203 $$ 54331203 $$ 137961203 $$ 263561203 $$ 325381204 $$ 42121204 $$ 338571204 $$ 378281205 $$ 70591205 $$ 300011205 $$ 414391206 $$ 49731206 $$ 155341206 $$ 218331207 $$ 18661207 $$ 24831207 $$ 29171208 $$ 16311208 $$ 19521208 $$ 101161208 $$ 205661208 $$ 304981209 $$ 14611209 $$ 207751209 $$ 273791210 $$ 34761210 $$ 53701211 $$ 13951211 $$ 20801211 $$ 21851211 $$ 24201211 $$ 121191211 $$ 328251212 $$ 14221212 $$ 367241213 $$ 25371214 $$ 43281214 $$ 93071214 $$ 334921214 $$ 338811214 $$ 392471215 $$ 406331216 $$ 12521216 $$ 65561216 $$ 155461216 $$ 287611217 $$ 14911217 $$ 300971217 $$ 425441217 $$ 442501218 $$ 13031218 $$ 18331218 $$ 164811219 $$ 15581219 $$ 19491219 $$ 22521219 $$ 35821219 $$ 73431219 $$ 100891219 $$ 125391219 $$ 136781219 $$ 284641221 $$ 13351221 $$ 19991221 $$ 27481221 $$ 108641221 $$ 200791221 $$ 224661221 $$ 306581221 $$ 368761222 $$ 191651223 $$ 37951223 $$ 88161223 $$ 303871224 $$ 299541224 $$ 324861225 $$ 59861225 $$ 231201225 $$ 312751226 $$ 12471226 $$ 82101226 $$ 353381227 $$ 26681228 $$ 19411228 $$ 156111228 $$ 204791229 $$ 12301229 $$ 23431229 $$ 24801229 $$ 37461229 $$ 73641229 $$ 82211229 $$ 308901229 $$ 405311230 $$ 23431230 $$ 24801230 $$ 37461230 $$ 139861230 $$ 139871230 $$ 252261230 $$ 316161231 $$ 224621231 $$ 270231232 $$ 30221232 $$ 200591232 $$ 267141232 $$ 359031233 $$ 208281234 $$ 204551235 $$ 112631236 $$ 14291236 $$ 19271236 $$ 27391236 $$ 35921236 $$ 45311236 $$ 281261236 $$ 299521236 $$ 384311236 $$ 384321237 $$ 282271238 $$ 22911238 $$ 23071238 $$ 26801238 $$ 135891238 $$ 323541239 $$ 37421239 $$ 85891239 $$ 218571239 $$ 428521240 $$ 15101240 $$ 17471240 $$ 26361240 $$ 30461240 $$ 95201240 $$ 210471240 $$ 210481240 $$ 210491240 $$ 210501240 $$ 229781241 $$ 36791241 $$ 45601241 $$ 78121241 $$ 198541242 $$ 277351243 $$ 12641243 $$ 22871243 $$ 297081244 $$ 15791244 $$ 58621244 $$ 91941244 $$ 105161244 $$ 282691244 $$ 292021245 $$ 18491245 $$ 27951245 $$ 73171245 $$ 73591245 $$ 224781245 $$ 426941246 $$ 69091246 $$ 69691246 $$ 148181247 $$ 312671247 $$ 312681247 $$ 415171248 $$ 337841248 $$ 442561250 $$ 22541250 $$ 45651250 $$ 50951250 $$ 64231250 $$ 69621250 $$ 70121250 $$ 75151250 $$ 76101250 $$ 111931250 $$ 127611250 $$ 128291250 $$ 129411250 $$ 228411250 $$ 293041251 $$ 28171251 $$ 136991251 $$ 420321252 $$ 15311252 $$ 19021252 $$ 155461252 $$ 303351252 $$ 306921252 $$ 343541253 $$ 63211253 $$ 118181254 $$ 23911255 $$ 19341255 $$ 153901256 $$ 14571257 $$ 141511257 $$ 144901257 $$ 254701257 $$ 363911257 $$ 414901258 $$ 13461258 $$ 15181258 $$ 15811258 $$ 16101258 $$ 28851258 $$ 87271258 $$ 134291258 $$ 145801258 $$ 150701258 $$ 258461259 $$ 135861259 $$ 337721259 $$ 390981260 $$ 287211261 $$ 13901261 $$ 14371261 $$ 14541261 $$ 24271261 $$ 39471261 $$ 40331261 $$ 47461261 $$ 66551261 $$ 377141261 $$ 393251262 $$ 16871262 $$ 19191262 $$ 34671262 $$ 34771262 $$ 311291262 $$ 396371264 $$ 102751264 $$ 137441264 $$ 238631264 $$ 253091264 $$ 425831265 $$ 14181265 $$ 25261265 $$ 30521265 $$ 32011265 $$ 46791265 $$ 47471265 $$ 51611265 $$ 71031265 $$ 157491265 $$ 227681265 $$ 244781265 $$ 263091265 $$ 273811265 $$ 350361265 $$ 350371265 $$ 429381266 $$ 16921266 $$ 24491266 $$ 76061266 $$ 225551266 $$ 389181266 $$ 389191267 $$ 15351267 $$ 21991267 $$ 22241267 $$ 22671267 $$ 296381267 $$ 307121267 $$ 343461267 $$ 412551268 $$ 19271268 $$ 37251268 $$ 438671269 $$ 17691269 $$ 25191269 $$ 132291269 $$ 376991269 $$ 379881270 $$ 248801271 $$ 19401271 $$ 31381271 $$ 61351271 $$ 317011272 $$ 19081272 $$ 30501272 $$ 54481272 $$ 62881272 $$ 77041272 $$ 238101272 $$ 262671272 $$ 323021273 $$ 13731273 $$ 78041273 $$ 197251273 $$ 296141273 $$ 316471274 $$ 13031274 $$ 17761274 $$ 122781274 $$ 273801274 $$ 381011275 $$ 17891275 $$ 22621275 $$ 26301275 $$ 33641275 $$ 51251275 $$ 59341275 $$ 86901275 $$ 139161275 $$ 179421275 $$ 184861275 $$ 202871275 $$ 272971275 $$ 337571275 $$ 439901275 $$ 439911276 $$ 59941276 $$ 176211276 $$ 176621276 $$ 206751276 $$ 290751276 $$ 323561276 $$ 424781276 $$ 424791277 $$ 39561277 $$ 46361277 $$ 58891277 $$ 62921277 $$ 89241277 $$ 123391278 $$ 16491278 $$ 16501278 $$ 26981278 $$ 131171278 $$ 229271280 $$ 15491280 $$ 18811280 $$ 23201280 $$ 36851280 $$ 61901280 $$ 64701280 $$ 64711280 $$ 64721280 $$ 73991280 $$ 132041280 $$ 237001280 $$ 277551280 $$ 324511280 $$ 343841280 $$ 343851281 $$ 56511281 $$ 340511281 $$ 418961281 $$ 418971282 $$ 27941282 $$ 36261282 $$ 36271282 $$ 83571282 $$ 98661282 $$ 157561282 $$ 242651282 $$ 270501283 $$ 17801283 $$ 18151283 $$ 18291283 $$ 18471283 $$ 20561283 $$ 21031283 $$ 29421283 $$ 29701283 $$ 42631283 $$ 44871283 $$ 47181283 $$ 49871283 $$ 74251283 $$ 74551283 $$ 81381283 $$ 139631283 $$ 209851283 $$ 224031283 $$ 226241283 $$ 428971284 $$ 26981284 $$ 75041285 $$ 12861285 $$ 314011285 $$ 386401285 $$ 399101286 $$ 23461286 $$ 29281286 $$ 314011286 $$ 399101287 $$ 14111287 $$ 75401287 $$ 320101287 $$ 392771288 $$ 58441288 $$ 395061289 $$ 19911289 $$ 61071289 $$ 146711289 $$ 168971289 $$ 176001289 $$ 413021290 $$ 14721290 $$ 14731290 $$ 23881291 $$ 71371291 $$ 424351292 $$ 14411292 $$ 19141292 $$ 118931293 $$ 92141293 $$ 131321293 $$ 347511294 $$ 13511294 $$ 25601294 $$ 36051294 $$ 160931294 $$ 290721294 $$ 307441294 $$ 386661295 $$ 399741296 $$ 131281297 $$ 17041297 $$ 160571298 $$ 14391298 $$ 19221298 $$ 20921298 $$ 34351298 $$ 102851298 $$ 334721298 $$ 442861299 $$ 14211299 $$ 14591299 $$ 26681299 $$ 108861299 $$ 225971299 $$ 244821299 $$ 406501299 $$ 426071300 $$ 25981300 $$ 45741300 $$ 82321300 $$ 108151300 $$ 151271300 $$ 255341300 $$ 337701301 $$ 93391301 $$ 122421301 $$ 147981302 $$ 31401303 $$ 17481303 $$ 19731303 $$ 23671303 $$ 122781303 $$ 358651304 $$ 237621305 $$ 55771306 $$ 13071306 $$ 14801306 $$ 17721306 $$ 19231306 $$ 38271306 $$ 123251306 $$ 195981306 $$ 198031306 $$ 257511306 $$ 396631306 $$ 428891307 $$ 14801307 $$ 17721307 $$ 19231307 $$ 38271307 $$ 72221307 $$ 195981307 $$ 257511307 $$ 396631307 $$ 428891308 $$ 30401308 $$ 97511308 $$ 204571308 $$ 392451309 $$ 33611309 $$ 43091309 $$ 55341309 $$ 77381309 $$ 109981309 $$ 123521309 $$ 263391311 $$ 14701311 $$ 20251311 $$ 21521311 $$ 40991311 $$ 59241311 $$ 73141311 $$ 79531311 $$ 86681311 $$ 104581311 $$ 218111311 $$ 229921311 $$ 275141311 $$ 427201312 $$ 19931312 $$ 84381312 $$ 272301312 $$ 363181313 $$ 13141313 $$ 159321313 $$ 230931313 $$ 307401313 $$ 307411314 $$ 159321314 $$ 230931314 $$ 307401314 $$ 307411315 $$ 16181315 $$ 16241315 $$ 17751315 $$ 20081315 $$ 22651315 $$ 24151315 $$ 24331315 $$ 24541315 $$ 53781315 $$ 117241315 $$ 333061316 $$ 15891316 $$ 17101316 $$ 51311316 $$ 64681316 $$ 389421317 $$ 13681317 $$ 13851318 $$ 15281318 $$ 21741318 $$ 24751318 $$ 29031318 $$ 41171318 $$ 49801318 $$ 56311318 $$ 207491318 $$ 288481319 $$ 240371320 $$ 240941321 $$ 14171321 $$ 14561322 $$ 14461322 $$ 17061322 $$ 22951322 $$ 23991322 $$ 33391322 $$ 37431322 $$ 43011322 $$ 47311322 $$ 65241322 $$ 85191322 $$ 95591322 $$ 126861322 $$ 127161322 $$ 138801322 $$ 185201323 $$ 21991323 $$ 22241323 $$ 22671323 $$ 33251323 $$ 50451323 $$ 92951323 $$ 92961323 $$ 277171323 $$ 341991323 $$ 434321324 $$ 14191324 $$ 14581324 $$ 17871324 $$ 76071324 $$ 186231324 $$ 274881325 $$ 14321325 $$ 20171326 $$ 29481326 $$ 37381326 $$ 54521326 $$ 64041326 $$ 65621326 $$ 103201326 $$ 159451326 $$ 225811326 $$ 282131326 $$ 312621327 $$ 27081327 $$ 28301327 $$ 42591327 $$ 336891327 $$ 441491328 $$ 252211328 $$ 303921328 $$ 376761329 $$ 31171329 $$ 258581329 $$ 315801329 $$ 399021330 $$ 16051330 $$ 16151330 $$ 22231330 $$ 49931330 $$ 59291330 $$ 85471330 $$ 85491330 $$ 86931330 $$ 104421330 $$ 169711330 $$ 177931330 $$ 255741330 $$ 255751330 $$ 338521330 $$ 382061330 $$ 434691331 $$ 26491331 $$ 32781331 $$ 62461331 $$ 70911331 $$ 76501331 $$ 268811331 $$ 361901331 $$ 382841331 $$ 390591332 $$ 13641332 $$ 15021332 $$ 38511332 $$ 51231332 $$ 75931332 $$ 192001332 $$ 333691333 $$ 21191333 $$ 29381333 $$ 37071333 $$ 39881333 $$ 49881333 $$ 64061333 $$ 106041334 $$ 157021334 $$ 294931334 $$ 315751335 $$ 200791335 $$ 426151335 $$ 426161336 $$ 16581336 $$ 45561337 $$ 15301337 $$ 18851337 $$ 19941337 $$ 22881337 $$ 61741337 $$ 109231337 $$ 137711337 $$ 193281337 $$ 330921338 $$ 21541338 $$ 260311339 $$ 149671339 $$ 199411339 $$ 436561340 $$ 54591340 $$ 216161341 $$ 14211341 $$ 16631341 $$ 33811341 $$ 204831341 $$ 287111342 $$ 29051342 $$ 205241342 $$ 323471343 $$ 14761343 $$ 16071343 $$ 16391343 $$ 16701343 $$ 28511343 $$ 34261343 $$ 36581343 $$ 54351343 $$ 261991343 $$ 433841344 $$ 42151344 $$ 87181344 $$ 111581344 $$ 377911344 $$ 436521345 $$ 18701345 $$ 35871345 $$ 99231345 $$ 116081345 $$ 224751345 $$ 342001345 $$ 363371345 $$ 387641345 $$ 422361345 $$ 435481346 $$ 15381346 $$ 15811346 $$ 16101346 $$ 37731346 $$ 79441346 $$ 129791346 $$ 143401346 $$ 180821347 $$ 79921347 $$ 103261347 $$ 199251347 $$ 288761348 $$ 14251348 $$ 29911348 $$ 30411348 $$ 38291348 $$ 170231348 $$ 202521348 $$ 241001348 $$ 252281348 $$ 252291349 $$ 14631349 $$ 14961349 $$ 28031349 $$ 47581349 $$ 81801349 $$ 97431349 $$ 103091349 $$ 202881349 $$ 202891349 $$ 263341349 $$ 352031349 $$ 357341349 $$ 415261350 $$ 15231350 $$ 136361350 $$ 155821350 $$ 236311350 $$ 282981350 $$ 366761350 $$ 410731351 $$ 25601351 $$ 36051351 $$ 136381351 $$ 160931351 $$ 290721351 $$ 386661352 $$ 16661352 $$ 123831353 $$ 134151353 $$ 268341353 $$ 301321353 $$ 335981354 $$ 13961354 $$ 34991354 $$ 52131354 $$ 104551354 $$ 244501354 $$ 318731354 $$ 385731355 $$ 13821355 $$ 14811355 $$ 66981355 $$ 94571355 $$ 342931356 $$ 131161356 $$ 401761357 $$ 67011358 $$ 20001358 $$ 335531358 $$ 357591358 $$ 357601358 $$ 402191359 $$ 16091359 $$ 111841360 $$ 17721360 $$ 19331360 $$ 63521360 $$ 159681360 $$ 199771360 $$ 263251360 $$ 442981361 $$ 15241361 $$ 122791361 $$ 224961361 $$ 252461361 $$ 380981362 $$ 114381362 $$ 173061362 $$ 356961362 $$ 372191363 $$ 31071363 $$ 39331363 $$ 260411363 $$ 314511363 $$ 382161363 $$ 386921364 $$ 15021364 $$ 34941364 $$ 38511364 $$ 51231364 $$ 75931365 $$ 50441365 $$ 200361365 $$ 308211365 $$ 343091366 $$ 36041366 $$ 44581366 $$ 73951366 $$ 77371366 $$ 326631366 $$ 400551367 $$ 22071367 $$ 264331367 $$ 367351367 $$ 412931368 $$ 135531368 $$ 135541368 $$ 205861368 $$ 296111369 $$ 16871369 $$ 24211369 $$ 46071369 $$ 62201369 $$ 71681369 $$ 200471369 $$ 278471369 $$ 321801370 $$ 15001370 $$ 15011370 $$ 15441370 $$ 16711370 $$ 20731370 $$ 33521370 $$ 35841370 $$ 40081370 $$ 44681370 $$ 47441370 $$ 47951370 $$ 54391370 $$ 63971370 $$ 80651370 $$ 126541370 $$ 130141370 $$ 141581370 $$ 166741370 $$ 173641370 $$ 178371370 $$ 343411370 $$ 394311371 $$ 29921371 $$ 115751372 $$ 18261372 $$ 23661372 $$ 61621373 $$ 20881373 $$ 243021373 $$ 274311373 $$ 307661373 $$ 434741374 $$ 22921374 $$ 30431374 $$ 73361374 $$ 95571374 $$ 122691374 $$ 123711374 $$ 201501374 $$ 363491375 $$ 15111376 $$ 342851377 $$ 45141377 $$ 137681378 $$ 14981378 $$ 21371378 $$ 21381378 $$ 21391378 $$ 21401378 $$ 21411378 $$ 21801378 $$ 21811378 $$ 25061378 $$ 26371378 $$ 51101378 $$ 230261378 $$ 230271379 $$ 27831379 $$ 29851379 $$ 45831379 $$ 50171379 $$ 78191379 $$ 81161379 $$ 197361379 $$ 430021380 $$ 16591380 $$ 22801380 $$ 33081380 $$ 33091380 $$ 47421380 $$ 119951381 $$ 14811381 $$ 27301381 $$ 40261382 $$ 18041382 $$ 18201382 $$ 18281382 $$ 18881382 $$ 20521382 $$ 20561382 $$ 22731382 $$ 224271382 $$ 224281382 $$ 224291382 $$ 342931382 $$ 343741382 $$ 437081383 $$ 16571383 $$ 18601383 $$ 18801383 $$ 19091383 $$ 45241383 $$ 240751384 $$ 19011384 $$ 337311385 $$ 358051386 $$ 22161386 $$ 52531386 $$ 243991386 $$ 310721387 $$ 14461387 $$ 15541387 $$ 21321387 $$ 24291387 $$ 26081387 $$ 45251387 $$ 48111387 $$ 57151387 $$ 115121387 $$ 176851387 $$ 198331387 $$ 349661387 $$ 349671387 $$ 356811387 $$ 358731387 $$ 422051388 $$ 198741388 $$ 283421389 $$ 33321389 $$ 40421389 $$ 43141389 $$ 109201389 $$ 337921390 $$ 14371390 $$ 24271390 $$ 40331390 $$ 66551390 $$ 80031390 $$ 377141390 $$ 393251391 $$ 348711392 $$ 73501392 $$ 77681392 $$ 324431392 $$ 330441392 $$ 410131393 $$ 14551393 $$ 17121393 $$ 121091393 $$ 150161394 $$ 21021394 $$ 143391395 $$ 21851395 $$ 24201395 $$ 47391395 $$ 121191395 $$ 252771396 $$ 48321396 $$ 70621396 $$ 210061398 $$ 22631398 $$ 29311398 $$ 40371398 $$ 57311398 $$ 357971399 $$ 20961400 $$ 18721400 $$ 32151400 $$ 36151400 $$ 95451400 $$ 115991400 $$ 416001401 $$ 32301401 $$ 105901401 $$ 122281401 $$ 154391401 $$ 331201401 $$ 331211402 $$ 24881402 $$ 33631402 $$ 39551402 $$ 41321402 $$ 41951402 $$ 50511402 $$ 57471402 $$ 79401402 $$ 82241402 $$ 90331402 $$ 90341402 $$ 131461402 $$ 131471402 $$ 248941403 $$ 20741403 $$ 30411403 $$ 202951403 $$ 318941403 $$ 370201403 $$ 381331403 $$ 433231404 $$ 43281404 $$ 173601404 $$ 200321405 $$ 15971405 $$ 21621405 $$ 263381406 $$ 18071406 $$ 25611406 $$ 56231407 $$ 16151407 $$ 16311407 $$ 28151407 $$ 28791407 $$ 125831407 $$ 337291408 $$ 68901408 $$ 217061409 $$ 21231409 $$ 30761409 $$ 127541409 $$ 144481409 $$ 153021410 $$ 17841411 $$ 18211413 $$ 419421414 $$ 20331414 $$ 21351414 $$ 26141415 $$ 18341415 $$ 42641415 $$ 136811416 $$ 20791416 $$ 382381417 $$ 14561417 $$ 70971417 $$ 71291417 $$ 252761418 $$ 30521418 $$ 47471418 $$ 216041419 $$ 18851419 $$ 24031419 $$ 123901419 $$ 186231419 $$ 315521419 $$ 366641420 $$ 56901420 $$ 68891420 $$ 256211420 $$ 262531420 $$ 435471421 $$ 25651421 $$ 30341421 $$ 169911421 $$ 406501421 $$ 442331422 $$ 14411422 $$ 15701422 $$ 22651422 $$ 50691422 $$ 50781423 $$ 198211424 $$ 15681424 $$ 33501424 $$ 42571424 $$ 312511424 $$ 312521425 $$ 29911425 $$ 30591425 $$ 31031425 $$ 32241425 $$ 47541425 $$ 244631425 $$ 259331425 $$ 316761425 $$ 442591426 $$ 20261426 $$ 343601427 $$ 20981427 $$ 22591427 $$ 52351427 $$ 299221427 $$ 338431428 $$ 48121428 $$ 116511428 $$ 242981429 $$ 19271429 $$ 27391429 $$ 35921429 $$ 187251429 $$ 281261429 $$ 299521429 $$ 384311429 $$ 384321430 $$ 15491430 $$ 16331430 $$ 16341430 $$ 16581430 $$ 25791430 $$ 26571430 $$ 26581430 $$ 33501430 $$ 38641430 $$ 109291430 $$ 137891430 $$ 206601430 $$ 276591430 $$ 309371430 $$ 337241431 $$ 46911431 $$ 51871431 $$ 51881431 $$ 71251431 $$ 78621431 $$ 90221431 $$ 92451431 $$ 92611431 $$ 93251431 $$ 102121431 $$ 105351431 $$ 109021431 $$ 109351431 $$ 109381431 $$ 115321431 $$ 135441431 $$ 135451431 $$ 136121431 $$ 144041431 $$ 144071431 $$ 149231431 $$ 149451431 $$ 172661431 $$ 243001431 $$ 327931431 $$ 365871431 $$ 365881431 $$ 367311432 $$ 17051432 $$ 29901432 $$ 38161433 $$ 73021433 $$ 116851433 $$ 339561433 $$ 440931434 $$ 21631434 $$ 136941434 $$ 142211434 $$ 142221434 $$ 155081434 $$ 278241434 $$ 286041434 $$ 405541434 $$ 414421436 $$ 16811436 $$ 29771436 $$ 61831436 $$ 171691437 $$ 26601437 $$ 40331437 $$ 136831437 $$ 414581438 $$ 28281438 $$ 322321439 $$ 19221439 $$ 20921439 $$ 54141439 $$ 102851439 $$ 158581439 $$ 300731439 $$ 323711440 $$ 17991440 $$ 207181440 $$ 207191440 $$ 229441441 $$ 19141441 $$ 20911441 $$ 21701441 $$ 33261441 $$ 50781441 $$ 58451441 $$ 79951441 $$ 343451441 $$ 367291442 $$ 163451442 $$ 214291442 $$ 214301443 $$ 383221443 $$ 401781444 $$ 37381444 $$ 341571444 $$ 352061446 $$ 23991446 $$ 36161446 $$ 65241446 $$ 158251446 $$ 158261446 $$ 334321446 $$ 334331446 $$ 415011447 $$ 14671447 $$ 14711447 $$ 14851447 $$ 14881447 $$ 15031447 $$ 15041447 $$ 15051447 $$ 15061447 $$ 15941447 $$ 16051447 $$ 17551447 $$ 18011447 $$ 18231447 $$ 68541447 $$ 217991447 $$ 218001447 $$ 286171447 $$ 348701447 $$ 404621447 $$ 404631448 $$ 20961448 $$ 53521448 $$ 73981448 $$ 165021448 $$ 217981450 $$ 15541450 $$ 18821450 $$ 48111450 $$ 61591450 $$ 94831450 $$ 125631450 $$ 347071450 $$ 412191450 $$ 429961451 $$ 17071451 $$ 328031451 $$ 328041454 $$ 16931454 $$ 17131454 $$ 17171454 $$ 17861454 $$ 17941454 $$ 21011454 $$ 47461454 $$ 47591454 $$ 120191454 $$ 169681454 $$ 405811454 $$ 425871454 $$ 436731455 $$ 16121455 $$ 49241455 $$ 56161455 $$ 65181455 $$ 66561455 $$ 76641455 $$ 76651455 $$ 102821455 $$ 111371455 $$ 112761455 $$ 115021455 $$ 121091455 $$ 145921455 $$ 156231455 $$ 214641455 $$ 230691455 $$ 276441455 $$ 289081455 $$ 304081455 $$ 361071455 $$ 369681455 $$ 426971455 $$ 438001456 $$ 70971456 $$ 71291456 $$ 252761457 $$ 77121458 $$ 14791458 $$ 371941459 $$ 356431459 $$ 356441459 $$ 439411460 $$ 14681460 $$ 14691460 $$ 34791460 $$ 423871460 $$ 436541460 $$ 436551461 $$ 420801462 $$ 31711462 $$ 60841462 $$ 209521462 $$ 217311462 $$ 224841463 $$ 17951463 $$ 28031463 $$ 131551463 $$ 301461464 $$ 352601465 $$ 316781466 $$ 14841466 $$ 15271466 $$ 228291466 $$ 236791466 $$ 236801466 $$ 434821466 $$ 434831467 $$ 14711467 $$ 14851467 $$ 14881467 $$ 15031467 $$ 15041467 $$ 15051467 $$ 15061467 $$ 15941467 $$ 17551467 $$ 18011467 $$ 18231467 $$ 68541467 $$ 217991467 $$ 218001467 $$ 348701467 $$ 404621467 $$ 404631468 $$ 14691468 $$ 34791468 $$ 235871468 $$ 235881468 $$ 423871468 $$ 436541468 $$ 436551469 $$ 34791469 $$ 37611470 $$ 45481470 $$ 75871470 $$ 86311470 $$ 88141470 $$ 90201470 $$ 101991470 $$ 142591470 $$ 188891470 $$ 214561470 $$ 286611470 $$ 421981470 $$ 440351471 $$ 14851471 $$ 14881471 $$ 15031471 $$ 15041471 $$ 15051471 $$ 15061471 $$ 15941471 $$ 17551471 $$ 18011471 $$ 18231471 $$ 68541471 $$ 217991471 $$ 218001471 $$ 348701471 $$ 404621471 $$ 404631472 $$ 14731472 $$ 311281473 $$ 22351473 $$ 367521473 $$ 369361474 $$ 22191474 $$ 26081474 $$ 35731474 $$ 141731475 $$ 24281476 $$ 16391476 $$ 16701476 $$ 16941476 $$ 28511476 $$ 34261476 $$ 36581476 $$ 43761476 $$ 82031476 $$ 304751476 $$ 304761476 $$ 433841477 $$ 32491477 $$ 261961478 $$ 17881478 $$ 20761478 $$ 295531478 $$ 302611478 $$ 436051479 $$ 45181479 $$ 106351479 $$ 122441479 $$ 141661479 $$ 230491479 $$ 335611479 $$ 371941479 $$ 378831480 $$ 17721480 $$ 19231480 $$ 123251480 $$ 195981480 $$ 198031480 $$ 396631480 $$ 428891481 $$ 421651481 $$ 421831482 $$ 141471483 $$ 25111483 $$ 330611483 $$ 372141483 $$ 424041484 $$ 15271484 $$ 228291484 $$ 236791484 $$ 236801484 $$ 434821484 $$ 434831485 $$ 14881485 $$ 15031485 $$ 15041485 $$ 15051485 $$ 15061485 $$ 15941485 $$ 17551485 $$ 18011485 $$ 18231485 $$ 68541485 $$ 217991485 $$ 218001485 $$ 348701485 $$ 404621485 $$ 404631486 $$ 23961486 $$ 30081486 $$ 38841486 $$ 43521486 $$ 163581486 $$ 273221486 $$ 391351487 $$ 20551487 $$ 28991487 $$ 40021487 $$ 88461487 $$ 249761487 $$ 255821487 $$ 343961487 $$ 418281488 $$ 15031488 $$ 15041488 $$ 15051488 $$ 15061488 $$ 15941488 $$ 17551488 $$ 18011488 $$ 18231488 $$ 63201488 $$ 68541488 $$ 348701488 $$ 404621488 $$ 404631489 $$ 15201489 $$ 86691489 $$ 93751489 $$ 112751489 $$ 114601489 $$ 120091489 $$ 125701489 $$ 138151489 $$ 162341489 $$ 165361489 $$ 200341489 $$ 204381489 $$ 229361489 $$ 246391489 $$ 249221489 $$ 325571489 $$ 350571489 $$ 394411489 $$ 404081489 $$ 410761489 $$ 420591489 $$ 426921489 $$ 429541489 $$ 433721489 $$ 438221490 $$ 87521490 $$ 209621490 $$ 209631490 $$ 254561491 $$ 264211492 $$ 18081492 $$ 44761492 $$ 48241492 $$ 60401492 $$ 346881493 $$ 177611493 $$ 372981494 $$ 21611494 $$ 24701494 $$ 39081494 $$ 114741494 $$ 126211494 $$ 168141494 $$ 202091494 $$ 202101494 $$ 264191494 $$ 400531495 $$ 18941495 $$ 23101495 $$ 57481495 $$ 73941495 $$ 353891495 $$ 426761496 $$ 17771496 $$ 47581496 $$ 48871496 $$ 51911496 $$ 53091496 $$ 78421496 $$ 237611496 $$ 263241496 $$ 415261497 $$ 15211497 $$ 15361497 $$ 15411497 $$ 15421497 $$ 15571497 $$ 15841497 $$ 18141497 $$ 20041497 $$ 38541497 $$ 41471497 $$ 75991497 $$ 213271497 $$ 252301497 $$ 290981497 $$ 363511497 $$ 400101498 $$ 20471498 $$ 387081499 $$ 16631499 $$ 17911499 $$ 53771499 $$ 69591500 $$ 15011500 $$ 15441500 $$ 16711500 $$ 20731500 $$ 33521500 $$ 40081500 $$ 44681500 $$ 51601500 $$ 54391500 $$ 80651500 $$ 126541500 $$ 130141500 $$ 141581500 $$ 173641500 $$ 178371500 $$ 205691500 $$ 343411500 $$ 394311500 $$ 402211500 $$ 402221500 $$ 425681501 $$ 15441501 $$ 16711501 $$ 20731501 $$ 33521501 $$ 54391501 $$ 80651501 $$ 126541501 $$ 130141501 $$ 141581501 $$ 173641501 $$ 178371501 $$ 343411501 $$ 394311501 $$ 425681502 $$ 38511502 $$ 51231503 $$ 15041503 $$ 15051503 $$ 15061503 $$ 15941503 $$ 17551503 $$ 18011503 $$ 18231503 $$ 63201503 $$ 68541503 $$ 217991503 $$ 218001503 $$ 348701503 $$ 404621503 $$ 404631504 $$ 15051504 $$ 15061504 $$ 15941504 $$ 17551504 $$ 18011504 $$ 18231504 $$ 63201504 $$ 68541504 $$ 217991504 $$ 218001504 $$ 348701504 $$ 404621504 $$ 404631505 $$ 15061505 $$ 15941505 $$ 17551505 $$ 18011505 $$ 18231505 $$ 63201505 $$ 68541505 $$ 217991505 $$ 218001505 $$ 348701506 $$ 15941506 $$ 17551506 $$ 18011506 $$ 18231506 $$ 63201506 $$ 68541506 $$ 217991506 $$ 218001506 $$ 348701506 $$ 404621506 $$ 404631507 $$ 20991507 $$ 88111507 $$ 93371507 $$ 118961507 $$ 231091507 $$ 333651507 $$ 352721507 $$ 434791507 $$ 434801509 $$ 412421510 $$ 250761510 $$ 413171511 $$ 72531511 $$ 122971512 $$ 15551512 $$ 16901512 $$ 49421512 $$ 102351512 $$ 284341512 $$ 289621512 $$ 415921513 $$ 25461513 $$ 387001513 $$ 392751514 $$ 18081514 $$ 34401514 $$ 45571514 $$ 177871514 $$ 215031514 $$ 261691514 $$ 373251515 $$ 30931516 $$ 52431516 $$ 122171516 $$ 266441516 $$ 342231516 $$ 424311516 $$ 438821517 $$ 59251517 $$ 132161518 $$ 15811518 $$ 16101518 $$ 28851518 $$ 295041519 $$ 35761519 $$ 45261519 $$ 53271519 $$ 63311519 $$ 337931519 $$ 342781520 $$ 86691520 $$ 112751520 $$ 114601520 $$ 165361520 $$ 200341520 $$ 204381520 $$ 394411520 $$ 410761520 $$ 426921520 $$ 438211521 $$ 15361521 $$ 15411521 $$ 15421521 $$ 15571521 $$ 15841521 $$ 18141521 $$ 20041521 $$ 38541521 $$ 41471521 $$ 75991521 $$ 400101522 $$ 144551522 $$ 236591522 $$ 403881522 $$ 412701523 $$ 21901524 $$ 308671524 $$ 328091524 $$ 391271525 $$ 21861525 $$ 41931525 $$ 320591526 $$ 29461526 $$ 34721526 $$ 109851526 $$ 265221527 $$ 228291528 $$ 86341529 $$ 17261529 $$ 19831529 $$ 36051529 $$ 42961529 $$ 96011529 $$ 107911529 $$ 124281529 $$ 406911530 $$ 19941530 $$ 22881530 $$ 40101530 $$ 64271530 $$ 81491530 $$ 109231530 $$ 215421531 $$ 236611531 $$ 253171531 $$ 414911532 $$ 26061532 $$ 31221532 $$ 35681533 $$ 17071533 $$ 139741533 $$ 424681534 $$ 330221534 $$ 396201534 $$ 409861535 $$ 32581535 $$ 307121536 $$ 15411536 $$ 15421536 $$ 15571536 $$ 15841536 $$ 18141536 $$ 20041536 $$ 38541536 $$ 41471536 $$ 252301537 $$ 58621537 $$ 331011537 $$ 430911538 $$ 16101538 $$ 45751538 $$ 59801539 $$ 73081539 $$ 326861540 $$ 74711540 $$ 143351540 $$ 159721540 $$ 159731540 $$ 299931540 $$ 316771541 $$ 15421541 $$ 15571541 $$ 15841541 $$ 18141541 $$ 20041541 $$ 38541541 $$ 41471541 $$ 75991541 $$ 400101542 $$ 15571542 $$ 15841542 $$ 18141542 $$ 20041542 $$ 38541542 $$ 41471542 $$ 75991542 $$ 400101543 $$ 26121544 $$ 16711544 $$ 20731544 $$ 33521544 $$ 51601544 $$ 54391544 $$ 80651544 $$ 126541544 $$ 130141544 $$ 141581544 $$ 173641544 $$ 178371544 $$ 343411544 $$ 394311544 $$ 402211544 $$ 402221544 $$ 425681545 $$ 16241545 $$ 18271545 $$ 212761547 $$ 18431547 $$ 22341547 $$ 270341548 $$ 18011548 $$ 18231548 $$ 18931548 $$ 437071549 $$ 18401549 $$ 33501549 $$ 42991549 $$ 54251549 $$ 90051549 $$ 137891549 $$ 254681549 $$ 303671550 $$ 72821551 $$ 126571551 $$ 349701551 $$ 368301552 $$ 23621552 $$ 67181552 $$ 106991552 $$ 144461552 $$ 240611553 $$ 32211553 $$ 173391553 $$ 174301553 $$ 324371553 $$ 368361553 $$ 437001553 $$ 437011553 $$ 441351554 $$ 18821554 $$ 61591554 $$ 94831554 $$ 144021554 $$ 145121554 $$ 176851554 $$ 247661554 $$ 347071555 $$ 16901555 $$ 17521555 $$ 20931555 $$ 62971555 $$ 359071555 $$ 380581556 $$ 34441556 $$ 85231556 $$ 443001557 $$ 15841557 $$ 18141557 $$ 20041557 $$ 38541557 $$ 41471557 $$ 75991557 $$ 400101558 $$ 19491558 $$ 22521558 $$ 73431558 $$ 284641558 $$ 306591559 $$ 50571559 $$ 56481559 $$ 121171559 $$ 385411559 $$ 414211560 $$ 18621561 $$ 16271562 $$ 91491562 $$ 269981562 $$ 290551562 $$ 296151562 $$ 328381563 $$ 21931563 $$ 254891564 $$ 58811564 $$ 228991565 $$ 206791565 $$ 282701566 $$ 15671566 $$ 15861566 $$ 17361566 $$ 20651566 $$ 30771566 $$ 55441566 $$ 59611566 $$ 73781566 $$ 204681566 $$ 225341567 $$ 15861567 $$ 17361567 $$ 22981567 $$ 30771567 $$ 33281567 $$ 73781567 $$ 273141568 $$ 33501568 $$ 42571568 $$ 78591568 $$ 299951568 $$ 384551569 $$ 17051569 $$ 23671569 $$ 28811569 $$ 326751569 $$ 422721570 $$ 22651570 $$ 77181570 $$ 102391570 $$ 410581571 $$ 24171571 $$ 25271571 $$ 30131571 $$ 33151571 $$ 36531571 $$ 53671571 $$ 57841571 $$ 312271571 $$ 341901572 $$ 104711572 $$ 399671573 $$ 20811573 $$ 26471573 $$ 40461573 $$ 48461573 $$ 50621573 $$ 54501573 $$ 63801573 $$ 64431573 $$ 75781573 $$ 83941573 $$ 89501573 $$ 91111573 $$ 91121573 $$ 99061573 $$ 100071573 $$ 159701573 $$ 167771573 $$ 190391573 $$ 255471573 $$ 311771573 $$ 319661573 $$ 319671573 $$ 337951573 $$ 371071573 $$ 386211573 $$ 396951573 $$ 403371574 $$ 60851574 $$ 67331574 $$ 366951575 $$ 249941575 $$ 409601577 $$ 54601577 $$ 69431577 $$ 286441578 $$ 16811578 $$ 28171578 $$ 132951578 $$ 250771579 $$ 91941579 $$ 282691579 $$ 292021581 $$ 16101581 $$ 28851581 $$ 87271581 $$ 134291581 $$ 145801581 $$ 150701581 $$ 258461581 $$ 295041583 $$ 23511584 $$ 18141584 $$ 20041584 $$ 38541584 $$ 41471584 $$ 75991584 $$ 252301584 $$ 400101585 $$ 33791585 $$ 317021585 $$ 332411586 $$ 17361586 $$ 22981586 $$ 30771586 $$ 33281586 $$ 73781586 $$ 190701586 $$ 225341586 $$ 235491586 $$ 406551587 $$ 20541588 $$ 303951588 $$ 306001589 $$ 17101589 $$ 31701589 $$ 54471589 $$ 293311589 $$ 293321590 $$ 44141590 $$ 44151590 $$ 91981590 $$ 253021590 $$ 283691590 $$ 406481590 $$ 422331592 $$ 338911592 $$ 398221593 $$ 18761593 $$ 21861593 $$ 40231593 $$ 328491593 $$ 423291593 $$ 423301594 $$ 17551594 $$ 18011594 $$ 18231594 $$ 63201594 $$ 108161594 $$ 348701594 $$ 404621594 $$ 404631594 $$ 406921595 $$ 16591595 $$ 18481595 $$ 22321595 $$ 82431595 $$ 360731595 $$ 407791596 $$ 16271596 $$ 30101596 $$ 426411597 $$ 63021597 $$ 82951598 $$ 20971598 $$ 21971598 $$ 61541598 $$ 95291598 $$ 314791600 $$ 22511600 $$ 69571600 $$ 265261600 $$ 368541600 $$ 396191600 $$ 407191601 $$ 18101601 $$ 256331601 $$ 317091603 $$ 245221603 $$ 430861603 $$ 430871604 $$ 24691604 $$ 42171604 $$ 42181604 $$ 64101605 $$ 49931605 $$ 85471605 $$ 85491605 $$ 213401605 $$ 261191605 $$ 338521605 $$ 361831605 $$ 382061606 $$ 17491606 $$ 32021606 $$ 45371606 $$ 169071606 $$ 382191606 $$ 407961606 $$ 416271607 $$ 16401607 $$ 16841607 $$ 146561608 $$ 16411608 $$ 23901608 $$ 28921608 $$ 40811608 $$ 89831608 $$ 304971608 $$ 328651608 $$ 390501609 $$ 111841610 $$ 28851610 $$ 37731611 $$ 55491611 $$ 73221612 $$ 18221612 $$ 24501612 $$ 56161612 $$ 304081613 $$ 16191613 $$ 17631613 $$ 22551613 $$ 51621613 $$ 54961613 $$ 71701613 $$ 91011613 $$ 176221613 $$ 202311613 $$ 282221613 $$ 388091614 $$ 18731614 $$ 21951614 $$ 57661615 $$ 16311615 $$ 17801615 $$ 28151615 $$ 28791615 $$ 125831615 $$ 180721615 $$ 337291616 $$ 20451616 $$ 21601616 $$ 22251616 $$ 22331616 $$ 25551616 $$ 30881616 $$ 439701617 $$ 69251618 $$ 20081618 $$ 24151618 $$ 24331618 $$ 24541618 $$ 333061619 $$ 115941620 $$ 16551620 $$ 157101620 $$ 234831620 $$ 341371620 $$ 356421620 $$ 435451621 $$ 16511621 $$ 22401621 $$ 31871621 $$ 101191621 $$ 329331621 $$ 329341621 $$ 329911621 $$ 335231621 $$ 393361622 $$ 21461622 $$ 31911622 $$ 293981622 $$ 337901623 $$ 20281623 $$ 42821623 $$ 52211623 $$ 62831623 $$ 87111623 $$ 92771623 $$ 119371623 $$ 119381623 $$ 143881623 $$ 152381623 $$ 161891623 $$ 166211623 $$ 238431623 $$ 260151623 $$ 286891623 $$ 305061624 $$ 18271624 $$ 53781624 $$ 212761625 $$ 27151625 $$ 43111625 $$ 50731625 $$ 57111625 $$ 97711625 $$ 101241625 $$ 143991625 $$ 333911625 $$ 427101626 $$ 17151626 $$ 20221626 $$ 120231626 $$ 186361626 $$ 193191626 $$ 369231627 $$ 280361627 $$ 280371627 $$ 280381627 $$ 280391627 $$ 426411628 $$ 83411628 $$ 131131628 $$ 199091628 $$ 238641628 $$ 347211628 $$ 389681629 $$ 16531629 $$ 17431629 $$ 17441629 $$ 17711629 $$ 22941629 $$ 47521629 $$ 77151629 $$ 78501629 $$ 97471629 $$ 122911629 $$ 131491629 $$ 143031629 $$ 214461629 $$ 269951630 $$ 17261630 $$ 17821630 $$ 23571630 $$ 27771630 $$ 29171630 $$ 42081630 $$ 50851630 $$ 56441630 $$ 66911630 $$ 159311630 $$ 215991630 $$ 220051630 $$ 220061630 $$ 220071630 $$ 282431630 $$ 300801631 $$ 19521631 $$ 125831631 $$ 162661631 $$ 201031631 $$ 266471632 $$ 18321632 $$ 42541632 $$ 79931632 $$ 99291632 $$ 278801632 $$ 278811632 $$ 308801632 $$ 312661633 $$ 16341634 $$ 18561634 $$ 310791634 $$ 397051635 $$ 18051635 $$ 20441635 $$ 20491635 $$ 23551635 $$ 54681635 $$ 61681635 $$ 128281635 $$ 141981635 $$ 270781635 $$ 280511635 $$ 393531636 $$ 105281636 $$ 106741636 $$ 116611636 $$ 230101637 $$ 223941638 $$ 89141639 $$ 16641639 $$ 34261639 $$ 66641639 $$ 104611639 $$ 261671639 $$ 304751639 $$ 304761640 $$ 16841640 $$ 146561641 $$ 23901641 $$ 24291641 $$ 73031643 $$ 98731643 $$ 187131644 $$ 230301645 $$ 16461645 $$ 16961645 $$ 19161645 $$ 19461645 $$ 22961645 $$ 25611645 $$ 25681645 $$ 34521645 $$ 36031645 $$ 36571645 $$ 40311645 $$ 41051645 $$ 41291645 $$ 44431645 $$ 44731645 $$ 57441645 $$ 57451645 $$ 59421645 $$ 61691645 $$ 69891645 $$ 84971645 $$ 90351645 $$ 90601645 $$ 91391645 $$ 104931645 $$ 129901645 $$ 134281645 $$ 166221645 $$ 189691645 $$ 245591645 $$ 346321645 $$ 372741645 $$ 424231645 $$ 427361645 $$ 427371645 $$ 441631646 $$ 16961646 $$ 19161646 $$ 19461646 $$ 22961646 $$ 25611646 $$ 25681646 $$ 34521646 $$ 36031646 $$ 36571646 $$ 40311646 $$ 41051646 $$ 41291646 $$ 44431646 $$ 44731646 $$ 56241646 $$ 57441646 $$ 57451646 $$ 59421646 $$ 61691646 $$ 69891646 $$ 84971646 $$ 90351646 $$ 90601646 $$ 91391646 $$ 104931646 $$ 134281646 $$ 166221646 $$ 245591646 $$ 346321646 $$ 372741646 $$ 424231646 $$ 427361646 $$ 427371646 $$ 441631647 $$ 18721647 $$ 26421647 $$ 94761647 $$ 125691647 $$ 170011647 $$ 261001647 $$ 351751648 $$ 131411648 $$ 254911648 $$ 404281649 $$ 16501649 $$ 338291650 $$ 258211651 $$ 22401651 $$ 28611651 $$ 83371651 $$ 83911651 $$ 101191651 $$ 154041651 $$ 154051651 $$ 175201651 $$ 334181651 $$ 399321652 $$ 34691652 $$ 100871652 $$ 270461653 $$ 17431653 $$ 17441653 $$ 17651653 $$ 17711653 $$ 22941653 $$ 77151653 $$ 88921653 $$ 97471653 $$ 131491653 $$ 214461655 $$ 157101655 $$ 234831655 $$ 341371655 $$ 356421655 $$ 393701655 $$ 435451656 $$ 30111656 $$ 73071656 $$ 112081656 $$ 116291656 $$ 217361656 $$ 276721656 $$ 343321657 $$ 22841657 $$ 189701658 $$ 31131658 $$ 285591658 $$ 305391659 $$ 28371659 $$ 40051659 $$ 107731661 $$ 17291661 $$ 66401661 $$ 66421661 $$ 80171661 $$ 90061661 $$ 91821661 $$ 92061661 $$ 121161661 $$ 141161661 $$ 143691661 $$ 148351661 $$ 149611661 $$ 158861661 $$ 158871661 $$ 236561661 $$ 240651661 $$ 246431661 $$ 251921661 $$ 255711661 $$ 282611661 $$ 284911661 $$ 285531661 $$ 290111661 $$ 315131661 $$ 329881661 $$ 329891661 $$ 369521662 $$ 22851662 $$ 45361663 $$ 69591664 $$ 31061664 $$ 91531664 $$ 261671664 $$ 358381665 $$ 17901665 $$ 18391665 $$ 19771665 $$ 59461665 $$ 64741665 $$ 330501666 $$ 64831666 $$ 123831667 $$ 218341668 $$ 22651668 $$ 36831668 $$ 124121669 $$ 22861669 $$ 30631669 $$ 40391669 $$ 51801669 $$ 53551669 $$ 206141669 $$ 399751670 $$ 18351670 $$ 20361670 $$ 293231671 $$ 20731671 $$ 33521671 $$ 141581671 $$ 241801671 $$ 425681672 $$ 77551672 $$ 239071673 $$ 26381673 $$ 181951673 $$ 205511673 $$ 365601674 $$ 325351675 $$ 37371675 $$ 37431675 $$ 40741675 $$ 45761675 $$ 58551675 $$ 63061675 $$ 68421675 $$ 87751675 $$ 89401675 $$ 161911675 $$ 186611675 $$ 197441675 $$ 275881675 $$ 276241675 $$ 305041675 $$ 330951675 $$ 341431675 $$ 423081676 $$ 33751676 $$ 37131676 $$ 41851676 $$ 75081676 $$ 77641676 $$ 80021676 $$ 134701676 $$ 142771676 $$ 149131676 $$ 158811676 $$ 264431676 $$ 424631676 $$ 428501676 $$ 434071677 $$ 25311677 $$ 31021677 $$ 31671677 $$ 78691677 $$ 213761678 $$ 16841678 $$ 36311678 $$ 38001678 $$ 43451678 $$ 74741678 $$ 133171678 $$ 321201679 $$ 22041679 $$ 310781680 $$ 17031680 $$ 25181680 $$ 28991680 $$ 42661680 $$ 42671680 $$ 44901680 $$ 76491680 $$ 101581680 $$ 117011680 $$ 440561680 $$ 440571681 $$ 24931681 $$ 32731681 $$ 181171681 $$ 250771682 $$ 19331682 $$ 21971682 $$ 23671682 $$ 62141682 $$ 316151683 $$ 36901683 $$ 251351683 $$ 252511683 $$ 329801684 $$ 23381684 $$ 36311684 $$ 43451684 $$ 74741685 $$ 74501686 $$ 21851686 $$ 244731687 $$ 17871687 $$ 34671687 $$ 49711688 $$ 22281688 $$ 31181688 $$ 61441689 $$ 198981689 $$ 406791691 $$ 230971691 $$ 414871692 $$ 41721692 $$ 48301692 $$ 76061692 $$ 100901692 $$ 132531692 $$ 141381692 $$ 393331693 $$ 17131693 $$ 17171693 $$ 17941693 $$ 78091693 $$ 254201693 $$ 316641693 $$ 316651693 $$ 436731694 $$ 20811694 $$ 22771694 $$ 36581694 $$ 40441694 $$ 40451694 $$ 40461694 $$ 43761694 $$ 49721694 $$ 50621694 $$ 53631694 $$ 82031694 $$ 96681694 $$ 106071694 $$ 129981694 $$ 144881695 $$ 31291695 $$ 160661696 $$ 21071696 $$ 33141696 $$ 36571696 $$ 40071696 $$ 40311696 $$ 41051696 $$ 41291696 $$ 57911696 $$ 59421696 $$ 69891696 $$ 84971696 $$ 134281696 $$ 166221696 $$ 372741696 $$ 441631697 $$ 17661697 $$ 28321697 $$ 30151697 $$ 31081697 $$ 33361697 $$ 34931697 $$ 39961697 $$ 59001697 $$ 63861697 $$ 64051697 $$ 65731697 $$ 69201697 $$ 76691697 $$ 99611697 $$ 100021697 $$ 129031697 $$ 172781697 $$ 187711697 $$ 188361697 $$ 196511697 $$ 302111697 $$ 354611698 $$ 39661698 $$ 69631698 $$ 85631698 $$ 184491699 $$ 18771699 $$ 25731699 $$ 27911699 $$ 43541699 $$ 52691699 $$ 76671699 $$ 79511699 $$ 84411699 $$ 279851700 $$ 342771701 $$ 74841701 $$ 280231702 $$ 265981703 $$ 25181703 $$ 28991703 $$ 42661703 $$ 42671703 $$ 44901703 $$ 76491703 $$ 101581703 $$ 117011703 $$ 225901704 $$ 148841704 $$ 373971705 $$ 168561705 $$ 200921705 $$ 392981706 $$ 18761706 $$ 61101706 $$ 81771706 $$ 90491706 $$ 95591706 $$ 109551706 $$ 161571707 $$ 168591707 $$ 254151707 $$ 254161707 $$ 328031707 $$ 328041707 $$ 420141708 $$ 28671708 $$ 29761708 $$ 198081708 $$ 357151709 $$ 26101709 $$ 33191709 $$ 45251709 $$ 53241709 $$ 122701709 $$ 122711709 $$ 128081709 $$ 329491709 $$ 405091710 $$ 31701710 $$ 41401710 $$ 127931710 $$ 206981710 $$ 337561712 $$ 25241713 $$ 17171713 $$ 17941713 $$ 78091713 $$ 254201713 $$ 436731715 $$ 261811715 $$ 392921716 $$ 49941716 $$ 52901716 $$ 109701716 $$ 161061716 $$ 434191717 $$ 17941717 $$ 78091717 $$ 254201717 $$ 316641717 $$ 316651717 $$ 436731718 $$ 186461719 $$ 27491720 $$ 17211720 $$ 17871720 $$ 18831720 $$ 20791720 $$ 24331720 $$ 26091720 $$ 77321721 $$ 18541721 $$ 25071721 $$ 225061721 $$ 415041723 $$ 17461723 $$ 43031723 $$ 61431724 $$ 17591724 $$ 19181725 $$ 29481725 $$ 421211726 $$ 405181727 $$ 17391727 $$ 311701728 $$ 18301729 $$ 90061729 $$ 141161729 $$ 143691729 $$ 148351729 $$ 236561729 $$ 240651729 $$ 246431729 $$ 251921729 $$ 255711729 $$ 336051729 $$ 408001730 $$ 23591730 $$ 294011730 $$ 307281731 $$ 294411731 $$ 368041731 $$ 398891732 $$ 17331732 $$ 19861732 $$ 20181732 $$ 223241733 $$ 19861733 $$ 20181733 $$ 37021733 $$ 223241733 $$ 408721733 $$ 408731733 $$ 435231734 $$ 282301735 $$ 18311735 $$ 20591735 $$ 20641735 $$ 28331735 $$ 83731735 $$ 94731735 $$ 95601735 $$ 261151736 $$ 20651736 $$ 55441736 $$ 59611736 $$ 190701736 $$ 225341737 $$ 177991737 $$ 193991737 $$ 320461738 $$ 143631738 $$ 300671740 $$ 408451741 $$ 21911741 $$ 31651742 $$ 31451742 $$ 47511742 $$ 50341742 $$ 97491742 $$ 97501742 $$ 122901743 $$ 17441743 $$ 17711743 $$ 22941743 $$ 47521743 $$ 77151743 $$ 78501743 $$ 97471743 $$ 122911743 $$ 131491743 $$ 143031743 $$ 214461744 $$ 17711744 $$ 22941744 $$ 47521744 $$ 77151744 $$ 78501744 $$ 97471744 $$ 122911744 $$ 131491744 $$ 143031747 $$ 26371747 $$ 30461747 $$ 434571748 $$ 20911748 $$ 21701748 $$ 39171748 $$ 241731748 $$ 294701748 $$ 328691750 $$ 38021750 $$ 171531751 $$ 17601751 $$ 88621751 $$ 183021751 $$ 235651751 $$ 254771752 $$ 25561752 $$ 61131752 $$ 244621752 $$ 410271752 $$ 424391752 $$ 442711753 $$ 19201753 $$ 198241753 $$ 198971753 $$ 318981755 $$ 18011755 $$ 18231755 $$ 217991755 $$ 218001755 $$ 348701755 $$ 404621755 $$ 404631756 $$ 29501756 $$ 30111756 $$ 53721756 $$ 251941757 $$ 260021757 $$ 296301757 $$ 419271758 $$ 47821758 $$ 48131758 $$ 50901758 $$ 57701758 $$ 62081758 $$ 74051758 $$ 82811758 $$ 97251758 $$ 141841758 $$ 192571759 $$ 24821759 $$ 136481759 $$ 355191760 $$ 88621760 $$ 125491760 $$ 235651760 $$ 254771761 $$ 118361761 $$ 133151761 $$ 223221761 $$ 401641763 $$ 81711764 $$ 24291765 $$ 27881765 $$ 236521766 $$ 19251766 $$ 25841766 $$ 27261766 $$ 32471766 $$ 34931766 $$ 59001766 $$ 60021766 $$ 63861766 $$ 69201766 $$ 71121766 $$ 86371766 $$ 94061766 $$ 99611766 $$ 100021766 $$ 120981766 $$ 120991766 $$ 121001766 $$ 145201766 $$ 145211766 $$ 179891766 $$ 186001766 $$ 186111766 $$ 186121766 $$ 187711766 $$ 188351766 $$ 195221766 $$ 253891766 $$ 354601766 $$ 383971766 $$ 384381767 $$ 18021768 $$ 316441769 $$ 40761769 $$ 47451769 $$ 51401769 $$ 54231769 $$ 54241769 $$ 61371769 $$ 122141769 $$ 122151769 $$ 221951769 $$ 342751770 $$ 18611770 $$ 24351770 $$ 24361770 $$ 24371770 $$ 122511770 $$ 408931771 $$ 22941771 $$ 47521771 $$ 57251771 $$ 77151771 $$ 78501771 $$ 122911771 $$ 143031771 $$ 214461772 $$ 19331772 $$ 159681773 $$ 22691773 $$ 64281773 $$ 64291773 $$ 133541773 $$ 156071773 $$ 285641773 $$ 319651774 $$ 242091775 $$ 94281775 $$ 199831775 $$ 199841775 $$ 333881775 $$ 387011775 $$ 387021775 $$ 387031775 $$ 411871776 $$ 187931777 $$ 48871777 $$ 48931777 $$ 51911777 $$ 52781777 $$ 53081777 $$ 53091777 $$ 55231777 $$ 102921777 $$ 138291778 $$ 25081778 $$ 47791778 $$ 199171779 $$ 18031779 $$ 25961779 $$ 163871779 $$ 256581779 $$ 302881780 $$ 18291780 $$ 29421780 $$ 48261780 $$ 61451780 $$ 145021780 $$ 224031781 $$ 26541781 $$ 60341781 $$ 118451781 $$ 170471781 $$ 231111785 $$ 20881786 $$ 19371786 $$ 19551786 $$ 20331786 $$ 21011786 $$ 21351786 $$ 28311786 $$ 38761786 $$ 41671786 $$ 425871787 $$ 19061787 $$ 22231787 $$ 33101787 $$ 54591787 $$ 77031787 $$ 95121787 $$ 95141787 $$ 235521787 $$ 322611788 $$ 20761788 $$ 31201788 $$ 198991788 $$ 302611789 $$ 22621789 $$ 51251789 $$ 86901789 $$ 107261789 $$ 146661789 $$ 254421790 $$ 18391790 $$ 19771790 $$ 176521790 $$ 330501791 $$ 25201791 $$ 37481791 $$ 53771791 $$ 105011791 $$ 382271791 $$ 382281792 $$ 32331792 $$ 43191793 $$ 31111793 $$ 80791793 $$ 81171793 $$ 112971793 $$ 113581793 $$ 170321793 $$ 174981794 $$ 316641794 $$ 316651794 $$ 436731795 $$ 23141795 $$ 425671796 $$ 19131796 $$ 43361796 $$ 150941798 $$ 28281798 $$ 30901798 $$ 42861798 $$ 51591798 $$ 93741798 $$ 364951798 $$ 410801799 $$ 45191799 $$ 281021799 $$ 282471800 $$ 19411800 $$ 38031800 $$ 44221800 $$ 58511800 $$ 63781800 $$ 105661800 $$ 132461801 $$ 18231801 $$ 18931801 $$ 266041801 $$ 348701802 $$ 340001803 $$ 72421803 $$ 216881804 $$ 18201804 $$ 18281804 $$ 18881804 $$ 20521804 $$ 62521804 $$ 247421805 $$ 20491805 $$ 20611805 $$ 22721805 $$ 381911807 $$ 22961807 $$ 65811807 $$ 123841807 $$ 269041809 $$ 22341810 $$ 111751810 $$ 197961810 $$ 290681810 $$ 400851812 $$ 19271812 $$ 30651813 $$ 28511813 $$ 122041813 $$ 148711814 $$ 20041814 $$ 38541814 $$ 75991814 $$ 252301814 $$ 400101815 $$ 18291815 $$ 22841815 $$ 73731815 $$ 74551815 $$ 98051815 $$ 330001816 $$ 25441816 $$ 30251816 $$ 338591817 $$ 21521817 $$ 75211817 $$ 85201817 $$ 97461817 $$ 99331817 $$ 270641817 $$ 275461817 $$ 368011818 $$ 22581818 $$ 31531818 $$ 55961818 $$ 75161818 $$ 199141818 $$ 200241818 $$ 278751818 $$ 294351819 $$ 242061819 $$ 299151820 $$ 18281820 $$ 18881820 $$ 20521820 $$ 62521823 $$ 18931823 $$ 266041823 $$ 314821823 $$ 348701825 $$ 19151826 $$ 21331826 $$ 67181826 $$ 156881826 $$ 293331826 $$ 326711826 $$ 400711827 $$ 212761827 $$ 337851828 $$ 18881828 $$ 20521828 $$ 62521828 $$ 247421829 $$ 20561829 $$ 21031829 $$ 29421829 $$ 74551829 $$ 224031829 $$ 306801830 $$ 52541830 $$ 347611831 $$ 20641831 $$ 28331831 $$ 83731831 $$ 94731831 $$ 261151831 $$ 377441831 $$ 434401831 $$ 434411832 $$ 20391832 $$ 24511832 $$ 24521832 $$ 24671832 $$ 25031832 $$ 45271832 $$ 76461832 $$ 87511832 $$ 112421832 $$ 137621832 $$ 138831832 $$ 158961832 $$ 218011832 $$ 278801832 $$ 278811832 $$ 292051832 $$ 366551832 $$ 390721832 $$ 390731833 $$ 23481833 $$ 135621834 $$ 37171834 $$ 136811834 $$ 206061834 $$ 358471835 $$ 20361835 $$ 21071835 $$ 62451835 $$ 101461835 $$ 121731835 $$ 121741835 $$ 377661835 $$ 382801835 $$ 413241836 $$ 47701836 $$ 137741836 $$ 315981837 $$ 22921837 $$ 24211837 $$ 26041837 $$ 27431837 $$ 29541837 $$ 201441837 $$ 376641837 $$ 388971838 $$ 22631838 $$ 82281839 $$ 19771840 $$ 21231840 $$ 43731840 $$ 44841840 $$ 181101840 $$ 343151840 $$ 425341840 $$ 425351841 $$ 33201841 $$ 273341842 $$ 20201842 $$ 69491842 $$ 100471843 $$ 22341843 $$ 255411845 $$ 133301847 $$ 44861847 $$ 44881847 $$ 81381847 $$ 139631847 $$ 209851847 $$ 226241847 $$ 378941847 $$ 387091847 $$ 437761848 $$ 22321848 $$ 236811849 $$ 27951849 $$ 191561849 $$ 220841849 $$ 220851849 $$ 332541850 $$ 32101850 $$ 39841850 $$ 50051850 $$ 57201850 $$ 61061850 $$ 76931850 $$ 81821850 $$ 82181850 $$ 91261850 $$ 94991850 $$ 134261850 $$ 154451850 $$ 166941850 $$ 171271850 $$ 175851851 $$ 38331851 $$ 381001852 $$ 21731852 $$ 55351852 $$ 70551852 $$ 97271852 $$ 110461852 $$ 151561852 $$ 176921852 $$ 279881852 $$ 279891852 $$ 279901854 $$ 60341854 $$ 225061854 $$ 324091854 $$ 375791854 $$ 375801855 $$ 90671855 $$ 342281856 $$ 307771856 $$ 321831856 $$ 397051857 $$ 71351857 $$ 94811857 $$ 264201858 $$ 44351859 $$ 356501860 $$ 18801860 $$ 19091860 $$ 29181860 $$ 45241860 $$ 69421860 $$ 82161860 $$ 122311861 $$ 24351861 $$ 24361861 $$ 24371861 $$ 122511861 $$ 182001861 $$ 217721861 $$ 408931862 $$ 20781862 $$ 21661862 $$ 30431862 $$ 89811862 $$ 104481862 $$ 145101862 $$ 412621863 $$ 25911863 $$ 405381864 $$ 23351864 $$ 28431864 $$ 30531864 $$ 32221864 $$ 33211864 $$ 34421864 $$ 34431864 $$ 44511864 $$ 45281864 $$ 47491864 $$ 47501864 $$ 50321864 $$ 50331864 $$ 50531864 $$ 57231864 $$ 73601864 $$ 82201864 $$ 84281864 $$ 96571864 $$ 98081864 $$ 122861864 $$ 240951864 $$ 293551865 $$ 19501865 $$ 24811865 $$ 29631865 $$ 57261865 $$ 64931865 $$ 123131865 $$ 186441867 $$ 27281867 $$ 136921867 $$ 228001868 $$ 19311868 $$ 440911869 $$ 30211869 $$ 40661869 $$ 425991869 $$ 426001870 $$ 36631870 $$ 51191870 $$ 56131870 $$ 186101870 $$ 265921870 $$ 387621871 $$ 45431872 $$ 19921872 $$ 26421872 $$ 27171872 $$ 39501872 $$ 43321872 $$ 58661872 $$ 407941872 $$ 407951873 $$ 30761875 $$ 31061875 $$ 37221875 $$ 48891875 $$ 420231876 $$ 33201876 $$ 33391876 $$ 37431876 $$ 40231876 $$ 61101876 $$ 61531876 $$ 97301876 $$ 155381876 $$ 423291876 $$ 423301877 $$ 25731877 $$ 27911877 $$ 62071877 $$ 411911878 $$ 137441878 $$ 143961878 $$ 191211878 $$ 236431879 $$ 30561879 $$ 135351879 $$ 374701880 $$ 19091880 $$ 29181880 $$ 45241880 $$ 69421880 $$ 82161880 $$ 122311881 $$ 19791881 $$ 64711881 $$ 64721881 $$ 241821881 $$ 272931882 $$ 33671882 $$ 57151882 $$ 94831882 $$ 201411882 $$ 412181882 $$ 429911883 $$ 45701883 $$ 77321884 $$ 27691884 $$ 321691885 $$ 22881885 $$ 35601885 $$ 123901885 $$ 137711885 $$ 209211886 $$ 21511886 $$ 25751886 $$ 30761886 $$ 135651886 $$ 153041886 $$ 207091886 $$ 207101886 $$ 367121887 $$ 21561888 $$ 20521888 $$ 62521888 $$ 247421889 $$ 20151889 $$ 23941889 $$ 28421889 $$ 373101889 $$ 405151890 $$ 19201891 $$ 20511891 $$ 22281891 $$ 26731891 $$ 39191891 $$ 41301891 $$ 42341891 $$ 54121891 $$ 58421891 $$ 64561891 $$ 146081891 $$ 152321891 $$ 152331891 $$ 196061891 $$ 259971892 $$ 22761892 $$ 103361892 $$ 282811893 $$ 286181893 $$ 292721893 $$ 314821893 $$ 329551894 $$ 21621894 $$ 246051894 $$ 246061894 $$ 341001894 $$ 373301894 $$ 421221895 $$ 24061895 $$ 70521895 $$ 86591895 $$ 90541895 $$ 205461895 $$ 335351895 $$ 343501895 $$ 395671895 $$ 419921896 $$ 21881896 $$ 372621897 $$ 59891897 $$ 60961898 $$ 29411898 $$ 30031898 $$ 33431898 $$ 34501898 $$ 57361898 $$ 58321898 $$ 58331898 $$ 74441898 $$ 76011898 $$ 82381898 $$ 82391898 $$ 142111898 $$ 430751899 $$ 23731899 $$ 156591899 $$ 313971900 $$ 401611901 $$ 337311902 $$ 112171902 $$ 272401903 $$ 209001904 $$ 314971904 $$ 385761905 $$ 133961906 $$ 22211906 $$ 33101906 $$ 315861908 $$ 20681908 $$ 22421908 $$ 30501908 $$ 41251908 $$ 77041908 $$ 265471908 $$ 323021908 $$ 438611909 $$ 29181909 $$ 45241909 $$ 69421909 $$ 82161909 $$ 122311910 $$ 23791910 $$ 95561910 $$ 252391911 $$ 25691911 $$ 30471911 $$ 39051911 $$ 104791911 $$ 139241911 $$ 139251911 $$ 172511911 $$ 414761912 $$ 57291912 $$ 336321912 $$ 393311912 $$ 421301914 $$ 21891914 $$ 32281914 $$ 291501914 $$ 343451915 $$ 23041915 $$ 357311916 $$ 19461916 $$ 22961916 $$ 25611916 $$ 25681916 $$ 34521916 $$ 36031916 $$ 36571916 $$ 40221916 $$ 44431916 $$ 44721916 $$ 57431916 $$ 57441916 $$ 57451916 $$ 61691916 $$ 90351916 $$ 96421916 $$ 104931916 $$ 129901916 $$ 189691916 $$ 427361916 $$ 427371917 $$ 442341919 $$ 32351919 $$ 56111919 $$ 203881919 $$ 375501919 $$ 420281919 $$ 437391920 $$ 318981921 $$ 265641923 $$ 195981925 $$ 25841925 $$ 27261925 $$ 32471925 $$ 60021925 $$ 71121925 $$ 86371925 $$ 136661925 $$ 160641925 $$ 383971926 $$ 32821926 $$ 249181927 $$ 41391927 $$ 86271927 $$ 400631928 $$ 20191928 $$ 26661928 $$ 46011928 $$ 280521928 $$ 350891930 $$ 26971932 $$ 35161932 $$ 118341932 $$ 349761933 $$ 276471933 $$ 299581934 $$ 144861934 $$ 256721935 $$ 19931935 $$ 29051935 $$ 68021935 $$ 84381935 $$ 108281935 $$ 157131935 $$ 293781936 $$ 34811936 $$ 46191936 $$ 46771936 $$ 119891936 $$ 283441936 $$ 283451936 $$ 420361937 $$ 19551937 $$ 21061937 $$ 38761937 $$ 41671937 $$ 98761939 $$ 21951939 $$ 362471939 $$ 362481939 $$ 366521940 $$ 366311941 $$ 58511941 $$ 387371942 $$ 19751942 $$ 19761942 $$ 20031942 $$ 25291942 $$ 35131942 $$ 38361942 $$ 47571942 $$ 53331942 $$ 57271942 $$ 57281942 $$ 59311942 $$ 59321942 $$ 59401942 $$ 66151942 $$ 69611942 $$ 73661942 $$ 81451942 $$ 82231942 $$ 92421942 $$ 94581942 $$ 101071942 $$ 123171942 $$ 123181942 $$ 133621942 $$ 133631942 $$ 137521942 $$ 139001942 $$ 139011942 $$ 139021942 $$ 162521942 $$ 162531942 $$ 175831942 $$ 175841942 $$ 212321942 $$ 213651942 $$ 244641942 $$ 270411942 $$ 342501944 $$ 37281944 $$ 123651945 $$ 20761945 $$ 43621945 $$ 50221945 $$ 67521945 $$ 236351946 $$ 22961946 $$ 25611946 $$ 25681946 $$ 34521946 $$ 36031946 $$ 40221946 $$ 40311946 $$ 44431946 $$ 44721946 $$ 57431946 $$ 57441946 $$ 57451946 $$ 61691946 $$ 79771946 $$ 96421946 $$ 129901946 $$ 189691946 $$ 269051946 $$ 427361946 $$ 427371948 $$ 303941949 $$ 35821949 $$ 125391949 $$ 136781949 $$ 284641949 $$ 306591951 $$ 19921951 $$ 23251951 $$ 61511951 $$ 171161951 $$ 207671951 $$ 207681951 $$ 420661952 $$ 41431952 $$ 101161952 $$ 204161952 $$ 205661952 $$ 304981953 $$ 20101953 $$ 25441953 $$ 196051954 $$ 352971954 $$ 441021955 $$ 21061955 $$ 38761955 $$ 41671955 $$ 98761957 $$ 25311957 $$ 28621957 $$ 62951957 $$ 186291957 $$ 414571958 $$ 195401961 $$ 20231961 $$ 20571961 $$ 20891961 $$ 21311961 $$ 77311961 $$ 117361961 $$ 117371961 $$ 225531961 $$ 253421961 $$ 253431962 $$ 23711962 $$ 77051962 $$ 424191963 $$ 33681964 $$ 22631964 $$ 37171964 $$ 68691964 $$ 82281965 $$ 22271966 $$ 24701966 $$ 156171966 $$ 200681966 $$ 394961966 $$ 424261967 $$ 20901967 $$ 51221967 $$ 155671968 $$ 21421968 $$ 22431968 $$ 30851968 $$ 138411968 $$ 215891968 $$ 416351969 $$ 57691969 $$ 199131973 $$ 67191973 $$ 70051973 $$ 78121973 $$ 106081975 $$ 19761975 $$ 20031975 $$ 25291975 $$ 35131975 $$ 38361975 $$ 47571975 $$ 53331975 $$ 57271975 $$ 57281975 $$ 59311975 $$ 59321975 $$ 66151975 $$ 69611975 $$ 73661975 $$ 81451975 $$ 82231975 $$ 85931975 $$ 94581975 $$ 101071975 $$ 115801975 $$ 123171975 $$ 123181975 $$ 133621975 $$ 133631975 $$ 137521975 $$ 139001975 $$ 139011975 $$ 139021975 $$ 162521975 $$ 162531975 $$ 173311975 $$ 212321975 $$ 270421975 $$ 342501975 $$ 365991975 $$ 369121975 $$ 421171975 $$ 442801976 $$ 20031976 $$ 25291976 $$ 35131976 $$ 38361976 $$ 47571976 $$ 53331976 $$ 57271976 $$ 57281976 $$ 59311976 $$ 59321976 $$ 66151976 $$ 69611976 $$ 73661976 $$ 81451976 $$ 82231976 $$ 85931976 $$ 94581976 $$ 101071976 $$ 115801976 $$ 123171976 $$ 123181976 $$ 133621976 $$ 133631976 $$ 137521976 $$ 139001976 $$ 139011976 $$ 139021976 $$ 162521976 $$ 162531976 $$ 173311976 $$ 212321976 $$ 270421976 $$ 342501976 $$ 365991976 $$ 369121976 $$ 421171976 $$ 442801977 $$ 176521977 $$ 330501979 $$ 100671979 $$ 437321980 $$ 158801980 $$ 282231981 $$ 281341983 $$ 89741983 $$ 145171983 $$ 235461983 $$ 243971983 $$ 258041983 $$ 438551984 $$ 27471984 $$ 40621984 $$ 220121984 $$ 225921984 $$ 225931985 $$ 201101985 $$ 376551986 $$ 20181986 $$ 223241986 $$ 329831986 $$ 329841986 $$ 435231989 $$ 25581989 $$ 28321989 $$ 42041989 $$ 290561992 $$ 142831992 $$ 207671992 $$ 207681993 $$ 29051993 $$ 108281994 $$ 22881994 $$ 61741994 $$ 109231994 $$ 193281994 $$ 330921995 $$ 42741995 $$ 43911995 $$ 116921995 $$ 118571995 $$ 172711995 $$ 377541996 $$ 177621996 $$ 391711997 $$ 28861997 $$ 266421997 $$ 339831997 $$ 370281998 $$ 21821998 $$ 216411999 $$ 237742000 $$ 157572001 $$ 286822001 $$ 289492002 $$ 64022002 $$ 227782002 $$ 363042003 $$ 25292003 $$ 35132003 $$ 38362003 $$ 47572003 $$ 53332003 $$ 57272003 $$ 57282003 $$ 59312003 $$ 59322003 $$ 59402003 $$ 66152003 $$ 69612003 $$ 73662003 $$ 81452003 $$ 82232003 $$ 92422003 $$ 94582003 $$ 101072003 $$ 123172003 $$ 123182003 $$ 137522003 $$ 139002003 $$ 139012003 $$ 139022003 $$ 162522003 $$ 162532003 $$ 175832003 $$ 175842003 $$ 212322003 $$ 213652003 $$ 244642003 $$ 342502004 $$ 252302005 $$ 116662005 $$ 413012005 $$ 419332006 $$ 245852007 $$ 74832007 $$ 87222007 $$ 264882008 $$ 23142008 $$ 166922009 $$ 40272009 $$ 45342009 $$ 57532009 $$ 57622009 $$ 177382009 $$ 392172010 $$ 25442010 $$ 139742010 $$ 278622011 $$ 23932011 $$ 24632011 $$ 27342011 $$ 48032011 $$ 308842013 $$ 21202013 $$ 21212013 $$ 21222013 $$ 25702013 $$ 25712013 $$ 25792013 $$ 26572013 $$ 26582013 $$ 28562013 $$ 31612013 $$ 33152013 $$ 38642013 $$ 45692013 $$ 53862013 $$ 53872013 $$ 53882013 $$ 53892013 $$ 58392013 $$ 78592013 $$ 159752013 $$ 189462013 $$ 216912013 $$ 387512013 $$ 387522013 $$ 391312014 $$ 25632014 $$ 32522014 $$ 83092014 $$ 148762014 $$ 148772014 $$ 272192014 $$ 303032014 $$ 336842014 $$ 427812014 $$ 427822015 $$ 23942015 $$ 373102017 $$ 21082017 $$ 21872018 $$ 364602018 $$ 367272019 $$ 26662019 $$ 113482019 $$ 222962019 $$ 390382019 $$ 419682019 $$ 425632020 $$ 100472021 $$ 25892021 $$ 383292022 $$ 321742023 $$ 20572023 $$ 20892023 $$ 21312023 $$ 77312023 $$ 117362023 $$ 117372023 $$ 225532024 $$ 27652024 $$ 39572024 $$ 40832024 $$ 48772024 $$ 77332024 $$ 87712024 $$ 143132024 $$ 426092024 $$ 439732025 $$ 45482025 $$ 59242025 $$ 90202025 $$ 104582025 $$ 427202027 $$ 138982027 $$ 209532027 $$ 230982028 $$ 62832028 $$ 87102028 $$ 87112028 $$ 92772028 $$ 119372028 $$ 119382028 $$ 143882028 $$ 152382028 $$ 161892028 $$ 166212028 $$ 238432028 $$ 242702028 $$ 260152028 $$ 275752028 $$ 278062028 $$ 280042028 $$ 286892028 $$ 305062028 $$ 318592028 $$ 440072029 $$ 34162029 $$ 281252030 $$ 388182030 $$ 388192030 $$ 431942030 $$ 434182031 $$ 20672032 $$ 26262032 $$ 86712033 $$ 21012033 $$ 21352033 $$ 72742033 $$ 140802033 $$ 328762034 $$ 94282034 $$ 123092034 $$ 134292034 $$ 135472034 $$ 428552035 $$ 27712035 $$ 168102035 $$ 240892036 $$ 62452036 $$ 155182036 $$ 377662036 $$ 382802036 $$ 413242036 $$ 418052038 $$ 48502038 $$ 409782039 $$ 41982039 $$ 158962039 $$ 187162039 $$ 396032040 $$ 23642040 $$ 40882040 $$ 42982040 $$ 55872041 $$ 68682041 $$ 436982044 $$ 20492044 $$ 23552045 $$ 21602045 $$ 22252045 $$ 22332045 $$ 25552045 $$ 30882045 $$ 347092045 $$ 439702046 $$ 63052046 $$ 378232047 $$ 353442047 $$ 353452048 $$ 41262048 $$ 162962048 $$ 332462048 $$ 332472049 $$ 23552049 $$ 43492049 $$ 64532049 $$ 84102050 $$ 45332050 $$ 291202050 $$ 293862051 $$ 24042051 $$ 26732051 $$ 39192051 $$ 94212051 $$ 136032051 $$ 170382051 $$ 265012051 $$ 347342053 $$ 205942053 $$ 205952053 $$ 205962053 $$ 328982055 $$ 88462055 $$ 255822056 $$ 22732056 $$ 23942056 $$ 217342057 $$ 20892057 $$ 21312057 $$ 77312057 $$ 117362057 $$ 117372057 $$ 225532057 $$ 253422057 $$ 253432058 $$ 25462058 $$ 33262058 $$ 250332059 $$ 32922059 $$ 42682059 $$ 79212059 $$ 95602059 $$ 144432059 $$ 159112059 $$ 173042059 $$ 396132060 $$ 31742060 $$ 39712060 $$ 43552060 $$ 75972060 $$ 107112060 $$ 195622060 $$ 211542060 $$ 435932061 $$ 22722061 $$ 23512061 $$ 160482061 $$ 244252061 $$ 286502061 $$ 385252062 $$ 37192062 $$ 70512062 $$ 253392063 $$ 118272063 $$ 198852063 $$ 419462064 $$ 261152065 $$ 30772065 $$ 33282065 $$ 36332065 $$ 55442065 $$ 59242065 $$ 59612066 $$ 26102066 $$ 27662066 $$ 34652066 $$ 291382067 $$ 21672068 $$ 22422068 $$ 36492068 $$ 80322068 $$ 105042068 $$ 143172068 $$ 414102069 $$ 80122069 $$ 311922070 $$ 21852071 $$ 397362072 $$ 32822072 $$ 78402072 $$ 158052073 $$ 33522073 $$ 35842073 $$ 54392073 $$ 80652073 $$ 126542073 $$ 130142073 $$ 141582073 $$ 173642073 $$ 178372073 $$ 343412074 $$ 43442074 $$ 274842075 $$ 27002075 $$ 31282075 $$ 89822075 $$ 158482075 $$ 234902076 $$ 67522076 $$ 278252077 $$ 422432078 $$ 30432078 $$ 42102078 $$ 266502079 $$ 49772079 $$ 124162079 $$ 382382080 $$ 24202080 $$ 378842081 $$ 22772081 $$ 26472081 $$ 40442081 $$ 40452081 $$ 40462081 $$ 43162081 $$ 49722081 $$ 50622081 $$ 53632081 $$ 64432081 $$ 96682081 $$ 106072081 $$ 276512081 $$ 427322082 $$ 23192082 $$ 29992082 $$ 57812082 $$ 261222083 $$ 230942085 $$ 237952085 $$ 387692086 $$ 22052086 $$ 22062086 $$ 24562086 $$ 25822086 $$ 27222086 $$ 27972086 $$ 28682086 $$ 69152086 $$ 84492086 $$ 98842086 $$ 185332086 $$ 292582086 $$ 302452087 $$ 97052088 $$ 434742089 $$ 21312089 $$ 28692089 $$ 30432089 $$ 77312089 $$ 117362089 $$ 117372089 $$ 225532089 $$ 253422089 $$ 253432090 $$ 27832090 $$ 37762090 $$ 51222090 $$ 295462090 $$ 428602091 $$ 21702091 $$ 29262091 $$ 30312091 $$ 44912092 $$ 440802093 $$ 72942093 $$ 212872093 $$ 212882094 $$ 88252094 $$ 377652095 $$ 75602095 $$ 187582095 $$ 383072095 $$ 389792096 $$ 46762096 $$ 78242096 $$ 406132097 $$ 283632097 $$ 314792098 $$ 26812098 $$ 34562098 $$ 218052098 $$ 352892099 $$ 118962099 $$ 434792099 $$ 434802100 $$ 29062101 $$ 21352101 $$ 28312101 $$ 86102101 $$ 425872102 $$ 331522103 $$ 36812104 $$ 23982104 $$ 235802104 $$ 296452105 $$ 223932106 $$ 37912106 $$ 247112106 $$ 273272107 $$ 33142107 $$ 40072107 $$ 121732107 $$ 121742107 $$ 155182107 $$ 158182107 $$ 289812108 $$ 21872108 $$ 262742111 $$ 34762111 $$ 312492112 $$ 37392112 $$ 49702112 $$ 252422112 $$ 254972115 $$ 339872116 $$ 228032117 $$ 30872117 $$ 43682117 $$ 76162117 $$ 85712117 $$ 295612118 $$ 352352118 $$ 405402119 $$ 39882119 $$ 49882119 $$ 64062119 $$ 106042119 $$ 436782120 $$ 21212120 $$ 21222120 $$ 25702120 $$ 25712120 $$ 25792120 $$ 26572120 $$ 26582120 $$ 37882120 $$ 45692120 $$ 55632120 $$ 58392120 $$ 189462120 $$ 212212120 $$ 235412120 $$ 236902121 $$ 21222121 $$ 25702121 $$ 25712121 $$ 25792121 $$ 26572121 $$ 26582121 $$ 37882121 $$ 45692121 $$ 55632121 $$ 58392121 $$ 189462121 $$ 212212121 $$ 235412121 $$ 236902122 $$ 25702122 $$ 25712122 $$ 25792122 $$ 26572122 $$ 26582122 $$ 37882122 $$ 45692122 $$ 55632122 $$ 58392122 $$ 189462122 $$ 212212122 $$ 235412122 $$ 236902123 $$ 252992124 $$ 157452126 $$ 21272126 $$ 32532126 $$ 33562126 $$ 59112126 $$ 64182127 $$ 32532127 $$ 59112127 $$ 64182127 $$ 429582128 $$ 429212130 $$ 160372131 $$ 28692131 $$ 30432131 $$ 77312131 $$ 117362131 $$ 117372131 $$ 225532131 $$ 253422131 $$ 253432132 $$ 24292132 $$ 45252132 $$ 71352132 $$ 94812132 $$ 115122132 $$ 118722132 $$ 118732132 $$ 349662132 $$ 349672133 $$ 36512133 $$ 400712134 $$ 370492134 $$ 421002135 $$ 72742135 $$ 328762136 $$ 61102136 $$ 122602136 $$ 159272136 $$ 275902137 $$ 21382137 $$ 21392137 $$ 21402137 $$ 21412137 $$ 21802137 $$ 21812137 $$ 26132137 $$ 230262137 $$ 230272137 $$ 334602137 $$ 334612138 $$ 21392138 $$ 21402138 $$ 21412138 $$ 21802138 $$ 21812138 $$ 26132138 $$ 230262138 $$ 230272138 $$ 334602138 $$ 334612139 $$ 21402139 $$ 21412139 $$ 21802139 $$ 21812139 $$ 26132139 $$ 230262139 $$ 230272139 $$ 334602139 $$ 334612140 $$ 21412140 $$ 21802140 $$ 21812140 $$ 26132140 $$ 230262140 $$ 230272140 $$ 334602140 $$ 334612141 $$ 21802141 $$ 21812141 $$ 26132141 $$ 230262141 $$ 230272141 $$ 334602141 $$ 334612142 $$ 22432142 $$ 78342143 $$ 23832143 $$ 24482143 $$ 25782143 $$ 28612143 $$ 29952143 $$ 76022143 $$ 76922143 $$ 97372143 $$ 111832143 $$ 124072143 $$ 126302143 $$ 131752143 $$ 134362143 $$ 135792143 $$ 207312144 $$ 22562144 $$ 35852145 $$ 26432145 $$ 50402145 $$ 67502146 $$ 26852146 $$ 31912151 $$ 61882151 $$ 262502151 $$ 324492152 $$ 108842152 $$ 115912152 $$ 200782152 $$ 258482152 $$ 373052152 $$ 375272154 $$ 260312155 $$ 23922155 $$ 24442155 $$ 24982155 $$ 29522155 $$ 32632155 $$ 50812155 $$ 58002155 $$ 59412155 $$ 59852155 $$ 65392155 $$ 85962155 $$ 96912155 $$ 103832155 $$ 115532155 $$ 115542155 $$ 241302155 $$ 253562155 $$ 259232155 $$ 260182155 $$ 279422156 $$ 34412157 $$ 26652157 $$ 54582157 $$ 83192158 $$ 320142160 $$ 22252160 $$ 22332160 $$ 25552160 $$ 30882160 $$ 439702161 $$ 39082162 $$ 373302163 $$ 67622163 $$ 87302163 $$ 388052164 $$ 24492164 $$ 307792164 $$ 437052166 $$ 41412166 $$ 52392166 $$ 368602166 $$ 412622167 $$ 428852168 $$ 21692168 $$ 22122168 $$ 431572168 $$ 431582169 $$ 22122169 $$ 431572169 $$ 431582170 $$ 91002171 $$ 24652171 $$ 207662171 $$ 222822172 $$ 22232172 $$ 39632172 $$ 88072173 $$ 176922174 $$ 24752174 $$ 252042175 $$ 412472177 $$ 124152178 $$ 255762178 $$ 391242179 $$ 267112180 $$ 21812180 $$ 26132180 $$ 334602180 $$ 334612181 $$ 26132181 $$ 230262181 $$ 230272182 $$ 36282182 $$ 79852182 $$ 216412182 $$ 332132182 $$ 340862182 $$ 383342182 $$ 385382183 $$ 22922183 $$ 28452183 $$ 38382183 $$ 116372183 $$ 168382183 $$ 342962183 $$ 394582183 $$ 407392183 $$ 433222184 $$ 75752185 $$ 24202185 $$ 121192185 $$ 235862185 $$ 246882186 $$ 76412186 $$ 265352186 $$ 284312186 $$ 289652187 $$ 48352188 $$ 372622189 $$ 32282189 $$ 38342189 $$ 41282189 $$ 105482189 $$ 274982190 $$ 37502192 $$ 27302192 $$ 109552192 $$ 243262193 $$ 254892194 $$ 309462194 $$ 406902195 $$ 25752195 $$ 329672197 $$ 24622197 $$ 316152198 $$ 23062198 $$ 53812198 $$ 152942198 $$ 242352198 $$ 289542199 $$ 22242199 $$ 22672200 $$ 23282200 $$ 25392201 $$ 58682201 $$ 223362202 $$ 65612203 $$ 69122203 $$ 75572203 $$ 155232204 $$ 151132204 $$ 300602205 $$ 22062205 $$ 24562205 $$ 25822205 $$ 27222205 $$ 27972205 $$ 28682205 $$ 69152205 $$ 84082205 $$ 84492205 $$ 185332205 $$ 292582205 $$ 302442206 $$ 24562206 $$ 25822206 $$ 27222206 $$ 27972206 $$ 28682206 $$ 69152206 $$ 84492206 $$ 98842206 $$ 185332206 $$ 253752206 $$ 292582207 $$ 32162208 $$ 24752208 $$ 38392208 $$ 49802208 $$ 54882209 $$ 45202209 $$ 143492209 $$ 224172211 $$ 25352211 $$ 41562211 $$ 48882211 $$ 253402213 $$ 48672213 $$ 106822215 $$ 132332215 $$ 292832216 $$ 23462216 $$ 24922216 $$ 28522216 $$ 29282216 $$ 50962216 $$ 354352216 $$ 354362217 $$ 27862217 $$ 128972219 $$ 141732220 $$ 395312221 $$ 54952222 $$ 286732224 $$ 22672225 $$ 22332226 $$ 35322226 $$ 39112226 $$ 107322226 $$ 367462226 $$ 367472228 $$ 31032228 $$ 31182228 $$ 132972228 $$ 152322228 $$ 152332228 $$ 270002231 $$ 50962231 $$ 288292231 $$ 288302231 $$ 288312232 $$ 25402232 $$ 74392232 $$ 132092233 $$ 347092233 $$ 439702234 $$ 39492235 $$ 29562238 $$ 235782238 $$ 252552239 $$ 67542239 $$ 69912239 $$ 197092239 $$ 201042239 $$ 266372239 $$ 333722239 $$ 339972239 $$ 343532239 $$ 358222240 $$ 329332240 $$ 329342241 $$ 27032241 $$ 94012241 $$ 105902242 $$ 171412242 $$ 383712244 $$ 22812244 $$ 49902244 $$ 56732244 $$ 60272244 $$ 85282244 $$ 117962244 $$ 129382244 $$ 172802244 $$ 240072244 $$ 279432244 $$ 295812245 $$ 25612245 $$ 37062245 $$ 87192245 $$ 283212246 $$ 223282247 $$ 23472247 $$ 328882247 $$ 344112247 $$ 344122249 $$ 46712249 $$ 250792250 $$ 42112250 $$ 67522250 $$ 321662250 $$ 321672250 $$ 325932251 $$ 253122252 $$ 35822252 $$ 73432254 $$ 23782254 $$ 26342254 $$ 69622254 $$ 76102254 $$ 106562254 $$ 133642254 $$ 167902254 $$ 258172254 $$ 270432255 $$ 71702255 $$ 407592256 $$ 107512258 $$ 54282258 $$ 200242258 $$ 259292258 $$ 287152259 $$ 71672260 $$ 27202260 $$ 157732261 $$ 27722262 $$ 107262262 $$ 146662262 $$ 184862262 $$ 254422263 $$ 40372263 $$ 57312263 $$ 82282263 $$ 108792263 $$ 147102264 $$ 27632264 $$ 55092265 $$ 67952265 $$ 124122265 $$ 321792266 $$ 421282266 $$ 421292268 $$ 33312268 $$ 43572268 $$ 90182268 $$ 116562268 $$ 140192268 $$ 159642268 $$ 226522268 $$ 276322269 $$ 156072269 $$ 383562270 $$ 276482271 $$ 23892271 $$ 37702271 $$ 74962271 $$ 99682272 $$ 23512272 $$ 286502273 $$ 23942273 $$ 217342274 $$ 43982274 $$ 201572274 $$ 346372274 $$ 441442275 $$ 48452275 $$ 102792275 $$ 157852277 $$ 26472277 $$ 40452277 $$ 40462277 $$ 50622277 $$ 106072277 $$ 427322278 $$ 155152278 $$ 317162278 $$ 419752279 $$ 66712279 $$ 432932279 $$ 432942280 $$ 33082280 $$ 33092281 $$ 43592281 $$ 49902281 $$ 56732281 $$ 60272281 $$ 85282281 $$ 172142281 $$ 279432281 $$ 388072282 $$ 314262284 $$ 73732284 $$ 103162284 $$ 198662284 $$ 339112284 $$ 340272284 $$ 340282284 $$ 357452284 $$ 378862285 $$ 45362285 $$ 366102286 $$ 35742286 $$ 51802286 $$ 206142287 $$ 237452287 $$ 402972288 $$ 61742288 $$ 109232288 $$ 137712288 $$ 193282288 $$ 330922288 $$ 361152291 $$ 141662292 $$ 38382292 $$ 265582292 $$ 407392293 $$ 34782293 $$ 58702293 $$ 122822293 $$ 138322293 $$ 262662293 $$ 273132293 $$ 325752294 $$ 77152294 $$ 78502294 $$ 143032294 $$ 214462295 $$ 310472296 $$ 25682296 $$ 26162296 $$ 61692296 $$ 123842296 $$ 278272296 $$ 427362296 $$ 427372297 $$ 28022298 $$ 33282299 $$ 23002299 $$ 23012299 $$ 388272300 $$ 23012300 $$ 388272302 $$ 72362302 $$ 290022303 $$ 24432303 $$ 30982303 $$ 50542303 $$ 50722303 $$ 53712303 $$ 55032303 $$ 55042303 $$ 57902303 $$ 66802303 $$ 74802303 $$ 86652303 $$ 115072303 $$ 134812303 $$ 144652303 $$ 154282303 $$ 159372303 $$ 160182303 $$ 174922303 $$ 181342303 $$ 202712303 $$ 235042303 $$ 235052303 $$ 251882303 $$ 251892303 $$ 251902303 $$ 267202303 $$ 277652303 $$ 277662303 $$ 435352303 $$ 435362305 $$ 105102305 $$ 256592305 $$ 256602306 $$ 152942307 $$ 23592307 $$ 323542308 $$ 23502308 $$ 40592308 $$ 43262308 $$ 45592308 $$ 47962308 $$ 47972308 $$ 47982308 $$ 47992308 $$ 48002308 $$ 53842308 $$ 53852308 $$ 106312308 $$ 125882308 $$ 130102308 $$ 269562308 $$ 296642308 $$ 313432309 $$ 23522309 $$ 25722309 $$ 102152309 $$ 312812309 $$ 362912312 $$ 30262312 $$ 38082312 $$ 67712312 $$ 104982312 $$ 157882312 $$ 391262313 $$ 31752313 $$ 423182314 $$ 66062314 $$ 260872314 $$ 410722315 $$ 173452315 $$ 199032317 $$ 24942317 $$ 263612317 $$ 368292317 $$ 429902318 $$ 23602318 $$ 65062318 $$ 418592318 $$ 438082319 $$ 69042319 $$ 103722319 $$ 219942319 $$ 219952319 $$ 219962319 $$ 219972319 $$ 219982322 $$ 23232322 $$ 27132322 $$ 93302322 $$ 117212323 $$ 27132323 $$ 93302323 $$ 117212326 $$ 37942326 $$ 86522326 $$ 200222326 $$ 386492327 $$ 47562327 $$ 266872327 $$ 356912327 $$ 391532328 $$ 25392328 $$ 28262328 $$ 48962328 $$ 101332328 $$ 186592328 $$ 355392328 $$ 359592328 $$ 359602330 $$ 285982331 $$ 216402333 $$ 178392333 $$ 337882334 $$ 33782334 $$ 101802334 $$ 337652334 $$ 348132335 $$ 28432335 $$ 30532335 $$ 32222335 $$ 33212335 $$ 34432335 $$ 44512335 $$ 45282335 $$ 47502335 $$ 50322335 $$ 50332335 $$ 50532335 $$ 57232335 $$ 73602335 $$ 82202335 $$ 84282335 $$ 122862335 $$ 240952336 $$ 151222337 $$ 82412338 $$ 29352338 $$ 49432338 $$ 103312338 $$ 151202339 $$ 44292340 $$ 32442340 $$ 47242340 $$ 143452340 $$ 261112341 $$ 131112341 $$ 151092343 $$ 87022343 $$ 224802343 $$ 224812343 $$ 405312344 $$ 43132344 $$ 112402345 $$ 261122346 $$ 24922346 $$ 28522346 $$ 29282346 $$ 53572346 $$ 72192346 $$ 127322346 $$ 310602346 $$ 317312350 $$ 40592350 $$ 43262350 $$ 45592350 $$ 47962350 $$ 47972350 $$ 47982350 $$ 47992350 $$ 48002350 $$ 53842350 $$ 53852350 $$ 106312350 $$ 125872350 $$ 131022350 $$ 131032350 $$ 131042350 $$ 131052350 $$ 269562350 $$ 296642351 $$ 55722351 $$ 130042351 $$ 426032352 $$ 25722352 $$ 74242352 $$ 102152352 $$ 312812352 $$ 399682353 $$ 137052353 $$ 202342353 $$ 213882353 $$ 436022354 $$ 27032354 $$ 32542354 $$ 85582354 $$ 132522354 $$ 141172354 $$ 262142354 $$ 431562355 $$ 54682355 $$ 107202355 $$ 145672356 $$ 296792356 $$ 349132357 $$ 29172357 $$ 50852357 $$ 56442357 $$ 282432358 $$ 434992359 $$ 155502359 $$ 332732359 $$ 413752360 $$ 65062360 $$ 418132360 $$ 438082361 $$ 33962361 $$ 247532362 $$ 41562363 $$ 329852364 $$ 324792365 $$ 432492366 $$ 229412366 $$ 328232366 $$ 428612367 $$ 125652367 $$ 366392368 $$ 238952368 $$ 247672368 $$ 250212368 $$ 391052370 $$ 40242370 $$ 42622370 $$ 73382370 $$ 349732370 $$ 378372371 $$ 35762371 $$ 57212371 $$ 89592373 $$ 34252373 $$ 73202374 $$ 242742374 $$ 307462376 $$ 377292378 $$ 26342378 $$ 167902379 $$ 53262379 $$ 59882379 $$ 95562380 $$ 24392380 $$ 24782380 $$ 25522380 $$ 28142380 $$ 30532380 $$ 32222380 $$ 48802380 $$ 51942380 $$ 57242380 $$ 98362380 $$ 116062380 $$ 143162380 $$ 347362381 $$ 374442382 $$ 340592383 $$ 24482383 $$ 25782383 $$ 28612383 $$ 76022383 $$ 140932383 $$ 207972384 $$ 29222384 $$ 50472384 $$ 110222384 $$ 276342384 $$ 388042384 $$ 439692385 $$ 74892385 $$ 340842385 $$ 372362387 $$ 209102387 $$ 216232388 $$ 29072389 $$ 99682392 $$ 24442392 $$ 29522392 $$ 58002392 $$ 65392392 $$ 260182393 $$ 24632393 $$ 27342393 $$ 308842394 $$ 217342394 $$ 373102395 $$ 45352395 $$ 53242395 $$ 162052395 $$ 304612395 $$ 370922395 $$ 370932395 $$ 370942396 $$ 50982398 $$ 158842399 $$ 29252399 $$ 34652399 $$ 34722399 $$ 65242399 $$ 67462399 $$ 82522399 $$ 97232399 $$ 295962399 $$ 415832399 $$ 415842401 $$ 302222401 $$ 368262402 $$ 30222402 $$ 40922402 $$ 40932402 $$ 86382402 $$ 167582402 $$ 407972403 $$ 315522404 $$ 26732404 $$ 41302404 $$ 94212404 $$ 136032404 $$ 170382404 $$ 205792404 $$ 265012404 $$ 347342405 $$ 26222406 $$ 70522406 $$ 86592406 $$ 90542406 $$ 334572406 $$ 395672407 $$ 24662407 $$ 66592407 $$ 414182408 $$ 29402408 $$ 32052408 $$ 49742408 $$ 305832409 $$ 204752409 $$ 329472409 $$ 442522411 $$ 236412414 $$ 256512416 $$ 51382416 $$ 76152416 $$ 128512417 $$ 33152417 $$ 36532417 $$ 53672417 $$ 57842419 $$ 94752419 $$ 267732420 $$ 42162420 $$ 121192421 $$ 26042421 $$ 27432421 $$ 200472421 $$ 329512422 $$ 24232422 $$ 24242422 $$ 24252422 $$ 25382422 $$ 37232422 $$ 37852422 $$ 42842422 $$ 50082422 $$ 74782422 $$ 97802422 $$ 119562422 $$ 182072423 $$ 24242423 $$ 24252423 $$ 25382423 $$ 37232423 $$ 37852423 $$ 42842423 $$ 50082423 $$ 74782423 $$ 97802423 $$ 119562423 $$ 182072424 $$ 24252424 $$ 25382424 $$ 37232424 $$ 37852424 $$ 42842424 $$ 50082424 $$ 74782424 $$ 97802424 $$ 119562424 $$ 182072425 $$ 25382425 $$ 37232425 $$ 37852425 $$ 42842425 $$ 50082425 $$ 97802425 $$ 119562426 $$ 31272426 $$ 119822426 $$ 132712426 $$ 252742427 $$ 66552427 $$ 80032427 $$ 286412427 $$ 393252428 $$ 132552429 $$ 324722430 $$ 224472430 $$ 354492431 $$ 27462431 $$ 93202431 $$ 437492432 $$ 108982433 $$ 26092434 $$ 34152434 $$ 77022435 $$ 24362435 $$ 24372435 $$ 122512435 $$ 182002436 $$ 24372436 $$ 122512437 $$ 122512439 $$ 24782439 $$ 25522439 $$ 28142439 $$ 30532439 $$ 32222439 $$ 48802439 $$ 51942439 $$ 98362439 $$ 143162439 $$ 292312439 $$ 347362440 $$ 58582440 $$ 71862440 $$ 113692440 $$ 172012440 $$ 385072440 $$ 390072440 $$ 390082440 $$ 398442441 $$ 92662441 $$ 366422441 $$ 429252442 $$ 365612443 $$ 30982443 $$ 50542443 $$ 50722443 $$ 53712443 $$ 55032443 $$ 55042443 $$ 57902443 $$ 66802443 $$ 74802443 $$ 86652443 $$ 108132443 $$ 110032443 $$ 115072443 $$ 154282443 $$ 159372443 $$ 160182443 $$ 174922443 $$ 189872443 $$ 189882443 $$ 202712443 $$ 277652443 $$ 277662443 $$ 319952443 $$ 319962443 $$ 338272443 $$ 420552443 $$ 435352443 $$ 435362443 $$ 442082443 $$ 442092444 $$ 29522444 $$ 58002444 $$ 65392444 $$ 260182445 $$ 104562445 $$ 141812445 $$ 244482445 $$ 270442446 $$ 24602446 $$ 25862446 $$ 141042447 $$ 32652447 $$ 236762448 $$ 25782448 $$ 28612448 $$ 76022449 $$ 30042449 $$ 55982449 $$ 76062451 $$ 24522451 $$ 24672451 $$ 25032451 $$ 45272451 $$ 56682451 $$ 64312451 $$ 90372451 $$ 91182451 $$ 152292451 $$ 158622452 $$ 24672452 $$ 25032452 $$ 45272452 $$ 56682452 $$ 64312452 $$ 90372452 $$ 91182452 $$ 152292452 $$ 158622453 $$ 199112453 $$ 200152453 $$ 207692453 $$ 352982456 $$ 25822456 $$ 27222456 $$ 27972456 $$ 28682456 $$ 84492456 $$ 192892456 $$ 302432457 $$ 51332457 $$ 59972457 $$ 77612457 $$ 221692457 $$ 221702459 $$ 49372459 $$ 106972459 $$ 155432460 $$ 25862460 $$ 41242460 $$ 141042461 $$ 37252461 $$ 172862461 $$ 316212461 $$ 329772462 $$ 28382462 $$ 386672463 $$ 48032464 $$ 27252464 $$ 135212464 $$ 206072464 $$ 334242464 $$ 438242466 $$ 66592467 $$ 25032467 $$ 45272467 $$ 56682467 $$ 90372467 $$ 91182467 $$ 158622467 $$ 416492469 $$ 400142470 $$ 126212470 $$ 200682470 $$ 424262471 $$ 30062471 $$ 361732473 $$ 29152473 $$ 121622474 $$ 394812475 $$ 29032475 $$ 40572475 $$ 54882475 $$ 271152475 $$ 295522475 $$ 312192475 $$ 312202475 $$ 375232476 $$ 29722476 $$ 60862476 $$ 432312478 $$ 25522478 $$ 28142478 $$ 30532478 $$ 48802478 $$ 51942478 $$ 292312478 $$ 332322478 $$ 332332478 $$ 347362479 $$ 209252480 $$ 151182480 $$ 248882480 $$ 316162481 $$ 57262481 $$ 123132481 $$ 403552482 $$ 403892482 $$ 419842483 $$ 135942484 $$ 24852484 $$ 40602484 $$ 48052484 $$ 67672484 $$ 85052484 $$ 109742484 $$ 112142484 $$ 124172484 $$ 133472484 $$ 184592484 $$ 306682484 $$ 416952485 $$ 85052485 $$ 159522485 $$ 183482485 $$ 306682486 $$ 24972486 $$ 25872486 $$ 39592486 $$ 49542486 $$ 54772486 $$ 90002486 $$ 90702486 $$ 101202486 $$ 106962486 $$ 138612486 $$ 160942486 $$ 172692486 $$ 323812486 $$ 323822486 $$ 323832486 $$ 398092487 $$ 335142488 $$ 41322488 $$ 137722488 $$ 139142488 $$ 265822488 $$ 341762489 $$ 289582492 $$ 28522492 $$ 29282492 $$ 53572492 $$ 72192492 $$ 317312493 $$ 181172495 $$ 27862496 $$ 178232496 $$ 250032497 $$ 25872497 $$ 32482497 $$ 43222497 $$ 54772497 $$ 70302497 $$ 80912497 $$ 106962497 $$ 154292497 $$ 338282497 $$ 368102497 $$ 376182498 $$ 29522498 $$ 31952498 $$ 32632498 $$ 36482498 $$ 43972498 $$ 97872498 $$ 103832498 $$ 157212499 $$ 36612499 $$ 86032499 $$ 172522499 $$ 315452499 $$ 435652500 $$ 95732503 $$ 45272503 $$ 56682503 $$ 64312503 $$ 90372503 $$ 91182503 $$ 152292503 $$ 158622504 $$ 42132507 $$ 245572507 $$ 380422507 $$ 389232507 $$ 421132508 $$ 47792509 $$ 29452510 $$ 263852510 $$ 334582510 $$ 418542511 $$ 26322511 $$ 372142511 $$ 392492513 $$ 186272513 $$ 368442514 $$ 115792514 $$ 323972514 $$ 363122514 $$ 363132517 $$ 199922517 $$ 202972517 $$ 337862517 $$ 442412517 $$ 442422518 $$ 28992518 $$ 42662518 $$ 42672518 $$ 44902518 $$ 76492518 $$ 94552518 $$ 101582518 $$ 117012518 $$ 138442519 $$ 31652519 $$ 376992519 $$ 379882520 $$ 27092520 $$ 359732523 $$ 418292524 $$ 34792524 $$ 200132524 $$ 216142524 $$ 224712525 $$ 140212525 $$ 226772525 $$ 266032525 $$ 395012525 $$ 441582526 $$ 32012526 $$ 52822526 $$ 227682527 $$ 30132527 $$ 341902528 $$ 29712529 $$ 35132529 $$ 38362529 $$ 47572529 $$ 53332529 $$ 57282529 $$ 66152529 $$ 73662529 $$ 82232529 $$ 85932529 $$ 115802529 $$ 139002529 $$ 139012529 $$ 139022529 $$ 162522529 $$ 162532529 $$ 342502529 $$ 365992529 $$ 369122529 $$ 421172529 $$ 442802530 $$ 67842530 $$ 73722530 $$ 87832530 $$ 125562530 $$ 146582530 $$ 293352531 $$ 31022531 $$ 206632533 $$ 136952533 $$ 284442534 $$ 343402535 $$ 26892535 $$ 32022535 $$ 41562535 $$ 48882535 $$ 70322536 $$ 382452537 $$ 216082537 $$ 225392538 $$ 37232538 $$ 37852538 $$ 42842538 $$ 50082538 $$ 97802538 $$ 119562538 $$ 182062539 $$ 28262539 $$ 48962539 $$ 186592539 $$ 355392540 $$ 132092543 $$ 353662543 $$ 358882544 $$ 41152545 $$ 381052546 $$ 37352546 $$ 110362546 $$ 331032547 $$ 391032548 $$ 266132548 $$ 329462548 $$ 346302548 $$ 376602549 $$ 212692550 $$ 113342550 $$ 392582550 $$ 433112552 $$ 28142552 $$ 30532552 $$ 32222552 $$ 48802552 $$ 51942552 $$ 98362552 $$ 116062552 $$ 143162552 $$ 347362554 $$ 144732555 $$ 30462555 $$ 30882555 $$ 32922555 $$ 38932555 $$ 50362555 $$ 170172555 $$ 289592555 $$ 441572556 $$ 61132556 $$ 61382556 $$ 424392558 $$ 39262558 $$ 42042558 $$ 67502558 $$ 109062558 $$ 123502558 $$ 131232560 $$ 160932560 $$ 359952561 $$ 25682561 $$ 34522561 $$ 57442561 $$ 57452561 $$ 96422563 $$ 53432563 $$ 75122563 $$ 171262563 $$ 428182564 $$ 57122564 $$ 63402564 $$ 143922564 $$ 216582565 $$ 269872565 $$ 282342565 $$ 296172565 $$ 307372568 $$ 34522568 $$ 36032568 $$ 44432568 $$ 57442568 $$ 57452568 $$ 61692568 $$ 96422568 $$ 129902568 $$ 189692568 $$ 269052568 $$ 427362568 $$ 427372569 $$ 30472569 $$ 39052569 $$ 104792569 $$ 139242569 $$ 139252570 $$ 25712570 $$ 25792570 $$ 26572570 $$ 26582570 $$ 28562570 $$ 37882570 $$ 45692570 $$ 58392570 $$ 189462570 $$ 236902571 $$ 25792571 $$ 26572571 $$ 26582571 $$ 37882571 $$ 45692571 $$ 58392571 $$ 189462571 $$ 235402571 $$ 236902572 $$ 102152572 $$ 312812572 $$ 362912573 $$ 27912573 $$ 52692573 $$ 76672573 $$ 79512573 $$ 279852574 $$ 26552574 $$ 48152575 $$ 30762575 $$ 378962576 $$ 25772576 $$ 26562576 $$ 32412576 $$ 33442576 $$ 34442576 $$ 51032576 $$ 54072576 $$ 243922577 $$ 26562577 $$ 32412577 $$ 33442577 $$ 34442577 $$ 51032577 $$ 54072578 $$ 28612578 $$ 76022579 $$ 26572579 $$ 26582579 $$ 33152579 $$ 38642579 $$ 45692579 $$ 58392579 $$ 78592579 $$ 159752579 $$ 189462579 $$ 216912579 $$ 276592581 $$ 218182581 $$ 396832582 $$ 27222582 $$ 27972582 $$ 28682582 $$ 69152582 $$ 84492582 $$ 185332582 $$ 292582582 $$ 387962582 $$ 387972583 $$ 28222583 $$ 29532584 $$ 27262584 $$ 32472584 $$ 64342584 $$ 71122584 $$ 84482584 $$ 86372584 $$ 192282584 $$ 383972586 $$ 141042587 $$ 28482587 $$ 39592587 $$ 49542587 $$ 54772587 $$ 56462587 $$ 67602587 $$ 73862587 $$ 79912587 $$ 88302587 $$ 90002587 $$ 90702587 $$ 91742587 $$ 101202587 $$ 106962587 $$ 160942587 $$ 286002587 $$ 323812587 $$ 323822587 $$ 323832588 $$ 424672589 $$ 357402589 $$ 383292589 $$ 396472589 $$ 398882589 $$ 405792590 $$ 347842590 $$ 347852591 $$ 405382592 $$ 424222592 $$ 429172595 $$ 154992595 $$ 166242596 $$ 34132597 $$ 46002597 $$ 128512598 $$ 28992598 $$ 30932598 $$ 42652598 $$ 257462599 $$ 75482599 $$ 279582600 $$ 63132602 $$ 27572602 $$ 67332603 $$ 280612604 $$ 27432604 $$ 329512605 $$ 38302606 $$ 385952610 $$ 34652610 $$ 57152610 $$ 291322610 $$ 318562611 $$ 35912611 $$ 40122611 $$ 92382611 $$ 101252611 $$ 151532611 $$ 213742611 $$ 353562611 $$ 373092611 $$ 408822611 $$ 412122611 $$ 427552612 $$ 71662612 $$ 132832613 $$ 209462613 $$ 381892616 $$ 35922616 $$ 103542616 $$ 208842616 $$ 212222617 $$ 98912619 $$ 26202619 $$ 26212620 $$ 26212622 $$ 97582622 $$ 260692622 $$ 307252622 $$ 307262623 $$ 195072626 $$ 86712626 $$ 330492629 $$ 199402629 $$ 264772630 $$ 33642630 $$ 59342630 $$ 139162632 $$ 386702633 $$ 28262633 $$ 33202633 $$ 35762633 $$ 50302633 $$ 57212633 $$ 146172634 $$ 122592634 $$ 167902634 $$ 316102635 $$ 145182638 $$ 93732638 $$ 162262638 $$ 205512639 $$ 28172640 $$ 26412642 $$ 204352642 $$ 410642646 $$ 200732646 $$ 329522646 $$ 399942647 $$ 41572647 $$ 50622647 $$ 64432647 $$ 427322648 $$ 397652650 $$ 37772650 $$ 58042650 $$ 62492650 $$ 85982652 $$ 249442653 $$ 40332653 $$ 262472654 $$ 335992655 $$ 48152655 $$ 137302655 $$ 222662656 $$ 32412656 $$ 33442656 $$ 34442656 $$ 51032656 $$ 54072657 $$ 26582657 $$ 33152657 $$ 37882657 $$ 38642657 $$ 45692657 $$ 58392657 $$ 78592657 $$ 189462657 $$ 236902657 $$ 276592658 $$ 33152658 $$ 37882658 $$ 38642658 $$ 45692658 $$ 58392658 $$ 78592658 $$ 189462658 $$ 236902658 $$ 276592659 $$ 87142659 $$ 211152661 $$ 65412661 $$ 85102661 $$ 329582662 $$ 35322663 $$ 33992663 $$ 133612663 $$ 235332663 $$ 425942664 $$ 198342664 $$ 382982665 $$ 32522665 $$ 54582665 $$ 161182666 $$ 46012666 $$ 88082666 $$ 140342666 $$ 280522668 $$ 28712672 $$ 223152673 $$ 64562673 $$ 265012677 $$ 42932677 $$ 43832679 $$ 27122679 $$ 340982680 $$ 400962681 $$ 352882682 $$ 54942684 $$ 167612688 $$ 286432688 $$ 310682689 $$ 32022691 $$ 253442692 $$ 340462693 $$ 27742693 $$ 57212693 $$ 179552693 $$ 201492693 $$ 313292693 $$ 313302696 $$ 33952696 $$ 425012697 $$ 377452697 $$ 382552697 $$ 419142700 $$ 158482703 $$ 325782703 $$ 401062703 $$ 424802704 $$ 162892704 $$ 412562704 $$ 428572705 $$ 30432705 $$ 176242705 $$ 416072706 $$ 27582706 $$ 27792706 $$ 28882707 $$ 132902707 $$ 353132709 $$ 359732710 $$ 34582710 $$ 50742712 $$ 340982712 $$ 411542714 $$ 169702716 $$ 97692720 $$ 285662721 $$ 348912721 $$ 367192722 $$ 27972722 $$ 28682722 $$ 84492722 $$ 385662723 $$ 202222723 $$ 436352724 $$ 130462725 $$ 78602725 $$ 438242726 $$ 32472726 $$ 34932726 $$ 71122726 $$ 94062726 $$ 383972726 $$ 384382726 $$ 384822727 $$ 110642727 $$ 112202727 $$ 129142727 $$ 344732729 $$ 61422729 $$ 99522729 $$ 141872729 $$ 190262729 $$ 190272729 $$ 190282729 $$ 190292729 $$ 190302729 $$ 190312729 $$ 195032730 $$ 394342730 $$ 402272731 $$ 435542733 $$ 35502733 $$ 54922733 $$ 349802736 $$ 56752736 $$ 406382739 $$ 35922740 $$ 36292740 $$ 81732740 $$ 335702741 $$ 28812741 $$ 51782741 $$ 70102741 $$ 80432741 $$ 163672741 $$ 204602741 $$ 314942741 $$ 314952741 $$ 314962741 $$ 350642741 $$ 350652741 $$ 401862741 $$ 413982741 $$ 434812743 $$ 329512746 $$ 31012746 $$ 366822747 $$ 220122747 $$ 225922747 $$ 225932748 $$ 368762749 $$ 31622749 $$ 33412749 $$ 244222750 $$ 79322753 $$ 315242756 $$ 164832756 $$ 418772758 $$ 27792758 $$ 28882759 $$ 31482759 $$ 34302759 $$ 48102759 $$ 198002759 $$ 207012761 $$ 46382761 $$ 201902761 $$ 201912762 $$ 31242763 $$ 77582765 $$ 77332765 $$ 425952766 $$ 317852766 $$ 324362767 $$ 77692768 $$ 281712770 $$ 441672771 $$ 29572773 $$ 61032773 $$ 67452774 $$ 42622774 $$ 332362776 $$ 35292776 $$ 139462776 $$ 187212777 $$ 220052777 $$ 220062777 $$ 220072778 $$ 329662779 $$ 28882781 $$ 29892782 $$ 171542783 $$ 37762784 $$ 62042788 $$ 53072789 $$ 31632789 $$ 63872789 $$ 79022789 $$ 111342789 $$ 111722789 $$ 111732789 $$ 231262789 $$ 286012789 $$ 320232789 $$ 339252791 $$ 52692791 $$ 79512791 $$ 84412791 $$ 279852794 $$ 270502795 $$ 57382795 $$ 342052795 $$ 432302797 $$ 28682797 $$ 292582798 $$ 36392798 $$ 54422798 $$ 99792798 $$ 140612798 $$ 189022798 $$ 189032804 $$ 197222804 $$ 387882805 $$ 376752808 $$ 48492808 $$ 371742812 $$ 357042814 $$ 48802814 $$ 51942814 $$ 116062814 $$ 347362815 $$ 337292815 $$ 356992815 $$ 357002816 $$ 439712817 $$ 400312818 $$ 28872818 $$ 316842819 $$ 236622820 $$ 36862822 $$ 29532822 $$ 262952822 $$ 361182823 $$ 45962823 $$ 82912823 $$ 128962824 $$ 73072824 $$ 81522824 $$ 255162824 $$ 260842826 $$ 68472827 $$ 430822827 $$ 430832828 $$ 30902828 $$ 51592828 $$ 287532828 $$ 287542829 $$ 46212829 $$ 48732829 $$ 343662829 $$ 394102829 $$ 406262831 $$ 314982832 $$ 31082832 $$ 39962832 $$ 60882832 $$ 61632832 $$ 67632832 $$ 120962832 $$ 129032832 $$ 163502832 $$ 233292832 $$ 233782832 $$ 302112832 $$ 393972832 $$ 393982832 $$ 411392832 $$ 417712833 $$ 83732833 $$ 114272833 $$ 333242833 $$ 340412833 $$ 377442834 $$ 94122834 $$ 367442836 $$ 174022837 $$ 112902837 $$ 217112837 $$ 224672837 $$ 341692838 $$ 47612838 $$ 48182841 $$ 78552843 $$ 30532843 $$ 32222843 $$ 33212843 $$ 34432843 $$ 45282843 $$ 47502843 $$ 50532843 $$ 73602843 $$ 122862843 $$ 240952848 $$ 56462848 $$ 74132848 $$ 74852848 $$ 79912848 $$ 88302848 $$ 91742848 $$ 96112848 $$ 96122848 $$ 157042848 $$ 181272848 $$ 254812849 $$ 172762849 $$ 296782850 $$ 29922850 $$ 35942850 $$ 47562850 $$ 341212851 $$ 291962852 $$ 29282852 $$ 53572853 $$ 118352856 $$ 31612856 $$ 37782856 $$ 53862856 $$ 53872856 $$ 53882856 $$ 53892856 $$ 387512856 $$ 387522856 $$ 391312856 $$ 407892856 $$ 425932857 $$ 54452857 $$ 104142858 $$ 29372858 $$ 31042858 $$ 37822858 $$ 74302858 $$ 83202858 $$ 97162860 $$ 42212860 $$ 296082860 $$ 296092860 $$ 307482861 $$ 83372861 $$ 83912861 $$ 154042861 $$ 154052862 $$ 38402862 $$ 62952862 $$ 101502863 $$ 101692863 $$ 290992863 $$ 300592864 $$ 31912866 $$ 38132868 $$ 84492868 $$ 302432869 $$ 30432869 $$ 85552874 $$ 368722874 $$ 384242876 $$ 116262876 $$ 441152877 $$ 419442878 $$ 341202881 $$ 51782881 $$ 80432881 $$ 204602881 $$ 413982883 $$ 115332883 $$ 371022883 $$ 371032885 $$ 295042886 $$ 266422891 $$ 59962891 $$ 306812891 $$ 306822892 $$ 429072893 $$ 363682893 $$ 378662895 $$ 28962895 $$ 28972895 $$ 48702895 $$ 98172895 $$ 201112896 $$ 28972896 $$ 48702896 $$ 98172896 $$ 201112896 $$ 369412896 $$ 369782897 $$ 48702897 $$ 98172897 $$ 201112897 $$ 369412897 $$ 369782898 $$ 29332898 $$ 29342898 $$ 30012898 $$ 136442898 $$ 276382899 $$ 42662899 $$ 42672899 $$ 44902899 $$ 76492899 $$ 117012899 $$ 257462899 $$ 340222900 $$ 32942900 $$ 284432901 $$ 53232901 $$ 68502901 $$ 146002901 $$ 397702901 $$ 404762902 $$ 47272902 $$ 77242903 $$ 397592904 $$ 32062904 $$ 148932905 $$ 40532905 $$ 68032906 $$ 119012907 $$ 30102909 $$ 31362909 $$ 41582909 $$ 241312910 $$ 29542911 $$ 29822911 $$ 56042911 $$ 366272911 $$ 366282912 $$ 34312912 $$ 35802912 $$ 35812912 $$ 53042913 $$ 36502913 $$ 258152913 $$ 432502914 $$ 121402914 $$ 381062915 $$ 33932915 $$ 121622917 $$ 50852917 $$ 56442917 $$ 159312917 $$ 174312917 $$ 300802922 $$ 43082922 $$ 410512923 $$ 48452923 $$ 57532923 $$ 57622923 $$ 124622923 $$ 177402925 $$ 182012925 $$ 365912925 $$ 415552926 $$ 30312926 $$ 32032926 $$ 51522926 $$ 56722926 $$ 65432926 $$ 69032926 $$ 85082926 $$ 104392926 $$ 117892926 $$ 223622926 $$ 347632927 $$ 45022928 $$ 72192928 $$ 310602928 $$ 317312929 $$ 301142930 $$ 300182931 $$ 250882933 $$ 29342933 $$ 30012934 $$ 30012934 $$ 136442934 $$ 276382935 $$ 49432935 $$ 151202936 $$ 29462936 $$ 60332936 $$ 125922937 $$ 37822937 $$ 83202937 $$ 97162938 $$ 37072938 $$ 130532940 $$ 32052940 $$ 49742940 $$ 291152940 $$ 305832941 $$ 30032941 $$ 202902941 $$ 430752942 $$ 224032943 $$ 68522943 $$ 193942944 $$ 40052944 $$ 386152944 $$ 441702945 $$ 255262946 $$ 225952948 $$ 64042948 $$ 103202948 $$ 131802948 $$ 225812949 $$ 31152949 $$ 162062950 $$ 30112950 $$ 251942950 $$ 253102951 $$ 31982951 $$ 166972951 $$ 172652951 $$ 290622951 $$ 384072951 $$ 384082951 $$ 385292951 $$ 398752951 $$ 398762951 $$ 398772952 $$ 31952952 $$ 58002952 $$ 65392952 $$ 97872952 $$ 131072952 $$ 131082952 $$ 260182952 $$ 350422952 $$ 350432952 $$ 350442953 $$ 361182954 $$ 53242955 $$ 379902956 $$ 34642956 $$ 54752956 $$ 64132956 $$ 433022959 $$ 352732959 $$ 388422960 $$ 32152961 $$ 54982961 $$ 267922961 $$ 296102963 $$ 392562964 $$ 338762969 $$ 268992969 $$ 270692970 $$ 31462970 $$ 34172970 $$ 34822970 $$ 35342970 $$ 74252970 $$ 350032972 $$ 54382972 $$ 223332972 $$ 272752974 $$ 29752974 $$ 52012974 $$ 102892974 $$ 250932975 $$ 52012975 $$ 102892976 $$ 32942976 $$ 69302977 $$ 123852977 $$ 404802978 $$ 439882982 $$ 56042984 $$ 64672984 $$ 154212985 $$ 34872985 $$ 43372985 $$ 45832985 $$ 48472985 $$ 50172985 $$ 50182985 $$ 60632985 $$ 81162985 $$ 121322985 $$ 121332985 $$ 173822986 $$ 162752992 $$ 61552994 $$ 32362994 $$ 34612994 $$ 103122994 $$ 125912994 $$ 136472995 $$ 29962995 $$ 30552995 $$ 34492995 $$ 61932995 $$ 76922995 $$ 82322995 $$ 95812995 $$ 101732995 $$ 162562995 $$ 266782995 $$ 402032996 $$ 30552996 $$ 34492996 $$ 35982996 $$ 35992996 $$ 53412996 $$ 82322996 $$ 124082996 $$ 134112996 $$ 134782996 $$ 402032997 $$ 325412998 $$ 38052998 $$ 128492998 $$ 394042998 $$ 394052998 $$ 409512999 $$ 57812999 $$ 312593000 $$ 53193000 $$ 58403003 $$ 430753004 $$ 93803006 $$ 50663006 $$ 132063006 $$ 361733007 $$ 30083007 $$ 34883007 $$ 98323007 $$ 172403008 $$ 43523008 $$ 43693010 $$ 210383011 $$ 251943011 $$ 343323012 $$ 115793013 $$ 89693013 $$ 92243013 $$ 206313013 $$ 341903015 $$ 196513016 $$ 381283016 $$ 392813016 $$ 394593016 $$ 433243016 $$ 433253018 $$ 267153018 $$ 329793018 $$ 430253019 $$ 60773021 $$ 400933021 $$ 412023024 $$ 413623025 $$ 338593027 $$ 316543028 $$ 101213029 $$ 30303029 $$ 31093029 $$ 179653029 $$ 195373029 $$ 413773030 $$ 31093030 $$ 179653030 $$ 195373030 $$ 413773032 $$ 30333032 $$ 34903032 $$ 178443033 $$ 34903034 $$ 139103034 $$ 442333036 $$ 37593037 $$ 403983037 $$ 429463037 $$ 429473041 $$ 381333042 $$ 38243043 $$ 53253044 $$ 238283045 $$ 419733047 $$ 39053051 $$ 31413051 $$ 406223052 $$ 404583052 $$ 420773052 $$ 429383053 $$ 34433053 $$ 45283053 $$ 48803053 $$ 57243053 $$ 73603053 $$ 75313053 $$ 96603053 $$ 102653053 $$ 122863053 $$ 146343053 $$ 223023053 $$ 318693055 $$ 34493055 $$ 61933055 $$ 76923055 $$ 82323055 $$ 95813055 $$ 101733055 $$ 162563055 $$ 266783055 $$ 402033057 $$ 97943057 $$ 253923057 $$ 337613059 $$ 31033059 $$ 47543059 $$ 148153059 $$ 244633059 $$ 254803059 $$ 259333059 $$ 316763061 $$ 297453061 $$ 392843061 $$ 392853062 $$ 416293066 $$ 412593067 $$ 183993069 $$ 172753070 $$ 83233070 $$ 85723070 $$ 114783070 $$ 265343071 $$ 74353071 $$ 83313072 $$ 43573072 $$ 75643072 $$ 90183072 $$ 116563073 $$ 33473073 $$ 389443073 $$ 389453075 $$ 97293075 $$ 100293075 $$ 395453076 $$ 153023077 $$ 33283077 $$ 36333077 $$ 73783083 $$ 59793084 $$ 175373087 $$ 43683087 $$ 191113087 $$ 195743087 $$ 309083088 $$ 32923088 $$ 38933088 $$ 86703088 $$ 160713088 $$ 289593090 $$ 42863090 $$ 43723090 $$ 51593090 $$ 93743090 $$ 162223091 $$ 202963092 $$ 373133093 $$ 269843095 $$ 347573097 $$ 377223098 $$ 55033098 $$ 55043098 $$ 66803098 $$ 86653098 $$ 89993098 $$ 108133098 $$ 110033098 $$ 134803098 $$ 160173098 $$ 160183098 $$ 202703098 $$ 338263098 $$ 376203098 $$ 397893098 $$ 397903098 $$ 397913098 $$ 420573098 $$ 435343098 $$ 442083098 $$ 442093099 $$ 389303100 $$ 71973100 $$ 110113100 $$ 160943100 $$ 398453100 $$ 398463100 $$ 398473103 $$ 47543103 $$ 148143103 $$ 244633103 $$ 259333103 $$ 316763104 $$ 74303106 $$ 51923106 $$ 65053106 $$ 420233106 $$ 429623108 $$ 39963108 $$ 58083108 $$ 58093108 $$ 64053108 $$ 66943108 $$ 68113108 $$ 68123108 $$ 125973108 $$ 129033108 $$ 129723108 $$ 163503108 $$ 302113108 $$ 393543109 $$ 179653109 $$ 195373110 $$ 57343110 $$ 69053110 $$ 73813110 $$ 84303111 $$ 370853112 $$ 388433112 $$ 406433113 $$ 285593117 $$ 348853120 $$ 198993120 $$ 441803121 $$ 383763125 $$ 224243126 $$ 307673126 $$ 307683127 $$ 240363127 $$ 252743128 $$ 32643128 $$ 87563128 $$ 103483128 $$ 229393130 $$ 35163130 $$ 53363130 $$ 337173131 $$ 52743131 $$ 92933131 $$ 366183132 $$ 241243135 $$ 39763136 $$ 41583136 $$ 146963136 $$ 241313138 $$ 317013141 $$ 406223146 $$ 34173146 $$ 34823146 $$ 35343147 $$ 310313148 $$ 198003148 $$ 207013151 $$ 40273151 $$ 60263151 $$ 69193151 $$ 331283151 $$ 393733152 $$ 412873154 $$ 220873156 $$ 65953156 $$ 66743156 $$ 81423157 $$ 47863157 $$ 65653157 $$ 256313157 $$ 311533158 $$ 207473159 $$ 76383161 $$ 37783161 $$ 53863161 $$ 53873161 $$ 53883161 $$ 53893161 $$ 407893162 $$ 33413163 $$ 79023164 $$ 32403169 $$ 34323169 $$ 154213169 $$ 330213172 $$ 34803172 $$ 43503172 $$ 47663172 $$ 58863172 $$ 63703172 $$ 79033172 $$ 90453172 $$ 111533172 $$ 111543172 $$ 111553172 $$ 111563172 $$ 291533172 $$ 313583172 $$ 313593173 $$ 111033173 $$ 289683173 $$ 292543174 $$ 43553174 $$ 410443175 $$ 423183178 $$ 67433186 $$ 166393186 $$ 358103187 $$ 68693187 $$ 329913187 $$ 393363191 $$ 315183192 $$ 400023193 $$ 81073193 $$ 363853194 $$ 213113195 $$ 36483195 $$ 57833198 $$ 60913198 $$ 71953198 $$ 71963198 $$ 73453198 $$ 75853198 $$ 75863198 $$ 76323198 $$ 85343198 $$ 110923198 $$ 112233198 $$ 113683198 $$ 116543198 $$ 142463198 $$ 160123198 $$ 160133198 $$ 239813198 $$ 239823198 $$ 283743198 $$ 283753198 $$ 303113198 $$ 303123198 $$ 303133198 $$ 305883198 $$ 305893198 $$ 305903198 $$ 305913198 $$ 310283198 $$ 310293198 $$ 310303198 $$ 311863198 $$ 312993198 $$ 346653198 $$ 346663198 $$ 346673198 $$ 346683198 $$ 351053198 $$ 351063198 $$ 351073198 $$ 351083198 $$ 351093198 $$ 357233198 $$ 398483201 $$ 52823201 $$ 227683201 $$ 422933202 $$ 107153203 $$ 167713203 $$ 347633205 $$ 49743205 $$ 98073205 $$ 156043205 $$ 305833205 $$ 326243206 $$ 358873208 $$ 39823208 $$ 61173208 $$ 77803208 $$ 90753208 $$ 145053208 $$ 227853208 $$ 272273208 $$ 336153208 $$ 396053209 $$ 416263210 $$ 81823212 $$ 32963212 $$ 244593214 $$ 95053215 $$ 36513215 $$ 424623217 $$ 314813218 $$ 267003221 $$ 55443222 $$ 33213222 $$ 34423222 $$ 34433222 $$ 45283222 $$ 47493222 $$ 50323222 $$ 50333222 $$ 96573222 $$ 98083222 $$ 98363222 $$ 143163222 $$ 146343223 $$ 39673223 $$ 288623227 $$ 295433227 $$ 304953227 $$ 413123228 $$ 332903228 $$ 418803229 $$ 296573229 $$ 308663230 $$ 154393230 $$ 331203230 $$ 331213234 $$ 314903235 $$ 38223235 $$ 56113235 $$ 244213235 $$ 437393236 $$ 34613236 $$ 125913238 $$ 274583239 $$ 202403240 $$ 224383240 $$ 230773241 $$ 33443241 $$ 34443241 $$ 243923244 $$ 47243245 $$ 45883245 $$ 86363245 $$ 147003245 $$ 336963247 $$ 71123247 $$ 84483247 $$ 86373247 $$ 383973248 $$ 43223248 $$ 54773248 $$ 70303248 $$ 72043248 $$ 72083248 $$ 79873248 $$ 80913248 $$ 91863248 $$ 135633248 $$ 376183248 $$ 435383249 $$ 187473250 $$ 152903253 $$ 429583254 $$ 141173255 $$ 296243256 $$ 59233257 $$ 44783259 $$ 32603259 $$ 163593260 $$ 163593262 $$ 53063262 $$ 365633263 $$ 58003263 $$ 59413263 $$ 67973263 $$ 85963263 $$ 279423264 $$ 33693264 $$ 103483264 $$ 229393264 $$ 360583265 $$ 265003269 $$ 47463270 $$ 412963270 $$ 434163271 $$ 198593272 $$ 37263272 $$ 50203272 $$ 72183272 $$ 78683272 $$ 140393272 $$ 273613273 $$ 377423273 $$ 419503275 $$ 266393276 $$ 38713278 $$ 76503278 $$ 380953278 $$ 390593282 $$ 78403282 $$ 249183283 $$ 163943287 $$ 142793290 $$ 434063291 $$ 43803292 $$ 289593293 $$ 121843293 $$ 197623294 $$ 69303295 $$ 99663295 $$ 167293299 $$ 246643300 $$ 71813300 $$ 395193303 $$ 40213303 $$ 66123303 $$ 123723303 $$ 131813303 $$ 336413303 $$ 350883303 $$ 391993303 $$ 400663304 $$ 50683304 $$ 347623304 $$ 391823305 $$ 57053305 $$ 358433306 $$ 49663306 $$ 352143306 $$ 369463308 $$ 33093311 $$ 377173312 $$ 146873315 $$ 38643315 $$ 53673315 $$ 57843315 $$ 78593315 $$ 89693315 $$ 111823317 $$ 47413319 $$ 363473320 $$ 34303320 $$ 45263320 $$ 50303320 $$ 53273320 $$ 71473320 $$ 146173320 $$ 380933321 $$ 34423321 $$ 34433321 $$ 96573321 $$ 98083321 $$ 240953323 $$ 276913325 $$ 123913327 $$ 35993327 $$ 61583327 $$ 61973327 $$ 61983327 $$ 334963328 $$ 36333328 $$ 221403331 $$ 112553332 $$ 51563332 $$ 229353334 $$ 41113334 $$ 85313335 $$ 34333335 $$ 34923335 $$ 65253335 $$ 266973336 $$ 64053336 $$ 97013336 $$ 97063336 $$ 129033336 $$ 179763339 $$ 50993339 $$ 73283339 $$ 97293339 $$ 190783339 $$ 304863339 $$ 315313339 $$ 435583339 $$ 435593340 $$ 38563340 $$ 78103341 $$ 244223343 $$ 48163343 $$ 48633343 $$ 76013343 $$ 82393344 $$ 34443344 $$ 243923348 $$ 293513349 $$ 41723349 $$ 100903349 $$ 143423349 $$ 150173350 $$ 42573351 $$ 111433352 $$ 141583354 $$ 354323354 $$ 368513356 $$ 52373356 $$ 59113356 $$ 83653356 $$ 402683357 $$ 72793357 $$ 75713357 $$ 106183357 $$ 134243357 $$ 167023357 $$ 263433357 $$ 439113360 $$ 379023361 $$ 55343361 $$ 65713361 $$ 143013361 $$ 245563361 $$ 249003362 $$ 56523363 $$ 41953363 $$ 137723363 $$ 139143363 $$ 248943364 $$ 202873364 $$ 439903364 $$ 439913366 $$ 377973367 $$ 201413367 $$ 429913369 $$ 160823369 $$ 360583370 $$ 342203375 $$ 413143376 $$ 333123378 $$ 337653379 $$ 332413380 $$ 173883381 $$ 204833382 $$ 38553387 $$ 38323387 $$ 142933387 $$ 245823388 $$ 169203389 $$ 334973389 $$ 377693390 $$ 34273390 $$ 35303390 $$ 141433390 $$ 295893390 $$ 295903399 $$ 235333401 $$ 89023403 $$ 43243408 $$ 44393409 $$ 257913412 $$ 41983412 $$ 55793412 $$ 251523412 $$ 266283413 $$ 54973415 $$ 411933416 $$ 329313417 $$ 34823417 $$ 35343418 $$ 441003419 $$ 43273422 $$ 165853424 $$ 171653424 $$ 267423424 $$ 377353425 $$ 34573425 $$ 54173425 $$ 67123425 $$ 68213425 $$ 73203425 $$ 118763425 $$ 129623426 $$ 116503426 $$ 342313427 $$ 35303427 $$ 35353427 $$ 141433427 $$ 299913430 $$ 35763430 $$ 45263430 $$ 50303430 $$ 53273430 $$ 71473430 $$ 380943431 $$ 35803431 $$ 35813432 $$ 330213433 $$ 34923433 $$ 65253433 $$ 266973434 $$ 263513435 $$ 197933435 $$ 387613436 $$ 236163437 $$ 58943440 $$ 215033440 $$ 427003442 $$ 96573442 $$ 98083443 $$ 73603443 $$ 240953445 $$ 141343445 $$ 381353445 $$ 442103449 $$ 61933449 $$ 82323449 $$ 95813449 $$ 101733449 $$ 162563449 $$ 266783449 $$ 402033452 $$ 36033452 $$ 44433452 $$ 57443452 $$ 57453453 $$ 147653459 $$ 37483459 $$ 53773460 $$ 41923460 $$ 308773462 $$ 34633462 $$ 180013462 $$ 180023463 $$ 180013463 $$ 180023465 $$ 67463465 $$ 112023465 $$ 143973465 $$ 191223465 $$ 295993465 $$ 296003465 $$ 296013472 $$ 58573472 $$ 73393472 $$ 85033472 $$ 295963474 $$ 90313477 $$ 288233477 $$ 311293477 $$ 316963478 $$ 281423480 $$ 43503480 $$ 47663480 $$ 58863480 $$ 63703480 $$ 79033480 $$ 88993480 $$ 240833481 $$ 420363481 $$ 437633483 $$ 60293483 $$ 90293483 $$ 443163484 $$ 394543485 $$ 68993486 $$ 161493487 $$ 38043487 $$ 48473487 $$ 58963487 $$ 129833489 $$ 48483489 $$ 86953489 $$ 156483489 $$ 195733489 $$ 274483492 $$ 65253492 $$ 253533492 $$ 266973493 $$ 59003493 $$ 60023493 $$ 63863493 $$ 69203493 $$ 94063493 $$ 99613493 $$ 100023493 $$ 160643493 $$ 187713493 $$ 188353493 $$ 195223493 $$ 384833495 $$ 104503496 $$ 34973496 $$ 35073496 $$ 43593496 $$ 64943496 $$ 76763496 $$ 81303496 $$ 85023496 $$ 85693496 $$ 108113496 $$ 129083496 $$ 135163496 $$ 147083496 $$ 200173496 $$ 256903496 $$ 256913496 $$ 365933497 $$ 35073497 $$ 64943497 $$ 76763497 $$ 81303497 $$ 85693497 $$ 103443497 $$ 108113497 $$ 111263497 $$ 135163497 $$ 147083497 $$ 200173497 $$ 256903497 $$ 256913497 $$ 439003499 $$ 333753499 $$ 343513499 $$ 419903499 $$ 419913500 $$ 35013501 $$ 154003503 $$ 132243503 $$ 253263507 $$ 43593507 $$ 64943507 $$ 76763507 $$ 81303507 $$ 85023507 $$ 85693507 $$ 108113507 $$ 135163507 $$ 147083507 $$ 200173507 $$ 256903507 $$ 256913511 $$ 47123511 $$ 342973512 $$ 197383513 $$ 38363513 $$ 47573513 $$ 53333513 $$ 57283513 $$ 59403513 $$ 82233513 $$ 162523513 $$ 162533513 $$ 175833513 $$ 175843513 $$ 342503516 $$ 337173517 $$ 86063517 $$ 431653517 $$ 433903518 $$ 412043525 $$ 35263525 $$ 36723525 $$ 39393525 $$ 43923525 $$ 46353525 $$ 89483525 $$ 109363525 $$ 112813525 $$ 168193525 $$ 350183525 $$ 365573525 $$ 389763526 $$ 36723526 $$ 39393526 $$ 43923526 $$ 46353526 $$ 89483526 $$ 109363526 $$ 112813526 $$ 168193526 $$ 282173526 $$ 350183526 $$ 382313526 $$ 389763528 $$ 65173528 $$ 81063528 $$ 167423530 $$ 141433530 $$ 295893530 $$ 295903535 $$ 299913536 $$ 98233536 $$ 238743536 $$ 249263547 $$ 245553551 $$ 357083554 $$ 419023555 $$ 378093558 $$ 70833559 $$ 103823559 $$ 128883559 $$ 261513562 $$ 124793563 $$ 316793565 $$ 199443565 $$ 423943567 $$ 36703567 $$ 433013569 $$ 87623570 $$ 54313570 $$ 241033571 $$ 37183571 $$ 45003571 $$ 236843571 $$ 236853571 $$ 326903574 $$ 343183574 $$ 360243575 $$ 79343575 $$ 220103575 $$ 315163576 $$ 45263576 $$ 53273577 $$ 62633580 $$ 35813582 $$ 125393582 $$ 136783583 $$ 37263583 $$ 50203583 $$ 53103583 $$ 77743584 $$ 36123584 $$ 68083586 $$ 49793586 $$ 287193588 $$ 436863589 $$ 89933589 $$ 89943589 $$ 217683589 $$ 217693589 $$ 226343591 $$ 40123591 $$ 151533591 $$ 412123592 $$ 208843592 $$ 318433594 $$ 77073595 $$ 294283598 $$ 35993598 $$ 53413598 $$ 124083599 $$ 53413599 $$ 61583599 $$ 61973599 $$ 61983599 $$ 124083602 $$ 44943602 $$ 52083602 $$ 55373603 $$ 44433603 $$ 245593605 $$ 255873606 $$ 65413606 $$ 135183606 $$ 138923606 $$ 138933606 $$ 150223606 $$ 158603606 $$ 193043606 $$ 291093606 $$ 409853606 $$ 415883606 $$ 441753606 $$ 441763608 $$ 264393612 $$ 57963612 $$ 68083612 $$ 126763613 $$ 53943613 $$ 228283614 $$ 180053621 $$ 358563622 $$ 45833622 $$ 50183622 $$ 292083622 $$ 306863627 $$ 306273629 $$ 335703630 $$ 246893630 $$ 246903632 $$ 202833632 $$ 368973632 $$ 370043632 $$ 440683633 $$ 39673637 $$ 36563638 $$ 48413639 $$ 54423640 $$ 325533641 $$ 151663643 $$ 44213646 $$ 72393646 $$ 141943646 $$ 144373647 $$ 45683647 $$ 51373647 $$ 75633647 $$ 75643647 $$ 110173647 $$ 289003647 $$ 374983648 $$ 43973648 $$ 46103648 $$ 58003648 $$ 67973649 $$ 83993649 $$ 105043651 $$ 132263651 $$ 424623652 $$ 132753654 $$ 203483654 $$ 400083655 $$ 39723657 $$ 40313657 $$ 41053657 $$ 41293657 $$ 59423657 $$ 69893657 $$ 90603657 $$ 109903657 $$ 125143658 $$ 43763658 $$ 82033658 $$ 354013661 $$ 172523661 $$ 435653662 $$ 243503663 $$ 387623665 $$ 65183665 $$ 434553666 $$ 397103666 $$ 435213667 $$ 102223667 $$ 338753667 $$ 347153668 $$ 173603669 $$ 314253671 $$ 38503671 $$ 358703671 $$ 358713671 $$ 374663671 $$ 374673672 $$ 43923672 $$ 46353672 $$ 89483672 $$ 109363672 $$ 168193672 $$ 350183673 $$ 36743673 $$ 36753673 $$ 36763673 $$ 36773673 $$ 38533673 $$ 39383673 $$ 55213673 $$ 65873673 $$ 86573673 $$ 89473673 $$ 116493673 $$ 168203673 $$ 207933673 $$ 319293673 $$ 319303673 $$ 330163673 $$ 339243673 $$ 349923673 $$ 365323673 $$ 367093674 $$ 36753674 $$ 36763674 $$ 36773674 $$ 38533674 $$ 39383674 $$ 65873674 $$ 86573674 $$ 89473674 $$ 116493674 $$ 319293674 $$ 319303674 $$ 330163674 $$ 349923674 $$ 365323674 $$ 367093675 $$ 36763675 $$ 36773675 $$ 38533675 $$ 39383675 $$ 65873675 $$ 86573675 $$ 89473675 $$ 116493675 $$ 319293675 $$ 319303675 $$ 330163675 $$ 349923675 $$ 365323675 $$ 367093676 $$ 36773676 $$ 38533676 $$ 39383676 $$ 65873676 $$ 79243676 $$ 116493676 $$ 207943676 $$ 349923677 $$ 38533677 $$ 39383677 $$ 65873677 $$ 79243677 $$ 116493677 $$ 349923678 $$ 55933681 $$ 60153681 $$ 65763681 $$ 360103681 $$ 360113683 $$ 136823683 $$ 218443686 $$ 49503687 $$ 334253688 $$ 242283688 $$ 246403690 $$ 252513690 $$ 255953690 $$ 260703691 $$ 41763691 $$ 108753691 $$ 273413698 $$ 108753702 $$ 92393702 $$ 408723702 $$ 408733703 $$ 308223706 $$ 283213707 $$ 130533710 $$ 73073710 $$ 176343710 $$ 216723710 $$ 221963713 $$ 275583713 $$ 286403717 $$ 358473718 $$ 45003718 $$ 236843718 $$ 236853720 $$ 242903721 $$ 69283721 $$ 372273723 $$ 37853723 $$ 97803723 $$ 182073725 $$ 263873726 $$ 140393726 $$ 273613727 $$ 121483729 $$ 37303735 $$ 66923735 $$ 331033737 $$ 45763737 $$ 364253737 $$ 423093738 $$ 159453738 $$ 316343738 $$ 352063738 $$ 357373739 $$ 252423739 $$ 254973742 $$ 95473742 $$ 316743743 $$ 43013743 $$ 47313743 $$ 68423743 $$ 83293743 $$ 83893743 $$ 85193743 $$ 87753743 $$ 276083743 $$ 279973743 $$ 305043744 $$ 114183746 $$ 73643746 $$ 308903747 $$ 123303747 $$ 432583747 $$ 435103748 $$ 244493749 $$ 57843752 $$ 45203757 $$ 45423759 $$ 191273760 $$ 39263760 $$ 146893763 $$ 47783767 $$ 45893770 $$ 74963776 $$ 295463778 $$ 407893778 $$ 425933779 $$ 43533779 $$ 48043779 $$ 63893779 $$ 159673779 $$ 178483785 $$ 97803785 $$ 182073788 $$ 235413789 $$ 54093790 $$ 45713790 $$ 54103790 $$ 54113790 $$ 86643790 $$ 106113793 $$ 194853793 $$ 194863794 $$ 86523794 $$ 200223794 $$ 422173796 $$ 51303796 $$ 59073796 $$ 128643798 $$ 250813800 $$ 41563801 $$ 372263804 $$ 58963804 $$ 129833805 $$ 289853808 $$ 67713808 $$ 391263810 $$ 290013812 $$ 54733812 $$ 159473812 $$ 298173816 $$ 147893818 $$ 41063818 $$ 59173818 $$ 59193818 $$ 69443818 $$ 83113818 $$ 130163818 $$ 333323821 $$ 60283821 $$ 65973821 $$ 85243821 $$ 254983822 $$ 43313822 $$ 100483822 $$ 100493822 $$ 100503822 $$ 100513823 $$ 42203823 $$ 226043825 $$ 98923825 $$ 234183826 $$ 154883827 $$ 257513828 $$ 250543829 $$ 170233829 $$ 368733830 $$ 56123830 $$ 67543830 $$ 69913830 $$ 376813830 $$ 411953831 $$ 366613832 $$ 142933832 $$ 245823834 $$ 41283834 $$ 277493836 $$ 53333836 $$ 162523836 $$ 162533836 $$ 173313836 $$ 342503838 $$ 342963838 $$ 407393839 $$ 52933840 $$ 101503840 $$ 391083840 $$ 391093842 $$ 52633842 $$ 86153842 $$ 367643844 $$ 54293844 $$ 264383844 $$ 333213844 $$ 340433845 $$ 41993846 $$ 45233846 $$ 265773848 $$ 351603850 $$ 429243851 $$ 51013853 $$ 39383853 $$ 65873853 $$ 79243853 $$ 116493853 $$ 349933854 $$ 41473855 $$ 55123855 $$ 388213856 $$ 43343858 $$ 257523858 $$ 257533859 $$ 68713859 $$ 108023859 $$ 218973864 $$ 78593864 $$ 276593866 $$ 427173867 $$ 77953870 $$ 240693874 $$ 141803876 $$ 41673883 $$ 251053885 $$ 55683892 $$ 41803896 $$ 246003897 $$ 250873919 $$ 64563919 $$ 146083921 $$ 359993926 $$ 109063927 $$ 40163927 $$ 252123935 $$ 55053936 $$ 150073936 $$ 251333937 $$ 294713938 $$ 65873938 $$ 79243938 $$ 116493938 $$ 259383938 $$ 349933939 $$ 46353939 $$ 55203939 $$ 109363939 $$ 144063939 $$ 242503941 $$ 42443942 $$ 115383942 $$ 369703943 $$ 376433944 $$ 353713948 $$ 159993953 $$ 287173954 $$ 42503954 $$ 56363954 $$ 60563954 $$ 72023954 $$ 79993954 $$ 156323954 $$ 283043954 $$ 283053954 $$ 288253954 $$ 296543954 $$ 296553954 $$ 405643954 $$ 405653954 $$ 405663954 $$ 432513955 $$ 295023959 $$ 49543959 $$ 90003959 $$ 101203959 $$ 386523962 $$ 60873962 $$ 66933964 $$ 66723964 $$ 73013964 $$ 91273964 $$ 340053964 $$ 360913966 $$ 299033968 $$ 44033970 $$ 42693970 $$ 416933971 $$ 103433972 $$ 71763974 $$ 333483974 $$ 431903978 $$ 111863978 $$ 158783982 $$ 145053985 $$ 42983985 $$ 372703985 $$ 408133988 $$ 59253988 $$ 106043988 $$ 348653992 $$ 39933992 $$ 41373993 $$ 41373995 $$ 53053995 $$ 57063995 $$ 304663996 $$ 64053996 $$ 129033996 $$ 163503996 $$ 233783996 $$ 302113996 $$ 393543996 $$ 411483997 $$ 425324002 $$ 343964005 $$ 382824005 $$ 386154005 $$ 430764007 $$ 158184007 $$ 289814008 $$ 44684012 $$ 412124015 $$ 79464016 $$ 252124019 $$ 40494019 $$ 395824021 $$ 66124021 $$ 123724021 $$ 131814021 $$ 350884022 $$ 44724022 $$ 57434022 $$ 79774022 $$ 296594022 $$ 296604023 $$ 181764023 $$ 193224024 $$ 73384024 $$ 83874025 $$ 233664028 $$ 319884029 $$ 77544029 $$ 77694031 $$ 41054031 $$ 41294031 $$ 59424031 $$ 69894031 $$ 90604031 $$ 109904040 $$ 75094041 $$ 50594043 $$ 79854044 $$ 96684045 $$ 40464045 $$ 49724045 $$ 106074046 $$ 41574046 $$ 106074049 $$ 395824051 $$ 282724052 $$ 225754053 $$ 68034053 $$ 193924053 $$ 425254055 $$ 125784059 $$ 43264059 $$ 45594059 $$ 47974059 $$ 53844059 $$ 53854059 $$ 106314059 $$ 125874059 $$ 125884059 $$ 130104059 $$ 131024059 $$ 131034059 $$ 131044059 $$ 131054059 $$ 181494059 $$ 195964060 $$ 43534060 $$ 48044060 $$ 48054060 $$ 101064060 $$ 109744060 $$ 112144060 $$ 122024060 $$ 178514060 $$ 184594064 $$ 109484064 $$ 165294064 $$ 370684065 $$ 283144068 $$ 409744069 $$ 206044069 $$ 335324069 $$ 335334071 $$ 74564072 $$ 256614073 $$ 44054074 $$ 197444075 $$ 273154076 $$ 50444076 $$ 54234076 $$ 54244076 $$ 56054076 $$ 71734076 $$ 114284076 $$ 153574076 $$ 153584076 $$ 272604078 $$ 83524081 $$ 304974083 $$ 87714083 $$ 245324088 $$ 58794091 $$ 48314091 $$ 52474091 $$ 159774092 $$ 40934092 $$ 167584092 $$ 211384092 $$ 424024093 $$ 167584093 $$ 424024095 $$ 114114096 $$ 99784096 $$ 135414099 $$ 86314099 $$ 88144099 $$ 395384101 $$ 128724102 $$ 231854103 $$ 159004104 $$ 53054104 $$ 78564105 $$ 41294105 $$ 59424105 $$ 69894105 $$ 90604105 $$ 109904105 $$ 125144105 $$ 441294106 $$ 59174106 $$ 59194106 $$ 69444106 $$ 83114106 $$ 130164106 $$ 333324107 $$ 334564113 $$ 375364117 $$ 49804117 $$ 56314117 $$ 288484119 $$ 351814126 $$ 86114126 $$ 197264126 $$ 386774127 $$ 438694129 $$ 59424129 $$ 69894129 $$ 84974129 $$ 134284129 $$ 166224129 $$ 346324129 $$ 372744129 $$ 424234129 $$ 441634130 $$ 42344130 $$ 94214141 $$ 91014141 $$ 198284143 $$ 111644143 $$ 204164143 $$ 292214146 $$ 364624146 $$ 367144148 $$ 41504148 $$ 146774148 $$ 174894148 $$ 424304150 $$ 174894150 $$ 202464150 $$ 424294152 $$ 46454155 $$ 51354155 $$ 66424155 $$ 70814155 $$ 77304156 $$ 48884158 $$ 241314162 $$ 44994162 $$ 340194162 $$ 379604163 $$ 146624172 $$ 48304174 $$ 296224181 $$ 46694181 $$ 101624181 $$ 142124181 $$ 236284181 $$ 243244185 $$ 146214186 $$ 300884198 $$ 251524200 $$ 52384202 $$ 48454202 $$ 109224205 $$ 306974210 $$ 266504210 $$ 282824210 $$ 305384215 $$ 111584216 $$ 138104216 $$ 296344217 $$ 42184221 $$ 296084221 $$ 296094221 $$ 307484223 $$ 304794226 $$ 42274226 $$ 42284227 $$ 42284229 $$ 294164234 $$ 146084238 $$ 301064244 $$ 293054244 $$ 293064250 $$ 56364250 $$ 60564250 $$ 72024250 $$ 79994250 $$ 156324250 $$ 283044250 $$ 283054250 $$ 288254250 $$ 296544250 $$ 296554250 $$ 432514254 $$ 79934254 $$ 308804254 $$ 312664254 $$ 355084256 $$ 83814257 $$ 312514257 $$ 312524262 $$ 332364262 $$ 378374263 $$ 44874264 $$ 44104264 $$ 74124266 $$ 42674266 $$ 44904266 $$ 76494266 $$ 117014266 $$ 225904267 $$ 44904267 $$ 76494267 $$ 117014267 $$ 340224268 $$ 79214270 $$ 405934273 $$ 44494273 $$ 158674274 $$ 66694274 $$ 66704274 $$ 118574274 $$ 162734278 $$ 304154278 $$ 304164279 $$ 335904282 $$ 52214284 $$ 50084284 $$ 74784286 $$ 51594286 $$ 93744286 $$ 114594286 $$ 358454286 $$ 364954289 $$ 99674290 $$ 290704290 $$ 409484291 $$ 294914293 $$ 43834293 $$ 52434293 $$ 59524293 $$ 64824293 $$ 72834295 $$ 285004296 $$ 318144298 $$ 366484302 $$ 407874308 $$ 50474309 $$ 312634309 $$ 312644310 $$ 78434310 $$ 79824310 $$ 82574310 $$ 90014310 $$ 96484310 $$ 165104310 $$ 165114310 $$ 165124310 $$ 299684310 $$ 299694310 $$ 338044310 $$ 362274310 $$ 403184311 $$ 48744313 $$ 112404314 $$ 51564318 $$ 45504318 $$ 245754318 $$ 245764320 $$ 103504321 $$ 45984321 $$ 196334321 $$ 286424321 $$ 364224322 $$ 54774322 $$ 70304322 $$ 74134322 $$ 74854322 $$ 80914322 $$ 129924322 $$ 129934322 $$ 135634322 $$ 153854322 $$ 160234322 $$ 338284322 $$ 368094322 $$ 376184326 $$ 47964326 $$ 47984326 $$ 47994326 $$ 48004329 $$ 60244330 $$ 88524331 $$ 100484331 $$ 100494331 $$ 100504331 $$ 100514332 $$ 58664332 $$ 88704333 $$ 260094333 $$ 260104333 $$ 260114333 $$ 260124333 $$ 394744333 $$ 409054334 $$ 65164334 $$ 85854340 $$ 285514342 $$ 256224342 $$ 421574343 $$ 248114347 $$ 307544350 $$ 47664350 $$ 58864350 $$ 63704350 $$ 90454350 $$ 111534350 $$ 111544350 $$ 111554350 $$ 111564350 $$ 313584350 $$ 313594351 $$ 49574351 $$ 412114353 $$ 48044353 $$ 63894353 $$ 159674353 $$ 160874355 $$ 69194355 $$ 410444355 $$ 435934356 $$ 51304356 $$ 51474356 $$ 59074356 $$ 100054356 $$ 187604356 $$ 287644356 $$ 287654356 $$ 361604359 $$ 49904359 $$ 99844359 $$ 99854362 $$ 343294362 $$ 416254362 $$ 420414363 $$ 100794364 $$ 63774364 $$ 66284364 $$ 158914364 $$ 159784369 $$ 47634369 $$ 112344369 $$ 422564372 $$ 51594372 $$ 162224373 $$ 425344373 $$ 425354374 $$ 47604374 $$ 79844376 $$ 82034383 $$ 55944383 $$ 59524383 $$ 64824384 $$ 207504392 $$ 46354392 $$ 89484392 $$ 112814392 $$ 350184392 $$ 365574392 $$ 382314392 $$ 389764393 $$ 338964395 $$ 66094397 $$ 46104400 $$ 45044400 $$ 45054400 $$ 79064400 $$ 211464400 $$ 252204402 $$ 83684403 $$ 365414405 $$ 83354405 $$ 110544407 $$ 410254407 $$ 410264410 $$ 141144414 $$ 44154414 $$ 253024415 $$ 253024417 $$ 238424428 $$ 235964434 $$ 235634443 $$ 245594445 $$ 252094449 $$ 158674451 $$ 45284451 $$ 47494451 $$ 50324451 $$ 50334451 $$ 73604451 $$ 82204451 $$ 84284451 $$ 293554456 $$ 383694456 $$ 388614456 $$ 431534458 $$ 61714463 $$ 56204466 $$ 291994472 $$ 57434472 $$ 79774473 $$ 56244473 $$ 91394473 $$ 129904473 $$ 296614475 $$ 52614475 $$ 111894476 $$ 48244476 $$ 60404476 $$ 281414483 $$ 116204483 $$ 382154483 $$ 419984484 $$ 343154485 $$ 162514485 $$ 162934485 $$ 175694485 $$ 330344486 $$ 44884486 $$ 81384488 $$ 108284489 $$ 59104490 $$ 76494490 $$ 117014490 $$ 340224491 $$ 47214491 $$ 59484491 $$ 295604493 $$ 48684493 $$ 73154494 $$ 52084494 $$ 55374497 $$ 45994497 $$ 47884497 $$ 65014497 $$ 70254497 $$ 76634497 $$ 76854497 $$ 86584497 $$ 367694497 $$ 383184497 $$ 387314504 $$ 45054504 $$ 79064504 $$ 252204505 $$ 79064505 $$ 252204507 $$ 422754507 $$ 422764510 $$ 55984510 $$ 72324512 $$ 121054512 $$ 346714514 $$ 67554514 $$ 157554515 $$ 92344516 $$ 314114518 $$ 420334523 $$ 47864523 $$ 83404523 $$ 392534525 $$ 122714525 $$ 349664525 $$ 349674527 $$ 152294528 $$ 47494528 $$ 50324528 $$ 50334528 $$ 122864528 $$ 293554530 $$ 255374534 $$ 177384535 $$ 58504535 $$ 110054535 $$ 162054535 $$ 304614535 $$ 323364535 $$ 344964535 $$ 370924535 $$ 370934535 $$ 370944536 $$ 162174536 $$ 431034537 $$ 290094538 $$ 50114538 $$ 95064539 $$ 181574539 $$ 193274541 $$ 230844544 $$ 294474551 $$ 314024555 $$ 217844555 $$ 372104555 $$ 372114556 $$ 52464556 $$ 52504557 $$ 47284557 $$ 342664557 $$ 343484557 $$ 373254559 $$ 47974559 $$ 53844559 $$ 53854559 $$ 106314559 $$ 125884559 $$ 130104559 $$ 181494559 $$ 269564569 $$ 58394569 $$ 189464571 $$ 54104571 $$ 54114571 $$ 86644571 $$ 203694572 $$ 175794574 $$ 48214578 $$ 223724581 $$ 236194581 $$ 237214583 $$ 50174583 $$ 50184583 $$ 81164583 $$ 173824583 $$ 185734586 $$ 68924586 $$ 68934586 $$ 68944586 $$ 74984589 $$ 71364590 $$ 214094590 $$ 330354590 $$ 413104591 $$ 45924591 $$ 54444591 $$ 161504592 $$ 54444592 $$ 161504593 $$ 48444593 $$ 76444593 $$ 84264593 $$ 112834593 $$ 214084593 $$ 413084598 $$ 108204598 $$ 348614598 $$ 364224599 $$ 47884599 $$ 65014599 $$ 70254599 $$ 76634599 $$ 76854599 $$ 86584599 $$ 367694599 $$ 383184599 $$ 387314599 $$ 432764599 $$ 432774599 $$ 432784600 $$ 77594600 $$ 128504602 $$ 51434602 $$ 99544602 $$ 99554602 $$ 128944603 $$ 264644603 $$ 397414603 $$ 410394603 $$ 410454606 $$ 62114606 $$ 95154606 $$ 135194606 $$ 291104609 $$ 58904609 $$ 150694614 $$ 377934614 $$ 389844621 $$ 48734621 $$ 343664621 $$ 394104621 $$ 406264621 $$ 437604623 $$ 125644624 $$ 207604626 $$ 332614626 $$ 341674626 $$ 422914628 $$ 113384628 $$ 344714629 $$ 81524630 $$ 390874633 $$ 428634635 $$ 89484635 $$ 109364635 $$ 168194635 $$ 350184635 $$ 365574636 $$ 429424640 $$ 46854641 $$ 373704644 $$ 296974645 $$ 211754645 $$ 211764650 $$ 55954652 $$ 88314652 $$ 107814653 $$ 141794657 $$ 88754657 $$ 146704658 $$ 50494659 $$ 249774662 $$ 78764669 $$ 142124669 $$ 236284671 $$ 250794672 $$ 46734672 $$ 236274672 $$ 240204673 $$ 240204677 $$ 439834679 $$ 89764679 $$ 107344679 $$ 254414681 $$ 254044684 $$ 103414686 $$ 52294686 $$ 107294686 $$ 237804686 $$ 244944686 $$ 248644686 $$ 248654687 $$ 236754692 $$ 78654693 $$ 338174711 $$ 64744711 $$ 343824712 $$ 72714717 $$ 65364717 $$ 90784717 $$ 154774717 $$ 165654717 $$ 166284717 $$ 360404717 $$ 360414718 $$ 49874718 $$ 428974720 $$ 240604721 $$ 52854728 $$ 73324728 $$ 103104728 $$ 210324728 $$ 343484728 $$ 416194728 $$ 416204732 $$ 48224732 $$ 58484732 $$ 58494733 $$ 58054733 $$ 119814735 $$ 49274740 $$ 111954740 $$ 284864741 $$ 359744743 $$ 367534744 $$ 63974745 $$ 61374745 $$ 122144745 $$ 122154746 $$ 323624746 $$ 323634747 $$ 263094749 $$ 50324749 $$ 50334749 $$ 73604749 $$ 82204749 $$ 84284749 $$ 293554750 $$ 50534750 $$ 57234752 $$ 122914754 $$ 244634755 $$ 123104755 $$ 123114756 $$ 341214756 $$ 356914756 $$ 384464758 $$ 415264761 $$ 48184765 $$ 82424765 $$ 91254765 $$ 109954766 $$ 58864766 $$ 63704766 $$ 79034766 $$ 125044769 $$ 146974773 $$ 133824773 $$ 226174776 $$ 50544776 $$ 50554776 $$ 82724776 $$ 82734776 $$ 96584776 $$ 111394776 $$ 125464776 $$ 157264776 $$ 179434776 $$ 179444776 $$ 181834776 $$ 181844776 $$ 181854776 $$ 181864776 $$ 181874778 $$ 271664785 $$ 382364788 $$ 65014788 $$ 70254788 $$ 76634788 $$ 76854788 $$ 86584788 $$ 367694788 $$ 383184788 $$ 387314793 $$ 49894793 $$ 50754793 $$ 51974793 $$ 312254794 $$ 329964794 $$ 329974794 $$ 410174796 $$ 47984796 $$ 47994796 $$ 48004797 $$ 53844797 $$ 53854797 $$ 106314797 $$ 125884797 $$ 130104797 $$ 181494798 $$ 47994798 $$ 48004799 $$ 48004804 $$ 63894804 $$ 159674804 $$ 178484811 $$ 57154811 $$ 429964813 $$ 50904813 $$ 85124813 $$ 192574814 $$ 62714814 $$ 78104814 $$ 439814815 $$ 137304815 $$ 422984816 $$ 48634821 $$ 95594822 $$ 58484822 $$ 58494824 $$ 60404826 $$ 61454826 $$ 129804829 $$ 54264830 $$ 48314838 $$ 75774838 $$ 91194838 $$ 98144838 $$ 100724838 $$ 127214838 $$ 144254838 $$ 146104838 $$ 157834838 $$ 180604838 $$ 194914838 $$ 194924838 $$ 194934838 $$ 194944839 $$ 110184839 $$ 115784839 $$ 328504840 $$ 78324840 $$ 285854842 $$ 51514842 $$ 91634842 $$ 101084844 $$ 112834844 $$ 214084845 $$ 109224845 $$ 128274845 $$ 368914847 $$ 58964848 $$ 271764855 $$ 51444855 $$ 54574855 $$ 187384857 $$ 259164860 $$ 405034861 $$ 352954865 $$ 51114865 $$ 79264865 $$ 92974865 $$ 113314865 $$ 113324865 $$ 113334865 $$ 159844865 $$ 201354865 $$ 201364865 $$ 357254865 $$ 381564865 $$ 381574867 $$ 102194873 $$ 343664873 $$ 394104873 $$ 406264873 $$ 437604874 $$ 225724877 $$ 136714877 $$ 136724877 $$ 439734878 $$ 51424879 $$ 198194880 $$ 332324880 $$ 332334883 $$ 389434884 $$ 240984885 $$ 367384887 $$ 51914887 $$ 53094891 $$ 50244892 $$ 259954894 $$ 72414896 $$ 355394896 $$ 359594896 $$ 359604900 $$ 49014900 $$ 49024900 $$ 52164900 $$ 52184900 $$ 55544900 $$ 148204900 $$ 148214900 $$ 256524901 $$ 49024901 $$ 52164901 $$ 52184901 $$ 55544901 $$ 148204901 $$ 148214901 $$ 256524902 $$ 52164902 $$ 52184902 $$ 55544902 $$ 148204902 $$ 148214902 $$ 256524904 $$ 148294904 $$ 148304907 $$ 107824913 $$ 75034913 $$ 141234913 $$ 254074929 $$ 163194942 $$ 142404942 $$ 235534954 $$ 66254954 $$ 86774954 $$ 90704954 $$ 138614954 $$ 172694954 $$ 384884957 $$ 171094957 $$ 261894957 $$ 296204957 $$ 380344960 $$ 410414965 $$ 420014966 $$ 436844967 $$ 328204968 $$ 56414968 $$ 136774971 $$ 282404979 $$ 287194988 $$ 64064994 $$ 52904994 $$ 75494998 $$ 119064999 $$ 119095000 $$ 50015000 $$ 52965001 $$ 52965003 $$ 50045003 $$ 56935004 $$ 56935007 $$ 51655007 $$ 53315007 $$ 168885007 $$ 364585008 $$ 119565012 $$ 392315013 $$ 53085014 $$ 261805018 $$ 60635018 $$ 287895018 $$ 313285020 $$ 72185021 $$ 95315024 $$ 113135024 $$ 352825032 $$ 50335032 $$ 293555033 $$ 293555034 $$ 315835035 $$ 123065035 $$ 373395036 $$ 170175038 $$ 68555038 $$ 128065038 $$ 230465038 $$ 230475044 $$ 54235044 $$ 54245047 $$ 67575050 $$ 263445051 $$ 57475051 $$ 82245051 $$ 263285053 $$ 57235060 $$ 62155062 $$ 53635062 $$ 64435062 $$ 129985062 $$ 427325064 $$ 83725064 $$ 99285065 $$ 253255066 $$ 377625067 $$ 51575067 $$ 73845067 $$ 75855067 $$ 370165068 $$ 373945068 $$ 391825074 $$ 64115081 $$ 59855081 $$ 241305081 $$ 253565083 $$ 90025085 $$ 56445085 $$ 282435088 $$ 68145088 $$ 85175096 $$ 354355096 $$ 354365099 $$ 190785099 $$ 304865099 $$ 315315103 $$ 54075107 $$ 262795114 $$ 79975119 $$ 56135123 $$ 75935123 $$ 333695127 $$ 86725127 $$ 152445130 $$ 51475130 $$ 59075130 $$ 100055130 $$ 128645130 $$ 187605130 $$ 287645130 $$ 287655134 $$ 92045136 $$ 192445144 $$ 63225144 $$ 129025144 $$ 143315144 $$ 187385144 $$ 216115144 $$ 245785144 $$ 251605144 $$ 278435144 $$ 322835147 $$ 59075147 $$ 100055147 $$ 128655155 $$ 347715156 $$ 132865156 $$ 139495157 $$ 73845157 $$ 75855157 $$ 370165159 $$ 162225160 $$ 205695165 $$ 53315165 $$ 168885165 $$ 364585166 $$ 51675169 $$ 347395173 $$ 166375174 $$ 172425174 $$ 329135175 $$ 64885175 $$ 101745178 $$ 80435178 $$ 163675178 $$ 204605178 $$ 413985178 $$ 428995178 $$ 434815178 $$ 437045180 $$ 206145183 $$ 379965192 $$ 65055192 $$ 156845192 $$ 294485192 $$ 311255202 $$ 53735202 $$ 68065202 $$ 77685202 $$ 246145203 $$ 71235204 $$ 360095207 $$ 69295207 $$ 103695208 $$ 55375212 $$ 66795212 $$ 72885212 $$ 80805212 $$ 138195212 $$ 138205212 $$ 229455215 $$ 316295216 $$ 52185216 $$ 55545216 $$ 148205216 $$ 148215218 $$ 55545218 $$ 148205218 $$ 148215224 $$ 331745229 $$ 237805237 $$ 83655238 $$ 236705239 $$ 246965242 $$ 71335245 $$ 80135245 $$ 287225245 $$ 312775246 $$ 52505248 $$ 266025252 $$ 6668
