# Supplementary material for: The Exploration of Novel Regulatory Relationships Drives Haloarchaeal Operon-Like Structural Dynamics over Short Evolutionary Distances
Source: Microorganisms. 2020 Nov 30;8(12):1900. doi: 10.3390/microorganisms8121900 (PMC7760734; doi:10.3390/microorganisms8121900)
Supplement: Supplementary file 1 [file microorganisms-08-01900-s001.zip › S5_IdenticalContentGroupsQuerySet.rtf]

&&only 1 $$ 25 $$ 456 $$ 1746&&only 1 $$ 121 $$ 3438&&only 1 $$ 176 $$ 277&&only 1 $$ 277&&only 1 $$ 391&&only 1 $$ 456 $$ 1723 $$ 1746&&only 1 $$ 456 $$ 1746&&only 1 $$ 456 $$ 4303&&only 1 $$ 456 $$ 6143&&only 1 $$ 456 $$ 16996&&only 1 $$ 466&&only 1 $$ 513 $$ 1355 $$ 6698&&only 1 $$ 603&&only 1 $$ 660&&only 1 $$ 737&&only 1 $$ 788&&only 1 $$ 993&&only 1 $$ 1396 $$ 7062&&only 1 $$ 1396&&only 1 $$ 2202&&only 1 $$ 2214&&only 1 $$ 2337&&only 1 $$ 2349&&only 1 $$ 2462 $$ 2838&&only 1 $$ 2615&&only 1 $$ 3258&&only 1 $$ 5153&&only 2 $$ 2 $$ 3 $$ 11 $$ 1283 $$ 4718 $$ 4987&&only 2 $$ 2 $$ 183 $$ 1312&&only 2 $$ 2 $$ 183 $$ 1935 $$ 1993&&only 2 $$ 2 $$ 183 $$ 1935&&only 2 $$ 2 $$ 183 $$ 1935 $$ 1993 $$ 2905&&only 2 $$ 2 $$ 1283 $$ 1815 $$ 1829 $$ 7455&&only 2 $$ 2 $$ 1283 $$ 1829 $$ 2942&&only 2 $$ 2 $$ 1283 $$ 1829&&only 2 $$ 2 $$ 1283 $$ 4263 $$ 4487&&only 2 $$ 2 $$ 1283 $$ 4487&&only 2 $$ 2 $$ 1312&&only 2 $$ 2 $$ 1382 $$ 2056 $$ 2273&&only 2 $$ 2 $$ 1847 $$ 4486 $$ 4488&&only 2 $$ 2 $$ 1935 $$ 1993 $$ 10828&&only 2 $$ 2 $$ 1935&&only 2 $$ 2 $$ 1935 $$ 6802&&only 2 $$ 2 $$ 2056 $$ 2273&&only 2 $$ 2 $$ 2273&&only 2 $$ 2 $$ 2895 $$ 2896&&only 2 $$ 2 $$ 2895 $$ 2896 $$ 2897&&only 2 $$ 2 $$ 2895 $$ 2896 $$ 2897 $$ 4870&&only 2 $$ 2 $$ 2895&&only 2 $$ 2 $$ 2970 $$ 3146 $$ 3417&&only 2 $$ 2 $$ 2970 $$ 3146 $$ 3417 $$ 3534&&only 2 $$ 2 $$ 3029 $$ 3030 $$ 3109&&only 2 $$ 28 $$ 1312&&only 2 $$ 80 $$ 2309 $$ 2352 $$ 2572 $$ 10215&&only 2 $$ 80 $$ 2309 $$ 2352 $$ 2572&&only 2 $$ 967 $$ 1081&&only 2 $$ 1012 $$ 1656&&only 2 $$ 1053 $$ 2274&&only 2 $$ 1081&&only 2 $$ 1333 $$ 2119&&only 2 $$ 1333 $$ 2938&&only 2 $$ 1517 $$ 5925 $$ 13216&&only 2 $$ 1656&&only 2 $$ 1842 $$ 2020 $$ 10047&&only 2 $$ 1842&&only 2 $$ 1842 $$ 2020&&only 2 $$ 2119&&only 2 $$ 2274&&only 2 $$ 2309&&only 2 $$ 2309 $$ 2352 $$ 2572&&only 2 $$ 2446 $$ 2460 $$ 2586 $$ 14104&&only 2 $$ 2446 $$ 2460 $$ 2586&&only 2 $$ 2452 $$ 2467 $$ 2503&&only 2 $$ 2640 $$ 2641&&only 2 $$ 2824&&only 2 $$ 3036 $$ 3759&&only 2 $$ 3036&&only 2 $$ 3710&&only 2 $$ 3847&&only 2 $$ 4452&&only 2 $$ 4590&&only 2 $$ 4765 $$ 8242&&only 2 $$ 4765 $$ 10995&&only 3 $$ 7 $$ 57 $$ 198 $$ 279 $$ 5462&&only 3 $$ 20 $$ 2379&&only 3 $$ 229 $$ 331&&only 3 $$ 279&&only 3 $$ 282&&only 3 $$ 309 $$ 1315 $$ 1618 $$ 2415&&only 3 $$ 484&&only 3 $$ 822&&only 3 $$ 822 $$ 1251 $$ 13699&&only 3 $$ 1168&&only 3 $$ 1251&&only 3 $$ 1493&&only 3 $$ 1606 $$ 3202&&only 3 $$ 1630&&only 3 $$ 2171 $$ 2465&&only 3 $$ 3283&&only 3 $$ 4735 $$ 4927&&only 4 $$ 25 $$ 62 $$ 262 $$ 794&&only 4 $$ 61 $$ 2555&&only 4 $$ 62 $$ 262 $$ 794&&only 4 $$ 280&&only 4 $$ 894 $$ 1120&&only 4 $$ 894 $$ 1561&&only 4 $$ 894&&only 4 $$ 1120&&only 4 $$ 1138&&only 4 $$ 1403&&only 4 $$ 1403 $$ 3041&&only 4 $$ 2845&&only 4 $$ 3204&&only 5 $$ 69 $$ 1611&&only 5 $$ 298 $$ 14843&&only 5 $$ 3115&&only 6 $$ 21 $$ 1633 $$ 1634&&only 6 $$ 91 $$ 654&&only 6 $$ 121 $$ 391&&only 6 $$ 136 $$ 3035&&only 6 $$ 276 $$ 367 $$ 381 $$ 2034 $$ 12309&&only 6 $$ 372 $$ 381 $$ 2228&&only 6 $$ 372 $$ 381 $$ 1688&&only 6 $$ 381&&only 6 $$ 381 $$ 1673&&only 6 $$ 391&&only 6 $$ 391 $$ 391&&only 6 $$ 792 $$ 2451 $$ 2452 $$ 2467 $$ 2503&&only 6 $$ 1088 $$ 3977&&only 6 $$ 1633 $$ 1634&&only 6 $$ 1633&&only 6 $$ 1647&&only 6 $$ 1660&&only 6 $$ 2284&&only 6 $$ 2284 $$ 10316&&only 6 $$ 2451 $$ 2452 $$ 2467 $$ 2503 $$ 4527&&only 6 $$ 2451 $$ 2452 $$ 2503 $$ 4527 $$ 15229&&only 6 $$ 2452 $$ 2467 $$ 2503&&only 6 $$ 3035&&only 6 $$ 3977&&only 6 $$ 4283&&only 7 $$ 7 $$ 1183&&only 7 $$ 7 $$ 1183 $$ 1222&&only 7 $$ 57 $$ 155 $$ 375 $$ 418 $$ 2159&&only 7 $$ 57 $$ 418 $$ 538&&only 7 $$ 57 $$ 418 $$ 538 $$ 2159&&only 7 $$ 57 $$ 418 $$ 8482&&only 7 $$ 57 $$ 418&&only 7 $$ 57 $$ 499&&only 7 $$ 61 $$ 304 $$ 499 $$ 3136 $$ 4158&&only 7 $$ 279&&only 7 $$ 322 $$ 1257&&only 7 $$ 322 $$ 1257 $$ 14151&&only 7 $$ 322 $$ 1257 $$ 14490&&only 7 $$ 499 $$ 3136&&only 7 $$ 674 $$ 1183 $$ 1222&&only 7 $$ 1183 $$ 1222&&only 7 $$ 1257&&only 7 $$ 1267 $$ 2199 $$ 2224 $$ 2267&&only 7 $$ 1323 $$ 2199 $$ 2224 $$ 2267&&only 7 $$ 1571 $$ 2417&&only 7 $$ 1571 $$ 2527&&only 7 $$ 1898 $$ 2941 $$ 3003&&only 7 $$ 1898 $$ 3003&&only 7 $$ 1898 $$ 3343&&only 7 $$ 1898 $$ 5736 $$ 5833&&only 7 $$ 1898 $$ 5832&&only 7 $$ 1898 $$ 7444 $$ 8238&&only 7 $$ 2199 $$ 2224 $$ 2267&&only 7 $$ 2224 $$ 2267&&only 7 $$ 2446 $$ 2460 $$ 2586&&only 7 $$ 3003&&only 7 $$ 3729 $$ 3730&&only 8 $$ 464&&only 8 $$ 480&&only 8 $$ 798 $$ 966&&only 8 $$ 1263&&only 8 $$ 1609&&only 9 $$ 1311 $$ 2025&&only 10 $$ 242 $$ 1461&&only 10 $$ 650&&only 10 $$ 737&&only 10 $$ 863&&only 10 $$ 1804 $$ 1820 $$ 1828 $$ 1888 $$ 2052&&only 10 $$ 3632&&only 11 $$ 18 $$ 196 $$ 2897 $$ 9817&&only 11 $$ 544 $$ 563&&only 11 $$ 544 $$ 563 $$ 691&&only 11 $$ 544 $$ 563 $$ 1223&&only 11 $$ 1322&&only 11 $$ 1438&&only 11 $$ 1528&&only 11 $$ 2611 $$ 3591 $$ 4012&&only 11 $$ 2721&&only 11 $$ 2736&&only 12 $$ 13 $$ 13 $$ 31 $$ 306 $$ 4900 $$ 4901 $$ 4902 $$ 5216 $$ 5218 $$ 5554&&only 12 $$ 13 $$ 13 $$ 31 $$ 413&&only 12 $$ 13 $$ 13 $$ 31 $$ 1853&&only 12 $$ 13 $$ 13 $$ 31 $$ 2211 $$ 2211 $$ 2211 $$ 2535 $$ 4888&&only 12 $$ 13 $$ 13 $$ 31 $$ 4913&&only 12 $$ 13 $$ 13 $$ 1552&&only 12 $$ 13 $$ 13 $$ 1552 $$ 2362&&only 12 $$ 13 $$ 31 $$ 37 $$ 2904&&only 12 $$ 13 $$ 31 $$ 45 $$ 1853&&only 12 $$ 13 $$ 31 $$ 45 $$ 2602&&only 12 $$ 13 $$ 31 $$ 45 $$ 2602 $$ 2757&&only 12 $$ 13 $$ 31 $$ 45 $$ 2602 $$ 6733&&only 12 $$ 13 $$ 31 $$ 45 $$ 2757&&only 12 $$ 13 $$ 45 $$ 589 $$ 1574&&only 12 $$ 13 $$ 45 $$ 803 $$ 804 $$ 970 $$ 1678 $$ 1684 $$ 4345&&only 12 $$ 13 $$ 45 $$ 1574&&only 12 $$ 13 $$ 45 $$ 1574 $$ 6733&&only 12 $$ 13 $$ 45 $$ 1678 $$ 1684 $$ 4345&&only 12 $$ 13 $$ 45 $$ 1678&&only 12 $$ 13 $$ 45 $$ 1678 $$ 1684 $$ 7474&&only 12 $$ 13 $$ 45 $$ 1678 $$ 1684 $$ 3631&&only 12 $$ 13 $$ 45 $$ 1678 $$ 3631&&only 12 $$ 13 $$ 45 $$ 1678 $$ 3800&&only 12 $$ 13 $$ 45 $$ 1678 $$ 4345&&only 12 $$ 13 $$ 45 $$ 1826 $$ 2133&&only 12 $$ 13 $$ 45 $$ 1826&&only 12 $$ 13 $$ 306 $$ 4900 $$ 4901 $$ 4902 $$ 5216 $$ 5218 $$ 14820 $$ 14821&&only 12 $$ 13 $$ 1574&&only 12 $$ 31 $$ 94 $$ 103 $$ 2837 $$ 11290&&only 12 $$ 31 $$ 103 $$ 1338&&only 12 $$ 31 $$ 103 $$ 1365&&only 12 $$ 31 $$ 103 $$ 2756&&only 12 $$ 31 $$ 103 $$ 2837&&only 12 $$ 31 $$ 103 $$ 2904&&only 12 $$ 31 $$ 1338 $$ 2154&&only 12 $$ 31 $$ 1338&&only 12 $$ 31 $$ 1877&&only 12 $$ 31 $$ 2154&&only 12 $$ 31 $$ 2904 $$ 14893&&only 12 $$ 103 $$ 1552 $$ 2362&&only 12 $$ 103 $$ 1552&&only 12 $$ 589&&only 12 $$ 1338&&only 12 $$ 1552&&only 12 $$ 1552 $$ 2362&&only 12 $$ 1574&&only 12 $$ 1826&&only 12 $$ 1853&&only 12 $$ 2362&&only 12 $$ 2756 $$ 16483&&only 12 $$ 2904&&only 13 $$ 45 $$ 1574&&only 13 $$ 45 $$ 1678&&only 13 $$ 45 $$ 1826&&only 13 $$ 45 $$ 1826 $$ 6718&&only 13 $$ 45 $$ 1826 $$ 15688&&only 13 $$ 1574&&only 14 $$ 350&&only 15 $$ 15 $$ 309&&only 15 $$ 235 $$ 309 $$ 364&&only 15 $$ 235 $$ 364&&only 15 $$ 309&&only 15 $$ 413&&only 15 $$ 413 $$ 1632 $$ 4254 $$ 7993&&only 15 $$ 1632&&only 15 $$ 1632 $$ 1832&&only 15 $$ 1774&&only 15 $$ 1832&&only 15 $$ 4254&&only 16 $$ 263 $$ 405&&only 16 $$ 263 $$ 2129&&only 16 $$ 263 $$ 2228 $$ 13297&&only 16 $$ 3462 $$ 3463&&only 16 $$ 3463&&only 16 $$ 3962 $$ 6087 $$ 6693&&only 16 $$ 4120&&only 17 $$ 1240&&only 17 $$ 2079 $$ 12416&&only 17 $$ 2473&&only 17 $$ 3715&&only 18 $$ 888&&only 18 $$ 904&&only 18 $$ 1185&&only 18 $$ 2286&&only 19 $$ 158 $$ 684 $$ 685&&only 19 $$ 310 $$ 524&&only 19 $$ 2487&&only 20 $$ 20 $$ 429 $$ 1276&&only 20 $$ 26 $$ 100 $$ 1636&&only 20 $$ 81 $$ 414 $$ 1169&&only 20 $$ 100 $$ 1726&&only 20 $$ 1355&&only 20 $$ 1481&&only 20 $$ 1595&&only 20 $$ 1887&&only 20 $$ 2548&&only 20 $$ 3628&&only 21 $$ 54 $$ 129 $$ 1968 $$ 2142 $$ 2243&&only 21 $$ 213 $$ 3285&&only 21 $$ 1677 $$ 3102&&only 21 $$ 1677&&only 21 $$ 1677 $$ 7869&&only 21 $$ 3915&&only 22 $$ 1113&&only 23 $$ 1033&&only 23 $$ 2146&&only 23 $$ 3281&&only 24 $$ 430&&only 24 $$ 818 $$ 6033 $$ 7407&&only 24 $$ 1593&&only 24 $$ 1593 $$ 1876&&only 24 $$ 2158&&only 24 $$ 2200 $$ 2328 $$ 2539&&only 24 $$ 2328 $$ 2539&&only 24 $$ 2328 $$ 2539 $$ 4896&&only 24 $$ 3075&&only 25 $$ 869&&only 25 $$ 1423&&only 25 $$ 1849&&only 26 $$ 333 $$ 1138&&only 26 $$ 333&&only 26 $$ 333 $$ 8083&&only 26 $$ 333 $$ 14673&&only 26 $$ 358&&only 26 $$ 358 $$ 5215&&only 26 $$ 1636&&only 26 $$ 5084&&only 27 $$ 941 $$ 1204 $$ 4212&&only 27 $$ 1018&&only 27 $$ 1018 $$ 1525&&only 27 $$ 1018 $$ 2182&&only 27 $$ 1018 $$ 8005&&only 27 $$ 1139 $$ 1942 $$ 1975 $$ 1976 $$ 2003 $$ 8145 $$ 8223 $$ 12317 $$ 12318&&only 27 $$ 1139&&only 27 $$ 3418&&only 27 $$ 4212&&only 28 $$ 83 $$ 116 $$ 233 $$ 276 $$ 367 $$ 2034&&only 28 $$ 83 $$ 116 $$ 276 $$ 367 $$ 2034&&only 28 $$ 83 $$ 116 $$ 1315 $$ 1624 $$ 5378&&only 28 $$ 83 $$ 116 $$ 1545 $$ 1624 $$ 1827&&only 28 $$ 83 $$ 116 $$ 1624 $$ 1827&&only 28 $$ 116 $$ 233 $$ 367 $$ 1775&&only 28 $$ 116 $$ 276 $$ 367 $$ 2034&&only 28 $$ 116 $$ 367 $$ 1775&&only 28 $$ 116 $$ 1545 $$ 1624 $$ 1827&&only 28 $$ 116 $$ 1624 $$ 1827&&only 28 $$ 116 $$ 1624 $$ 5378&&only 28 $$ 238 $$ 2299 $$ 2300 $$ 2301&&only 28 $$ 276 $$ 367 $$ 1775&&only 28 $$ 276 $$ 367 $$ 2034&&only 28 $$ 276 $$ 367 $$ 2034 $$ 9428&&only 28 $$ 367 $$ 1775&&only 28 $$ 572 $$ 1384&&only 28 $$ 572 $$ 1384 $$ 1901&&only 28 $$ 1384&&only 28 $$ 1545 $$ 1624 $$ 1827&&only 28 $$ 1624 $$ 1827&&only 28 $$ 2299 $$ 2300 $$ 2301&&only 28 $$ 2301&&only 28 $$ 2640 $$ 2641&&only 28 $$ 2641&&only 28 $$ 3992 $$ 3993 $$ 4137&&only 29 $$ 147 $$ 1313 $$ 1314&&only 29 $$ 1313 $$ 1314&&only 29 $$ 1314&&only 29 $$ 1474 $$ 2219&&only 29 $$ 1474 $$ 2219 $$ 14173&&only 29 $$ 2219&&only 29 $$ 2576 $$ 2577 $$ 2656 $$ 5103 $$ 5407&&only 29 $$ 2576 $$ 2577 $$ 2656 $$ 3241 $$ 3344&&only 29 $$ 2576 $$ 3344&&only 29 $$ 2577 $$ 2656 $$ 5103 $$ 5407&&only 30 $$ 632 $$ 848&&only 30 $$ 632 $$ 848 $$ 2311&&only 30 $$ 632 $$ 2311&&only 30 $$ 632&&only 30 $$ 795&&only 30 $$ 1825&&only 30 $$ 2311&&only 32 $$ 350&&only 32 $$ 1508&&only 32 $$ 1540&&only 32 $$ 1939&&only 32 $$ 2253&&only 32 $$ 2330&&only 33 $$ 582&&only 33 $$ 582 $$ 10840&&only 33 $$ 1511&&only 33 $$ 2304&&only 33 $$ 2862 $$ 3840&&only 34 $$ 416&&only 34 $$ 1625&&only 34 $$ 2069&&only 34 $$ 2069 $$ 8012&&only 34 $$ 2105&&only 35 $$ 3227&&only 36 $$ 1788&&only 37 $$ 210 $$ 1395&&only 37 $$ 317&&only 37 $$ 1959&&only 37 $$ 2749&&only 37 $$ 3206&&only 38 $$ 63 $$ 273 $$ 274&&only 38 $$ 273 $$ 274&&only 38 $$ 274&&only 38 $$ 353&&only 38 $$ 384 $$ 7296&&only 38 $$ 2356&&only 39 $$ 536&&only 39 $$ 565 $$ 566 $$ 795 $$ 964&&only 39 $$ 2828&&only 39 $$ 4286&&only 39 $$ 4729&&only 40 $$ 278&&only 40 $$ 430&&only 40 $$ 1782&&only 40 $$ 2031&&only 41 $$ 537&&only 42 $$ 315 $$ 785 $$ 786&&only 42 $$ 350&&only 42 $$ 1961 $$ 2023 $$ 2057 $$ 2089 $$ 2131&&only 42 $$ 1961&&only 42 $$ 1961 $$ 2023&&only 42 $$ 3752&&only 43 $$ 859 $$ 1011 $$ 1608 $$ 1641 $$ 2390&&only 43 $$ 2037&&only 43 $$ 2153&&only 43 $$ 2390&&only 43 $$ 2453&&only 43 $$ 3058&&only 43 $$ 3602&&only 44 $$ 571 $$ 602&&only 44 $$ 602&&only 44 $$ 664&&only 44 $$ 664 $$ 7846&&only 44 $$ 664 $$ 14353 $$ 14354&&only 44 $$ 1111&&only 44 $$ 1213&&only 44 $$ 1235&&only 46 $$ 87 $$ 121 $$ 1420&&only 46 $$ 121 $$ 2027&&only 46 $$ 2027&&only 46 $$ 2027 $$ 13898&&only 47 $$ 564&&only 47 $$ 3327&&only 47 $$ 3327 $$ 3599 $$ 6158 $$ 6197 $$ 6198&&only 47 $$ 3969&&only 47 $$ 5027&&only 48 $$ 304 $$ 1035 $$ 1265 $$ 15749&&only 48 $$ 314 $$ 323 $$ 1035 $$ 1134 $$ 1265 $$ 3052&&only 48 $$ 314 $$ 323 $$ 1035 $$ 1134 $$ 1265 $$ 4679&&only 48 $$ 314 $$ 1035 $$ 1134 $$ 1265 $$ 4747&&only 48 $$ 314 $$ 1035 $$ 1134 $$ 1265 $$ 5161&&only 48 $$ 314 $$ 1035 $$ 1134 $$ 1265 $$ 3052&&only 48 $$ 389&&only 48 $$ 1035&&only 48 $$ 1035 $$ 1265&&only 48 $$ 1965&&only 48 $$ 2227&&only 48 $$ 2697&&only 49 $$ 86 $$ 1442&&only 49 $$ 279&&only 49 $$ 790&&only 49 $$ 2369&&only 50 $$ 276 $$ 873 $$ 2106 $$ 3791&&only 50 $$ 1998&&only 50 $$ 2649&&only 50 $$ 3791&&only 51 $$ 1844&&only 51 $$ 3500 $$ 3501&&only 51 $$ 3501&&only 51 $$ 3501 $$ 15400&&only 52 $$ 434&&only 52 $$ 1850 $$ 5005 $$ 5720 $$ 6106 $$ 8218&&only 53 $$ 53 $$ 332&&only 53 $$ 316&&only 53 $$ 332&&only 53 $$ 1111&&only 54 $$ 1906&&only 54 $$ 2243&&only 54 $$ 2331&&only 55 $$ 68 $$ 72 $$ 216 $$ 796&&only 55 $$ 68 $$ 2827&&only 55 $$ 1559&&only 55 $$ 5262&&only 56 $$ 76 $$ 945 $$ 1142&&only 57 $$ 418 $$ 538&&only 57 $$ 499&&only 57 $$ 499 $$ 2749 $$ 3162 $$ 3341&&only 57 $$ 499 $$ 3162 $$ 3341&&only 57 $$ 499 $$ 3162&&only 57 $$ 499 $$ 9157&&only 57 $$ 2749 $$ 3341&&only 58 $$ 1330&&only 58 $$ 1673&&only 58 $$ 1912 $$ 5729&&only 58 $$ 2853 $$ 11835&&only 59 $$ 69 $$ 3037&&only 59 $$ 69 $$ 3714&&only 59 $$ 375&&only 59 $$ 1300 $$ 2598&&only 59 $$ 2936 $$ 2946&&only 59 $$ 3037&&only 59 $$ 3932&&only 59 $$ 4242&&only 60 $$ 2496&&only 60 $$ 2661&&only 60 $$ 2661 $$ 6541 $$ 8510&&only 60 $$ 2727&&only 60 $$ 2727 $$ 11064&&only 60 $$ 2855&&only 60 $$ 3606 $$ 13893 $$ 15022&&only 60 $$ 3606 $$ 15860&&only 61 $$ 180 $$ 201 $$ 304 $$ 4730&&only 61 $$ 200 $$ 314 $$ 3390 $$ 3427 $$ 3530 $$ 14143&&only 61 $$ 275&&only 61 $$ 304&&only 61 $$ 314 $$ 3390 $$ 3530&&only 61 $$ 1160 $$ 2555 $$ 5036 $$ 17017&&only 61 $$ 1598&&only 61 $$ 3390&&only 61 $$ 3437&&only 62 $$ 262 $$ 1114 $$ 2618&&only 62 $$ 265 $$ 1462&&only 62 $$ 2618&&only 62 $$ 3629&&only 62 $$ 4387&&only 62 $$ 5120&&only 62 $$ 5176&&only 63 $$ 273&&only 63 $$ 2877&&only 63 $$ 3567&&only 63 $$ 4181 $$ 4669&&only 64 $$ 1282 $$ 1282 $$ 2794&&only 64 $$ 1282 $$ 2794&&only 64 $$ 1282 $$ 3626&&only 64 $$ 1282 $$ 3627&&only 64 $$ 1282 $$ 9866&&only 64 $$ 1620 $$ 1655&&only 64 $$ 1655&&only 64 $$ 1716&&only 64 $$ 2762&&only 64 $$ 2762 $$ 2762 $$ 3124&&only 64 $$ 3627&&only 65 $$ 65 $$ 1605&&only 65 $$ 244 $$ 1598&&only 65 $$ 346 $$ 3528 $$ 8106&&only 65 $$ 2767&&only 65 $$ 3446&&only 65 $$ 3528&&only 65 $$ 3528 $$ 6517&&only 66 $$ 2298&&only 66 $$ 3458&&only 66 $$ 4002&&only 67 $$ 2100&&only 67 $$ 2484 $$ 2485&&only 67 $$ 2484 $$ 13347&&only 67 $$ 2906 $$ 11901&&only 68 $$ 2407 $$ 2466&&only 68 $$ 2407&&only 68 $$ 2407 $$ 2466 $$ 6659&&only 68 $$ 2827&&only 69 $$ 1611&&only 69 $$ 2286&&only 69 $$ 3037&&only 69 $$ 3714&&only 70 $$ 934 $$ 2520&&only 70 $$ 2520 $$ 2709&&only 70 $$ 2520&&only 71 $$ 1685&&only 72 $$ 4743&&only 73 $$ 3144&&only 74 $$ 298 $$ 3202&&only 74 $$ 812&&only 74 $$ 2751&&only 75 $$ 526&&only 75 $$ 3423&&only 76 $$ 506 $$ 1049 $$ 6017&&only 76 $$ 945 $$ 1142&&only 76 $$ 1142&&only 77 $$ 1801 $$ 1823 $$ 1893&&only 78 $$ 1090&&only 79 $$ 1576&&only 79 $$ 2988&&only 80 $$ 389 $$ 1159&&only 80 $$ 1228 $$ 15611&&only 80 $$ 1913&&only 81 $$ 414 $$ 1169&&only 81 $$ 414&&only 81 $$ 414 $$ 1169 $$ 5039&&only 81 $$ 414 $$ 1169 $$ 2846&&only 81 $$ 606 $$ 612 $$ 613&&only 81 $$ 612&&only 81 $$ 1169&&only 82 $$ 2347&&only 83 $$ 490 $$ 2282&&only 83 $$ 1454 $$ 4759&&only 84 $$ 2368&&only 86 $$ 557 $$ 1063 $$ 1075 $$ 1150 $$ 1748&&only 86 $$ 557 $$ 1063 $$ 1075 $$ 1150 $$ 1303 $$ 1748&&only 86 $$ 557 $$ 1063 $$ 1075 $$ 1150 $$ 1748 $$ 3917&&only 86 $$ 1063&&only 86 $$ 1063 $$ 1150&&only 86 $$ 1063 $$ 1150 $$ 11615&&only 86 $$ 1380 $$ 2280 $$ 3308 $$ 3309&&only 86 $$ 1560&&only 86 $$ 2368&&only 87 $$ 943 $$ 1420&&only 87 $$ 943 $$ 6596&&only 87 $$ 1420&&only 88 $$ 362 $$ 1566 $$ 1567 $$ 1586 $$ 3077 $$ 7378&&only 88 $$ 631&&only 88 $$ 1239&&only 89 $$ 905 $$ 15618&&only 89 $$ 1188&&only 90 $$ 298 $$ 6572&&only 90 $$ 1064&&only 92 $$ 470&&only 92 $$ 502&&only 93 $$ 736 $$ 910 $$ 1043&&only 93 $$ 801&&only 94 $$ 4143 $$ 11164&&only 95 $$ 2382&&only 96 $$ 141 $$ 452 $$ 476 $$ 531 $$ 738 $$ 739 $$ 740 $$ 914 $$ 1229 $$ 1230 $$ 2343&&only 96 $$ 334 $$ 1097&&only 96 $$ 334 $$ 5036&&only 96 $$ 334&&only 96 $$ 374&&only 97 $$ 1742 $$ 12290&&only 97 $$ 2702&&only 98 $$ 769 $$ 776&&only 99 $$ 1577&&only 99 $$ 5014&&only 100 $$ 2944&&only 101 $$ 1198&&only 103 $$ 1338&&only 103 $$ 1552&&only 104 $$ 199 $$ 3325 $$ 12391&&only 104 $$ 199 $$ 3928&&only 105 $$ 197 $$ 406 $$ 732 $$ 5329 $$ 12288&&only 105 $$ 2667&&only 105 $$ 4861&&only 106 $$ 1692 $$ 7606 $$ 10090 $$ 13253&&only 106 $$ 3323&&only 106 $$ 3324&&only 106 $$ 3349 $$ 15017&&only 106 $$ 4017&&only 106 $$ 4830 $$ 4831&&only 107 $$ 3198&&only 107 $$ 3964 $$ 6672&&only 108 $$ 4300&&only 108 $$ 4567&&only 109 $$ 220 $$ 3058&&only 109 $$ 977&&only 109 $$ 1763 $$ 8171&&only 110 $$ 1119&&only 110 $$ 1689&&only 110 $$ 2863&&only 111 $$ 295 $$ 2182&&only 111 $$ 2182&&only 111 $$ 2775&&only 111 $$ 3152&&only 111 $$ 3425 $$ 5417&&only 113 $$ 392 $$ 2291&&only 113 $$ 1649 $$ 1650&&only 114 $$ 555&&only 115 $$ 830&&only 117 $$ 841 $$ 12015&&only 117 $$ 841&&only 118 $$ 3535&&only 120 $$ 1611 $$ 5549&&only 120 $$ 2134&&only 121 $$ 3438&&only 122 $$ 1016 $$ 1394&&only 122 $$ 1394&&only 122 $$ 1394 $$ 2102&&only 122 $$ 1394 $$ 14339&&only 122 $$ 2983&&only 123 $$ 734&&only 123 $$ 734 $$ 1228&&only 126 $$ 306&&only 126 $$ 609 $$ 2524&&only 126 $$ 724 $$ 895&&only 127 $$ 796&&only 128 $$ 1870&&only 128 $$ 4584&&only 129 $$ 1730&&only 129 $$ 4073 $$ 4405&&only 130 $$ 258 $$ 818 $$ 1450 $$ 1554&&only 130 $$ 258 $$ 818 $$ 1034 $$ 1450 $$ 1554 $$ 1882&&only 130 $$ 818 $$ 1034 $$ 1450 $$ 1554 $$ 1882&&only 130 $$ 1034&&only 130 $$ 1034 $$ 1709 $$ 4525&&only 130 $$ 258 $$ 1034&&only 130 $$ 165 $$ 1387 $$ 2132&&only 130 $$ 1446&&only 130 $$ 1450&&only 130 $$ 258 $$ 1450&&only 130 $$ 130 $$ 165 $$ 1554 $$ 14402&&only 130 $$ 130 $$ 165 $$ 1554 $$ 14512&&only 130 $$ 3339 $$ 7328&&only 131 $$ 451&&only 132 $$ 419 $$ 878 $$ 1030&&only 132 $$ 419 $$ 683 $$ 878 $$ 1030 $$ 1125&&only 132 $$ 419 $$ 1030&&only 132 $$ 419 $$ 878 $$ 1029 $$ 1030&&only 132 $$ 444&&only 132 $$ 1029 $$ 1030&&only 132 $$ 1030&&only 134 $$ 332&&only 134 $$ 1269&&only 135 $$ 2244&&only 136 $$ 769&&only 136 $$ 838 $$ 12006&&only 136 $$ 1524&&only 136 $$ 2512&&only 137 $$ 2869&&only 140 $$ 505&&only 141 $$ 452 $$ 476 $$ 914 $$ 1230&&only 141 $$ 452 $$ 1230&&only 141 $$ 452 $$ 476 $$ 531 $$ 738 $$ 739 $$ 740 $$ 914 $$ 1229 $$ 1230 $$ 2343&&only 141 $$ 452 $$ 476 $$ 914 $$ 7364&&only 141 $$ 452 $$ 476 $$ 531 $$ 738 $$ 739 $$ 740 $$ 914 $$ 1229 $$ 1230 $$ 3746&&only 141 $$ 1377&&only 142 $$ 143 $$ 1682&&only 144 $$ 1815&&only 144 $$ 3612 $$ 12676&&only 144 $$ 3612 $$ 5796&&only 145 $$ 719&&only 145 $$ 756 $$ 12353 $$ 13119&&only 145 $$ 933&&only 146 $$ 1300&&only 146 $$ 1300 $$ 10815&&only 146 $$ 146 $$ 1852 $$ 5535 $$ 7055&&only 146 $$ 146 $$ 1852 $$ 5535 $$ 9727&&only 146 $$ 4603&&only 147 $$ 1223&&only 148 $$ 1000&&only 148 $$ 1324&&only 148 $$ 1454&&only 149 $$ 1040 $$ 15609&&only 149 $$ 2792&&only 150 $$ 1387&&only 150 $$ 162 $$ 3043&&only 151 $$ 1714&&only 153 $$ 1252&&only 153 $$ 2701&&only 153 $$ 4904&&only 154 $$ 1224&&only 154 $$ 1756 $$ 2950&&only 154 $$ 2950&&only 154 $$ 5003 $$ 5004&&only 155 $$ 2142&&only 157 $$ 2074&&only 158 $$ 684 $$ 685&&only 158 $$ 1317&&only 158 $$ 5203&&only 159 $$ 512&&only 159 $$ 512 $$ 8686&&only 159 $$ 159 $$ 512&&only 159 $$ 4650 $$ 5595&&only 159 $$ 159 $$ 4650&&only 160 $$ 295&&only 161 $$ 1104&&only 162 $$ 357&&only 162 $$ 1446 $$ 15825 $$ 15826&&only 162 $$ 2132 $$ 9481 $$ 11872 $$ 11873&&only 162 $$ 2399 $$ 2925&&only 162 $$ 2693&&only 162 $$ 4732 $$ 4822&&only 163 $$ 1041&&only 164 $$ 249 $$ 284 $$ 1401&&only 164 $$ 249 $$ 1401&&only 164 $$ 1401&&only 164 $$ 1859&&only 164 $$ 249 $$ 1859&&only 165 $$ 1034&&only 165 $$ 1387 $$ 2132&&only 165 $$ 1387&&only 165 $$ 258 $$ 1882&&only 166 $$ 380 $$ 4122&&only 166 $$ 380&&only 166 $$ 562&&only 166 $$ 1425&&only 166 $$ 3593&&only 166 $$ 4122&&only 167 $$ 513&&only 167 $$ 1355&&only 168 $$ 289&&only 170 $$ 1868 $$ 1931&&only 171 $$ 513&&only 172 $$ 1936 $$ 4619&&only 172 $$ 4619&&only 173 $$ 701 $$ 702 $$ 4174&&only 173 $$ 1284 $$ 2698&&only 173 $$ 1651 $$ 2861 $$ 8337 $$ 8391 $$ 15404 $$ 15405&&only 173 $$ 1663&&only 173 $$ 2698&&only 173 $$ 3383&&only 173 $$ 4174&&only 174 $$ 2025&&only 174 $$ 2152&&only 174 $$ 2344&&only 175 $$ 977 $$ 1170 $$ 11902&&only 176 $$ 277 $$ 8363&&only 176 $$ 277&&only 176 $$ 894&&only 177 $$ 474 $$ 774 $$ 775&&only 177 $$ 774 $$ 775&&only 178 $$ 339 $$ 1751 $$ 1760&&only 178 $$ 1863&&only 179 $$ 1929&&only 180 $$ 201 $$ 607&&only 180 $$ 201 $$ 1512 $$ 10235&&only 180 $$ 201 $$ 1724 $$ 1759&&only 180 $$ 201 $$ 1759 $$ 2482&&only 180 $$ 201 $$ 1759&&only 180 $$ 201 $$ 2482&&only 180 $$ 201 $$ 4730&&only 181 $$ 208 $$ 344&&only 181 $$ 344 $$ 2962&&only 181 $$ 344&&only 181 $$ 208 $$ 344 $$ 5240&&only 182 $$ 287&&only 183 $$ 1993&&only 184 $$ 1079&&only 184 $$ 1901&&only 184 $$ 3089&&only 184 $$ 3512&&only 185 $$ 521 $$ 963 $$ 975 $$ 1089 $$ 1165&&only 185 $$ 521 $$ 963 $$ 975 $$ 1089 $$ 1165 $$ 2246&&only 185 $$ 521 $$ 963 $$ 1165&&only 185 $$ 521 $$ 963 $$ 17448&&only 185 $$ 963 $$ 1165&&only 185 $$ 963 $$ 975 $$ 1089 $$ 1165 $$ 2218&&only 185 $$ 963 $$ 975 $$ 1089 $$ 1165&&only 185 $$ 963 $$ 975 $$ 1089 $$ 1165 $$ 2246&&only 186 $$ 1691&&only 186 $$ 4772&&only 187 $$ 3928&&only 189 $$ 928&&only 189 $$ 3870&&only 192 $$ 433 $$ 713 $$ 714&&only 192 $$ 433&&only 193 $$ 1762&&only 193 $$ 4018&&only 194 $$ 880&&only 195 $$ 493&&only 196 $$ 798&&only 196 $$ 815&&only 196 $$ 2697&&only 197 $$ 732&&only 197 $$ 1765&&only 198 $$ 1727 $$ 1739&&only 198 $$ 1739&&only 198 $$ 3335 $$ 3433 $$ 3492&&only 198 $$ 3335 $$ 3433&&only 198 $$ 3335&&only 198 $$ 3433 $$ 3492&&only 199 $$ 1802&&only 200 $$ 787&&only 200 $$ 3427&&only 202 $$ 407&&only 202 $$ 1669 $$ 4039&&only 202 $$ 3485&&only 203 $$ 673 $$ 867&&only 203 $$ 2434&&only 203 $$ 3006 $$ 13206&&only 204 $$ 609 $$ 830&&only 204 $$ 830&&only 204 $$ 3503&&only 205 $$ 1436&&only 206 $$ 1769 $$ 4076 $$ 5423 $$ 5424&&only 206 $$ 2498 $$ 2952 $$ 3195&&only 206 $$ 2498 $$ 9787 $$ 15721&&only 206 $$ 2952 $$ 13107 $$ 13108&&only 206 $$ 3263 $$ 5800 $$ 6797&&only 206 $$ 3648 $$ 5800 $$ 6797&&only 206 $$ 3648 $$ 4397 $$ 4610&&only 206 $$ 4076 $$ 5423 $$ 5424&&only 206 $$ 4076 $$ 15357 $$ 15358&&only 206 $$ 4397 $$ 4610&&only 206 $$ 4586 $$ 6892 $$ 6893&&only 206 $$ 4586 $$ 6894 $$ 7498&&only 207 $$ 2046&&only 208 $$ 974&&only 209 $$ 607 $$ 6221&&only 209 $$ 607 $$ 6221 $$ 12764&&only 209 $$ 747 $$ 924&&only 209 $$ 1143&&only 210 $$ 651 $$ 1211 $$ 1395 $$ 2185 $$ 2420 $$ 12119&&only 210 $$ 728&&only 210 $$ 1395 $$ 4739&&only 210 $$ 1395&&only 211 $$ 753 $$ 1366&&only 211 $$ 753 $$ 1366 $$ 7395&&only 211 $$ 753&&only 211 $$ 753 $$ 13110&&only 211 $$ 753 $$ 1366 $$ 3604&&only 211 $$ 1366&&only 214 $$ 720&&only 214 $$ 1525&&only 214 $$ 1982&&only 215 $$ 581&&only 215 $$ 581 $$ 1682 $$ 6214&&only 215 $$ 581 $$ 2247&&only 218 $$ 253 $$ 852&&only 218 $$ 852&&only 218 $$ 852 $$ 4277&&only 218 $$ 1929&&only 219 $$ 840 $$ 853 $$ 1004&&only 219 $$ 853 $$ 1004&&only 219 $$ 1004&&only 219 $$ 2781&&only 220 $$ 1235&&only 220 $$ 2453&&only 220 $$ 3058&&only 220 $$ 4077&&only 222 $$ 286 $$ 545 $$ 1301 $$ 12242&&only 222 $$ 922&&only 224 $$ 2597 $$ 4600&&only 224 $$ 4600&&only 225 $$ 968&&only 226 $$ 341 $$ 443&&only 229 $$ 331 $$ 5681&&only 229 $$ 331&&only 229 $$ 331 $$ 4114&&only 229 $$ 331 $$ 332&&only 229 $$ 726 $$ 12237&&only 230 $$ 3317&&only 230 $$ 4820&&only 231 $$ 720&&only 233 $$ 1478 $$ 1788&&only 233 $$ 1478&&only 235 $$ 309 $$ 364&&only 235 $$ 309 $$ 309 $$ 364&&only 235 $$ 364&&only 235 $$ 364 $$ 15414&&only 236 $$ 357 $$ 1322&&only 236 $$ 477 $$ 915 $$ 1997&&only 237 $$ 1029&&only 237 $$ 3741&&only 239 $$ 857&&only 239 $$ 1549&&only 239 $$ 1668 $$ 2265 $$ 12412&&only 241 $$ 1232&&only 241 $$ 2353&&only 242 $$ 487 $$ 14936&&only 242 $$ 1461&&only 242 $$ 3814&&only 243 $$ 337 $$ 383 $$ 959&&only 243 $$ 959&&only 244 $$ 352&&only 244 $$ 449 $$ 741&&only 245 $$ 1512 $$ 1555 $$ 1690&&only 245 $$ 1555&&only 246 $$ 328&&only 246 $$ 1340&&only 247 $$ 991&&only 247 $$ 2222&&only 248 $$ 348 $$ 1117 $$ 1809&&only 248 $$ 348&&only 250 $$ 271 $$ 481 $$ 510&&only 250 $$ 481 $$ 510&&only 250 $$ 481 $$ 510 $$ 909&&only 250 $$ 4019 $$ 4049&&only 250 $$ 4765&&only 250 $$ 4765 $$ 9125&&only 252 $$ 422&&only 252 $$ 605&&only 252 $$ 2259&&only 252 $$ 4611&&only 253 $$ 648&&only 254 $$ 1589 $$ 5447&&only 254 $$ 1864 $$ 2335 $$ 2843 $$ 3053 $$ 4528 $$ 12286&&only 254 $$ 1864 $$ 2335 $$ 2843 $$ 3222 $$ 3321 $$ 3443&&only 254 $$ 1864 $$ 3222 $$ 3321 $$ 3443&&only 254 $$ 1864 $$ 2335 $$ 2843 $$ 3321 $$ 3443&&only 254 $$ 1864 $$ 3222 $$ 3321 $$ 3442 $$ 9657 $$ 9808&&only 254 $$ 1864 $$ 4451 $$ 4528 $$ 4749 $$ 5032 $$ 5033&&only 254 $$ 2380 $$ 2439 $$ 2478 $$ 2552 $$ 2814 $$ 4880&&only 254 $$ 2380 $$ 2439 $$ 2552 $$ 3222 $$ 9836 $$ 14316&&only 254 $$ 2380 $$ 2552 $$ 2814&&only 254 $$ 2380 $$ 2439 $$ 2478 $$ 2552 $$ 2814 $$ 5194&&only 254 $$ 2380 $$ 2552 $$ 2814 $$ 11606&&only 254 $$ 2814&&only 254 $$ 3053&&only 254 $$ 3053 $$ 10265&&only 254 $$ 4140&&only 254 $$ 4451&&only 255 $$ 1486&&only 255 $$ 1486 $$ 2396&&only 255 $$ 1486 $$ 3884&&only 255 $$ 2371&&only 258 $$ 1034 $$ 1882&&only 258 $$ 2760&&only 259 $$ 5060 $$ 6215&&only 260 $$ 912 $$ 13167&&only 260 $$ 912&&only 264 $$ 529&&only 264 $$ 776&&only 264 $$ 1216&&only 264 $$ 3126&&only 265 $$ 389 $$ 1462&&only 265 $$ 1462&&only 267 $$ 323&&only 268 $$ 1968&&only 269 $$ 282&&only 269 $$ 1950&&only 269 $$ 3465&&only 270 $$ 354&&only 270 $$ 2112 $$ 4970&&only 271 $$ 908 $$ 1161&&only 272 $$ 15285&&only 273 $$ 274&&only 275 $$ 323&&only 275 $$ 416&&only 276 $$ 367 $$ 2034&&only 276 $$ 873 $$ 2106&&only 277 $$ 2187&&only 278 $$ 1529 $$ 9601 $$ 12428&&only 278 $$ 1661 $$ 6640 $$ 9182&&only 278 $$ 4061&&only 280 $$ 385 $$ 986&&only 281 $$ 891&&only 281 $$ 1785&&only 281 $$ 10700&&only 282 $$ 1106&&only 282 $$ 3140&&only 283 $$ 923&&only 283 $$ 923 $$ 1379&&only 283 $$ 923 $$ 1379 $$ 7819&&only 283 $$ 13127&&only 284 $$ 995 $$ 996 $$ 1562 $$ 9149&&only 284 $$ 1562&&only 285 $$ 1147&&only 286 $$ 6377&&only 287 $$ 1086&&only 287 $$ 2112&&only 287 $$ 10766&&only 289 $$ 1252&&only 290 $$ 621&&only 290 $$ 621 $$ 1475&&only 290 $$ 1432&&only 291 $$ 14791&&only 292 $$ 2216&&only 293 $$ 842&&only 294 $$ 346 $$ 633 $$ 634 $$ 635 $$ 636 $$ 637 $$ 638 $$ 639 $$ 640 $$ 641 $$ 642 $$ 643 $$ 644 $$ 645 $$ 646 $$ 647 $$ 849 $$ 850 $$ 851 $$ 1002 $$ 1178 $$ 1219 $$ 1949 $$ 3582&&only 294 $$ 346 $$ 634 $$ 635 $$ 636 $$ 637 $$ 638 $$ 639 $$ 640 $$ 641 $$ 642 $$ 643 $$ 644 $$ 645 $$ 646 $$ 647 $$ 849 $$ 850 $$ 851 $$ 1002 $$ 1178 $$ 1219 $$ 1558 $$ 2252&&only 294 $$ 346 $$ 637 $$ 638 $$ 639 $$ 640 $$ 641 $$ 642 $$ 643 $$ 644 $$ 645 $$ 646 $$ 647 $$ 850 $$ 851 $$ 1178 $$ 1219 $$ 2252 $$ 3582&&only 294 $$ 346 $$ 634 $$ 635 $$ 636 $$ 637 $$ 638 $$ 639 $$ 640 $$ 641 $$ 642 $$ 643 $$ 644 $$ 645 $$ 646 $$ 647 $$ 849 $$ 850 $$ 851 $$ 1002 $$ 1178 $$ 1219 $$ 1558 $$ 1949&&only 294 $$ 346 $$ 633 $$ 634 $$ 635 $$ 636 $$ 637 $$ 638 $$ 639 $$ 640 $$ 641 $$ 642 $$ 643 $$ 644 $$ 645 $$ 646 $$ 647 $$ 849 $$ 850 $$ 851 $$ 1002 $$ 1178 $$ 1219 $$ 1558 $$ 2252&&only 294 $$ 644 $$ 645 $$ 646 $$ 647&&only 296 $$ 352 $$ 1582&&only 296 $$ 1185&&only 296 $$ 16806&&only 297 $$ 8075&&only 297 $$ 11200&&only 297 $$ 17512 $$ 17513&&only 298 $$ 904&&only 298 $$ 3202 $$ 10715&&only 298 $$ 3202&&only 298 $$ 3561&&only 298 $$ 16728&&only 299 $$ 1041&&only 300 $$ 1067 $$ 1153 $$ 1443&&only 300 $$ 1153&&only 300 $$ 1443&&only 300 $$ 3617&&only 301 $$ 2686&&only 301 $$ 15343 $$ 15344&&only 302 $$ 363 $$ 1407&&only 302 $$ 363 $$ 515 $$ 791 $$ 1407 $$ 1615 $$ 1631 $$ 12583&&only 302 $$ 363&&only 302 $$ 363 $$ 5375&&only 302 $$ 363 $$ 515 $$ 791 $$ 1407 $$ 1615 $$ 2815&&only 302 $$ 515&&only 302 $$ 515 $$ 2815&&only 302 $$ 12188&&only 305 $$ 1285 $$ 1286&&only 305 $$ 1705&&only 305 $$ 1883&&only 306 $$ 355 $$ 1155 $$ 1565&&only 306 $$ 953 $$ 1667&&only 308 $$ 321&&only 309 $$ 13309&&only 310 $$ 524&&only 310 $$ 524 $$ 6913&&only 310 $$ 524 $$ 1905&&only 311 $$ 719&&only 312 $$ 4820&&only 312 $$ 5408&&only 313 $$ 618&&only 313 $$ 1130 $$ 1131 $$ 1769 $$ 4745 $$ 6137 $$ 12214 $$ 12215&&only 313 $$ 1131&&only 314 $$ 323 $$ 1134&&only 314 $$ 1134&&only 314 $$ 1134 $$ 5161&&only 315 $$ 785 $$ 786&&only 315 $$ 786&&only 316 $$ 500&&only 316 $$ 1494&&only 317 $$ 1644&&only 317 $$ 17041&&only 318 $$ 1143&&only 319 $$ 1303 $$ 1973&&only 319 $$ 1303&&only 319 $$ 1973&&only 319 $$ 6345&&only 320 $$ 463&&only 320 $$ 463 $$ 821&&only 320 $$ 5834&&only 320 $$ 10346&&only 322 $$ 345&&only 322 $$ 1257&&only 322 $$ 6128&&only 323 $$ 8800 $$ 8801 $$ 8802&&only 324 $$ 387 $$ 989&&only 324 $$ 989&&only 325 $$ 1070&&only 325 $$ 2177&&only 326 $$ 7124&&only 327 $$ 1348&&only 327 $$ 2322 $$ 2323 $$ 2713&&only 328 $$ 445&&only 329 $$ 902 $$ 1258 $$ 1581 $$ 1610&&only 329 $$ 902 $$ 1258 $$ 1581&&only 329 $$ 902 $$ 1258 $$ 1518 $$ 1581&&only 329 $$ 902 $$ 1258&&only 329 $$ 902 $$ 1258 $$ 1581 $$ 14580&&only 329 $$ 902 $$ 1258 $$ 1518 $$ 1581 $$ 1610&&only 329 $$ 1571 $$ 2527&&only 329 $$ 1898 $$ 3450 $$ 5832&&only 329 $$ 5007 $$ 5165 $$ 5331&&only 329 $$ 5007 $$ 5165 $$ 5331 $$ 16888&&only 330 $$ 749 $$ 930 $$ 1051 $$ 1052 $$ 1053 $$ 1196 $$ 1295&&only 330 $$ 749 $$ 930 $$ 972 $$ 1051 $$ 1052 $$ 1053 $$ 1054 $$ 1196 $$ 1295&&only 330 $$ 749 $$ 930 $$ 972 $$ 1051 $$ 1052 $$ 1053 $$ 1054 $$ 1196&&only 330 $$ 749 $$ 930 $$ 972 $$ 1051 $$ 1052 $$ 1053 $$ 1054 $$ 1196 $$ 1591&&only 330 $$ 930&&only 330 $$ 930 $$ 972 $$ 1052&&only 330 $$ 930 $$ 972 $$ 1051 $$ 1052 $$ 1196 $$ 1295&&only 330 $$ 930 $$ 972 $$ 1051 $$ 1052 $$ 1054 $$ 1196 $$ 1295&&only 330 $$ 930 $$ 972 $$ 1051 $$ 1052 $$ 1184 $$ 1196&&only 330 $$ 930 $$ 972 $$ 1051 $$ 1052 $$ 1184 $$ 1196 $$ 1295&&only 330 $$ 930 $$ 972 $$ 1051 $$ 1052 $$ 1196&&only 330 $$ 930 $$ 972 $$ 1052 $$ 1184&&only 331 $$ 4114&&only 332 $$ 1393 $$ 15016&&only 332 $$ 6756&&only 333 $$ 16115&&only 335 $$ 3798&&only 335 $$ 6341&&only 335 $$ 7467&&only 335 $$ 12953&&only 335 $$ 14188&&only 335 $$ 17241&&only 336 $$ 759 $$ 1363&&only 336 $$ 6247&&only 337 $$ 383&&only 337 $$ 14558 $$ 14559&&only 338 $$ 13203&&only 339 $$ 1751 $$ 1760&&only 339 $$ 1751 $$ 1760 $$ 8862&&only 339 $$ 1760&&only 339 $$ 3995 $$ 5305 $$ 5706&&only 339 $$ 3995 $$ 5706&&only 339 $$ 4493 $$ 4868&&only 339 $$ 4992 $$ 5287 $$ 5288 $$ 11820&&only 339 $$ 4992 $$ 5287 $$ 5288&&only 340 $$ 11868&&only 341 $$ 443&&only 341 $$ 443 $$ 3935&&only 341 $$ 1082&&only 341 $$ 1611 $$ 7322&&only 341 $$ 1665 $$ 5946 $$ 6474&&only 341 $$ 2605&&only 342 $$ 1482&&only 343 $$ 617&&only 345 $$ 840 $$ 1687&&only 346 $$ 633 $$ 634 $$ 635 $$ 636 $$ 637 $$ 638 $$ 639 $$ 640 $$ 641 $$ 642 $$ 643 $$ 849 $$ 850 $$ 851 $$ 1002 $$ 1178 $$ 1219 $$ 1949 $$ 3582&&only 346 $$ 633 $$ 634 $$ 635 $$ 636 $$ 637 $$ 638 $$ 639 $$ 640 $$ 641 $$ 642 $$ 643 $$ 849 $$ 850 $$ 851 $$ 1002 $$ 1178 $$ 1219 $$ 1558 $$ 2252&&only 346 $$ 633 $$ 634 $$ 635 $$ 636 $$ 637 $$ 638 $$ 639 $$ 640 $$ 641 $$ 642 $$ 643 $$ 644 $$ 645 $$ 646 $$ 647 $$ 849 $$ 850 $$ 851 $$ 1002 $$ 1178 $$ 1219 $$ 1558 $$ 2252&&only 346 $$ 634 $$ 635 $$ 636 $$ 637 $$ 638 $$ 639 $$ 640 $$ 641 $$ 642 $$ 643 $$ 849 $$ 850 $$ 851 $$ 1002 $$ 1178 $$ 1219 $$ 1558 $$ 1949&&only 346 $$ 634 $$ 635 $$ 636 $$ 637 $$ 638 $$ 639 $$ 640 $$ 641 $$ 642 $$ 643 $$ 644 $$ 645 $$ 646 $$ 647 $$ 849 $$ 850 $$ 851 $$ 1002 $$ 1178 $$ 1219 $$ 1558 $$ 2252&&only 346 $$ 637 $$ 638 $$ 639 $$ 640 $$ 641 $$ 642 $$ 643 $$ 849 $$ 850 $$ 851 $$ 1178 $$ 1219 $$ 1949 $$ 3582 $$ 12539&&only 346 $$ 640 $$ 641 $$ 642 $$ 643 $$ 850 $$ 851 $$ 1178 $$ 1219 $$ 1558 $$ 1949&&only 346 $$ 640 $$ 641 $$ 642 $$ 643 $$ 850 $$ 851 $$ 1178 $$ 1219 $$ 1949&&only 346 $$ 642 $$ 643 $$ 850 $$ 851 $$ 1178&&only 346 $$ 4148 $$ 4150&&only 347 $$ 365&&only 347 $$ 365 $$ 1297&&only 349 $$ 541 $$ 677 $$ 1122 $$ 12177&&only 349 $$ 541 $$ 2623&&only 349 $$ 1294 $$ 1351&&only 349 $$ 2623&&only 349 $$ 4676&&only 349 $$ 6627&&only 349 $$ 7983&&only 349 $$ 14816&&only 349 $$ 15610&&only 351 $$ 428 $$ 3695&&only 352 $$ 1582&&only 353 $$ 1254&&only 353 $$ 1358&&only 355 $$ 3160&&only 356 $$ 370&&only 356 $$ 1346 $$ 1610 $$ 3773&&only 356 $$ 2654&&only 356 $$ 3773&&only 357 $$ 1175&&only 357 $$ 1322&&only 359 $$ 393 $$ 517&&only 359 $$ 504 $$ 548 $$ 729&&only 359 $$ 548&&only 359 $$ 1036&&only 359 $$ 12280&&only 360 $$ 9760&&only 360 $$ 16355&&only 361 $$ 1105&&only 361 $$ 7536&&only 362 $$ 1566 $$ 1567 $$ 1586&&only 362 $$ 1566 $$ 1567 $$ 1736&&only 362 $$ 1567&&only 362 $$ 7377 $$ 12411&&only 363 $$ 791 $$ 1407 $$ 1615&&only 365 $$ 557 $$ 8162 $$ 9410&&only 366 $$ 10747&&only 366 $$ 14141&&only 367 $$ 1775&&only 367 $$ 2034&&only 370 $$ 1316 $$ 5131&&only 370 $$ 5131&&only 371 $$ 1361 $$ 12279&&only 371 $$ 1361&&only 372 $$ 1688 $$ 6144&&only 372 $$ 1688 $$ 2228&&only 372 $$ 2228&&only 374 $$ 764&&only 374 $$ 2103&&only 374 $$ 7841&&only 374 $$ 9731&&only 375 $$ 482 $$ 767&&only 375 $$ 482&&only 376 $$ 1129&&only 377 $$ 377&&only 377 $$ 13285&&only 378 $$ 11905&&only 379 $$ 16766&&only 380 $$ 562 $$ 11515&&only 380 $$ 4608&&only 380 $$ 8781&&only 380 $$ 14554&&only 382 $$ 5629&&only 384 $$ 2592&&only 384 $$ 2695&&only 384 $$ 5634&&only 384 $$ 8442&&only 384 $$ 15575&&only 385 $$ 399 $$ 986&&only 385 $$ 986&&only 385 $$ 986 $$ 5497&&only 385 $$ 3413&&only 385 $$ 3413 $$ 5497&&only 386 $$ 1349&&only 386 $$ 1358 $$ 2000&&only 386 $$ 9879&&only 386 $$ 14763 $$ 14764&&only 387 $$ 989&&only 388 $$ 2298&&only 389 $$ 1323&&only 389 $$ 1346 $$ 1610&&only 390 $$ 790&&only 392 $$ 675 $$ 676 $$ 1479&&only 392 $$ 675 $$ 1479&&only 392 $$ 1238 $$ 2291&&only 392 $$ 1479&&only 392 $$ 2291&&only 393 $$ 517&&only 393 $$ 517 $$ 4304&&only 393 $$ 3201&&only 394 $$ 394 $$ 5156 $$ 13949&&only 394 $$ 987 $$ 16835&&only 394 $$ 3332 $$ 5156&&only 395 $$ 1254&&only 396 $$ 2401&&only 396 $$ 3087&&only 396 $$ 4047&&only 396 $$ 10045&&only 397 $$ 440&&only 397 $$ 479 $$ 1386&&only 398 $$ 11912&&only 401 $$ 652 $$ 1107 $$ 1321 $$ 1417 $$ 1456&&only 401 $$ 1107 $$ 1321 $$ 1417 $$ 1456&&only 401 $$ 1107 $$ 1417 $$ 1456&&only 401 $$ 1107 $$ 1321&&only 401 $$ 1107&&only 401 $$ 1280 $$ 1881 $$ 6471 $$ 6472&&only 401 $$ 1280 $$ 6470 $$ 6471 $$ 6472&&only 401 $$ 1280 $$ 6471 $$ 6472&&only 402 $$ 1195&&only 403 $$ 3165&&only 403 $$ 5838&&only 404 $$ 5252&&only 404 $$ 10306&&only 405 $$ 2616&&only 406 $$ 2264&&only 407 $$ 1362&&only 407 $$ 2476&&only 410 $$ 1398&&only 410 $$ 2773&&only 411 $$ 459 $$ 2532&&only 411 $$ 459&&only 412 $$ 816&&only 412 $$ 816 $$ 9894&&only 412 $$ 816 $$ 14914&&only 412 $$ 816 $$ 4469&&only 413 $$ 3346&&only 415 $$ 934 $$ 4051&&only 415 $$ 993&&only 416 $$ 846 $$ 1786 $$ 2101 $$ 2831&&only 416 $$ 1786 $$ 1937 $$ 1955 $$ 3876&&only 416 $$ 1786 $$ 3876&&only 416 $$ 1786 $$ 2101&&only 416 $$ 3269&&only 416 $$ 4510 $$ 5598&&only 416 $$ 4510 $$ 7232&&only 416 $$ 5598 $$ 15652&&only 417 $$ 854&&only 417 $$ 2223&&only 417 $$ 2241&&only 419 $$ 878&&only 419 $$ 878 $$ 1030&&only 419 $$ 878 $$ 16444&&only 419 $$ 7437&&only 419 $$ 14382&&only 420 $$ 11108&&only 422 $$ 1198 $$ 5155&&only 423 $$ 1200&&only 423 $$ 14691&&only 424 $$ 14825&&only 426 $$ 1392&&only 426 $$ 3546&&only 427 $$ 1475 $$ 2428&&only 427 $$ 2428&&only 427 $$ 2428 $$ 13255&&only 427 $$ 2639&&only 427 $$ 2639 $$ 2817&&only 427 $$ 3991&&only 427 $$ 8240&&only 427 $$ 8240 $$ 9627 $$ 16174&&only 428 $$ 13134&&only 429 $$ 1279&&only 430 $$ 3910&&only 430 $$ 16370&&only 431 $$ 1953 $$ 2010&&only 431 $$ 4365&&only 432 $$ 883 $$ 884 $$ 1024 $$ 1025 $$ 1225&&only 432 $$ 883 $$ 884 $$ 1225&&only 432 $$ 884&&only 432 $$ 884 $$ 8703&&only 432 $$ 884 $$ 1225&&only 432 $$ 1530 $$ 4010&&only 433 $$ 713 $$ 714&&only 434 $$ 1421 $$ 16991&&only 435 $$ 1615&&only 436 $$ 1629 $$ 1743 $$ 1744 $$ 1771 $$ 4752 $$ 12291&&only 436 $$ 1629 $$ 1653 $$ 1743 $$ 1744&&only 436 $$ 1629 $$ 1743 $$ 1744&&only 436 $$ 1629 $$ 1743 $$ 1744 $$ 1771 $$ 2294&&only 436 $$ 1629 $$ 1653 $$ 1743 $$ 1744 $$ 1771 $$ 2294&&only 437 $$ 932 $$ 9483&&only 437 $$ 932&&only 437 $$ 1811&&only 437 $$ 6313&&only 437 $$ 16839&&only 439 $$ 1465&&only 439 $$ 7178&&only 439 $$ 8847 $$ 12699&&only 439 $$ 10527&&only 439 $$ 12698&&only 439 $$ 13176&&only 440 $$ 1066 $$ 1725&&only 441 $$ 754&&only 442 $$ 1163&&only 444 $$ 529&&only 444 $$ 8704&&only 445 $$ 658&&only 445 $$ 13869&&only 446 $$ 1719&&only 446 $$ 10572&&only 448 $$ 688 $$ 1021&&only 448 $$ 1330&&only 448 $$ 1441&&only 449 $$ 741&&only 450 $$ 1914 $$ 2189 $$ 3228&&only 450 $$ 4771&&only 451 $$ 1037&&only 451 $$ 1037 $$ 1135&&only 453 $$ 2381&&only 454 $$ 2636&&only 454 $$ 3046&&only 454 $$ 3733&&only 454 $$ 12364&&only 455 $$ 551&&only 455 $$ 1062 $$ 1495&&only 455 $$ 2696 $$ 3395&&only 455 $$ 6973&&only 456 $$ 1746&&only 456 $$ 7277 $$ 11361&&only 456 $$ 11372 $$ 16403&&only 457 $$ 4642&&only 457 $$ 8861&&only 458 $$ 798 $$ 966&&only 458 $$ 966&&only 458 $$ 1090 $$ 4248&&only 458 $$ 2063 $$ 11827&&only 458 $$ 4248&&only 458 $$ 4688&&only 460 $$ 5878&&only 461 $$ 1269 $$ 13229&&only 461 $$ 5027&&only 464 $$ 614 $$ 615 $$ 616 $$ 1414 $$ 2033 $$ 2135&&only 464 $$ 614 $$ 615 $$ 616 $$ 1414&&only 464 $$ 614 $$ 615&&only 464 $$ 614 $$ 615 $$ 616 $$ 4506&&only 464 $$ 614 $$ 615 $$ 1414&&only 464 $$ 615 $$ 616 $$ 1414&&only 465 $$ 676 $$ 1836&&only 465 $$ 979 $$ 2617&&only 465 $$ 1836&&only 465 $$ 2617&&only 465 $$ 2617 $$ 9891&&only 466 $$ 837 $$ 992 $$ 1263&&only 466 $$ 837 $$ 992&&only 466 $$ 1292&&only 467 $$ 468 $$ 497 $$ 498&&only 467 $$ 497&&only 468 $$ 498&&only 471 $$ 1300&&only 472 $$ 2258&&only 472 $$ 4658&&only 473 $$ 14239&&only 475 $$ 913&&only 475 $$ 913 $$ 7567&&only 475 $$ 913 $$ 1180&&only 476 $$ 914&&only 477 $$ 915&&only 477 $$ 915 $$ 1378&&only 477 $$ 1378&&only 477 $$ 3242&&only 479 $$ 723&&only 479 $$ 1386&&only 479 $$ 6966&&only 480 $$ 1238&&only 480 $$ 1341&&only 480 $$ 3312&&only 481 $$ 510&&only 482 $$ 7735 $$ 10313&&only 484 $$ 813 $$ 11851&&only 484 $$ 1289&&only 484 $$ 7001&&only 485 $$ 1181 $$ 12120&&only 485 $$ 1181&&only 486 $$ 797&&only 486 $$ 797 $$ 1078&&only 486 $$ 797 $$ 10478&&only 486 $$ 1078&&only 486 $$ 3900&&only 487 $$ 4247&&only 488 $$ 586&&only 490 $$ 2282&&only 491 $$ 1519 $$ 3576 $$ 4526 $$ 5327&&only 491 $$ 6303&&only 493 $$ 988&&only 493 $$ 4879&&only 494 $$ 934 $$ 935 $$ 1499 $$ 1791&&only 494 $$ 935 $$ 1499 $$ 1791 $$ 5377&&only 494 $$ 935 $$ 3459 $$ 3748&&only 494 $$ 935&&only 494 $$ 1499&&only 494 $$ 3748&&only 494 $$ 3775&&only 496 $$ 861&&only 496 $$ 1397&&only 499 $$ 3136&&only 501 $$ 2147&&only 501 $$ 5434&&only 501 $$ 5501&&only 501 $$ 8435&&only 501 $$ 8625&&only 501 $$ 15936&&only 504 $$ 548 $$ 729&&only 504 $$ 729&&only 506 $$ 660 $$ 1012 $$ 1049&&only 506 $$ 1049&&only 506 $$ 1049 $$ 5633&&only 507 $$ 1376&&only 507 $$ 7584&&only 507 $$ 12856&&only 508 $$ 2846&&only 508 $$ 3858&&only 508 $$ 6749&&only 509 $$ 597 $$ 1195&&only 509 $$ 597&&only 511 $$ 558&&only 511 $$ 558 $$ 592 $$ 1734&&only 511 $$ 558 $$ 16633&&only 512 $$ 1252&&only 513 $$ 1070&&only 514 $$ 1005&&only 514 $$ 1911 $$ 2569&&only 514 $$ 1911 $$ 2569 $$ 13924 $$ 13925&&only 514 $$ 1911 $$ 2569 $$ 3905&&only 514 $$ 1911 $$ 2569 $$ 10479&&only 514 $$ 1911&&only 514 $$ 2803&&only 515 $$ 791 $$ 1631 $$ 1952&&only 515 $$ 791 $$ 1208 $$ 1631&&only 515 $$ 791 $$ 1208 $$ 1631 $$ 1952&&only 515 $$ 1631&&only 518 $$ 772&&only 520 $$ 580&&only 522 $$ 810&&only 522 $$ 810 $$ 13321&&only 525 $$ 599 $$ 600&&only 525 $$ 599&&only 525 $$ 599 $$ 1811&&only 526 $$ 1433&&only 526 $$ 3981&&only 527 $$ 828&&only 527 $$ 3608&&only 527 $$ 5776&&only 527 $$ 11897&&only 529 $$ 1172 $$ 1216&&only 529 $$ 8022&&only 530 $$ 3885 $$ 5568&&only 530 $$ 4110&&only 530 $$ 4363&&only 530 $$ 5518&&only 530 $$ 7212&&only 530 $$ 9860&&only 530 $$ 11295&&only 530 $$ 11423&&only 530 $$ 13788&&only 530 $$ 16432&&only 531 $$ 738 $$ 739 $$ 740 $$ 1229 $$ 2480&&only 531 $$ 738 $$ 739 $$ 740 $$ 1229 $$ 8221&&only 531 $$ 738 $$ 739 $$ 740 $$ 1229 $$ 2343&&only 531 $$ 738 $$ 739 $$ 740 $$ 2480 $$ 15118&&only 532 $$ 4237&&only 533 $$ 14985&&only 533 $$ 17373&&only 534 $$ 3609&&only 535 $$ 1297&&only 535 $$ 8739&&only 537 $$ 935 $$ 1791&&only 537 $$ 12388&&only 540 $$ 904&&only 540 $$ 1577&&only 540 $$ 5757&&only 540 $$ 14607&&only 541 $$ 13628&&only 542 $$ 681 $$ 1560&&only 543 $$ 688 $$ 1021&&only 544 $$ 563 $$ 2214&&only 544 $$ 563&&only 545 $$ 12770&&only 546 $$ 733&&only 546 $$ 1114&&only 547 $$ 14144&&only 549 $$ 1136&&only 549 $$ 1136 $$ 9951&&only 549 $$ 4288&&only 550 $$ 742&&only 551 $$ 1277 $$ 12339&&only 551 $$ 1277 $$ 5889&&only 551 $$ 1277&&only 551 $$ 5889&&only 551 $$ 16831&&only 555 $$ 1442&&only 556 $$ 1441&&only 556 $$ 4476&&only 556 $$ 8112&&only 556 $$ 11127&&only 557 $$ 1075&&only 557 $$ 1075 $$ 1748&&only 557 $$ 1075 $$ 1150 $$ 1748&&only 557 $$ 1075 $$ 1748 $$ 2091 $$ 2170&&only 559 $$ 5456&&only 560 $$ 3624&&only 560 $$ 4342&&only 560 $$ 10249&&only 561 $$ 6402 $$ 12826&&only 562 $$ 990 $$ 1299&&only 562 $$ 2037&&only 564 $$ 3581&&only 564 $$ 6357&&only 565 $$ 566 $$ 795&&only 565 $$ 795&&only 568 $$ 1426&&only 568 $$ 1740&&only 568 $$ 5566&&only 569 $$ 1317 $$ 1368&&only 569 $$ 1317&&only 569 $$ 1944&&only 573 $$ 574 $$ 575&&only 573 $$ 574 $$ 575 $$ 802&&only 573 $$ 574 $$ 575 $$ 1894&&only 573 $$ 574 $$ 575 $$ 11176&&only 573 $$ 574 $$ 575 $$ 16906&&only 573 $$ 575&&only 577 $$ 578 $$ 11838&&only 577 $$ 578 $$ 2533&&only 577 $$ 578 $$ 2042&&only 577 $$ 578&&only 579 $$ 9183&&only 583 $$ 1271 $$ 3138&&only 583 $$ 8007&&only 584 $$ 595&&only 584 $$ 595 $$ 3106&&only 584 $$ 1664 $$ 3106&&only 585 $$ 3185&&only 589 $$ 2778&&only 590 $$ 1507&&only 591 $$ 981 $$ 1167 $$ 11863&&only 591 $$ 981 $$ 1167&&only 591 $$ 981 $$ 13327&&only 591 $$ 1167&&only 593 $$ 817&&only 593 $$ 817 $$ 2429&&only 593 $$ 817 $$ 824 $$ 2429&&only 594 $$ 3242&&only 596 $$ 2082 $$ 2999 $$ 5781&&only 596 $$ 2999 $$ 5781&&only 597 $$ 1195&&only 598 $$ 825 $$ 826 $$ 827 $$ 983 $$ 1095 $$ 9486&&only 598 $$ 1533&&only 602 $$ 6959&&only 603 $$ 1526&&only 604 $$ 869 $$ 870 $$ 1017 $$ 2124&&only 604 $$ 870 $$ 1017&&only 604 $$ 870 $$ 1017 $$ 2124&&only 604 $$ 870 $$ 1017 $$ 5154&&only 604 $$ 870&&only 605 $$ 1238&&only 605 $$ 4320&&only 606 $$ 613&&only 607 $$ 796&&only 608 $$ 1389&&only 608 $$ 5156 $$ 13286&&only 608 $$ 15642&&only 611 $$ 1174&&only 616 $$ 1414&&only 617 $$ 1453&&only 617 $$ 6217&&only 617 $$ 13284&&only 618 $$ 766&&only 618 $$ 2231&&only 618 $$ 5643&&only 619 $$ 620&&only 619 $$ 1892&&only 619 $$ 3688&&only 619 $$ 4145&&only 619 $$ 6219&&only 621 $$ 1475&&only 622 $$ 1217&&only 623 $$ 835 $$ 836 $$ 1218 $$ 1833&&only 623 $$ 1218&&only 623 $$ 1218 $$ 16481&&only 624 $$ 10780&&only 624 $$ 14112&&only 625 $$ 3868&&only 626 $$ 627 $$ 839 $$ 1415 $$ 1834&&only 626 $$ 627 $$ 1834&&only 626 $$ 627&&only 626 $$ 839 $$ 1415&&only 627 $$ 1834&&only 628 $$ 6261&&only 628 $$ 7800&&only 629 $$ 841&&only 630 $$ 7227&&only 631 $$ 8744 $$ 13746&&only 632 $$ 2311&&only 633 $$ 634 $$ 635 $$ 636&&only 633 $$ 634 $$ 635 $$ 636 $$ 637 $$ 638 $$ 639 $$ 849 $$ 1002&&only 634 $$ 635 $$ 636 $$ 849 $$ 1002&&only 634 $$ 635 $$ 636 $$ 637 $$ 638 $$ 639 $$ 640 $$ 641 $$ 849 $$ 1002 $$ 1558 $$ 2252&&only 634 $$ 635 $$ 636 $$ 1002&&only 634 $$ 635 $$ 636 $$ 637 $$ 638 $$ 639 $$ 849 $$ 1002&&only 644 $$ 645 $$ 646 $$ 647&&only 649 $$ 1011&&only 651 $$ 1005 $$ 1211&&only 651 $$ 1211&&only 651 $$ 1211 $$ 2420&&only 652 $$ 1110&&only 652 $$ 1321&&only 652 $$ 9191&&only 652 $$ 10571&&only 653 $$ 3231&&only 653 $$ 4020&&only 653 $$ 4305&&only 654 $$ 3213&&only 654 $$ 6361&&only 655 $$ 857 $$ 1914&&only 656 $$ 1008 $$ 1009 $$ 1663&&only 656 $$ 1008 $$ 1009&&only 656 $$ 1008 $$ 1009 $$ 2072&&only 656 $$ 1008 $$ 2072 $$ 15805&&only 656 $$ 1009&&only 657 $$ 858 $$ 1010&&only 657 $$ 1938&&only 657 $$ 5441&&only 657 $$ 8740&&only 658 $$ 861&&only 658 $$ 1653&&only 659 $$ 4344&&only 660 $$ 3655&&only 660 $$ 11492&&only 662 $$ 2091 $$ 2170&&only 662 $$ 2170 $$ 9100&&only 662 $$ 2170&&only 663 $$ 865 $$ 1457&&only 663 $$ 865&&only 663 $$ 3454&&only 665 $$ 666&&only 665 $$ 666 $$ 6324&&only 667 $$ 668&&only 667 $$ 1014 $$ 1015 $$ 1115 $$ 1116&&only 669 $$ 4664&&only 670 $$ 1221 $$ 10864&&only 670 $$ 2403&&only 670 $$ 12150 $$ 12151&&only 672 $$ 673 $$ 867&&only 672 $$ 867&&only 673 $$ 867&&only 673 $$ 867 $$ 9784&&only 673 $$ 867 $$ 957 $$ 5532&&only 673 $$ 867 $$ 957&&only 674 $$ 1183 $$ 1222&&only 675 $$ 676 $$ 8966&&only 676 $$ 7101&&only 677 $$ 1122&&only 677 $$ 1122 $$ 1561&&only 677 $$ 14270&&only 679 $$ 999 $$ 1020&&only 679 $$ 1020&&only 679 $$ 1020 $$ 13230&&only 680 $$ 1269&&only 681 $$ 1560&&only 681 $$ 3904&&only 682 $$ 808&&only 683 $$ 878 $$ 1125&&only 683 $$ 1125&&only 684 $$ 685&&only 684 $$ 685 $$ 6539&&only 686 $$ 1126&&only 686 $$ 1284&&only 686 $$ 1284 $$ 7504&&only 687 $$ 1097&&only 687 $$ 1980&&only 688 $$ 1021&&only 689 $$ 690 $$ 1458&&only 689 $$ 690&&only 689 $$ 1609&&only 690 $$ 1458&&only 691 $$ 1223&&only 693 $$ 1359&&only 694 $$ 1245&&only 694 $$ 1245 $$ 7317&&only 695 $$ 696 $$ 12199&&only 695 $$ 696 $$ 1537&&only 695 $$ 696 $$ 13736&&only 695 $$ 696 $$ 6005&&only 695 $$ 696 $$ 10515&&only 695 $$ 696 $$ 10467&&only 695 $$ 696 $$ 15048&&only 696 $$ 1537&&only 697 $$ 1244 $$ 10516&&only 697 $$ 1579&&only 697 $$ 5474&&only 698 $$ 699 $$ 700 $$ 7355&&only 698 $$ 699 $$ 700 $$ 2505&&only 698 $$ 699 $$ 700 $$ 9863&&only 698 $$ 699 $$ 700 $$ 2238&&only 698 $$ 699 $$ 700 $$ 7734&&only 699 $$ 700 $$ 7734&&only 701 $$ 702 $$ 12206&&only 701 $$ 702&&only 703 $$ 704 $$ 705 $$ 706 $$ 707 $$ 1226 $$ 8210&&only 703 $$ 704 $$ 705 $$ 706 $$ 707 $$ 1226 $$ 1247&&only 703 $$ 704 $$ 705 $$ 706 $$ 1226&&only 703 $$ 704 $$ 705 $$ 1226&&only 704 $$ 705 $$ 706 $$ 707 $$ 1247&&only 706 $$ 707 $$ 1247&&only 707 $$ 1247&&only 708 $$ 709 $$ 710 $$ 711&&only 713 $$ 714&&only 715 $$ 2005&&only 715 $$ 2893&&only 715 $$ 12631&&only 716 $$ 890 $$ 1187 $$ 1345&&only 716 $$ 890 $$ 1187 $$ 1345 $$ 9923&&only 716 $$ 890 $$ 1345&&only 716 $$ 890&&only 716 $$ 890 $$ 1187 $$ 1345 $$ 3587&&only 717 $$ 1026&&only 717 $$ 1026 $$ 2429&&only 717 $$ 1026 $$ 10748&&only 718 $$ 13231&&only 719 $$ 6152&&only 720 $$ 3704&&only 720 $$ 5329&&only 721 $$ 3092&&only 721 $$ 10273&&only 721 $$ 12216&&only 721 $$ 13238&&only 722 $$ 1132 $$ 1273&&only 723 $$ 1971&&only 724 $$ 814 $$ 895&&only 724 $$ 895&&only 724 $$ 895 $$ 2864&&only 724 $$ 895 $$ 11107&&only 724 $$ 2223&&only 725 $$ 896&&only 725 $$ 896 $$ 13215&&only 726 $$ 727&&only 726 $$ 9036&&only 726 $$ 9555&&only 727 $$ 1642&&only 728 $$ 1006&&only 728 $$ 1708&&only 730 $$ 1137 $$ 1304&&only 730 $$ 1304&&only 731 $$ 1275 $$ 1789 $$ 2262&&only 731 $$ 1275 $$ 2630 $$ 13916&&only 731 $$ 1275 $$ 2630 $$ 3364&&only 731 $$ 1275 $$ 1789 $$ 5125&&only 731 $$ 1275 $$ 2630 $$ 5934&&only 731 $$ 1789 $$ 2262&&only 731 $$ 1789 $$ 2262 $$ 10726 $$ 14666&&only 733 $$ 1589 $$ 1710&&only 733 $$ 12289&&only 733 $$ 13741&&only 734 $$ 1228&&only 735 $$ 736 $$ 910 $$ 1043 $$ 1603&&only 735 $$ 1603&&only 736 $$ 910 $$ 1043&&only 736 $$ 910&&only 738 $$ 739 $$ 740 $$ 1229 $$ 2480&&only 743 $$ 744 $$ 1231&&only 743 $$ 1231&&only 745 $$ 921&&only 746 $$ 922&&only 747 $$ 924&&only 748 $$ 1234&&only 748 $$ 8226&&only 749 $$ 1053&&only 750 $$ 751 $$ 1148 $$ 1236 $$ 4531&&only 750 $$ 751 $$ 1148 $$ 1236 $$ 1429&&only 750 $$ 1148&&only 750 $$ 4628 $$ 11338&&only 751 $$ 1236 $$ 1429&&only 751 $$ 1236 $$ 1429 $$ 2739&&only 751 $$ 1236 $$ 4531&&only 751 $$ 1236 $$ 1429 $$ 2739 $$ 3592&&only 751 $$ 1429&&only 752 $$ 931&&only 752 $$ 6069&&only 753 $$ 1603&&only 755 $$ 1309 $$ 7738&&only 755 $$ 6170&&only 756 $$ 1309 $$ 12352&&only 756 $$ 3922&&only 757 $$ 12356&&only 758 $$ 938 $$ 1523 $$ 2190&&only 758 $$ 938&&only 758 $$ 938 $$ 2190 $$ 3750&&only 758 $$ 2190&&only 759 $$ 1363&&only 759 $$ 1363 $$ 3933&&only 759 $$ 1363 $$ 3107&&only 759 $$ 3107&&only 759 $$ 3933&&only 759 $$ 12898&&only 761 $$ 2006&&only 761 $$ 5042&&only 762 $$ 1013&&only 762 $$ 14291&&only 764 $$ 3332&&only 765 $$ 3362&&only 767 $$ 1458&&only 768 $$ 2074&&only 768 $$ 3924&&only 768 $$ 13126&&only 768 $$ 15869&&only 768 $$ 16342&&only 770 $$ 1406 $$ 2561&&only 770 $$ 1807 $$ 2296&&only 770 $$ 1916 $$ 1946 $$ 4022 $$ 4472 $$ 5743&&only 770 $$ 2561&&only 770 $$ 4022&&only 770 $$ 8719&&only 771 $$ 772&&only 771 $$ 1749&&only 771 $$ 15142&&only 773 $$ 1816&&only 773 $$ 1816 $$ 3025&&only 773 $$ 3753&&only 776 $$ 2214&&only 778 $$ 15171&&only 779 $$ 780 $$ 781 $$ 952 $$ 1154 $$ 12393&&only 779 $$ 780 $$ 781 $$ 952 $$ 1154 $$ 1206&&only 779 $$ 780 $$ 952 $$ 1154&&only 779 $$ 780 $$ 781 $$ 952 $$ 1154&&only 779 $$ 780 $$ 781 $$ 952 $$ 1154 $$ 1206 $$ 4973&&only 781 $$ 1206&&only 782 $$ 1667&&only 782 $$ 3502&&only 782 $$ 12395&&only 782 $$ 14553&&only 782 $$ 16919&&only 783 $$ 809&&only 784 $$ 5046&&only 787 $$ 1207 $$ 1866&&only 788 $$ 1721&&only 788 $$ 1778&&only 789 $$ 1207 $$ 1866&&only 789 $$ 1207 $$ 2917&&only 789 $$ 2483&&only 789 $$ 2629&&only 789 $$ 6455 $$ 6662&&only 790 $$ 4609&&only 792 $$ 2451 $$ 2452 $$ 2467 $$ 2503 $$ 5668&&only 794 $$ 2068 $$ 2242&&only 794 $$ 3649&&only 796 $$ 3972&&only 797 $$ 11528&&only 798 $$ 966&&only 799 $$ 1831 $$ 2064&&only 799 $$ 2325&&only 799 $$ 11828&&only 800 $$ 1637&&only 801 $$ 3089&&only 802 $$ 2065&&only 803 $$ 804 $$ 970&&only 803 $$ 804 $$ 970 $$ 1085&&only 803 $$ 804 $$ 970 $$ 1607 $$ 1640&&only 803 $$ 970&&only 807 $$ 4516&&only 809 $$ 7037&&only 811 $$ 2718&&only 811 $$ 4431&&only 811 $$ 4447&&only 811 $$ 8457&&only 811 $$ 13322&&only 811 $$ 14922&&only 813 $$ 2536&&only 813 $$ 10835&&only 814 $$ 895 $$ 1028&&only 814 $$ 1028 $$ 12219&&only 814 $$ 1028&&only 818 $$ 1450&&only 818 $$ 1450 $$ 1554&&only 818 $$ 1554&&only 819 $$ 844 $$ 2469&&only 821 $$ 11888&&only 822 $$ 1251&&only 825 $$ 826 $$ 827 $$ 983 $$ 1095 $$ 1707&&only 825 $$ 826 $$ 827 $$ 983 $$ 1094 $$ 1095 $$ 1451 $$ 1707&&only 825 $$ 826 $$ 827 $$ 983 $$ 1095 $$ 7507&&only 825 $$ 826 $$ 827 $$ 983 $$ 1095 $$ 4278&&only 825 $$ 826 $$ 827 $$ 983 $$ 1094 $$ 1095 $$ 1707 $$ 16859&&only 825 $$ 826 $$ 827 $$ 983 $$ 1095 $$ 8169&&only 825 $$ 1094 $$ 1451 $$ 1707&&only 826 $$ 827 $$ 983 $$ 1095&&only 828 $$ 830&&only 828 $$ 1718&&only 830 $$ 14278&&only 830 $$ 16913&&only 832 $$ 1147 $$ 2635&&only 832 $$ 2150&&only 833 $$ 16924&&only 834 $$ 1264&&only 835 $$ 993&&only 837 $$ 992&&only 837 $$ 992 $$ 3771&&only 839 $$ 1415&&only 839 $$ 4264 $$ 7412&&only 839 $$ 4410 $$ 14114&&only 840 $$ 1262 $$ 1687&&only 840 $$ 1687&&only 840 $$ 1687 $$ 4971&&only 840 $$ 2167&&only 840 $$ 5704&&only 841 $$ 1762&&only 843 $$ 1264&&only 843 $$ 14692&&only 843 $$ 16329&&only 844 $$ 3578&&only 845 $$ 997 $$ 1604 $$ 2469&&only 845 $$ 997 $$ 3652 $$ 13275&&only 845 $$ 997 $$ 998 $$ 1179&&only 845 $$ 1559&&only 845 $$ 6305&&only 846 $$ 10876 $$ 15066&&only 848 $$ 2231&&only 853 $$ 1004&&only 854 $$ 6362&&only 854 $$ 14989&&only 855 $$ 1967&&only 857 $$ 1787 $$ 1906 $$ 3310&&only 857 $$ 1906&&only 857 $$ 2058 $$ 3326&&only 857 $$ 3326&&only 858 $$ 1010&&only 858 $$ 1010 $$ 2860 $$ 4221&&only 858 $$ 1010 $$ 4221&&only 859 $$ 1011&&only 859 $$ 1824&&only 859 $$ 3139&&only 859 $$ 3267&&only 860 $$ 909 $$ 1161&&only 860 $$ 909&&only 860 $$ 909 $$ 4935&&only 862 $$ 3020&&only 862 $$ 14135&&only 862 $$ 14494&&only 863 $$ 3014&&only 863 $$ 5019&&only 864 $$ 6689&&only 864 $$ 7928&&only 864 $$ 11473&&only 865 $$ 15004&&only 866 $$ 1119&&only 869 $$ 2035&&only 869 $$ 2771&&only 871 $$ 10505&&only 873 $$ 1937 $$ 1955 $$ 2106&&only 873 $$ 2106&&only 873 $$ 4006&&only 874 $$ 5531&&only 875 $$ 1691&&only 877 $$ 954 $$ 1792&&only 877 $$ 954&&only 879 $$ 3048&&only 879 $$ 3316&&only 879 $$ 7353&&only 879 $$ 13196&&only 880 $$ 881 $$ 13197&&only 880 $$ 881 $$ 4035&&only 881 $$ 3330&&only 881 $$ 4035&&only 881 $$ 15129&&only 882 $$ 9541 $$ 12198&&only 883 $$ 884 $$ 1225&&only 883 $$ 1024 $$ 1025&&only 883 $$ 1225&&only 884 $$ 1225&&only 885 $$ 985&&only 885 $$ 985 $$ 2961&&only 886 $$ 1550&&only 886 $$ 16346&&only 886 $$ 16803&&only 887 $$ 12210&&only 888 $$ 2041&&only 888 $$ 6344&&only 889 $$ 1155&&only 889 $$ 1972&&only 891 $$ 1795&&only 891 $$ 14336&&only 892 $$ 1325&&only 893 $$ 898 $$ 899 $$ 1190&&only 894 $$ 1561&&only 897 $$ 1702&&only 898 $$ 899 $$ 1190&&only 899 $$ 1190&&only 900 $$ 1032&&only 901 $$ 1583&&only 902 $$ 1258 $$ 1518 $$ 1581 $$ 2885&&only 902 $$ 1518 $$ 1581&&only 902 $$ 1518 $$ 1581 $$ 2885&&only 902 $$ 1518&&only 902 $$ 1518 $$ 2885&&only 902 $$ 8118&&only 903 $$ 1209&&only 905 $$ 1047&&only 906 $$ 9402&&only 907 $$ 1039&&only 907 $$ 1039 $$ 14939&&only 908 $$ 1161&&only 909 $$ 1161&&only 910 $$ 1043&&only 911 $$ 9190&&only 912 $$ 1276&&only 913 $$ 8516&&only 915 $$ 1448 $$ 5352&&only 916 $$ 1326&&only 916 $$ 1326 $$ 6562&&only 916 $$ 7084&&only 917 $$ 1045&&only 919 $$ 1883&&only 919 $$ 3692&&only 920 $$ 5418&&only 920 $$ 9812&&only 920 $$ 12327&&only 923 $$ 927 $$ 1379&&only 923 $$ 1379&&only 926 $$ 1047&&only 926 $$ 12338&&only 927 $$ 2515&&only 928 $$ 1648&&only 931 $$ 1054 $$ 1482&&only 931 $$ 1056&&only 931 $$ 16173&&only 932 $$ 2222&&only 932 $$ 2671&&only 932 $$ 4682&&only 932 $$ 5052&&only 934 $$ 935 $$ 1791&&only 934 $$ 3329&&only 934 $$ 4051&&only 935 $$ 1791&&only 935 $$ 3459&&only 935 $$ 4058&&only 936 $$ 1001&&only 936 $$ 1001 $$ 1918&&only 936 $$ 1001 $$ 9368&&only 937 $$ 1350&&only 937 $$ 4963&&only 937 $$ 5519&&only 938 $$ 1523&&only 938 $$ 1598&&only 939 $$ 1237&&only 941 $$ 1204&&only 942 $$ 1369&&only 942 $$ 5969&&only 944 $$ 1564&&only 944 $$ 1564 $$ 5881&&only 944 $$ 4375&&only 945 $$ 1142&&only 946 $$ 947&&only 946 $$ 947 $$ 1278 $$ 13117&&only 946 $$ 947 $$ 1278&&only 947 $$ 1278&&only 948 $$ 1267&&only 949 $$ 7456&&only 950 $$ 951&&only 950 $$ 951 $$ 2919&&only 953 $$ 1667&&only 955 $$ 1360&&only 955 $$ 7722&&only 955 $$ 9576&&only 956 $$ 7045&&only 958 $$ 2562&&only 958 $$ 7351&&only 960 $$ 2817&&only 960 $$ 12264&&only 961 $$ 1470&&only 961 $$ 4099&&only 965 $$ 1079&&only 965 $$ 3186&&only 966 $$ 1979&&only 969 $$ 1761 $$ 11836&&only 969 $$ 1761&&only 969 $$ 1761 $$ 13315&&only 969 $$ 2648&&only 971 $$ 1340&&only 972 $$ 1054&&only 973 $$ 16760&&only 974 $$ 13320&&only 975 $$ 1089 $$ 2246&&only 975 $$ 1089 $$ 2218&&only 975 $$ 1089&&only 976 $$ 1638&&only 977 $$ 1400&&only 977 $$ 1400 $$ 9545&&only 977 $$ 2876&&only 977 $$ 2876 $$ 11626&&only 977 $$ 7066&&only 977 $$ 13300&&only 978 $$ 16479&&only 979 $$ 2581&&only 980 $$ 1995 $$ 4274 $$ 11857&&only 980 $$ 1995&&only 980 $$ 1995 $$ 17271&&only 980 $$ 1995 $$ 11692&&only 982 $$ 1281&&only 982 $$ 9351&&only 986 $$ 7224&&only 987 $$ 1669 $$ 3063&&only 987 $$ 2780&&only 987 $$ 6863&&only 988 $$ 3828&&only 988 $$ 5777&&only 990 $$ 1299&&only 990 $$ 1299 $$ 1459&&only 993 $$ 2802&&only 994 $$ 1737&&only 995 $$ 996&&only 997 $$ 1005&&only 997 $$ 1731&&only 998 $$ 1454 $$ 12019&&only 998 $$ 3874 $$ 14180&&only 998 $$ 16459&&only 999 $$ 1267&&only 999 $$ 1343 $$ 1476&&only 999 $$ 1343 $$ 1476 $$ 1639&&only 999 $$ 1343 $$ 1476 $$ 2851&&only 999 $$ 1476&&only 1000 $$ 3211&&only 1000 $$ 7506&&only 1003 $$ 9140&&only 1005 $$ 1123&&only 1005 $$ 3160&&only 1007 $$ 1109 $$ 12122&&only 1011 $$ 1641&&only 1013 $$ 4836&&only 1014 $$ 1015 $$ 1115 $$ 1116&&only 1014 $$ 1015 $$ 1115&&only 1014 $$ 1115&&only 1015 $$ 1116&&only 1016 $$ 1730&&only 1016 $$ 12152&&only 1023 $$ 1436&&only 1023 $$ 4533&&only 1023 $$ 8209&&only 1024 $$ 1025&&only 1027 $$ 2377&&only 1028 $$ 1189&&only 1028 $$ 3193&&only 1031 $$ 5413&&only 1032 $$ 1870&&only 1033 $$ 2114&&only 1034 $$ 1882 $$ 3367&&only 1034 $$ 16609&&only 1036 $$ 1764&&only 1036 $$ 3368&&only 1040 $$ 2774&&only 1042 $$ 1278&&only 1042 $$ 1941&&only 1042 $$ 3473&&only 1046 $$ 1194 $$ 1306 $$ 1307 $$ 1480&&only 1046 $$ 1194 $$ 1306 $$ 1307 $$ 3827&&only 1046 $$ 1307&&only 1046 $$ 13483&&only 1047 $$ 1562&&only 1050 $$ 10490&&only 1050 $$ 15146&&only 1051 $$ 1052 $$ 1196 $$ 1295&&only 1051 $$ 1196 $$ 1295&&only 1051 $$ 1295&&only 1053 $$ 4394&&only 1054 $$ 1466 $$ 1484 $$ 1527&&only 1055 $$ 6206&&only 1055 $$ 12347&&only 1056 $$ 2055&&only 1057 $$ 1885&&only 1057 $$ 2149&&only 1059 $$ 2145 $$ 5040&&only 1059 $$ 2145&&only 1059 $$ 2558 $$ 12350&&only 1059 $$ 2558 $$ 13123&&only 1059 $$ 2558 $$ 6750&&only 1059 $$ 2558 $$ 3926 $$ 10906&&only 1059 $$ 2558&&only 1059 $$ 2558 $$ 4204&&only 1059 $$ 3760 $$ 3926&&only 1059 $$ 3760&&only 1060 $$ 1612&&only 1060 $$ 2167&&only 1061 $$ 1767 $$ 1802&&only 1061 $$ 4377&&only 1062 $$ 1445&&only 1062 $$ 1495&&only 1062 $$ 1495 $$ 7394&&only 1062 $$ 5748&&only 1066 $$ 1725&&only 1066 $$ 1725 $$ 2948&&only 1067 $$ 1153&&only 1067 $$ 1153 $$ 1865&&only 1067 $$ 1153 $$ 1443&&only 1068 $$ 1427&&only 1070 $$ 4047&&only 1071 $$ 16281&&only 1072 $$ 1377&&only 1073 $$ 1156&&only 1073 $$ 1156 $$ 12813&&only 1073 $$ 1156 $$ 7756&&only 1073 $$ 1156 $$ 3931&&only 1073 $$ 1156 $$ 6629&&only 1073 $$ 1156 $$ 1699 $$ 2573&&only 1074 $$ 7813&&only 1074 $$ 10765&&only 1075 $$ 1748&&only 1076 $$ 1160&&only 1078 $$ 1256&&only 1078 $$ 10841&&only 1078 $$ 11823&&only 1078 $$ 13700&&only 1078 $$ 14882&&only 1080 $$ 1578 $$ 13295&&only 1080 $$ 1681&&only 1080 $$ 1681 $$ 2493&&only 1080 $$ 3273&&only 1083 $$ 2533 $$ 13695&&only 1083 $$ 8370&&only 1083 $$ 11592&&only 1083 $$ 11837&&only 1083 $$ 16895&&only 1084 $$ 1186 $$ 1813&&only 1084 $$ 1186&&only 1084 $$ 1682 $$ 2367&&only 1084 $$ 1813&&only 1084 $$ 15047&&only 1085 $$ 8731&&only 1085 $$ 9163&&only 1086 $$ 4187&&only 1086 $$ 14925 $$ 14926&&only 1087 $$ 4341&&only 1087 $$ 5865&&only 1087 $$ 7568&&only 1087 $$ 8351&&only 1087 $$ 15432&&only 1090 $$ 3641&&only 1091 $$ 3897&&only 1092 $$ 1676 $$ 3713&&only 1092 $$ 1676 $$ 7508&&only 1092 $$ 1676 $$ 4185&&only 1092 $$ 1676 $$ 14277&&only 1092 $$ 1676 $$ 14913&&only 1092 $$ 1676 $$ 8002&&only 1092 $$ 1676 $$ 15881&&only 1092 $$ 1676 $$ 3375&&only 1092 $$ 3375&&only 1092 $$ 3713&&only 1092 $$ 4185 $$ 14621&&only 1092 $$ 7764&&only 1092 $$ 13326&&only 1092 $$ 13471 $$ 16240&&only 1093 $$ 2962&&only 1093 $$ 5465&&only 1094 $$ 1451&&only 1094 $$ 5114 $$ 7997&&only 1094 $$ 5114&&only 1098 $$ 1810 $$ 11175&&only 1099 $$ 1101&&only 1099 $$ 2612&&only 1099 $$ 14952&&only 1100 $$ 1764&&only 1100 $$ 13249&&only 1101 $$ 1922&&only 1101 $$ 6307&&only 1101 $$ 13280&&only 1102 $$ 1176&&only 1102 $$ 1176 $$ 13745&&only 1102 $$ 2264&&only 1102 $$ 4402&&only 1104 $$ 12024&&only 1106 $$ 1417 $$ 1456 $$ 7097&&only 1106 $$ 1417 $$ 1456 $$ 7129&&only 1106 $$ 1863&&only 1109 $$ 3023&&only 1109 $$ 4116&&only 1111 $$ 14695&&only 1117 $$ 1221&&only 1118 $$ 1730&&only 1124 $$ 1534&&only 1125 $$ 3131 $$ 9293&&only 1126 $$ 3005&&only 1128 $$ 1270&&only 1129 $$ 2257&&only 1130 $$ 1131 $$ 1324&&only 1130 $$ 1324 $$ 1419&&only 1131 $$ 5478&&only 1131 $$ 5620&&only 1131 $$ 7972&&only 1132 $$ 1273 $$ 1373&&only 1132 $$ 1273&&only 1132 $$ 1273 $$ 7804&&only 1133 $$ 12830&&only 1136 $$ 7033&&only 1137 $$ 1304&&only 1138 $$ 4026&&only 1140 $$ 2716&&only 1142 $$ 1151&&only 1144 $$ 1308&&only 1144 $$ 1308 $$ 3040&&only 1144 $$ 3040&&only 1145 $$ 1146&&only 1145 $$ 1146 $$ 1884&&only 1146 $$ 1884&&only 1147 $$ 13726&&only 1149 $$ 2373&&only 1149 $$ 14781&&only 1151 $$ 1426&&only 1151 $$ 12513&&only 1152 $$ 1356&&only 1153 $$ 1443&&only 1155 $$ 1565&&only 1158 $$ 3820&&only 1158 $$ 3890&&only 1158 $$ 4859&&only 1158 $$ 5467&&only 1158 $$ 7113&&only 1158 $$ 8108&&only 1158 $$ 10944&&only 1158 $$ 13348&&only 1160 $$ 6202&&only 1161 $$ 5342&&only 1164 $$ 1266&&only 1164 $$ 3318&&only 1166 $$ 11855&&only 1168 $$ 2305&&only 1168 $$ 11890&&only 1169 $$ 1296&&only 1171 $$ 3571 $$ 3718 $$ 4500&&only 1171 $$ 11484&&only 1172 $$ 1179&&only 1172 $$ 1388&&only 1173 $$ 9844&&only 1174 $$ 2302 $$ 7236&&only 1174 $$ 11991&&only 1175 $$ 3320 $$ 5030&&only 1175 $$ 3430&&only 1175 $$ 5653&&only 1177 $$ 2226 $$ 10732&&only 1177 $$ 10854&&only 1179 $$ 1499 $$ 1663&&only 1180 $$ 4946&&only 1180 $$ 14302&&only 1180 $$ 14647&&only 1182 $$ 2216&&only 1182 $$ 6655 $$ 17189&&only 1183 $$ 1222&&only 1184 $$ 1288&&only 1184 $$ 1679&&only 1184 $$ 3401&&only 1184 $$ 3401 $$ 8902&&only 1184 $$ 7739&&only 1184 $$ 8255&&only 1186 $$ 1813 $$ 12204&&only 1186 $$ 1813&&only 1187 $$ 1345&&only 1187 $$ 6523&&only 1188 $$ 4979&&only 1192 $$ 1891 $$ 5412 $$ 5842&&only 1192 $$ 1891 $$ 2051&&only 1192 $$ 1891 $$ 2051 $$ 3919&&only 1192 $$ 1891 $$ 3919 $$ 6456&&only 1192 $$ 1891 $$ 2228 $$ 15232 $$ 15233&&only 1192 $$ 1891 $$ 4130 $$ 4234&&only 1192 $$ 2051 $$ 2404 $$ 2673&&only 1192 $$ 2673&&only 1194 $$ 1306 $$ 1480&&only 1194 $$ 1306 $$ 1307 $$ 1480&&only 1194 $$ 1306 $$ 1480 $$ 12325&&only 1194 $$ 1306 $$ 1307 $$ 3827&&only 1194 $$ 1306 $$ 3827&&only 1194 $$ 1480&&only 1194 $$ 3827&&only 1198 $$ 1838&&only 1198 $$ 3654&&only 1201 $$ 1350&&only 1201 $$ 1350 $$ 1523&&only 1202 $$ 1404&&only 1203 $$ 13796&&only 1207 $$ 1866&&only 1207 $$ 2483&&only 1208 $$ 1952&&only 1209 $$ 1461&&only 1210 $$ 5370&&only 1212 $$ 1422&&only 1214 $$ 4328&&only 1216 $$ 6556&&only 1227 $$ 2668&&only 1232 $$ 3022&&only 1238 $$ 13589&&only 1239 $$ 3742&&only 1244 $$ 5862&&only 1244 $$ 9194&&only 1245 $$ 1849 $$ 2795&&only 1246 $$ 6909&&only 1246 $$ 6969&&only 1246 $$ 14818&&only 1250 $$ 2254 $$ 6962 $$ 7610&&only 1250 $$ 4565&&only 1250 $$ 5095&&only 1250 $$ 6423&&only 1250 $$ 7012&&only 1250 $$ 7515&&only 1250 $$ 11193&&only 1253 $$ 6321&&only 1253 $$ 11818&&only 1255 $$ 1934&&only 1259 $$ 13586&&only 1261 $$ 1390&&only 1261 $$ 3947&&only 1261 $$ 4746&&only 1262 $$ 3477&&only 1264 $$ 10275&&only 1266 $$ 1692&&only 1268 $$ 1268&&only 1268 $$ 1927&&only 1268 $$ 3725&&only 1269 $$ 2519&&only 1271 $$ 1940&&only 1271 $$ 3138&&only 1271 $$ 6135&&only 1272 $$ 1908 $$ 3050&&only 1272 $$ 1908 $$ 7704&&only 1272 $$ 6288&&only 1274 $$ 1303 $$ 12278&&only 1274 $$ 1776&&only 1275 $$ 1789 $$ 5125&&only 1275 $$ 1789 $$ 8690&&only 1275 $$ 2630 $$ 5934&&only 1276 $$ 5994&&only 1277 $$ 3956&&only 1277 $$ 8924&&only 1280 $$ 2320&&only 1280 $$ 3685&&only 1280 $$ 6190&&only 1280 $$ 7399&&only 1280 $$ 13204&&only 1282 $$ 8357&&only 1282 $$ 15756&&only 1283 $$ 1829 $$ 2056&&only 1285 $$ 1286&&only 1287 $$ 1411&&only 1287 $$ 7540&&only 1288 $$ 5844&&only 1289 $$ 14671&&only 1289 $$ 16897&&only 1290 $$ 1472&&only 1290 $$ 1472 $$ 1473&&only 1291 $$ 7137&&only 1292 $$ 11893&&only 1293 $$ 9214&&only 1293 $$ 13132&&only 1294 $$ 1351&&only 1294 $$ 1351 $$ 2560&&only 1297 $$ 1704&&only 1298 $$ 1439 $$ 1922&&only 1298 $$ 1922&&only 1298 $$ 3435&&only 1300 $$ 15127&&only 1301 $$ 9339&&only 1301 $$ 14798&&only 1302 $$ 3140&&only 1305 $$ 5577&&only 1306 $$ 1307&&only 1307 $$ 7222&&only 1309 $$ 3361 $$ 5534&&only 1309 $$ 7738&&only 1311 $$ 1470&&only 1311 $$ 2025&&only 1311 $$ 2152&&only 1311 $$ 4099&&only 1311 $$ 7314&&only 1311 $$ 7953&&only 1313 $$ 1314&&only 1315 $$ 1618&&only 1315 $$ 1618 $$ 2415&&only 1315 $$ 1618 $$ 2008&&only 1316 $$ 1589 $$ 1710&&only 1317 $$ 1368&&only 1317 $$ 1385&&only 1318 $$ 1528&&only 1318 $$ 2174&&only 1318 $$ 2174 $$ 2475&&only 1318 $$ 2903&&only 1318 $$ 4117&&only 1322 $$ 2295&&only 1322 $$ 12686 $$ 12716&&only 1325 $$ 1432&&only 1325 $$ 2017&&only 1327 $$ 2708&&only 1327 $$ 4259&&only 1330 $$ 1605&&only 1330 $$ 5929&&only 1330 $$ 8693&&only 1330 $$ 8693 $$ 16971&&only 1330 $$ 10442&&only 1331 $$ 2649&&only 1331 $$ 6246&&only 1331 $$ 7091&&only 1331 $$ 7650&&only 1332 $$ 1364&&only 1332 $$ 1364 $$ 1502&&only 1332 $$ 1364 $$ 7593&&only 1332 $$ 1502&&only 1333 $$ 2119 $$ 4988&&only 1333 $$ 2119 $$ 3988&&only 1333 $$ 2119 $$ 3988 $$ 10604&&only 1333 $$ 2119 $$ 4988 $$ 6406&&only 1333 $$ 2938 $$ 3707&&only 1336 $$ 1658&&only 1336 $$ 4556&&only 1337 $$ 1530 $$ 1994 $$ 2288 $$ 10923&&only 1337 $$ 1885 $$ 2288 $$ 13771&&only 1337 $$ 1994&&only 1337 $$ 1994 $$ 2288 $$ 6174&&only 1337 $$ 1994 $$ 2288&&only 1337 $$ 2288&&only 1339 $$ 14967&&only 1343 $$ 1476 $$ 1670&&only 1343 $$ 1476&&only 1343 $$ 1607&&only 1343 $$ 5435&&only 1344 $$ 4215 $$ 11158&&only 1346 $$ 1610&&only 1346 $$ 1610 $$ 3773&&only 1346 $$ 7944&&only 1346 $$ 12979&&only 1346 $$ 14340&&only 1347 $$ 7992&&only 1348 $$ 3829 $$ 17023&&only 1349 $$ 9743 $$ 10309&&only 1352 $$ 1666 $$ 12383&&only 1353 $$ 13415&&only 1354 $$ 3499&&only 1354 $$ 5213&&only 1354 $$ 10455&&only 1355 $$ 1481&&only 1355 $$ 9457&&only 1356 $$ 13116&&only 1358 $$ 2000&&only 1359 $$ 1609 $$ 11184&&only 1360 $$ 1772&&only 1360 $$ 1772 $$ 1933&&only 1360 $$ 6352&&only 1362 $$ 17306&&only 1363 $$ 3107&&only 1364 $$ 3494&&only 1365 $$ 5044&&only 1367 $$ 2207&&only 1369 $$ 6220&&only 1369 $$ 7168&&only 1370 $$ 1500 $$ 2073 $$ 5439&&only 1370 $$ 1500 $$ 1501 $$ 1544 $$ 2073 $$ 5439&&only 1370 $$ 1500 $$ 1501 $$ 1544 $$ 2073 $$ 12654 $$ 13014&&only 1370 $$ 1500 $$ 1501 $$ 1544 $$ 1671 $$ 2073 $$ 3352&&only 1370 $$ 1500 $$ 1501 $$ 1544 $$ 2073 $$ 3352&&only 1370 $$ 1500 $$ 4008 $$ 4468&&only 1370 $$ 1500 $$ 1501 $$ 1544 $$ 2073 $$ 8065&&only 1370 $$ 1500 $$ 1501 $$ 1544 $$ 2073 $$ 17364&&only 1370 $$ 2073 $$ 3584&&only 1370 $$ 4744 $$ 6397&&only 1370 $$ 16674&&only 1371 $$ 2992&&only 1371 $$ 11575&&only 1372 $$ 6162&&only 1374 $$ 2292&&only 1374 $$ 3043&&only 1374 $$ 9557&&only 1374 $$ 12269&&only 1377 $$ 4514&&only 1378 $$ 1498&&only 1378 $$ 2137 $$ 2138 $$ 2139 $$ 2140 $$ 2141 $$ 2180 $$ 2181&&only 1380 $$ 1659&&only 1380 $$ 3308 $$ 3309&&only 1380 $$ 4742&&only 1380 $$ 11995&&only 1381 $$ 1481&&only 1381 $$ 4026&&only 1383 $$ 1860 $$ 1880 $$ 1909 $$ 4524&&only 1386 $$ 2216&&only 1386 $$ 5253&&only 1387 $$ 2132&&only 1389 $$ 3332&&only 1389 $$ 4042&&only 1389 $$ 4314&&only 1390 $$ 2427&&only 1390 $$ 8003&&only 1393 $$ 1455 $$ 12109&&only 1398 $$ 2263 $$ 5731&&only 1398 $$ 2263 $$ 4037&&only 1399 $$ 2096&&only 1400 $$ 3615&&only 1402 $$ 3363 $$ 4195&&only 1402 $$ 3955&&only 1402 $$ 4132&&only 1402 $$ 4195&&only 1402 $$ 5051 $$ 5747&&only 1402 $$ 7940&&only 1402 $$ 8224&&only 1402 $$ 9033&&only 1402 $$ 13146 $$ 13147&&only 1403 $$ 3041&&only 1404 $$ 4328&&only 1406 $$ 1807&&only 1406 $$ 5623&&only 1408 $$ 6890&&only 1409 $$ 2123&&only 1409 $$ 3076 $$ 15302&&only 1409 $$ 12754&&only 1409 $$ 14448&&only 1410 $$ 1784&&only 1411 $$ 1821&&only 1417 $$ 1456&&only 1419 $$ 1885 $$ 12390&&only 1421 $$ 2565&&only 1422 $$ 1441 $$ 5078&&only 1422 $$ 5078&&only 1424 $$ 4257&&only 1425 $$ 2991&&only 1425 $$ 3059 $$ 3103&&only 1425 $$ 3059&&only 1425 $$ 3059 $$ 3103 $$ 4754&&only 1425 $$ 3224&&only 1426 $$ 2026&&only 1427 $$ 5235&&only 1428 $$ 4812&&only 1428 $$ 11651&&only 1430 $$ 1549&&only 1430 $$ 1549 $$ 13789&&only 1430 $$ 1658&&only 1430 $$ 10929&&only 1431 $$ 7125&&only 1431 $$ 7862&&only 1431 $$ 9022&&only 1432 $$ 2990&&only 1432 $$ 3816&&only 1433 $$ 11685&&only 1434 $$ 2163&&only 1434 $$ 13694&&only 1436 $$ 6183&&only 1437 $$ 2660&&only 1438 $$ 2828&&only 1439 $$ 5414&&only 1439 $$ 10285&&only 1439 $$ 15858&&only 1440 $$ 1799&&only 1441 $$ 1914&&only 1442 $$ 16345&&only 1446 $$ 3616&&only 1446 $$ 6524&&only 1447 $$ 1467 $$ 1471 $$ 1485&&only 1447 $$ 1467 $$ 1471 $$ 1485 $$ 1488 $$ 1503 $$ 1504 $$ 1505 $$ 1506 $$ 1594 $$ 1755&&only 1447 $$ 1467 $$ 1471 $$ 1485 $$ 1488 $$ 1504 $$ 1505 $$ 1506 $$ 1755&&only 1447 $$ 1467 $$ 1471 $$ 1485 $$ 1488 $$ 1505 $$ 1506 $$ 1755&&only 1447 $$ 1467 $$ 1471 $$ 1485 $$ 1488 $$ 1503 $$ 1504 $$ 1505 $$ 1506 $$ 1755&&only 1447 $$ 1467 $$ 1471 $$ 1485 $$ 1488 $$ 1504 $$ 1505 $$ 1506 $$ 6854&&only 1447 $$ 1467 $$ 1471 $$ 1485 $$ 1488 $$ 1503 $$ 1504 $$ 1505 $$ 1506 $$ 6854&&only 1447 $$ 1471 $$ 1485&&only 1454 $$ 16968&&only 1455 $$ 4924&&only 1455 $$ 5616&&only 1455 $$ 6656&&only 1455 $$ 7664&&only 1455 $$ 7665&&only 1455 $$ 11137&&only 1455 $$ 11276&&only 1455 $$ 11502&&only 1455 $$ 14592&&only 1455 $$ 15623&&only 1460 $$ 1468 $$ 1469&&only 1460 $$ 1468 $$ 1469 $$ 3479&&only 1460 $$ 1468&&only 1462 $$ 3171&&only 1463 $$ 1795&&only 1463 $$ 13155&&only 1466 $$ 1484 $$ 1527&&only 1466 $$ 1484&&only 1467 $$ 1488 $$ 1504 $$ 1505 $$ 1506 $$ 1755&&only 1467 $$ 1488 $$ 1505 $$ 1506 $$ 1755&&only 1470 $$ 4548&&only 1474 $$ 2219&&only 1474 $$ 3573&&only 1476 $$ 1694 $$ 3658 $$ 4376 $$ 8203&&only 1478 $$ 1788&&only 1479 $$ 10635&&only 1482 $$ 14147&&only 1484 $$ 1527&&only 1487 $$ 2055 $$ 8846&&only 1488 $$ 1503 $$ 1504 $$ 1505 $$ 1506 $$ 1594 $$ 6320&&only 1488 $$ 1504 $$ 1505 $$ 1506 $$ 6320&&only 1489 $$ 1520 $$ 11275&&only 1489 $$ 1520 $$ 11460 $$ 16536&&only 1489 $$ 9375&&only 1489 $$ 12009&&only 1489 $$ 12570&&only 1489 $$ 16234&&only 1490 $$ 8752&&only 1492 $$ 1492&&only 1492 $$ 1808&&only 1492 $$ 4824&&only 1494 $$ 2161 $$ 3908&&only 1494 $$ 11474&&only 1494 $$ 16814&&only 1496 $$ 7842&&only 1497 $$ 1521 $$ 1536 $$ 1541 $$ 1542 $$ 1557 $$ 1584 $$ 1814 $$ 2004&&only 1497 $$ 1521 $$ 1541 $$ 1542 $$ 1557 $$ 1584 $$ 1814 $$ 7599&&only 1497 $$ 1521 $$ 1536 $$ 1541 $$ 1542 $$ 1557 $$ 1584 $$ 3854 $$ 4147&&only 1497 $$ 1536 $$ 1584 $$ 1814 $$ 2004&&only 1497 $$ 1584&&only 1498 $$ 2047&&only 1499 $$ 1663&&only 1500 $$ 1544 $$ 5160&&only 1500 $$ 1544&&only 1501 $$ 1544&&only 1503 $$ 1504 $$ 1594&&only 1503 $$ 1504&&only 1503 $$ 1594&&only 1507 $$ 1507 $$ 2099&&only 1507 $$ 2099 $$ 11896&&only 1507 $$ 2099&&only 1507 $$ 8811 $$ 9337&&only 1512 $$ 4942&&only 1514 $$ 1808&&only 1514 $$ 3440&&only 1516 $$ 12217&&only 1519 $$ 6331&&only 1521 $$ 1536 $$ 1541 $$ 1542 $$ 1557 $$ 1814 $$ 2004&&only 1521 $$ 1541 $$ 1542 $$ 1557&&only 1525 $$ 2186&&only 1525 $$ 4193&&only 1526 $$ 2946&&only 1526 $$ 3472&&only 1529 $$ 1983&&only 1529 $$ 4296&&only 1529 $$ 10791&&only 1530 $$ 4010&&only 1530 $$ 6427&&only 1530 $$ 8149&&only 1532 $$ 3122&&only 1532 $$ 3568&&only 1535 $$ 3258&&only 1538 $$ 4575&&only 1538 $$ 5980&&only 1543 $$ 2612&&only 1545 $$ 1624 $$ 1827&&only 1547 $$ 1843&&only 1548 $$ 1801 $$ 1823 $$ 1893&&only 1549 $$ 9005&&only 1550 $$ 7282&&only 1553 $$ 3221&&only 1553 $$ 17339&&only 1555 $$ 1752&&only 1555 $$ 6297&&only 1556 $$ 8523&&only 1559 $$ 12117&&only 1560 $$ 1862&&only 1561 $$ 1627&&only 1563 $$ 2193&&only 1566 $$ 1567 $$ 1586 $$ 1736&&only 1566 $$ 1586 $$ 1736&&only 1566 $$ 1586&&only 1566 $$ 1736&&only 1568 $$ 3350&&only 1568 $$ 7859&&only 1569 $$ 1705&&only 1570 $$ 2265&&only 1571 $$ 2417&&only 1571 $$ 2417 $$ 3315&&only 1571 $$ 2417 $$ 3315 $$ 5367&&only 1571 $$ 2527&&only 1572 $$ 10471&&only 1573 $$ 2647&&only 1573 $$ 9111&&only 1573 $$ 16777&&only 1577 $$ 1577&&only 1578 $$ 1681&&only 1578 $$ 2817&&only 1583 $$ 2351&&only 1587 $$ 2054&&only 1589 $$ 1710&&only 1589 $$ 1710 $$ 3170&&only 1590 $$ 9198&&only 1595 $$ 1848 $$ 2232&&only 1598 $$ 2197&&only 1604 $$ 4217&&only 1606 $$ 16907&&only 1607 $$ 1640&&only 1608 $$ 2892&&only 1608 $$ 4081&&only 1608 $$ 8983&&only 1612 $$ 1822&&only 1613 $$ 5496&&only 1614 $$ 1873&&only 1614 $$ 5766&&only 1616 $$ 2045 $$ 2160 $$ 2225 $$ 2233&&only 1616 $$ 2045 $$ 2160&&only 1616 $$ 2045 $$ 2160 $$ 2233&&only 1616 $$ 2160&&only 1617 $$ 6925&&only 1620 $$ 1655&&only 1621 $$ 1651 $$ 2240&&only 1623 $$ 2028 $$ 11937&&only 1623 $$ 2028 $$ 14388&&only 1623 $$ 2028 $$ 15238&&only 1623 $$ 2028 $$ 8711&&only 1623 $$ 2028 $$ 9277&&only 1623 $$ 2028 $$ 11938&&only 1623 $$ 2028 $$ 16189&&only 1623 $$ 4282 $$ 5221&&only 1623 $$ 6283&&only 1623 $$ 9277&&only 1625 $$ 2715&&only 1625 $$ 4311&&only 1625 $$ 5073&&only 1626 $$ 12023&&only 1629 $$ 1653 $$ 1743 $$ 1744 $$ 13149&&only 1629 $$ 1653 $$ 1743 $$ 1771 $$ 2294&&only 1629 $$ 1653 $$ 1743 $$ 1744 $$ 1771 $$ 2294 $$ 7715&&only 1629 $$ 1743 $$ 1744 $$ 7850&&only 1630 $$ 2357 $$ 2917 $$ 5085 $$ 5644&&only 1630 $$ 2917 $$ 15931&&only 1632 $$ 1832&&only 1632 $$ 9929&&only 1633 $$ 1634&&only 1635 $$ 2044 $$ 2049 $$ 2355&&only 1635 $$ 2049&&only 1635 $$ 12828&&only 1635 $$ 14198&&only 1638 $$ 8914&&only 1643 $$ 9873&&only 1645 $$ 1646 $$ 1916 $$ 1946 $$ 2568 $$ 5744 $$ 5745 $$ 6169&&only 1645 $$ 1646 $$ 1916 $$ 5744 $$ 5745&&only 1645 $$ 1646 $$ 3603 $$ 4443&&only 1645 $$ 1646&&only 1645 $$ 1646 $$ 1916 $$ 9035 $$ 10493&&only 1645 $$ 1646 $$ 4473 $$ 9139&&only 1647 $$ 12569 $$ 17001&&only 1648 $$ 13141&&only 1649 $$ 1650&&only 1651 $$ 8337 $$ 8391 $$ 17520&&only 1652 $$ 3469&&only 1653 $$ 8892&&only 1659 $$ 10773&&only 1661 $$ 1729 $$ 14116&&only 1661 $$ 1729 $$ 14369&&only 1661 $$ 1729 $$ 9006&&only 1661 $$ 1729 $$ 14835&&only 1661 $$ 8017 $$ 9206&&only 1661 $$ 12116&&only 1661 $$ 14961&&only 1661 $$ 15886 $$ 15887&&only 1662 $$ 2285&&only 1664 $$ 9153&&only 1665 $$ 1790 $$ 1839 $$ 1977&&only 1665 $$ 1790 $$ 1839&&only 1669 $$ 3063&&only 1669 $$ 5180&&only 1669 $$ 5355&&only 1675 $$ 4074&&only 1675 $$ 4576&&only 1675 $$ 5855&&only 1675 $$ 6306&&only 1675 $$ 6842&&only 1675 $$ 8940&&only 1675 $$ 16191&&only 1676 $$ 3375&&only 1676 $$ 13470&&only 1680 $$ 1703&&only 1680 $$ 1703 $$ 2518 $$ 4266&&only 1680 $$ 1703 $$ 2518&&only 1680 $$ 1703 $$ 2518 $$ 2899 $$ 4266 $$ 4267 $$ 4490 $$ 7649 $$ 11701&&only 1682 $$ 1933&&only 1683 $$ 3690&&only 1684 $$ 2338&&only 1688 $$ 3118&&only 1693 $$ 1713 $$ 1717 $$ 1794&&only 1693 $$ 1713 $$ 1794&&only 1693 $$ 7809&&only 1694 $$ 2081 $$ 5062 $$ 5363&&only 1694 $$ 4044&&only 1694 $$ 4045&&only 1694 $$ 4045 $$ 4972&&only 1694 $$ 4376&&only 1694 $$ 5062 $$ 5363&&only 1694 $$ 5062 $$ 5363 $$ 12998&&only 1694 $$ 5363&&only 1694 $$ 14488&&only 1696 $$ 2107 $$ 3314&&only 1696 $$ 2107 $$ 4007&&only 1697 $$ 3015&&only 1697 $$ 3336&&only 1697 $$ 6573&&only 1697 $$ 7669&&only 1697 $$ 17278&&only 1698 $$ 6963 $$ 8563&&only 1699 $$ 2573 $$ 2791&&only 1699 $$ 2573 $$ 7667&&only 1699 $$ 2573&&only 1699 $$ 2573 $$ 2791 $$ 7951&&only 1699 $$ 2791 $$ 8441&&only 1699 $$ 4354&&only 1699 $$ 5269&&only 1704 $$ 14884&&only 1705 $$ 16856&&only 1706 $$ 9049&&only 1706 $$ 16157&&only 1709 $$ 3319&&only 1709 $$ 4525 $$ 12271&&only 1709 $$ 5324&&only 1709 $$ 12808&&only 1713 $$ 1717 $$ 1794&&only 1713 $$ 1717&&only 1720 $$ 1721&&only 1720 $$ 2079&&only 1720 $$ 2433&&only 1720 $$ 2433 $$ 2609&&only 1720 $$ 7732&&only 1728 $$ 1830&&only 1732 $$ 1733 $$ 1986 $$ 2018&&only 1732 $$ 1986 $$ 2018&&only 1732 $$ 2018&&only 1733 $$ 1986&&only 1735 $$ 1831 $$ 2064&&only 1735 $$ 1831 $$ 9473&&only 1735 $$ 2059 $$ 9560&&only 1735 $$ 2064&&only 1738 $$ 14363&&only 1741 $$ 2191&&only 1742 $$ 5034&&only 1742 $$ 9749&&only 1742 $$ 9750&&only 1744 $$ 7715&&only 1747 $$ 2637&&only 1750 $$ 3802&&only 1752 $$ 6113&&only 1756 $$ 5372&&only 1758 $$ 4782&&only 1758 $$ 4813&&only 1758 $$ 5770&&only 1758 $$ 6208&&only 1758 $$ 7405&&only 1758 $$ 8281&&only 1758 $$ 9725&&only 1763 $$ 8171&&only 1764 $$ 2429&&only 1765 $$ 2788&&only 1766 $$ 1925 $$ 2726 $$ 3247&&only 1766 $$ 1925&&only 1766 $$ 2726 $$ 7112&&only 1766 $$ 2726&&only 1766 $$ 2726 $$ 9406&&only 1766 $$ 3493&&only 1766 $$ 5900&&only 1766 $$ 6002&&only 1766 $$ 12098 $$ 12099 $$ 12100&&only 1767 $$ 1802&&only 1770 $$ 1861 $$ 2435 $$ 2436 $$ 2437 $$ 12251&&only 1770 $$ 1861 $$ 2435 $$ 2436 $$ 2437&&only 1770 $$ 1861&&only 1770 $$ 1861 $$ 2435 $$ 2436&&only 1770 $$ 1861 $$ 2436 $$ 2437&&only 1771 $$ 2294&&only 1771 $$ 4752&&only 1771 $$ 5725&&only 1772 $$ 1933&&only 1773 $$ 2269&&only 1773 $$ 6428&&only 1773 $$ 6429&&only 1773 $$ 13354&&only 1777 $$ 4887 $$ 5309&&only 1777 $$ 5309&&only 1779 $$ 1803&&only 1779 $$ 2596&&only 1779 $$ 16387&&only 1780 $$ 4826&&only 1780 $$ 14502&&only 1781 $$ 2654&&only 1781 $$ 11845&&only 1786 $$ 2033 $$ 2101 $$ 2135&&only 1786 $$ 2101&&only 1787 $$ 5459&&only 1787 $$ 7703&&only 1787 $$ 9512&&only 1791 $$ 10501&&only 1792 $$ 3233&&only 1792 $$ 4319&&only 1793 $$ 3111&&only 1793 $$ 8079&&only 1793 $$ 8117&&only 1793 $$ 11297&&only 1793 $$ 11358&&only 1793 $$ 17032&&only 1793 $$ 17498&&only 1799 $$ 4519&&only 1800 $$ 3803&&only 1800 $$ 4422&&only 1800 $$ 5851&&only 1800 $$ 6378&&only 1800 $$ 10566&&only 1800 $$ 13246&&only 1801 $$ 1823&&only 1801 $$ 1823 $$ 1893&&only 1804 $$ 1820 $$ 1828 $$ 1888 $$ 6252&&only 1804 $$ 1820 $$ 1828 $$ 1888 $$ 2052&&only 1804 $$ 1820 $$ 1828 $$ 2052&&only 1804 $$ 1820 $$ 2052&&only 1805 $$ 2061 $$ 2272&&only 1809 $$ 2234&&only 1812 $$ 1927&&only 1813 $$ 14871&&only 1815 $$ 7373&&only 1815 $$ 9805&&only 1817 $$ 2152&&only 1817 $$ 7521&&only 1817 $$ 8520&&only 1817 $$ 9746 $$ 9933&&only 1818 $$ 3153&&only 1818 $$ 5596&&only 1825 $$ 1915&&only 1831 $$ 2064&&only 1831 $$ 8373&&only 1832 $$ 2039&&only 1832 $$ 2039 $$ 15896&&only 1832 $$ 13762&&only 1833 $$ 2348&&only 1833 $$ 13562&&only 1835 $$ 2036 $$ 6245&&only 1835 $$ 2036&&only 1835 $$ 2107 $$ 12173 $$ 12174&&only 1835 $$ 2107&&only 1835 $$ 10146&&only 1837 $$ 2292&&only 1838 $$ 2263 $$ 8228&&only 1840 $$ 2123&&only 1841 $$ 3320&&only 1845 $$ 13330&&only 1849 $$ 2795&&only 1850 $$ 3210 $$ 8182&&only 1850 $$ 3984&&only 1850 $$ 5720&&only 1850 $$ 7693&&only 1850 $$ 9499 $$ 15445&&only 1850 $$ 16694&&only 1851 $$ 3833&&only 1852 $$ 2173&&only 1852 $$ 15156&&only 1854 $$ 6034&&only 1858 $$ 4435&&only 1860 $$ 1880 $$ 1909 $$ 6942&&only 1860 $$ 1880 $$ 1909 $$ 4524&&only 1860 $$ 1880 $$ 1909 $$ 2918&&only 1860 $$ 1909&&only 1860 $$ 1909 $$ 2918&&only 1862 $$ 8981&&only 1862 $$ 10448&&only 1862 $$ 14510&&only 1863 $$ 2591&&only 1864 $$ 2335 $$ 4750 $$ 5053 $$ 5723&&only 1865 $$ 2481&&only 1868 $$ 1931&&only 1869 $$ 4066&&only 1870 $$ 3663&&only 1872 $$ 1992&&only 1872 $$ 2642&&only 1872 $$ 2717&&only 1872 $$ 3950&&only 1873 $$ 3076&&only 1875 $$ 4889&&only 1876 $$ 4023&&only 1877 $$ 6207&&only 1878 $$ 14396&&only 1879 $$ 13535&&only 1880 $$ 2918&&only 1880 $$ 8216&&only 1882 $$ 3367&&only 1883 $$ 4570&&only 1884 $$ 2769&&only 1885 $$ 3560&&only 1886 $$ 3076&&only 1886 $$ 15304&&only 1887 $$ 2156&&only 1889 $$ 2015&&only 1889 $$ 2842&&only 1890 $$ 1920&&only 1892 $$ 2276&&only 1896 $$ 2188&&only 1897 $$ 5989&&only 1897 $$ 6096&&only 1898 $$ 3343&&only 1898 $$ 14211&&only 1902 $$ 11217&&only 1905 $$ 13396&&only 1908 $$ 3050&&only 1908 $$ 4125&&only 1908 $$ 7704&&only 1916 $$ 1946 $$ 2568 $$ 3452&&only 1916 $$ 1946 $$ 4472 $$ 5743&&only 1919 $$ 3235&&only 1925 $$ 2584 $$ 3247&&only 1927 $$ 4139&&only 1928 $$ 2666&&only 1928 $$ 2666 $$ 4601&&only 1932 $$ 11834&&only 1934 $$ 14486&&only 1936 $$ 11989&&only 1937 $$ 1955&&only 1937 $$ 1955 $$ 9876&&only 1942 $$ 1975 $$ 1976 $$ 5727 $$ 5931 $$ 5932 $$ 13362 $$ 13363&&only 1942 $$ 2003 $$ 4757 $$ 6961&&only 1942 $$ 2003 $$ 3513 $$ 5333&&only 1942 $$ 2003 $$ 5940 $$ 9242&&only 1942 $$ 2003 $$ 5940 $$ 6615&&only 1942 $$ 2003 $$ 3513 $$ 5728&&only 1944 $$ 12365&&only 1945 $$ 2076 $$ 6752&&only 1945 $$ 2076&&only 1945 $$ 5022&&only 1946 $$ 2568 $$ 9642&&only 1946 $$ 4472 $$ 7977&&only 1953 $$ 2010&&only 1957 $$ 2862 $$ 6295&&only 1961 $$ 2023 $$ 2057 $$ 2089 $$ 2131 $$ 11736 $$ 11737&&only 1963 $$ 3368&&only 1964 $$ 3717&&only 1964 $$ 6869&&only 1966 $$ 15617&&only 1967 $$ 2090&&only 1968 $$ 3085&&only 1968 $$ 13841&&only 1969 $$ 5769&&only 1973 $$ 7812&&only 1975 $$ 1976 $$ 5727 $$ 5931 $$ 5932&&only 1975 $$ 1976&&only 1975 $$ 1976 $$ 5931 $$ 5932&&only 1975 $$ 1976 $$ 2529 $$ 3836&&only 1975 $$ 1976 $$ 2529 $$ 8593&&only 1975 $$ 1976 $$ 2529 $$ 11580&&only 1980 $$ 15880&&only 1983 $$ 8974 $$ 14517&&only 1984 $$ 2747&&only 1984 $$ 2747 $$ 2747&&only 1984 $$ 4062&&only 2000 $$ 15757&&only 2002 $$ 6402&&only 2007 $$ 7483&&only 2007 $$ 8722&&only 2009 $$ 4027&&only 2009 $$ 4534&&only 2011 $$ 2393&&only 2011 $$ 2393 $$ 2463&&only 2011 $$ 2463 $$ 4803&&only 2011 $$ 4803&&only 2013 $$ 2120 $$ 2121 $$ 2122 $$ 2570 $$ 2571 $$ 2579 $$ 2657 $$ 2658 $$ 4569 $$ 5839&&only 2013 $$ 2579 $$ 2657 $$ 2658 $$ 5839&&only 2013 $$ 2579 $$ 2657 $$ 2658 $$ 3864&&only 2013 $$ 2579&&only 2013 $$ 2579 $$ 2657 $$ 2658 $$ 3315 $$ 3864 $$ 7859&&only 2013 $$ 2579 $$ 3864&&only 2013 $$ 2579 $$ 15975&&only 2013 $$ 2856 $$ 3161 $$ 5386 $$ 5387 $$ 5388 $$ 5389&&only 2014 $$ 2563&&only 2014 $$ 3252&&only 2014 $$ 14876 $$ 14877&&only 2017 $$ 2108&&only 2017 $$ 2108 $$ 2187&&only 2023 $$ 2057 $$ 2089 $$ 2131&&only 2024 $$ 2765 $$ 7733&&only 2024 $$ 3957&&only 2024 $$ 4877&&only 2024 $$ 7733&&only 2024 $$ 8771&&only 2024 $$ 14313&&only 2025 $$ 9020&&only 2028 $$ 8710&&only 2029 $$ 3416&&only 2030 $$ 2030&&only 2031 $$ 2067&&only 2032 $$ 2626&&only 2032 $$ 2626 $$ 8671&&only 2033 $$ 2135&&only 2033 $$ 2135 $$ 7274&&only 2033 $$ 14080&&only 2035 $$ 16810&&only 2040 $$ 2364&&only 2044 $$ 2355&&only 2045 $$ 2225 $$ 2233&&only 2048 $$ 16296&&only 2049 $$ 4349&&only 2050 $$ 4533&&only 2051 $$ 2404&&only 2057 $$ 2131&&only 2059 $$ 3292&&only 2059 $$ 4268 $$ 7921&&only 2059 $$ 4268&&only 2059 $$ 7921&&only 2059 $$ 14443&&only 2059 $$ 15911&&only 2060 $$ 3971&&only 2060 $$ 7597&&only 2060 $$ 10711&&only 2061 $$ 2272&&only 2061 $$ 16048&&only 2062 $$ 7051&&only 2065 $$ 3077 $$ 3328 $$ 3633&&only 2065 $$ 5924&&only 2065 $$ 5961&&only 2066 $$ 2610&&only 2066 $$ 2766&&only 2068 $$ 2242&&only 2068 $$ 8032&&only 2068 $$ 10504&&only 2075 $$ 2700&&only 2075 $$ 2700 $$ 15848&&only 2075 $$ 3128&&only 2075 $$ 8982&&only 2079 $$ 4977&&only 2081 $$ 4316&&only 2081 $$ 4972&&only 2081 $$ 9668&&only 2082 $$ 2319&&only 2086 $$ 2205 $$ 2206 $$ 2456 $$ 2582 $$ 2722 $$ 2868&&only 2086 $$ 2205 $$ 2206 $$ 2456 $$ 2582 $$ 2722 $$ 2797 $$ 2868&&only 2086 $$ 2205 $$ 2456 $$ 2582 $$ 2722 $$ 2868&&only 2086 $$ 2206 $$ 9884&&only 2086 $$ 2206&&only 2086 $$ 2206 $$ 2797&&only 2086 $$ 2206 $$ 6915&&only 2087 $$ 9705&&only 2090 $$ 2783 $$ 3776&&only 2091 $$ 2170&&only 2094 $$ 8825&&only 2096 $$ 7824&&only 2100 $$ 2906&&only 2101 $$ 8610&&only 2107 $$ 4007 $$ 15818&&only 2107 $$ 4007&&only 2108 $$ 2187&&only 2112 $$ 3739&&only 2112 $$ 4970&&only 2117 $$ 3087 $$ 4368&&only 2117 $$ 4368&&only 2117 $$ 7616&&only 2117 $$ 8571&&only 2120 $$ 2121 $$ 2122 $$ 2570 $$ 2571 $$ 4569&&only 2120 $$ 2121 $$ 2122 $$ 5563&&only 2120 $$ 2121 $$ 2122 $$ 2570 $$ 2571 $$ 3788&&only 2120 $$ 2121 $$ 2122 $$ 2570 $$ 2571 $$ 2657 $$ 2658 $$ 3788&&only 2124 $$ 15745&&only 2126 $$ 2127 $$ 5911&&only 2126 $$ 2127 $$ 3253&&only 2126 $$ 2127 $$ 6418&&only 2126 $$ 3356 $$ 5911&&only 2127 $$ 5911&&only 2133 $$ 3651&&only 2136 $$ 12260&&only 2137 $$ 2138 $$ 2139 $$ 2140 $$ 2141 $$ 2180 $$ 2181 $$ 2613&&only 2137 $$ 2138 $$ 2139 $$ 2140 $$ 2180 $$ 2181&&only 2137 $$ 2138 $$ 2139 $$ 2140 $$ 2141 $$ 2180 $$ 2181&&only 2137 $$ 2138 $$ 2139 $$ 2180&&only 2140 $$ 2141 $$ 2181&&only 2142 $$ 7834&&only 2143 $$ 2383 $$ 2448 $$ 2578 $$ 2861&&only 2143 $$ 2383 $$ 2448 $$ 2578&&only 2143 $$ 2383 $$ 2448 $$ 2578 $$ 7602&&only 2143 $$ 2861&&only 2143 $$ 11183&&only 2143 $$ 12630 $$ 13175&&only 2144 $$ 2256&&only 2144 $$ 3585&&only 2146 $$ 3191&&only 2155 $$ 3263 $$ 8596&&only 2155 $$ 3263 $$ 5941&&only 2155 $$ 3263&&only 2155 $$ 5081 $$ 5985&&only 2155 $$ 5081&&only 2155 $$ 9691&&only 2155 $$ 11553&&only 2157 $$ 2665 $$ 5458&&only 2157 $$ 5458&&only 2163 $$ 6762&&only 2163 $$ 8730&&only 2166 $$ 4141&&only 2166 $$ 5239&&only 2168 $$ 2169 $$ 2212&&only 2171 $$ 2465&&only 2172 $$ 2223&&only 2172 $$ 3963&&only 2177 $$ 12415&&only 2183 $$ 3838&&only 2183 $$ 11637&&only 2183 $$ 16838&&only 2184 $$ 7575&&only 2186 $$ 7641&&only 2187 $$ 4835&&only 2189 $$ 3834 $$ 4128&&only 2189 $$ 4128&&only 2189 $$ 10548&&only 2195 $$ 2575&&only 2198 $$ 2306&&only 2198 $$ 2306 $$ 15294&&only 2201 $$ 5868&&only 2202 $$ 6561&&only 2204 $$ 15113&&only 2205 $$ 2456 $$ 2582 $$ 2722 $$ 2868&&only 2205 $$ 8408&&only 2208 $$ 4980&&only 2209 $$ 14349&&only 2213 $$ 4867&&only 2215 $$ 13233&&only 2217 $$ 2786&&only 2217 $$ 12897&&only 2226 $$ 3532&&only 2226 $$ 3911&&only 2231 $$ 5096&&only 2232 $$ 2540&&only 2232 $$ 7439&&only 2234 $$ 3949&&only 2235 $$ 2956&&only 2239 $$ 6754 $$ 6991&&only 2241 $$ 2703&&only 2242 $$ 17141&&only 2244 $$ 2281 $$ 5673&&only 2244 $$ 2281 $$ 8528&&only 2244 $$ 2281 $$ 4990&&only 2244 $$ 2281 $$ 6027&&only 2244 $$ 11796 $$ 12938&&only 2250 $$ 6752&&only 2251 $$ 2251&&only 2254 $$ 2378&&only 2254 $$ 2378 $$ 2634&&only 2254 $$ 2378 $$ 2634 $$ 16790&&only 2254 $$ 6962 $$ 10656&&only 2254 $$ 7610 $$ 13364&&only 2256 $$ 10751&&only 2259 $$ 7167&&only 2260 $$ 15773&&only 2261 $$ 2772&&only 2263 $$ 4037&&only 2263 $$ 10879&&only 2263 $$ 14710&&only 2264 $$ 2763&&only 2265 $$ 6795&&only 2268 $$ 3331&&only 2268 $$ 4357 $$ 15964&&only 2271 $$ 2389&&only 2271 $$ 3770&&only 2275 $$ 4845&&only 2275 $$ 15785&&only 2277 $$ 2647&&only 2277 $$ 4046&&only 2279 $$ 6671&&only 2281 $$ 4359&&only 2281 $$ 4990&&only 2281 $$ 6027&&only 2293 $$ 3478&&only 2293 $$ 5870&&only 2293 $$ 12282&&only 2293 $$ 13832&&only 2299 $$ 2300&&only 2303 $$ 2443 $$ 5054 $$ 5072 $$ 5371 $$ 5790&&only 2303 $$ 2443 $$ 5503&&only 2303 $$ 11507&&only 2303 $$ 14465&&only 2305 $$ 10510&&only 2308 $$ 2350 $$ 4059 $$ 4559 $$ 4797 $$ 5384 $$ 5385&&only 2308 $$ 2350 $$ 4326 $$ 4796 $$ 4798 $$ 4799 $$ 4800&&only 2308 $$ 2350 $$ 4796&&only 2308 $$ 2350&&only 2312 $$ 3026&&only 2312 $$ 3808&&only 2312 $$ 3808 $$ 6771&&only 2312 $$ 15788&&only 2313 $$ 3175&&only 2315 $$ 17345&&only 2318 $$ 2360&&only 2322 $$ 2323 $$ 11721&&only 2322 $$ 2323 $$ 2713&&only 2322 $$ 2323 $$ 9330&&only 2328 $$ 10133&&only 2334 $$ 3378&&only 2334 $$ 10180&&only 2335 $$ 2843&&only 2336 $$ 15122&&only 2338 $$ 2935 $$ 4943&&only 2338 $$ 2935&&only 2338 $$ 2935 $$ 15120&&only 2340 $$ 4724&&only 2340 $$ 14345&&only 2341 $$ 13111&&only 2346 $$ 2492 $$ 2852 $$ 2928&&only 2346 $$ 2492 $$ 2852 $$ 5357&&only 2346 $$ 2492 $$ 2928 $$ 7219&&only 2346 $$ 5357 $$ 12732&&only 2352 $$ 7424&&only 2354 $$ 3254&&only 2354 $$ 3254 $$ 14117&&only 2354 $$ 8558&&only 2354 $$ 13252&&only 2355 $$ 5468&&only 2355 $$ 10720&&only 2361 $$ 3396&&only 2379 $$ 5326&&only 2379 $$ 5988&&only 2379 $$ 9556&&only 2380 $$ 2439 $$ 2478 $$ 2552 $$ 4880&&only 2383 $$ 2448 $$ 2578&&only 2383 $$ 2578&&only 2383 $$ 14093&&only 2384 $$ 2922&&only 2384 $$ 5047&&only 2385 $$ 7489&&only 2388 $$ 2907&&only 2392 $$ 2444&&only 2396 $$ 5098&&only 2402 $$ 3022&&only 2402 $$ 8638&&only 2406 $$ 7052 $$ 9054&&only 2408 $$ 2940 $$ 3205 $$ 4974&&only 2416 $$ 5138&&only 2416 $$ 7615&&only 2420 $$ 4216&&only 2421 $$ 2604 $$ 2743&&only 2422 $$ 2423 $$ 2424 $$ 2425 $$ 2538 $$ 5008 $$ 11956&&only 2422 $$ 2423 $$ 2424 $$ 2425 $$ 2538 $$ 3723 $$ 3785&&only 2422 $$ 2423 $$ 2424 $$ 4284 $$ 5008&&only 2422 $$ 2423 $$ 2424 $$ 2425 $$ 2538 $$ 4284 $$ 5008&&only 2422 $$ 2423 $$ 2424 $$ 4284 $$ 7478&&only 2425 $$ 2538&&only 2426 $$ 11982&&only 2426 $$ 13271&&only 2431 $$ 2746&&only 2432 $$ 10898&&only 2433 $$ 2609&&only 2434 $$ 7702&&only 2439 $$ 2478&&only 2439 $$ 2478 $$ 5194&&only 2439 $$ 2478 $$ 4880&&only 2441 $$ 9266&&only 2443 $$ 5504&&only 2445 $$ 10456&&only 2445 $$ 14181&&only 2446 $$ 2460 $$ 2586&&only 2446 $$ 2586&&only 2451 $$ 4527&&only 2451 $$ 9037&&only 2456 $$ 2722&&only 2456 $$ 2868&&only 2459 $$ 4937&&only 2459 $$ 10697&&only 2461 $$ 3725&&only 2461 $$ 17286&&only 2462 $$ 2838&&only 2464 $$ 2725&&only 2464 $$ 13521&&only 2471 $$ 3006&&only 2473 $$ 2915 $$ 12162&&only 2475 $$ 2903&&only 2483 $$ 13594&&only 2484 $$ 2485&&only 2484 $$ 6767&&only 2485 $$ 8505&&only 2486 $$ 2587 $$ 3959&&only 2486 $$ 3959&&only 2497 $$ 8091&&only 2499 $$ 3661&&only 2499 $$ 3661 $$ 17252&&only 2499 $$ 8603&&only 2504 $$ 4213&&only 2509 $$ 2945&&only 2511 $$ 2632&&only 2514 $$ 11579&&only 2518 $$ 9455&&only 2518 $$ 13844&&only 2526 $$ 3201 $$ 5282&&only 2528 $$ 2971&&only 2530 $$ 6784&&only 2544 $$ 4115&&only 2546 $$ 11036&&only 2554 $$ 14473&&only 2555 $$ 3088&&only 2563 $$ 5343&&only 2563 $$ 7512&&only 2563 $$ 17126&&only 2564 $$ 5712 $$ 6340&&only 2564 $$ 14392&&only 2574 $$ 2655 $$ 4815&&only 2577 $$ 2656 $$ 3241&&only 2584 $$ 3247 $$ 8448&&only 2584 $$ 3247&&only 2584 $$ 6434&&only 2587 $$ 3959&&only 2595 $$ 16624&&only 2598 $$ 2899&&only 2598 $$ 4265&&only 2599 $$ 7548&&only 2611 $$ 3591 $$ 15153&&only 2611 $$ 3591 $$ 4012&&only 2611 $$ 9238&&only 2611 $$ 10125&&only 2612 $$ 7166&&only 2612 $$ 13283&&only 2619 $$ 2620 $$ 2621&&only 2620 $$ 2621&&only 2622 $$ 9758&&only 2633 $$ 2826&&only 2633 $$ 3576&&only 2633 $$ 5721&&only 2634 $$ 12259&&only 2638 $$ 9373&&only 2638 $$ 16226&&only 2647 $$ 4157&&only 2650 $$ 3777&&only 2650 $$ 6249&&only 2650 $$ 8598&&only 2653 $$ 4033&&only 2657 $$ 2658&&only 2663 $$ 3399&&only 2663 $$ 13361&&only 2668 $$ 2871&&only 2679 $$ 2712&&only 2684 $$ 16761&&only 2704 $$ 16289&&only 2706 $$ 2758 $$ 2779 $$ 2888&&only 2706 $$ 2758 $$ 2888&&only 2706 $$ 2758&&only 2707 $$ 13290&&only 2710 $$ 3458&&only 2710 $$ 5074&&only 2714 $$ 16970&&only 2729 $$ 14187&&only 2733 $$ 3550&&only 2733 $$ 5492&&only 2741 $$ 5178 $$ 8043&&only 2749 $$ 3341&&only 2750 $$ 7932&&only 2759 $$ 3148&&only 2761 $$ 4638&&only 2762 $$ 3124&&only 2763 $$ 7758&&only 2771 $$ 2957&&only 2776 $$ 13946&&only 2781 $$ 2989&&only 2784 $$ 2784 $$ 6204&&only 2784 $$ 2784&&only 2788 $$ 5307&&only 2789 $$ 3163&&only 2789 $$ 3163 $$ 7902&&only 2789 $$ 6387&&only 2789 $$ 11172&&only 2798 $$ 3639 $$ 5442&&only 2798 $$ 3639&&only 2818 $$ 2887&&only 2822 $$ 2953&&only 2823 $$ 4596&&only 2823 $$ 8291&&only 2823 $$ 12896&&only 2828 $$ 3090 $$ 5159&&only 2832 $$ 3108 $$ 3996&&only 2832 $$ 6088 $$ 6763&&only 2834 $$ 9412&&only 2838 $$ 4761 $$ 4818&&only 2841 $$ 7855&&only 2848 $$ 7485&&only 2848 $$ 9611 $$ 9612&&only 2848 $$ 15704&&only 2850 $$ 2992&&only 2850 $$ 4756&&only 2856 $$ 3161 $$ 3778&&only 2858 $$ 2937 $$ 8320 $$ 9716&&only 2858 $$ 2937 $$ 3782&&only 2858 $$ 3104 $$ 7430&&only 2858 $$ 3782&&only 2858 $$ 7430&&only 2862 $$ 3840&&only 2866 $$ 3813&&only 2869 $$ 2869&&only 2883 $$ 11533&&only 2897 $$ 4870&&only 2898 $$ 2933 $$ 2934 $$ 3001&&only 2898 $$ 2934 $$ 13644&&only 2899 $$ 4267 $$ 4490&&only 2900 $$ 3294&&only 2901 $$ 14600&&only 2911 $$ 2982&&only 2911 $$ 2982 $$ 5604&&only 2912 $$ 3431 $$ 3580&&only 2912 $$ 3431 $$ 3580 $$ 3581&&only 2912 $$ 5304&&only 2914 $$ 12140&&only 2915 $$ 3393&&only 2923 $$ 4845&&only 2923 $$ 5753&&only 2923 $$ 5762&&only 2923 $$ 12462&&only 2926 $$ 3031&&only 2926 $$ 3203&&only 2926 $$ 6543&&only 2926 $$ 8508&&only 2926 $$ 11789&&only 2927 $$ 4502&&only 2933 $$ 3001&&only 2936 $$ 2946&&only 2938 $$ 3707 $$ 13053&&only 2940 $$ 3205 $$ 4974&&only 2943 $$ 6852&&only 2951 $$ 3198&&only 2954 $$ 5324&&only 2956 $$ 3464&&only 2956 $$ 5475&&only 2970 $$ 3146 $$ 3417 $$ 3482&&only 2974 $$ 2975&&only 2974 $$ 2975 $$ 5201&&only 2976 $$ 3294&&only 2985 $$ 4583 $$ 5018&&only 2985 $$ 4583 $$ 5017 $$ 8116&&only 2985 $$ 5017 $$ 12132 $$ 12133&&only 2985 $$ 5018 $$ 6063&&only 2994 $$ 3236 $$ 3461&&only 2994 $$ 3236 $$ 12591&&only 2995 $$ 3055 $$ 3449 $$ 9581&&only 2995 $$ 3055 $$ 3449 $$ 6193&&only 2995 $$ 3055 $$ 3449 $$ 10173&&only 2995 $$ 3055 $$ 3449 $$ 16256&&only 2995 $$ 3055 $$ 7692&&only 2996 $$ 3598 $$ 3599 $$ 5341 $$ 12408&&only 2996 $$ 3598&&only 2998 $$ 3805&&only 2998 $$ 12849&&only 3007 $$ 3008&&only 3007 $$ 3488&&only 3007 $$ 9832&&only 3007 $$ 17240&&only 3008 $$ 4352&&only 3019 $$ 6077&&only 3032 $$ 3033&&only 3032 $$ 3033 $$ 3490&&only 3042 $$ 3824&&only 3051 $$ 3141&&only 3059 $$ 14815&&only 3071 $$ 7435&&only 3071 $$ 8331&&only 3072 $$ 9018&&only 3073 $$ 3347&&only 3077 $$ 7378&&only 3084 $$ 17537&&only 3090 $$ 4286 $$ 9374&&only 3090 $$ 4372 $$ 5159 $$ 16222&&only 3090 $$ 5159&&only 3103 $$ 4754&&only 3103 $$ 14814&&only 3108 $$ 5809 $$ 6811 $$ 6812&&only 3108 $$ 5809&&only 3110 $$ 5734&&only 3110 $$ 6905&&only 3110 $$ 7381&&only 3110 $$ 8430&&only 3128 $$ 3264 $$ 10348&&only 3131 $$ 5274&&only 3131 $$ 9293&&only 3135 $$ 3976&&only 3151 $$ 4027&&only 3151 $$ 6026&&only 3156 $$ 6674&&only 3157 $$ 4786&&only 3157 $$ 6565&&only 3159 $$ 7638&&only 3169 $$ 3432&&only 3172 $$ 3480 $$ 5886 $$ 7903&&only 3172 $$ 3480 $$ 4766 $$ 5886 $$ 7903&&only 3173 $$ 11103&&only 3174 $$ 4355&&only 3198 $$ 7195 $$ 7196&&only 3203 $$ 16771&&only 3205 $$ 4974&&only 3205 $$ 9807&&only 3205 $$ 15604&&only 3208 $$ 3982 $$ 14505&&only 3208 $$ 6117&&only 3208 $$ 7780 $$ 9075&&only 3212 $$ 3296&&only 3223 $$ 3967&&only 3235 $$ 3822&&only 3245 $$ 4588&&only 3245 $$ 8636&&only 3245 $$ 14700&&only 3248 $$ 7204 $$ 7208 $$ 7987 $$ 9186&&only 3248 $$ 7987&&only 3250 $$ 15290&&only 3256 $$ 5923&&only 3259 $$ 3260&&only 3262 $$ 5306&&only 3264 $$ 3369&&only 3272 $$ 3726&&only 3272 $$ 5020&&only 3272 $$ 5020 $$ 7218&&only 3272 $$ 7868&&only 3276 $$ 3871&&only 3282 $$ 7840&&only 3287 $$ 14279&&only 3293 $$ 12184&&only 3295 $$ 9966 $$ 16729&&only 3300 $$ 7181&&only 3303 $$ 4021 $$ 12372&&only 3303 $$ 4021 $$ 13181&&only 3303 $$ 4021 $$ 6612&&only 3303 $$ 4021&&only 3304 $$ 5068&&only 3305 $$ 5705&&only 3306 $$ 4966&&only 3308 $$ 3309&&only 3312 $$ 14687&&only 3315 $$ 8969 $$ 11182&&only 3320 $$ 3430 $$ 4526 $$ 5030 $$ 5327 $$ 7147&&only 3334 $$ 4111&&only 3334 $$ 8531&&only 3336 $$ 9706&&only 3339 $$ 5099&&only 3340 $$ 3856&&only 3340 $$ 7810&&only 3343 $$ 4816 $$ 4863&&only 3349 $$ 4172&&only 3349 $$ 14342&&only 3351 $$ 11143&&only 3357 $$ 7279&&only 3357 $$ 7571&&only 3357 $$ 10618&&only 3357 $$ 13424&&only 3357 $$ 16702&&only 3361 $$ 5534 $$ 14301&&only 3361 $$ 6571&&only 3362 $$ 5652&&only 3387 $$ 3832&&only 3388 $$ 16920&&only 3403 $$ 4324&&only 3408 $$ 4439&&only 3412 $$ 4198&&only 3419 $$ 4327&&only 3422 $$ 16585&&only 3425 $$ 11876&&only 3426 $$ 11650&&only 3430 $$ 3576&&only 3445 $$ 14134&&only 3453 $$ 14765&&only 3472 $$ 7339 $$ 8503&&only 3474 $$ 9031&&only 3480 $$ 4766 $$ 6370&&only 3483 $$ 6029 $$ 9029&&only 3485 $$ 6899&&only 3487 $$ 3804&&only 3489 $$ 4848&&only 3495 $$ 10450&&only 3496 $$ 3497 $$ 3507&&only 3496 $$ 3497 $$ 3507 $$ 6494&&only 3496 $$ 3507&&only 3496 $$ 3507 $$ 4359&&only 3497 $$ 11126&&only 3503 $$ 13224&&only 3525 $$ 3526 $$ 3672 $$ 4635 $$ 10936 $$ 16819&&only 3525 $$ 4392 $$ 4635 $$ 8948&&only 3526 $$ 4392 $$ 4635&&only 3567 $$ 3670&&only 3571 $$ 3718 $$ 4500&&only 3571 $$ 3718&&only 3577 $$ 6263&&only 3583 $$ 3726&&only 3583 $$ 5310&&only 3583 $$ 7774&&only 3584 $$ 3612 $$ 6808&&only 3594 $$ 7707&&only 3599 $$ 5341&&only 3602 $$ 4494 $$ 5208 $$ 5537&&only 3602 $$ 4494 $$ 5208&&only 3603 $$ 4443&&only 3606 $$ 13892&&only 3612 $$ 6808&&only 3613 $$ 5394&&only 3637 $$ 3656&&only 3638 $$ 4841&&only 3641 $$ 15166&&only 3643 $$ 4421&&only 3646 $$ 7239&&only 3647 $$ 5137 $$ 7563&&only 3647 $$ 5137&&only 3647 $$ 11017&&only 3651 $$ 13226&&only 3657 $$ 4031 $$ 4105&&only 3657 $$ 4031&&only 3657 $$ 4031 $$ 4105 $$ 10990&&only 3657 $$ 4031 $$ 4105 $$ 5942&&only 3671 $$ 3850&&only 3673 $$ 3674 $$ 3675 $$ 8947&&only 3673 $$ 5521 $$ 16820&&only 3673 $$ 8947&&only 3674 $$ 3675&&only 3674 $$ 3675 $$ 3676 $$ 3677 $$ 3853 $$ 3938 $$ 6587&&only 3676 $$ 3677 $$ 3853 $$ 3938 $$ 7924&&only 3676 $$ 3677 $$ 7924&&only 3676 $$ 3677 $$ 3853 $$ 3938&&only 3676 $$ 3677 $$ 6587&&only 3677 $$ 3853 $$ 3938&&only 3681 $$ 6015 $$ 6576&&only 3681 $$ 6576&&only 3686 $$ 4950&&only 3691 $$ 4176&&only 3698 $$ 10875&&only 3721 $$ 6928&&only 3727 $$ 12148&&only 3742 $$ 9547&&only 3743 $$ 8329&&only 3744 $$ 11418&&only 3747 $$ 12330&&only 3752 $$ 4520&&only 3757 $$ 4542&&only 3760 $$ 14689&&only 3763 $$ 4778&&only 3767 $$ 4589&&only 3779 $$ 4353 $$ 4804 $$ 6389&&only 3779 $$ 4353 $$ 4804&&only 3779 $$ 6389&&only 3789 $$ 5409&&only 3790 $$ 5410&&only 3812 $$ 5473&&only 3816 $$ 14789&&only 3818 $$ 4106 $$ 5919 $$ 6944&&only 3818 $$ 5919&&only 3821 $$ 6028 $$ 6597&&only 3821 $$ 8524&&only 3823 $$ 4220&&only 3842 $$ 5263&&only 3842 $$ 8615&&only 3846 $$ 4523&&only 3853 $$ 3938&&only 3859 $$ 10802&&only 3867 $$ 7795&&only 3892 $$ 4180&&only 3927 $$ 4016&&only 3935 $$ 5505&&only 3936 $$ 15007&&only 3941 $$ 4244&&only 3948 $$ 15999&&only 3954 $$ 4250 $$ 5636 $$ 6056 $$ 7999&&only 3954 $$ 4250 $$ 5636 $$ 7202&&only 3954 $$ 4250 $$ 6056 $$ 15632&&only 3968 $$ 4403&&only 3970 $$ 4269&&only 3978 $$ 11186&&only 3978 $$ 15878&&only 4024 $$ 8387&&only 4040 $$ 7509&&only 4041 $$ 5059&&only 4046 $$ 4157&&only 4053 $$ 6803&&only 4055 $$ 12578&&only 4060 $$ 4805&&only 4060 $$ 10106&&only 4073 $$ 4405&&only 4078 $$ 8352&&only 4089 $$ 4089&&only 4091 $$ 5247&&only 4091 $$ 15977&&only 4092 $$ 4093&&only 4095 $$ 11411&&only 4101 $$ 12872&&only 4103 $$ 15900&&only 4106 $$ 5917&&only 4106 $$ 6944&&only 4129 $$ 8497&&only 4148 $$ 4150 $$ 17489&&only 4148 $$ 14677&&only 4155 $$ 5135&&only 4155 $$ 6642 $$ 7730&&only 4155 $$ 7730&&only 4162 $$ 4499&&only 4163 $$ 14662&&only 4200 $$ 5238&&only 4226 $$ 4227 $$ 4228&&only 4227 $$ 4228&&only 4256 $$ 8381&&only 4264 $$ 4410&&only 4273 $$ 4449&&only 4286 $$ 11459&&only 4293 $$ 4383 $$ 5952 $$ 6482&&only 4308 $$ 5047&&only 4310 $$ 7843&&only 4318 $$ 4550&&only 4321 $$ 4598&&only 4330 $$ 8852&&only 4334 $$ 6516&&only 4334 $$ 8585&&only 4350 $$ 6370&&only 4351 $$ 4957&&only 4353 $$ 16087&&only 4356 $$ 5130 $$ 5147 $$ 5907 $$ 10005&&only 4356 $$ 5130&&only 4359 $$ 9985&&only 4363 $$ 10079&&only 4364 $$ 6377&&only 4364 $$ 6628&&only 4364 $$ 15891&&only 4364 $$ 15978&&only 4369 $$ 4763&&only 4369 $$ 11234&&only 4374 $$ 4760 $$ 7984&&only 4395 $$ 6609&&only 4400 $$ 4504 $$ 4505&&only 4400 $$ 4504 $$ 4505 $$ 7906&&only 4402 $$ 8368&&only 4405 $$ 8335&&only 4458 $$ 6171&&only 4473 $$ 5624&&only 4475 $$ 5261&&only 4475 $$ 11189&&only 4485 $$ 16251&&only 4485 $$ 16293&&only 4485 $$ 17569&&only 4489 $$ 5910&&only 4491 $$ 5948&&only 4497 $$ 4599 $$ 4788 $$ 6501 $$ 7685&&only 4497 $$ 4599&&only 4504 $$ 4505&&only 4512 $$ 12105&&only 4514 $$ 6755&&only 4523 $$ 8340&&only 4538 $$ 5011&&only 4574 $$ 4821&&only 4591 $$ 4592 $$ 5444&&only 4591 $$ 4592&&only 4591 $$ 4592 $$ 16150&&only 4593 $$ 7644&&only 4593 $$ 8426&&only 4598 $$ 10820&&only 4600 $$ 7759&&only 4602 $$ 5143&&only 4606 $$ 9515&&only 4609 $$ 5890 $$ 15069&&only 4621 $$ 4873&&only 4629 $$ 8152&&only 4640 $$ 4685&&only 4652 $$ 8831&&only 4652 $$ 10781&&only 4653 $$ 14179&&only 4657 $$ 8875&&only 4658 $$ 5049&&only 4662 $$ 7876&&only 4686 $$ 5229&&only 4686 $$ 10729&&only 4721 $$ 5285&&only 4728 $$ 7332&&only 4728 $$ 10310&&only 4732 $$ 4822 $$ 5848 $$ 5849&&only 4733 $$ 11981&&only 4735 $$ 4927&&only 4755 $$ 12310 $$ 12311&&only 4769 $$ 14697&&only 4776 $$ 8272&&only 4776 $$ 11139&&only 4788 $$ 8658&&only 4793 $$ 4989 $$ 5075 $$ 5197&&only 4793 $$ 5075 $$ 5197&&only 4813 $$ 8512&&only 4814 $$ 6271&&only 4826 $$ 12980&&only 4829 $$ 5426&&only 4830 $$ 4831&&only 4838 $$ 7577 $$ 9119 $$ 15783&&only 4838 $$ 9814 $$ 14425 $$ 14610&&only 4842 $$ 5151&&only 4842 $$ 9163&&only 4878 $$ 5142&&only 4887 $$ 5309&&only 4894 $$ 7241&&only 4904 $$ 14829 $$ 14830&&only 4907 $$ 10782&&only 4954 $$ 6625&&only 4957 $$ 17109&&only 4968 $$ 5641&&only 4994 $$ 5290&&only 4998 $$ 11906&&only 4999 $$ 11909&&only 5000 $$ 5001 $$ 5296&&only 5000 $$ 5296&&only 5003 $$ 5004 $$ 5693&&only 5013 $$ 5308&&only 5018 $$ 6063&&only 5038 $$ 6855&&only 5067 $$ 5157&&only 5088 $$ 6814&&only 5127 $$ 15244&&only 5134 $$ 9204&&only 5144 $$ 14331&&only 5166 $$ 5167&&only 5173 $$ 16637&&only 5175 $$ 6488&&only 5175 $$ 10174&&only 5192 $$ 6505&&only 5192 $$ 15684&&only 5202 $$ 5373 $$ 6806&&only 5207 $$ 6929&&only 5212 $$ 6679 $$ 7288 $$ 8080&&only 5212 $$ 7288&&only 5242 $$ 7133&&only 5245 $$ 8013&&only 5246 $$ 5250
