# Supplementary material for: The Exploration of Novel Regulatory Relationships Drives Haloarchaeal Operon-Like Structural Dynamics over Short Evolutionary Distances
Source: Microorganisms. 2020 Nov 30;8(12):1900. doi: 10.3390/microorganisms8121900 (PMC7760734; doi:10.3390/microorganisms8121900)
Supplement: Supplementary file 1 [file microorganisms-08-01900-s001.zip › S6_AdjacentGenePairsQuerySet.rtf]

1 ; 81 ; 2111 ; 2451 ; 2691 ; 2771 ; 3911 ; 4001 ; 4561 ; 4661 ; 6031 ; 6601 ; 7371 ; 7881 ; 9931 ; 10051 ; 12521 ; 13551 ; 13961 ; 17231 ; 17521 ; 20371 ; 22021 ; 22141 ; 23371 ; 23491 ; 23601 ; 26151 ; 28381 ; 28811 ; 28921 ; 29891 ; 32581 ; 33021 ; 34381 ; 51531 ; 64001 ; 70621 ; 209251 ; 210061 ; 245241 ; 303351 ; 306921 ; 359071 ; 404232 ; 22 ; 112 ; 282 ; 642 ; 912 ; 1832 ; 3282 ; 3392 ; 4652 ; 9672 ; 10122 ; 10812 ; 13122 ; 13332 ; 13822 ; 15172 ; 16562 ; 18152 ; 18292 ; 18422 ; 19352 ; 20202 ; 21032 ; 21192 ; 22732 ; 22742 ; 23092 ; 24672 ; 25032 ; 25722 ; 25862 ; 26412 ; 28242 ; 28952 ; 28962 ; 29052 ; 29382 ; 29422 ; 29702 ; 30302 ; 30362 ; 31462 ; 37102 ; 37592 ; 38472 ; 43312 ; 43982 ; 44522 ; 44872 ; 44882 ; 45182 ; 45902 ; 47182 ; 47652 ; 59252 ; 69492 ; 81382 ; 100512 ; 109952 ; 112082 ; 141042 ; 191272 ; 214092 ; 217342 ; 217362 ; 226242 ; 260842 ; 272302 ; 276722 ; 286402 ; 330352 ; 363182 ; 374482 ; 413103 ; 113 ; 1183 ; 2793 ; 2823 ; 3313 ; 4843 ; 4953 ; 5863 ; 8223 ; 11683 ; 12513 ; 13823 ; 13993 ; 14403 ; 14933 ; 15303 ; 16063 ; 16183 ; 16303 ; 20953 ; 21713 ; 21783 ; 23793 ; 24153 ; 25603 ; 25933 ; 29493 ; 31153 ; 32023 ; 32553 ; 32833 ; 33603 ; 47353 ; 136993 ; 222823 ; 360813 ; 383073 ; 387474 ; 114 ; 174 ; 254 ; 2624 ; 2804 ; 8944 ; 11204 ; 11384 ; 14034 ; 15614 ; 25554 ; 28454 ; 30414 ; 32045 ; 16115 ; 31155 ; 148435 ; 318145 ; 336056 ; 916 ; 1216 ; 1366 ; 3816 ; 3916 ; 7706 ; 10886 ; 16336 ; 16476 ; 16576 ; 16606 ; 16736 ; 16886 ; 18496 ; 20346 ; 22286 ; 22846 ; 24676 ; 25036 ; 30356 ; 39776 ; 42836 ; 189916 ; 198666 ; 216726 ; 220856 ; 304106 ; 321676 ; 357456 ; 374206 ; 378866 ; 407946 ; 413026 ; 416497 ; 77 ; 577 ; 617 ; 2797 ; 3297 ; 4657 ; 9467 ; 10917 ; 11717 ; 11837 ; 12577 ; 12677 ; 13237 ; 15717 ; 21597 ; 22327 ; 22677 ; 25867 ; 29417 ; 30037 ; 31367 ; 33437 ; 36537 ; 37297 ; 50077 ; 51657 ; 57367 ; 58327 ; 58337 ; 65257 ; 74447 ; 75037 ; 76017 ; 82387 ; 84827 ; 141517 ; 144907 ; 254707 ; 258747 ; 266367 ; 266977 ; 290847 ; 363917 ; 414908 ; 4648 ; 4808 ; 6178 ; 7988 ; 12638 ; 16098 ; 48398 ; 315739 ; 5619 ; 131110 ; 1010 ; 24210 ; 65010 ; 73710 ; 86310 ; 134410 ; 160210 ; 171810 ; 188810 ; 228510 ; 358110 ; 363210 ; 415510 ; 516910 ; 765010 ; 2028310 ; 3473910 ; 3600910 ; 3689710 ; 3700410 ; 3905910 ; 4406811 ; 1811 ; 19611 ; 56311 ; 69111 ; 77611 ; 122311 ; 132211 ; 143811 ; 152811 ; 255511 ; 272111 ; 273611 ; 294711 ; 401211 ; 501211 ; 992411 ; 3234712 ; 3112 ; 27812 ; 58912 ; 133812 ; 155212 ; 157412 ; 167812 ; 182612 ; 185312 ; 213312 ; 236212 ; 253512 ; 275612 ; 275712 ; 283712 ; 290412 ; 673312 ; 1129012 ; 1482012 ; 1489312 ; 2171112 ; 2246712 ; 3416912 ; 4187713 ; 1313 ; 3113 ; 4513 ; 18013 ; 33913 ; 41313 ; 155213 ; 157413 ; 167813 ; 182613 ; 221113 ; 268913 ; 415613 ; 491313 ; 521813 ; 750313 ; 1069913 ; 1482013 ; 2406113 ; 2565213 ; 2591113 ; 3267113 ; 3669514 ; 35014 ; 288314 ; 306215 ; 1515 ; 23515 ; 30915 ; 36415 ; 41315 ; 60115 ; 163215 ; 177415 ; 183215 ; 425415 ; 875116 ; 26316 ; 33816 ; 36616 ; 87116 ; 222816 ; 251716 ; 346216 ; 346316 ; 396216 ; 412016 ; 423516 ; 489916 ; 669317 ; 124017 ; 199717 ; 207917 ; 247317 ; 371517 ; 1241618 ; 33018 ; 70218 ; 88818 ; 90418 ; 105718 ; 118518 ; 166218 ; 228618 ; 245018 ; 298518 ; 317118 ; 470618 ; 1738218 ; 2017519 ; 15819 ; 52419 ; 248719 ; 276520 ; 2020 ; 10020 ; 37120 ; 42920 ; 46020 ; 116920 ; 127620 ; 135520 ; 148120 ; 159520 ; 172620 ; 178120 ; 188720 ; 210320 ; 219420 ; 237920 ; 254820 ; 362821 ; 5421 ; 12921 ; 21321 ; 41721 ; 97421 ; 163421 ; 163921 ; 167721 ; 391522 ; 111322 ; 327522 ; 3168123 ; 31223 ; 103323 ; 111523 ; 125523 ; 166923 ; 178723 ; 214623 ; 328123 ; 355123 ; 1539023 ; 3085724 ; 2424 ; 13024 ; 35724 ; 43024 ; 81824 ; 81924 ; 155324 ; 159324 ; 183724 ; 187624 ; 215824 ; 232824 ; 253924 ; 307524 ; 333924 ; 361624 ; 481124 ; 740724 ; 3422824 ; 3596024 ; 3954525 ; 86925 ; 104525 ; 142325 ; 172625 ; 174625 ; 184925 ; 232325 ; 1915626 ; 16726 ; 33326 ; 35826 ; 113826 ; 163626 ; 460226 ; 473526 ; 508426 ; 559726 ; 981026 ; 995426 ; 1052826 ; 1467326 ; 2301026 ; 2400026 ; 2579426 ; 3754427 ; 101827 ; 113927 ; 152527 ; 189727 ; 341827 ; 421227 ; 459627 ; 800527 ; 2375928 ; 8028 ; 8328 ; 9328 ; 11628 ; 13528 ; 53028 ; 138428 ; 177528 ; 182728 ; 190128 ; 203428 ; 230028 ; 230128 ; 264128 ; 399328 ; 537828 ; 942828 ; 2804129 ; 102729 ; 131429 ; 140429 ; 194029 ; 221929 ; 257629 ; 295229 ; 334429 ; 510329 ; 578329 ; 689229 ; 1593229 ; 2309329 ; 3504430 ; 3030 ; 48930 ; 63230 ; 79530 ; 105830 ; 166630 ; 182530 ; 231130 ; 299730 ; 438231 ; 10331 ; 187731 ; 215431 ; 750331 ; 2260232 ; 29132 ; 35032 ; 150832 ; 154032 ; 193932 ; 225332 ; 233032 ; 501432 ; 2618032 ; 2859833 ; 58233 ; 86333 ; 151133 ; 230433 ; 384034 ; 41634 ; 162534 ; 206934 ; 210534 ; 443735 ; 322735 ; 2954335 ; 3049535 ; 3584436 ; 45336 ; 178837 ; 5937 ; 21037 ; 28337 ; 31737 ; 195937 ; 274937 ; 290437 ; 320637 ; 505738 ; 27438 ; 35338 ; 235638 ; 269538 ; 729639 ; 4139 ; 53639 ; 96439 ; 282839 ; 320639 ; 428639 ; 472939 ; 515939 ; 520739 ; 523339 ; 773139 ; 2587439 ; 3131639 ; 3771740 ; 6640 ; 27840 ; 43040 ; 178240 ; 203140 ; 254040 ; 427141 ; 46941 ; 53741 ; 143842 ; 35042 ; 78542 ; 78642 ; 196142 ; 202342 ; 375243 ; 160843 ; 203743 ; 215343 ; 239043 ; 245343 ; 305843 ; 360244 ; 60244 ; 66444 ; 111144 ; 121344 ; 123544 ; 221344 ; 488544 ; 784644 ; 2186945 ; 24745 ; 260245 ; 363145 ; 380045 ; 434545 ; 608545 ; 747445 ; 1331745 ; 3212045 ; 3267145 ; 3351445 ; 3669545 ; 4007146 ; 12146 ; 142046 ; 202746 ; 1389846 ; 2095346 ; 2309847 ; 56447 ; 299647 ; 332747 ; 396947 ; 502748 ; 6148 ; 36448 ; 38948 ; 103548 ; 160048 ; 196548 ; 222748 ; 269748 ; 1574948 ; 2447848 ; 2630948 ; 2738148 ; 2816648 ; 4293849 ; 8649 ; 27949 ; 37549 ; 79049 ; 138049 ; 144249 ; 185049 ; 236949 ; 342249 ; 371949 ; 1704749 ; 3665350 ; 199850 ; 203750 ; 264950 ; 379151 ; 8451 ; 42051 ; 184451 ; 189451 ; 350051 ; 350151 ; 1540052 ; 43452 ; 199652 ; 489552 ; 610653 ; 5353 ; 22953 ; 31653 ; 33253 ; 111153 ; 112253 ; 402554 ; 117354 ; 190654 ; 224354 ; 233154 ; 277654 ; 352954 ; 2164055 ; 6855 ; 56755 ; 110355 ; 155955 ; 526255 ; 4308256 ; 94556 ; 471756 ; 2035756 ; 4017557 ; 19857 ; 41857 ; 49957 ; 222457 ; 274957 ; 334157 ; 1072257 ; 2543158 ; 103158 ; 133058 ; 167358 ; 191258 ; 285358 ; 1183558 ; 2229558 ; 3077759 ; 6959 ; 37559 ; 259859 ; 293659 ; 294659 ; 303759 ; 371459 ; 393259 ; 424259 ; 4039859 ; 4294660 ; 249660 ; 266160 ; 272760 ; 285560 ; 654160 ; 1351860 ; 1389360 ; 1502260 ; 1586060 ; 1782360 ; 2122760 ; 2122860 ; 3295860 ; 4158860 ; 4417560 ; 4417661 ; 27561 ; 27861 ; 30461 ; 153461 ; 159861 ; 229261 ; 255561 ; 283761 ; 339061 ; 343761 ; 385761 ; 473061 ; 503661 ; 876261 ; 1414361 ; 2292862 ; 26262 ; 26562 ; 79462 ; 261862 ; 303862 ; 362962 ; 438762 ; 512062 ; 517662 ; 981062 ; 2134663 ; 27363 ; 235663 ; 287763 ; 356763 ; 418163 ; 466963 ; 2432463 ; 2600163 ; 3794764 ; 7164 ; 28364 ; 128264 ; 165564 ; 171664 ; 276264 ; 279464 ; 312464 ; 317964 ; 362664 ; 362764 ; 433164 ; 835764 ; 986664 ; 1610664 ; 2348364 ; 2426564 ; 3062764 ; 3937064 ; 4354565 ; 6565 ; 24465 ; 160565 ; 276765 ; 344665 ; 352865 ; 651765 ; 810666 ; 52766 ; 81366 ; 229866 ; 345866 ; 400266 ; 1320967 ; 210067 ; 248467 ; 1190168 ; 21668 ; 240768 ; 246668 ; 282768 ; 4141869 ; 161169 ; 228669 ; 303769 ; 371470 ; 252071 ; 168571 ; 213272 ; 21672 ; 36872 ; 79672 ; 177972 ; 246172 ; 474373 ; 314473 ; 376973 ; 675274 ; 81274 ; 101474 ; 174574 ; 275174 ; 320274 ; 451774 ; 492574 ; 3473975 ; 52675 ; 127775 ; 170175 ; 208475 ; 342375 ; 3778576 ; 104976 ; 114276 ; 136576 ; 574676 ; 601776 ; 3158477 ; 112677 ; 189377 ; 219478 ; 99378 ; 109078 ; 175778 ; 276178 ; 2019078 ; 2019179 ; 157679 ; 298880 ; 11680 ; 27780 ; 38980 ; 40480 ; 115980 ; 191380 ; 235280 ; 382280 ; 471080 ; 561280 ; 1021580 ; 1561180 ; 3128180 ; 3629180 ; 3740180 ; 3768180 ; 3996881 ; 41481 ; 61281 ; 116981 ; 2840881 ; 3872482 ; 116682 ; 234783 ; 11683 ; 23383 ; 65983 ; 131583 ; 178783 ; 228283 ; 475983 ; 1998384 ; 218684 ; 236884 ; 2389584 ; 2476785 ; 142785 ; 371986 ; 106386 ; 112086 ; 138086 ; 144286 ; 156086 ; 236886 ; 709286 ; 1161586 ; 1837187 ; 56887 ; 94387 ; 142087 ; 569087 ; 659687 ; 2625388 ; 43588 ; 63188 ; 123988 ; 158688 ; 332889 ; 118889 ; 133089 ; 167989 ; 336989 ; 1561890 ; 106490 ; 478990 ; 657290 ; 3107890 ; 4210091 ; 65491 ; 252491 ; 3216692 ; 47092 ; 50293 ; 73693 ; 80193 ; 108293 ; 4267894 ; 10394 ; 165994 ; 292494 ; 1116494 ; 2922195 ; 36495 ; 120395 ; 136995 ; 221395 ; 238295 ; 284495 ; 2656495 ; 2704495 ; 3405996 ; 33496 ; 37496 ; 53196 ; 109796 ; 209696 ; 2092996 ; 2949797 ; 270297 ; 1229097 ; 3158398 ; 76998 ; 77698 ; 196798 ; 319499 ; 119999 ; 142499 ; 157799 ; 208299 ; 209499 ; 420099 ; 501499 ; 39654100 ; 1191100 ; 1636100 ; 2019100 ; 2944100 ; 40518101 ; 275101 ; 1198101 ; 2479102 ; 474102 ; 662103 ; 413103 ; 583103 ; 1338103 ; 1365103 ; 1552103 ; 2904104 ; 199104 ; 1071104 ; 2469104 ; 2642104 ; 3325104 ; 3578104 ; 40014105 ; 732105 ; 2667105 ; 4861106 ; 942106 ; 1015106 ; 1188106 ; 1585106 ; 3323106 ; 3324106 ; 4017106 ; 4172106 ; 4831106 ; 10090106 ; 15017106 ; 38918107 ; 3198107 ; 4322107 ; 6672107 ; 11092107 ; 28375108 ; 4300108 ; 4567109 ; 360109 ; 977109 ; 2975109 ; 3058109 ; 8171109 ; 10289110 ; 482110 ; 1119110 ; 1689110 ; 1770110 ; 2780110 ; 2863110 ; 3160110 ; 10169111 ; 295111 ; 1267111 ; 1508111 ; 2182111 ; 2373111 ; 2775111 ; 3152111 ; 3425111 ; 3457111 ; 3628111 ; 5417111 ; 6821111 ; 33521111 ; 34086111 ; 38538112 ; 818112 ; 844112 ; 880112 ; 1620112 ; 1681112 ; 3031113 ; 1649113 ; 2291114 ; 555114 ; 42041115 ; 830115 ; 22065116 ; 233116 ; 299116 ; 5270116 ; 9428116 ; 11724116 ; 19984116 ; 29487116 ; 30100116 ; 38702116 ; 41187117 ; 415117 ; 841117 ; 28264118 ; 1627118 ; 2844118 ; 3427118 ; 3535118 ; 29991120 ; 2134120 ; 5549120 ; 21181121 ; 3438122 ; 1016122 ; 1163122 ; 1394122 ; 2102122 ; 2983122 ; 19161123 ; 734123 ; 1673123 ; 18999124 ; 1974125 ; 1430125 ; 1973126 ; 306126 ; 530126 ; 609126 ; 724126 ; 1022126 ; 1176126 ; 1453126 ; 1469126 ; 2192126 ; 2524127 ; 796127 ; 2590127 ; 4958128 ; 1135128 ; 1528128 ; 1870128 ; 4584129 ; 538129 ; 227129 ; 8243129 ; 1730129 ; 1968129 ; 2668129 ; 4405130 ; 258130 ; 1450130 ; 1034130 ; 9483130 ; 4525130 ; 24215130 ; 4811130 ; 34967130 ; 1554130 ; 130130 ; 165130 ; 17685130 ; 11512130 ; 1446130 ; 4023130 ; 1593130 ; 3339130 ; 9729130 ; 7328130 ; 5099131 ; 451131 ; 17116131 ; 2147131 ; 17402132 ; 1030132 ; 1029132 ; 36331132 ; 444133 ; 930133 ; 1455133 ; 1685133 ; 2418134 ; 332134 ; 5323134 ; 1269135 ; 2244135 ; 2326136 ; 292136 ; 720136 ; 769136 ; 838136 ; 1300136 ; 1524136 ; 2512137 ; 357137 ; 1675137 ; 2597137 ; 2869140 ; 430140 ; 505140 ; 1131140 ; 2510140 ; 188140 ; 13180141 ; 476141 ; 452141 ; 25226141 ; 28019141 ; 30890141 ; 1377142 ; 143142 ; 1682143 ; 1604144 ; 788144 ; 1140144 ; 1815144 ; 3612144 ; 5121145 ; 719145 ; 12353145 ; 933145 ; 1658146 ; 471146 ; 1902146 ; 5535146 ; 7055146 ; 1252146 ; 1300146 ; 10815146 ; 9727146 ; 4603147 ; 407147 ; 903147 ; 1223147 ; 1313148 ; 1977148 ; 626148 ; 1000148 ; 1188148 ; 1454148 ; 1324148 ; 43673148 ; 1693149 ; 15609149 ; 1779149 ; 2792150 ; 1387150 ; 1882150 ; 17624150 ; 2705150 ; 162151 ; 33365151 ; 1714152 ; 241153 ; 1252153 ; 30335153 ; 30692153 ; 1531153 ; 2701153 ; 4904154 ; 22491154 ; 1224154 ; 29954154 ; 32486154 ; 2950154 ; 1756154 ; 5003154 ; 5004155 ; 2159155 ; 375155 ; 26636155 ; 2142155 ; 3855156 ; 279157 ; 1673157 ; 1906157 ; 2074157 ; 2374157 ; 38842158 ; 684158 ; 9996158 ; 856158 ; 8357158 ; 1317158 ; 1589158 ; 2755158 ; 5203159 ; 512159 ; 159159 ; 4763159 ; 4650159 ; 5595160 ; 295161 ; 1012161 ; 1104161 ; 3508162 ; 357162 ; 165162 ; 9483162 ; 1322162 ; 15825162 ; 15826162 ; 11872162 ; 2399162 ; 2693162 ; 20149162 ; 3043162 ; 2759162 ; 3354162 ; 3576162 ; 4822163 ; 1041164 ; 1401164 ; 249164 ; 1859164 ; 15439164 ; 10590164 ; 33120164 ; 35650164 ; 3824165 ; 258165 ; 6159165 ; 1882165 ; 1034165 ; 4811165 ; 1387165 ; 17685165 ; 35873165 ; 11512165 ; 42205166 ; 380166 ; 4122166 ; 3655166 ; 562166 ; 15437166 ; 1425166 ; 3224166 ; 2991166 ; 3593167 ; 16316167 ; 513167 ; 1355167 ; 1481168 ; 289170 ; 1868170 ; 1900171 ; 396171 ; 513171 ; 1187171 ; 874171 ; 9505171 ; 3353171 ; 3772171 ; 4047172 ; 4619172 ; 18758172 ; 2164173 ; 4174173 ; 701173 ; 2698173 ; 1278173 ; 15405173 ; 1663173 ; 3383174 ; 10347174 ; 483174 ; 1938174 ; 1939174 ; 36247174 ; 2025174 ; 2152174 ; 2344175 ; 11902175 ; 1170175 ; 30496176 ; 277176 ; 5895176 ; 38922176 ; 894177 ; 774177 ; 775177 ; 1019177 ; 1455177 ; 1934178 ; 339178 ; 3168178 ; 8869178 ; 1863178 ; 5166179 ; 931179 ; 1929180 ; 201180 ; 1037180 ; 607180 ; 1759180 ; 211181 ; 344181 ; 208181 ; 23184181 ; 1603181 ; 7950181 ; 566182 ; 16480182 ; 287182 ; 5097183 ; 1312183 ; 1993183 ; 8438183 ; 1935183 ; 15713183 ; 2970183 ; 35003184 ; 185184 ; 478184 ; 26119184 ; 1079184 ; 18698184 ; 40161184 ; 1901184 ; 2063184 ; 3089184 ; 3374184 ; 3512185 ; 317185 ; 963185 ; 1165185 ; 17448185 ; 534185 ; 3418186 ; 815186 ; 1691186 ; 4772187 ; 30878187 ; 957187 ; 2324187 ; 4610187 ; 3928189 ; 928189 ; 1962189 ; 3870189 ; 24069190 ; 394192 ; 433192 ; 38750193 ; 1762193 ; 4018193 ; 4657193 ; 14670194 ; 880194 ; 881194 ; 1606195 ; 493195 ; 1171195 ; 1587195 ; 3295195 ; 3971196 ; 280196 ; 815196 ; 798196 ; 40135196 ; 40136196 ; 2697196 ; 1930196 ; 38255196 ; 9817196 ; 2897197 ; 5329197 ; 732197 ; 1765198 ; 5462198 ; 1727198 ; 1739198 ; 3162198 ; 3335198 ; 3433199 ; 403199 ; 1802199 ; 3928200 ; 14143200 ; 3427200 ; 787200 ; 23663200 ; 7398201 ; 4730201 ; 2482201 ; 10235201 ; 10383201 ; 41984201 ; 7032202 ; 407202 ; 4039202 ; 3485203 ; 673203 ; 37416203 ; 1989203 ; 2434203 ; 3006204 ; 609204 ; 830204 ; 670204 ; 1426204 ; 2026204 ; 3503204 ; 4552205 ; 962205 ; 1436206 ; 11689206 ; 11690206 ; 6350206 ; 5877206 ; 5423206 ; 5424206 ; 2498206 ; 3195206 ; 4397206 ; 9787206 ; 13107206 ; 13108206 ; 5800206 ; 35042206 ; 35043206 ; 6797206 ; 4610206 ; 15357206 ; 15358206 ; 7173206 ; 27260206 ; 11428206 ; 5605206 ; 6892206 ; 6893206 ; 7498206 ; 6894206 ; 43277206 ; 43278207 ; 2046207 ; 33246207 ; 33247207 ; 2161208 ; 5240208 ; 34640208 ; 42534208 ; 974208 ; 4373209 ; 607209 ; 6221209 ; 924209 ; 1143209 ; 20456209 ; 26338209 ; 1405209 ; 2708210 ; 2185210 ; 1395210 ; 728211 ; 753211 ; 1366211 ; 28127211 ; 35873211 ; 42205212 ; 390212 ; 4107213 ; 864213 ; 1881213 ; 3285214 ; 1211214 ; 720214 ; 1457214 ; 1514214 ; 1525214 ; 1982214 ; 3840215 ; 581216 ; 299218 ; 253218 ; 852218 ; 2330218 ; 1929219 ; 431219 ; 1004219 ; 840219 ; 21504219 ; 2781220 ; 760220 ; 1235220 ; 2453220 ; 3058220 ; 4077222 ; 545222 ; 12242222 ; 922222 ; 934222 ; 1416222 ; 2811223 ; 35449224 ; 4600225 ; 671225 ; 968226 ; 443226 ; 660226 ; 1313227 ; 365229 ; 5681229 ; 331229 ; 4114229 ; 1393229 ; 4025229 ; 6756229 ; 33525229 ; 332229 ; 39344229 ; 12237229 ; 726230 ; 1182230 ; 3317230 ; 4820230 ; 5123231 ; 720231 ; 2108231 ; 2703231 ; 3317232 ; 2602233 ; 1478233 ; 233233 ; 393233 ; 601233 ; 1788233 ; 2076235 ; 309235 ; 364235 ; 22832235 ; 8440235 ; 42009236 ; 357236 ; 1997236 ; 32437236 ; 2826236 ; 1175236 ; 1322236 ; 2366236 ; 42861236 ; 4023237 ; 604237 ; 1029237 ; 3741238 ; 619238 ; 2301239 ; 857239 ; 1549239 ; 30367239 ; 1668239 ; 2265239 ; 8969240 ; 542240 ; 2923240 ; 3601241 ; 1232241 ; 2353242 ; 487242 ; 35903242 ; 1461242 ; 3079242 ; 3814243 ; 959243 ; 383244 ; 352244 ; 741244 ; 865244 ; 1598244 ; 1708245 ; 918245 ; 1512245 ; 1555246 ; 328246 ; 42422246 ; 384246 ; 1340247 ; 588247 ; 607247 ; 991247 ; 2222248 ; 348249 ; 15439249 ; 3824250 ; 481250 ; 271250 ; 4049250 ; 39582250 ; 4765250 ; 9125251 ; 1143251 ; 4725252 ; 355252 ; 422252 ; 605252 ; 1408252 ; 2259252 ; 4611253 ; 648254 ; 5447254 ; 1589254 ; 4528254 ; 3053254 ; 3222254 ; 3321254 ; 24095254 ; 4451254 ; 7360254 ; 2814254 ; 2380254 ; 9660254 ; 10265254 ; 7531254 ; 22302254 ; 4140255 ; 318255 ; 1486255 ; 2396255 ; 3884255 ; 1934255 ; 2371257 ; 834258 ; 1034258 ; 1387258 ; 1450258 ; 9481258 ; 7135258 ; 1876258 ; 2760259 ; 30311259 ; 7345259 ; 6215260 ; 912260 ; 17621260 ; 17662260 ; 13167260 ; 20675260 ; 27656260 ; 32356260 ; 42479260 ; 29075261 ; 2795262 ; 2618262 ; 1114263 ; 405263 ; 2129263 ; 13297264 ; 529264 ; 776264 ; 1216264 ; 24094264 ; 1320264 ; 3126265 ; 1462265 ; 389265 ; 20952265 ; 21731265 ; 22484265 ; 2435266 ; 925266 ; 1565267 ; 36220267 ; 323267 ; 932267 ; 1863267 ; 2053267 ; 2164267 ; 30779268 ; 20403268 ; 1968268 ; 2053268 ; 4398269 ; 282269 ; 863269 ; 1950269 ; 3465270 ; 354270 ; 4970270 ; 2359271 ; 40879271 ; 1161272 ; 15285272 ; 2580273 ; 274273 ; 36624273 ; 41042274 ; 13875274 ; 26001275 ; 26038275 ; 8557275 ; 323275 ; 416275 ; 1383275 ; 1109275 ; 43224276 ; 367276 ; 2106276 ; 3791276 ; 35717277 ; 8363277 ; 24681277 ; 2187277 ; 38922278 ; 12428278 ; 1529278 ; 4061278 ; 22784278 ; 1661278 ; 30684278 ; 2340278 ; 42684279 ; 29846279 ; 6525279 ; 4950279 ; 36653279 ; 17047279 ; 5151280 ; 385280 ; 37745280 ; 1388280 ; 41914281 ; 3366281 ; 836281 ; 10700281 ; 1785281 ; 38037281 ; 38939281 ; 891281 ; 43796282 ; 3140282 ; 1106282 ; 28208282 ; 1302283 ; 923283 ; 13127283 ; 19736283 ; 2710283 ; 7819283 ; 28409283 ; 4011283 ; 6596283 ; 4583283 ; 44122284 ; 1562284 ; 24796284 ; 338284 ; 9149284 ; 1401285 ; 12384285 ; 2296285 ; 13800285 ; 27828285 ; 1147285 ; 36911285 ; 1062285 ; 8891286 ; 1301286 ; 6377286 ; 7092286 ; 40671287 ; 1086287 ; 10766287 ; 2112287 ; 6601288 ; 21227288 ; 1600288 ; 35212288 ; 37708288 ; 41115289 ; 1252289 ; 2977289 ; 38092289 ; 42930290 ; 1432290 ; 621290 ; 1303290 ; 4447290 ; 7315290 ; 36438290 ; 37401290 ; 40624290 ; 41956291 ; 1171291 ; 14791291 ; 1228291 ; 10578292 ; 2216292 ; 44191293 ; 842293 ; 22693293 ; 26661293 ; 35342293 ; 39893294 ; 647294 ; 7343295 ; 13573295 ; 20672295 ; 10905295 ; 32990295 ; 33449295 ; 33508295 ; 34234295 ; 34286295 ; 1038295 ; 776295 ; 41496295 ; 42137296 ; 1582296 ; 1185296 ; 2593296 ; 16806296 ; 416297 ; 12806297 ; 13761297 ; 6855297 ; 27008297 ; 28268297 ; 11200297 ; 11307297 ; 8075297 ; 34847297 ; 41091297 ; 17513298 ; 904298 ; 2689298 ; 3202298 ; 3561298 ; 21181298 ; 6572298 ; 25445298 ; 14843298 ; 1380298 ; 2508298 ; 4897298 ; 16728298 ; 39784299 ; 1041299 ; 26030299 ; 6959299 ; 36080299 ; 919299 ; 39243299 ; 2149299 ; 40560300 ; 1443300 ; 1153300 ; 3617300 ; 38132300 ; 770301 ; 15343301 ; 31816301 ; 2686301 ; 34891301 ; 41179301 ; 42530301 ; 1182302 ; 12188302 ; 363302 ; 515302 ; 12583302 ; 31318302 ; 33729303 ; 5071303 ; 31728303 ; 2008304 ; 24478304 ; 25851304 ; 26309304 ; 27381304 ; 28166304 ; 15749304 ; 31687305 ; 2569305 ; 1705305 ; 1286305 ; 29551305 ; 2216305 ; 3807305 ; 532305 ; 1883305 ; 41847305 ; 43539306 ; 355306 ; 3720306 ; 953306 ; 4900306 ; 4901306 ; 4205306 ; 1155307 ; 19398307 ; 1320308 ; 321308 ; 901308 ; 3641308 ; 41662308 ; 43982309 ; 364309 ; 13309309 ; 8751309 ; 413309 ; 13883309 ; 23206309 ; 24626309 ; 25200309 ; 1315309 ; 34868309 ; 36006309 ; 36655310 ; 524311 ; 719311 ; 21090311 ; 6522312 ; 4820312 ; 5408313 ; 2580313 ; 6137313 ; 618313 ; 1131313 ; 1199314 ; 7103314 ; 1134314 ; 4747314 ; 1265314 ; 3052314 ; 5161314 ; 3530314 ; 4679314 ; 3390314 ; 35037315 ; 786315 ; 30157315 ; 30429315 ; 35807316 ; 500316 ; 26944316 ; 316316 ; 1494317 ; 1644317 ; 29198317 ; 36708317 ; 17041317 ; 41435317 ; 43506318 ; 1143318 ; 16102318 ; 534318 ; 43183319 ; 1665319 ; 6345319 ; 1973319 ; 24411319 ; 1303319 ; 28679319 ; 10228319 ; 886320 ; 463320 ; 5834320 ; 10346320 ; 23212320 ; 23346321 ; 34547321 ; 35950321 ; 43982322 ; 1257322 ; 345322 ; 2683322 ; 6128323 ; 1134323 ; 1418323 ; 4124323 ; 8802323 ; 28794323 ; 35354324 ; 989324 ; 43804325 ; 2177325 ; 1109325 ; 1070325 ; 30747326 ; 23107326 ; 7124327 ; 22352327 ; 1348327 ; 2323327 ; 24100327 ; 25229327 ; 30878327 ; 34375327 ; 42031327 ; 42671327 ; 416328 ; 445328 ; 1283329 ; 1581329 ; 1258329 ; 5165329 ; 5007329 ; 908329 ; 15070329 ; 2586329 ; 28909329 ; 1571329 ; 8181329 ; 3450329 ; 5832329 ; 8239329 ; 971330 ; 1052330 ; 930330 ; 972330 ; 21592330 ; 23056331 ; 5681331 ; 4114331 ; 23366331 ; 35079331 ; 39344332 ; 6756332 ; 17720332 ; 25261332 ; 15016332 ; 27830332 ; 32355332 ; 38051333 ; 12894333 ; 10528333 ; 16115333 ; 8083334 ; 5036334 ; 773335 ; 6341335 ; 12953335 ; 7467335 ; 3798335 ; 14188335 ; 2441335 ; 35348335 ; 17241335 ; 42924336 ; 6247336 ; 759336 ; 21665336 ; 22442336 ; 3422336 ; 34397336 ; 42497337 ; 383337 ; 20933337 ; 14559337 ; 32528337 ; 41242337 ; 42374338 ; 13203339 ; 8193339 ; 4992339 ; 5287339 ; 5706339 ; 3995339 ; 1760339 ; 5304339 ; 4493339 ; 20835339 ; 8862339 ; 25407339 ; 13412339 ; 4331339 ; 5885339 ; 6528339 ; 400340 ; 11868340 ; 31455341 ; 2605341 ; 1082341 ; 443341 ; 7322341 ; 6474341 ; 1665341 ; 3935341 ; 1413341 ; 33050341 ; 1790342 ; 342342 ; 1482342 ; 34073342 ; 36375342 ; 41326343 ; 1023343 ; 617344 ; 2962344 ; 39821344 ; 42535345 ; 840345 ; 3467345 ; 42723346 ; 850346 ; 851346 ; 4150346 ; 4148346 ; 29879346 ; 29880346 ; 30659346 ; 3528346 ; 16742346 ; 37005347 ; 365347 ; 1297347 ; 39106347 ; 40081347 ; 42965347 ; 44030348 ; 1809348 ; 31573348 ; 5630349 ; 12177349 ; 2623349 ; 4676349 ; 25144349 ; 14816349 ; 7983349 ; 6627349 ; 15610349 ; 1294349 ; 3021350 ; 16986350 ; 1033350 ; 1189351 ; 3695351 ; 33873351 ; 40464352 ; 1582353 ; 20041353 ; 20062353 ; 1358353 ; 1254353 ; 8682354 ; 3288354 ; 31984355 ; 1155355 ; 3160355 ; 33257355 ; 39505355 ; 42287356 ; 3773356 ; 1795356 ; 370356 ; 2654357 ; 1322357 ; 1175357 ; 23143357 ; 27725357 ; 1736357 ; 2705357 ; 33064357 ; 3043357 ; 10953358 ; 5215358 ; 10674358 ; 16714358 ; 11661359 ; 12280359 ; 548359 ; 1036359 ; 393359 ; 2559359 ; 6982359 ; 30919360 ; 9760360 ; 4435360 ; 10480360 ; 16355360 ; 34188360 ; 35159360 ; 36990361 ; 1105361 ; 7536361 ; 28263361 ; 648362 ; 12411362 ; 7378362 ; 1567362 ; 15096362 ; 25904362 ; 888363 ; 1407363 ; 1615363 ; 26648363 ; 5375363 ; 1631363 ; 16266363 ; 28996364 ; 22929364 ; 15414364 ; 5255364 ; 465364 ; 1023365 ; 1103365 ; 9410365 ; 8162366 ; 14141366 ; 24025366 ; 10747366 ; 25592366 ; 28907366 ; 30018366 ; 31216367 ; 2034367 ; 1775367 ; 13547367 ; 29487368 ; 4292368 ; 31326370 ; 392370 ; 26075370 ; 5131370 ; 1316370 ; 3586370 ; 43518371 ; 1361371 ; 12279371 ; 24194371 ; 540371 ; 40076371 ; 1524372 ; 2228372 ; 6144372 ; 1603372 ; 1688372 ; 1463372 ; 549372 ; 33242372 ; 2151372 ; 37846372 ; 3585373 ; 1424373 ; 26377373 ; 33903373 ; 39792373 ; 4005374 ; 2103374 ; 9731374 ; 764374 ; 23767374 ; 7841374 ; 26559374 ; 27040374 ; 29497374 ; 1422374 ; 36107374 ; 3847374 ; 43455375 ; 482375 ; 13538375 ; 556375 ; 7920376 ; 1129376 ; 33011377 ; 377377 ; 13285377 ; 6285377 ; 21034377 ; 746377 ; 1366377 ; 24597377 ; 737377 ; 32578377 ; 35067378 ; 11905378 ; 15530379 ; 19855379 ; 21855379 ; 26003379 ; 16766379 ; 38178379 ; 40157379 ; 42313379 ; 42848380 ; 4608380 ; 14554380 ; 8781380 ; 11515381 ; 12309381 ; 40183381 ; 2532382 ; 2000382 ; 5629382 ; 30871383 ; 37898384 ; 2695384 ; 8442384 ; 19525384 ; 2592384 ; 15303384 ; 27696384 ; 15575384 ; 5634384 ; 30061384 ; 30520384 ; 30943384 ; 31981384 ; 32812384 ; 33142384 ; 34113384 ; 7296385 ; 986385 ; 5497385 ; 3413385 ; 399385 ; 2596385 ; 31982386 ; 9879386 ; 1349386 ; 1358386 ; 14763386 ; 26334386 ; 28650386 ; 30468386 ; 2889386 ; 2078386 ; 3022386 ; 41631387 ; 989387 ; 607387 ; 26506387 ; 37443387 ; 26085387 ; 1143387 ; 35842388 ; 1079388 ; 2298389 ; 1323389 ; 32248389 ; 35844389 ; 1610389 ; 11133389 ; 1159390 ; 1588390 ; 790390 ; 24572390 ; 26004390 ; 31791391 ; 391391 ; 9530391 ; 42010391 ; 43220392 ; 1479392 ; 675392 ; 2291392 ; 37194392 ; 1458393 ; 517393 ; 4304393 ; 3201393 ; 25935393 ; 1036394 ; 5156394 ; 13949394 ; 22366394 ; 1481394 ; 2978394 ; 31627394 ; 16835394 ; 40722394 ; 2626395 ; 1254395 ; 31260396 ; 2617396 ; 2401396 ; 3087396 ; 19574396 ; 4047396 ; 30222396 ; 32469396 ; 10045396 ; 3356397 ; 440397 ; 2216397 ; 1386397 ; 33617397 ; 34477397 ; 36335397 ; 1481398 ; 11912398 ; 2948398 ; 5064398 ; 28140399 ; 17962399 ; 34596399 ; 35318400 ; 1337400 ; 5913400 ; 8068401 ; 1456401 ; 1107401 ; 1280401 ; 1881401 ; 25585401 ; 28095401 ; 42873402 ; 1195402 ; 22382402 ; 27704402 ; 1047402 ; 8397402 ; 40496403 ; 5838403 ; 3165403 ; 2519403 ; 38462404 ; 1125404 ; 13218404 ; 10306404 ; 22495404 ; 5252404 ; 6668404 ; 607405 ; 17999405 ; 12799405 ; 20138405 ; 14236405 ; 7085405 ; 813405 ; 599405 ; 4570405 ; 2616406 ; 12288406 ; 5329406 ; 2264406 ; 36381407 ; 2476407 ; 1362409 ; 32843409 ; 38503409 ; 40859409 ; 42580410 ; 2773410 ; 1398411 ; 459411 ; 471411 ; 41934411 ; 462411 ; 44127412 ; 816412 ; 9894412 ; 26160412 ; 27560412 ; 32051412 ; 42507413 ; 951413 ; 3346413 ; 27880413 ; 1632413 ; 7993413 ; 4254413 ; 30880413 ; 31266413 ; 36208413 ; 4089414 ; 13128414 ; 21895414 ; 3858414 ; 5039414 ; 2846415 ; 993415 ; 27823415 ; 934415 ; 34843415 ; 35837415 ; 37448415 ; 38211416 ; 1786416 ; 3269416 ; 5598416 ; 7232416 ; 40713416 ; 1454416 ; 3830417 ; 12124417 ; 6764417 ; 854417 ; 9524417 ; 4148417 ; 2241417 ; 2223417 ; 42465418 ; 12153418 ; 538418 ; 15980418 ; 39348419 ; 878419 ; 1030419 ; 14382419 ; 7437419 ; 16444419 ; 36995420 ; 11108420 ; 30634420 ; 422420 ; 43700420 ; 43701421 ; 30332421 ; 7927422 ; 5155422 ; 34486423 ; 1200423 ; 14691423 ; 34888423 ; 828424 ; 22276424 ; 3234424 ; 24452424 ; 14825424 ; 1958425 ; 8812426 ; 1392426 ; 20240426 ; 3546426 ; 2961426 ; 42522426 ; 1168427 ; 2428427 ; 1475427 ; 8240427 ; 2639427 ; 20859427 ; 27544427 ; 3991427 ; 16174427 ; 34794427 ; 36539427 ; 36715427 ; 2817427 ; 42175427 ; 44055428 ; 3695428 ; 13134428 ; 5110428 ; 34790428 ; 38194428 ; 42026429 ; 1279429 ; 15702429 ; 33718429 ; 38470430 ; 3910430 ; 16370431 ; 1953431 ; 2333431 ; 4365431 ; 33057431 ; 1514432 ; 1225432 ; 884432 ; 1530432 ; 20471432 ; 8703432 ; 23120432 ; 432432 ; 31275432 ; 42112433 ; 713433 ; 1549434 ; 15909434 ; 1421434 ; 16991434 ; 42188435 ; 1615435 ; 30404436 ; 1744436 ; 987437 ; 932437 ; 9483437 ; 21363437 ; 22289437 ; 6313437 ; 754437 ; 1811437 ; 592437 ; 16839437 ; 41498439 ; 1465439 ; 17922439 ; 12698439 ; 18346439 ; 8336439 ; 13176439 ; 20413439 ; 10527439 ; 24467439 ; 12699439 ; 8847439 ; 13049439 ; 7178440 ; 37968440 ; 1725440 ; 1320440 ; 4292440 ; 1066441 ; 754441 ; 22288441 ; 25587442 ; 1163443 ; 2283443 ; 3716443 ; 5597443 ; 20051444 ; 529444 ; 23290444 ; 1614444 ; 1873444 ; 8704444 ; 1700444 ; 16271445 ; 13869445 ; 658445 ; 37341445 ; 44168446 ; 19956446 ; 10572446 ; 1719446 ; 4705446 ; 35077447 ; 19945447 ; 25841447 ; 32414448 ; 1051448 ; 1021448 ; 1330448 ; 1441448 ; 36729449 ; 741449 ; 43094450 ; 3228450 ; 4771450 ; 29535450 ; 43165450 ; 43390450 ; 44172451 ; 1037452 ; 1230452 ; 13986452 ; 7364452 ; 25226452 ; 25422453 ; 2381453 ; 1167453 ; 29655453 ; 30863453 ; 42956454 ; 1240454 ; 3046454 ; 12364454 ; 2636454 ; 23382454 ; 3733454 ; 28763454 ; 35630454 ; 10002455 ; 1062455 ; 19423455 ; 551455 ; 6973455 ; 24009455 ; 2696455 ; 32001455 ; 919456 ; 1746456 ; 6143456 ; 20926456 ; 20927456 ; 4303456 ; 11361456 ; 7277456 ; 11372456 ; 16403456 ; 16996456 ; 17444456 ; 42172456 ; 43758456 ; 43759457 ; 22225457 ; 4642457 ; 8861457 ; 3477457 ; 796458 ; 2063458 ; 1979458 ; 966458 ; 4688458 ; 27878458 ; 4248458 ; 43202459 ; 2532459 ; 30329459 ; 493460 ; 5878460 ; 8699460 ; 3226460 ; 505460 ; 4404461 ; 5027461 ; 13229461 ; 20750461 ; 21545461 ; 21778461 ; 23443461 ; 15094461 ; 32164461 ; 4136461 ; 1602461 ; 43193462 ; 2599462 ; 1447462 ; 28617462 ; 5267462 ; 1605462 ; 35791463 ; 821463 ; 27891464 ; 614464 ; 615465 ; 9097465 ; 2617465 ; 1836466 ; 1263466 ; 1292466 ; 838466 ; 992466 ; 35827466 ; 43450467 ; 497467 ; 21673467 ; 29316468 ; 498468 ; 25605468 ; 39532469 ; 6539469 ; 685469 ; 23608469 ; 32889470 ; 13870470 ; 10071470 ; 27868471 ; 5023471 ; 1902471 ; 2287471 ; 30089471 ; 1300471 ; 9224471 ; 3013472 ; 2258472 ; 1818472 ; 4658472 ; 1858472 ; 30333473 ; 545473 ; 14239474 ; 775475 ; 913475 ; 7567475 ; 29682475 ; 42993475 ; 43585476 ; 914476 ; 13987476 ; 28018477 ; 915477 ; 1997477 ; 1378477 ; 5110477 ; 3242477 ; 29456477 ; 38871477 ; 42344478 ; 40993479 ; 723479 ; 1386479 ; 6966479 ; 32427479 ; 44197480 ; 1238480 ; 1341480 ; 3312480 ; 2836481 ; 510481 ; 18991481 ; 28647482 ; 767482 ; 37688482 ; 7735482 ; 10313482 ; 26375482 ; 1721483 ; 40959484 ; 11851484 ; 1289484 ; 7001484 ; 1006484 ; 40937485 ; 12120485 ; 1181485 ; 18740485 ; 485485 ; 10324485 ; 13749485 ; 28258485 ; 2374485 ; 32493485 ; 40036486 ; 797486 ; 1078486 ; 3900486 ; 4020486 ; 38832487 ; 3902487 ; 14936487 ; 4247487 ; 1311487 ; 42471488 ; 586488 ; 43853489 ; 20886489 ; 25282489 ; 4318490 ; 2282490 ; 19853491 ; 1519491 ; 3576491 ; 1322491 ; 6303491 ; 1034491 ; 5421491 ; 2371492 ; 20090492 ; 42942492 ; 4636493 ; 988493 ; 4879493 ; 19819493 ; 37865494 ; 935494 ; 1499494 ; 5377494 ; 494494 ; 22399494 ; 3748494 ; 3775494 ; 34352494 ; 4452495 ; 6138495 ; 1824496 ; 1397496 ; 4816496 ; 27509496 ; 861496 ; 28750497 ; 498497 ; 25606497 ; 5939497 ; 28229497 ; 28938497 ; 29029497 ; 30035497 ; 31050498 ; 28229498 ; 28938498 ; 39227499 ; 3136499 ; 4158499 ; 3162499 ; 6923499 ; 3780499 ; 9157500 ; 26944500 ; 2209501 ; 5434501 ; 28346501 ; 2147501 ; 15936501 ; 15418501 ; 31700501 ; 5501501 ; 8435501 ; 8625501 ; 39798501 ; 41300501 ; 42343502 ; 10071502 ; 42623503 ; 1092504 ; 729504 ; 27751504 ; 11086504 ; 30369504 ; 17183505 ; 30440505 ; 6083506 ; 1049506 ; 1012506 ; 21312506 ; 21313506 ; 8365506 ; 29658506 ; 35721506 ; 37541506 ; 38429507 ; 1376507 ; 12856507 ; 7584507 ; 31997507 ; 34285508 ; 13128508 ; 2846508 ; 19965508 ; 6749508 ; 3858508 ; 5039508 ; 597509 ; 597509 ; 4867509 ; 8049510 ; 909511 ; 558511 ; 592511 ; 16633512 ; 1252512 ; 8686512 ; 26484512 ; 1385512 ; 34989512 ; 17446513 ; 1070513 ; 1355513 ; 6698513 ; 38599513 ; 42253514 ; 2569514 ; 1911514 ; 2803514 ; 1005514 ; 37943514 ; 39319515 ; 1631515 ; 2815515 ; 37222515 ; 20103515 ; 26647515 ; 35700516 ; 25095516 ; 28835516 ; 37749517 ; 10968517 ; 2232518 ; 22505518 ; 27019518 ; 772518 ; 5765518 ; 4334519 ; 29320520 ; 580520 ; 11666520 ; 3522521 ; 963521 ; 6974522 ; 810522 ; 17687524 ; 6913524 ; 1905524 ; 886524 ; 17236525 ; 599525 ; 1811526 ; 1433526 ; 3981527 ; 11897527 ; 5776527 ; 3608527 ; 828527 ; 4978527 ; 7040527 ; 43410528 ; 19952528 ; 25542528 ; 5951528 ; 40591529 ; 1172529 ; 1216529 ; 25281529 ; 8022529 ; 1630529 ; 42975530 ; 17606530 ; 17673530 ; 4363530 ; 5518530 ; 21254530 ; 13788530 ; 4110530 ; 22323530 ; 5568530 ; 14254530 ; 25306530 ; 9860530 ; 14703530 ; 27759530 ; 28005530 ; 7212530 ; 11295530 ; 33522530 ; 16432530 ; 35145530 ; 11423530 ; 36963531 ; 738531 ; 28016532 ; 927532 ; 8507532 ; 4237533 ; 14985533 ; 17373534 ; 2053534 ; 3609535 ; 1297535 ; 8739535 ; 42391536 ; 1769537 ; 12388537 ; 1791537 ; 25787538 ; 15980539 ; 6973539 ; 1711540 ; 1577540 ; 904540 ; 14607540 ; 4779540 ; 1778540 ; 11216540 ; 5757541 ; 677541 ; 12177541 ; 2623541 ; 13628542 ; 1560542 ; 1438542 ; 40663543 ; 688543 ; 3332544 ; 563544 ; 21142545 ; 1301545 ; 21787545 ; 12770546 ; 17618546 ; 733546 ; 10126546 ; 20372546 ; 27555546 ; 1114547 ; 21737547 ; 14144547 ; 25362547 ; 1553547 ; 32437547 ; 33877548 ; 729548 ; 33063548 ; 36216548 ; 37327548 ; 39465548 ; 42104548 ; 30369548 ; 2022548 ; 25801549 ; 1136549 ; 9951549 ; 4288549 ; 25530549 ; 36509550 ; 742550 ; 28309550 ; 32885550 ; 33758550 ; 37540550 ; 38428550 ; 39691550 ; 43089551 ; 21569551 ; 24009551 ; 12339551 ; 1277551 ; 5889551 ; 31998551 ; 16831552 ; 26046552 ; 2216553 ; 24331553 ; 723554 ; 5871554 ; 556554 ; 40104555 ; 614555 ; 1149555 ; 1404555 ; 1442555 ; 1123555 ; 35828555 ; 7213555 ; 11305555 ; 3415555 ; 2434556 ; 1441556 ; 22507556 ; 4476556 ; 28141556 ; 11127556 ; 29979556 ; 31599556 ; 34823556 ; 36130556 ; 8112556 ; 40573557 ; 9410557 ; 1748557 ; 1150557 ; 1075557 ; 24173557 ; 25625557 ; 29470557 ; 32869557 ; 2170558 ; 1734558 ; 26482558 ; 942559 ; 5456559 ; 2087560 ; 4342560 ; 3624560 ; 8473560 ; 10249560 ; 23046560 ; 24932560 ; 3941560 ; 33393560 ; 38215560 ; 41998560 ; 42157561 ; 12826561 ; 19040561 ; 3471561 ; 29877562 ; 11515562 ; 1299562 ; 2037562 ; 32364562 ; 4828563 ; 2214564 ; 6357564 ; 21829564 ; 13740564 ; 3581564 ; 35220565 ; 795566 ; 795566 ; 964566 ; 2090566 ; 1416566 ; 39095566 ; 4785568 ; 1740568 ; 1934568 ; 5566568 ; 12190568 ; 1426568 ; 1421569 ; 1944569 ; 4094569 ; 1317569 ; 1238569 ; 658570 ; 40125570 ; 2348571 ; 602572 ; 1384572 ; 604572 ; 1346572 ; 8523573 ; 574573 ; 575573 ; 11176574 ; 575574 ; 33935574 ; 15546574 ; 1172574 ; 34870574 ; 1823575 ; 802575 ; 1894575 ; 29548575 ; 16906575 ; 2161576 ; 1239577 ; 11838577 ; 578577 ; 2533577 ; 2042579 ; 28444579 ; 9183579 ; 1383580 ; 13675580 ; 1783581 ; 6214581 ; 2247581 ; 43195582 ; 10840583 ; 1271583 ; 26103583 ; 8007583 ; 30572584 ; 595584 ; 6505584 ; 3106584 ; 35838585 ; 3185585 ; 10369585 ; 5207585 ; 1173585 ; 22932586 ; 801587 ; 25090588 ; 7472588 ; 13228588 ; 2851588 ; 30819589 ; 22603589 ; 2778590 ; 1507591 ; 981591 ; 1167591 ; 13327591 ; 31427591 ; 41902592 ; 1335592 ; 34884592 ; 38268593 ; 817593 ; 824593 ; 21996593 ; 10372593 ; 32472594 ; 20426594 ; 2537594 ; 3242594 ; 3157594 ; 42072594 ; 42939594 ; 43844595 ; 42023595 ; 42962595 ; 29934596 ; 5781596 ; 2999596 ; 605596 ; 28845596 ; 3577597 ; 1195598 ; 1533598 ; 827599 ; 600600 ; 3671600 ; 43865601 ; 4117601 ; 4980601 ; 39742602 ; 6959603 ; 2820603 ; 1526603 ; 626603 ; 1415604 ; 870604 ; 34009605 ; 1238605 ; 4320605 ; 831605 ; 5066605 ; 25662605 ; 1290605 ; 1080606 ; 613607 ; 11984607 ; 12764607 ; 796608 ; 5156608 ; 15642608 ; 1389609 ; 32077609 ; 12900610 ; 30397610 ; 41488611 ; 1174612 ; 613613 ; 16719614 ; 2135615 ; 1414615 ; 26958615 ; 4506616 ; 1414616 ; 4506616 ; 2614617 ; 6217617 ; 13284617 ; 1453618 ; 20937618 ; 766618 ; 2231618 ; 5643618 ; 40047619 ; 620619 ; 6219619 ; 4145619 ; 3688619 ; 24228619 ; 2314619 ; 1892619 ; 31502619 ; 33392619 ; 10202619 ; 9348620 ; 26087620 ; 20571620 ; 8943620 ; 29107620 ; 1919621 ; 1475621 ; 2622621 ; 30040621 ; 37444622 ; 1217622 ; 27789622 ; 30097622 ; 1491622 ; 42544623 ; 1218623 ; 836623 ; 835624 ; 14112624 ; 10780624 ; 31044625 ; 3868625 ; 3315626 ; 1834626 ; 1415626 ; 3717626 ; 627626 ; 26959626 ; 35847627 ; 1834627 ; 13681628 ; 6261628 ; 19311628 ; 7800628 ; 25603628 ; 27786628 ; 7967628 ; 33048629 ; 841629 ; 1228630 ; 12739630 ; 22986630 ; 7227631 ; 13746631 ; 27841631 ; 6118631 ; 40584631 ; 5496631 ; 42427632 ; 28262632 ; 848632 ; 2311632 ; 11205632 ; 15727633 ; 634634 ; 635635 ; 636636 ; 1002636 ; 17913637 ; 638637 ; 849637 ; 28464638 ; 639639 ; 1558639 ; 3582640 ; 2252640 ; 641640 ; 1949640 ; 10089641 ; 1219641 ; 30659642 ; 851642 ; 1178642 ; 13678643 ; 1178643 ; 644643 ; 13678643 ; 20629644 ; 645645 ; 646646 ; 647649 ; 26882649 ; 1011650 ; 6728651 ; 1211651 ; 2420651 ; 24440651 ; 25455651 ; 32825652 ; 1321652 ; 10571652 ; 1110652 ; 720652 ; 9191652 ; 33966653 ; 4020653 ; 4305653 ; 3231653 ; 27545654 ; 12124654 ; 6361654 ; 9524654 ; 3213654 ; 3095655 ; 18999655 ; 857655 ; 25260656 ; 1008656 ; 1009656 ; 15805657 ; 1938657 ; 858657 ; 8740657 ; 5441658 ; 861658 ; 1765658 ; 1653658 ; 22969658 ; 2622658 ; 30363658 ; 2546658 ; 1062659 ; 4344659 ; 2074659 ; 1652659 ; 3182660 ; 1012660 ; 8365660 ; 3356660 ; 34738660 ; 3655660 ; 11492661 ; 1119662 ; 2170662 ; 26385662 ; 33896662 ; 16344663 ; 865663 ; 3454663 ; 7712663 ; 28087663 ; 31626663 ; 13423663 ; 42722664 ; 6959664 ; 1663664 ; 14353665 ; 666665 ; 6324666 ; 5045666 ; 39525667 ; 1116667 ; 668669 ; 4664669 ; 44200670 ; 4365670 ; 12150670 ; 12151670 ; 2403670 ; 2943670 ; 1221670 ; 25537670 ; 10864671 ; 3194672 ; 867672 ; 34264672 ; 35151672 ; 38928672 ; 39168672 ; 41518673 ; 867673 ; 1268673 ; 9784673 ; 5532673 ; 957673 ; 33007674 ; 1222675 ; 1479675 ; 8966676 ; 1479676 ; 1836676 ; 8966676 ; 6395676 ; 7101676 ; 5289676 ; 31259676 ; 32171677 ; 1122677 ; 14270677 ; 2901677 ; 40476678 ; 21765678 ; 26276679 ; 1020679 ; 22640680 ; 1269680 ; 23798681 ; 1560681 ; 3904681 ; 25150681 ; 41369682 ; 808682 ; 1292682 ; 27723682 ; 43958683 ; 1125683 ; 878683 ; 10139683 ; 719684 ; 685684 ; 21105685 ; 6539686 ; 1126686 ; 1284686 ; 7504686 ; 38376687 ; 1097687 ; 1980687 ; 2130687 ; 1185688 ; 1021688 ; 25901688 ; 28870689 ; 1609689 ; 690689 ; 26322690 ; 29029690 ; 1458690 ; 1324690 ; 33704690 ; 40694691 ; 1223691 ; 3795693 ; 1359693 ; 990694 ; 1245694 ; 7317694 ; 24098694 ; 26631694 ; 29489694 ; 2795694 ; 37884694 ; 9503694 ; 42694695 ; 696695 ; 43091696 ; 12199696 ; 1537696 ; 13736696 ; 6005696 ; 10515696 ; 10467696 ; 15048696 ; 42695697 ; 1579697 ; 10516697 ; 5474697 ; 35917697 ; 36560697 ; 39929697 ; 41113698 ; 7355698 ; 2505698 ; 9863698 ; 2238698 ; 7734698 ; 33010698 ; 40254699 ; 7355699 ; 700699 ; 2505699 ; 9863699 ; 2238699 ; 7734699 ; 33010699 ; 40254700 ; 23578700 ; 25255700 ; 32254701 ; 12206701 ; 702701 ; 18083701 ; 19760702 ; 3754703 ; 1226703 ; 17671703 ; 31268703 ; 39169704 ; 1226704 ; 705704 ; 31267705 ; 706706 ; 707707 ; 8210707 ; 1247707 ; 35338708 ; 709709 ; 710710 ; 711712 ; 25253713 ; 714715 ; 12631715 ; 2005715 ; 2844715 ; 37866715 ; 2893716 ; 890716 ; 9923716 ; 28942716 ; 34200716 ; 36337716 ; 11608716 ; 39139716 ; 43548717 ; 1026717 ; 23492717 ; 24410717 ; 10748717 ; 722717 ; 33568717 ; 34201718 ; 13231718 ; 2222719 ; 6152719 ; 37289719 ; 37985720 ; 3704720 ; 5329720 ; 37502721 ; 12216721 ; 13238721 ; 20294721 ; 10273721 ; 13624721 ; 1398721 ; 3092721 ; 4362721 ; 35141721 ; 9338722 ; 1273723 ; 1386723 ; 36026723 ; 1971723 ; 29675723 ; 30409723 ; 5602723 ; 12430724 ; 895724 ; 2223724 ; 2864724 ; 3191724 ; 1605725 ; 896725 ; 13215725 ; 25634726 ; 12925726 ; 727726 ; 9036726 ; 9555727 ; 1642727 ; 29450728 ; 1006728 ; 1708728 ; 31522728 ; 9424729 ; 33063729 ; 36216729 ; 37327729 ; 39465729 ; 42104729 ; 35868730 ; 1304730 ; 20811730 ; 21726730 ; 30885730 ; 32368730 ; 43090731 ; 1789731 ; 2630731 ; 43990732 ; 22422733 ; 12289733 ; 1710733 ; 13741733 ; 22420733 ; 29331733 ; 30598734 ; 1228734 ; 28803734 ; 29376734 ; 32884735 ; 1603735 ; 1043735 ; 12875735 ; 30722736 ; 910736 ; 42678737 ; 21768737 ; 911738 ; 739739 ; 740740 ; 8221740 ; 2343740 ; 3746740 ; 2480741 ; 26316741 ; 27369741 ; 1337742 ; 28309742 ; 32885742 ; 33758742 ; 37540742 ; 38428742 ; 39691742 ; 36815742 ; 40553742 ; 42267743 ; 1231743 ; 5915743 ; 28833743 ; 10228744 ; 1231744 ; 22462744 ; 27023745 ; 921745 ; 39908746 ; 922747 ; 924747 ; 22518747 ; 24908748 ; 1234748 ; 8226748 ; 36636749 ; 930749 ; 1053749 ; 1054749 ; 26971749 ; 31472749 ; 1859749 ; 44145750 ; 1148750 ; 11338751 ; 1236751 ; 4531751 ; 1429751 ; 18725751 ; 29482752 ; 1429752 ; 931752 ; 6069753 ; 7395753 ; 13110753 ; 7737753 ; 3604753 ; 4309753 ; 31263753 ; 32663753 ; 1603753 ; 38641753 ; 40055754 ; 1443754 ; 22288754 ; 12989754 ; 25219754 ; 40204755 ; 28127755 ; 6170755 ; 7738755 ; 26339755 ; 3654756 ; 12352756 ; 13119756 ; 20349756 ; 3922756 ; 34753756 ; 10998757 ; 12356758 ; 2190758 ; 938759 ; 1363759 ; 12898759 ; 3933759 ; 10915759 ; 10259759 ; 26041759 ; 31451759 ; 3107759 ; 38216759 ; 2431759 ; 43750760 ; 26040760 ; 2245760 ; 38693760 ; 39313761 ; 2006761 ; 5042762 ; 1013762 ; 20535762 ; 14291762 ; 38198762 ; 38577762 ; 39419762 ; 40416763 ; 41236764 ; 3332764 ; 24533764 ; 6150765 ; 3362765 ; 19969766 ; 21866766 ; 21867767 ; 1458768 ; 13126768 ; 2074768 ; 3924768 ; 26363768 ; 15869768 ; 31574768 ; 16342768 ; 11475768 ; 42734769 ; 1932769 ; 22472769 ; 24475769 ; 29078769 ; 1763770 ; 2296770 ; 1807770 ; 1406770 ; 2561770 ; 8719770 ; 2245770 ; 4022770 ; 2616771 ; 1749771 ; 15142771 ; 772771 ; 37789773 ; 3753773 ; 1816776 ; 2214776 ; 26327777 ; 33832778 ; 21518778 ; 23731778 ; 24327778 ; 24587778 ; 15171778 ; 26479778 ; 34836778 ; 36940778 ; 36979778 ; 43174779 ; 1154779 ; 15534779 ; 4973780 ; 1154780 ; 952780 ; 15534780 ; 21832780 ; 27554781 ; 952781 ; 12393781 ; 1206781 ; 21833782 ; 1667782 ; 12395782 ; 3502782 ; 21834782 ; 14553782 ; 25897782 ; 16919783 ; 809783 ; 31459783 ; 43170784 ; 5046784 ; 2720784 ; 32867784 ; 37785784 ; 947785 ; 786785 ; 22404785 ; 29652787 ; 18138787 ; 23663787 ; 1207787 ; 37342788 ; 1778788 ; 10389788 ; 1721789 ; 1866789 ; 2629789 ; 1207789 ; 2917789 ; 12668789 ; 22375789 ; 27791789 ; 4580789 ; 2483789 ; 36671789 ; 2730789 ; 6662790 ; 4609790 ; 1982790 ; 31667791 ; 1407791 ; 2879791 ; 1952791 ; 1631791 ; 1208791 ; 30498791 ; 1358792 ; 2467792 ; 1333792 ; 1832792 ; 30580792 ; 2130793 ; 21036794 ; 3649794 ; 2242794 ; 38906794 ; 14317794 ; 41410796 ; 3972796 ; 5768796 ; 1245796 ; 16641796 ; 37416797 ; 10478797 ; 1393797 ; 11528798 ; 966798 ; 1212798 ; 22395798 ; 25832799 ; 11828799 ; 1831799 ; 2325799 ; 6371799 ; 26877799 ; 1951800 ; 1637800 ; 2054801 ; 3089801 ; 39071801 ; 8190802 ; 24605802 ; 24606802 ; 2065802 ; 5864802 ; 3575802 ; 42464802 ; 3705803 ; 970803 ; 1684803 ; 28978804 ; 970804 ; 1085804 ; 1607804 ; 14656804 ; 11526804 ; 41953805 ; 22331805 ; 4874806 ; 11047806 ; 2834807 ; 23713807 ; 24315807 ; 24409807 ; 4516807 ; 28266807 ; 31411807 ; 2185807 ; 9769809 ; 7037809 ; 41895810 ; 22328810 ; 13321810 ; 28283810 ; 29314810 ; 43404811 ; 4447811 ; 8457811 ; 2718811 ; 19148811 ; 19561811 ; 13322811 ; 4431811 ; 14922811 ; 969811 ; 3843811 ; 42738812 ; 968812 ; 42101813 ; 11851813 ; 2536813 ; 10835813 ; 29446813 ; 29529813 ; 34359814 ; 1028814 ; 895814 ; 12219814 ; 3404814 ; 5483814 ; 3193815 ; 1852815 ; 39757815 ; 40135815 ; 41306816 ; 14914816 ; 4469816 ; 33310817 ; 2429818 ; 1450818 ; 1554818 ; 12563818 ; 7407818 ; 6033818 ; 1446818 ; 34707818 ; 9482819 ; 844819 ; 6943819 ; 23789819 ; 27820819 ; 3075819 ; 1657819 ; 2278821 ; 11888822 ; 1251823 ; 32133823 ; 40215824 ; 14284824 ; 43774825 ; 983825 ; 9486825 ; 1707825 ; 13974825 ; 7507825 ; 4278825 ; 32804825 ; 8169826 ; 1095826 ; 983826 ; 25415826 ; 30415826 ; 32803827 ; 1095827 ; 25416827 ; 27862827 ; 30416828 ; 21994828 ; 1718828 ; 830829 ; 19879829 ; 26091830 ; 2723830 ; 22065830 ; 14278830 ; 36192830 ; 16913831 ; 1238831 ; 23723831 ; 41921832 ; 1147832 ; 2150832 ; 29684832 ; 38430833 ; 19939833 ; 36756833 ; 16924834 ; 25761834 ; 1264834 ; 1762835 ; 993835 ; 1833835 ; 5860835 ; 1932835 ; 10356835 ; 3439835 ; 27957835 ; 39307835 ; 42002837 ; 992837 ; 22075838 ; 43450838 ; 12006839 ; 1415839 ; 7412839 ; 4264839 ; 14114839 ; 31501840 ; 1687840 ; 2167840 ; 3476840 ; 4971840 ; 28240840 ; 5704840 ; 1262840 ; 3467841 ; 12015841 ; 1762841 ; 3079841 ; 28264843 ; 1999843 ; 1264843 ; 13744843 ; 1878843 ; 13918843 ; 23658843 ; 23863843 ; 24233843 ; 25309843 ; 14692843 ; 25573843 ; 2295843 ; 26660843 ; 4412843 ; 16329843 ; 6476843 ; 42379843 ; 42583844 ; 1577844 ; 2469844 ; 3578844 ; 3213845 ; 2469845 ; 1604845 ; 6305845 ; 2046845 ; 13275845 ; 3652845 ; 997845 ; 1179845 ; 2019845 ; 1559845 ; 2074845 ; 28719845 ; 4979846 ; 2101846 ; 2831846 ; 6771846 ; 15066846 ; 32946846 ; 37660846 ; 5942846 ; 1269847 ; 19629847 ; 3578847 ; 38561848 ; 28262848 ; 24980848 ; 2231849 ; 1002849 ; 12539850 ; 1219850 ; 37043850 ; 36592852 ; 4277853 ; 1004853 ; 26173854 ; 6362854 ; 17794854 ; 21852854 ; 14989855 ; 1967855 ; 6253855 ; 27661856 ; 18998857 ; 1914857 ; 3326857 ; 1906857 ; 1787857 ; 25033858 ; 1010858 ; 30750858 ; 43648859 ; 1011859 ; 1641859 ; 2116859 ; 5281859 ; 1824859 ; 3267859 ; 9248859 ; 3139859 ; 2429860 ; 909860 ; 1161860 ; 20931860 ; 4935860 ; 40500862 ; 14135862 ; 14494862 ; 25543862 ; 15403862 ; 34752862 ; 3020863 ; 5019863 ; 3014863 ; 1437863 ; 26498863 ; 39654863 ; 10150863 ; 41457863 ; 1110864 ; 1712864 ; 1927864 ; 7928864 ; 33528864 ; 11473864 ; 6689865 ; 1457865 ; 31626865 ; 15004865 ; 27418865 ; 3632865 ; 31045865 ; 39530866 ; 13626866 ; 1119867 ; 35151867 ; 3462868 ; 23070869 ; 2124869 ; 2035869 ; 24089869 ; 2771870 ; 1017871 ; 10505871 ; 25235872 ; 2599873 ; 2106873 ; 4006873 ; 19699873 ; 1937873 ; 33559873 ; 38419874 ; 5531875 ; 40663875 ; 20161875 ; 1691875 ; 36355876 ; 25847876 ; 25934877 ; 954877 ; 1792878 ; 17006879 ; 3048879 ; 13196879 ; 7353879 ; 1022879 ; 3316879 ; 30379879 ; 2768879 ; 8208879 ; 38735880 ; 4035880 ; 13197880 ; 26248880 ; 27009880 ; 38901880 ; 1248881 ; 13197881 ; 4035881 ; 3330881 ; 15129881 ; 29466881 ; 31100881 ; 35911882 ; 12198882 ; 9541882 ; 22641882 ; 23086883 ; 1025883 ; 1225883 ; 5986884 ; 885884 ; 1225884 ; 21544884 ; 43000885 ; 985885 ; 35878886 ; 17157886 ; 6913886 ; 1550886 ; 16346886 ; 16803887 ; 12210888 ; 6344888 ; 18649888 ; 5200888 ; 32929888 ; 2041889 ; 1972889 ; 1155889 ; 7068889 ; 4521889 ; 32352890 ; 1345890 ; 30622890 ; 41380891 ; 1795891 ; 14336891 ; 16359891 ; 8818892 ; 1325892 ; 1810892 ; 1432892 ; 31660892 ; 13718892 ; 33888892 ; 39514893 ; 898893 ; 19993893 ; 21608893 ; 22539894 ; 1561894 ; 30029894 ; 12665894 ; 5037894 ; 41512895 ; 11107895 ; 29999895 ; 30688895 ; 18603896 ; 33675896 ; 38880896 ; 42406896 ; 1347897 ; 1702897 ; 28761898 ; 1190898 ; 23095898 ; 20712898 ; 25905898 ; 29688899 ; 1190899 ; 3102899 ; 23096899 ; 13760899 ; 29688899 ; 2892900 ; 1032900 ; 5345901 ; 1583901 ; 29761902 ; 1258902 ; 1518902 ; 1581902 ; 36679902 ; 8118903 ; 1209903 ; 23529904 ; 2508904 ; 25169904 ; 35706904 ; 38450905 ; 15618905 ; 29691905 ; 1047905 ; 1293905 ; 42003905 ; 42004906 ; 24073906 ; 31669906 ; 35078906 ; 9402907 ; 1039907 ; 23536907 ; 31120907 ; 39980908 ; 21105908 ; 1161908 ; 27020909 ; 1161909 ; 4935909 ; 29697909 ; 4644910 ; 1043911 ; 25233911 ; 9190912 ; 1276912 ; 20675913 ; 923913 ; 1180913 ; 8516914 ; 1229914 ; 31616915 ; 1230915 ; 5352915 ; 26244916 ; 1326916 ; 6404916 ; 7084916 ; 15514916 ; 3738916 ; 5452917 ; 1045917 ; 22582917 ; 17440918 ; 1242918 ; 28868918 ; 41447919 ; 2077919 ; 1405919 ; 1883919 ; 3692919 ; 32593920 ; 12327920 ; 9812920 ; 5418920 ; 22570920 ; 32268921 ; 31681921 ; 10957922 ; 32263923 ; 17654923 ; 1379923 ; 2783923 ; 30407924 ; 4256924 ; 20456925 ; 2767925 ; 30377926 ; 12338926 ; 1047926 ; 28129926 ; 32541927 ; 1379927 ; 2515927 ; 4617928 ; 1648928 ; 40428928 ; 43369930 ; 1054930 ; 1184930 ; 39974930 ; 39273931 ; 29684931 ; 1056931 ; 1482931 ; 1054931 ; 17881931 ; 2719931 ; 6606931 ; 16173932 ; 5052932 ; 4682932 ; 2222932 ; 2671932 ; 42194934 ; 4051934 ; 1791934 ; 3329934 ; 2520934 ; 34353934 ; 38227935 ; 1791935 ; 5377935 ; 3459935 ; 4058936 ; 1001936 ; 1918936 ; 9368936 ; 2482937 ; 5519937 ; 1350937 ; 24066937 ; 4963937 ; 30776938 ; 2190938 ; 1523938 ; 3750938 ; 25208938 ; 1598939 ; 1237939 ; 29946939 ; 40423940 ; 6339940 ; 1774940 ; 3937941 ; 1204942 ; 1369942 ; 1687942 ; 27874942 ; 5969942 ; 35782942 ; 43176943 ; 1716943 ; 1268943 ; 4139944 ; 1564944 ; 5881944 ; 22899944 ; 4375944 ; 8591945 ; 1142945 ; 20358945 ; 21240945 ; 6460945 ; 44300946 ; 947946 ; 1278946 ; 3499946 ; 22927947 ; 13117947 ; 20968947 ; 42503947 ; 1278947 ; 8114948 ; 1267948 ; 28105949 ; 3424949 ; 7456949 ; 22470950 ; 951950 ; 2919951 ; 20326951 ; 22946953 ; 1667953 ; 18420953 ; 37339954 ; 27125954 ; 38618954 ; 39061954 ; 40127955 ; 9576955 ; 1360955 ; 7722956 ; 7045956 ; 13809956 ; 24206956 ; 24630956 ; 1819957 ; 5532957 ; 18712957 ; 4372957 ; 20898957 ; 25657958 ; 2562958 ; 7351958 ; 23740958 ; 25057958 ; 35305958 ; 35830958 ; 37450960 ; 12264960 ; 4541960 ; 1578960 ; 5496960 ; 2817960 ; 11654960 ; 39848960 ; 6095961 ; 4099961 ; 1470961 ; 7587962 ; 3789962 ; 32236962 ; 32871962 ; 33259962 ; 35234962 ; 35769962 ; 37349962 ; 41431964 ; 17813964 ; 29440964 ; 3085965 ; 1079965 ; 3186966 ; 10067966 ; 1769966 ; 1979967 ; 10268967 ; 37628969 ; 1761969 ; 11836969 ; 13315969 ; 2648969 ; 39765970 ; 35806970 ; 40165970 ; 27654971 ; 1340972 ; 1054972 ; 1052972 ; 3217972 ; 26972973 ; 21379973 ; 22410973 ; 16760974 ; 13320974 ; 1718974 ; 33952974 ; 34816974 ; 38834974 ; 42500975 ; 1089975 ; 2246975 ; 2218976 ; 1638976 ; 5527977 ; 11902977 ; 13300977 ; 2876977 ; 7066977 ; 1400977 ; 9545977 ; 26163977 ; 1872977 ; 11626978 ; 21629978 ; 16479978 ; 35790978 ; 44090979 ; 2617979 ; 2581979 ; 29959980 ; 1995980 ; 4274980 ; 4391982 ; 1281982 ; 35265982 ; 9351982 ; 5651984 ; 34907984 ; 16477985 ; 2961986 ; 25509986 ; 39234986 ; 7224986 ; 37037987 ; 16835987 ; 6863987 ; 18917987 ; 3063987 ; 3783987 ; 2780988 ; 5777988 ; 3828988 ; 4291988 ; 43378989 ; 26506989 ; 37443990 ; 1299990 ; 1459990 ; 22597990 ; 35643991 ; 1366992 ; 1263992 ; 35827992 ; 3771992 ; 32047992 ; 33385993 ; 1586993 ; 4615993 ; 2802993 ; 41266994 ; 1737994 ; 1874995 ; 996996 ; 9149997 ; 998997 ; 1604997 ; 13275997 ; 21656997 ; 1005997 ; 13067997 ; 1731998 ; 42587998 ; 1454998 ; 14180998 ; 10077998 ; 28645998 ; 33863998 ; 16459998 ; 2030999 ; 1020999 ; 1267999 ; 1476999 ; 27505999 ; 1639999 ; 2851999 ; 429681000 ; 32111000 ; 177981000 ; 75061000 ; 240461000 ; 87911000 ; 91331000 ; 82091000 ; 300151000 ; 306931000 ; 36371001 ; 209831001 ; 306361001 ; 419831003 ; 350361003 ; 261711003 ; 91401004 ; 218481005 ; 12111005 ; 31601005 ; 11231005 ; 315201007 ; 11091007 ; 300701008 ; 16631008 ; 20721008 ; 382071008 ; 428801009 ; 68661009 ; 11101009 ; 382081010 ; 42211010 ; 296091010 ; 46341011 ; 16411011 ; 16081011 ; 251061011 ; 328651012 ; 294001012 ; 26051012 ; 13971013 ; 48361013 ; 109661014 ; 11151014 ; 10151014 ; 329751014 ; 372981015 ; 11161016 ; 121521016 ; 17301016 ; 19441016 ; 386361016 ; 404371016 ; 439271017 ; 21241017 ; 51541018 ; 21821018 ; 158071019 ; 13181019 ; 237531019 ; 56311020 ; 209331020 ; 132301021 ; 10531021 ; 45461023 ; 82091023 ; 45331023 ; 29771023 ; 14831023 ; 14361024 ; 10251024 ; 421101024 ; 369241024 ; 389241024 ; 392441024 ; 438741026 ; 47251026 ; 11291026 ; 24291026 ; 13971026 ; 342011026 ; 217181026 ; 287231026 ; 306611026 ; 76711027 ; 23771027 ; 32161027 ; 251201027 ; 68601027 ; 386841027 ; 402061027 ; 46181028 ; 122191028 ; 19821028 ; 11891028 ; 291571028 ; 31931028 ; 23591029 ; 335721030 ; 283621031 ; 54131031 ; 20291031 ; 10411031 ; 441481032 ; 217741032 ; 230441032 ; 18701032 ; 245201032 ; 273021033 ; 21141033 ; 287591033 ; 369551033 ; 31301034 ; 18821034 ; 200581034 ; 13871034 ; 43331034 ; 353731034 ; 166091034 ; 435761035 ; 12651035 ; 107341036 ; 17641036 ; 50441036 ; 22311036 ; 33681037 ; 11351037 ; 15251037 ; 347171038 ; 335211038 ; 380521038 ; 389201039 ; 198861039 ; 218221039 ; 149391039 ; 260811039 ; 313961039 ; 377221040 ; 27741040 ; 156091040 ; 82251040 ; 378351041 ; 13411042 ; 19411042 ; 34731042 ; 12781043 ; 300431043 ; 273311044 ; 198101044 ; 206841044 ; 224911044 ; 261181044 ; 372331044 ; 396811046 ; 13071046 ; 134831046 ; 17721046 ; 425361047 ; 15621047 ; 207021047 ; 16041047 ; 428921047 ; 434751049 ; 425301049 ; 60171049 ; 57461049 ; 56331049 ; 315841049 ; 72401049 ; 384291050 ; 225161050 ; 151461050 ; 281391050 ; 308941050 ; 104901051 ; 12951051 ; 11961051 ; 10541052 ; 11961052 ; 215921052 ; 230561052 ; 438371052 ; 350691053 ; 12951053 ; 15911053 ; 22741053 ; 10541053 ; 314731053 ; 47591053 ; 338721053 ; 43941054 ; 14661054 ; 68541054 ; 314811054 ; 387441055 ; 123471055 ; 62061055 ; 221841055 ; 25121055 ; 269681055 ; 276771055 ; 389311056 ; 27191056 ; 15401056 ; 20551056 ; 368181056 ; 14431057 ; 21491057 ; 18851057 ; 50521057 ; 430851058 ; 338181058 ; 376261058 ; 31841058 ; 113171059 ; 123501059 ; 25581059 ; 50401059 ; 21451059 ; 131231059 ; 67501059 ; 37601059 ; 109061059 ; 42041059 ; 26431060 ; 16121060 ; 54881060 ; 21671060 ; 18221060 ; 27201061 ; 216641061 ; 17671061 ; 43771061 ; 374201062 ; 14951062 ; 57481062 ; 73941062 ; 263821062 ; 14451062 ; 294671062 ; 66771063 ; 11501063 ; 241741064 ; 435091065 ; 359041066 ; 17251066 ; 284341066 ; 421211067 ; 11531067 ; 341581068 ; 14271068 ; 204031068 ; 308571070 ; 40471071 ; 162811071 ; 412461072 ; 13771072 ; 295001072 ; 374361073 ; 77561073 ; 11561073 ; 128131073 ; 39311073 ; 66291073 ; 87561073 ; 331871073 ; 349141073 ; 431551074 ; 198661074 ; 78131074 ; 107651074 ; 264501074 ; 413041075 ; 17481075 ; 13031075 ; 39171075 ; 21641075 ; 357731075 ; 201751075 ; 256241075 ; 20601075 ; 428821076 ; 11601076 ; 178121076 ; 279001076 ; 374171077 ; 265111077 ; 328161077 ; 407041078 ; 118231078 ; 137001078 ; 12561078 ; 148821078 ; 108411078 ; 268741079 ; 296311079 ; 22981080 ; 16811080 ; 24931080 ; 132951080 ; 15781080 ; 32731080 ; 374081080 ; 382501081 ; 353321082 ; 178221083 ; 118371083 ; 83701083 ; 186881083 ; 136951083 ; 246091083 ; 13201083 ; 301191083 ; 333491083 ; 343691083 ; 349601083 ; 168951083 ; 115921083 ; 434211084 ; 16821084 ; 11861084 ; 18131084 ; 150471085 ; 268741085 ; 87311085 ; 91631086 ; 209051086 ; 41871086 ; 149251086 ; 149261086 ; 401491087 ; 43411087 ; 75681087 ; 75691087 ; 111081087 ; 58651087 ; 83511087 ; 349991087 ; 154321088 ; 13671089 ; 11651090 ; 42481090 ; 36411090 ; 45021091 ; 186631091 ; 38971091 ; 250871091 ; 70721092 ; 33751092 ; 77641092 ; 37131092 ; 75081092 ; 133261092 ; 41851092 ; 142771092 ; 149131092 ; 264431092 ; 80021092 ; 158811092 ; 162401092 ; 413141092 ; 424631092 ; 434071093 ; 54651093 ; 29621094 ; 17071094 ; 14511094 ; 168591094 ; 51141096 ; 189931096 ; 339561096 ; 14331096 ; 440931097 ; 21301098 ; 290681098 ; 111751098 ; 305161099 ; 26121099 ; 102041099 ; 149521099 ; 72331099 ; 11011099 ; 17161099 ; 374461099 ; 410731100 ; 17641100 ; 132491100 ; 399911101 ; 63071101 ; 132801101 ; 95431101 ; 28401101 ; 19221102 ; 11761102 ; 137451102 ; 22641102 ; 44021103 ; 53141103 ; 434661104 ; 120241104 ; 199501104 ; 203091104 ; 17381104 ; 300671104 ; 414561106 ; 14171106 ; 14561106 ; 31681106 ; 18631106 ; 88691107 ; 13211107 ; 437921107 ; 14851108 ; 11201109 ; 30231109 ; 121221109 ; 58891109 ; 41161110 ; 50161110 ; 44061111 ; 146951111 ; 74681111 ; 46141111 ; 389841111 ; 46841111 ; 94211112 ; 134231112 ; 316251112 ; 399461112 ; 442671113 ; 126961113 ; 49891114 ; 28471114 ; 209461115 ; 442041117 ; 18091117 ; 12211117 ; 306581118 ; 17301119 ; 177831119 ; 18541119 ; 225061119 ; 344811119 ; 380421119 ; 389231119 ; 15951120 ; 29151121 ; 195991121 ; 196261121 ; 294861122 ; 15611122 ; 18701123 ; 58451123 ; 77241123 ; 28471123 ; 21101124 ; 15341124 ; 75491124 ; 316751124 ; 372301124 ; 426301125 ; 281711125 ; 64431125 ; 92931126 ; 28671126 ; 296931126 ; 66311126 ; 30051126 ; 55411126 ; 81771127 ; 12681127 ; 283001127 ; 316221127 ; 175191128 ; 12701129 ; 16521129 ; 22571129 ; 425981130 ; 17691130 ; 122141130 ; 13241130 ; 14191130 ; 11311130 ; 281091130 ; 322611131 ; 122151131 ; 47451131 ; 54781131 ; 95441131 ; 49511131 ; 56201131 ; 44631131 ; 79721132 ; 12731132 ; 13731132 ; 197251132 ; 78041132 ; 287511133 ; 128301133 ; 54401133 ; 11401134 ; 350361135 ; 54691135 ; 24411136 ; 365091136 ; 70331137 ; 13041137 ; 208111137 ; 33131137 ; 287281138 ; 206861138 ; 281171138 ; 40261138 ; 341311138 ; 383671139 ; 82231139 ; 237591140 ; 27161141 ; 341591141 ; 378071141 ; 396641142 ; 203581142 ; 72401142 ; 64601142 ; 11511142 ; 311681143 ; 36451143 ; 19911143 ; 224591143 ; 17961143 ; 342541144 ; 30401144 ; 13081144 ; 204571144 ; 225171144 ; 249091144 ; 392451145 ; 11461145 ; 321691146 ; 18841146 ; 321691147 ; 26351147 ; 137261147 ; 57421148 ; 12361148 ; 222951149 ; 147811149 ; 279631149 ; 23731151 ; 14261151 ; 125131151 ; 203571151 ; 20761151 ; 14781151 ; 401751151 ; 427441152 ; 13561152 ; 315761153 ; 14431153 ; 18651153 ; 341581155 ; 15651155 ; 19721156 ; 379471156 ; 31281156 ; 349141156 ; 25731156 ; 381171156 ; 329091157 ; 138541157 ; 140161158 ; 185081158 ; 99571158 ; 38201158 ; 54671158 ; 133481158 ; 48591158 ; 38901158 ; 109441158 ; 71131158 ; 81081158 ; 424241158 ; 432561159 ; 373231159 ; 24051160 ; 62021160 ; 50361160 ; 170171160 ; 18931161 ; 53421161 ; 251671161 ; 15751162 ; 337911162 ; 399101163 ; 218701163 ; 229951164 ; 33181164 ; 12661164 ; 225041164 ; 15251164 ; 337161165 ; 229771166 ; 118551166 ; 309011167 ; 118631168 ; 118901168 ; 23051168 ; 350411168 ; 352281169 ; 12961169 ; 12591169 ; 67491169 ; 110481169 ; 328731169 ; 390371170 ; 255991170 ; 304961171 ; 45001171 ; 114841172 ; 12161172 ; 13881172 ; 11791173 ; 98441173 ; 186931173 ; 292081174 ; 119911174 ; 256021174 ; 72361175 ; 50301175 ; 17091175 ; 276091175 ; 106571175 ; 53251175 ; 59241175 ; 76801175 ; 68471175 ; 26331175 ; 56531175 ; 99121175 ; 34301176 ; 22411176 ; 53171177 ; 32941177 ; 107321177 ; 108541177 ; 95291177 ; 15981179 ; 19451179 ; 78241179 ; 16631179 ; 307141179 ; 391291179 ; 406511179 ; 31991180 ; 49461180 ; 143021180 ; 146471180 ; 21191181 ; 121201181 ; 17081181 ; 14411182 ; 22161182 ; 338901182 ; 13861182 ; 361701182 ; 171891182 ; 421891182 ; 429361183 ; 12221183 ; 75031184 ; 82551184 ; 16791184 ; 77391184 ; 34011184 ; 89021184 ; 12881184 ; 316721185 ; 21301186 ; 122041186 ; 18131186 ; 163471187 ; 18701187 ; 13451187 ; 35871187 ; 413811187 ; 65231187 ; 289411188 ; 49791188 ; 254051188 ; 87571188 ; 48891191 ; 236151192 ; 54121192 ; 18911192 ; 26731192 ; 24041192 ; 39191192 ; 152331192 ; 42341192 ; 136031192 ; 347341192 ; 170381192 ; 94211192 ; 173171193 ; 40891193 ; 58771193 ; 15411193 ; 51211193 ; 396601194 ; 14801194 ; 13061194 ; 38271195 ; 102051195 ; 304061195 ; 424761196 ; 365751196 ; 269721197 ; 17491197 ; 255221197 ; 266671197 ; 283141198 ; 51551198 ; 36541198 ; 18381198 ; 57501199 ; 47011199 ; 54091199 ; 24821201 ; 410731201 ; 13501201 ; 200481201 ; 205521201 ; 236311201 ; 366761201 ; 428651202 ; 14041203 ; 137961203 ; 263561203 ; 54331203 ; 325381204 ; 42121204 ; 338571204 ; 378281205 ; 70591205 ; 300011205 ; 414391207 ; 18661207 ; 24831208 ; 19521209 ; 207751209 ; 273791209 ; 14611210 ; 53701210 ; 34761212 ; 14221212 ; 367241213 ; 25371214 ; 334921214 ; 338811214 ; 93071214 ; 43281214 ; 392471215 ; 406331216 ; 287611216 ; 65561217 ; 442501218 ; 18331218 ; 13031218 ; 164811221 ; 108641221 ; 306581221 ; 200791221 ; 224661221 ; 19991221 ; 368761221 ; 27481222 ; 191651223 ; 88161223 ; 303871225 ; 231201225 ; 312751225 ; 59861227 ; 26681228 ; 19411228 ; 204791228 ; 156111229 ; 82211229 ; 23431229 ; 37461229 ; 24801229 ; 405311231 ; 270231232 ; 200591232 ; 267141232 ; 359031232 ; 30221233 ; 208281234 ; 204551235 ; 112631236 ; 19271236 ; 384321236 ; 35921237 ; 282271238 ; 22911238 ; 135891238 ; 323541238 ; 26801239 ; 218571239 ; 37421239 ; 85891239 ; 428521240 ; 17471240 ; 15101240 ; 210491240 ; 95201240 ; 229781241 ; 45601241 ; 198541241 ; 78121241 ; 36791242 ; 277351243 ; 12641243 ; 297081243 ; 22871244 ; 105161244 ; 15791244 ; 282691244 ; 292021244 ; 91941244 ; 58621245 ; 224781245 ; 18491246 ; 69091246 ; 69691246 ; 148181247 ; 415171248 ; 337841248 ; 442561250 ; 50951250 ; 128291250 ; 75151250 ; 64231250 ; 76101250 ; 45651250 ; 228411250 ; 70121250 ; 127611250 ; 293041250 ; 111931250 ; 129411251 ; 420321251 ; 28171252 ; 155461252 ; 343541253 ; 118181253 ; 63211254 ; 23911255 ; 19341256 ; 14571257 ; 141511257 ; 144901257 ; 254701258 ; 134291258 ; 15811259 ; 135861259 ; 337721259 ; 390981260 ; 287211261 ; 13901261 ; 39471261 ; 377141261 ; 393251261 ; 47461262 ; 16871262 ; 19191262 ; 34771262 ; 311291262 ; 396371264 ; 137441264 ; 238631264 ; 253091264 ; 102751265 ; 71031265 ; 47471265 ; 30521265 ; 51611265 ; 46791265 ; 350371265 ; 227681266 ; 76061266 ; 24491266 ; 225551266 ; 16921266 ; 389181266 ; 389191267 ; 21991267 ; 296381267 ; 307121267 ; 343461267 ; 412551268 ; 12681268 ; 37251268 ; 19271268 ; 438671269 ; 132291269 ; 17691269 ; 376991269 ; 379881269 ; 25191270 ; 248801271 ; 19401271 ; 31381271 ; 61351271 ; 317011272 ; 19081272 ; 62881272 ; 238101272 ; 262671272 ; 54481273 ; 296141273 ; 316471274 ; 13031274 ; 17761274 ; 273801274 ; 381011275 ; 22621275 ; 139161275 ; 33641275 ; 51251275 ; 86901275 ; 59341275 ; 179421275 ; 272971275 ; 337571276 ; 424781276 ; 59941276 ; 290751277 ; 89241277 ; 62921277 ; 39561277 ; 46361278 ; 16501280 ; 64721280 ; 61901280 ; 132041280 ; 73991280 ; 15491280 ; 36851280 ; 237001280 ; 277551280 ; 23201280 ; 324511280 ; 343841281 ; 56511281 ; 340511281 ; 418961281 ; 418971282 ; 83571282 ; 12821282 ; 157561283 ; 20561283 ; 18291283 ; 42631283 ; 44871283 ; 81381283 ; 18471283 ; 224031283 ; 17801283 ; 49871283 ; 47181283 ; 29701283 ; 74551283 ; 74251283 ; 428971284 ; 26981285 ; 12861285 ; 314011285 ; 386401286 ; 399101286 ; 23461287 ; 14111287 ; 75401287 ; 320101287 ; 392771288 ; 58441288 ; 395061289 ; 176001289 ; 146711289 ; 19911289 ; 61071289 ; 168971289 ; 413021290 ; 14721290 ; 23881290 ; 14731291 ; 71371291 ; 424351292 ; 118931292 ; 14411293 ; 131321293 ; 92141293 ; 347511294 ; 13511294 ; 290721294 ; 307441294 ; 25601297 ; 17041297 ; 160571298 ; 34351298 ; 19221298 ; 14391298 ; 334721298 ; 442861299 ; 426071299 ; 108861299 ; 406501299 ; 244821299 ; 26681300 ; 25981300 ; 255341300 ; 151271300 ; 45741300 ; 337701300 ; 82321301 ; 147981301 ; 93391302 ; 31401303 ; 19731303 ; 122781303 ; 358651303 ; 23671304 ; 237621305 ; 55771306 ; 13071306 ; 123251307 ; 72221308 ; 204571308 ; 392451308 ; 97511309 ; 77381309 ; 263391309 ; 123521309 ; 109981309 ; 33611309 ; 43091311 ; 14701311 ; 20251311 ; 73141311 ; 40991311 ; 218111311 ; 59241311 ; 229921311 ; 104581311 ; 79531311 ; 275141311 ; 21521311 ; 86681311 ; 427201313 ; 13141313 ; 307401315 ; 16181315 ; 22651315 ; 333061315 ; 20081315 ; 24541316 ; 51311316 ; 17101316 ; 64681316 ; 389421317 ; 13681317 ; 13851318 ; 41171318 ; 288481318 ; 21741318 ; 207491318 ; 24751318 ; 15281318 ; 56311318 ; 29031319 ; 240371320 ; 240941322 ; 65241322 ; 95591322 ; 22951322 ; 185201322 ; 47311322 ; 85191322 ; 43011322 ; 126861322 ; 23991322 ; 33391323 ; 50451323 ; 33251323 ; 277171323 ; 341991323 ; 434321324 ; 17871324 ; 274881324 ; 76071325 ; 14321325 ; 20171326 ; 65621326 ; 282131326 ; 159451326 ; 312621327 ; 28301327 ; 27081327 ; 42591327 ; 336891327 ; 441491328 ; 252211328 ; 303921328 ; 376761329 ; 258581329 ; 315801329 ; 31171329 ; 399021330 ; 177931330 ; 16051330 ; 255741330 ; 16151330 ; 59291330 ; 22231330 ; 104421330 ; 86931330 ; 169711331 ; 62461331 ; 70911331 ; 268811331 ; 26491331 ; 76501331 ; 361901331 ; 382841332 ; 13641332 ; 15021332 ; 192001332 ; 75931332 ; 51231332 ; 333691333 ; 39881333 ; 64061333 ; 49881333 ; 106041333 ; 37071334 ; 157021334 ; 294931334 ; 315751335 ; 200791335 ; 426161336 ; 16581336 ; 45561337 ; 19941337 ; 193281337 ; 22881339 ; 199411339 ; 149671339 ; 436561340 ; 216161340 ; 54591341 ; 204831341 ; 287111341 ; 14211341 ; 16631342 ; 29051342 ; 205241342 ; 323471343 ; 14761343 ; 16071343 ; 54351343 ; 16701343 ; 261991343 ; 433841344 ; 87181344 ; 111581344 ; 377911344 ; 436521345 ; 387641345 ; 224751345 ; 422361346 ; 16101346 ; 180821346 ; 129791346 ; 143401346 ; 79441346 ; 15381347 ; 199251347 ; 103261347 ; 288761347 ; 79921348 ; 202521348 ; 29911348 ; 30411348 ; 170231349 ; 47581349 ; 202891349 ; 97431349 ; 81801349 ; 352031349 ; 357341349 ; 415261349 ; 28031349 ; 14631350 ; 136361350 ; 15231350 ; 366761350 ; 282981351 ; 25601351 ; 36051351 ; 386661351 ; 136381352 ; 123831353 ; 134151353 ; 268341353 ; 301321353 ; 335981354 ; 13961354 ; 34991354 ; 104551354 ; 52131354 ; 244501354 ; 318731354 ; 385731355 ; 94571355 ; 14811355 ; 342931356 ; 131161356 ; 401761357 ; 67011358 ; 20001358 ; 335531358 ; 357601358 ; 402191359 ; 111841360 ; 199771360 ; 17721360 ; 63521360 ; 263251360 ; 19331360 ; 159681360 ; 442981361 ; 224961361 ; 252461361 ; 380981362 ; 356961362 ; 372191362 ; 173061363 ; 382161363 ; 31071363 ; 386921364 ; 51231364 ; 38511364 ; 34941365 ; 200361365 ; 308211365 ; 343091365 ; 50441366 ; 400551366 ; 44581367 ; 22071367 ; 264331367 ; 367351367 ; 412931368 ; 135541368 ; 205861368 ; 296111369 ; 62201369 ; 200471369 ; 278471369 ; 321801369 ; 71681369 ; 46071370 ; 35841370 ; 20731370 ; 15001370 ; 47441370 ; 63971370 ; 40081370 ; 166741370 ; 394311370 ; 47951371 ; 29921371 ; 115751372 ; 23661372 ; 18261372 ; 61621373 ; 243021373 ; 274311373 ; 307661373 ; 434741374 ; 95571374 ; 201501374 ; 122691374 ; 123711374 ; 363491374 ; 22921374 ; 73361374 ; 30431375 ; 15111377 ; 137681377 ; 45141378 ; 21371378 ; 14981378 ; 25061378 ; 26371379 ; 430021380 ; 119951380 ; 33091380 ; 47421380 ; 16591381 ; 40261381 ; 14811381 ; 27301382 ; 342931382 ; 224291382 ; 18881382 ; 343741382 ; 437081383 ; 18601383 ; 16571383 ; 240751385 ; 358051386 ; 22161386 ; 243991386 ; 310721386 ; 52531387 ; 21321387 ; 57151387 ; 26081387 ; 14461387 ; 198331387 ; 356811388 ; 198741388 ; 283421389 ; 33321389 ; 43141389 ; 40421389 ; 109201389 ; 337921390 ; 24271390 ; 14371390 ; 80031391 ; 348711392 ; 77681392 ; 73501392 ; 324431392 ; 330441392 ; 410131393 ; 150161393 ; 14551393 ; 17121394 ; 21021394 ; 143391395 ; 121191395 ; 47391395 ; 252771396 ; 48321398 ; 13981398 ; 57311398 ; 40371398 ; 357971398 ; 29311399 ; 20961400 ; 36151400 ; 32151400 ; 115991401 ; 122281402 ; 82241402 ; 57471402 ; 131461402 ; 41321402 ; 41951402 ; 248941402 ; 90331402 ; 79401402 ; 39551402 ; 90341403 ; 202951403 ; 30411403 ; 318941403 ; 370201403 ; 381331403 ; 20741403 ; 433231404 ; 43281404 ; 200321404 ; 173601405 ; 21621405 ; 15971406 ; 18071406 ; 56231408 ; 68901408 ; 217061409 ; 127541409 ; 21231409 ; 144481409 ; 153021410 ; 17841411 ; 18211413 ; 419421416 ; 382381417 ; 14561418 ; 216041419 ; 186231419 ; 18851419 ; 315521419 ; 24031419 ; 366641420 ; 256211420 ; 435471421 ; 406501421 ; 25651421 ; 442331422 ; 50691422 ; 50781422 ; 15701423 ; 198211424 ; 42571424 ; 33501424 ; 312521425 ; 29911425 ; 32241425 ; 30591425 ; 31031425 ; 47541425 ; 442591426 ; 343601426 ; 20261427 ; 52351427 ; 20981427 ; 299221427 ; 338431427 ; 22591428 ; 242981428 ; 48121428 ; 116511429 ; 27391429 ; 281261429 ; 299521430 ; 15491430 ; 206601430 ; 137891430 ; 109291430 ; 276591430 ; 16341430 ; 309371430 ; 337241430 ; 16581431 ; 144041431 ; 144071431 ; 51881431 ; 243001431 ; 46911431 ; 109351431 ; 71251431 ; 149231431 ; 90221431 ; 109021431 ; 149451431 ; 109381431 ; 92451431 ; 78621431 ; 105351431 ; 92611431 ; 115321431 ; 93251431 ; 136121431 ; 365871431 ; 365881431 ; 135451431 ; 367311431 ; 102121432 ; 17051432 ; 38161432 ; 29901433 ; 339561433 ; 440931433 ; 73021433 ; 116851434 ; 21631434 ; 136941434 ; 142221434 ; 278241434 ; 286041434 ; 155081434 ; 405541434 ; 414421436 ; 29771436 ; 61831436 ; 16811436 ; 171691437 ; 40331437 ; 26601437 ; 136831437 ; 414581438 ; 28281438 ; 322321439 ; 19221439 ; 20921439 ; 102851439 ; 54141439 ; 158581439 ; 300731439 ; 323711440 ; 17991440 ; 207181440 ; 229441441 ; 19141441 ; 50781441 ; 79951441 ; 58451441 ; 343451441 ; 21701441 ; 33261442 ; 214291442 ; 163451443 ; 401781443 ; 383221444 ; 341571444 ; 352061446 ; 65241446 ; 36161446 ; 158261446 ; 334321446 ; 415011447 ; 14711447 ; 14851447 ; 16051448 ; 53521448 ; 20961448 ; 73981448 ; 217981448 ; 165021450 ; 347071450 ; 412191454 ; 425871454 ; 120191454 ; 47461454 ; 47591454 ; 169681454 ; 405811454 ; 436731455 ; 115021455 ; 121091455 ; 76641455 ; 102821455 ; 214641455 ; 230691455 ; 49241455 ; 145921455 ; 276441455 ; 56161455 ; 111371455 ; 156231455 ; 16121455 ; 66561455 ; 76651455 ; 369681455 ; 112761455 ; 426971456 ; 70971456 ; 71291456 ; 252761458 ; 371941459 ; 439411460 ; 14681460 ; 14691460 ; 423871460 ; 436541461 ; 420801462 ; 31711462 ; 60841463 ; 301461463 ; 17951463 ; 131551464 ; 352601465 ; 316781466 ; 14841467 ; 14881467 ; 14711467 ; 68541467 ; 218001468 ; 235871469 ; 34791469 ; 37611470 ; 75871470 ; 188891470 ; 45481470 ; 214561470 ; 142591470 ; 286611470 ; 86311470 ; 90201470 ; 440351472 ; 311281473 ; 22351473 ; 367521473 ; 369361474 ; 22191474 ; 35731474 ; 141731474 ; 26081476 ; 304761476 ; 36581476 ; 82031476 ; 43761477 ; 32491477 ; 261961478 ; 17881478 ; 295531478 ; 302611478 ; 436051479 ; 122441479 ; 45181479 ; 230491479 ; 335611479 ; 141661479 ; 378831479 ; 106351480 ; 195981480 ; 396631480 ; 428891480 ; 198031481 ; 421831481 ; 421651482 ; 141471483 ; 330611483 ; 372141483 ; 424041484 ; 15271484 ; 228291484 ; 236791484 ; 434821485 ; 348701486 ; 273221486 ; 391351486 ; 43521487 ; 88461487 ; 249761487 ; 255821487 ; 343961487 ; 28991487 ; 418281488 ; 15061488 ; 404621488 ; 17551489 ; 120091489 ; 125701489 ; 200341489 ; 204381489 ; 138151489 ; 229361489 ; 246391489 ; 249221489 ; 325571489 ; 112751489 ; 162341489 ; 350571489 ; 114601489 ; 93751489 ; 394411489 ; 404081489 ; 410761489 ; 420591489 ; 426921489 ; 429541489 ; 433721489 ; 438221490 ; 209631490 ; 87521490 ; 254561491 ; 264211492 ; 18081492 ; 14921492 ; 48241492 ; 346881493 ; 372981493 ; 177611494 ; 202091494 ; 39081494 ; 264191494 ; 114741494 ; 168141494 ; 400531494 ; 126211495 ; 18941495 ; 353891495 ; 23101495 ; 426761496 ; 47581496 ; 17771496 ; 78421496 ; 237611496 ; 263241497 ; 15841497 ; 213271497 ; 290981497 ; 363511497 ; 15361498 ; 20471498 ; 387081499 ; 16631500 ; 15441500 ; 51601500 ; 205691500 ; 402211500 ; 425681501 ; 15441501 ; 126541501 ; 16711503 ; 15941503 ; 15041504 ; 15051504 ; 404631505 ; 17551505 ; 68541505 ; 63201506 ; 17551506 ; 404621506 ; 217991506 ; 68541506 ; 63201507 ; 118961507 ; 20991507 ; 231091507 ; 333651507 ; 352721507 ; 93371507 ; 434801509 ; 412421510 ; 250761510 ; 413171511 ; 122971511 ; 72531512 ; 284341512 ; 49421512 ; 102351512 ; 289621512 ; 415921513 ; 25461513 ; 387001513 ; 392751514 ; 177871514 ; 34401514 ; 261691514 ; 18081514 ; 373251515 ; 30931516 ; 122171516 ; 266441516 ; 342231516 ; 424311516 ; 438821518 ; 28851519 ; 63311519 ; 337931519 ; 342781520 ; 200341520 ; 86691520 ; 112751520 ; 165361520 ; 394411520 ; 410761520 ; 426921520 ; 438211521 ; 15411521 ; 15571522 ; 236591522 ; 144551522 ; 403881522 ; 412701524 ; 308671524 ; 328091524 ; 391271525 ; 21861525 ; 41931525 ; 320591526 ; 34721526 ; 29461526 ; 265221527 ; 228291528 ; 86341529 ; 96011529 ; 19831529 ; 17261529 ; 107911529 ; 36051529 ; 42961529 ; 406911530 ; 40101530 ; 109231530 ; 81491530 ; 215421530 ; 64271531 ; 236611531 ; 253171531 ; 414911532 ; 31221532 ; 35681532 ; 26061533 ; 424681534 ; 330221534 ; 396201534 ; 409861535 ; 307121535 ; 32581536 ; 18141536 ; 15841536 ; 41471537 ; 58621537 ; 331011538 ; 45751538 ; 59801539 ; 73081539 ; 326861540 ; 74711540 ; 143351540 ; 159721540 ; 316771541 ; 400101542 ; 15571542 ; 20041542 ; 75991542 ; 38541543 ; 26121545 ; 16241547 ; 18431547 ; 270341547 ; 22341548 ; 18011548 ; 437071549 ; 33501549 ; 90051549 ; 254681549 ; 42991549 ; 18401550 ; 72821551 ; 126571551 ; 349701551 ; 368301552 ; 67181553 ; 437011553 ; 32211553 ; 368361553 ; 173391553 ; 174301553 ; 441351554 ; 144021554 ; 145121554 ; 247661555 ; 16901555 ; 62971555 ; 20931555 ; 359071555 ; 17521555 ; 380581556 ; 443001556 ; 85231556 ; 34441558 ; 22521558 ; 19491559 ; 121171559 ; 385411559 ; 414211559 ; 50571560 ; 18621561 ; 16271562 ; 269981562 ; 290551562 ; 296151562 ; 328381563 ; 21931565 ; 206791565 ; 282701566 ; 15671566 ; 15861566 ; 17361566 ; 204681566 ; 59611567 ; 15861567 ; 33281567 ; 273141568 ; 33501568 ; 78591568 ; 299951568 ; 384551569 ; 17051569 ; 23671569 ; 326751569 ; 28811569 ; 422721570 ; 22651570 ; 102391570 ; 77181570 ; 410581571 ; 25271571 ; 312271571 ; 24171572 ; 104711572 ; 399671573 ; 26471573 ; 190391573 ; 48461573 ; 99061573 ; 255471573 ; 91111573 ; 89501573 ; 40461573 ; 319671573 ; 337951573 ; 167771573 ; 371071573 ; 386211573 ; 396951573 ; 403371575 ; 249941575 ; 409601577 ; 15771577 ; 286441578 ; 28171578 ; 16811579 ; 282691579 ; 292021579 ; 91941581 ; 16101581 ; 145801581 ; 258461581 ; 150701581 ; 87271581 ; 295041583 ; 23511584 ; 18141585 ; 317021585 ; 332411586 ; 17361586 ; 225341586 ; 235491586 ; 406551587 ; 20541588 ; 303951588 ; 306001589 ; 17101589 ; 293321589 ; 31701590 ; 253021590 ; 283691590 ; 91981590 ; 406481590 ; 422331592 ; 338911592 ; 398221593 ; 21861593 ; 328491594 ; 406921594 ; 108161595 ; 16591595 ; 22321595 ; 360731595 ; 407791595 ; 82431596 ; 30101596 ; 426411597 ; 82951597 ; 63021598 ; 95291598 ; 21971598 ; 20971598 ; 61541600 ; 69571600 ; 265261600 ; 368541600 ; 396191600 ; 22511600 ; 407191601 ; 256331601 ; 317091601 ; 18101603 ; 245221603 ; 430871604 ; 64101604 ; 42171605 ; 261191605 ; 338521605 ; 382061605 ; 213401605 ; 361831606 ; 17491606 ; 45371606 ; 169071606 ; 382191606 ; 407961606 ; 416271607 ; 16401608 ; 328651608 ; 28921608 ; 89831608 ; 40811608 ; 304971608 ; 390501609 ; 111841610 ; 37731611 ; 73221611 ; 55491612 ; 18221612 ; 24501613 ; 176221613 ; 16191613 ; 54961613 ; 282221613 ; 17631613 ; 388091613 ; 51621613 ; 22551614 ; 57661614 ; 21951614 ; 18731615 ; 16311615 ; 28151615 ; 180721615 ; 17801616 ; 21601617 ; 69251618 ; 24151618 ; 24331619 ; 115941620 ; 16551620 ; 341371621 ; 16511621 ; 329341621 ; 329911621 ; 335231621 ; 393361621 ; 31871622 ; 31911622 ; 293981622 ; 337901622 ; 21461623 ; 119371623 ; 62831623 ; 52211623 ; 238431623 ; 143881623 ; 152381623 ; 260151623 ; 286891623 ; 305061623 ; 87111623 ; 92771623 ; 119381623 ; 166211623 ; 161891624 ; 53781624 ; 18271625 ; 27151625 ; 43111625 ; 143991625 ; 50731625 ; 97711625 ; 333911625 ; 57111626 ; 120231626 ; 186361626 ; 193191626 ; 17151626 ; 369231626 ; 20221627 ; 426411627 ; 280371627 ; 280381628 ; 131131628 ; 199091628 ; 238641628 ; 83411628 ; 347211628 ; 389681629 ; 47521629 ; 17431629 ; 22941629 ; 269951630 ; 215991630 ; 220071630 ; 17261630 ; 17821630 ; 42081630 ; 282431630 ; 56441630 ; 29171630 ; 159311630 ; 300801630 ; 66911632 ; 18321632 ; 99291633 ; 16341634 ; 310791634 ; 397051635 ; 20491635 ; 128281635 ; 23551635 ; 141981635 ; 270781635 ; 280511635 ; 393531636 ; 105281636 ; 106741636 ; 116611637 ; 223941638 ; 89141639 ; 34261639 ; 261671639 ; 104611639 ; 66641641 ; 24291641 ; 73031643 ; 98731643 ; 187131644 ; 230301645 ; 16461645 ; 19161645 ; 59421645 ; 129901645 ; 90601646 ; 427371646 ; 57441646 ; 41291646 ; 44431646 ; 90351646 ; 44731647 ; 94761647 ; 261001647 ; 170011647 ; 18721648 ; 404281648 ; 131411648 ; 254911649 ; 16501649 ; 338291650 ; 258211651 ; 22401651 ; 101191651 ; 83911651 ; 154041651 ; 334181651 ; 399321651 ; 175201652 ; 34691652 ; 270461653 ; 17441653 ; 17431653 ; 214461653 ; 88921655 ; 356421655 ; 157101655 ; 393701656 ; 343321656 ; 116291656 ; 73071657 ; 189701658 ; 285591658 ; 305391659 ; 107731659 ; 40051661 ; 284911661 ; 285531661 ; 290111661 ; 91821661 ; 315131661 ; 121161661 ; 17291661 ; 141161661 ; 236561661 ; 143691661 ; 240651661 ; 246431661 ; 251921661 ; 90061661 ; 255711661 ; 148351661 ; 149611661 ; 282611661 ; 158861661 ; 92061661 ; 329881661 ; 66421661 ; 369521662 ; 22851662 ; 45361664 ; 358381664 ; 31061664 ; 261671664 ; 91531665 ; 18391665 ; 330501666 ; 123831666 ; 64831667 ; 218341668 ; 124121668 ; 36831669 ; 30631669 ; 53551669 ; 51801669 ; 206141669 ; 40391669 ; 399751670 ; 20361670 ; 293231671 ; 141581671 ; 241801672 ; 239071672 ; 77551673 ; 365601673 ; 181951673 ; 205511674 ; 325351675 ; 58551675 ; 63061675 ; 186611675 ; 40741675 ; 68421675 ; 275881675 ; 37431675 ; 330951675 ; 89401675 ; 161911675 ; 341431675 ; 45761675 ; 423081676 ; 37131676 ; 75081676 ; 41851676 ; 142771676 ; 149131676 ; 264431676 ; 77641676 ; 80021676 ; 158811676 ; 33751676 ; 434071676 ; 134701676 ; 428501677 ; 31671677 ; 213761677 ; 31021677 ; 78691677 ; 25311679 ; 310781679 ; 22041680 ; 17031680 ; 25181680 ; 101581680 ; 117011680 ; 440571681 ; 181171681 ; 250771682 ; 62141682 ; 23671682 ; 316151682 ; 19331683 ; 36901683 ; 251351683 ; 252511683 ; 329801684 ; 43451684 ; 74741684 ; 36311684 ; 23381685 ; 74501686 ; 21851686 ; 244731687 ; 17871688 ; 61441688 ; 22281688 ; 31181689 ; 198981689 ; 406791691 ; 230971691 ; 414871692 ; 76061692 ; 132531692 ; 393331692 ; 48301693 ; 17941693 ; 78091694 ; 43761694 ; 53631694 ; 40441694 ; 144881694 ; 40451695 ; 160661695 ; 31291696 ; 69891696 ; 372741696 ; 84971696 ; 134281696 ; 441631696 ; 33141696 ; 57911696 ; 40071697 ; 69201697 ; 63861697 ; 99611697 ; 188361697 ; 30151697 ; 196511697 ; 76691697 ; 65731697 ; 129031697 ; 33361697 ; 354611697 ; 172781698 ; 184491698 ; 85631698 ; 69631698 ; 39661699 ; 25731699 ; 27911699 ; 84411699 ; 76671699 ; 43541699 ; 52691700 ; 342771701 ; 74841701 ; 280231702 ; 265981703 ; 42661703 ; 25181703 ; 225901704 ; 148841704 ; 373971704 ; 17041705 ; 200921705 ; 168561705 ; 392981706 ; 95591706 ; 18761706 ; 109551706 ; 161571706 ; 90491706 ; 81771707 ; 420141708 ; 28671708 ; 29761708 ; 198081708 ; 357151709 ; 45251709 ; 53241709 ; 33191709 ; 128081709 ; 26101709 ; 329491709 ; 405091710 ; 127931710 ; 206981710 ; 337561710 ; 41401712 ; 25241713 ; 17171713 ; 17941715 ; 261811715 ; 392921716 ; 109701716 ; 52901716 ; 434191717 ; 17941717 ; 254201717 ; 316641718 ; 186461719 ; 27491720 ; 17211720 ; 24331720 ; 77321720 ; 18831720 ; 20791720 ; 17871721 ; 18541721 ; 25071721 ; 415041724 ; 17591724 ; 19181725 ; 29481727 ; 311701728 ; 18301729 ; 336051729 ; 408001730 ; 23591730 ; 294011730 ; 307281731 ; 294411731 ; 368041731 ; 398891732 ; 20181732 ; 19861732 ; 223241732 ; 17331733 ; 19861733 ; 408721734 ; 282301735 ; 95601735 ; 20641735 ; 20591735 ; 94731735 ; 83731736 ; 190701737 ; 177991737 ; 193991737 ; 320461738 ; 143631740 ; 408451741 ; 31651741 ; 21911742 ; 122901742 ; 50341742 ; 97501742 ; 97491742 ; 31451742 ; 47511743 ; 122911743 ; 17441744 ; 122911744 ; 131491744 ; 77151744 ; 78501744 ; 97471747 ; 26371747 ; 434571748 ; 294701750 ; 171531750 ; 38021751 ; 17601751 ; 235651751 ; 254771751 ; 183021752 ; 244621752 ; 61131752 ; 410271752 ; 424391752 ; 442711753 ; 198241753 ; 198971753 ; 19201755 ; 404631756 ; 29501756 ; 53721757 ; 260021757 ; 296301757 ; 419271758 ; 74051758 ; 47821758 ; 97251758 ; 62081758 ; 48131758 ; 141841758 ; 82811758 ; 57701759 ; 355191760 ; 125491761 ; 401641761 ; 223221763 ; 81711764 ; 24291765 ; 236521765 ; 27881766 ; 34931766 ; 59001766 ; 120981766 ; 179891766 ; 186001766 ; 186111766 ; 186121766 ; 188351766 ; 19251766 ; 27261766 ; 86371766 ; 60021766 ; 253891766 ; 145211766 ; 145201766 ; 354601767 ; 18021768 ; 316441769 ; 61371769 ; 54231769 ; 221951769 ; 342751769 ; 51401770 ; 18611770 ; 122511770 ; 24361770 ; 408931771 ; 47521771 ; 22941771 ; 57251772 ; 19331773 ; 22691773 ; 64281773 ; 133541773 ; 64291773 ; 285641773 ; 319651773 ; 156071774 ; 242091775 ; 411871775 ; 199841775 ; 333881775 ; 387011776 ; 187931777 ; 53091777 ; 55231777 ; 102921777 ; 53081778 ; 25081778 ; 199171779 ; 25961779 ; 256581779 ; 18031779 ; 302881779 ; 163871780 ; 48261780 ; 145021781 ; 170471781 ; 118451781 ; 231111781 ; 26541781 ; 60341785 ; 20881786 ; 21011786 ; 38761786 ; 20331787 ; 33101787 ; 322611787 ; 54591787 ; 77031787 ; 235521787 ; 95121787 ; 22231787 ; 95141788 ; 198991789 ; 22621789 ; 51251789 ; 86901790 ; 18391790 ; 19771790 ; 176521791 ; 105011792 ; 32331792 ; 43191793 ; 112971793 ; 113581793 ; 80791793 ; 81171793 ; 170321793 ; 31111793 ; 174981795 ; 23141795 ; 425671796 ; 150941796 ; 43361796 ; 19131798 ; 30901798 ; 42861798 ; 28281798 ; 410801799 ; 281021799 ; 282471799 ; 45191800 ; 58511800 ; 132461800 ; 63781800 ; 38031800 ; 105661800 ; 44221801 ; 18231802 ; 340001803 ; 216881803 ; 72421804 ; 18281804 ; 20521804 ; 62521804 ; 247421805 ; 20491805 ; 22721805 ; 381911807 ; 269041809 ; 22341810 ; 290681810 ; 111751810 ; 197961810 ; 400851812 ; 19271812 ; 30651813 ; 28511813 ; 148711814 ; 20041814 ; 75991814 ; 38541815 ; 73731815 ; 22841815 ; 98051815 ; 330001816 ; 30251816 ; 25441816 ; 338591817 ; 97461817 ; 99331817 ; 75211817 ; 85201817 ; 21521817 ; 270641817 ; 275461817 ; 368011818 ; 200241818 ; 31531818 ; 199141818 ; 55961818 ; 278751818 ; 294351818 ; 75161819 ; 242061819 ; 299151820 ; 20521820 ; 62521823 ; 18931823 ; 266041823 ; 314821825 ; 19151826 ; 67181826 ; 156881826 ; 293331827 ; 212761827 ; 337851828 ; 18881829 ; 224031829 ; 306801830 ; 52541830 ; 347611831 ; 20641831 ; 261151831 ; 94731831 ; 83731831 ; 28331831 ; 377441831 ; 434401832 ; 218011832 ; 138831832 ; 366551832 ; 278811832 ; 112421832 ; 137621832 ; 20391832 ; 292051832 ; 158961832 ; 390731832 ; 76461833 ; 23481833 ; 135621834 ; 206061835 ; 20361835 ; 121731835 ; 21071835 ; 62451835 ; 101461835 ; 377661835 ; 382801835 ; 413241836 ; 47701836 ; 315981836 ; 137741837 ; 201441837 ; 22921837 ; 376641837 ; 388971837 ; 26041837 ; 29541838 ; 82281840 ; 43731840 ; 181101840 ; 21231840 ; 343151841 ; 33201841 ; 273341842 ; 100471843 ; 255411845 ; 133301847 ; 209851847 ; 44861847 ; 378941847 ; 387091847 ; 437761848 ; 22321848 ; 236811849 ; 27951849 ; 191561849 ; 332541850 ; 81821850 ; 57201850 ; 82181850 ; 175851850 ; 76931850 ; 39841850 ; 154451850 ; 134261850 ; 166941850 ; 171271850 ; 91261851 ; 381001851 ; 38331852 ; 55351852 ; 21731852 ; 176921852 ; 151561852 ; 279881852 ; 279891854 ; 324091854 ; 60341854 ; 375801855 ; 342281855 ; 90671856 ; 397051856 ; 307771856 ; 321831857 ; 264201857 ; 71351858 ; 44351860 ; 19091861 ; 24351861 ; 182001861 ; 217721862 ; 104481862 ; 145101862 ; 89811862 ; 20781862 ; 412621863 ; 25911863 ; 405381864 ; 122861864 ; 45281864 ; 50531864 ; 47501864 ; 33211864 ; 34431864 ; 96571864 ; 50331864 ; 82201864 ; 73601865 ; 123131865 ; 24811865 ; 186441865 ; 19501865 ; 64931865 ; 29631867 ; 136921867 ; 228001867 ; 27281868 ; 19311868 ; 440911869 ; 40661869 ; 426001869 ; 30211870 ; 186101870 ; 265921870 ; 56131870 ; 36631870 ; 387621871 ; 45431872 ; 26421872 ; 27171872 ; 19921872 ; 43321872 ; 58661872 ; 39501872 ; 407951873 ; 30761875 ; 420231875 ; 37221875 ; 48891876 ; 423301876 ; 18761876 ; 61101876 ; 40231876 ; 97301876 ; 37431876 ; 33391876 ; 33201876 ; 155381876 ; 61531877 ; 25731877 ; 62071877 ; 411911878 ; 191211878 ; 236431878 ; 143961879 ; 135351879 ; 30561879 ; 374701880 ; 45241880 ; 122311880 ; 69421880 ; 82161880 ; 29181881 ; 241821881 ; 272931881 ; 19791882 ; 33671882 ; 201411882 ; 57151882 ; 412181882 ; 429911883 ; 45701884 ; 27691885 ; 137711885 ; 123901885 ; 209211885 ; 35601886 ; 30761886 ; 207091886 ; 25751886 ; 153041886 ; 21511886 ; 135651886 ; 367121887 ; 21561889 ; 20151889 ; 28421889 ; 405151890 ; 19201891 ; 58421891 ; 20511891 ; 196061891 ; 64561891 ; 146081891 ; 152321891 ; 259971891 ; 41301892 ; 103361892 ; 22761892 ; 282811893 ; 286181893 ; 292721893 ; 329551894 ; 246051894 ; 341001894 ; 373301894 ; 421221895 ; 205461895 ; 86591895 ; 335351895 ; 343501895 ; 419921896 ; 21881897 ; 59891897 ; 60961898 ; 58321898 ; 30031898 ; 142111898 ; 33431898 ; 82391898 ; 74441898 ; 58331898 ; 430751899 ; 156591899 ; 313971899 ; 23731900 ; 401611901 ; 337311902 ; 272401902 ; 112171903 ; 209001904 ; 314971904 ; 385761905 ; 133961906 ; 22211906 ; 315861908 ; 30501908 ; 77041908 ; 323021908 ; 41251908 ; 265471908 ; 20681908 ; 438611909 ; 45241909 ; 122311909 ; 29181909 ; 69421909 ; 82161910 ; 23791910 ; 252391911 ; 30471911 ; 414761912 ; 57291912 ; 336321912 ; 393311912 ; 421301914 ; 32281914 ; 21891914 ; 291501915 ; 23041915 ; 357311916 ; 61691916 ; 34521916 ; 57431916 ; 96421916 ; 19461916 ; 36571917 ; 442341919 ; 32351919 ; 203881919 ; 375501919 ; 420281919 ; 56111920 ; 318981921 ; 265641923 ; 195981925 ; 32471925 ; 136661925 ; 160641926 ; 249181927 ; 86271927 ; 41391927 ; 400631928 ; 26661928 ; 280521928 ; 350891932 ; 118341932 ; 349761932 ; 35161933 ; 276471933 ; 299581934 ; 144861934 ; 256721935 ; 108281935 ; 68021936 ; 119891936 ; 283451936 ; 46771936 ; 46191936 ; 420361937 ; 38761937 ; 19551937 ; 98761939 ; 21951939 ; 366521940 ; 366311941 ; 58511941 ; 387371942 ; 123181942 ; 20031942 ; 69611942 ; 133621942 ; 35131942 ; 212321942 ; 137521942 ; 139021942 ; 66151942 ; 92421944 ; 37281944 ; 123651945 ; 20761945 ; 67521945 ; 50221945 ; 236351945 ; 43621946 ; 25681946 ; 22961946 ; 25611946 ; 40221946 ; 44721946 ; 40311948 ; 303941949 ; 35821951 ; 207681951 ; 171161951 ; 420661952 ; 205661952 ; 204161953 ; 20101953 ; 196051954 ; 352971954 ; 441021955 ; 41671957 ; 414571957 ; 62951957 ; 25311958 ; 195401961 ; 117361961 ; 117371961 ; 20231961 ; 253421962 ; 77051962 ; 23711962 ; 424191963 ; 33681964 ; 37171964 ; 68691964 ; 82281966 ; 200681966 ; 156171966 ; 394961966 ; 424261967 ; 20901967 ; 155671968 ; 21421968 ; 215891968 ; 138411968 ; 30851968 ; 416351969 ; 199131969 ; 57691973 ; 78121973 ; 67191973 ; 70051975 ; 19761975 ; 59311976 ; 123171976 ; 57271976 ; 73661976 ; 25291976 ; 38361976 ; 270421976 ; 85931976 ; 115801976 ; 365991976 ; 421171976 ; 442801979 ; 100671979 ; 437321980 ; 282231980 ; 158801981 ; 281341983 ; 235461983 ; 145171983 ; 89741983 ; 243971983 ; 258041983 ; 438551984 ; 40621984 ; 27471984 ; 220121984 ; 225931984 ; 19841985 ; 201101985 ; 376551986 ; 435231986 ; 329831989 ; 25581989 ; 290561989 ; 28321992 ; 207671992 ; 207681992 ; 142831993 ; 108281994 ; 22881995 ; 118571995 ; 377541995 ; 172711995 ; 116921996 ; 177621996 ; 19961996 ; 391711997 ; 266421997 ; 339831997 ; 370281998 ; 216411999 ; 237742000 ; 157572001 ; 286822001 ; 289492002 ; 227782002 ; 363042002 ; 64022003 ; 82232003 ; 47572003 ; 175832003 ; 59402003 ; 57282003 ; 53332003 ; 162532003 ; 175842004 ; 252302005 ; 419332005 ; 413012006 ; 245852007 ; 264882007 ; 74832007 ; 87222008 ; 166922008 ; 23142009 ; 45342009 ; 40272009 ; 57532009 ; 57622009 ; 392172010 ; 278622010 ; 25442011 ; 23932011 ; 48032011 ; 308842013 ; 25792013 ; 53892013 ; 33152013 ; 78592014 ; 25632014 ; 32522014 ; 148772014 ; 272192014 ; 83092014 ; 303032014 ; 336842014 ; 427822015 ; 373102017 ; 21082018 ; 364602018 ; 367272019 ; 222962019 ; 26662019 ; 390382019 ; 419682019 ; 425632021 ; 383292022 ; 321742023 ; 20892023 ; 117362023 ; 21312024 ; 48772024 ; 77332024 ; 27652024 ; 87712024 ; 143132024 ; 39572024 ; 426092025 ; 90202025 ; 45482028 ; 119372028 ; 62832028 ; 238432028 ; 143882028 ; 152382028 ; 260152028 ; 286892028 ; 305062028 ; 87112028 ; 92772028 ; 119382028 ; 166212028 ; 161892028 ; 87102028 ; 242702028 ; 275752028 ; 278062028 ; 280042028 ; 318592028 ; 440072029 ; 281252029 ; 34162030 ; 20302030 ; 388182030 ; 431942030 ; 434182031 ; 20672032 ; 26262033 ; 21352033 ; 140802034 ; 134292035 ; 240892035 ; 168102036 ; 155182038 ; 48502038 ; 409782039 ; 158962039 ; 187162039 ; 41982039 ; 396032040 ; 23642040 ; 55872040 ; 42982040 ; 40882041 ; 68682041 ; 436982044 ; 23552045 ; 22332045 ; 21602045 ; 25552045 ; 439702045 ; 347092046 ; 378232047 ; 353452048 ; 332462048 ; 41262048 ; 162962049 ; 64532049 ; 43492049 ; 84102050 ; 45332050 ; 291202050 ; 293862051 ; 24042053 ; 205962053 ; 328982055 ; 88462055 ; 255822056 ; 22732057 ; 21312057 ; 225532057 ; 77312058 ; 250332058 ; 33262058 ; 25462059 ; 95602059 ; 79212059 ; 42682059 ; 32922059 ; 159112059 ; 144432059 ; 173042059 ; 396132060 ; 39712060 ; 75972060 ; 211542060 ; 107112060 ; 43552061 ; 286502061 ; 22722061 ; 244252061 ; 160482061 ; 385252062 ; 253392062 ; 37192062 ; 70512063 ; 118272063 ; 198852063 ; 419462065 ; 55442065 ; 33282065 ; 30772065 ; 59242065 ; 59612066 ; 26102066 ; 27662066 ; 291382067 ; 21672068 ; 22422068 ; 105042068 ; 143172068 ; 80322068 ; 20682069 ; 80122069 ; 311922070 ; 21852071 ; 397362072 ; 32822073 ; 178372073 ; 54392073 ; 130142073 ; 33522073 ; 80652073 ; 173642074 ; 274842075 ; 234902075 ; 27002075 ; 89822075 ; 31282076 ; 278252077 ; 422432078 ; 30432078 ; 266502079 ; 382382079 ; 49772080 ; 24202080 ; 378842081 ; 64432081 ; 50622081 ; 40462081 ; 106072081 ; 49722081 ; 96682081 ; 43162081 ; 276512081 ; 26472082 ; 29992082 ; 261222082 ; 23192083 ; 230942083 ; 20832085 ; 237952085 ; 387692086 ; 25822086 ; 22062086 ; 302452087 ; 97052088 ; 434742088 ; 20882089 ; 21312089 ; 253432090 ; 51222090 ; 37762090 ; 27832090 ; 428602091 ; 21702091 ; 44912091 ; 29262092 ; 440802093 ; 212882093 ; 72942094 ; 88252094 ; 377652095 ; 187582095 ; 75602095 ; 383072095 ; 389792096 ; 78242096 ; 46762096 ; 406132097 ; 314792097 ; 283632098 ; 218052098 ; 352892098 ; 26812098 ; 34562100 ; 29062101 ; 86102102 ; 331522103 ; 36812104 ; 235802104 ; 296452104 ; 23982105 ; 223932106 ; 247112106 ; 273272107 ; 33142107 ; 40072107 ; 121742107 ; 155182108 ; 21872108 ; 262742111 ; 34762111 ; 312492112 ; 37392112 ; 49702115 ; 339872116 ; 228032117 ; 43682117 ; 295612117 ; 85712117 ; 76162118 ; 352352118 ; 405402119 ; 39882119 ; 49882119 ; 436782120 ; 45692120 ; 21212120 ; 212212120 ; 55632120 ; 37882120 ; 236902121 ; 21222123 ; 252992124 ; 157452126 ; 59112126 ; 32532126 ; 33562126 ; 64182127 ; 59112127 ; 32532127 ; 64182128 ; 429212130 ; 160372131 ; 30432132 ; 349662132 ; 24292132 ; 118722132 ; 94812132 ; 71352133 ; 36512134 ; 370492134 ; 421002135 ; 328762135 ; 72742136 ; 122602136 ; 275902136 ; 159272136 ; 61102137 ; 21382137 ; 26132138 ; 21392139 ; 21802139 ; 230262140 ; 21802140 ; 21812140 ; 230272140 ; 334602141 ; 21812141 ; 334612142 ; 78342143 ; 28612143 ; 24482143 ; 131752143 ; 207312143 ; 124072143 ; 111832143 ; 76022143 ; 29952144 ; 35852144 ; 22562146 ; 31912146 ; 26852151 ; 61882151 ; 262502151 ; 324492152 ; 200782152 ; 108842152 ; 373052152 ; 375272152 ; 115912154 ; 260312155 ; 96912155 ; 85962155 ; 32632155 ; 59852155 ; 50812155 ; 259232155 ; 58002155 ; 29522155 ; 59412155 ; 279422155 ; 103832155 ; 24982155 ; 115532155 ; 115542156 ; 34412157 ; 83192157 ; 54582158 ; 320142161 ; 39082162 ; 373302163 ; 67622163 ; 87302163 ; 388052164 ; 24492164 ; 437052166 ; 412622166 ; 41412166 ; 52392166 ; 368602167 ; 428852168 ; 21692169 ; 22122169 ; 431572170 ; 91002171 ; 24652171 ; 207662171 ; 222822172 ; 88072172 ; 22232172 ; 39632174 ; 24752174 ; 252042175 ; 412472177 ; 124152178 ; 255762178 ; 391242179 ; 267112182 ; 216412182 ; 79852182 ; 332132182 ; 383342183 ; 38382183 ; 168382183 ; 116372183 ; 394582183 ; 407392183 ; 28452183 ; 433222184 ; 75752185 ; 24202185 ; 235862185 ; 246882186 ; 265352186 ; 76412186 ; 284312186 ; 289652187 ; 48352188 ; 372622189 ; 41282189 ; 105482189 ; 274982192 ; 243262192 ; 109552192 ; 27302193 ; 254892194 ; 309462194 ; 406902195 ; 25752195 ; 329672197 ; 316152197 ; 24622198 ; 23062198 ; 242352198 ; 289542198 ; 53812199 ; 22242200 ; 23282201 ; 58682201 ; 223362202 ; 65612203 ; 75572203 ; 155232203 ; 69122204 ; 151132204 ; 300602205 ; 27222205 ; 25822205 ; 84082205 ; 302442206 ; 84492206 ; 27972206 ; 98842206 ; 69152206 ; 253752207 ; 32162208 ; 24752208 ; 54882208 ; 49802208 ; 38392209 ; 224172209 ; 45202209 ; 143492211 ; 48882211 ; 22112211 ; 253402213 ; 106822213 ; 48672215 ; 132332215 ; 292832216 ; 50962216 ; 29282217 ; 27862217 ; 128972220 ; 395312221 ; 54952222 ; 286732224 ; 22672225 ; 22332226 ; 107322226 ; 35322226 ; 39112226 ; 367472228 ; 270002228 ; 152332228 ; 31032231 ; 50962231 ; 288292232 ; 74392232 ; 25402233 ; 439702234 ; 39492235 ; 29562239 ; 343532239 ; 197092239 ; 201042239 ; 266372239 ; 69912239 ; 333722239 ; 339972239 ; 358222240 ; 329332241 ; 105902241 ; 27032241 ; 94012242 ; 171412242 ; 383712244 ; 22812244 ; 240072244 ; 295812244 ; 129382244 ; 172802245 ; 283212246 ; 223282247 ; 23472247 ; 328882247 ; 344112247 ; 344122249 ; 250792250 ; 325932250 ; 67522250 ; 42112251 ; 22512251 ; 253122252 ; 35822254 ; 69622254 ; 76102254 ; 133642254 ; 23782254 ; 106562254 ; 258172254 ; 270432254 ; 26342255 ; 71702255 ; 407592256 ; 107512258 ; 54282258 ; 259292258 ; 287152259 ; 71672260 ; 157732260 ; 27202261 ; 27722262 ; 107262262 ; 184862263 ; 57312263 ; 40372263 ; 82282263 ; 147102263 ; 108792264 ; 27632264 ; 55092265 ; 67952265 ; 321792266 ; 421282266 ; 421292268 ; 33312268 ; 226522268 ; 276322268 ; 90182268 ; 43572269 ; 383562270 ; 276482271 ; 23892271 ; 37702271 ; 74962272 ; 23512274 ; 201572274 ; 346372274 ; 441442275 ; 48452275 ; 102792275 ; 157852277 ; 40462277 ; 26472278 ; 317162278 ; 155152278 ; 419752279 ; 66712279 ; 432932280 ; 33082281 ; 56732281 ; 85282281 ; 49902281 ; 279432281 ; 60272281 ; 43592281 ; 172142281 ; 388072282 ; 314262284 ; 103162284 ; 339112284 ; 340272285 ; 366102286 ; 206142286 ; 35742287 ; 237452287 ; 402972288 ; 61742288 ; 137712288 ; 109232288 ; 330922288 ; 361152291 ; 141662292 ; 407392292 ; 265582293 ; 122822293 ; 58702293 ; 34782293 ; 138322293 ; 262662293 ; 273132293 ; 325752295 ; 310472296 ; 278272296 ; 26162297 ; 28022298 ; 33282299 ; 23002299 ; 388272302 ; 72362302 ; 290022303 ; 24432303 ; 181342303 ; 134812303 ; 235052303 ; 251882303 ; 267202303 ; 115072303 ; 144652303 ; 174922305 ; 105102305 ; 256602306 ; 152942307 ; 323542307 ; 23592308 ; 23502308 ; 45592308 ; 130102308 ; 47962308 ; 313432312 ; 38082312 ; 30262312 ; 104982312 ; 157882313 ; 31752313 ; 423182314 ; 66062314 ; 410722315 ; 199032315 ; 173452317 ; 263612317 ; 368292317 ; 24942317 ; 429902318 ; 23602318 ; 418592318 ; 65062319 ; 219982319 ; 69042322 ; 23232322 ; 27132322 ; 117212322 ; 93302326 ; 200222326 ; 386492326 ; 86522327 ; 266872327 ; 356912327 ; 391532328 ; 186592328 ; 101332328 ; 28262328 ; 25392328 ; 48962333 ; 178392333 ; 337882334 ; 101802334 ; 337652334 ; 348132334 ; 33782335 ; 28432335 ; 122862335 ; 57232335 ; 50532335 ; 34432335 ; 84282335 ; 82202335 ; 50322335 ; 50332336 ; 151222337 ; 82412338 ; 103312338 ; 49432338 ; 29352338 ; 151202339 ; 44292340 ; 47242340 ; 143452340 ; 261112341 ; 131112341 ; 151092343 ; 224812344 ; 112402345 ; 261122346 ; 29282346 ; 24922346 ; 53572346 ; 127322346 ; 310602350 ; 296642350 ; 131052351 ; 55722351 ; 130042351 ; 426032352 ; 25722352 ; 102152352 ; 312812352 ; 74242352 ; 399682353 ; 202342353 ; 137052353 ; 436022354 ; 32542354 ; 132522354 ; 85582354 ; 262142354 ; 27032354 ; 431562355 ; 54682355 ; 107202355 ; 145672356 ; 296792356 ; 349132357 ; 50852358 ; 434992359 ; 155502359 ; 332732359 ; 413752360 ; 438082360 ; 418132361 ; 33962361 ; 247532362 ; 41562363 ; 329852364 ; 324792365 ; 432492366 ; 229412366 ; 328232366 ; 428612367 ; 125652367 ; 366392368 ; 247672368 ; 250212368 ; 391052370 ; 40242370 ; 349732370 ; 378372371 ; 57212371 ; 89592371 ; 35762373 ; 73202374 ; 242742374 ; 307462376 ; 377292378 ; 167902378 ; 26342379 ; 95562379 ; 59882379 ; 53262380 ; 25522380 ; 57242381 ; 374442383 ; 25782383 ; 207972383 ; 140932384 ; 29222384 ; 50472384 ; 110222384 ; 276342384 ; 388042385 ; 74892385 ; 340842385 ; 372362387 ; 209102387 ; 216232388 ; 29072389 ; 99682392 ; 24442392 ; 260182393 ; 24632393 ; 27342394 ; 373102394 ; 217342395 ; 45352395 ; 53242395 ; 304612395 ; 370942396 ; 50982398 ; 158842399 ; 29252399 ; 34722399 ; 295962399 ; 34652399 ; 82522399 ; 97232399 ; 415832401 ; 368262402 ; 30222402 ; 86382402 ; 167582402 ; 40922402 ; 407972404 ; 41302404 ; 205792405 ; 26222406 ; 86592406 ; 90542406 ; 334572407 ; 66592408 ; 29402409 ; 204752409 ; 329472409 ; 442522411 ; 236412414 ; 256512416 ; 51382416 ; 76152416 ; 128512417 ; 33152417 ; 57842419 ; 267732419 ; 94752420 ; 42162421 ; 200472421 ; 27432421 ; 329512422 ; 119562422 ; 37852422 ; 42842423 ; 119562423 ; 24242423 ; 37852423 ; 42842424 ; 50082424 ; 37232424 ; 74782425 ; 50082425 ; 25382425 ; 37232425 ; 97802426 ; 119822426 ; 132712426 ; 31272426 ; 252742427 ; 286412428 ; 132552430 ; 224472430 ; 354492431 ; 27462431 ; 437492432 ; 108982433 ; 26092434 ; 77022436 ; 122512436 ; 24372439 ; 24782439 ; 98362440 ; 385072440 ; 390072440 ; 390082440 ; 398442441 ; 92662441 ; 366422441 ; 429252442 ; 365612443 ; 50722443 ; 86652443 ; 55032443 ; 55042443 ; 115072443 ; 174922443 ; 435362443 ; 189872443 ; 24432443 ; 319962443 ; 108132445 ; 104562445 ; 141812445 ; 244482445 ; 270442446 ; 24602446 ; 25862447 ; 236762447 ; 32652448 ; 25782449 ; 30042449 ; 55982451 ; 24522451 ; 45272451 ; 64312451 ; 90372452 ; 25032453 ; 199112453 ; 200152453 ; 207692453 ; 352982456 ; 28682456 ; 27222456 ; 192892456 ; 302432457 ; 77612457 ; 221702457 ; 59972459 ; 49372459 ; 106972459 ; 155432460 ; 41242461 ; 316212461 ; 329772461 ; 37252461 ; 172862462 ; 28382462 ; 386672463 ; 48032464 ; 27252464 ; 206072464 ; 135212464 ; 334242464 ; 438242467 ; 56682467 ; 158622470 ; 126212470 ; 200682470 ; 424262471 ; 361732471 ; 30062473 ; 29152474 ; 394812475 ; 29032475 ; 40572475 ; 271152475 ; 54882475 ; 295522475 ; 312192475 ; 375232476 ; 60862476 ; 29722476 ; 432312478 ; 48802478 ; 51942478 ; 347362478 ; 292312478 ; 332322479 ; 209252480 ; 316162480 ; 248882480 ; 151182481 ; 57262481 ; 403552482 ; 403892483 ; 135942484 ; 124172484 ; 24852484 ; 40602484 ; 133472484 ; 67672484 ; 112142485 ; 85052485 ; 306682485 ; 183482485 ; 159522486 ; 106962486 ; 172692486 ; 160942486 ; 39592486 ; 90702487 ; 335142488 ; 41322488 ; 137722488 ; 265822488 ; 341762489 ; 289582492 ; 28522492 ; 72192492 ; 317312495 ; 27862496 ; 250032497 ; 25872497 ; 154292497 ; 338282497 ; 80912497 ; 368102499 ; 36612499 ; 315452499 ; 86032500 ; 95732503 ; 56682504 ; 42132507 ; 380422507 ; 389232507 ; 245572507 ; 421132509 ; 29452510 ; 263852510 ; 334582510 ; 418542511 ; 372142511 ; 392492511 ; 26322513 ; 186272513 ; 368442514 ; 323972514 ; 363132514 ; 115792517 ; 199922517 ; 202972517 ; 337862517 ; 442412517 ; 442422518 ; 42662518 ; 94552518 ; 138442519 ; 376992519 ; 379882520 ; 27092523 ; 418292524 ; 34792524 ; 200132524 ; 216142524 ; 224712525 ; 226772525 ; 266032525 ; 395012525 ; 441582526 ; 32012526 ; 227682527 ; 341902528 ; 29712529 ; 139012529 ; 139022529 ; 66152529 ; 38362529 ; 162522529 ; 35132529 ; 85932529 ; 115802529 ; 365992529 ; 369122529 ; 421172529 ; 442802530 ; 87832530 ; 67842530 ; 125562530 ; 293352530 ; 146582530 ; 73722531 ; 206632533 ; 284442533 ; 136952534 ; 343402535 ; 41562536 ; 382452537 ; 216082537 ; 225392538 ; 182062543 ; 353662543 ; 358882544 ; 41152545 ; 381052546 ; 331032546 ; 110362547 ; 391032548 ; 329462548 ; 376602548 ; 266132548 ; 346302549 ; 212692550 ; 392582550 ; 433112552 ; 48802552 ; 51942552 ; 143162552 ; 347362552 ; 116062554 ; 144732555 ; 30882555 ; 38932555 ; 30462555 ; 441572556 ; 424392556 ; 61382558 ; 39262560 ; 160932560 ; 359952563 ; 53432563 ; 75122563 ; 171262563 ; 428182564 ; 57122564 ; 216582564 ; 143922565 ; 269872565 ; 282342565 ; 296172565 ; 307372568 ; 61692568 ; 34522568 ; 96422568 ; 269052569 ; 139242569 ; 39052569 ; 104792570 ; 25712570 ; 45692570 ; 236902570 ; 37882570 ; 28562571 ; 26582571 ; 189462571 ; 235402572 ; 362912574 ; 26552575 ; 30762575 ; 378962576 ; 34442577 ; 26562579 ; 38642579 ; 276592579 ; 58392579 ; 216912579 ; 159752581 ; 218182581 ; 396832582 ; 387962582 ; 387972583 ; 28222584 ; 32472584 ; 383972584 ; 64342587 ; 106962587 ; 323812587 ; 39592587 ; 90002587 ; 54772587 ; 90702587 ; 101202587 ; 79912587 ; 56462587 ; 49542588 ; 424672589 ; 383292589 ; 357402589 ; 396472589 ; 398882589 ; 405792590 ; 347852592 ; 429172595 ; 154992595 ; 166242597 ; 46002597 ; 128512598 ; 42652598 ; 30932598 ; 28992598 ; 257462599 ; 75482599 ; 279582600 ; 63132603 ; 280612604 ; 27432605 ; 38302606 ; 385952610 ; 34652610 ; 291322610 ; 318562611 ; 40122611 ; 35912611 ; 101252611 ; 213742611 ; 151532611 ; 92382611 ; 408822611 ; 427552612 ; 132832612 ; 71662613 ; 209462613 ; 381892616 ; 35922616 ; 212222616 ; 103542617 ; 98912619 ; 26202620 ; 26212622 ; 260692622 ; 97582622 ; 307262623 ; 195072626 ; 86712626 ; 330492629 ; 199402629 ; 264772630 ; 139162630 ; 33642630 ; 59342632 ; 386702633 ; 146172633 ; 57212633 ; 28262633 ; 35762634 ; 122592634 ; 316102635 ; 145182638 ; 205512638 ; 162262638 ; 93732640 ; 26412642 ; 204352642 ; 410642646 ; 200732646 ; 329522646 ; 399942647 ; 64432647 ; 41572650 ; 58042650 ; 62492650 ; 85982650 ; 37772652 ; 249442653 ; 40332653 ; 262472654 ; 335992655 ; 48152655 ; 137302655 ; 222662656 ; 54072656 ; 32412657 ; 26582657 ; 38642657 ; 58392658 ; 189462659 ; 211152661 ; 65412661 ; 85102662 ; 35322663 ; 133612663 ; 33992663 ; 235332663 ; 425942664 ; 198342664 ; 382982665 ; 54582665 ; 32522666 ; 46012666 ; 140342666 ; 88082668 ; 28712672 ; 223152673 ; 265012677 ; 43832677 ; 42932679 ; 340982679 ; 27122680 ; 400962681 ; 352882682 ; 54942684 ; 167612688 ; 286432688 ; 310682691 ; 253442692 ; 340462693 ; 179552693 ; 27742693 ; 57212693 ; 313302696 ; 33952696 ; 425012697 ; 377452697 ; 419142697 ; 382552700 ; 158482703 ; 325782703 ; 401062703 ; 424802704 ; 162892704 ; 412562704 ; 428572705 ; 416072706 ; 28882706 ; 27582707 ; 132902707 ; 353132709 ; 359732710 ; 50742710 ; 34582712 ; 340982712 ; 411542714 ; 169702715 ; 27152716 ; 97692720 ; 285662721 ; 348912721 ; 367192722 ; 385662723 ; 202222723 ; 436352724 ; 130462725 ; 78602726 ; 71122726 ; 94062726 ; 384382726 ; 384822727 ; 110642727 ; 112202727 ; 129142727 ; 344732729 ; 190272729 ; 190282729 ; 61422729 ; 195032729 ; 141872730 ; 394342730 ; 402272731 ; 435542733 ; 54922733 ; 35502733 ; 349802736 ; 56752736 ; 406382740 ; 335702740 ; 81732741 ; 28812741 ; 204602741 ; 314952741 ; 314962741 ; 163672741 ; 350652741 ; 80432741 ; 401862741 ; 413982741 ; 434812746 ; 366822746 ; 31012747 ; 220122747 ; 27472748 ; 368762749 ; 33412749 ; 244222750 ; 79322753 ; 315242756 ; 418772756 ; 164832759 ; 31482759 ; 48102759 ; 198002759 ; 207012759 ; 34302761 ; 201912761 ; 46382762 ; 31242763 ; 77582765 ; 425952766 ; 317852766 ; 324362767 ; 77692768 ; 281712770 ; 441672771 ; 29572773 ; 61032773 ; 67452774 ; 332362776 ; 187212776 ; 139462777 ; 220052778 ; 329662779 ; 28882781 ; 29892782 ; 171542783 ; 37762784 ; 27842784 ; 62042788 ; 53072789 ; 231262789 ; 31632789 ; 79022789 ; 63872789 ; 286012789 ; 111342789 ; 111722789 ; 111732789 ; 320232789 ; 339252791 ; 79512791 ; 279852794 ; 270502795 ; 342052795 ; 57382795 ; 432302797 ; 292582798 ; 36392798 ; 54422798 ; 189032798 ; 140612804 ; 197222804 ; 387882805 ; 376752808 ; 48492808 ; 371742812 ; 357042816 ; 439712817 ; 400312818 ; 28872818 ; 316842819 ; 236622820 ; 36862822 ; 29532822 ; 262952822 ; 361182823 ; 82912823 ; 45962823 ; 128962824 ; 73072824 ; 255162827 ; 430832828 ; 51592828 ; 287532829 ; 343662829 ; 394102829 ; 406262829 ; 46212831 ; 314982832 ; 39962832 ; 120962832 ; 67632832 ; 233292832 ; 393972832 ; 417712833 ; 333242833 ; 340412833 ; 114272834 ; 367442834 ; 94122836 ; 174022837 ; 217112837 ; 224672837 ; 112902837 ; 341692838 ; 47612841 ; 78552848 ; 79912848 ; 96122848 ; 74852848 ; 254812848 ; 157042849 ; 296782849 ; 172762850 ; 29922850 ; 35942850 ; 47562851 ; 291962856 ; 31612856 ; 53862856 ; 387522856 ; 391312856 ; 37782856 ; 425932857 ; 54452858 ; 97162858 ; 83202858 ; 37822858 ; 74302858 ; 31042860 ; 42212861 ; 83372862 ; 62952862 ; 38402863 ; 290992863 ; 300592866 ; 38132869 ; 30432869 ; 28692869 ; 85552874 ; 368722874 ; 384242876 ; 441152877 ; 419442878 ; 341202883 ; 115332883 ; 371032886 ; 266422891 ; 306812891 ; 306822892 ; 429072893 ; 378662893 ; 363682896 ; 28972896 ; 369412896 ; 369782897 ; 98172897 ; 201112897 ; 48702897 ; 369412897 ; 369782898 ; 29342899 ; 76492899 ; 257462899 ; 44902899 ; 340222900 ; 284432900 ; 32942901 ; 146002901 ; 68502901 ; 397702902 ; 47272902 ; 77242903 ; 397592904 ; 148932904 ; 32062905 ; 40532906 ; 119012907 ; 30102909 ; 241312909 ; 41582910 ; 29542911 ; 29822911 ; 366282912 ; 53042912 ; 35802912 ; 35812913 ; 258152913 ; 36502913 ; 432502914 ; 121402914 ; 381062915 ; 121622915 ; 33932917 ; 174312922 ; 43082922 ; 410512923 ; 124622923 ; 177402923 ; 57532923 ; 57622923 ; 48452925 ; 182012925 ; 365912925 ; 415552926 ; 30312926 ; 32032926 ; 223622926 ; 69032926 ; 56722926 ; 104392926 ; 117892926 ; 65432926 ; 85082927 ; 45022929 ; 301142930 ; 300182931 ; 250882933 ; 30012934 ; 30012934 ; 136442934 ; 276382936 ; 125922936 ; 29462936 ; 60332937 ; 83202937 ; 37822938 ; 37072940 ; 32052940 ; 291152941 ; 202902943 ; 68522943 ; 193942944 ; 386152944 ; 40052944 ; 441702945 ; 255262946 ; 225952948 ; 225812948 ; 131802949 ; 162062950 ; 251942950 ; 253102951 ; 31982951 ; 290622951 ; 384082951 ; 385292951 ; 398772952 ; 31952952 ; 131072952 ; 97872952 ; 350432954 ; 53242955 ; 379902956 ; 34642956 ; 54752956 ; 433022959 ; 352732959 ; 388422960 ; 32152961 ; 267922961 ; 296102963 ; 392562964 ; 338762969 ; 268992969 ; 270692970 ; 34172970 ; 31462972 ; 54382972 ; 223332972 ; 272752974 ; 29752974 ; 52012974 ; 250932976 ; 69302976 ; 32942977 ; 123852977 ; 404802978 ; 439882982 ; 56042984 ; 154212985 ; 81162985 ; 50172985 ; 43372985 ; 34872985 ; 50182985 ; 173822986 ; 162752992 ; 61552994 ; 32362994 ; 34612994 ; 136472994 ; 103122994 ; 125912995 ; 76922995 ; 95812995 ; 30552995 ; 61932995 ; 101732995 ; 266782995 ; 162562995 ; 402032996 ; 82322996 ; 35982996 ; 134782996 ; 134112997 ; 325412998 ; 38052998 ; 128492998 ; 394042998 ; 409512999 ; 312593000 ; 58403000 ; 53193004 ; 93803006 ; 132063006 ; 50663007 ; 34883007 ; 172403007 ; 98323007 ; 30083008 ; 43523008 ; 43693010 ; 210383011 ; 343323011 ; 251943012 ; 115793013 ; 341903013 ; 206313013 ; 89693016 ; 381283016 ; 392813016 ; 394593016 ; 433243018 ; 267153018 ; 329793018 ; 430253019 ; 60773021 ; 400933021 ; 412023024 ; 413623027 ; 316543028 ; 101213029 ; 31093029 ; 30303029 ; 413773032 ; 30333032 ; 178443032 ; 34903033 ; 34903034 ; 442333034 ; 139103042 ; 38243043 ; 53253044 ; 238283045 ; 419733051 ; 31413051 ; 406223052 ; 404583052 ; 420773053 ; 318693057 ; 253923057 ; 97943057 ; 337613059 ; 254803059 ; 148153061 ; 297453061 ; 392843062 ; 416293066 ; 412593067 ; 183993069 ; 172753070 ; 83233070 ; 265343070 ; 114783070 ; 85723071 ; 83313071 ; 74353072 ; 43573072 ; 90183073 ; 33473073 ; 389443075 ; 100293075 ; 97293076 ; 153023077 ; 73783083 ; 59793084 ; 175373087 ; 43683087 ; 191113087 ; 309083088 ; 289593088 ; 160713088 ; 86703090 ; 51593090 ; 43723090 ; 93743091 ; 202963092 ; 373133093 ; 269843095 ; 347573097 ; 377223098 ; 160183098 ; 66803098 ; 442093098 ; 134803098 ; 202703098 ; 110033098 ; 338263098 ; 160173098 ; 376203098 ; 397913098 ; 420573098 ; 435343099 ; 389303100 ; 160943103 ; 47543103 ; 244633103 ; 259333103 ; 316763103 ; 148143106 ; 51923108 ; 39963108 ; 302113108 ; 163503108 ; 68123108 ; 125973108 ; 58093108 ; 66943108 ; 64053109 ; 179653109 ; 195373110 ; 69053110 ; 57343110 ; 84303110 ; 73813111 ; 370853112 ; 388433112 ; 406433113 ; 285593117 ; 348853120 ; 198993120 ; 441803121 ; 383763125 ; 224243126 ; 307673127 ; 252743127 ; 240363128 ; 32643128 ; 229393130 ; 53363130 ; 337173131 ; 92933131 ; 366183131 ; 52743132 ; 241243135 ; 39763136 ; 146963146 ; 35343147 ; 310313148 ; 198003148 ; 207013151 ; 40273151 ; 60263151 ; 69193151 ; 331283151 ; 393733152 ; 412873154 ; 220873156 ; 66743156 ; 81423156 ; 65953157 ; 47863157 ; 65653157 ; 256313157 ; 311533158 ; 207473159 ; 76383161 ; 407893164 ; 32403169 ; 154213169 ; 330213169 ; 34323172 ; 58863172 ; 111563173 ; 111033173 ; 289683174 ; 43553174 ; 410443178 ; 67433186 ; 358103187 ; 329913187 ; 393363187 ; 68693191 ; 315183192 ; 400023193 ; 363853194 ; 213113195 ; 36483195 ; 57833198 ; 113683198 ; 116543198 ; 239813198 ; 239823198 ; 71953198 ; 60913198 ; 283743198 ; 305883198 ; 310303198 ; 112233198 ; 311863198 ; 312993198 ; 110923198 ; 346653198 ; 346663198 ; 351063198 ; 351073198 ; 75863198 ; 75853201 ; 52823201 ; 422933202 ; 107153203 ; 347633203 ; 167713205 ; 49743205 ; 305833205 ; 98073205 ; 326243205 ; 156043206 ; 358873208 ; 61173208 ; 227853208 ; 145053208 ; 90753208 ; 272273208 ; 336153208 ; 396053209 ; 416263210 ; 81823212 ; 244593212 ; 32963214 ; 95053215 ; 36513215 ; 424623217 ; 314813218 ; 267003221 ; 55443222 ; 146343223 ; 39673223 ; 288623227 ; 295433227 ; 304953227 ; 413123228 ; 332903228 ; 418803229 ; 296573229 ; 308663230 ; 331213230 ; 154393234 ; 314903235 ; 38223235 ; 244213238 ; 274583239 ; 202403240 ; 224383240 ; 230773241 ; 33443241 ; 243923244 ; 47243245 ; 45883245 ; 86363245 ; 147003245 ; 336963247 ; 84483248 ; 54773248 ; 79873248 ; 91863248 ; 435383249 ; 187473250 ; 152903253 ; 429583254 ; 141173255 ; 296243256 ; 59233257 ; 44783259 ; 163593259 ; 32603262 ; 365633262 ; 53063263 ; 67973264 ; 103483264 ; 33693265 ; 265003269 ; 47463270 ; 412963270 ; 434163271 ; 198593272 ; 50203272 ; 37263272 ; 78683273 ; 377423273 ; 419503275 ; 266393276 ; 38713278 ; 390593278 ; 380953282 ; 249183282 ; 78403283 ; 163943287 ; 142793290 ; 434063291 ; 43803292 ; 289593293 ; 121843293 ; 197623295 ; 99663299 ; 246643300 ; 71813300 ; 395193303 ; 123723303 ; 40213303 ; 131813303 ; 66123303 ; 336413303 ; 350883303 ; 391993303 ; 400663304 ; 50683304 ; 347623305 ; 57053305 ; 358433306 ; 49663306 ; 352143306 ; 369463308 ; 33093311 ; 377173312 ; 146873315 ; 57843315 ; 53673315 ; 78593315 ; 89693317 ; 47413319 ; 363473320 ; 50303320 ; 380933323 ; 276913325 ; 123913327 ; 61583327 ; 334963328 ; 36333328 ; 221403331 ; 112553332 ; 51563332 ; 229353334 ; 85313334 ; 41113336 ; 179763336 ; 97013336 ; 97063339 ; 50993339 ; 190783339 ; 315313339 ; 435583340 ; 38563340 ; 78103341 ; 244223343 ; 48163343 ; 48633348 ; 293513349 ; 41723349 ; 143423349 ; 150173349 ; 100903351 ; 111433354 ; 354323354 ; 368513356 ; 52373356 ; 402683357 ; 75713357 ; 134243357 ; 106183357 ; 263433357 ; 72793357 ; 167023357 ; 439113360 ; 379023361 ; 55343361 ; 65713361 ; 143013361 ; 245563361 ; 249003362 ; 56523363 ; 41953363 ; 248943363 ; 137723364 ; 439913364 ; 202873366 ; 377973369 ; 360583369 ; 160823370 ; 342203376 ; 333123378 ; 337653379 ; 332413380 ; 173883381 ; 204833382 ; 38553387 ; 142933387 ; 38323388 ; 169203389 ; 334973389 ; 377693403 ; 43243408 ; 44393409 ; 257913412 ; 41983412 ; 266283412 ; 55793415 ; 411933416 ; 329313417 ; 34823418 ; 441003419 ; 43273422 ; 165853424 ; 267423424 ; 377353425 ; 118763426 ; 342313426 ; 116503427 ; 299913430 ; 71473430 ; 53273430 ; 35763431 ; 35803432 ; 330213433 ; 34923434 ; 263513435 ; 197933435 ; 387613436 ; 236163437 ; 58943440 ; 215033440 ; 427003442 ; 98083445 ; 34453445 ; 141343445 ; 381353445 ; 442103449 ; 95813449 ; 61933449 ; 101733449 ; 266783449 ; 162563449 ; 82323449 ; 402033453 ; 147653460 ; 308773460 ; 41923462 ; 180023465 ; 67463465 ; 191223465 ; 143973465 ; 112023472 ; 85033472 ; 58573474 ; 90313477 ; 288233477 ; 316963478 ; 281423480 ; 58863480 ; 47663480 ; 79033480 ; 88993481 ; 420363481 ; 437633483 ; 60293483 ; 90293483 ; 443163484 ; 394543485 ; 68993486 ; 161493487 ; 48473487 ; 38043487 ; 58963489 ; 48483489 ; 195733489 ; 274483489 ; 86953489 ; 156483492 ; 266973492 ; 253533493 ; 187713493 ; 100023493 ; 160643495 ; 104503496 ; 129083496 ; 35073496 ; 43593496 ; 365933497 ; 35073497 ; 64943497 ; 108113497 ; 111263497 ; 103443497 ; 439003499 ; 333753499 ; 343513499 ; 419903499 ; 419913503 ; 132243503 ; 253263507 ; 85023511 ; 342973511 ; 47123512 ; 197383513 ; 162523516 ; 337173517 ; 431653517 ; 433903517 ; 86063518 ; 412043525 ; 89483525 ; 36723525 ; 109363525 ; 350183525 ; 365573525 ; 168193525 ; 389763526 ; 46353526 ; 39393526 ; 112813526 ; 282173526 ; 382313528 ; 81063528 ; 65173530 ; 295903536 ; 98233547 ; 245553551 ; 357083554 ; 419023555 ; 378093558 ; 70833559 ; 128883559 ; 261513559 ; 103823562 ; 124793563 ; 316793565 ; 199443565 ; 423943567 ; 36703567 ; 433013569 ; 87623570 ; 241033571 ; 45003571 ; 37183571 ; 236853571 ; 326903574 ; 343183574 ; 360243575 ; 220103575 ; 79343575 ; 315163576 ; 45263577 ; 62633583 ; 53103583 ; 50203583 ; 77743583 ; 37263584 ; 68083586 ; 287193588 ; 436863589 ; 217683589 ; 217693589 ; 226343589 ; 89943589 ; 89933591 ; 412123592 ; 208843592 ; 318433594 ; 77073595 ; 294283598 ; 53413599 ; 53413599 ; 124083599 ; 61973599 ; 61983602 ; 52083603 ; 44433603 ; 245593605 ; 255873606 ; 193043606 ; 135183606 ; 65413606 ; 138933606 ; 291093606 ; 158603606 ; 138923606 ; 409853606 ; 415883606 ; 441763608 ; 264393612 ; 68083612 ; 126763612 ; 57963613 ; 53943613 ; 228283614 ; 180053621 ; 358563622 ; 292083622 ; 45833622 ; 306863629 ; 335703630 ; 246893632 ; 440683633 ; 39673637 ; 36563638 ; 48413640 ; 325533641 ; 151663643 ; 44213646 ; 72393646 ; 144373647 ; 75633647 ; 51373647 ; 110173647 ; 289003647 ; 374983647 ; 45683647 ; 75643648 ; 43973648 ; 67973649 ; 105043649 ; 83993651 ; 132263654 ; 203483654 ; 400083655 ; 39723657 ; 40313657 ; 90603657 ; 59423657 ; 109903657 ; 125143658 ; 82033661 ; 172523661 ; 435653662 ; 243503665 ; 434553665 ; 65183666 ; 397103666 ; 435213667 ; 338753667 ; 347153667 ; 102223668 ; 173603669 ; 314253671 ; 38503671 ; 358713671 ; 374663673 ; 207933673 ; 89473673 ; 36743673 ; 319293673 ; 330163673 ; 339243673 ; 349923673 ; 365323673 ; 367093673 ; 168203673 ; 116493674 ; 36753675 ; 65873676 ; 65873676 ; 36773676 ; 207943676 ; 79243677 ; 38533678 ; 55933681 ; 65763681 ; 360113683 ; 218443683 ; 136823686 ; 49503687 ; 334253688 ; 242283688 ; 246403690 ; 255953690 ; 260703691 ; 41763691 ; 273413691 ; 108753698 ; 108753702 ; 408723702 ; 408733702 ; 92393703 ; 308223706 ; 283213707 ; 130533710 ; 176343710 ; 73073710 ; 221963713 ; 275583713 ; 286403717 ; 358473720 ; 242903721 ; 69283721 ; 372273723 ; 182073725 ; 263873726 ; 140393726 ; 273613727 ; 121483729 ; 37303735 ; 331033735 ; 66923737 ; 45763737 ; 364253737 ; 423093738 ; 352063738 ; 316343738 ; 357373739 ; 252423739 ; 254973742 ; 95473742 ; 316743743 ; 47313743 ; 305043743 ; 83293743 ; 276083743 ; 279973743 ; 87753744 ; 114183747 ; 123303747 ; 432583747 ; 435103748 ; 244493749 ; 57843752 ; 45203757 ; 45423760 ; 39263760 ; 146893763 ; 47783767 ; 45893776 ; 295463779 ; 178483779 ; 48043779 ; 63893779 ; 159673788 ; 235413789 ; 54093790 ; 54103790 ; 86643790 ; 45713793 ; 194853794 ; 200223794 ; 86523794 ; 422173796 ; 51303798 ; 250813801 ; 372263804 ; 58963804 ; 129833805 ; 289853808 ; 67713808 ; 391263810 ; 290013812 ; 54733812 ; 298173816 ; 147893818 ; 59193818 ; 130163818 ; 69443818 ; 59173818 ; 83113821 ; 85243821 ; 254983821 ; 60283822 ; 43313823 ; 226043823 ; 42203825 ; 98923825 ; 234183826 ; 154883827 ; 257513828 ; 250543829 ; 170233829 ; 368733830 ; 376813830 ; 69913830 ; 411953831 ; 366613832 ; 142933832 ; 245823834 ; 41283834 ; 277493836 ; 173313838 ; 342963839 ; 52933840 ; 101503840 ; 391093842 ; 86153842 ; 52633842 ; 367643844 ; 264383844 ; 333213844 ; 340433844 ; 54293845 ; 41993846 ; 265773846 ; 45233848 ; 351603850 ; 429243851 ; 51013853 ; 39383853 ; 349933854 ; 41473855 ; 388213855 ; 55123856 ; 43343858 ; 257523859 ; 218973859 ; 108023866 ; 427173867 ; 77953874 ; 141803883 ; 251053885 ; 55683892 ; 41803896 ; 246003897 ; 250873921 ; 359993927 ; 40163927 ; 252123935 ; 55053936 ; 251333936 ; 150073937 ; 294713938 ; 259383939 ; 242503939 ; 55203939 ; 144063941 ; 42443942 ; 369703943 ; 376433944 ; 353713948 ; 159993953 ; 287173954 ; 296543954 ; 56363954 ; 72023954 ; 283043954 ; 60563954 ; 156323954 ; 405643954 ; 432513955 ; 295023959 ; 90003959 ; 49543959 ; 386523962 ; 60873964 ; 66723964 ; 340053964 ; 360913964 ; 73013964 ; 91273966 ; 299033968 ; 44033970 ; 42693970 ; 416933971 ; 103433972 ; 71763974 ; 333483974 ; 431903978 ; 111863978 ; 158783982 ; 145053985 ; 42983985 ; 372703985 ; 408133988 ; 59253992 ; 41373992 ; 39933995 ; 53053995 ; 304663996 ; 233783996 ; 411483997 ; 425324002 ; 343964005 ; 382824005 ; 430764007 ; 289814007 ; 158184008 ; 44684015 ; 79464019 ; 40494022 ; 296594023 ; 181764023 ; 193224024 ; 73384024 ; 83874028 ; 319884029 ; 77544029 ; 77694031 ; 41054040 ; 75094041 ; 50594043 ; 79854044 ; 96684045 ; 106074045 ; 49724046 ; 41574051 ; 282724052 ; 225754053 ; 68034053 ; 193924053 ; 425254055 ; 125784059 ; 53844059 ; 53854059 ; 106314059 ; 125884059 ; 131024059 ; 43264060 ; 184594060 ; 112144060 ; 109744060 ; 178514060 ; 48054060 ; 101064060 ; 122024060 ; 43534064 ; 165294064 ; 109484064 ; 370684065 ; 283144068 ; 409744069 ; 206044069 ; 335324071 ; 74564072 ; 256614073 ; 44054074 ; 197444075 ; 273154076 ; 54244076 ; 153574076 ; 71734076 ; 114284078 ; 83524081 ; 304974083 ; 87714088 ; 58794089 ; 40894091 ; 52474091 ; 159774091 ; 48314092 ; 40934092 ; 211384092 ; 424024095 ; 114114096 ; 135414099 ; 86314099 ; 395384101 ; 128724102 ; 231854103 ; 159004104 ; 78564105 ; 125144105 ; 441294106 ; 130164106 ; 69444106 ; 59174106 ; 333324106 ; 83114107 ; 334564113 ; 375364119 ; 351814126 ; 197264126 ; 386774126 ; 86114127 ; 438694129 ; 424234129 ; 69894129 ; 346324129 ; 166224129 ; 84974129 ; 134284141 ; 198284141 ; 91014143 ; 204164143 ; 292214143 ; 111644146 ; 364624146 ; 367144148 ; 146774148 ; 41504148 ; 424304150 ; 174894150 ; 202464150 ; 424294152 ; 46454155 ; 51354155 ; 77304155 ; 66424155 ; 70814158 ; 241314162 ; 44994162 ; 340194162 ; 379604163 ; 146624172 ; 48304174 ; 296224181 ; 142124181 ; 101624185 ; 146214186 ; 300884198 ; 251524200 ; 52384202 ; 109224205 ; 306974210 ; 266504210 ; 282824210 ; 305384215 ; 111584216 ; 296344217 ; 42184221 ; 307484223 ; 304794226 ; 42274227 ; 42284229 ; 294164238 ; 301064244 ; 293064250 ; 296544250 ; 72024250 ; 60564254 ; 79934254 ; 308804254 ; 312664254 ; 355084256 ; 83814262 ; 378374262 ; 332364264 ; 74124264 ; 44104267 ; 117014267 ; 44904270 ; 405934273 ; 44494273 ; 158674274 ; 162734279 ; 335904282 ; 52214286 ; 93744286 ; 114594289 ; 99674290 ; 290704290 ; 409484291 ; 294914293 ; 64824293 ; 59524293 ; 52434293 ; 72834295 ; 285004296 ; 318144298 ; 366484301 ; 43014301 ; 138804301 ; 87754301 ; 47314302 ; 407874308 ; 50474310 ; 96484310 ; 82574310 ; 90014310 ; 78434310 ; 79824310 ; 338044310 ; 165124310 ; 362274310 ; 403184311 ; 48744313 ; 112404314 ; 51564318 ; 45504318 ; 245754318 ; 245764320 ; 103504321 ; 196334321 ; 45984321 ; 286424322 ; 54774322 ; 70304322 ; 74134322 ; 74854322 ; 129924322 ; 129934322 ; 153854322 ; 160234322 ; 368094326 ; 48004329 ; 60244330 ; 88524332 ; 88704333 ; 260094333 ; 394744333 ; 409054334 ; 65164334 ; 85854340 ; 285514342 ; 256224343 ; 248114347 ; 307544350 ; 111534350 ; 63704351 ; 49574351 ; 412114353 ; 48044353 ; 160874355 ; 435934355 ; 69194356 ; 187604356 ; 51304356 ; 287644356 ; 361604357 ; 159644357 ; 116564357 ; 75644359 ; 99854359 ; 99844362 ; 343294362 ; 416254362 ; 420414363 ; 100794364 ; 63774364 ; 66284364 ; 158914364 ; 159784369 ; 47634369 ; 422564369 ; 112344373 ; 425344374 ; 47604383 ; 59524383 ; 55944384 ; 207504392 ; 89484392 ; 46354392 ; 350184392 ; 365574392 ; 389764392 ; 112814393 ; 338964395 ; 66094400 ; 211464400 ; 45054402 ; 83684403 ; 365414405 ; 83354407 ; 410264410 ; 141144414 ; 44154414 ; 253024417 ; 238424428 ; 235964434 ; 235634445 ; 252094451 ; 293554456 ; 383694456 ; 388614456 ; 431534458 ; 61714466 ; 291994472 ; 57434472 ; 79774473 ; 91394473 ; 56244473 ; 296614475 ; 52614475 ; 111894476 ; 60404483 ; 382154483 ; 419984483 ; 116204484 ; 343154485 ; 330344485 ; 162514485 ; 162934485 ; 175694486 ; 44884486 ; 81384488 ; 108284489 ; 59104490 ; 76494491 ; 47214491 ; 59484491 ; 295604493 ; 48684493 ; 73154494 ; 52084494 ; 55374497 ; 76634497 ; 45994497 ; 367694497 ; 383184497 ; 387314504 ; 45054504 ; 252204504 ; 79064507 ; 422754510 ; 55984510 ; 72324512 ; 121054512 ; 346714514 ; 67554514 ; 157554515 ; 92344518 ; 420334523 ; 83404523 ; 47864523 ; 392534525 ; 349674525 ; 122714526 ; 53274527 ; 152294530 ; 255374534 ; 177384535 ; 58504535 ; 323364535 ; 110054535 ; 344964536 ; 162174536 ; 431034537 ; 290094538 ; 50114538 ; 95064539 ; 181574539 ; 193274541 ; 230844544 ; 294474548 ; 421984551 ; 314024555 ; 217844555 ; 372104556 ; 52464557 ; 373254557 ; 342664557 ; 47284559 ; 47974559 ; 269564559 ; 181494571 ; 203694572 ; 175794574 ; 48214578 ; 223724581 ; 236194581 ; 237214583 ; 50174583 ; 50184586 ; 68934586 ; 68944589 ; 71364591 ; 45924591 ; 161504592 ; 54444593 ; 84264593 ; 76444593 ; 48444593 ; 214084593 ; 413084598 ; 364224598 ; 108204598 ; 348614599 ; 70254599 ; 76854599 ; 432764599 ; 432774600 ; 128504600 ; 77594602 ; 128944602 ; 51434603 ; 264644603 ; 397414603 ; 410454603 ; 410394606 ; 62114606 ; 291104606 ; 95154606 ; 135194609 ; 58904614 ; 389844614 ; 377934621 ; 343664621 ; 48734621 ; 394104621 ; 406264621 ; 437604623 ; 125644624 ; 207604626 ; 332614626 ; 341674626 ; 422914628 ; 113384628 ; 344714629 ; 81524630 ; 390874633 ; 428634635 ; 109364640 ; 46854641 ; 373704644 ; 296974645 ; 211754652 ; 88314652 ; 107814653 ; 141794657 ; 88754658 ; 50494659 ; 249774662 ; 78764669 ; 236284671 ; 250794672 ; 236274672 ; 240204672 ; 46734673 ; 240204677 ; 439834679 ; 89764679 ; 254414681 ; 254044684 ; 103414686 ; 52294686 ; 244944686 ; 248644686 ; 107294687 ; 236754692 ; 78654693 ; 338174711 ; 343824712 ; 72714717 ; 65364717 ; 360414720 ; 240604721 ; 52854728 ; 343484728 ; 73324728 ; 210324728 ; 103104728 ; 416194731 ; 83894732 ; 48224732 ; 58484733 ; 119814733 ; 58054735 ; 49274740 ; 284864740 ; 111954741 ; 359744743 ; 367534746 ; 323624749 ; 50334749 ; 50324749 ; 84284749 ; 82204755 ; 123104756 ; 356914756 ; 341214756 ; 384464758 ; 415264760 ; 79844761 ; 48184765 ; 82424766 ; 63704766 ; 125044769 ; 146974773 ; 133824773 ; 226174776 ; 96584776 ; 50544776 ; 181834776 ; 82724776 ; 125464776 ; 111394776 ; 157264778 ; 271664785 ; 382364788 ; 70254788 ; 65014788 ; 76854788 ; 86584793 ; 312254793 ; 49894793 ; 51974794 ; 329964794 ; 410174796 ; 47984797 ; 53844797 ; 106314798 ; 47994799 ; 48004805 ; 184594805 ; 178514811 ; 429964813 ; 192574813 ; 85124814 ; 62714814 ; 78104814 ; 439814815 ; 137304815 ; 422984821 ; 95594824 ; 60404826 ; 61454826 ; 129804829 ; 54264830 ; 48314838 ; 127214838 ; 98144838 ; 194914838 ; 75774838 ; 146104838 ; 157834839 ; 110184839 ; 328504839 ; 115784840 ; 78324840 ; 285854842 ; 51514842 ; 91634844 ; 112834845 ; 109224845 ; 128274845 ; 368914846 ; 63804848 ; 271764855 ; 51444855 ; 54574857 ; 259164860 ; 405034861 ; 352954865 ; 201354865 ; 113314865 ; 159844867 ; 102194874 ; 225724877 ; 439734877 ; 136714878 ; 51424883 ; 389434884 ; 240984885 ; 367384887 ; 53094887 ; 51914891 ; 50244892 ; 259954893 ; 55234894 ; 72414896 ; 355394900 ; 55544900 ; 148214901 ; 49024904 ; 148304907 ; 107824913 ; 254074913 ; 141234929 ; 163194942 ; 142404954 ; 90704954 ; 138614954 ; 66254954 ; 86774954 ; 384884957 ; 261894957 ; 296204957 ; 171094957 ; 380344960 ; 410414965 ; 420014966 ; 436844967 ; 328204968 ; 56414968 ; 136774992 ; 118204993 ; 131384993 ; 85464993 ; 85494993 ; 85474994 ; 52904994 ; 75494998 ; 119064999 ; 119095000 ; 52965001 ; 52965003 ; 56935004 ; 56935005 ; 57205005 ; 175855007 ; 53315007 ; 364585012 ; 392315013 ; 53085017 ; 121325017 ; 185735018 ; 60635018 ; 287895018 ; 313285020 ; 72185021 ; 95315024 ; 352825030 ; 146175030 ; 71475034 ; 315835035 ; 373395035 ; 123065038 ; 230475038 ; 68555038 ; 128065044 ; 54235047 ; 67575050 ; 263445051 ; 57475051 ; 263285051 ; 82245054 ; 50725054 ; 57905054 ; 319955054 ; 319965054 ; 179435055 ; 82735060 ; 62155062 ; 53635062 ; 129985062 ; 427325064 ; 83725064 ; 99285065 ; 253255066 ; 377625067 ; 51575067 ; 75855068 ; 391825068 ; 373945072 ; 74805074 ; 64115075 ; 51975081 ; 241305081 ; 253565083 ; 90025085 ; 282435085 ; 56445088 ; 68145088 ; 85175090 ; 192575096 ; 354355099 ; 304865103 ; 54075107 ; 262795111 ; 79265111 ; 113335114 ; 79975119 ; 56135127 ; 152445127 ; 86725130 ; 59075130 ; 187605130 ; 287655133 ; 221695134 ; 92045136 ; 192445144 ; 187385144 ; 216115144 ; 143315144 ; 245785144 ; 63225144 ; 251605144 ; 278435144 ; 322835144 ; 129025147 ; 59075147 ; 100055147 ; 128655151 ; 101085152 ; 104395155 ; 347715156 ; 132865157 ; 73845157 ; 370165159 ; 162225166 ; 51675173 ; 166375174 ; 329135174 ; 172425175 ; 64885175 ; 101745178 ; 204605178 ; 163675178 ; 80435178 ; 413985178 ; 434815178 ; 428995178 ; 437045183 ; 379965187 ; 51885192 ; 65055192 ; 156845192 ; 294485192 ; 311255200 ; 210335202 ; 53735202 ; 246145202 ; 77685203 ; 71235204 ; 360095207 ; 69295211 ; 52115212 ; 138205212 ; 229455212 ; 72885215 ; 316295216 ; 52185216 ; 55545216 ; 148215224 ; 331745229 ; 237805238 ; 236705239 ; 246965242 ; 71335243 ; 424315245 ; 80135245 ; 287225246 ; 52505248 ; 266025278 ; 102925278 ; 138295287 ; 52885287 ; 53045287 ; 208355289 ; 321715302 ; 63075305 ; 78565323 ; 404765331 ; 168885371 ; 57905371 ; 319955373 ; 68065386 ; 53875387 ; 53885388 ; 53895410 ; 54115425 ; 254685431 ; 241035445 ; 104145450 ; 403375458 ; 161185460 ; 69435469 ; 85465496 ; 202315498 ; 296105503 ; 66805503 ; 159375503 ; 154285503 ; 338275503 ; 110035504 ; 66805504 ; 420555520 ; 242505521 ; 168205531 ; 208225534 ; 143015534 ; 245565534 ; 249005544 ; 59615554 ; 256525598 ; 156525611 ; 437395616 ; 289085616 ; 304085631 ; 288485636 ; 296555636 ; 79995636 ; 288255646 ; 91745648 ; 385415651 ; 418975690 ; 68895711 ; 427105712 ; 63405715 ; 291325724 ; 96605727 ; 133635727 ; 101075744 ; 57455744 ; 189695800 ; 65395808 ; 129725809 ; 125975848 ; 58495858 ; 390085858 ; 71865865 ; 349995886 ; 79035890 ; 150695900 ; 63865907 ; 128645915 ; 202925915 ; 112265915 ; 312935915 ; 439785925 ; 132165925 ; 348655929 ; 434695931 ; 59325946 ; 64745951 ; 199525951 ; 405915966 ; 135695966 ; 404965996 ; 306816015 ; 65766028 ; 65976050 ; 389246088 ; 67636091 ; 112236106 ; 82186118 ; 405846132 ; 391296151 ; 171166158 ; 61986163 ; 417716168 ; 280516189 ; 70856224 ; 225166295 ; 186296349 ; 63506371 ; 268776380 ; 99066386 ; 99616386 ; 195226395 ; 226256402 ; 128266404 ; 103206405 ; 129036405 ; 393546413 ; 433026434 ; 192286455 ; 66626467 ; 154216470 ; 64716471 ; 64726474 ; 343826494 ; 76766499 ; 107226511 ; 374466518 ; 438006522 ; 221636539 ; 260186581 ; 269046601 ; 164806615 ; 270416631 ; 296936640 ; 284916640 ; 285536640 ; 91826655 ; 171896655 ; 393256656 ; 438006669 ; 66706670 ; 162736672 ; 91276677 ; 88916679 ; 80806680 ; 160186680 ; 86656680 ; 442086704 ; 99246712 ; 68216712 ; 129626714 ; 99126746 ; 415846752 ; 321676754 ; 69916760 ; 79916763 ; 411396764 ; 177126784 ; 293356811 ; 68126811 ; 129726842 ; 276246871 ; 218976961 ; 101076961 ; 244646973 ; 155477010 ; 163677010 ; 401867030 ; 338287030 ; 376187030 ; 135637030 ; 435387033 ; 217277052 ; 90547052 ; 395677068 ; 138177103 ; 89767147 ; 380947186 ; 172017195 ; 71967195 ; 305887195 ; 305897196 ; 303117196 ; 305897196 ; 160127197 ; 398467197 ; 110117204 ; 72087204 ; 91867288 ; 80807315 ; 81937315 ; 165677317 ; 73597339 ; 85037345 ; 303127366 ; 94587366 ; 213657366 ; 139007377 ; 124117386 ; 106967413 ; 181277467 ; 212247480 ; 277667485 ; 181277503 ; 259117503 ; 89727503 ; 141237577 ; 194927577 ; 91197578 ; 100077586 ; 76327597 ; 195627632 ; 357237648 ; 199397650 ; 382847676 ; 200177692 ; 82327726 ; 225047756 ; 381167756 ; 389707780 ; 90757809 ; 254207812 ; 106087843 ; 299697850 ; 143037880 ; 372897899 ; 254607899 ; 96387926 ; 92977926 ; 381567926 ; 381577987 ; 91867991 ; 286008005 ; 158078005 ; 313418013 ; 312778017 ; 282618017 ; 92068065 ; 343418091 ; 376188107 ; 363858130 ; 147088130 ; 85698145 ; 123178145 ; 123188152 ; 260848203 ; 354018209 ; 154378240 ; 96278273 ; 179448335 ; 110548337 ; 83918394 ; 89508448 ; 192288449 ; 185338534 ; 311868547 ; 338528631 ; 88148657 ; 330168664 ; 106118665 ; 202718665 ; 154288665 ; 435358665 ; 108138669 ; 204388695 ; 136268702 ; 224808703 ; 215438714 ; 211158727 ; 134298744 ; 137468771 ; 245328775 ; 305048775 ; 276088800 ; 88018801 ; 88028811 ; 93378830 ; 91748850 ; 244408850 ; 254558899 ; 240838969 ; 111828976 ; 107348999 ; 134809035 ; 104939037 ; 91189045 ; 313599045 ; 111559062 ; 419839070 ; 172699070 ; 398099078 ; 165659078 ; 360409101 ; 282229111 ; 159709111 ; 311779112 ; 311779219 ; 413069224 ; 358069238 ; 353569238 ; 373099245 ; 327939295 ; 92969296 ; 434329297 ; 113329299 ; 378079299 ; 396649318 ; 94829320 ; 437499361 ; 369119374 ; 364959458 ; 212329458 ; 137529476 ; 351759481 ; 118739499 ; 154459520 ; 210509611 ; 96129638 ; 107229657 ; 98089737 ; 135799737 ; 134369743 ; 103099787 ; 157219823 ; 238749823 ; 249269836 ; 143169952 ; 190269954 ; 99559966 ; 167299978 ; 135419979 ; 189029979 ; 1890310007 ; 1903910048 ; 1004910049 ; 1005010050 ; 1005110072 ; 1949210072 ; 1949310087 ; 2704610090 ; 1325310116 ; 2056610124 ; 1439910199 ; 2866110205 ; 2049710228 ; 2985210273 ; 2145010292 ; 1382910298 ; 2103410320 ; 2258110356 ; 1374710372 ; 2199710528 ; 2355910631 ; 2695610722 ; 2546010726 ; 1466610726 ; 2544210734 ; 2544110811 ; 2569010811 ; 1470810876 ; 1506610884 ; 2584810914 ; 1091510936 ; 1681910985 ; 2652211002 ; 3979811011 ; 3984711022 ; 4396911046 ; 2799011103 ; 2925411153 ; 1115411154 ; 1115511156 ; 2915311156 ; 3135811202 ; 2960111214 ; 4169511313 ; 3528211328 ; 3400911331 ; 1133211331 ; 3815611332 ; 1598411333 ; 3572511334 ; 3925811348 ; 3903811368 ; 3466811369 ; 3900711438 ; 3569611438 ; 3721911459 ; 3584511460 ; 1653611502 ; 3610711532 ; 1726611538 ; 3697011552 ; 1166111599 ; 4160011666 ; 4193311688 ; 1168911690 ; 4203111796 ; 1293812098 ; 1209912099 ; 1210012132 ; 1213312153 ; 1761512153 ; 1771612214 ; 1221512270 ; 4050912310 ; 1231112353 ; 1311912407 ; 1357912430 ; 3438012430 ; 4197012559 ; 1799912569 ; 1700112587 ; 1310312587 ; 1310412630 ; 1317512686 ; 1271612721 ; 1806012903 ; 3021112990 ; 1896912990 ; 2966112992 ; 1602313102 ; 1310313104 ; 1310513105 ; 1959613146 ; 1314713362 ; 1336313412 ; 2710413412 ; 4222613471 ; 1624013481 ; 2672013516 ; 2001713544 ; 1440713547 ; 4285513553 ; 1355413573 ; 3743613605 ; 4296513648 ; 3551913671 ; 1367213705 ; 2138813772 ; 1391413810 ; 2963413819 ; 1382013870 ; 2306213900 ; 1390113924 ; 1392513963 ; 2262413986 ; 1398714019 ; 2265214021 ; 2267714086 ; 2306214138 ; 3933314166 ; 3356114194 ; 1443714212 ; 2362814221 ; 1422214226 ; 2825814240 ; 2355314246 ; 2398114353 ; 1435414425 ; 1461014446 ; 2406114558 ; 1455914634 ; 3186914763 ; 1476414829 ; 1483014876 ; 1487715343 ; 1534415403 ; 2619115404 ; 1540515428 ; 1601815477 ; 3604015477 ; 1662815518 ; 4180515546 ; 3484515547 ; 1554815582 ; 2829815713 ; 2937815807 ; 3134115886 ; 1588715927 ; 1592715947 ; 2981715972 ; 1597315973 ; 2999316012 ; 1601316013 ; 3059016013 ; 3102916017 ; 3382616064 ; 3848316094 ; 3238316094 ; 3984516205 ; 3709316205 ; 3709416253 ; 3425016347 ; 3917016358 ; 3913516510 ; 1651116511 ; 1651216622 ; 3727416628 ; 3604116639 ; 3581016697 ; 3987516986 ; 3694917006 ; 3699617165 ; 3773517199 ; 3850217199 ; 3850317236 ; 1723717251 ; 4147617261 ; 3890117265 ; 3987617265 ; 3987717398 ; 4309017436 ; 4237417492 ; 4353617512 ; 1751317687 ; 1768817943 ; 1794418001 ; 1800218183 ; 1818418184 ; 1818518185 ; 1818618186 ; 1818718419 ; 1842018987 ; 1898819026 ; 1902719028 ; 1902919029 ; 1903019030 ; 1903119485 ; 1948619493 ; 1949420135 ; 2013620209 ; 2021020288 ; 2028920491 ; 2049220492 ; 2049320493 ; 2049420494 ; 2049520495 ; 2049620496 ; 2049720594 ; 2059520595 ; 2059620709 ; 2071020718 ; 2071920962 ; 2096321035 ; 2103621047 ; 2104821048 ; 2104921175 ; 2117621287 ; 2128821429 ; 2143021543 ; 2154421799 ; 2180021994 ; 2199521995 ; 2199621997 ; 2199822005 ; 2200622006 ; 2200722074 ; 2207522084 ; 2208522169 ; 2217022184 ; 2218522375 ; 2237622427 ; 2242822428 ; 2242922480 ; 2248122592 ; 2259322602 ; 2260322639 ; 2264022928 ; 2292923026 ; 2302723046 ; 2304723095 ; 2309623212 ; 2321323289 ; 2329023381 ; 2338223504 ; 2350523587 ; 2358823679 ; 2368023684 ; 2368524689 ; 2469024864 ; 2486525188 ; 2518925189 ; 2519025228 ; 2522925342 ; 2534325415 ; 2541625574 ; 2557525605 ; 2560625659 ; 2566025690 ; 2569125752 ; 2575325896 ; 2589726009 ; 2601026010 ; 2601126011 ; 2601227124 ; 2712527750 ; 2775127765 ; 2776627866 ; 2786727867 ; 2786827989 ; 2799028005 ; 2800628014 ; 2801528015 ; 2801628019 ; 2802028036 ; 2803728038 ; 2803928304 ; 2830528344 ; 2834528374 ; 2837528678 ; 2867928753 ; 2875428829 ; 2883028830 ; 2883128870 ; 2887129305 ; 2930629331 ; 2933229375 ; 2937629589 ; 2959029599 ; 2960029600 ; 2960129608 ; 2960929659 ; 2966029968 ; 2996930014 ; 3001530028 ; 3002930312 ; 3031330415 ; 3041630475 ; 3047630590 ; 3059130725 ; 3072630740 ; 3074130767 ; 3076831028 ; 3102931219 ; 3122031251 ; 3125231263 ; 3126431267 ; 3126831293 ; 3129431329 ; 3133031358 ; 3135931494 ; 3149531624 ; 3162531664 ; 3166531929 ; 3193031966 ; 3196732362 ; 3236332381 ; 3238232382 ; 3238332803 ; 3280432933 ; 3293432983 ; 3298432988 ; 3298932996 ; 3299733047 ; 3304833120 ; 3312133232 ; 3323333432 ; 3343333460 ; 3346133532 ; 3353334027 ; 3402834384 ; 3438534666 ; 3466734667 ; 3466834784 ; 3478534790 ; 3479134906 ; 3490735064 ; 3506535105 ; 3510635107 ; 3510835108 ; 3510935331 ; 3533235344 ; 3534535435 ; 3543635630 ; 3563135643 ; 3564435699 ; 3570035759 ; 3576035867 ; 3586835870 ; 3587135959 ; 3596036005 ; 3600636010 ; 3601136080 ; 3608136247 ; 3624836312 ; 3631336627 ; 3662836635 ; 3663636746 ; 3674736995 ; 3699637092 ; 3709337102 ; 3710337210 ; 3721137466 ; 3746737579 ; 3758038227 ; 3822838407 ; 3840838431 ; 3843238702 ; 3870338744 ; 3874538745 ; 3874638746 ; 3874738751 ; 3875238818 ; 3881938944 ; 3894539072 ; 3907339108 ; 3910939284 ; 3928539397 ; 3939839404 ; 3940539789 ; 3979039790 ; 3979139845 ; 3984639875 ; 3987639907 ; 3990839990 ; 3999140221 ; 4022240564 ; 4056540565 ; 4056640623 ; 4062440794 ; 4079541025 ; 4102641299 ; 4130041380 ; 4138141583 ; 4158441619 ; 4162042275 ; 4227642329 ; 4233042599 ; 4260042615 ; 4261642736 ; 4273742781 ; 4278242882 ; 4288342946 ; 4294743082 ; 4308343086 ; 4308743157 ; 4315843293 ; 4329443324 ; 4332543440 ; 4344143479 ; 4348043482 ; 4348343558 ; 4355943585 ; 4358643654 ; 4365543791 ; 4379243851 ; 4385243852 ; 4385343990 ; 4399144056 ; 4405744208 ; 44209
