# Supplementary material for: The Exploration of Novel Regulatory Relationships Drives Haloarchaeal Operon-Like Structural Dynamics over Short Evolutionary Distances
Source: Microorganisms. 2020 Nov 30;8(12):1900. doi: 10.3390/microorganisms8121900 (PMC7760734; doi:10.3390/microorganisms8121900)
Supplement: Supplementary file 1 [file microorganisms-08-01900-s001.zip › S7_OperonModificationExamples.rtf]

#cluster	Modification_type278	Prepend287	Prepend290	Append293	Prepend298	Append299	Prepend300	Append304	Prepend310	Append316	Insertion320	Append327	Prepend335	Prepend336	Prepend348	Prepend349	Prepend350	Prepend352	Prepend361	Append362	Append374	Insertion380	Append384	Append386	Prepend389	Insertion393	Append394	Append402	Append404	Append415	Prepend416	Append423	Append429	Append431	Append435	Prepend436	Insertion437	Prepend439	Prepend441	Insertion445	Append449	Append451	Append454	Prepend461	Prepend463	Append465	Append466	Insertion469	Insertion472	Append488	Append490	Prepend493	Prepend496	Prepend500	Insertion507	Append512	Append517	Append521	Prepend522	Append524	Append528	Insertion529	Append530	Append536	Append541	Append543	Prepend550	Insertion556	Prepend560	Prepend565	Prepend566	Append569	Prepend571	Prepend572	Append577	Append578	Append581	Append582	Append585	Prepend586	Append588	Append592	Append596	Prepend600	Prepend601	Append605	Prepend606	Append612	Append613	Append617	Prepend619	Insertion623	Append633	Append655	Append659	Prepend660	Prepend662	Prepend664	Prepend672	Append674	Append676	Insertion679	Append681	Prepend682	Append686	Prepend689	Append698	Prepend703	Prepend714	Append715	Insertion721	Prepend728	Prepend730	Append731	Append733	Prepend735	Prepend737	Prepend741	Append742	Insertion745	Append748	Prepend750	Prepend752	Prepend754	Insertion755	Prepend762	Prepend773	Append774	Append775	Append776	Prepend792	Prepend800	Prepend807	Prepend808	Append810	Append811	Prepend815	Append838	Insertion842	Prepend845	Prepend873	Prepend874	Prepend875	Prepend877	Append880	Prepend881	Append893	Prepend900	Prepend904	Prepend905	Append908	Append912	Prepend915	Prepend919	Prepend921	Append928	Insertion932	Prepend936	Prepend937	Append941	Append942	Prepend943	Prepend944	Prepend954	Append955	Append956	Insertion957	Insertion960	Insertion961	Insertion965	Prepend981	Prepend982	Insertion991	Prepend994	Prepend997	Prepend998	Prepend1001	Prepend1012	Prepend1013	Prepend1016	Prepend1019	Append1020	Append1023	Insertion1032	Prepend1037	Append1042	Append1047	Append1056	Insertion1057	Append1060	Append1076	Prepend1078	Append1079	Prepend1081	Append1082	Prepend1087	Insertion1091	Insertion1094	Prepend1096	Insertion1097	Append1101	Append1104	Prepend1105	Append1106	Append1109	Prepend1111	Insertion1119	Prepend1120	Prepend1128	Prepend1129	Append1137	Prepend1138	Prepend1143	Append1151	Append1158	Prepend1159	Insertion1160	Prepend1163	Prepend1172	Append1174	Append1175	Prepend1180	Prepend1182	Append1184	Prepend1185	Prepend1188	Append1198	Append1199	Prepend1200	Append1206	Append1217	Prepend1218	Append1224	Prepend1228	Append1234	Prepend1240	Prepend1244	Insertion1252	Prepend1261	Append1266	Append1268	Prepend1269	Insertion1270	Prepend1275	Prepend1277	Prepend1284	Prepend1290	Prepend1295	Append1298	Prepend1300	Prepend1309	Prepend1311	Append1312	Prepend1316	Insertion1320	Insertion1324	Append1331	Insertion1338	Append1348	Prepend1358	Append1360	Append1368	Append1376	Append1378	Append1392	Prepend1393	Prepend1396	Prepend1400	Prepend1402	Prepend1404	Append1418	Prepend1419	Append1422	Append1430	Append1433	Insertion1436	Insertion1439	Append1441	Prepend1448	Append1453	Prepend1454	Append1457	Append1458	Prepend1459	Append1461	Prepend1465	Prepend1467	Append1468	Append1471	Append1472	Prepend1475	Prepend1485	Append1488	Append1491	Append1492	Prepend1495	Prepend1497	Prepend1505	Append1506	Append1507	Append1514	Prepend1530	Prepend1540	Prepend1545	Append1547	Append1560	Prepend1561	Append1563	Prepend1564	Prepend1570	Append1577	Append1578	Append1582	Prepend1593	Append1594	Prepend1598	Insertion1604	Append1607	Prepend1608	Insertion1611	Prepend1612	Prepend1613	Append1614	Insertion1621	Insertion1622	Insertion1623	Append1630	Prepend1637	Prepend1639	Append1640	Prepend1647	Append1648	Insertion1656	Prepend1662	Prepend1663	Prepend1665	Append1671	Append1675	Prepend1676	Append1677	Append1680	Prepend1683	Prepend1684	Prepend1691	Prepend1694	Prepend1697	Append1709	Append1721	Prepend1726	Prepend1727	Append1733	Append1734	Prepend1737	Prepend1738	Prepend1739	Prepend1744	Insertion1752	Append1755	Append1756	Insertion1763	Append1765	Prepend1767	Prepend1773	Append1780	Append1787	Prepend1800	Append1802	Prepend1804	Append1807	Prepend1816	Append1817	Append1819	Insertion1820	Append1828	Append1836	Append1843	Append1847	Append1854	Prepend1863	Append1868	Append1870	Append1872	Append1873	Insertion1876	Prepend1880	Append1885	Append1888	Append1896	Append1906	Append1908	Prepend1914	Prepend1916	Append1922	Prepend1930	Append1931	Append1933	Insertion1941	Append1944	Append1945	Append1946	Prepend1961	Append1963	Prepend1965	Prepend1967	Append1972	Insertion1975	Append1976	Append1983	Append1993	Append2000	Append2009	Prepend2017	Prepend2018	Prepend2020	Append2025	Append2027	Prepend2028	Prepend2032	Append2033	Prepend2039	Insertion2051	Append2052	Append2056	Append2057	Append2060	Prepend2061	Prepend2066	Append2069	Prepend2075	Prepend2076	Append2089	Append2090	Append2091	Append2095	Insertion2099	Append2101	Prepend2102	Insertion2105	Prepend2106	Prepend2107	Prepend2117	Append2119	Prepend2122	Insertion2124	Append2126	Append2127	Prepend2130	Prepend2131	Append2133	Append2135	Prepend2141	Append2145	Prepend2146	Append2149	Append2152	Append2157	Append2167	Append2168	Append2173	Prepend2174	Insertion2182	Prepend2183	Prepend2186	Append2187	Append2188	Append2189	Prepend2190	Prepend2193	Prepend2198	Prepend2199	Append2205	Prepend2212	Prepend2214	Prepend2216	Append2227	Append2228	Append2232	Prepend2238	Prepend2246	Append2250	Append2251	Prepend2258	Append2263	Prepend2264	Prepend2265	Append2269	Append2272	Prepend2274	Append2282	Prepend2283	Append2285	Prepend2291	Append2295	Append2297	Prepend2304	Append2306	Prepend2312	Prepend2313	Prepend2322	Append2323	Append2325	Prepend2330	Prepend2331	Prepend2334	Insertion2340	Append2353	Prepend2354	Prepend2362	Append2366	Insertion2370	Append2380	Append2382	Append2383	Prepend2389	Prepend2395	Prepend2399	Append2402	Prepend2406	Append2426	Insertion2427	Append2429	Prepend2439	Prepend2460	Append2462	Append2464	Prepend2465	Prepend2466	Append2469	Append2471	Prepend2475	Prepend2478	Prepend2480	Append2493	Prepend2496	Prepend2498	Prepend2499	Append2518	Prepend2519	Insertion2520	Append2527	Append2529	Append2530	Insertion2535	Append2540	Prepend2552	Append2561	Append2568	Prepend2574	Append2575	Append2577	Append2582	Prepend2584	Prepend2591	Append2597	Append2598	Insertion2602	Prepend2609	Append2610	Append2617	Append2620	Prepend2621	Prepend2630	Prepend2636	Prepend2639	Append2641	Prepend2648	Prepend2663	Prepend2673	Append2679	Insertion2689	Append2693	Prepend2700	Prepend2706	Append2709	Append2712	Insertion2713	Append2715	Append2723	Append2725	Prepend2727	Append2739	Append2748	Insertion2757	Append2758	Append2759	Insertion2771	Prepend2784	Append2789	Append2795	Prepend2797	Append2814	Append2815	Prepend2822	Prepend2827	Prepend2829	Insertion2832	Prepend2837	Insertion2838	Append2843	Prepend2850	Prepend2852	Append2861	Append2862	Append2863	Append2864	Prepend2867	Append2868	Prepend2876	Append2888	Append2893	Insertion2895	Append2896	Append2897	Append2904	Append2911	Append2912	Append2917	Prepend2918	Prepend2935	Prepend2936	Append2940	Prepend2941	Append2942	Prepend2946	Append2949	Prepend2953	Prepend2982	Append2983	Append2985	Prepend2991	Append2994	Prepend2995	Append2996	Append2999	Prepend3006	Prepend3008	Append3015	Append3022	Prepend3023	Prepend3029	Prepend3030	Prepend3031	Append3033	Insertion3035	Prepend3036	Append3040	Append3043	Append3046	Prepend3050	Prepend3051	Prepend3053	Append3055	Append3058	Append3075	Prepend3076	Insertion3087	Prepend3088	Prepend3090	Prepend3106	Prepend3109	Prepend3127	Insertion3128	Prepend3131	Append3138	Prepend3141	Prepend3148	Insertion3151	Append3152	Prepend3156	Append3163	Append3169	Insertion3173	Append3175	Prepend3187	Insertion3203	Prepend3205	Prepend3222	Append3224	Prepend3227	Insertion3253	Append3254	Prepend3263	Append3272	Prepend3273	Prepend3282	Prepend3303	Prepend3304	Prepend3308	Append3309	Append3332	Prepend3335	Append3344	Append3352	Append3364	Prepend3367	Prepend3369	Append3378	Insertion3399	Prepend3401	Prepend3412	Prepend3413	Append3418	Prepend3425	Prepend3431	Append3432	Insertion3438	Append3440	Prepend3452	Prepend3459	Append3462	Prepend3463	Append3472	Append3477	Prepend3487	Append3490	Prepend3492	Prepend3493	Prepend3497	Prepend3501	Prepend3513	Append3526	Append3535	Append3536	Prepend3571	Append3580	Append3581	Append3602	Append3603	Prepend3606	Prepend3612	Prepend3627	Append3631	Append3639	Append3646	Append3647	Append3648	Append3661	Append3663	Append3672	Append3676	Prepend3681	Append3688	Insertion3690	Prepend3713	Prepend3717	Insertion3718	Append3728	Append3739	Append3743	Insertion3746	Prepend3760	Append3770	Append3773	Prepend3778	Prepend3779	Prepend3782	Append3798	Prepend3808	Prepend3821	Prepend3822	Append3836	Append3838	Prepend3840	Append3850	Append3864	Append3870	Prepend3897	Insertion3905	Prepend3927	Prepend3933	Prepend3938	Prepend3939	Insertion3954	Append3962	Prepend3964	Insertion3977	Prepend3988	Prepend3995	Prepend4007	Prepend4008	Prepend4010	Prepend4016	Prepend4019	Prepend4021	Prepend4023	Prepend4037	Prepend4039	Append4045	Prepend4049	Prepend4051	Append4053	Prepend4059	Prepend4073	Prepend4076	Append4081	Insertion4092	Prepend4093	Prepend4105	Prepend4122	Prepend4128	Prepend4129	Prepend4132	Prepend4137	Append4155	Append4167	Prepend4195	Prepend4198	Prepend4200	Prepend4204	Append4217	Append4221	Append4227	Append4228	Append4234	Prepend4248	Prepend4250	Append4266	Append4267	Append4268	Append4273	Prepend4277	Prepend4286	Append4303	Append4318	Prepend4321	Append4322	Prepend4328	Append4331	Append4334	Append4342	Prepend4344	Prepend4350	Prepend4352	Append4359	Prepend4365	Append4368	Append4369	Prepend4400	Prepend4443	Prepend4447	Prepend4449	Prepend4458	Append4472	Append4473	Append4476	Prepend4487	Prepend4488	Prepend4490	Append4494	Append4500	Append4516	Prepend4524	Append4525	Append4527	Append4531	Append4534	Prepend4535	Prepend4550	Prepend4559	Prepend4571	Append4576	Prepend4583	Prepend4586	Append4590	Prepend4598	Append4603	Append4614	Insertion4619	Prepend4644	Insertion4650	Prepend4672	Insertion4673	Insertion4684	Prepend4686	Prepend4701	Prepend4724	Append4732	Append4744	Append4746	Append4747	Prepend4750	Append4754	Prepend4756	Prepend4759	Append4763	Prepend4765	Prepend4766	Append4788	Prepend4797	Prepend4798	Append4799	Append4800	Append4803	Append4816	Append4822	Append4824	Prepend4826	Append4830	Append4831	Append4842	Prepend4845	Prepend4863	Append4865	Append4877	Append4879	Prepend4896	Append4913	Prepend4935	Prepend4943	Append4968	Append4970	Prepend4972	Prepend4974	Prepend4979	Append4988	Prepend5000	Append5004	Prepend5007	Append5014	Prepend5018	Prepend5020	Prepend5032	Prepend5062	Prepend5067	Append5068	Prepend5078	Append5085	Prepend5103	Append5114	Prepend5125	Prepend5130	Prepend5131	Prepend5147	Append5157	Append5160	Append5161	Append5165	Append5208	Append5212	Prepend5221	Append5229	Prepend5238	Prepend5245	Prepend5246	Append5250	Append5252	Append
