# Supplementary material for: The Exploration of Novel Regulatory Relationships Drives Haloarchaeal Operon-Like Structural Dynamics over Short Evolutionary Distances
Source: Microorganisms. 2020 Nov 30;8(12):1900. doi: 10.3390/microorganisms8121900 (PMC7760734; doi:10.3390/microorganisms8121900)
Supplement: Supplementary file 1 [file microorganisms-08-01900-s001.zip › S8_DisagreementWithPhylogeny.pdf]

S10: Operon Topology disagreement with Phylogeny

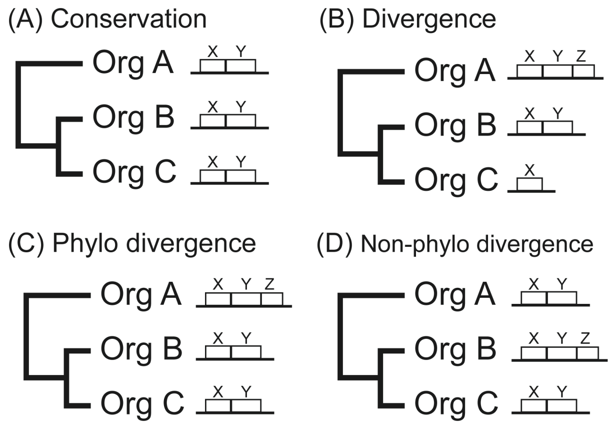

A theoretical sequence-divergence based phylogenetic tree of organisms A, B, and C is compared to an analogous operon contained in each organism. (A), (B) and (C) are all reflective of cases that agree with the phylogeny: in (A) the operon is topologically conserved despite overall organismal changes (the operon evolves more slowly than the host species), in (B) the operon diverges faster than the overall species evolution, and in (C) the operon topology matches the topological structure of the phylogeny. In (D) we see an example of divergence that does not match the phylogeny: Organisms A and C have more topologically similar operons, though they are more distantly related than organisms B and C. In example D, we might postulate that the state of the this operon in the last common ancestor of A,B, and C as well as the last common ancestor of B and C contained only genes X and Y, and following the speciation event of B and C, the operon has evolved to include the gene Z in organism B, but not in organism C.
