# Supplementary material for: The Exploration of Novel Regulatory Relationships Drives Haloarchaeal Operon-Like Structural Dynamics over Short Evolutionary Distances
Source: Microorganisms. 2020 Nov 30;8(12):1900. doi: 10.3390/microorganisms8121900 (PMC7760734; doi:10.3390/microorganisms8121900)
Supplement: Supplementary file 1 [file microorganisms-08-01900-s001.zip › S12_Cluster_distance_conservation.pdf]

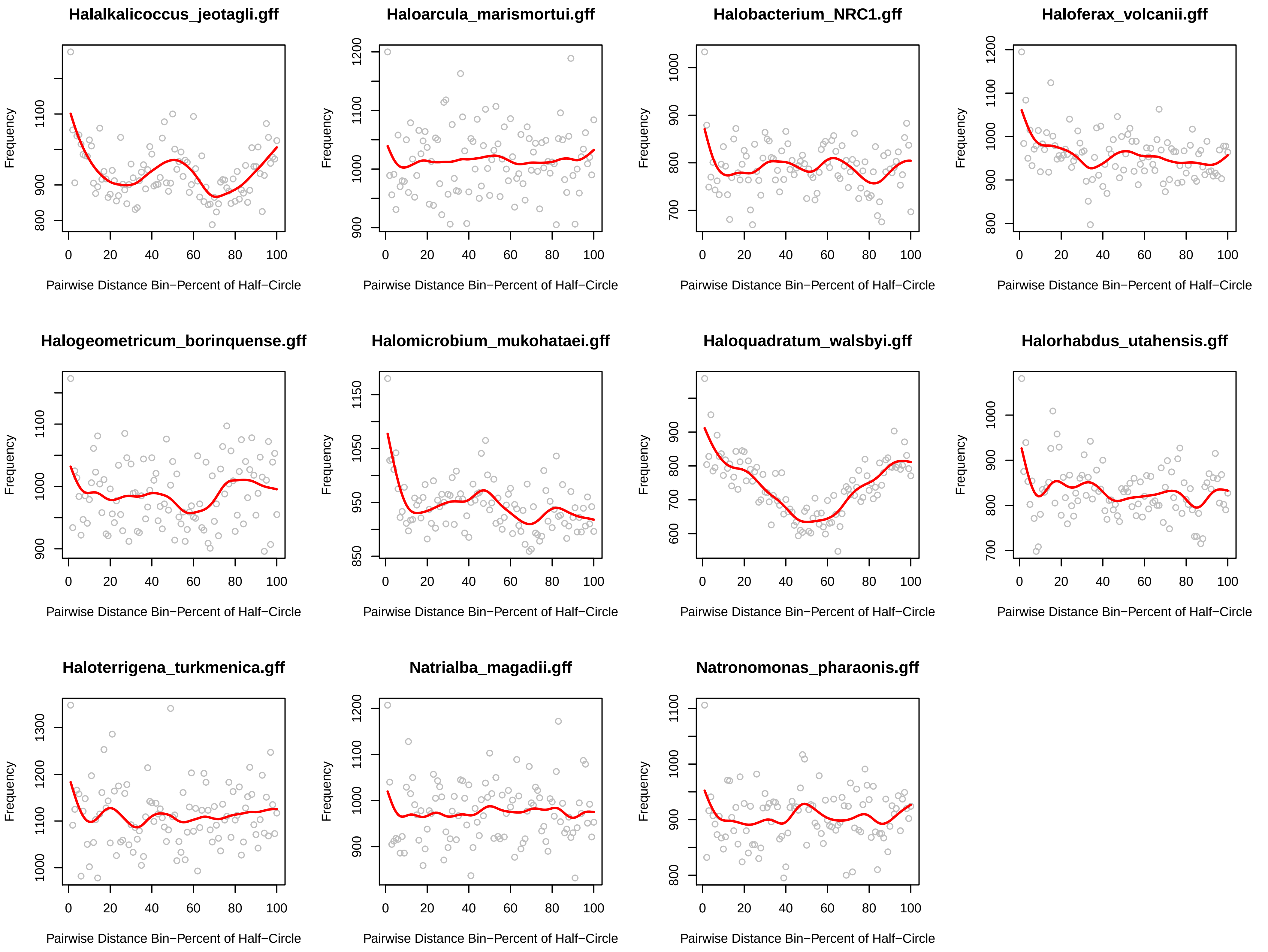
Halalkalicoccus_jeotagli.gff
Haloarcula_marismortui.gff
Halobacterium_NRC1.gff
Haloferax_volcanii.gff
Halogeometricum_borinquense.gff
Halomicrobium_mukohataei.gff
Haloquadratum_walsbyi.gff
Halorhabdus_utahensis.gff
Haloterrigena_turkmenica.gff
Natrialba_magadii.gff
Natronomonas_pharaonis.gff
Frequency
Pairwise Distance Bin−Percent of Half−Circle
0
20
40
60
80
100
600
700
800
850
900
950
1000
1050
1100
1150
1200
1300
