# Supplementary material for: The Exploration of Novel Regulatory Relationships Drives Haloarchaeal Operon-Like Structural Dynamics over Short Evolutionary Distances
Source: Microorganisms. 2020 Nov 30;8(12):1900. doi: 10.3390/microorganisms8121900 (PMC7760734; doi:10.3390/microorganisms8121900)
Supplement: Supplementary file 1 [file microorganisms-08-01900-s001.zip › S13_JCE_Pipeline_Instructions_v3.pdf]

JCE Pipeline Instructions

Written by Ariana Cisneros
Modified by Andrew Yao

-=-

These instructions describe the installation and use of a set of preprocessing scripts that create input files required for comparative genomic context analysis of orthologous gene clusters using JContextExplorer (Seitzer 2013). These instructions detail an installation of the pipeline and its dependencies on the **MacOS**. However, a user should be able to install and execute the pipeline on any Linux distribution provided the required dependencies are installed. It is likely that the installation will be very similar for other operating systems.

This pipeline executes a collection of scripts that process a directory of Genbank (.gbff) files into the input required for JContextExplorer (JCE). The scripts invoke and compute an all-by-all BLAST (Camacho 2009) search of protein sequences to identify putative homologous proteins. These results are filtered and clusters of putatively orthologous genes are identified by Ortho-MCL (Enright 2002). This output is subsequently filtered and new cluster and .gff files are created for input into JCE.

The pipeline attempts to parallelize computational steps using GNU parallel when possible (Tange 2018). Scripts automatically detect the number of CPU cores available and allocate the maximum number of cores to the respective process.

Prerequisites: Linux/Unix system (MacOS)

Dependencies:

1. Xcode
2. Homebrew
3. Perl (Bio::Perl libraries)
4. R
5. Blast command line tools
6. MCL-edge
7. parallel GNU
8. Java
9. JContextExplorer

Operating Procedure (If you have all of the dependencies installed already skip to step 4).

1. **Installing Dependencies:**
    - **1.1.** Ensure Xcode developer tools are installed via the App Store.
        - **1.1.1.** If your Mac does not support the current Xcode distribution.
            - **1.1.1.1.** Find your MacOS version: *Do: (Toolbar) Apple —> About this Mac —> write down the Version # (ie. Version 10.XX.X)*

1.1.1.2. Find the version that is compatible with your version of MacOS (https://developer.apple.com/library/archive/technotes/tn2456/_index.html)

1.1.1.3. Go to: *https://developer.apple.com/*

1.1.1.4. Login with your Apple ID and navigate to "Account" —> Downloads —> More —> Download the version of Xcode that is compatible with your version of MacOS

1.2. Homebrew

1.2.1. Install Homebrew *(If you already have Homebrew installed go to step 1.3)*

1.2.1.1. In the terminal type:

```
/bin/bash -c "$(curl -fsSL https://raw.githubusercontent.com/Homebrew/install/master/install.sh)"
```

1.3. Perl & Bio::Perl libraries

1.3.1. Install cpanm

1.3.1.1. In the terminal type:

```
brew install cpanm
```

1.3.1.2. ***Homebrew likes to frequently update - do not be alarmed that before you run a 'brew' command Homebrew will first take time to update (sometimes multiple times a day).***

1.3.2. Install local::lib

1.3.2.1. In the terminal type:

```
cpanm --local-lib=~/perl5 local::lib && eval $(perl -I ~/perl5/lib/perl5/ -Mlocal::lib)
```

1.3.3. Install Bio::Perl

1.3.3.1. In the terminal type (you might be prompted for your password):

```
sudo cpanm Bio::SeqIO
sudo cpanm Bio::SearchIO
sudo cpanm File::Basename
sudo cpanm Bio::Perl
```

1.3.3.1.1. You may be prompted to force install some of the packages, to do so you may use (you might be prompted for your password again)

```
sudo cpanm Package::Package -force
```

1.4. R (Version 4.0 or later)

1.4.1. Install R

1.4.1.1. In the terminal type (if you are installing R for the first time):

```
brew install R
```

(If you are updating):

```
brew upgrade R
```

1.5. Blast command line tools

1.5.1. Access https://www.ncbi.nlm.nih.gov/books/NBK279671/ and download the version of Blast+ that is compatible with your system *(MacOS users should download the .dmg version)*.

1.5.1.1. (MacOS) Users will have to right click -> open the .dmg file to execute the file for installation.

1.5.1.2. Ensure blast installation was successful by typing ‘blastp’ in any terminal window. Successful installation should result in this output:

```
$ blastp
BLAST query/options error: Either a BLAST database or subject sequence(s) must be specified
Please refer to the BLAST+ user_manual.
```

1.5.2. Correct path to blast commands in ‘legacy_blast.pl' script.

1.5.2.1. (MacOS): In terminal (you will be prompted for your password):

```
sudo nano /usr/local/ncbi/blast/bin/legacy_blast.pl
```

1.5.2.2. Change line 85:

```
use constant DEFAULT_PATH => "/usr/bin";
```

to

```
use constant DEFAULT_PATH => “/usr/local/ncbi/blast/bin";
```

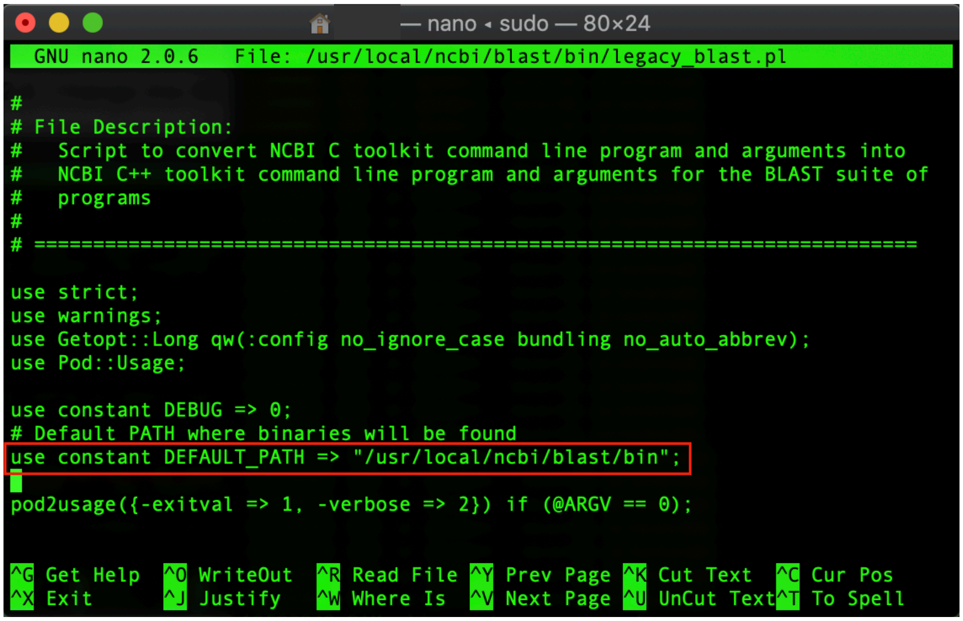

1.5.2.3. Save and close file (press ‘*control* + *x*’, when prompted to save press ‘*y*’ then ‘*return*’).

1.6. MCL-edge

1.6.1. Access https://micans.org/mcl/src/ and download the latest version of MCL-edge "mcl-latest.tar.gz".

1.6.2. In the terminal, open the tar ball (.tar file) by typing:

```
tar -xvf /path/to/mcl_latest.tar
```

1.6.3. Change directory to the unzipped folder containing the downloaded and uncompressed items from step 3.5.2. Then run sequentially:

```
./configure %% make %% sudo make install
```

1.6.3.1. Test that mcl installed correctly by typing 'mcl' into any Terminal window. Successful installation should results in this output:

```
$ mcl
[mcl] usage: mcl <-|file name> [options], do 'mcl -h' or 'man mcl' for help
```

1.7. Parallel GNU

1.7.1. In the terminal type:

```
wget https://ftpmirror.gnu.org/parallel/parallel-20200622.tar.bz2
bzip2 -dc parallel-20200622.tar.bz2 | tar xvf -
cd parallel-20200622
./configure && make && sudo make install
```

1.7.1.1. Test that parallel installed correctly by typing 'parallel' into any Terminal window. Successful installation should results in this output:

```
$ parallel
parallel: Warning: Input is read from the terminal. You are either an expert
parallel: Warning: (in which case: YOU ARE AWESOME!) or maybe you forgot
parallel: Warning: ::: or :::: or -a or to pipe data into parallel. If so
parallel: Warning: consider going through the tutorial: man parallel_tutorial
parallel: Warning: Press CTRL-D to exit.
```

Press '*control+D*' to exit.

1.8. JContextExplorer

1.8.1. Navigate to https://facciotti.bme.ucdavis.edu/resources_data/software/ and download the JContextExplorer 3.0 JAR file.

1.9. Java

1.9.1. Navigate to https://www.java.com/ES/download/ to download Java.

1.9.2. If you are unsure if you have java; type 'java' in any terminal window. If you do not have java installed you will see a "java: command not found" and will need to complete step 3.9.1.

2. Download JCE Pipeline

2.1. Download and unzip the JCE Pipeline directory to the directory of your choice: https://facciotti.bme.ucdavis.edu/resources_data/software/ .

3. Genome Files

3.1. Download Genome Files (XX_gbff.gz) into a single directory within the JCE Pipeline directory for comparison. Gbff files can be downloaded from: https://ftp.ncbi.nlm.nih.gov/genomes/genbank/

3.2. Export path to directory containing Genome Files by typing the following command in Terminal

```
export GENBANK_FILES=/PATH/TO/FOLDER/CONTAINING/GBFF_FILES
****there should be no trailing '/' at the end of the export PATH******
```

3.3. For example if your Genomes are in a folder called Example_Genomes in your Downloads directory:

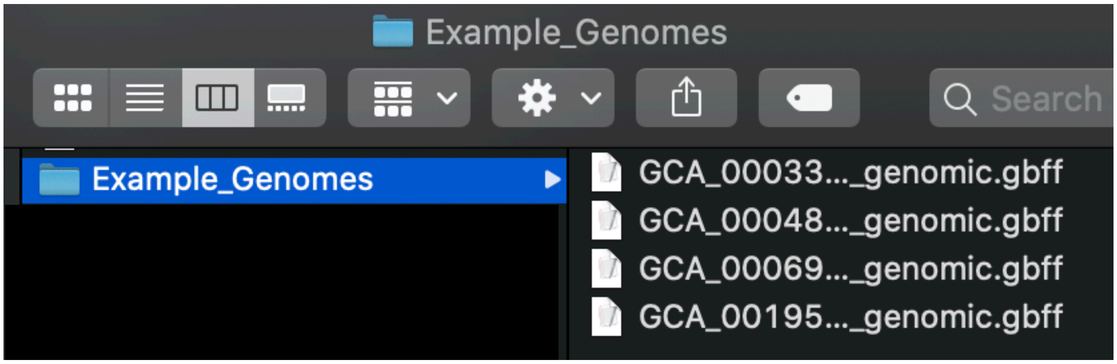

The export path would be:

```
export GENBANK_FILES=~/Downloads/Example_Genomes
```

3.3.1. **Hint** You can copy ('command + c') a folder in Finder and paste ('command + v') it into terminal to quickly type out the entire path of a directory

4. Preparing to run JCE Pipeline

4.1. Navigate to the 'JCE_HC_PIPELINE' directory that contains the 'JCE_BASE_DIRECTORY' and 'Wrapper.sh' file and enable the executable file:

```
chmod +x Wrapper.sh
```

5. **Running JCE Pipeline**
    5.1. In the same directory run the pipeline by typing:

    ```
    ./Wrapper.sh
    ```

    5.2. You will be prompted for a name for the run. This can be any string without spaces i.e.: HALO_FOR_JCE
    5.3. You may see the following warning message (sometimes multiple times):

```
--------------------- WARNING ---------------------
MSG: Did not define the number of conserved matches in the HSP; assuming conserved == identical (239)
---------------------------------------------------
```

These messages can be ignored.

6. **JCE HC Pipeline Output**
    6.1. The files you will upload into JCE will be in the newly named directory within the ‘OUTPUT_FOR_JCE’ subdirectory of your named run directory.
7. **Uploading HC Pipeline Output into JCE**
    7.1. Open JCE in the terminal by typing:

    ```
    java -jar /PATH/TO/JContextExplorer30.jar
    ```

    or

    ```
    java -Xmx1024M -jar /PATH/TO/JContextExplorer30.jar
    ```

    7.2. In the JCE GUI select Genomes -> New Genome Set

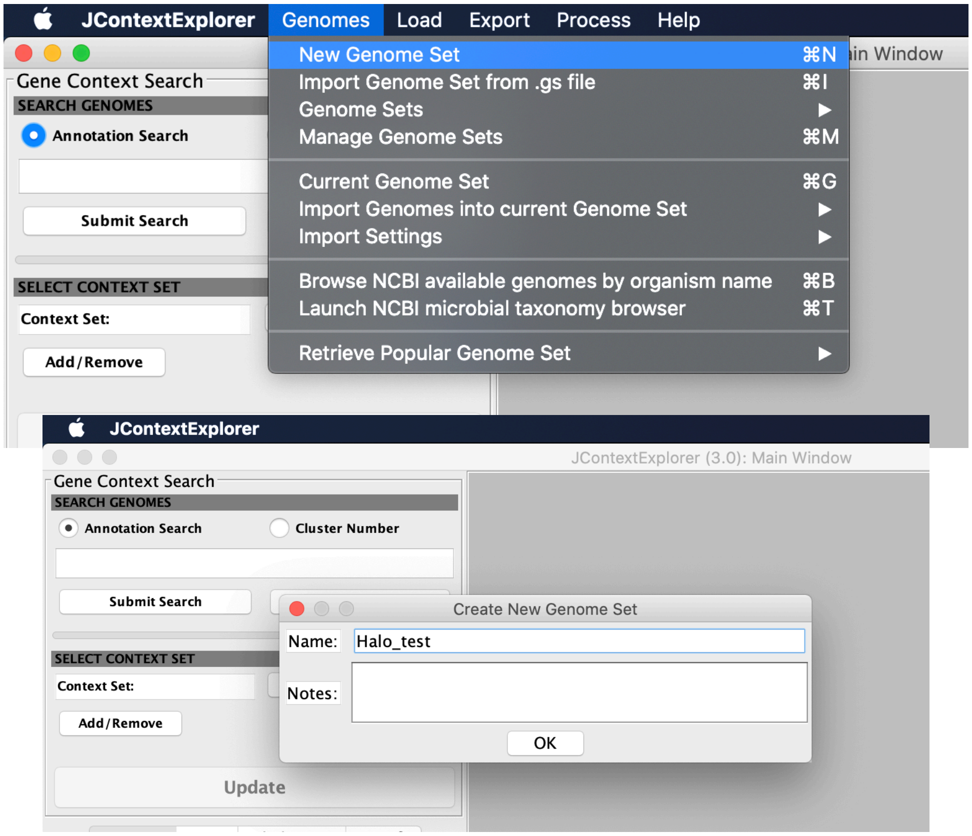

7.3. Then provide a name for the genome set and press ‘OK’.
7.4. Then select Genomes -> Import Genomes into current Genome Set ->

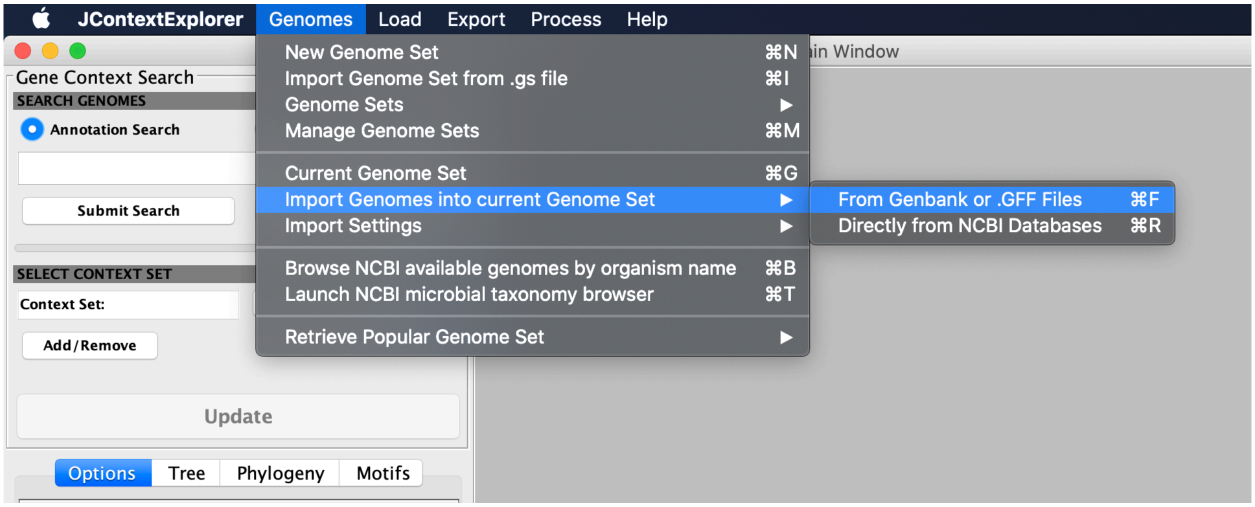

From Genbank or .GFF Files (or command + f).

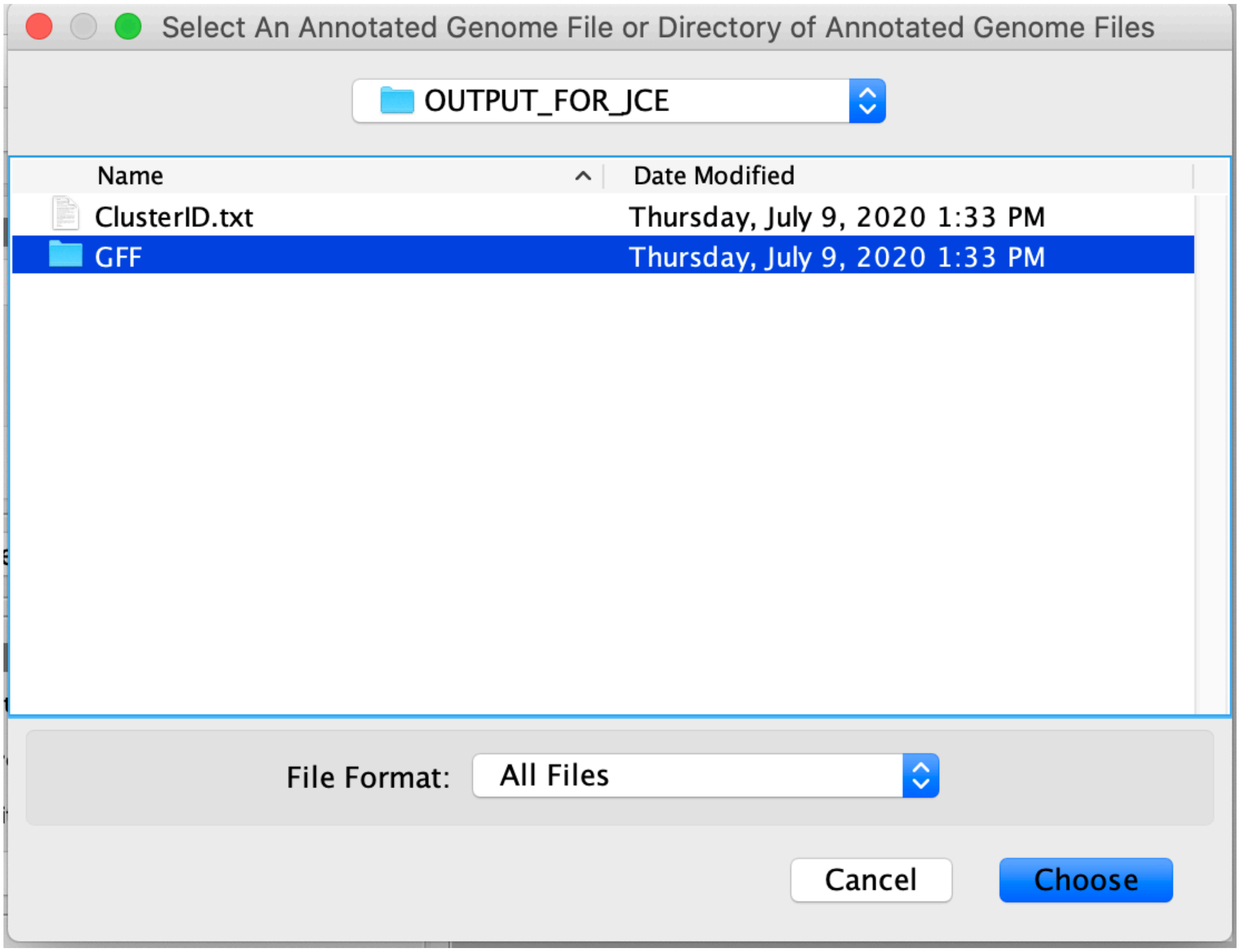

7.5. Navigate to your OUTPUT_FOR_JCE directory from step 8.1 and select the GFF directory. Press Choose to select this folder.

7.6. Next select Load -> Homology Clusters (or command + u).

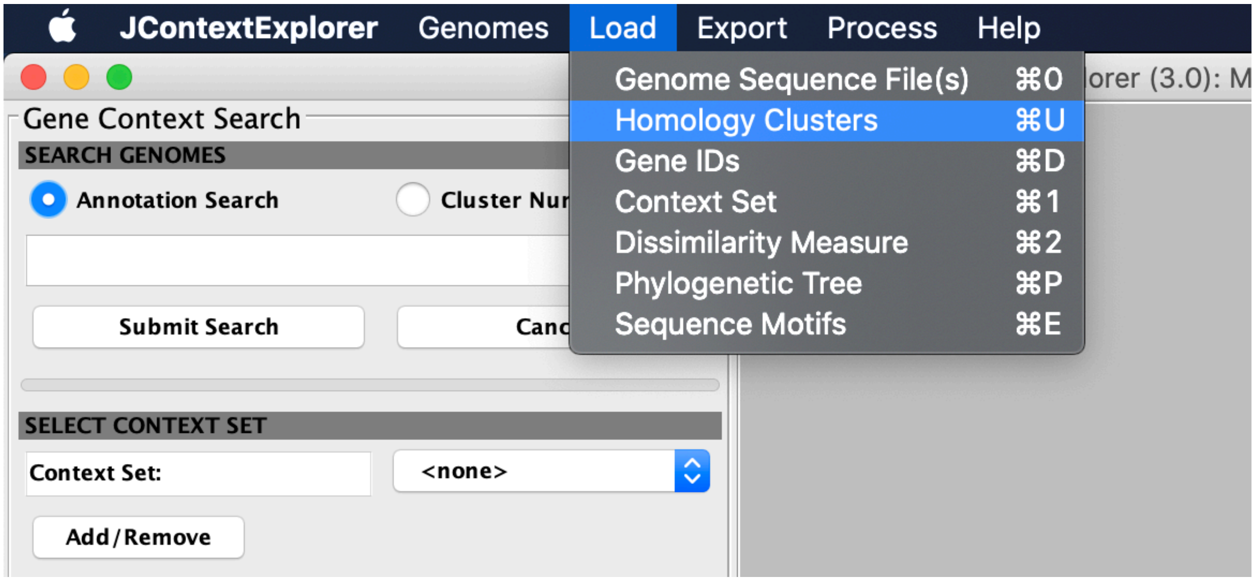

7.7. Select the ‘ClusterID.txt’ file and press Choose.

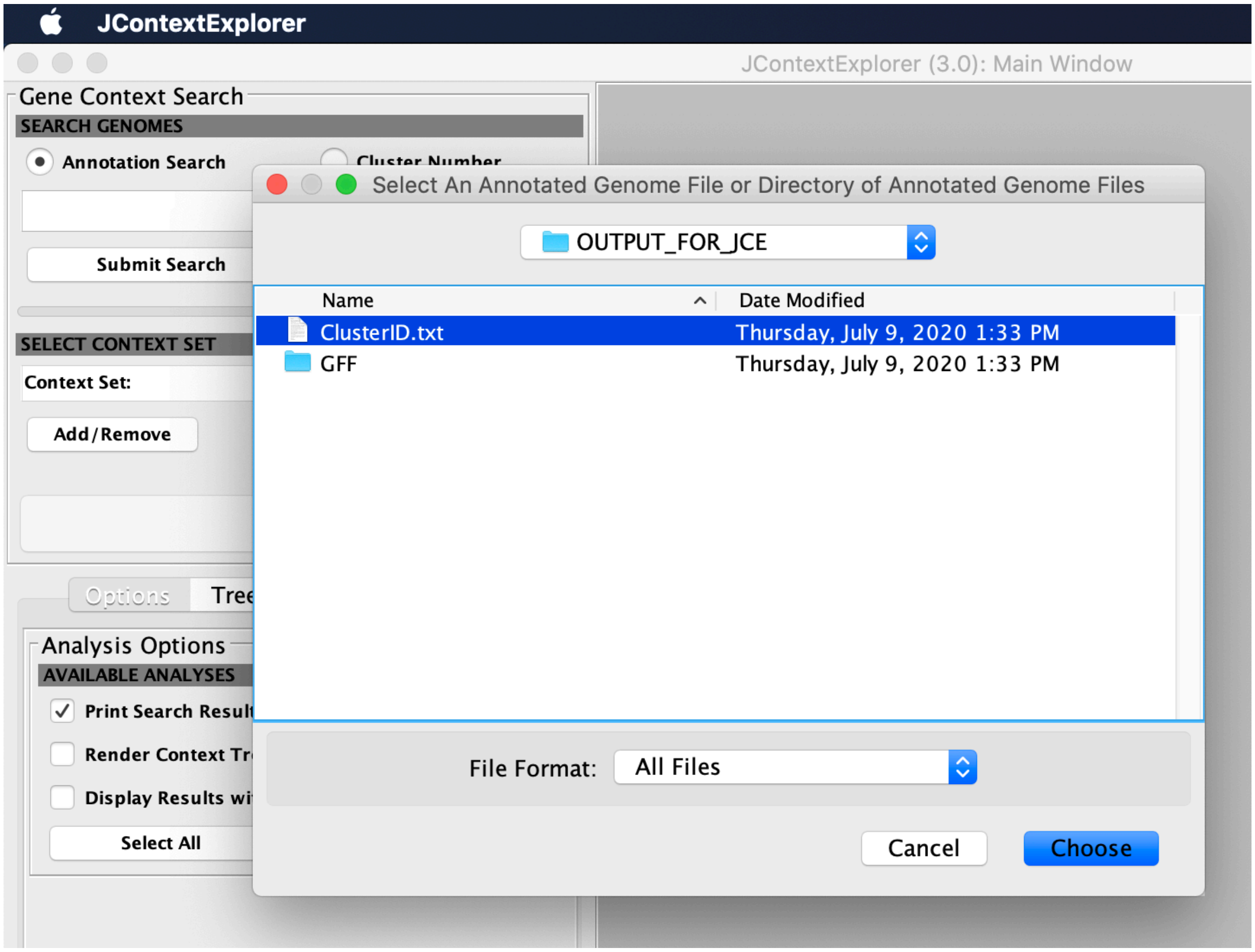

7.8. Now you can explore your Genome Contexts and Clusters in JCE! (The JCE Manual can be found here: **https://facciotti.bme.ucdavis.edu/files/2011/06/UsersManual_v2.pdf**)

-=-

1. Seitzer, P., Huynh, T. A. & Facciotti, M. T. JContextExplorer: a tree-based approach to facilitate cross-species genomic context comparison. *BMC Bioinformatics* **14**, 18 (2013).

2. Camacho, C. *et al.* BLAST+: architecture and applications. *BMC Bioinformatics* **10**, 421 (2009).

3. Enright, A. J. An efficient algorithm for large-scale detection of protein families. *Nucleic Acids Research* **30**, 1575–1584 (2002).

4. Tange, O. *Gnu Parallel 2018*. (Zenodo, 2018). doi:10.5281/ZENODO.1146014.
