# Supplementary figures and images for: The Exploration of Novel Regulatory Relationships Drives Haloarchaeal Operon-Like Structural Dynamics over Short Evolutionary Distances
Source: Microorganisms. 2020 Nov 30;8(12):1900. doi: 10.3390/microorganisms8121900 (PMC7760734; doi:10.3390/microorganisms8121900)

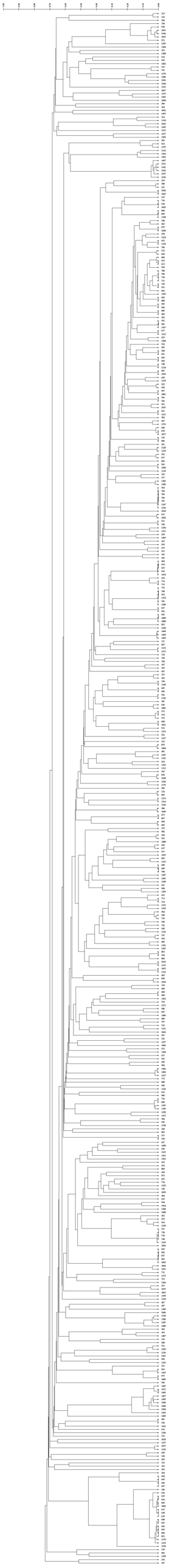

Supplement: Supplementary file 1 [file microorganisms-08-01900-s001.zip › S11_OperonForest.pdf]
